# DNA barcoding of marine teleost fishes (Teleostei) in Cebu, the Philippines, a biodiversity hotspot of the coral triangle

**DOI:** 10.1038/s41598-023-41832-9

**Published:** 2023-09-08

**Authors:** Wen-Chien Huang, Florence Chan Evacitas, Rodulf Anthony Balisco, Cleto L. Nañola, Tak-Kei Chou, Wei-Cheng Jhuang, Chih-Wei Chang, Kang-Ning Shen, Kwang-Tsao Shao, Te-Yu Liao

**Affiliations:** 1 Doctoral Degree Program in Marine Biotechnology, National Sun Yat-sen University, Kaohsiung, Taiwan; 2 Doctoral Degree Program in Marine Biotechnology, Academia Sinica, Taipei, Taiwan; 3 Department of Biology and Environmental Science, University of the Philippines Cebu, Cebu City, Philippines; 4 Department of Oceanography, National Sun Yat-sen University, Kaohsiung, Taiwan; 5 College of Fisheries and Aquatic Sciences, Western Philippines University, Puerto Princesa, Philippines; 6 Department of Biological Sciences and Environmental Studies, University of the Philippines Mindanao, Davao City, Philippines; 7 Marine Ecology and Conservation Research Center, National Academy of Marine Research, Kaohsiung, Taiwan; 8 Institute of Marine Ecology and Conservation, National Sun Yat-sen University, Kaohsiung, Taiwan; 9 National Museum of Marine Biology and Aquarium, Pingtung, Taiwan; 10 Graduate Institute of Marine Biology, National Dong Hwa University, Pingtung, Taiwan; 11 Biodiversity Research Center, Academia Sinica, Taipei, Taiwan

**Keywords:** Ichthyology, Eukaryote, Biodiversity

## Abstract

A morphology-based barcoding library of market teleost fishes (Teleostei) in Cebu is built based on cytochrome c oxidase subunit I (*COI*) sequences and voucher specimens which aimed to establish a reliable reference of frequently traded fishes in the province, a biodiversity hotspot at the center of the Philippine archipelago. A total of 1721 specimens were collected from 18 fish markets and landing sites around the province, in which 538 specimens were sequenced belonging to 393 species from 229 genera, 86 families, and 37 orders. Most speciose families are coral reef or reef-related shallow-water species. Twelve species from 11 families are newly recorded in the Philippine waters, among which 7 species are deep-sea inhabitants, while 3 species have expanded their distribution range. Only 20 taxa could not be identified to the species level due to the difficulty in morphological examinations, absence of matched reference sequences in online databases, and/or problematic species awaiting further studies. This first comprehensive DNA barcoding survey of Cebu fishes can facilitate further taxonomic research as well as the conservation and management of fisheries in the Philippines.

## Introduction

The DNA-based identification method has become increasingly popular in various taxa^1^. This can be attributed to the ease of using molecular markers (e.g., cytochrome oxidase subunit I, *COI*; cytochrome b; 12S ribosomal RNA) and the reduced reliance on taxonomic keys, which can be challenging for non-taxonomists^2^. DNA barcode reference libraries for bony fishes have been established in various geographic regions, aiding in the enrichment of local fish species lists. Starting from earlier Australian studies (e.g.^3^) and extending to more recent ones (e.g.^4–6^), the increasing utilization of DNA barcoding has expanded its scope from identifying species of conservation concern to revealing previously unrecognized cryptic species and accurately mapping the distribution ranges of known species. Accurate species identification is vital to ensure the successful management of fish stocks and will bring insight into processes maintaining marine biodiversity^7^.

In the Philippines, DNA barcoding has remained an underutilized tool in ichthyofaunal studies. This method has been increasingly used for identification and authentication of component species in commercial fishery products to ensure traceability and food safety (e.g.^8,9^); however, few studies have reported its use in the taxonomy, phylogeny, specimen-based identification, or to enrich species listing of marine fishes. To date, fish DNA barcoding studies in the country have primarily concentrated on major freshwater lakes^10–13^, while its application to marine ichthyofaunal diversity has been focused on specific groups of taxa with commercial importance, such as sardines (Dorosomatidae)^14,15^, groupers (Epinephelidae)^16^, trevallies (Carangidae) and snappers (Lutjanidae)^17^ which were collected from different sites in the country or from a specific area such as in the Cuyo Island, Palawan or in Northern Mindanao. Similarly, Willette & Padin^18^ and Torres & Santos^19^ used DNA barcodes of three marine *Caranx* species from the freshwater environment to resolve the identification and establish their phylogenetic relationship with extant marine species. Its recent use in specimen-based identification was reported by Bemis et al.^5^, who established the most comprehensive DNA barcode reference library of Philippine marine fishes based on 2,525 voucher specimens representing 984 species, significantly enhancing the coverage and utility of the method in the country.

The Cebu archipelago is located in the epicenter of marine biodiversity and its coasts harbor well-developed fringing reefs, strips of mangrove forests, and seagrass beds of varying patch sizes. Furthermore, the narrow and deep Tañon and Cebu Straits located in southern Cebu are the main channels for water exchanges in the Central Visayas, offering specific habitats for deep-sea fauna^20^. The variety of these habitats and ecosystems has made Cebu waters one of the seven most important marine biodiversity areas in the Coral Triangle^21^, and one of the most productive areas in the Philippines that have long served as a major fishing ground and source of fisheries revenues^22^. However, the presence of important coastal habitats and ecosystems amidst expanding human settlements has turned Central Visayas into a hotspot of overexploitation, thereby impacting species diversity in the region^23,24^.

Since the early 2000s, there has been a decrease in catch volume and changes in species composition (disappearance of some species and rise in number of invertebrates) landed in Cebu and surrounding waters due to changes in fishing gears and the increasing fishing pressure. This situation has had an impact on the livelihoods of artisanal fishermen^20,23^. Hence, the waters surrounding Cebu have become the priority areas for conservation, particularly for the reef fishes^20^. Nevertheless, previous fish species listings in Cebu waters were either site-specific (e.g.^25,26^) or focused on marine protected areas (e.g.^27,28^). While Bemis et al.^5^ have created the first DNA barcode reference library of marine market fishes in the Philippines, there is still a need to enhance comprehensive regional information on species composition through broader sampling coverage. As such, this study was conducted to identify the marine ichthyofauna of Cebu using DNA barcoding and voucher specimens. The aim is to expand the DNA barcode library of Philippine marine fishes and create a comprehensive fish species checklist for Cebu that encompasses additional economically important species, new species records, and the detection of cryptic species.

## Results

### Species identification and composition

**Figure 1:**
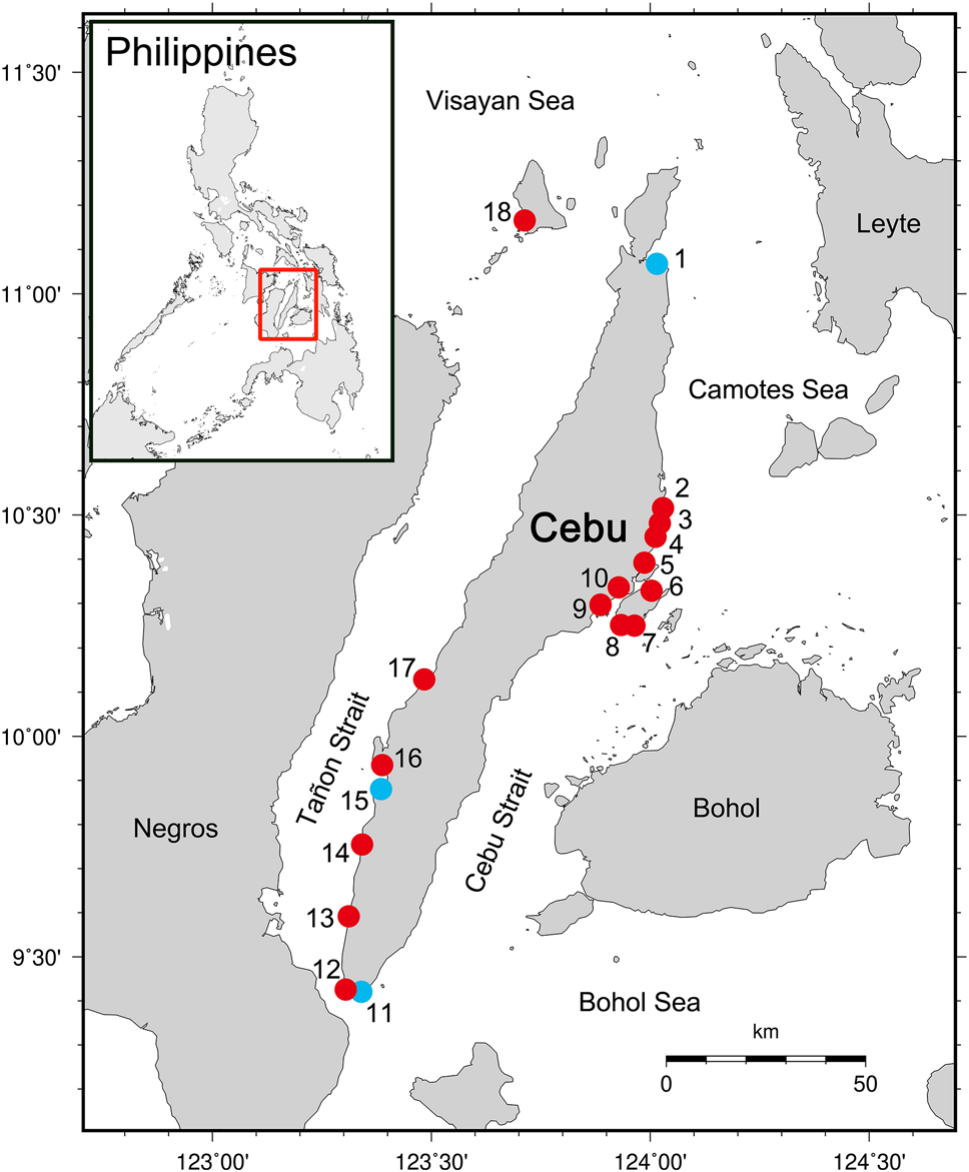
Map of 18 sampling localities in Cebu, the Philippines. Blue circles for sampling localities of the 23 deep-sea species in Table 5. Details of sampling localities in Supplementary Table S1. The map was generated by GMT version 5.4.5 (http://gmt.soest.hawaii.edu/doc/5.4.5/gmt.html) and modified by Photoshop CS5.

**Figure 2:**
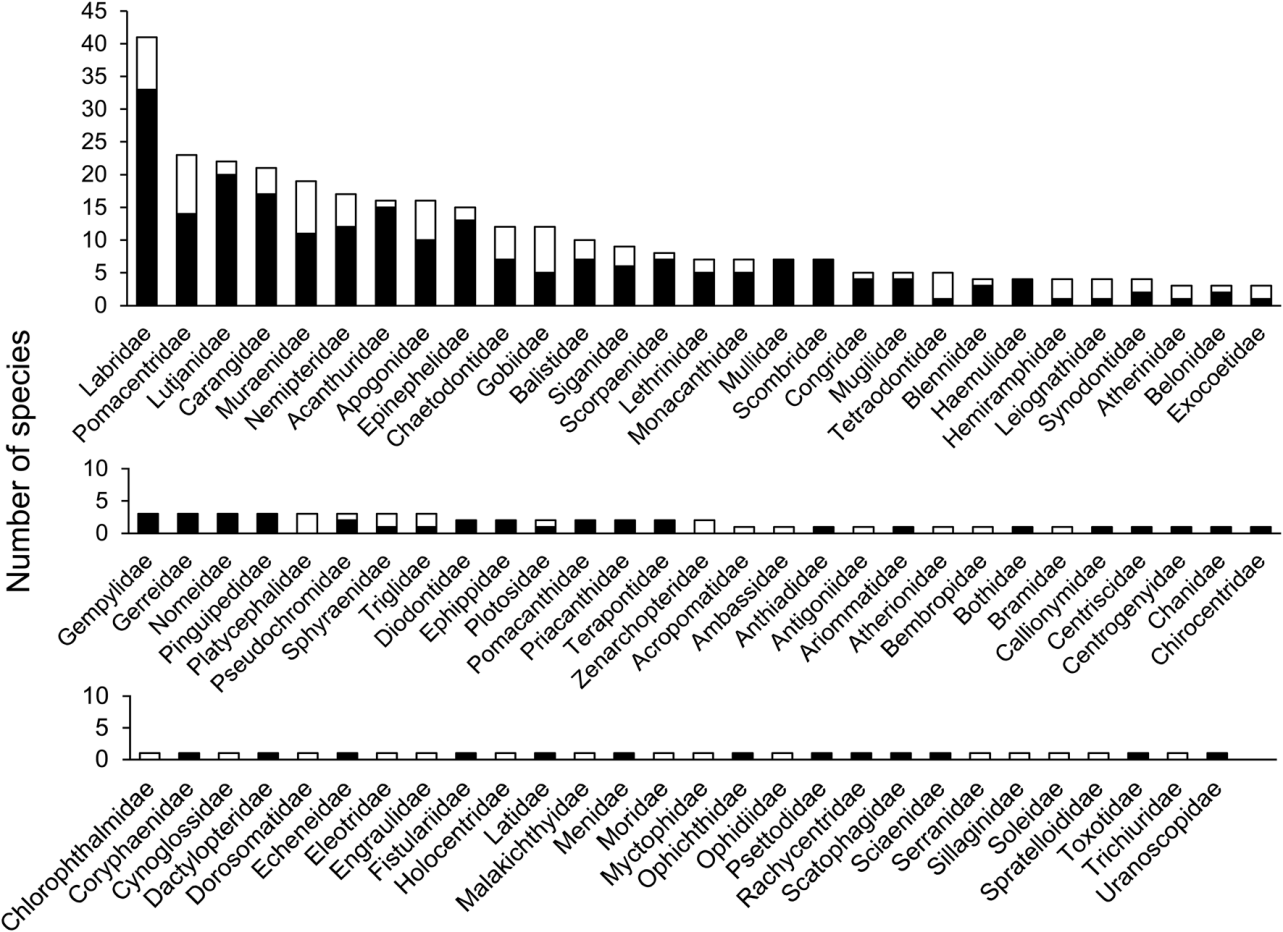
Number of species in the 86 families collected in this study. Black bars for species recorded in Bemis et al.^5^; white bars for species not included in Bemis et al.^5^.

A total of 1721 specimens were collected from Cebu (Fig. 1, Supplementary Table S1), in which 538 specimens were sequenced, representing 393 species in 229 genera, 86 families, and 37 orders (Supplementary Fig. S1, Table S2). The length of obtained *COI* sequences ranged from 507 to 691 bp without any indel or stop codon. Twenty taxa could not be annotated at the species level because of any one or the combination of the following reasons: (1) species with difficulty in morphological identification; (2) species without corresponding blast result of genetic similarity higher than 98%; (3) species with blast results of genetic similarities higher than 98%, but the sequence from database was apparently misidentified; and (4) species may represent undescribed taxa awaiting further studies (Table 1). Twelve families contained ≥ 10 species and they accounted for 57.0% of the total species identified in this study, including Labridae (41 species, 10.4%), Pomacentridae (23 species, 5.9%), Lutjanidae (22 species, 5.6%), Carangidae (21 species, 5.3%), Muraenidae (19 species, 4.8%), Nemipteridae (17 species, 4.3%), Acanthuridae (16 species, 4.1%), Apogonidae (16 species, 4.1%), Epinephelidae (15 species, 3.8%), Chaetodontidae (12 species, 3.1%), Gobiidae (12 species, 3.1%), and Balistidae (10 species, 2.5%) (Fig. 2). Twelve species from 11 families were newly recorded in the Philippines, and three of these species exhibited expanded distribution ranges (Table 2). According to IUCN Red List status, none of the species involved in this study were categorized as Critically Endangered (CR) and Endangered (EN). Only *Epinephelus fuscoguttatus* (0.3% of all species) was considered Vulnerable (VU) species, while *Choerodon schoenleinii* and *Sardinella lemuru* (0.5%) were Near Threatened (NT) species. Two hundred and seventy-seven species (70.5%) were Least Concern (LC), 106 species (27.0%) were Not Evaluated (NE), and seven species (1.8%) were Data Deficient (DD) (Supplementary Table S2).

**Table 1:** List of the 20 taxa that cannot be identified to the species level in this study.

Family	Scientific name	Accession number
Chlorophthalmidae	*Chlorophthalmus* sp.	OR113865
Congridae	*Rhynchoconger* sp.	OR113871
Cynoglossidae	*Cynoglossus* sp.	OR113873
Gobiidae	*Paratrypauchen* sp.	OR113899
Labridae	*Iniistius* sp.	OR114219, OR114220
	*Oxycheilinus* sp.	OR113940
	*Pteragogus* sp.	OR113943, OR114221
Leiognathidae	*Eubleekeria* sp.	OR113951
Mugilidae	*Planiliza* sp.	OR113987
Muraenidae	*Gymnothorax* cf. *chilospilus*	OR114003, OR114184, OR114185
	*Gymnothorax* cf. *cribroris*	OR114004
Nemipteridae	*Nemipterus* sp.	OR114265
Plotosidae	*Paraplotosus* sp.	OR114050
Pomacentridae	*Abudefduf* sp.	OR114064
Pseudochromidae	*Labracinus* sp.	OR114088, OR114089
Serranidae	*Chelidoperca* sp.	OR114043
Sphyraenidae	*Sphyraena* cf. *jello*	OR114144
	*Sphyraena* sp.	OR114142
Spratelloididae	*Spratelloides* cf. *delicatulus*	OR114163
Zenarchopteridae	*Zenarchopterus* sp.	OR113756

**Table 2:** List of the 12 species newly recorded in the Philippines.

Family	Species	Original distribution range
Atherinidae	***Doboatherina duodecimalis***	Indo-West Pacific: Comoro, Madagascar, Seychelles, Sri Lanka, Thailand, Indonesia, Papua New Guinea, Vanuatu and New Caledonia
Bramidae	*Brama pauciradiata*	
Gobiidae	*Bathygobius coalitus*	
Malakichthyidae	*Verilus pacificus*	
Moridae	***Physiculus chigodarana***	Japan and Taiwan
Muraenidae	*Gymnothorax formosus*	
Nemipteridae	***Parascolopsis rufomaculata***	Northwestern Australia and southern Java, Indonesia
Ophidiidae	*Neobythites bimaculatus*	
Tetraodontidae	*Lagocephalus lagocephalus*	
	*Lagocephalus spadiceus*	
Trichiuridae	*Trichiurus nanhaiensis*	
Triglidae	*Satyrichthys laticeps*	

Species with distribution range expansion are in bold, and their original distribution ranges are shown.

### Genetic distance and species delimitation

**Figure 3:**
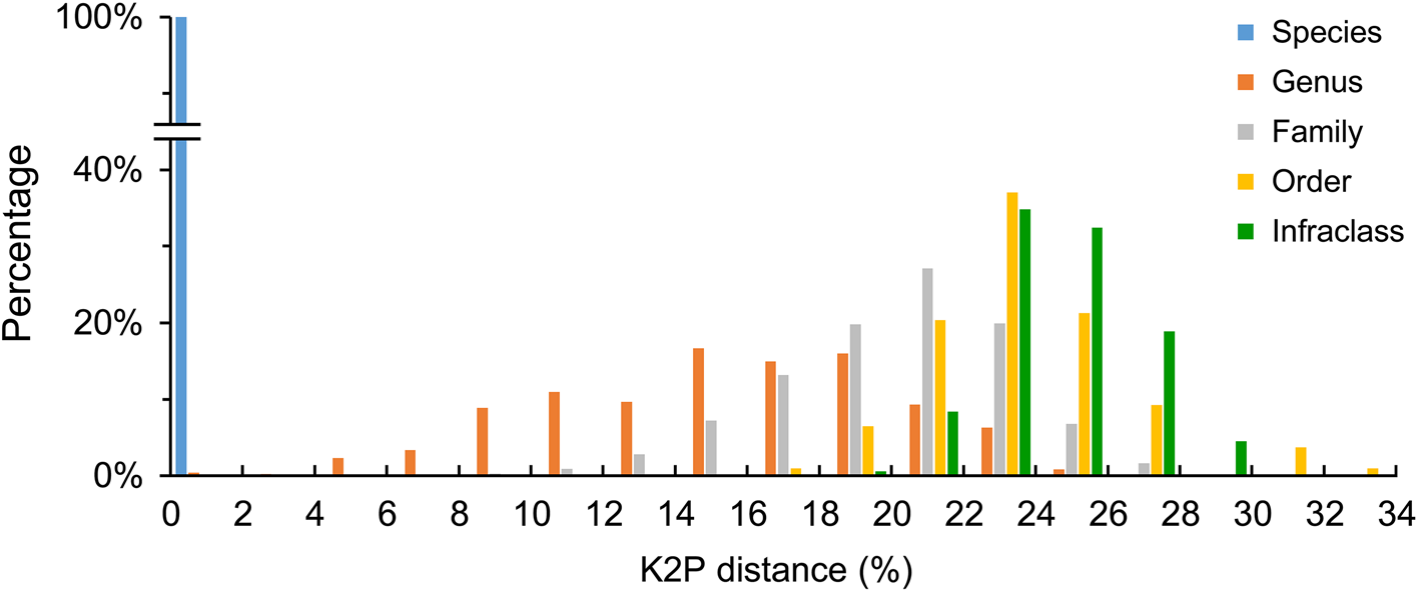
Distribution of K2P genetic distances at different taxonomic levels based on 507 bp of 538 *COI* sequences.

**Figure 4:**
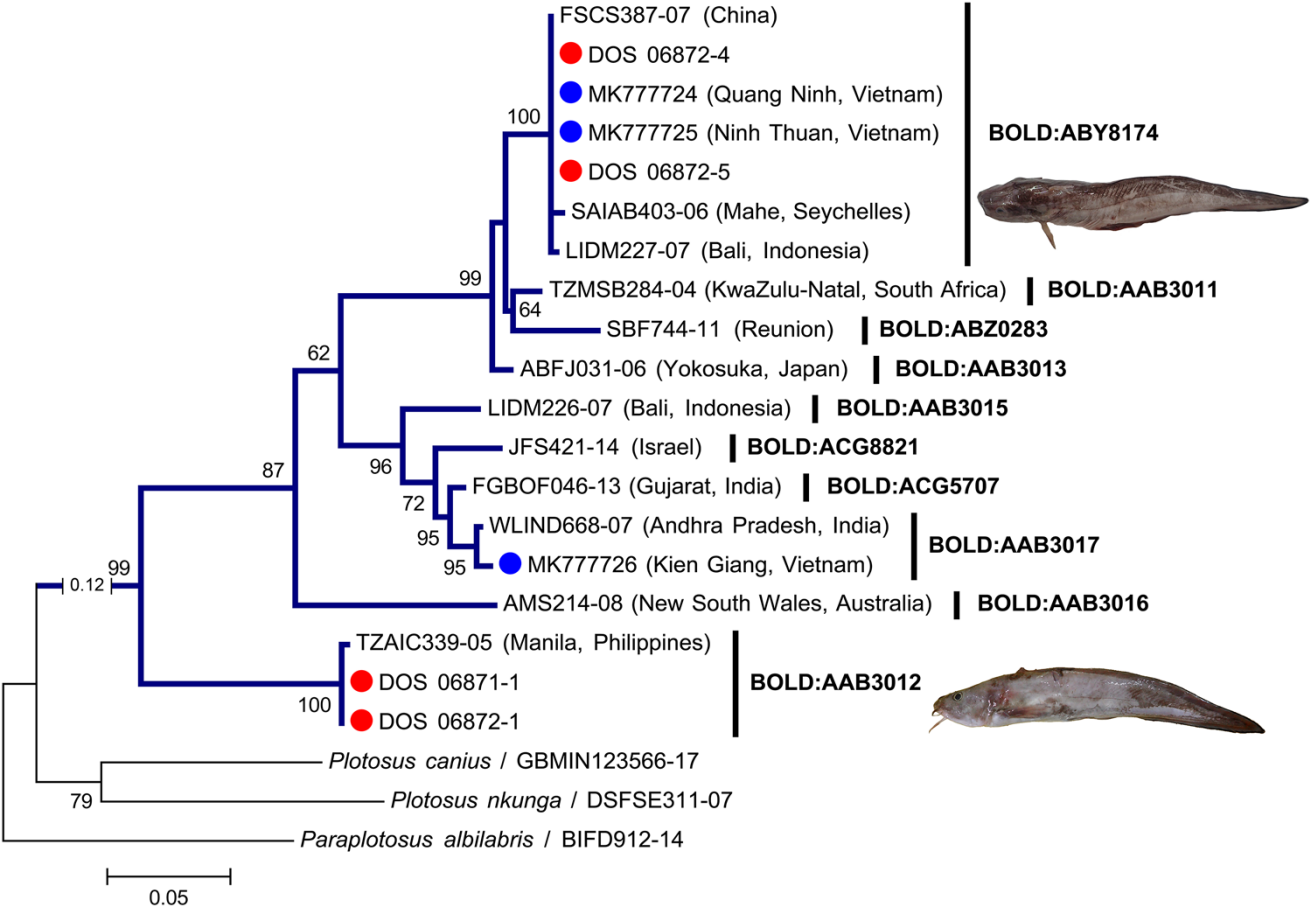
Maximum likelihood tree of selected *COI* sequences (433 bp) from 10 BINs under the species names *Plotosus lineatus* + *P. japonicus* on BOLD Systems. Tree reconstructed based on GTR + *Γ* + *I* model. Numerals beside the internal branches for bootstrap values and those lower than 50 not shown. *Plotosus canius*, *P. nkunga*, and *Paraplotosus albilabris* are outgroups. Blue and red circles represent sequences of two lineages from Vietnam^4^ and sequences from this study, respectively. Insets show photos of Cebu specimens of the two lineages.

After trimming, 507 bp of all sequences were used in data analyses. The K2P genetic distances revealed an increased trend at higher taxonomies, with the mean values of 0.26 ± 0.03 (standard error) %, 15.30 ± 0.22%, 20.08 ± 0.12%, 23.42 ± 0.26%, and 24.50 ± 0.08% in intra-specific, -generic, -families, -orders, and -intraclass levels, respectively (Fig. 3, Table 3). At the intra-specific level, *Plotosus lineatus* showed large genetic distances between two genetic lineages that were far beyond 2%, but no obvious morphological difference was found between specimens (mean intra-specific K2P distance 15.00 ± 1.90%, n = 4; Fig. 4). Although this rare case may imply the exposure of divergent evolutionary lineages or cryptic species that have yet to be described, we temporarily annotated it under the same species and excluded it from the calculation of intra-specific distance. By contrast, two species pairs had clearly distinguishable morphology but revealed very low genetic divergences, viz., *Abudefduf sexfasciatus* vs. *A. vaigiensis* (inter-specific K2P distance 0.30 ± 0.17%, n = 3) and *Arothron immaculatus* vs. *A. manilensis* (0.79 ± 0.39%, n = 2), which were retained in the analysis. Except for the aforementioned three cases, the barcoding data showed a maximum intra-specific K2P distance of 1.30 ± 0.40% (*Gymnothorax* cf. *chilospilus*) and a minimum inter-specific K2P distance of 3.25 ± 0.80% (*Satyrichthys welchi* vs. *S. laticeps*), indicating a discernible barcoding gap spanning the range of 1.31–3.24% K2P distance.

**Table 3:** Summary of genetic divergences within various taxonomic levels based on 538 *COI* sequences (507 bp). *Plotosus lineatus* sequences not included in the intra-specific distance calculation.

Comparisons within	Number of taxa	K2P distances (%)	
		Range	Mean ± SE
Species	102 Species	0.00–1.30	0.26 ± 0.03
Genus	64 Genera (228 species)	0.30–24.98	15.30 ± 0.22
Family	36 Families (179 genera)	7.81–28.74	20.08 ± 0.12
Order	21 Orders (70 families)	16.03–32.14	23.42 ± 0.26
Infraclass	1 Infraclass (37 orders)	18.66–32.11	24.50 ± 0.08

A total of 393 species were identified by the combination of morphology and blast results. Similarly, 379 existing BINs were recorded from BOLD Systems and 13 species represented unidentified BINs. Among all BINs, *Abudefduf sexfasciatus* vs. *A. vaigiensis* and *Arothron immaculatus* vs. *A. manilensis* were respectively designated in the same BINs, while *Plotosus lineatus* was represented by two BINs, resulting in a total of 392 BINs, which is one less than the total species number. The ABGD analysis revealed the same partition pattern of 392 OTUs with BINs. The ASAP showed 391 OTUs, which is very similar to BINs and ABGD, except for *Satyrichthys welchi* and *S. laticeps*, a species pair that had a K2P distance of 3.25 ± 0.80% but were deemed as the same OTU. The bPTP presented the most partitioned result that contained 420 OTUs.

The NJ tree based on 538 *COI* sequences revealed the monophyly in each species except for *Abudefduf sexfasciatus* vs. *A. vaigiensis* (Supplementary Fig. S2). However, 29 out of the 229 genera from 19 families were not monophyletic. We collected all sequences of these families from our *COI* data set and reconstructed an independent maximum likelihood (ML) tree for each family to test the monophyly of the aforementioned genera. Trees were constructed using the best-suggested models, along with 1000 bootstrap replications and outgroup sequences of the closest available taxa (according to the phylogenetic tree topology in Betancur-R et al.^29^) from our dataset. After checking, 19 genera in 12 families remained non-monophyletic (Supplementary Fig. S3, Table 4).

**Table 4:** List of the 19 non-monophyletic genera in the NJ tree.

Family	Genus/genera	ML tree	References
Apogonidae	*Cheilodipterus*, *Ostorhinchus*	Fig. S3a	^30^
Balistidae	*Balistoides*	Fig. S3b	^31^
Blenniidae	*Salarias*	Fig. S3c	^32,33^
Chaetodontidae	*Chaetodon*	Fig. S3d	^34^
Epinephelidae	*Cephalopholis*, *Epinephelus*	Fig. S3e	^35–37^
Gempylidae	*Rexea*	Fig. S3f	^38^
Haemulidae	*Plectorhinchus*	Fig. S3g	^39^
Labridae	*Coris*, *Halichoeres*, *Oxycheilinus*, *Stethojulis*	Fig. S3h	^40^
Lutjanidae	*Caesio*, *Lutjanus*	Fig. S3g	^29,41,42^
Muraenidae	*Echidna*, *Gymnothorax*	Fig. S3i	^43^
Nomeidae	*Cubiceps*	Fig. S3j	^44^
Pomacentridae	*Chromis*	Fig. S3k	^45^

## Discussions

The majority of species examined in this study exhibit monophyly in the NJ tree (Supplementary Fig. S2), coupled with a barcoding gap of 1.31–3.24% K2P genetic distance, supporting the efficiency and reliability of using *COI* fragments to identify teleost fishes. Increasing genetic distances in higher taxonomic levels (Fig. 3, Table 3) conform with many previous barcoding studies (e.g.^3,4,46–48^). In the species delimitation, 392, 392, 391, and 420 OTUs are identified by BIN, ABGD, ASAP, and bPTP, respectively. The remarkable consistency in OTU numbers across BIN, ABGD, and ASAP implies the robustness of species delimitation through DNA barcoding. Combining various delimitation methods with the morphology of voucher specimens can effectively enhance the accuracy of identification. Nevertheless, 19 genera spanning 12 families do not exhibit monophyly (Supplementary Fig. S3, Table 4), suggesting either insufficient resolution of the used marker or the requirement for additional taxonomic revisions of these taxa. After careful comparison, all topologies of non-monophyletic genera based on *COI* sequences are similar to those in phylogenetic studies conducted with multi-loci or genome-wide data, in which their taxonomies are usually contentious (Table 4 and reference therein). This result supports that *COI* sequences may have certain resolutions at the generic level.

Only two species pairs cannot be delimitated from each other by *COI* sequences, viz., *Abudefduf sexfasciatus* vs. *A. vaigiensis* and *Arothron immaculatus* vs. *A. manilensis*. Both species in pairs possess specific coloration patterns that are easy to recognize and differentiate from one another (Supplementary Fig. S1). Similar results of these two species pairs have been observed in previous studies^49,50^. Incomplete lineage sorting of recent speciation or introgressive hybridization could result in two closely related species sharing haplotypes^51^, a common phenomenon found in marine fishes^52–54^. In contrast, two divergent genetic lineages of *Plotosus lineatus* are observed in Cebu specimens which is similar to the finding of Thu et al.^4^ in Vietnam (Fig. 4). *Plotosus japonicus* is a congener that resembles *P. lineatus* and could be misidentified. The two species can merely be separated by a few meristic characters but both are considered valid^55^. Nevertheless, *COI* sequences of *P. lineatus* + *P. japonicus* from BOLD Systems comprise 10 deeply evolved lineages (BINs) in Indo-West Pacific without a geographical distribution pattern (Fig. 4). Goren et al.^56^ have reviewed nine of the 10 BINs and designated them into four species, including *P. lineatus* (BOLD:AAB3017, BOLD:ACG5707, and BOLD:ACG8821), *P. japonicus* (BOLD:ABZ0283, BOLD:AAB3011, BOLD:AAB3013, and BOLD:ABY8174), *Plotosus* sp. 1 (BOLD:AAB3016), and *Plotosus* sp. 2 (BOLD:AAB3012). Although the two Cebu lineages of *P. lineatus* align with *P. japonicus* and *Plotosus* sp. 2 as defined by Goren et al.^56^, we take a more conservative decision that places both lineages under *P. lineatus*, since the morphology in each lineage has yet to be thoroughly examined for determining the molecular traits of true *P. lineatus* and *P. japonicus*.

Among the 12 Philippine new records, seven species are deep-sea fishes, suggesting that the deep-sea biodiversity in the Philippines may still be incompletely explored and underestimated (Tables 2, 5). Aside from deep-sea species, the other newly recorded species are either small-sized (e.g., Atherinidae and Gobiidae), morphologically similar to their congeners (e.g., Trichiuridae), or reclusive (e.g., Muraenidae). Three species have expanded distribution range, including *Doboatherina duodecimalis* (Atherinidae), *Physiculus chigodarana* (Moridae), and *Parascolopsis rufomaculata* (Nemipteridae). *Doboatherina duodecimalis* was widely distributed in Indo-West Pacific, ranging from Madagascar to Vanuatu, and north to the Gulf of Thailand and central Philippines. However, Sasaki & Kimura^57^ resurrected *D. balabacensis*, a Philippine endemic species previously considered a junior synonym of *D. duodecimalis,* and recognized all Philippine *D. duodecimalis* records as *D. balabacensis*. The authors also identified two genetic lineages (Madagascar & Andaman Sea vs. Ambon & Sulawesi) of *D. duodecimalis*, with no morphological differentiation observed among specimens. In the present study, a *COI* sequence from Cebu belongs to the Ambon & Sulawesi lineage of *D. duodecimalis* according to a GenBank sequence (AB849035) identified by Sasaki & Kimura^57^, representing a range expansion as well as a sympatric distribution with *D. balabacensis* in the Philippine waters. The identification of *Physiculus chigodarana* in this study is based on two diagnostic morphological characters (the anteriorly placed light organ and the extended first dorsal fin) that are unique in the genus^58^. There is no available *COI* sequence of *P. chigodarana* in databases; however, the BIN that matches this specimen comprises two monophyletic clades of *COI* sequences with a 1.8% inter-clade K2P distance, including (1) five sequences of *P. natalensis* from South Africa; and (2) five sequences of four species from various areas (our *P. chigodarana* from Cebu, two *Physiculus* sp. and one *P. roseus* from Western Australia, and one *P. japonicus* from Taiwan). This result suggests that the four sequences from Western Australia and Taiwan might have been misidentified and could potentially correspond to *P. chigodarana*. The record represents the range expansion of *P. chigodarana* from Japan and Taiwan to the central Philippines. *Parascolopsis rufomaculata*, on the other hand, is a rare deep-sea nemipterid that has only been documented in northwestern Australia and southern Java^59^. The Cebu record of *P. rufomaculata* expands its distribution range across the equatorial to the northern hemisphere, suggesting that this little-known nemipterid might have a broader distribution in northern Australia and the East Indies regions.

**Table 5:** List of the 23 deep-sea species recorded in this study inhabiting a depth range greater than 200 m.

Family	Species	Depth (meters)	Collection locality
Acropomatidae	*Doederleinia berycoides*	100–600	Bogo
Antigoniidae	*Antigonia rubescens*	50–750	Bogo
Ariommatidae	*Ariomma brevimanus*	–350	Badian
Bembropidae	*Bembrops caudimacula*	186–500	Bogo
Bramidae	*Brama pauciradiata*	80–550	n/a
Chlorophthalmidae	*Chlorophthalmus* sp.	n/a	Badian
Dactylopteridae	*Dactyloptena tiltoni*	119–565	Badian
Gempylidae	*Lepidocybium flavobrunneum*	200–1100	Badian
	*Rexea prometheoides*	135–540	Badian
Malakichthyidae	*Verilus pacificus*	60–500	Badian
Moridae	*Physiculus chigodarana*	300–500	Bogo
Myctophidae	*Diaphus watasei*	100–2005	Badian
Nemipteridae	*Parascolopsis akatamae*	100–200	n/a
	*Parascolopsis eriomma*	150–200	Santander
	*Parascolopsis rufomaculata*	210–320	Bogo
Nomeidae	*Cubiceps pauciradiatus*	58–1000	Badian
	*Psenes cyanophrys*	20–550	Badian
Ophidiidae	*Neobythites bimaculatus*	435–500	Bogo
Serranidae	*Chelidoperca* sp.	n/a	Badian
Tetraodontidae	*Lagocephalus lagocephalus*	10–476	Santander
Triglidae	*Satyrichthys laticeps*	58–300	Bogo
	*Satyrichthys rieffeli*	65–600	Badian
	*Satyrichthys welchi*	80–228	Bogo

The species composition of collected specimens in this study may reflect the environmental condition of fishery grounds, fishing gears and methods used by local fishers, or the diet habits of the local people since most materials are obtained from local markets and fish landing sites. In the species list, the majority are coral reef or reef-related shallow-water species, suggesting that coastal communities in Cebu have heavily relied on the coral reef resource^60,61^ (Fig. 2). Furthermore, 23 deep-sea species in 17 families that can inhabit a depth range greater than 200 m are discovered (Table 5). All the deep-sea species are collected from either the southwestern (Badian and Santander) or northeastern (Bogo) coasts of the province connecting to the Tañon Strait and Camotes Sea fishery ecosystems, respectively, the two deepest marine areas in Central Visayas^20^.

A notable difference is observed in species composition compared to another market fish barcoding survey in Vietnam^4^. The most speciose 10 families in Thu et al.^4^ are the Gobiidae (8.2%), Carangidae (6.1%), Lutjanidae (3.8%), Nemipteridae (3.6%), Epinephelidae (3.3%), Apogonidae (2.9%), Callionymidae (2.1%), Cynoglossidae (2.1%), Leiognathidae (2.1%), and Platycephalidae (2.1%). Most of these species are associated with sandy or muddy substrates, particularly the latter four families, which are relatively uncommon in Cebu. The discrepancy can largely be attributed to variations in habitats, as two of the three sampling areas in Thu et al.^4^, the Halong Bay and Gulf of Thailand, are characterized by soft-bottomed terrains^62,63^. In contrast, our Cebu checklist and that of Bemis et al.^5^ share the identical composition of the top 11 speciose families, differing only slightly in their rankings. Nevertheless, this study identified 120 species that were not included in Bemis et al.^5^, in which the top five speciose families are Pomacentridae (nine species), Labridae (eight species), Muraenidae (eight species), Gobiidae (seven species), and Apogonidae (six species) (Fig. 2). Moreover, six families (Atherionidae, Bembropidae, Cynoglossidae, Myctophidae, Sillaginidae, and Zenarchopteridae) were absent from Bemis et al.^5^, demonstrating the significant contribution of this study in expanding the barcode reference library for Philippine marine fishes. Likewise, the Cebu market survey and a recent fish checklist in Palawan, based on underwater visual census (UVC) in coral reefs^64^, show a very similar composition of speciose families. The 10 most prevalent families in the latter (Pomacentridae, Labridae, Lutjanidae, Chaetodontidae, Epinephelidae, Carangidae, Acanthuridae, Gobiidae, Scaridae, and Apogonidae) closely align with those in this study, with the exception of Muraenidae and Nemipteridae, which hold higher proportions in our findings. Balisco et al.^64^ reported a higher number of nemipterids (24 vs. 16 in this study), while the count of muraenid species is lower (11 vs. 19). The predictable underestimation of reclusive taxa like moray eels is a recognized limitation of using UVC to assess fish diversity^64^. On the other hand, moray eels are an important component of popular local dishes and have been regarded as one of the main targets of commercial fisheries in the Central Visayas region^65^, resulting in more species obtainable from fish markets.

In the present study, 12 (3%) of species documented in Cebu are new records in the Philippines, indicating a rich reserve of biodiversity yet to be fully explored. While no species classified as critically endangered or endangered are identified, it is important to highlight that nearly a third of the species remain unevaluated or lack adequate information (IUCN catalogs NE and DD), hindering comprehensive assessments. The Central Visayas, formerly renowned for hosting one of the world’s most prolific coral reef fish populations, now has faced a contrasting reality^23,24^. Recent investigations revealed that both this area and the southern Philippine Seas exhibit the lowest species richness. This significant decline in biomass and diversity has largely been attributed to prolonged overexploitation and habitat degradation^23,24,66^. Apparently, there have been no published references comprehensively documenting the marine fishes of Cebu waters prior to this study. With the reported decline in species diversity and biomass in the area, some species could have become locally extinct before being properly documented. Therefore, the establishment of a reliable morphology-based DNA barcoding library for commonly caught fishes in Cebu is an urgent necessity. This resource can assist researchers and fishery authorities in accurately identifying fish species present in the province, as well as in detecting threatened and cryptic species. It can serve as a foundation for fishery managers to implement biodiversity conservation and formulate suitable management policies, particularly for species that are already at risk.

## Material and methods

### Sample collection

Two fish collection activities were conducted in February 2010 and December 2018–January 2019, spanning a total of 21 days. Marine fish specimens were collected from 18 fish markets and landing sites across Cebu province, including Mactan and Bantayan islands (Fig. 1, Supplementary Table S1). Each fish was photographed, and either their fins or muscle tissue were clipped, stored in 95% ethanol, and frozen at − 20 °C before DNA extraction. Small specimens were taken as voucher specimens, while only the fin clips or tissue samples were preserved for large and/or expensive specimens (Supplementary Table S2). Voucher specimens were fixed using 10% formalin and gradually transferred to 70% ethanol for further preservation. All fin clip or tissue samples and voucher specimens were deposited in the ichthyological collection of the Department of Oceanography, National Sun Yat-sen University, Kaohsiung (DOS), Marine Ecology and Conservation Research Center, National Academy of Marine Research, Kaohsiung (NAMR), and National Museum of the Philippines, Manila (PNM). Necessary permits were obtained from the Bureau of Fisheries and Aquatic Resources Region 7 before the specimen transport. An initial checklist was created by assessing the morphology of the species using photographs and voucher specimens.

### DNA sequencing

At least one specimen per species (according to the initial morphology-based checklist) was chosen for DNA extraction and molecular analysis to confirm the identification. A GeneMark DNA Purification Kit (GMbiolab, Taichung, Taiwan) was used to extract DNA from tissue samples. Partial fragments of the mitochondrial *COI* gene were amplified by polymerase chain reaction (PCR) using different combinations of fish-specific primers designed by Ward et al.^3^: FishF1 (5′-TCA ACC AAC CAC AAA GAC ATT GGC AC-3′), FishF2 (5′-TCG ACT AAT CAT AAA GAT ATC GGC AC-3′), FishR1 (5′-TAG ACT TCT GGG TGG CCA AAG AAT CA-3′), and FishR2 (5′-ACT TCA GGG TGA CCG AAG AAT CAG AA-3′). The PCR material, thermal cycling profile of PCR, quality check and purification of the PCR products, and DNA sequencing followed Thu et al.^4^. Sequences were trimmed and edited manually in MEGA version 11^67^. The edited sequences were then translated into amino acid sequences to identify the possible occurrence of insertion-deletion mutations (indels) or stop codons, which may represent a sign of nuclear mitochondrial pseudogenes^68^. All *COI* sequences have been submitted to GenBank, and their accession numbers are provided in Supplementary Table S2.

### Data analyses

Obtained *COI* sequences were blasted in GenBank and BOLD Systems online public databases to create another species list. Species identification was accepted only when the similarity value exceeded 98% with the nearest DNA barcode in the database^69^. The barcode index number (BIN) of each sequence was also recorded. Each BIN corresponds to an operational taxonomical unit (OTU) designated via a sequence-based clustering method from BOLD Systems^70^, which is helpful for filtering possible misidentifications of sequences or revealing cryptic species. In cases where a specimen displayed conflicting morphological and molecular identifications, or online databases proposed two or more species for a specimen with high similarities, we further examined the photo and voucher specimen, if any, using taxonomic keys and published literature to confirm their identification (e.g.^58,71^). We followed the taxonomic classification by Betancur-R et al.^29^ for the order level, and by Eschmeyer's Catalog of Fishes^72^ for the family, genus, and species levels. The final species list was cross-referenced with records from FishBase^73^, Eschmeyer's Catalog of Fishes^72^, Balisco et al.^64^, and Bemis et al*.*^5^ to ascertain the number of newly recorded species in the Philippines. Additionally, we determined the conservation status of each species by referencing the International Union for Conservation of Nature Red List of Threatened Species^74^.

After species identification, all sequences were aligned and trimmed to the same length using MEGA version 11 for further analyses. The Kimura-2-Parameter (K2P)^75^ genetic distances at different taxonomic levels were calculated, including intra-specific distance (excluding species with only one sequence), inter-specific distance within the same genus (excluding genera with only one species), inter-genus distance within the same family (excluding families with only one genus), inter-family distance within the same order (excluding orders with only one family), and inter-order distance within the infraclass Teleostei. All *COI* sequences were used to construct a neighbor-joining (NJ) tree using Tamura-Nei + *Γ* model with 1000 bootstrap replications^76–78^. Both the model testing and tree construction were conducted using MEGA version 11.

Lastly, we used the barcoding data set from Cebu to test the species delimitation using three clustering models, including the Automatic Barcode Gap Discovery (ABGD^79^), the Assemble Species by Automatic Partitioning (ASAP^80^), and the Bayesian implementation of the Poisson Tree Processes (bPTP^81^). These results were compared with the final species list (based on morphology and blast results) and the number of BINs.

### Supplementary Information

Supplementary Figure S1.

Supplementary Figures.

Supplementary Tables.

## Data Availability

Catalog numbers and GenBank accession numbers for all sequenced specimens are provided in Supplementary Table S2, and their corresponding photos can be found in Supplementary Fig. S1. All sequences are available on GenBank (https://www.ncbi.nlm.nih.gov/genbank/) with accession numbers OM037461, OM037474, OM037539, ON351491, OQ508847, and OR113751–OR114283.
