# Supplementary material for: DNA barcoding of marine teleost fishes (Teleostei) in Cebu, the Philippines, a biodiversity hotspot of the coral triangle
Source: Sci Rep. 2023 Sep 8;13:14867. doi: 10.1038/s41598-023-41832-9 (PMC10491795; doi:10.1038/s41598-023-41832-9)
Supplement: Supplementary file 1 — Supplementary Figure S1. [file 41598_2023_41832_MOESM1_ESM.pdf]

DNA barcoding of marine teleost fishes (Teleostei) in Cebu, the Philippines, a biodiversity hotspot of the coral triangle

Wen-Chien Huang[1,2,**], Florence Chan Evacitas[3,**], Rodulf Anthony Balisco[4,5], Cleto L. Nañola Jr.[6], Tak-Kei Chou[4], Wei-Cheng Jhuang[1,2], Chih-Wei Chang[7], Kang-Ning Shen[7], Kwang-Tsao Shao[4,8], Te-Yu Liao[4,*]

[1]Doctoral Degree Program in Marine Biotechnology, National Sun Yat-sen University, Kaohsiung, Taiwan
[2]Doctoral Degree Program in Marine Biotechnology, Academia Sinica, Taipei, Taiwan
[3]Department of Biology and Environmental Science, University of the Philippines Cebu, Cebu City, Philippines
[4]Department of Oceanography, National Sun Yat-sen University, Kaohsiung, Taiwan
[5]College of Fisheries and Aquatic Sciences, Western Philippines University, Puerto Princesa City, Palawan, Philippines
[6]Department of Biological Sciences and Environmental Studies, University of the Philippines Mindanao, Davao City, Philippines
[7]Marine Ecology and Conservation Research Center, National Academy of Marine Research, Kaohsiung, Taiwan
[8]Biodiversity Research Center, Academia Sinica, Taipei, Taiwan

**Corresponding author*: swp0117@gmail.com
**Authors contributed equally

Supplementary Figure S1. Photographs of sequenced voucher specimens.

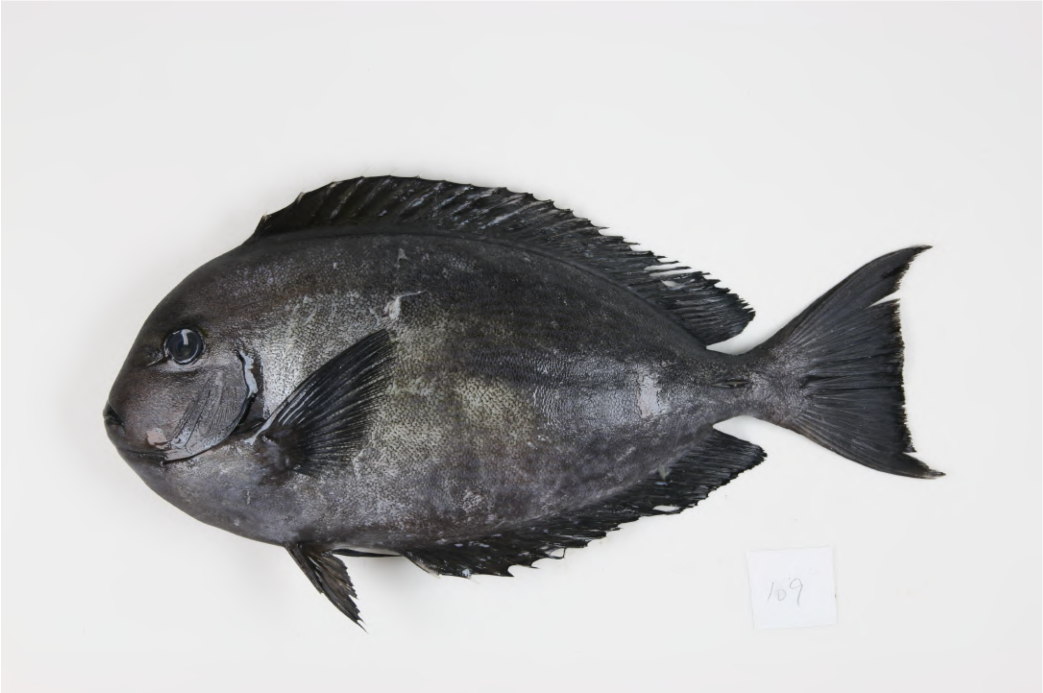

DOS 08612, *Acanthurus mata*, OR114176.

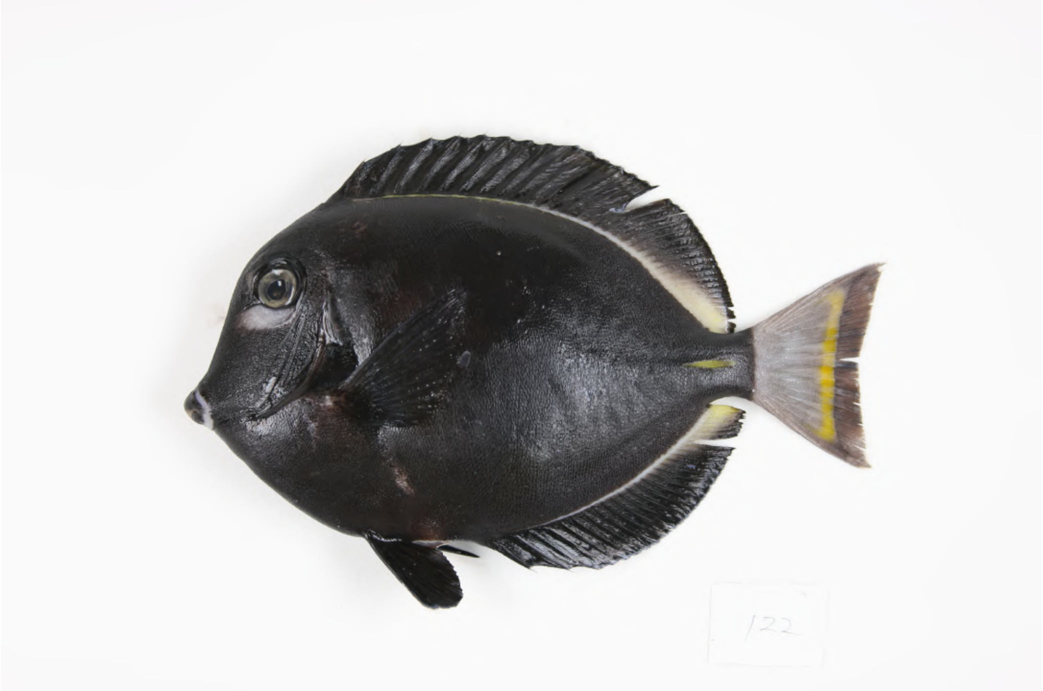

DOS 08613, *Acanthurus nigricans*, OR114177.

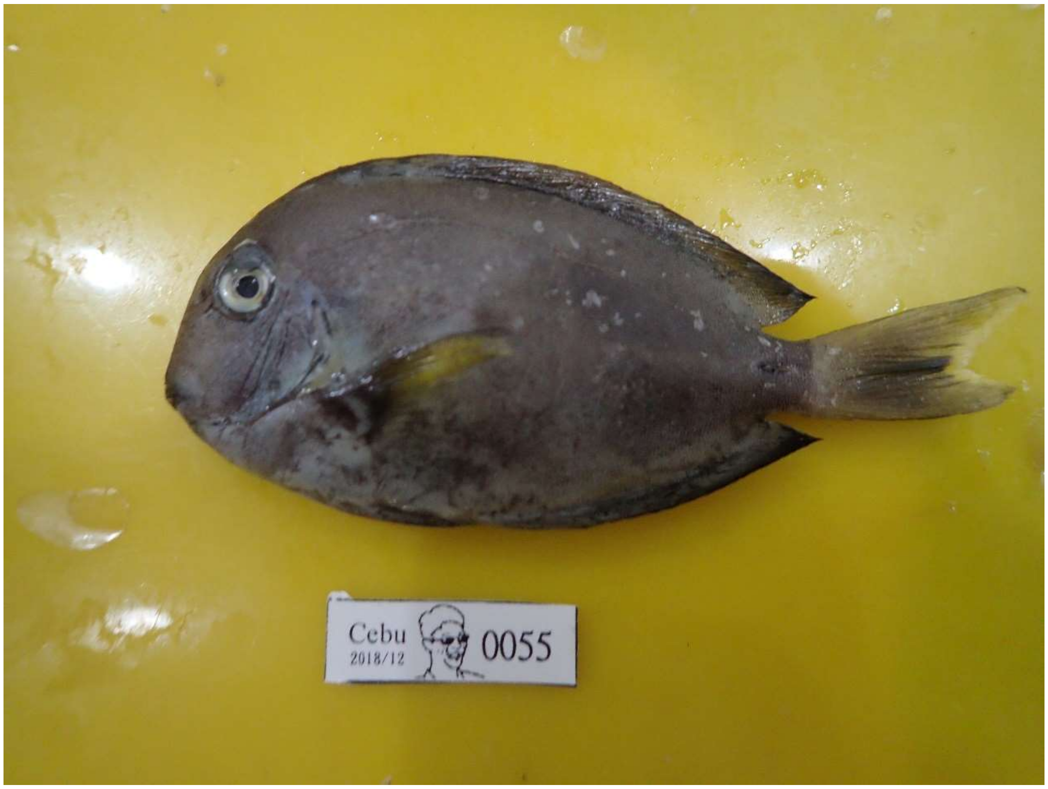

DOS 06559, *Acanthurus nigricauda*, OR113763. (specimen not preserved)

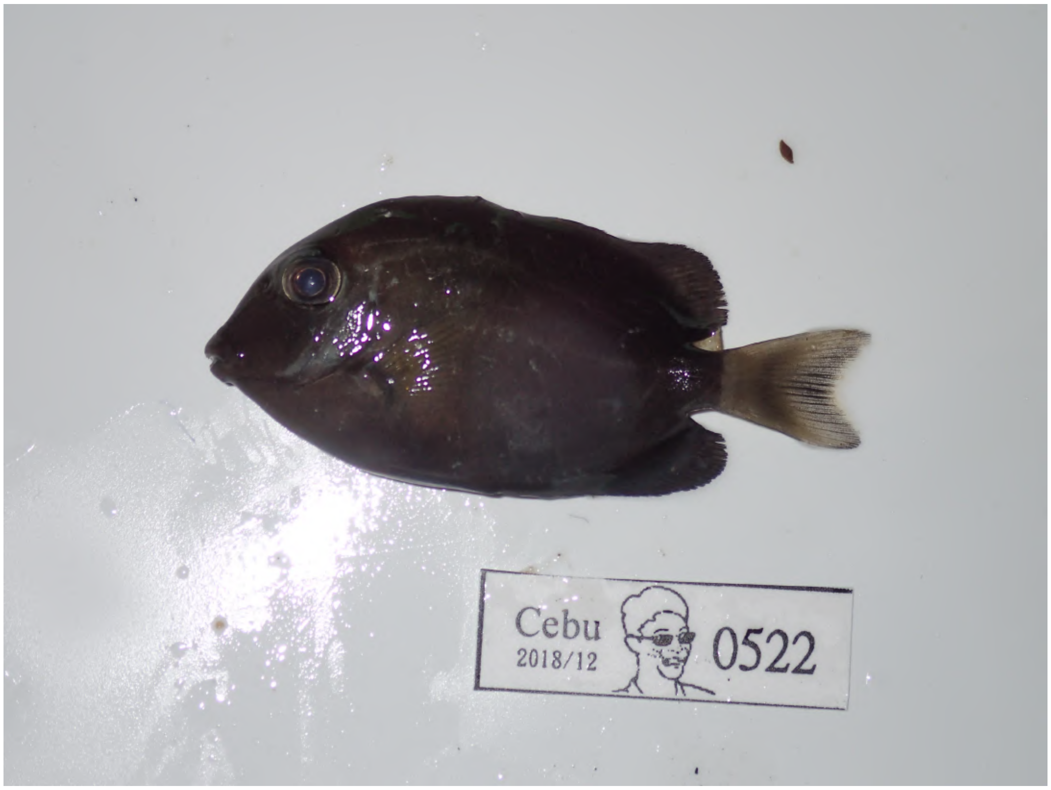

DOS 06562-1, *Acanthurus nigricauda*, OR113765.

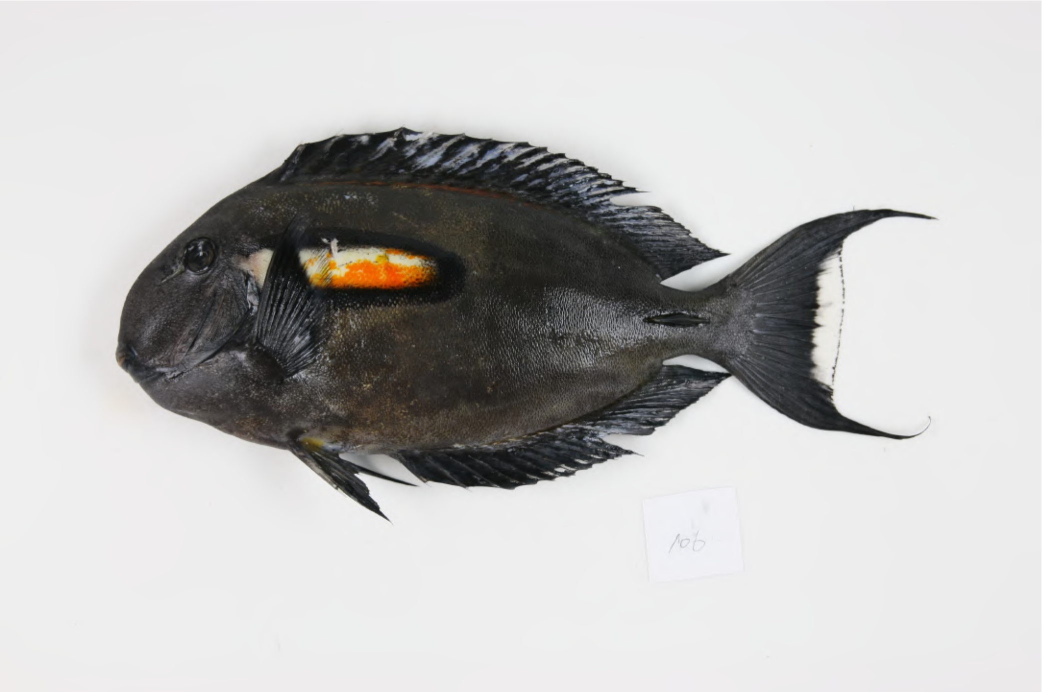

DOS 08614, *Acanthurus olivaceus*, OR114178.

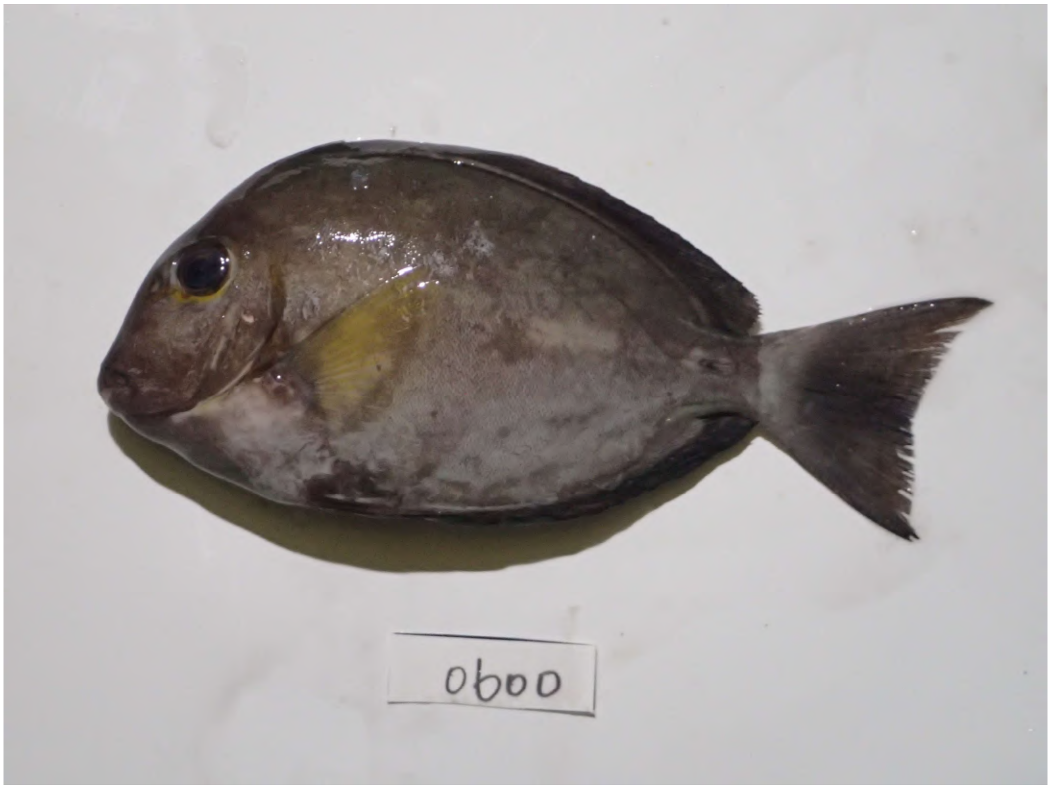

DOS 06558, *Acanthurus xanthopterus*, OR113762.

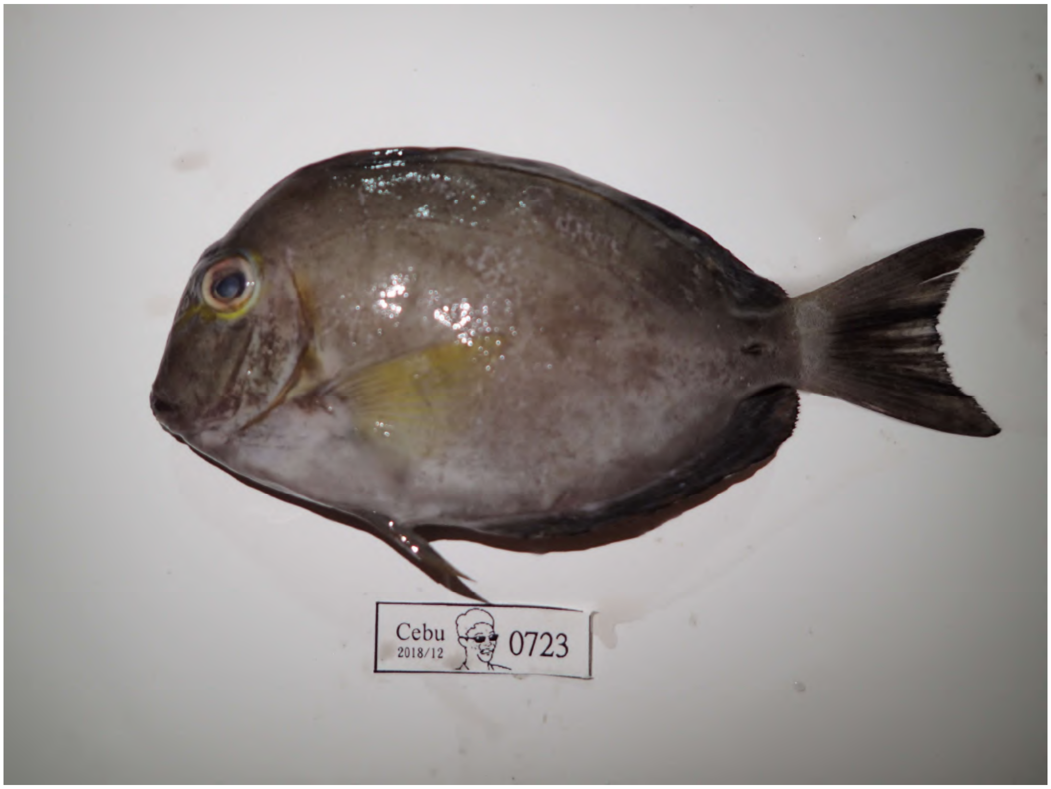

DOS 06563-1, *Acanthurus xanthopterus*, OR113766.

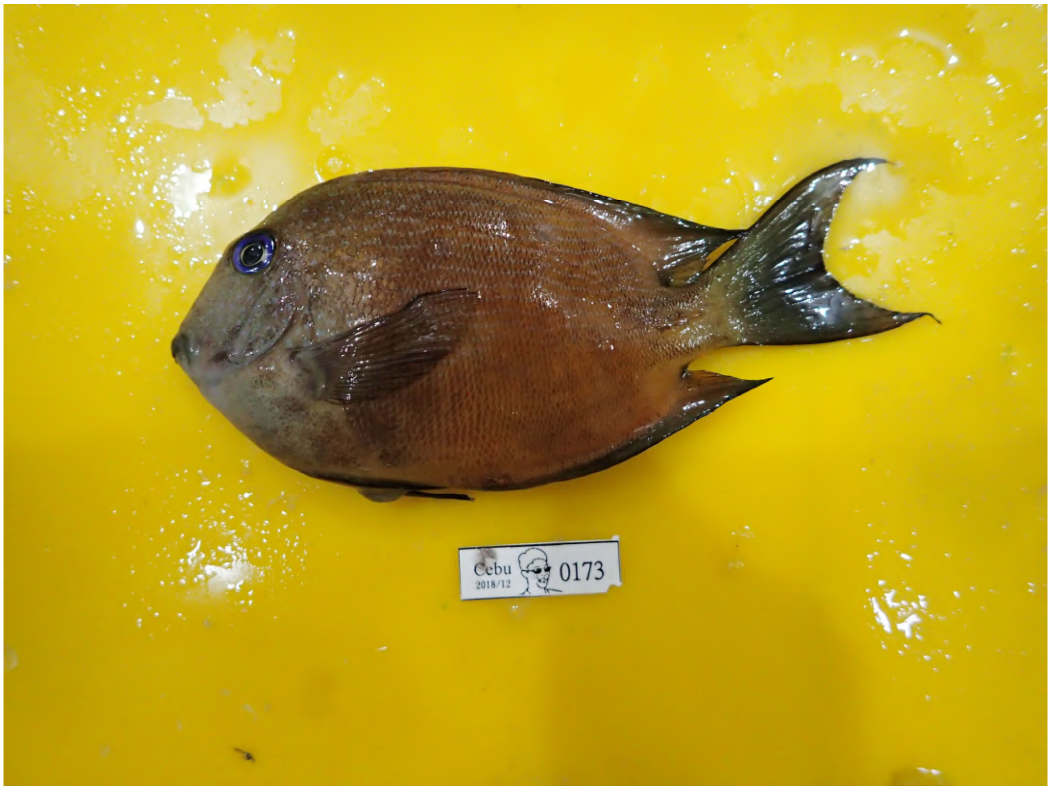

DOS 06564, *Ctenochaetus binotatus*, OR113767. (specimen not preserved)

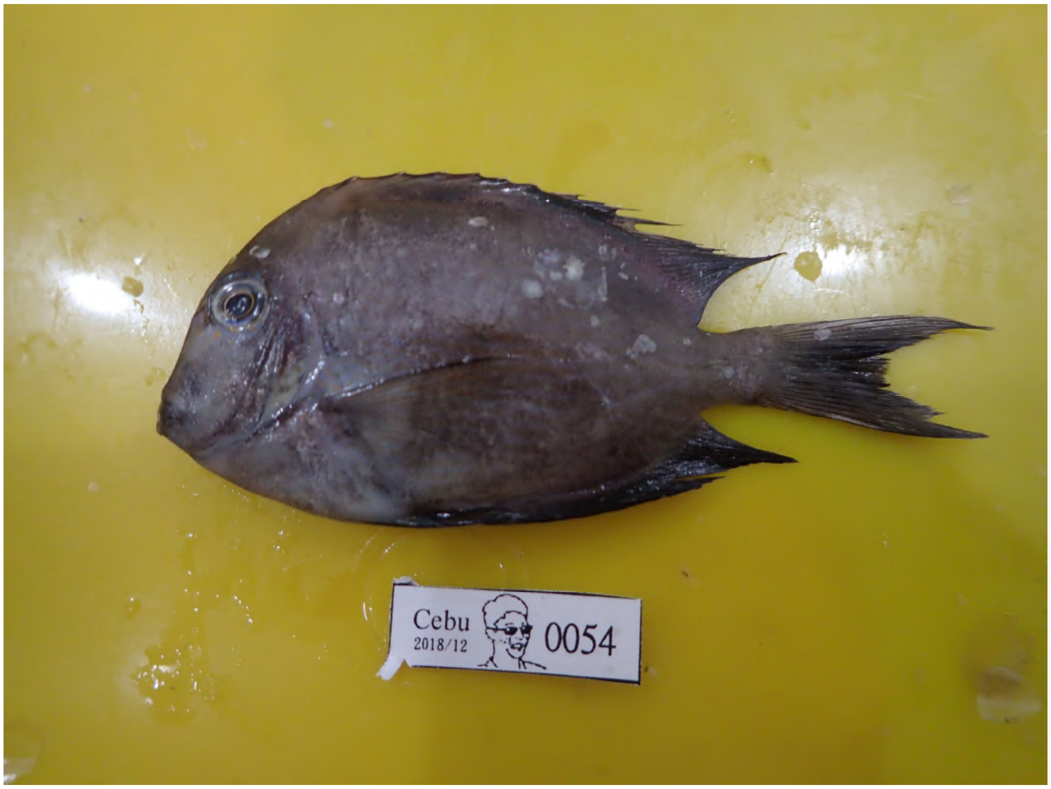

DOS 06557, *Ctenochaetus striatus*, OR113761.

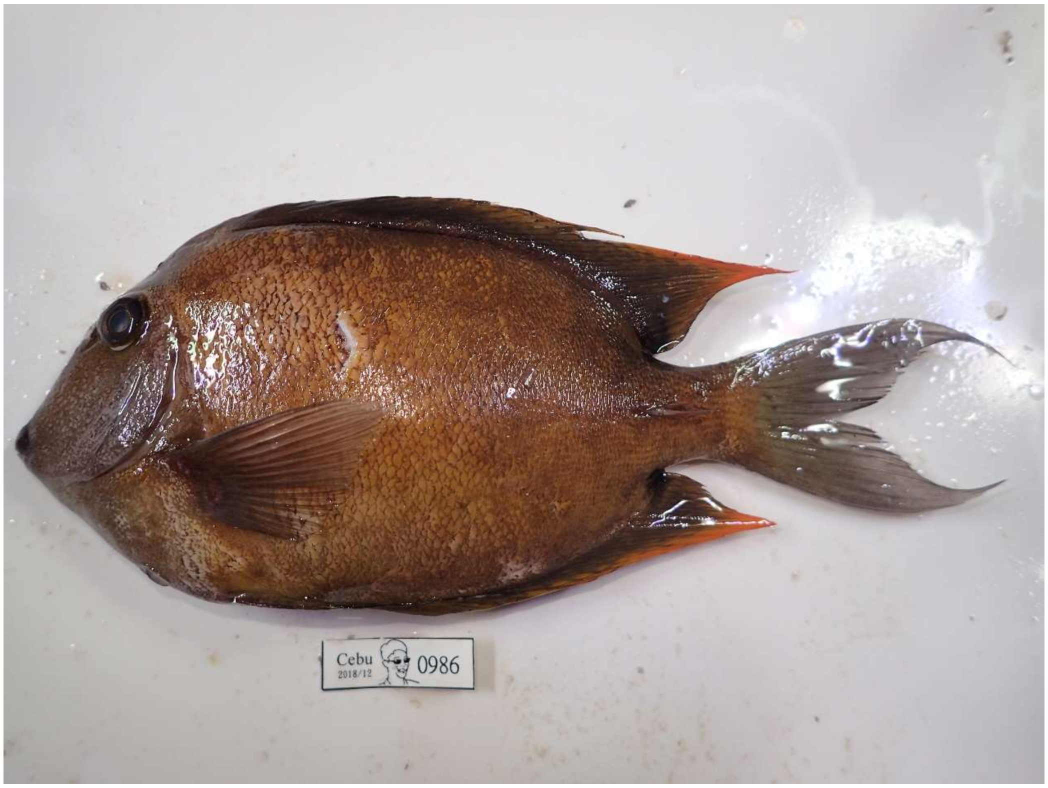

DOS 06561, *Ctenochaetus tominiensis*, OR113764.

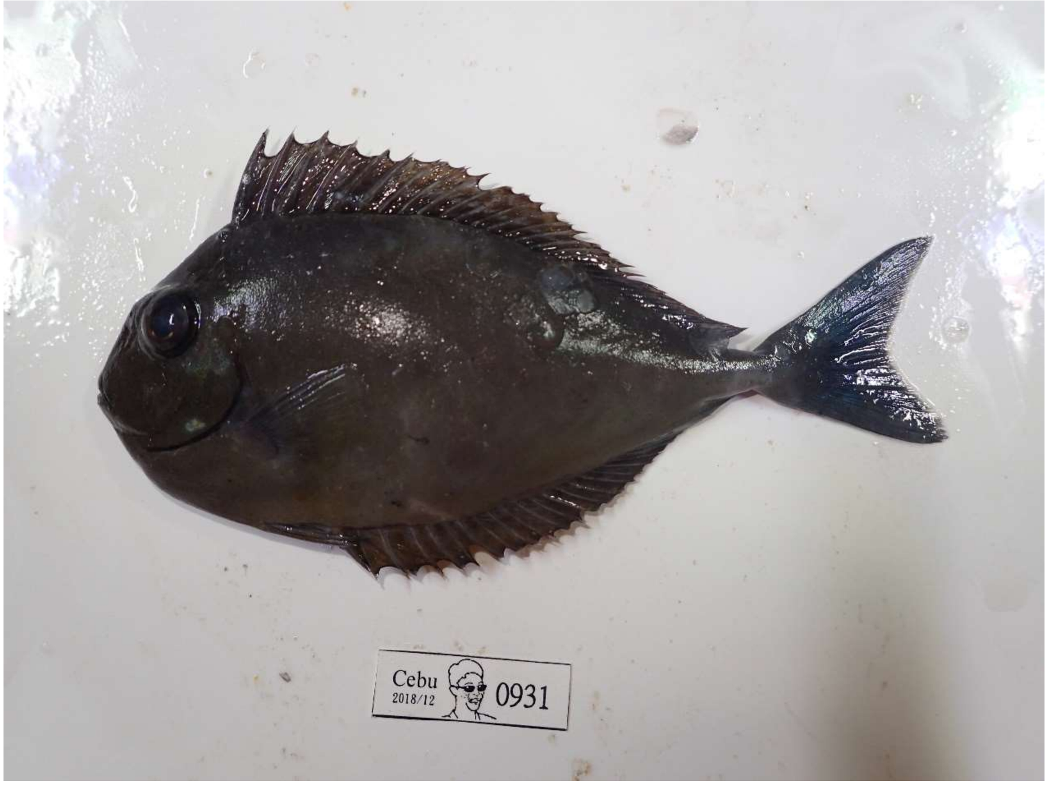

DOS 06571, *Naso hexacanthus*, OR113774. (specimen not preserved)

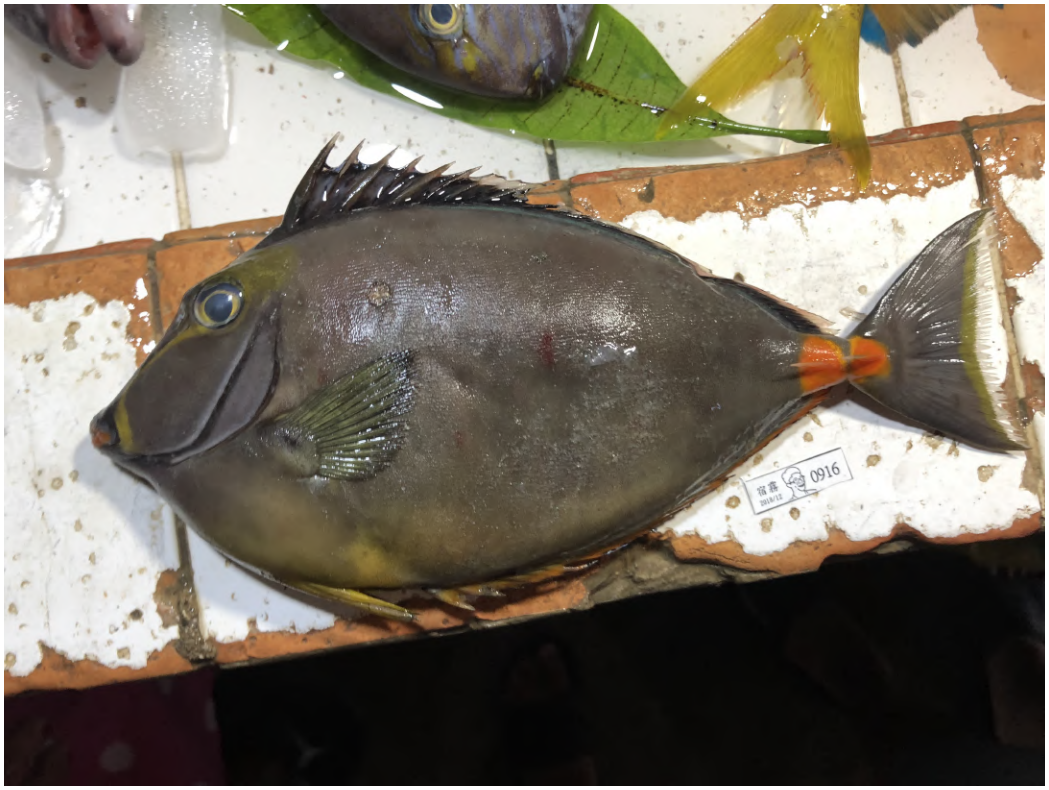

DOS 06567-3, *Naso lituratus*, OR113770. (specimen not preserved)

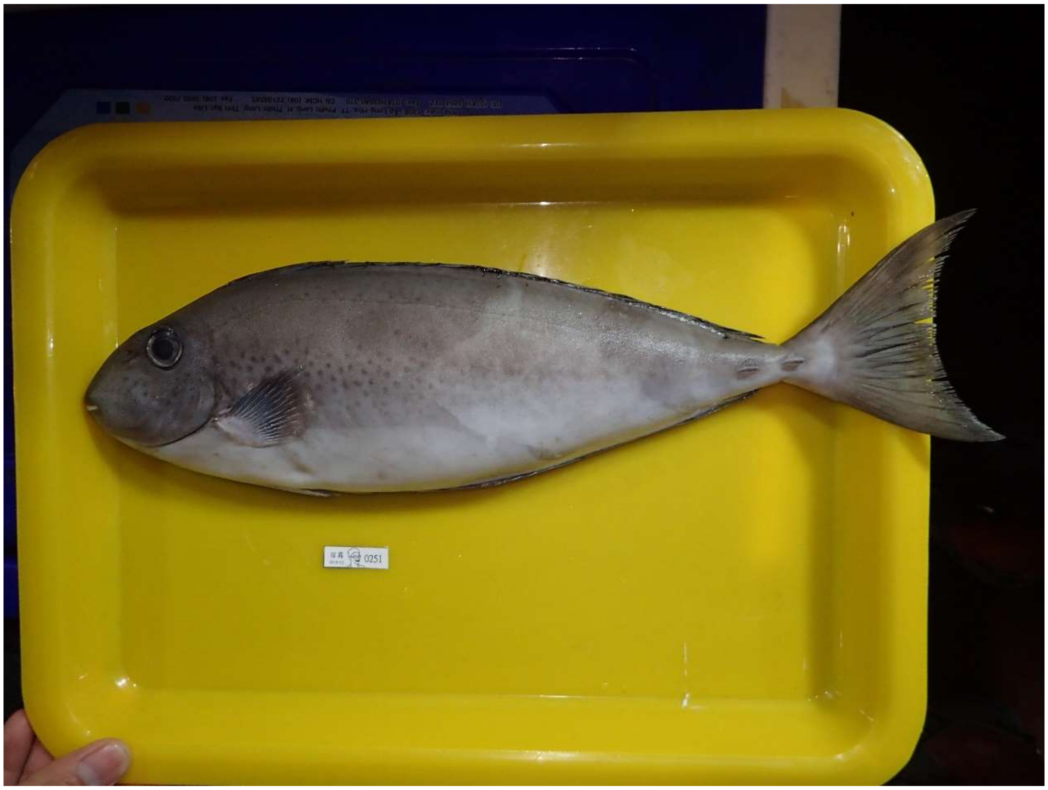

DOS 06568-1, *Naso lopezi*, OR113771. (specimen not preserved)

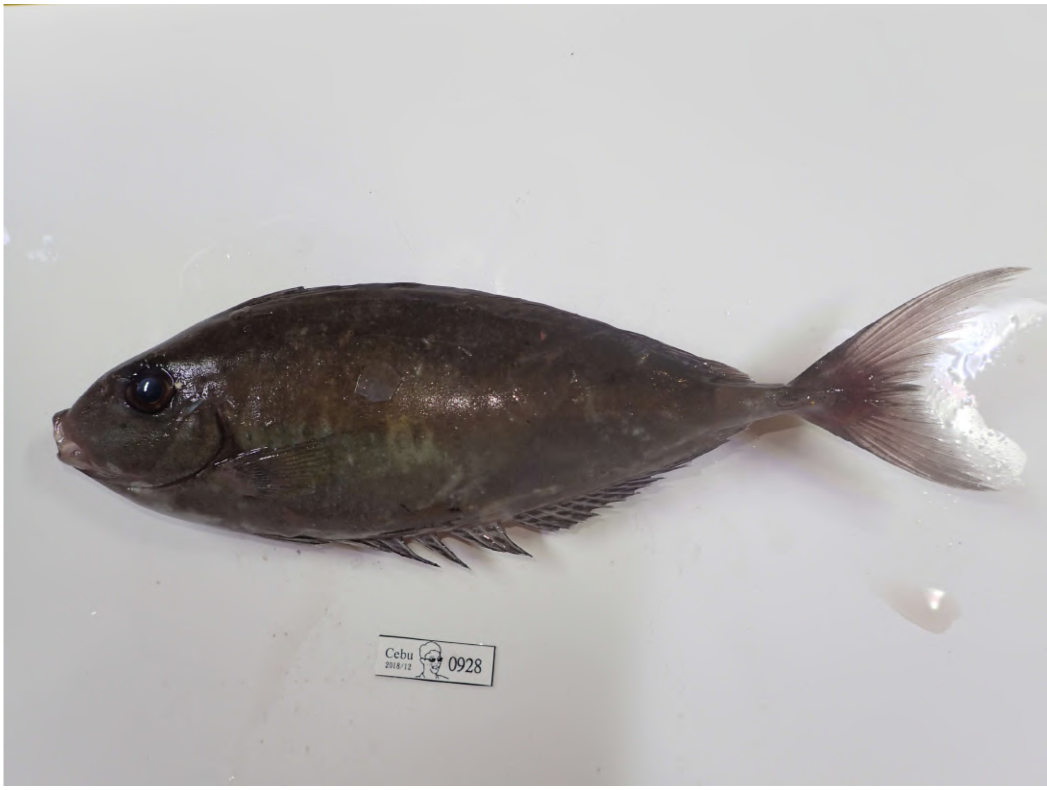

DOS 06569-2, *Naso thynnoides*, OR113772. (specimen not preserved)

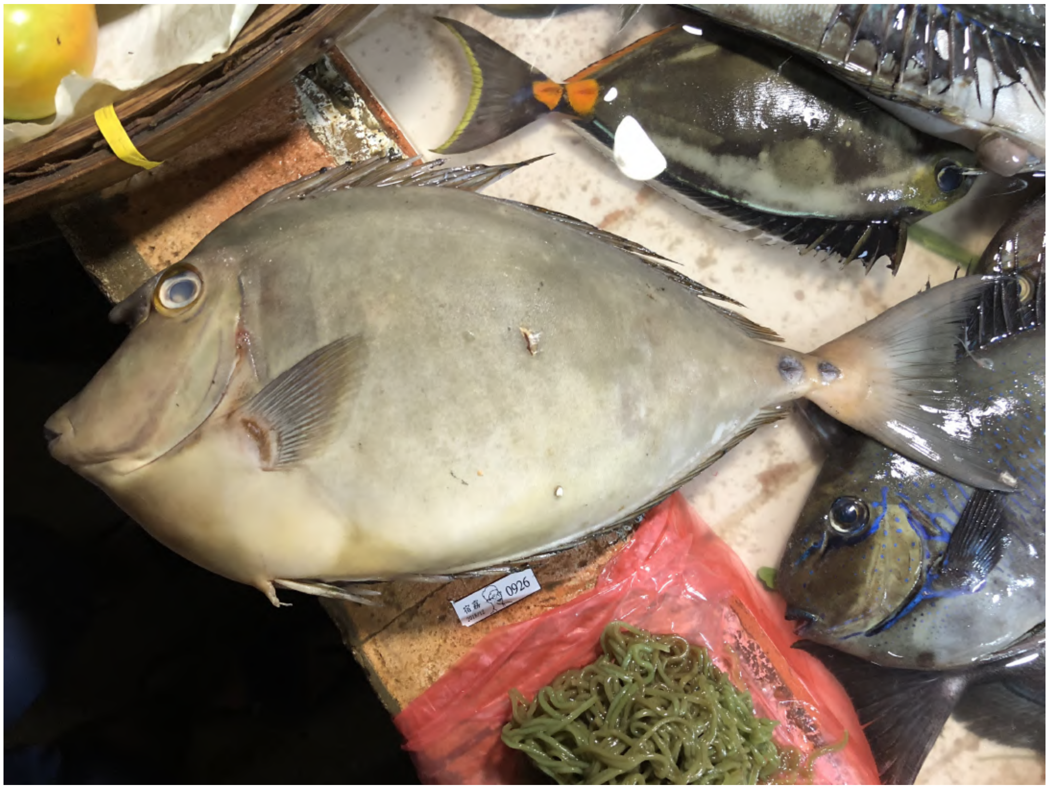

DOS 06572, *Naso unicornis*, OR113775. (specimen not preserved)

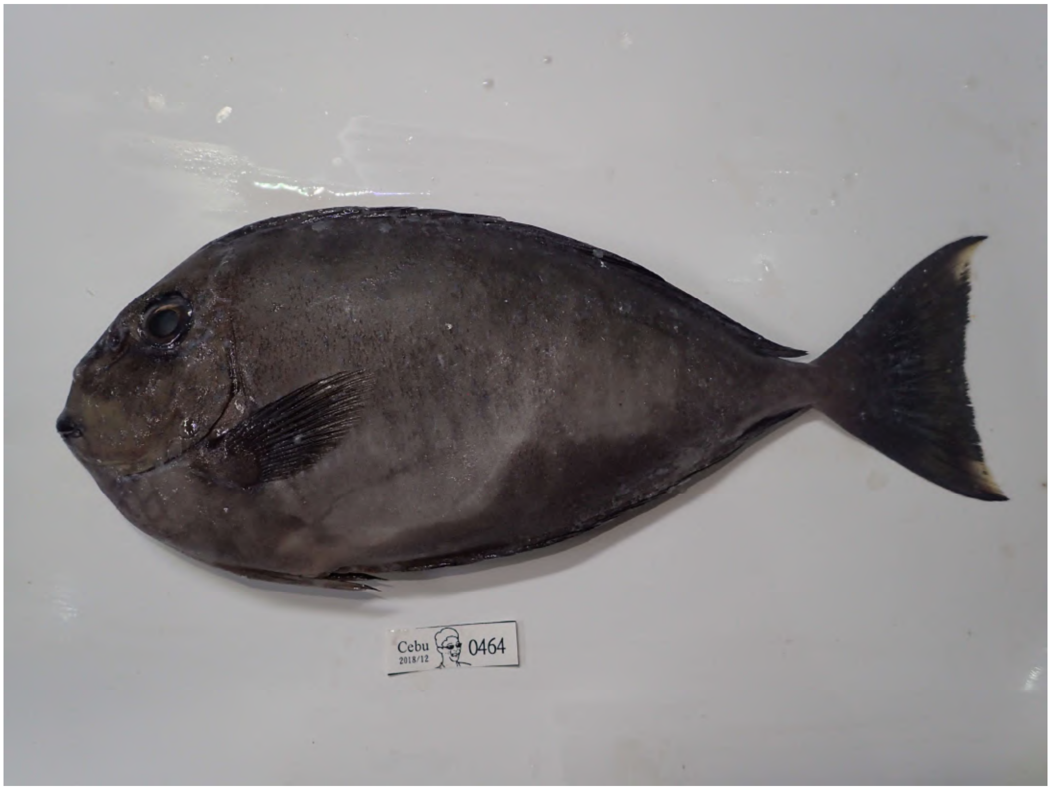

DOS 06565, *Naso vlamingii*, OR113768.

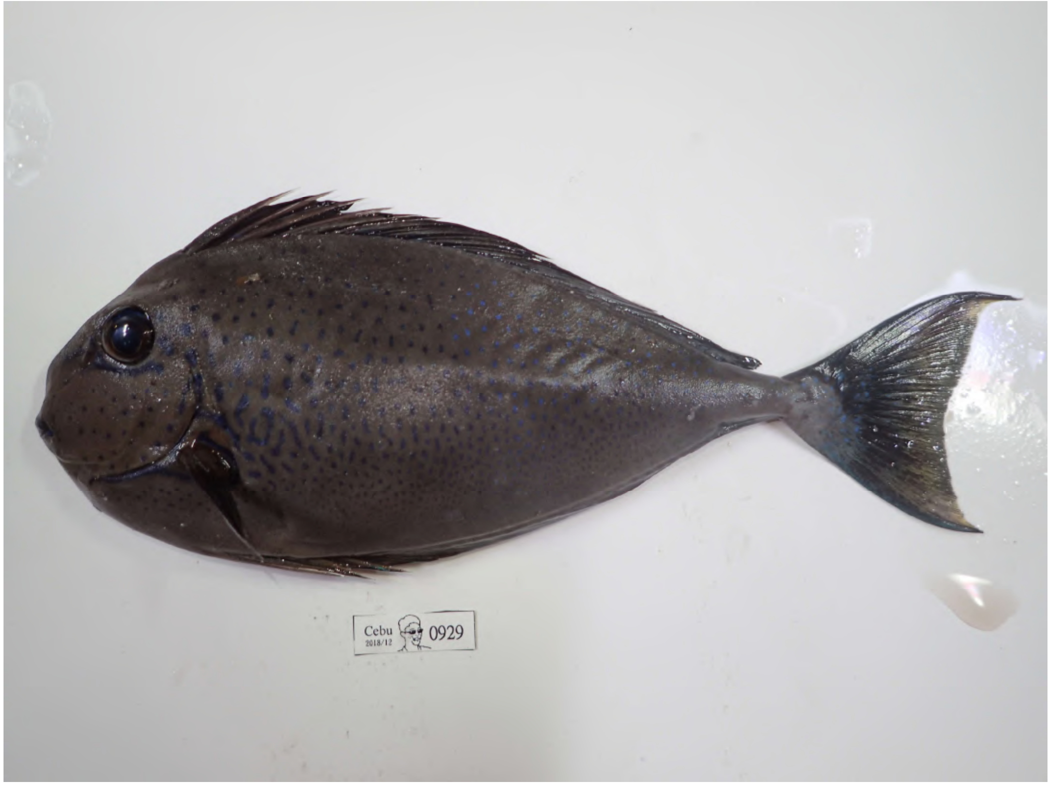

DOS 06570-1, *Naso vlamingii*, OR113773. (specimen not preserved)

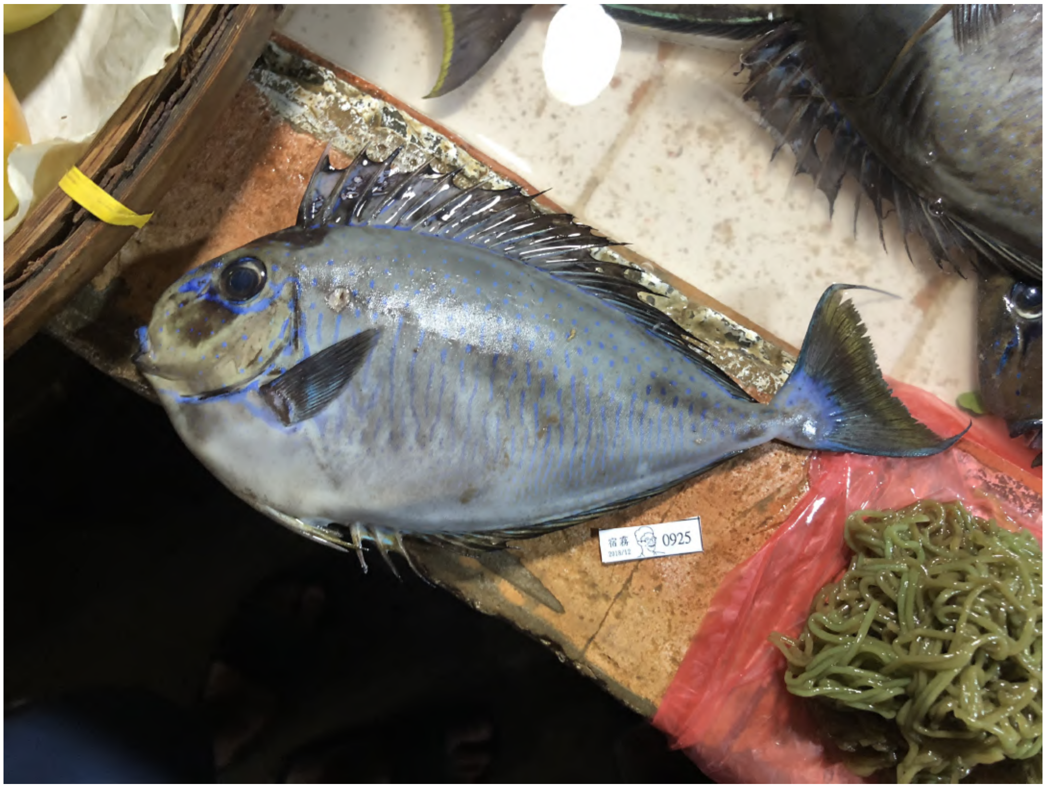

DOS 06573, *Naso vlamingii*, OR113776. (specimen not preserved)

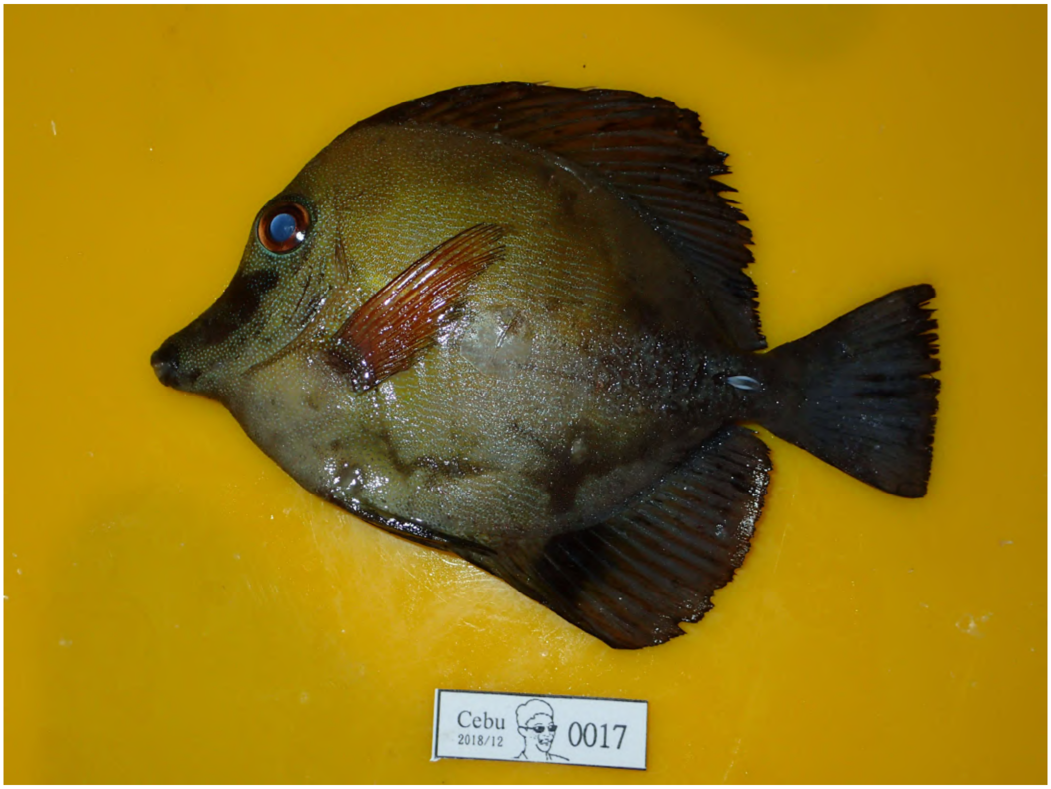

DOS 06574-1, *Zebrasoma scopas*, OR113777.

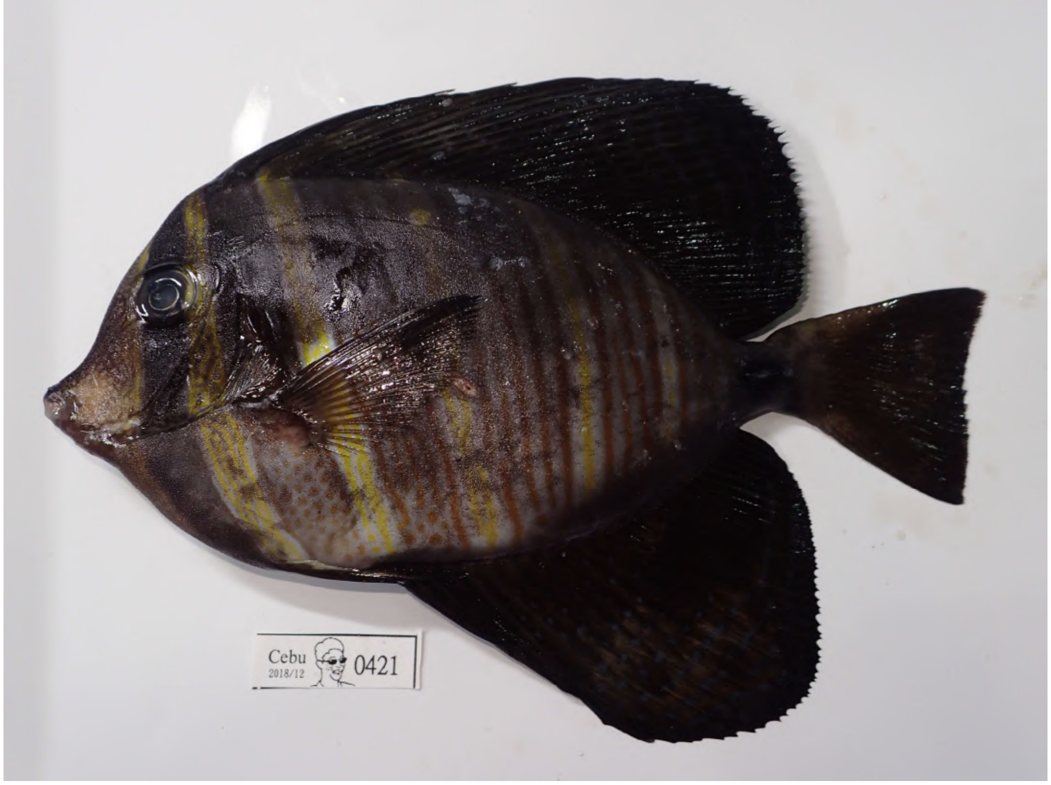

DOS 06575-1, *Zebrasoma velifer*, OR113778.

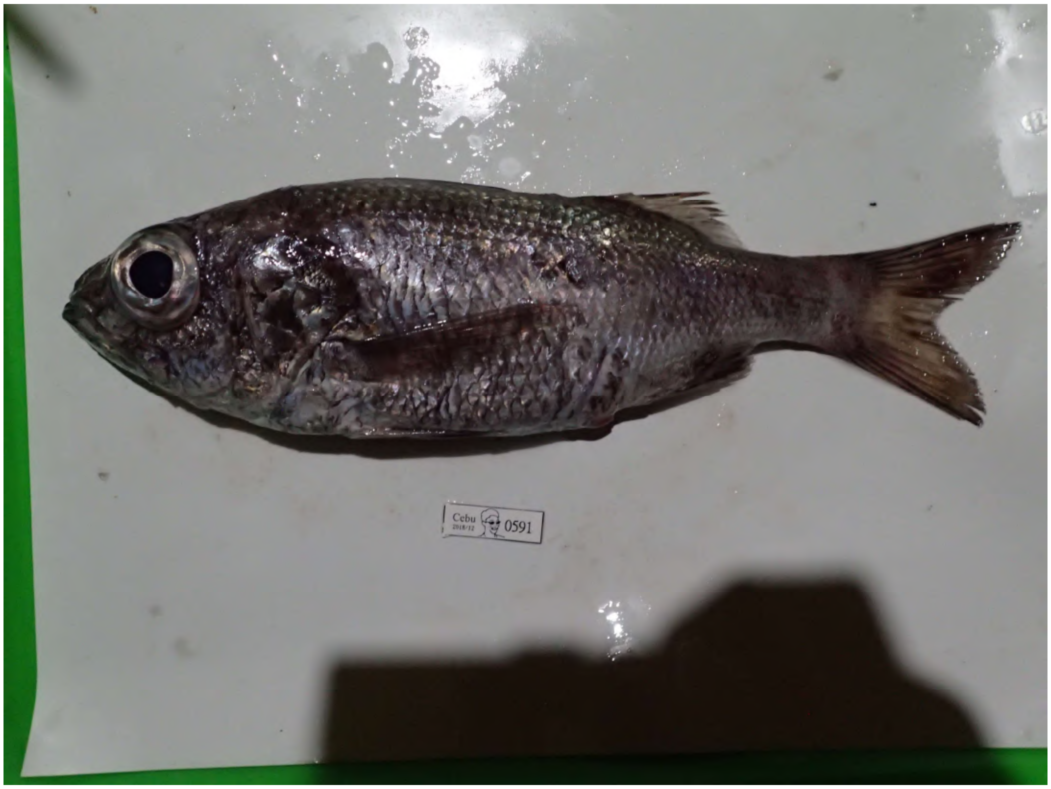

DOS 06996-1, *Verilus pacificus*, OR114164.

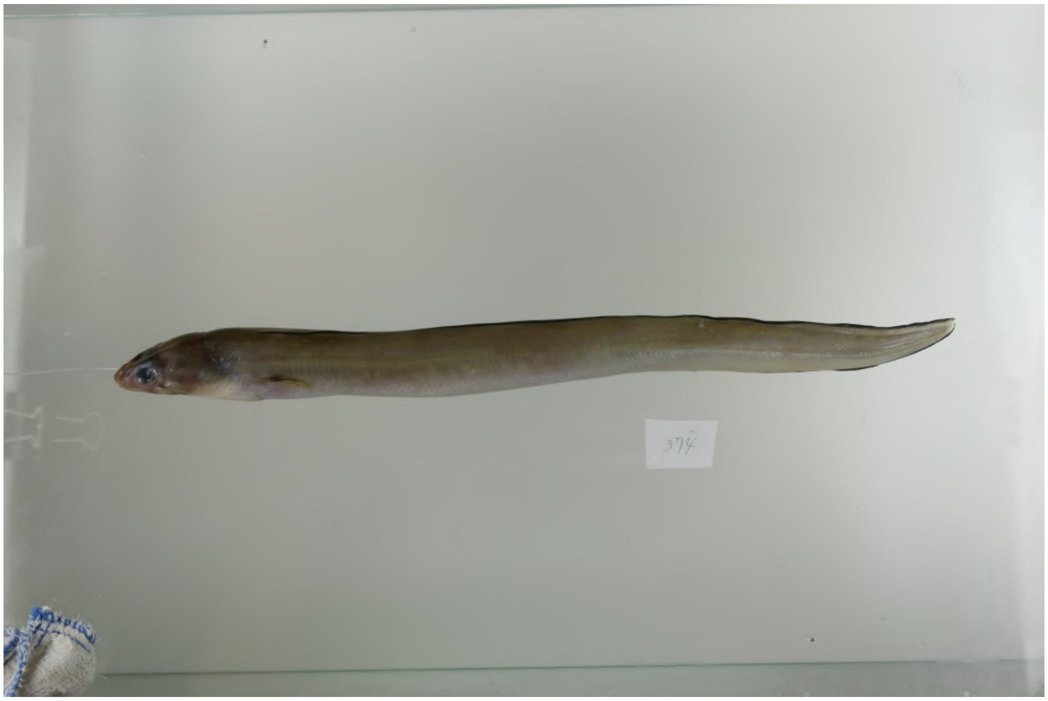

DOS 08615, *Ariosoma meeki*, OR114179.

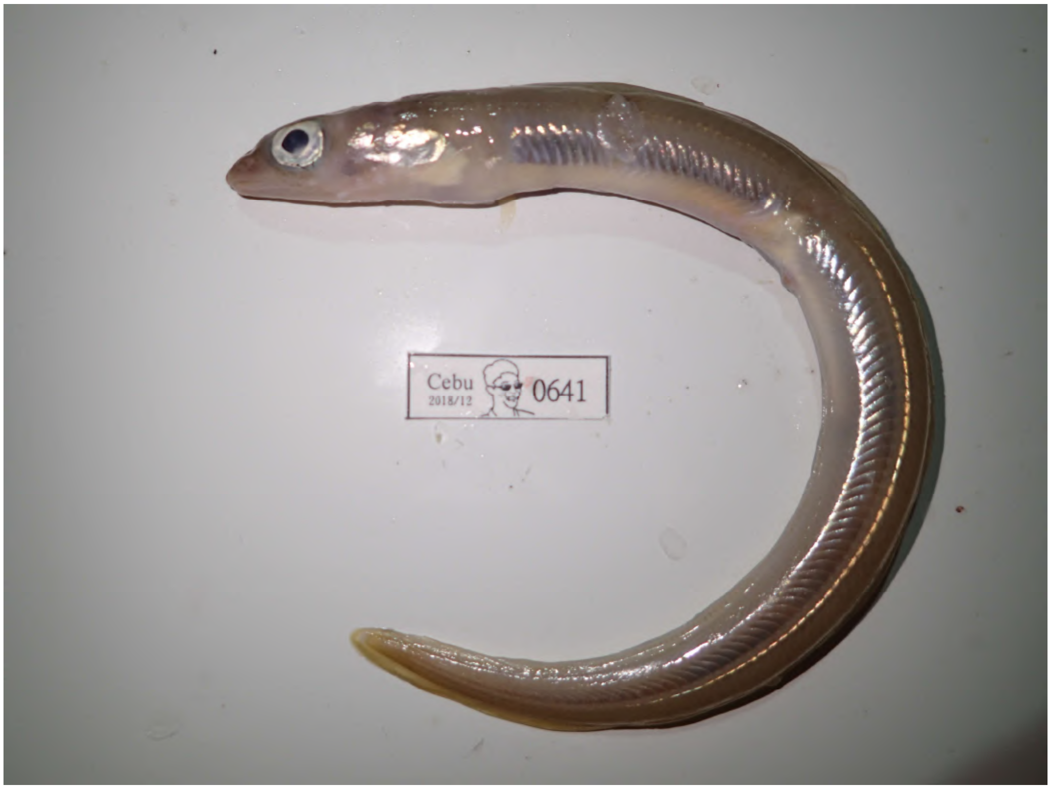

DOS 06679-1, *Ariosoma scheelei*, OR113869.

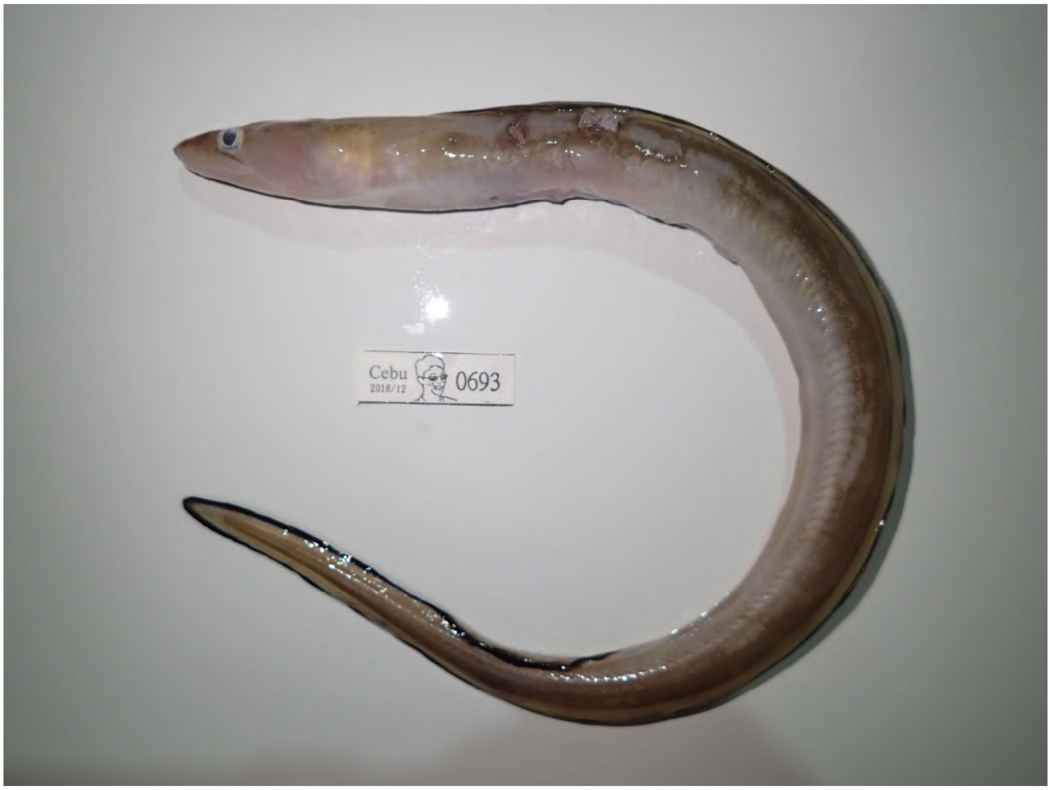

DOS 06680-1, *Conger cinereus*, OR113870.

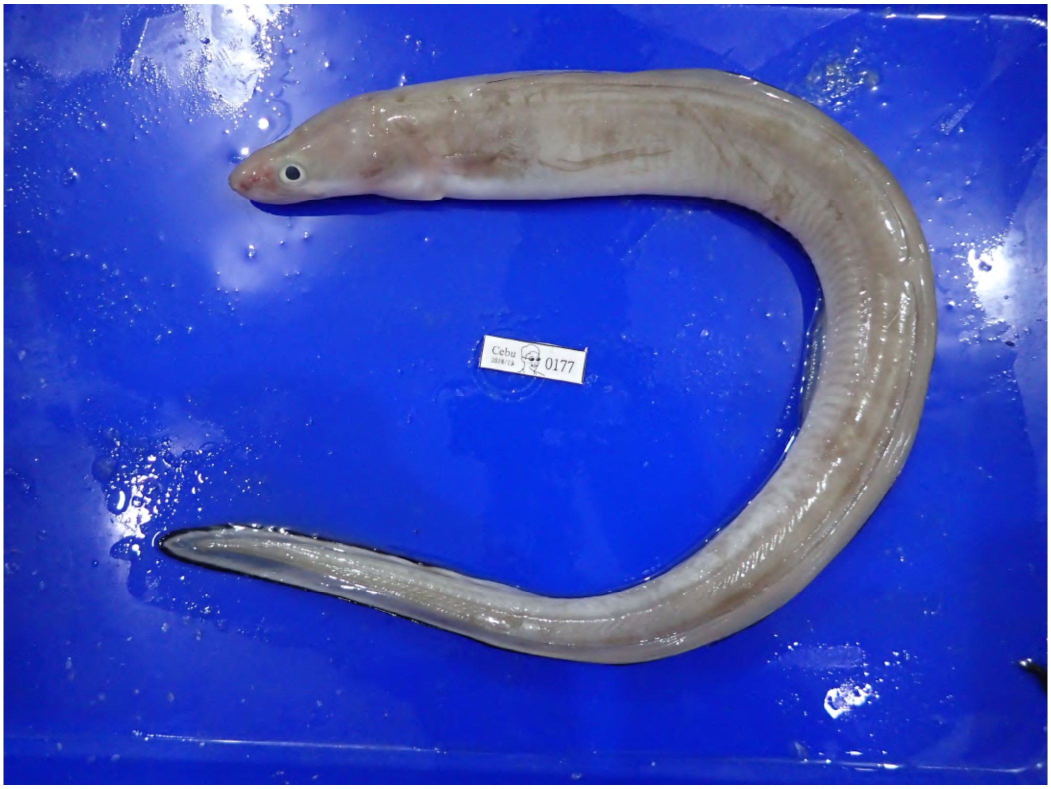

DOS 06819, *Conger cinereus*, OR114000. (specimen not preserved)

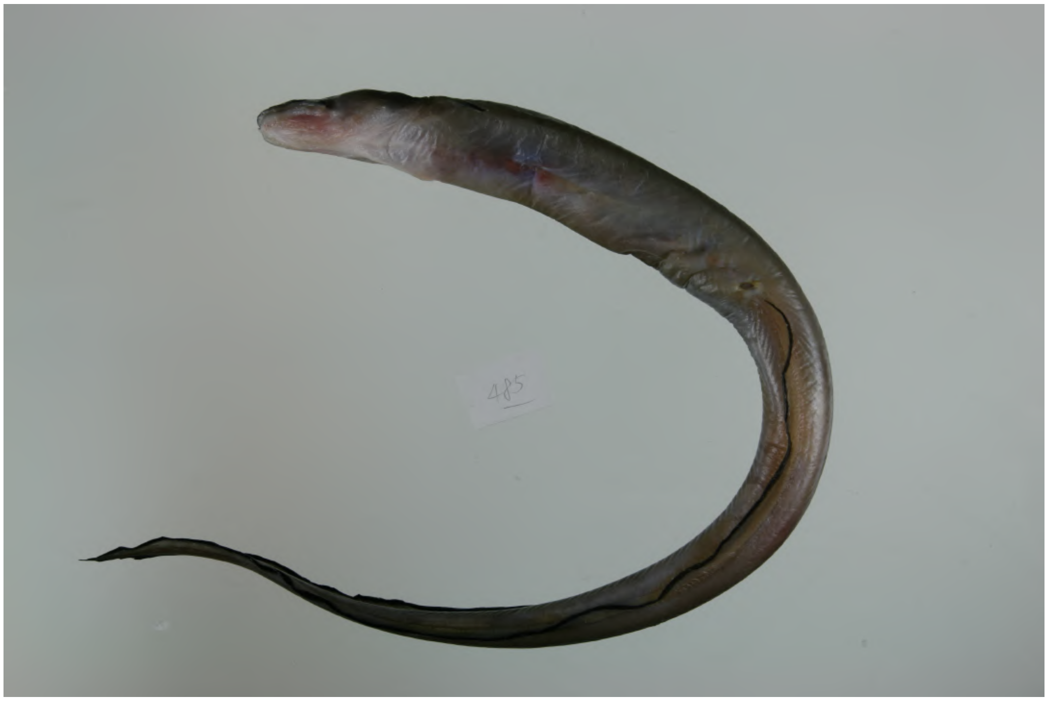

DOS 08616, *Conger cinereus*, OR114180.

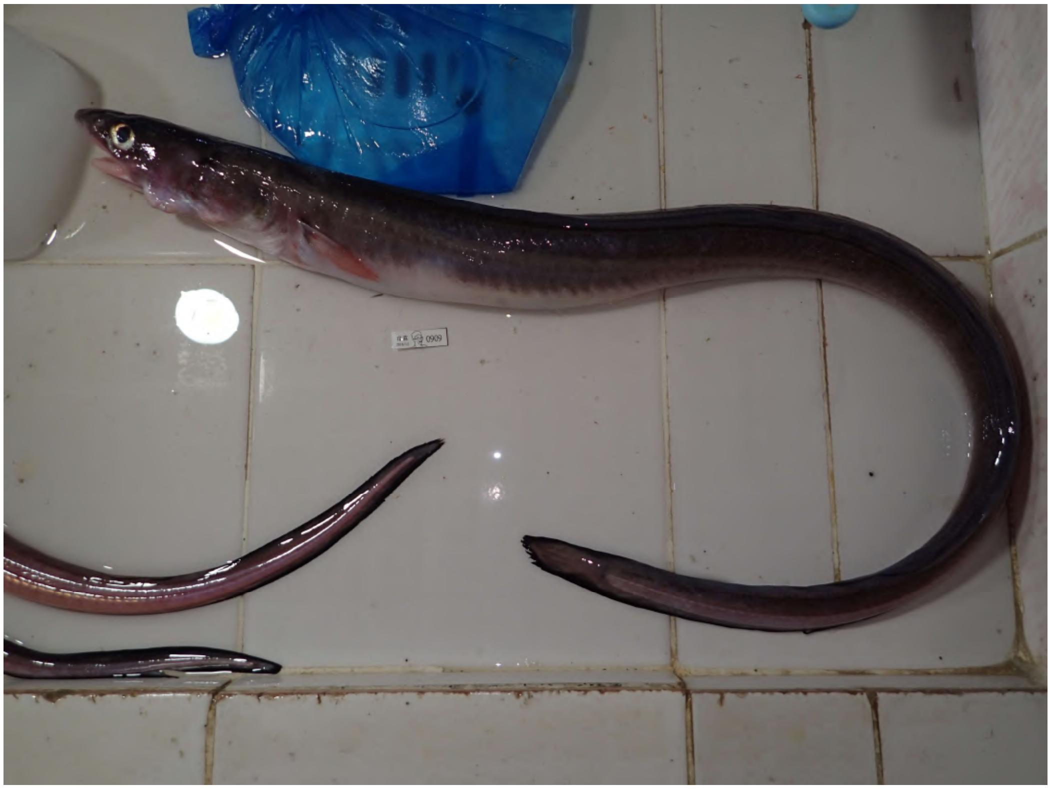

DOS 06681-1, *Rhynchoconger* sp., OR113871. (specimen not preserved)

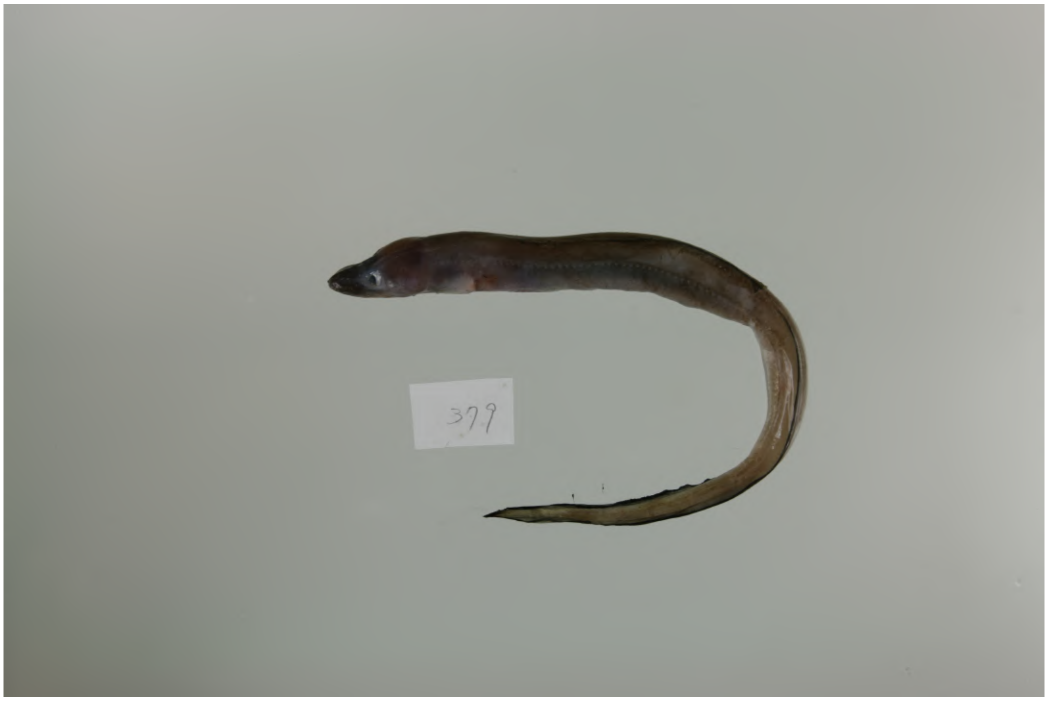

DOS 08617, *Uroconger lepturus*, OR114181.

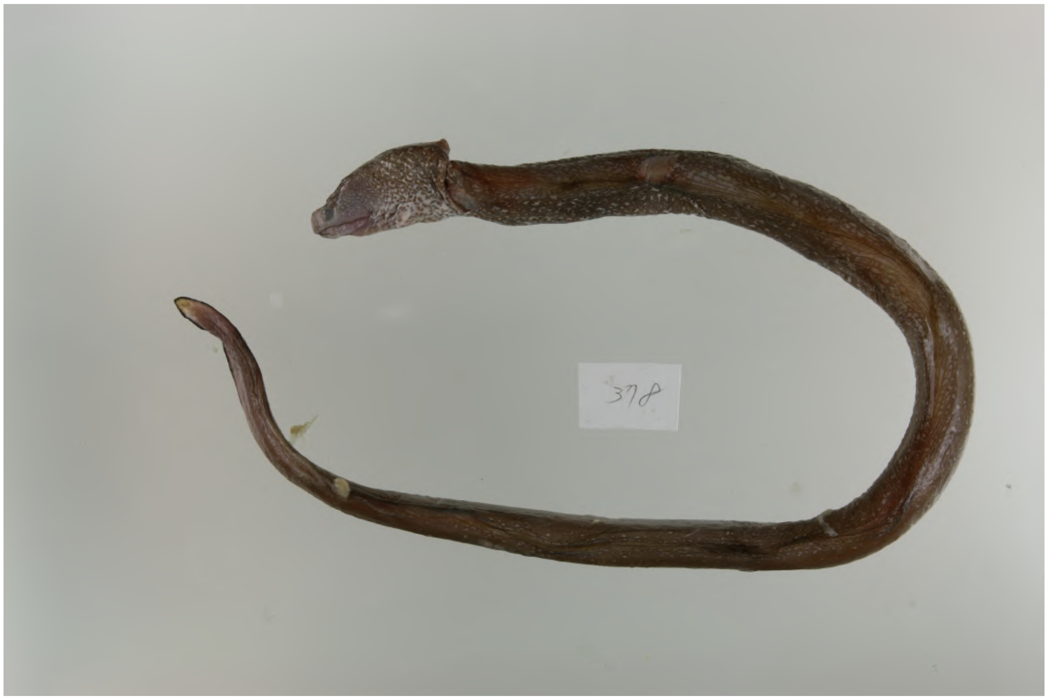

DOS 08618, *Diaphenchelys laimospila*, OR114182.

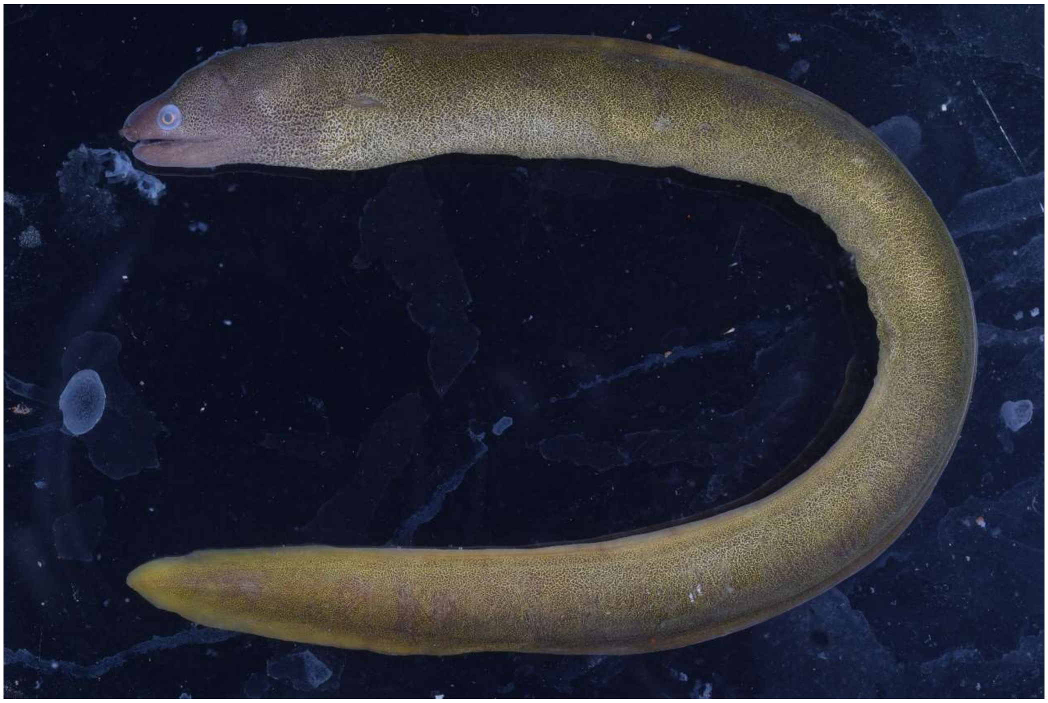

DOS 06820-1, *Echidna delicatula*, OR114001.

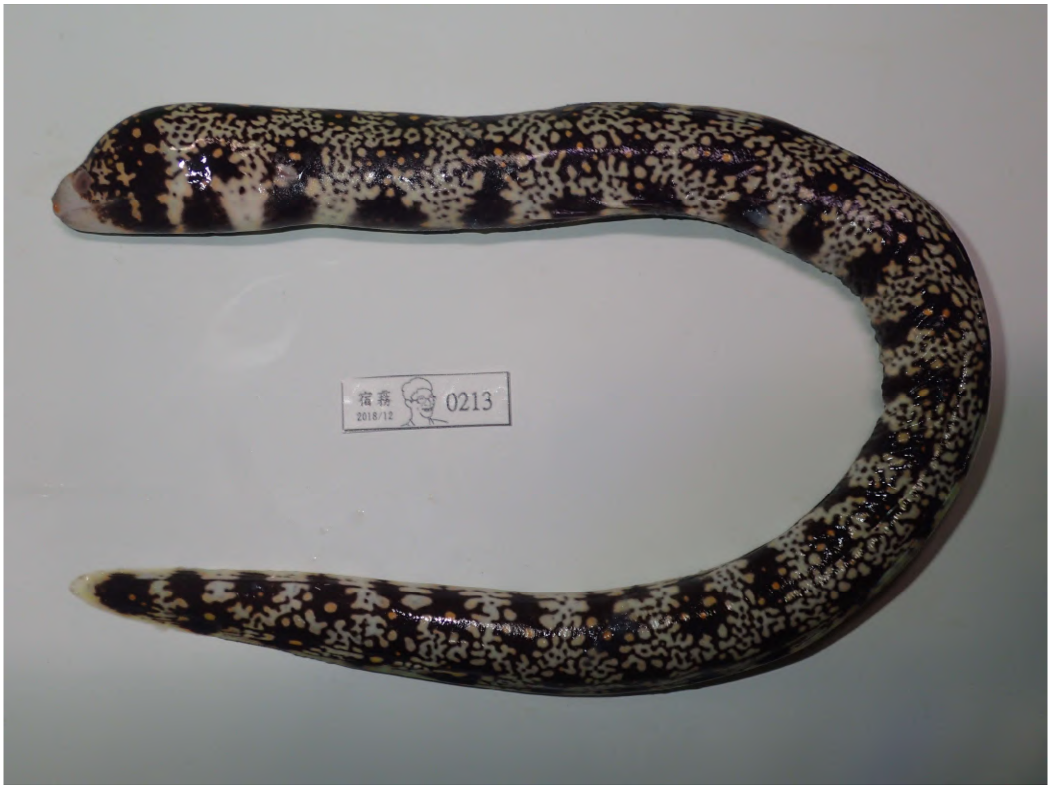

DOS 06821, *Echidna nebulosa*, OR114002.

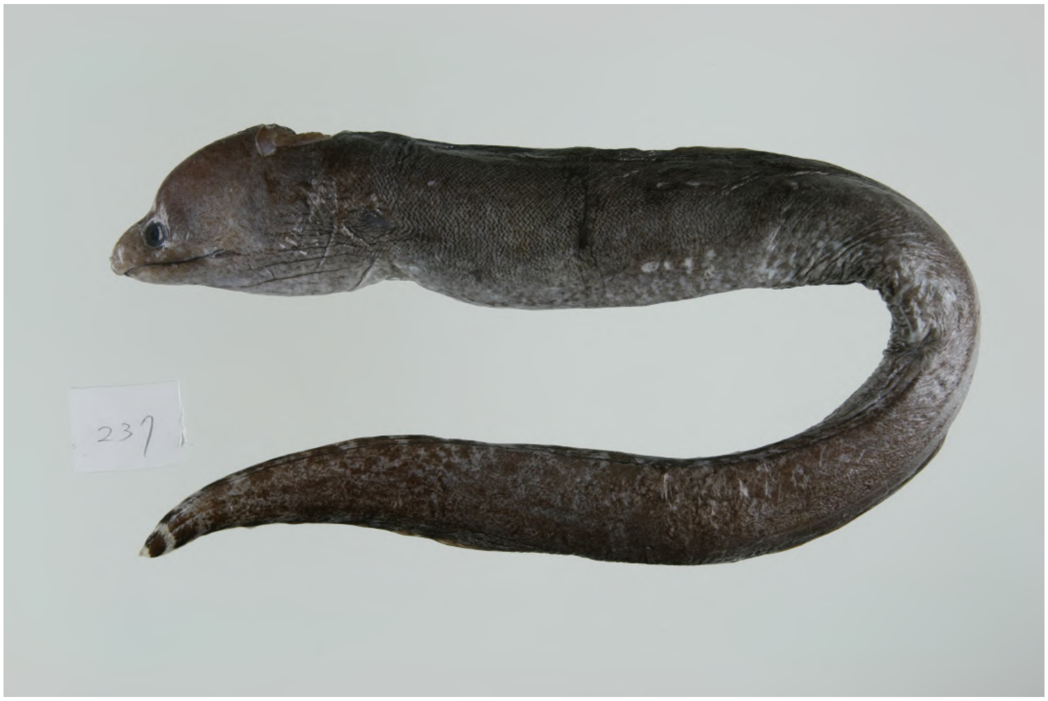

DOS 08619, *Echidna polyzona*, OR114183.

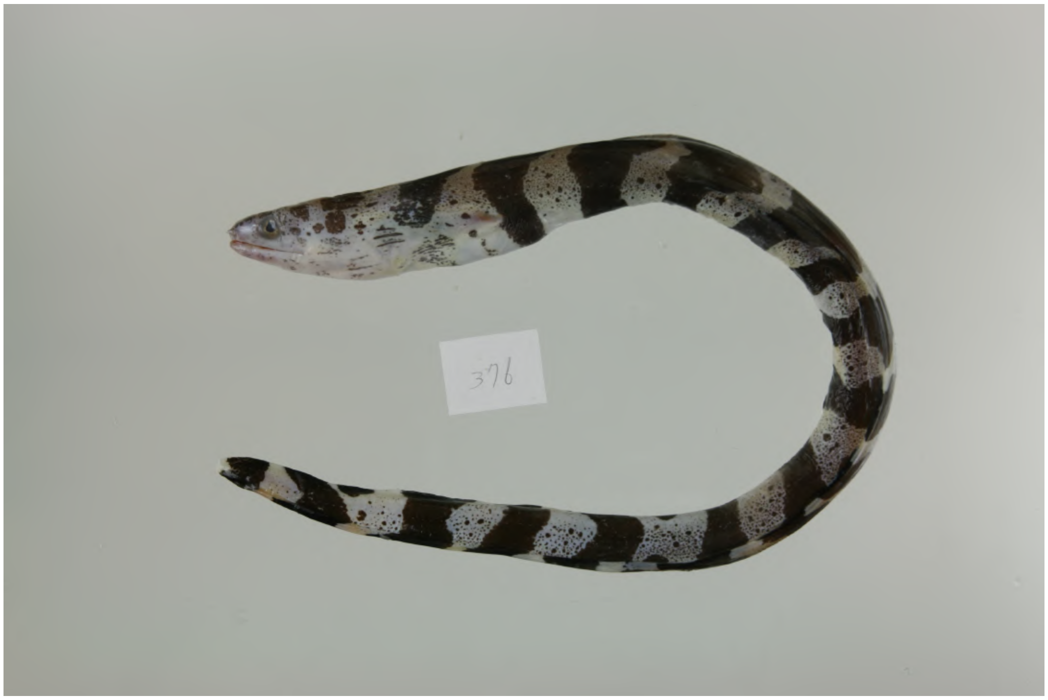

DOS 08621, *Gymnothorax annulatus*, OR114186.

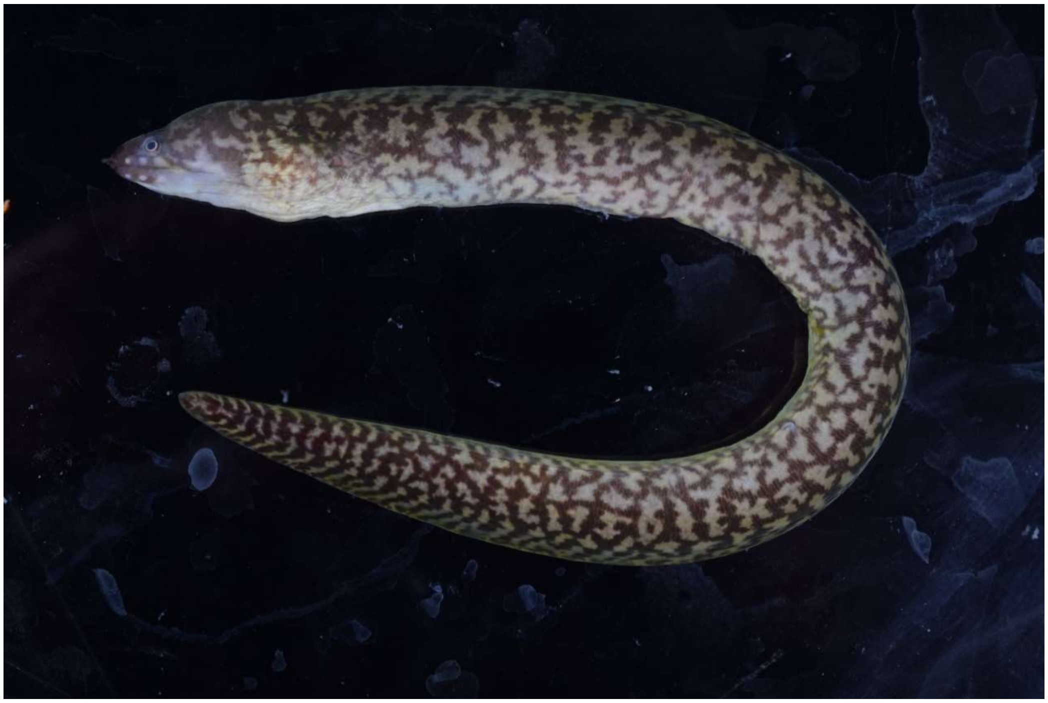

DOS 06822-1, *Gymnothorax* cf. *chilospilus*, OR114003.

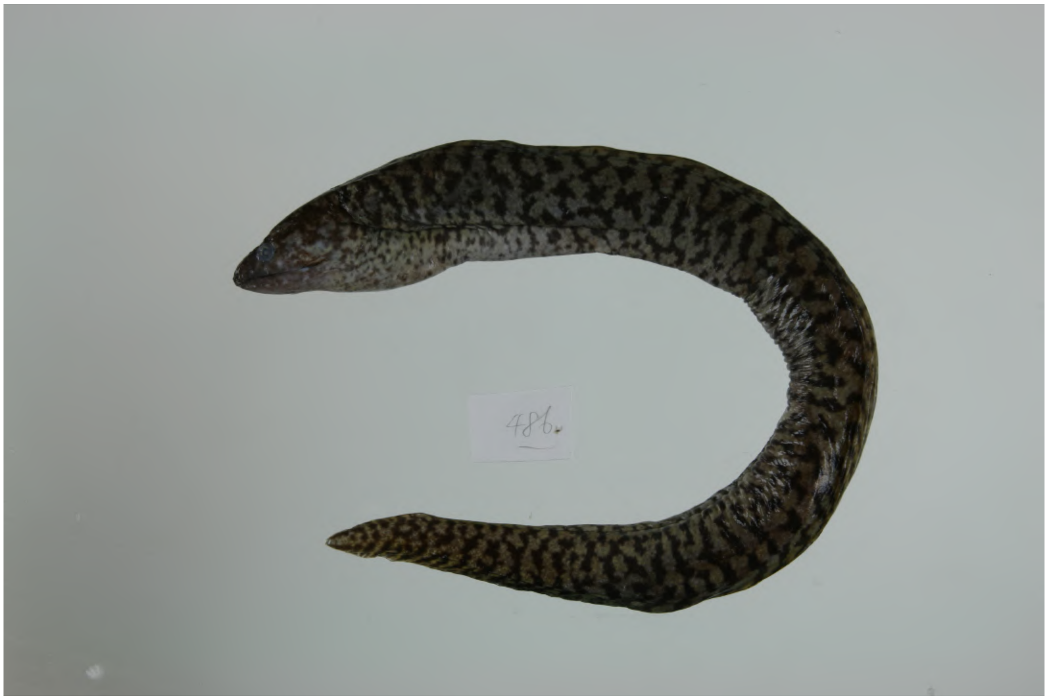

DOS 08620-1, *Gymnothorax* cf. *chilospilus*, OR114184.

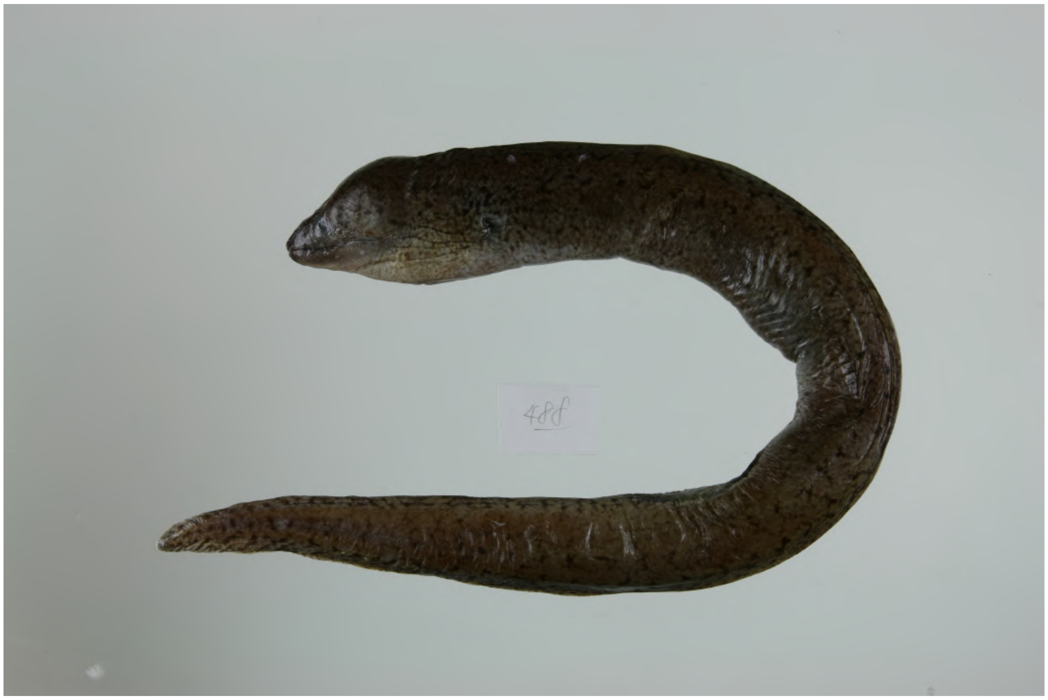

DOS 08620-2, *Gymnothorax* cf. *chilospilus*, OR114185.

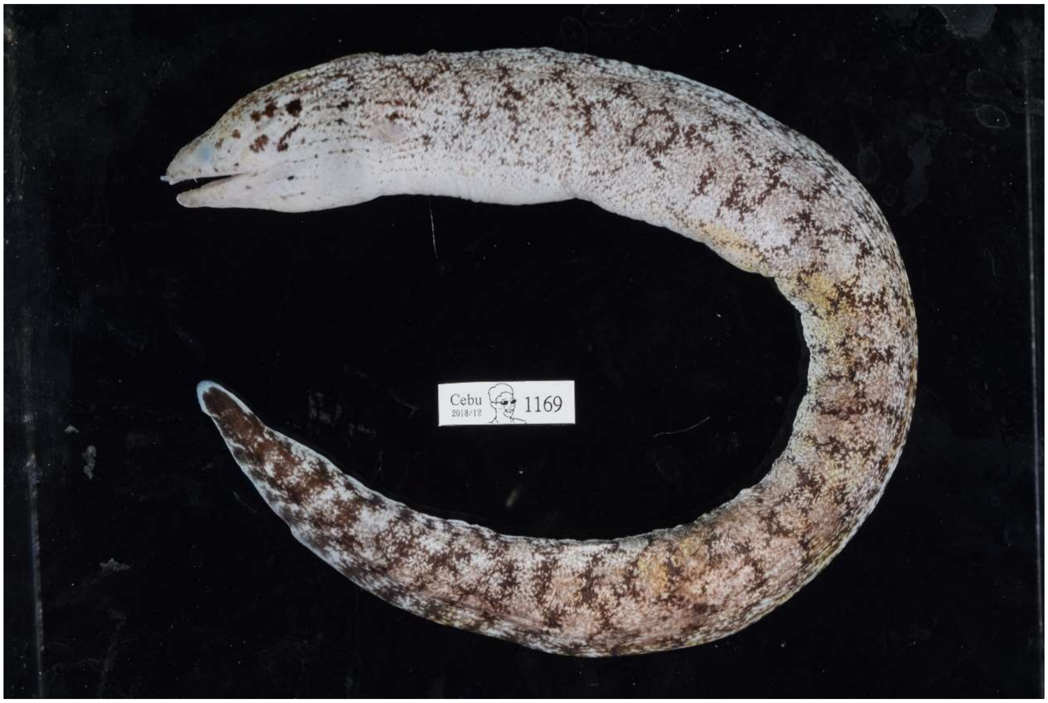

DOS 06823, *Gymnothorax* cf. *cribroris*, OR114004.

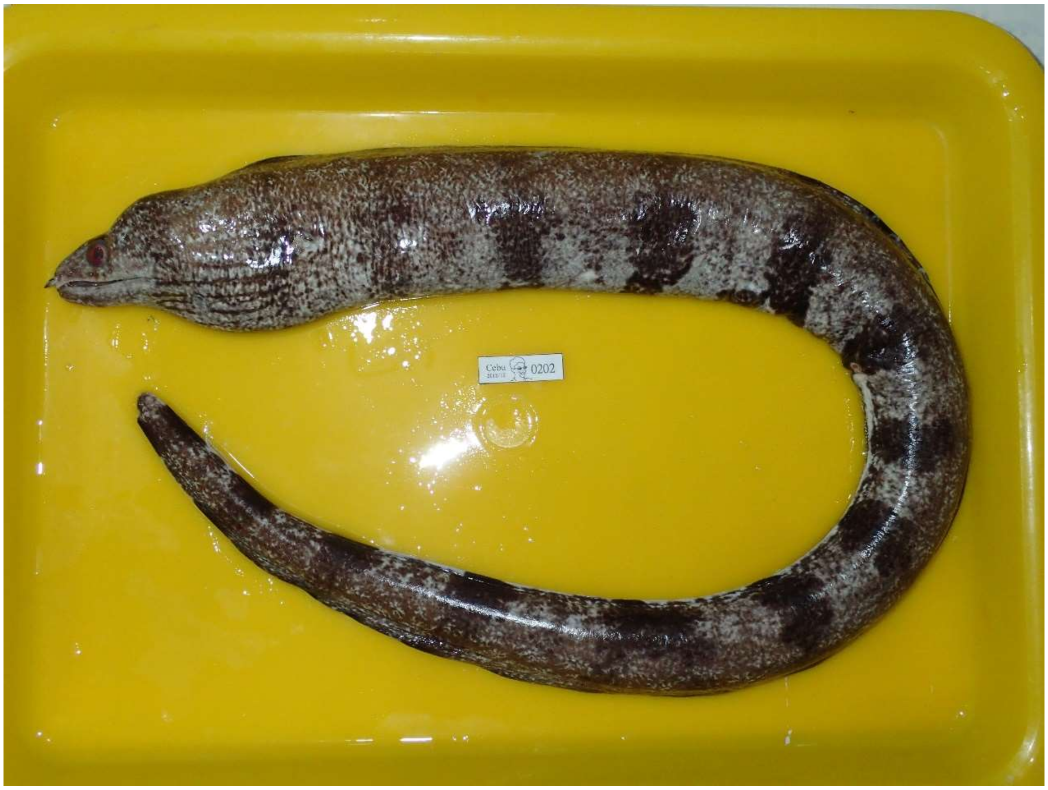

DOS 06824, *Gymnothorax enigmaticus*, OM037461.

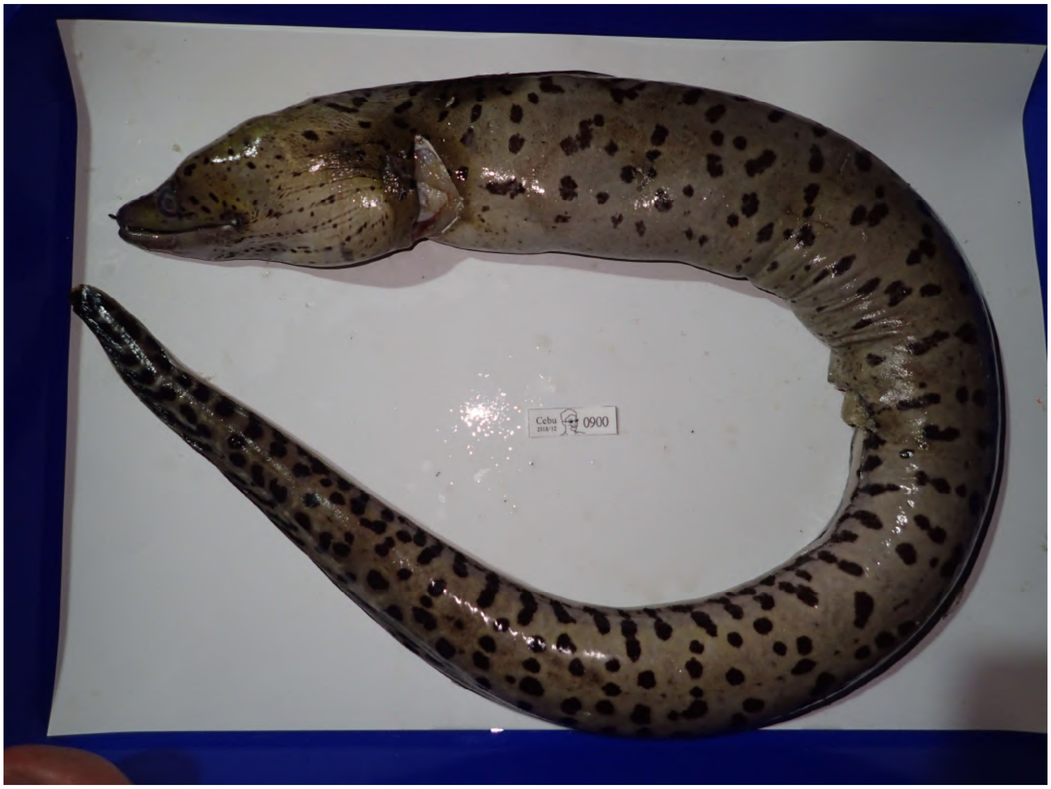

DOS 06826-1, *Gymnothorax fimbriatus*, OR114006.

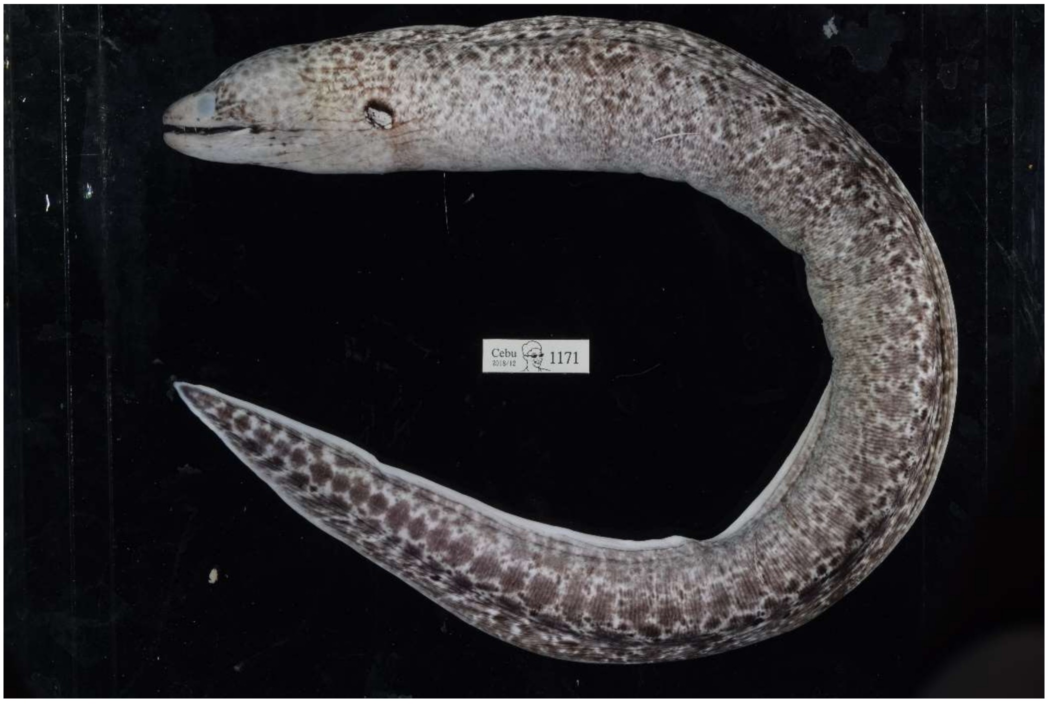

DOS 06827, *Gymnothorax formosus*, OR114007.

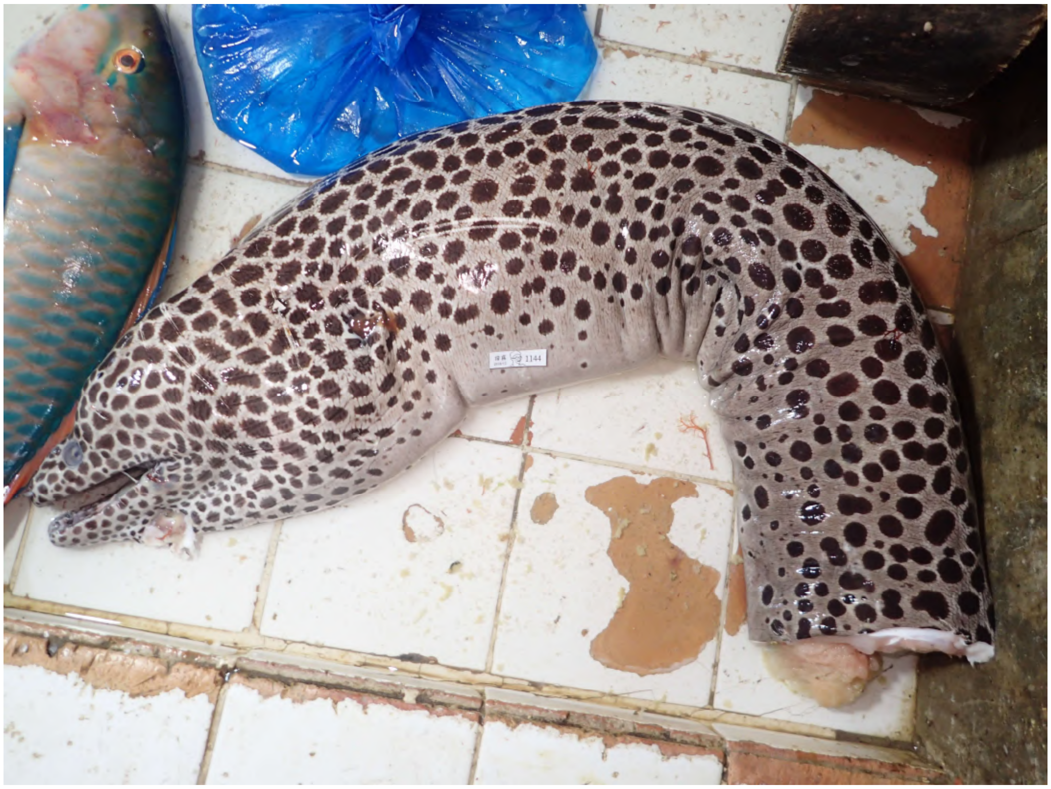

DOS 06825, *Gymnothorax isingteena*, OR114005. (specimen not preserved)

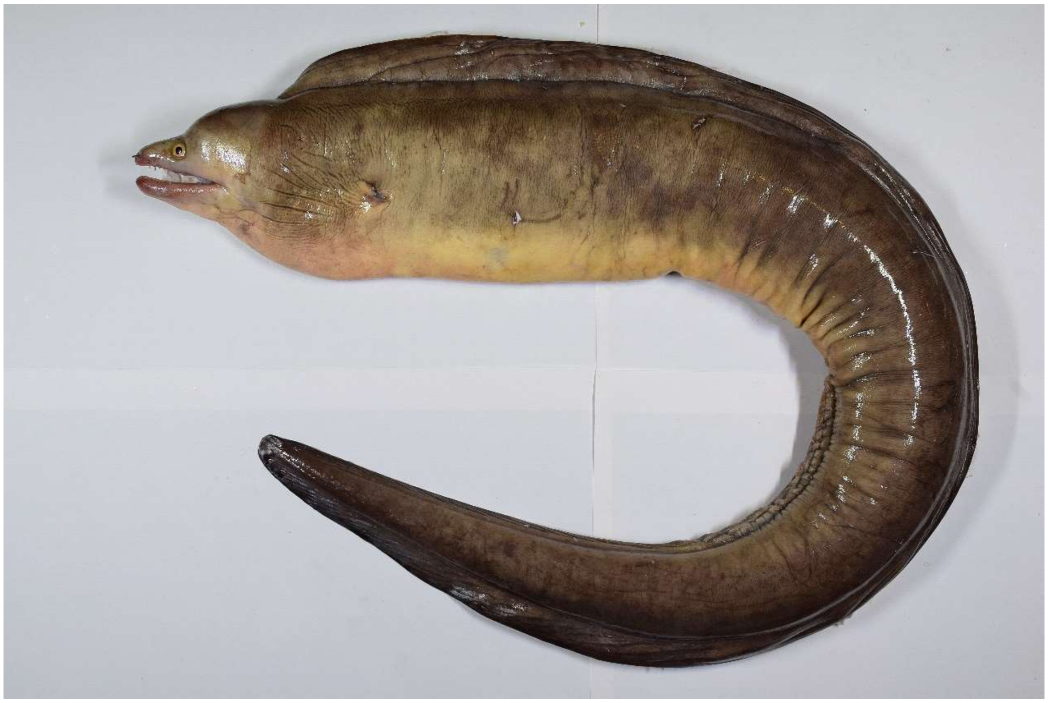

DOS 06828-1, *Gymnothorax monochrous*, ON351491.

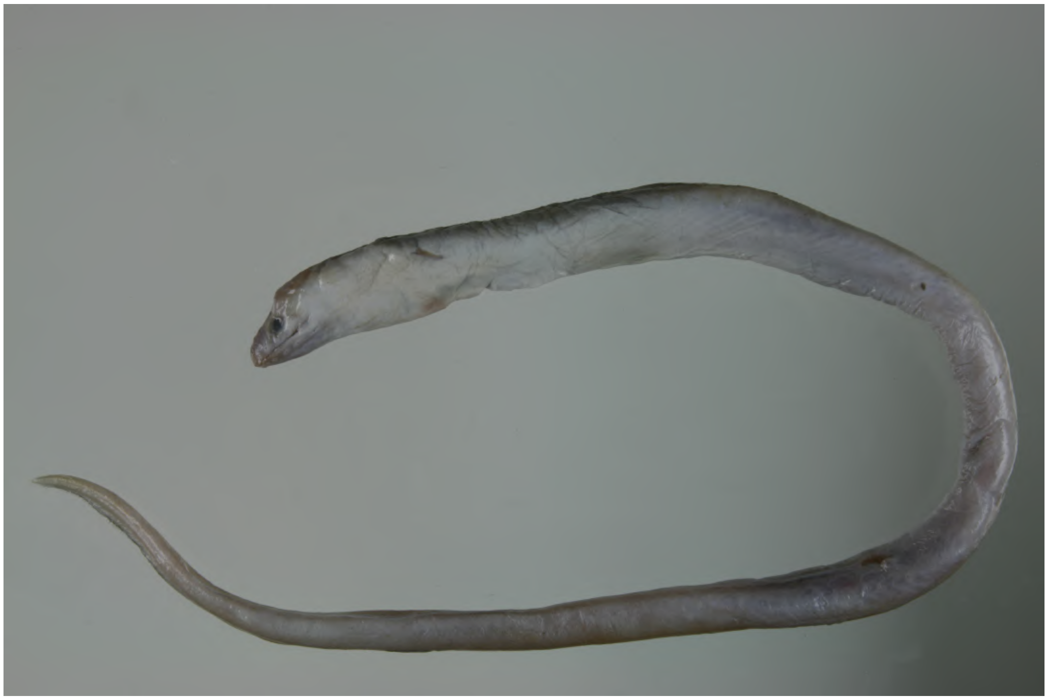

DOS 08622, *Gymnothorax phasmatodes*, OR114187.

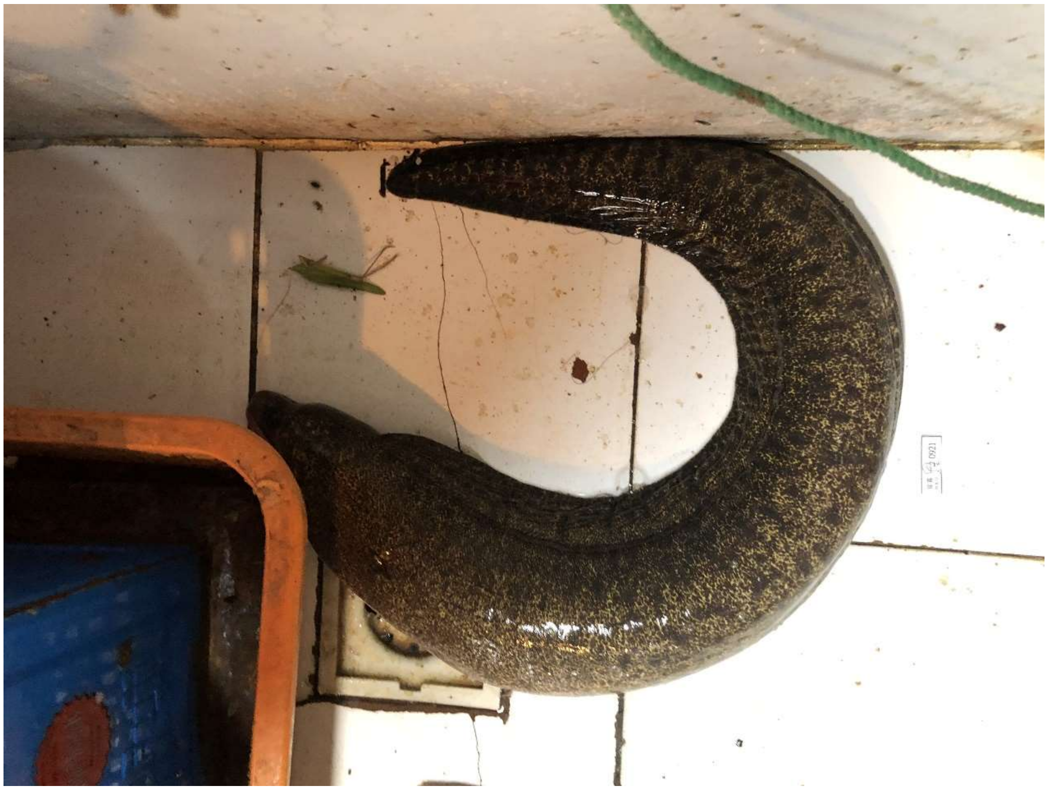

DOS 06829-1, *Gymnothorax pseudothyrsoideus*, OM037539. (specimen not preserved)

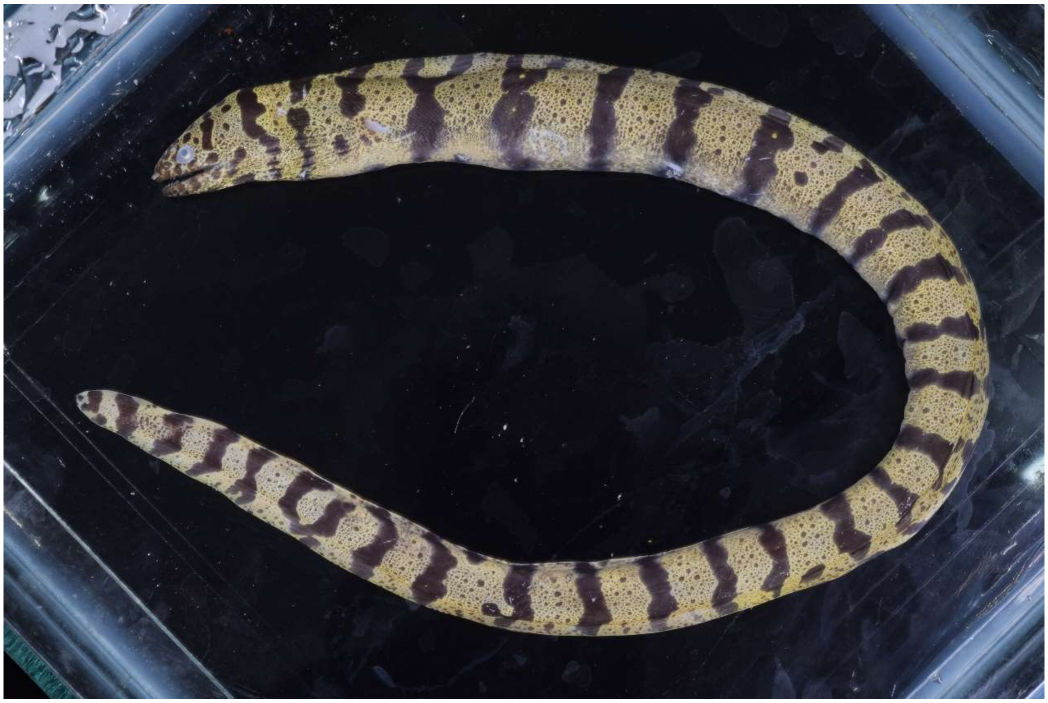

DOS 06830-1, *Gymnothorax punctatofasciatus*, OR114008.

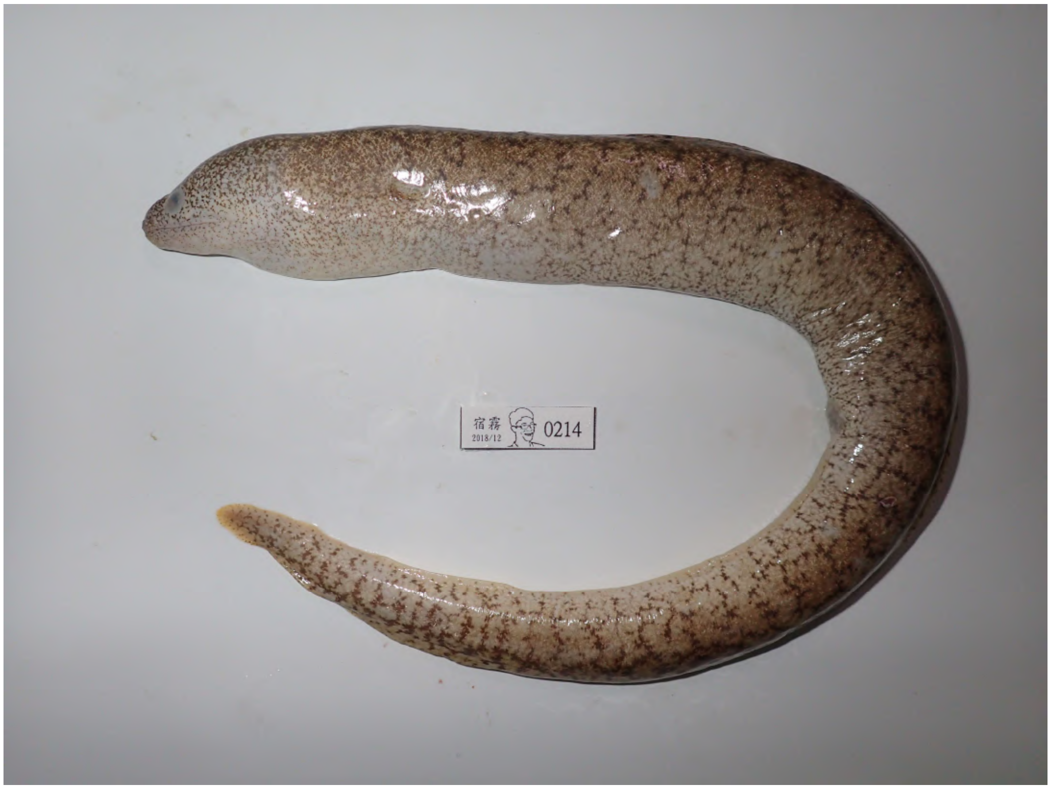

DOS 06831-1, *Gymnothorax richardsonii*, OR114009.

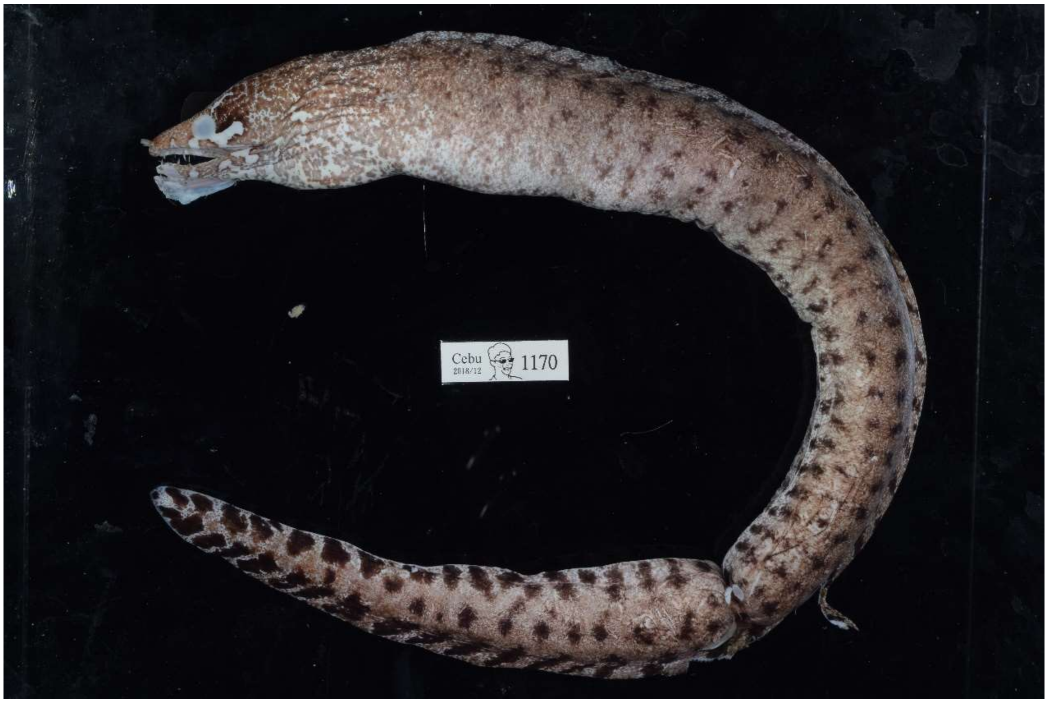

DOS 06832, *Gymnothorax zonipectis*, OM037474.

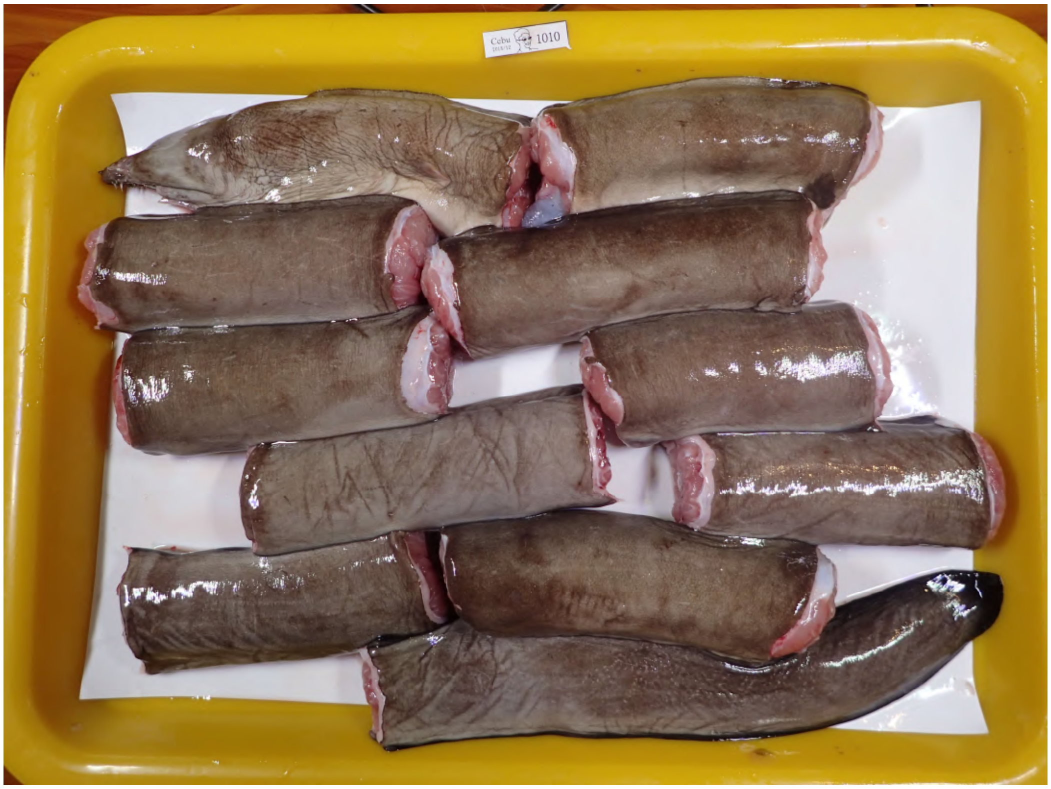

DOS 06833, *Strophidon sathete*, OR114010.

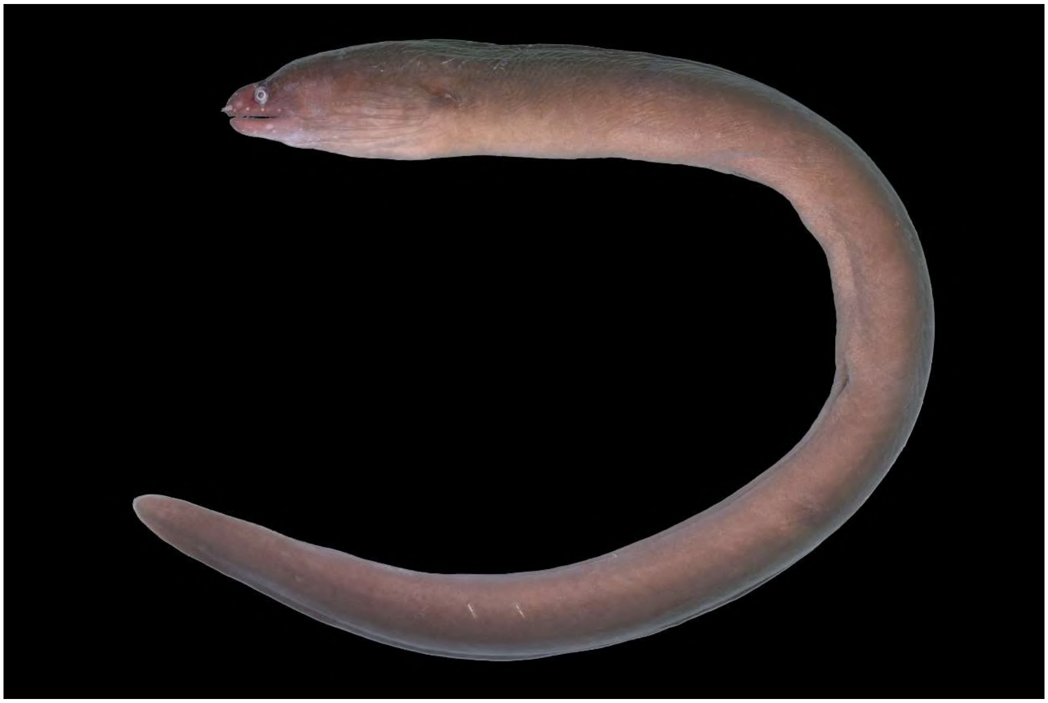

PNM 15711, *Uropterygius* sp., OQ508847.

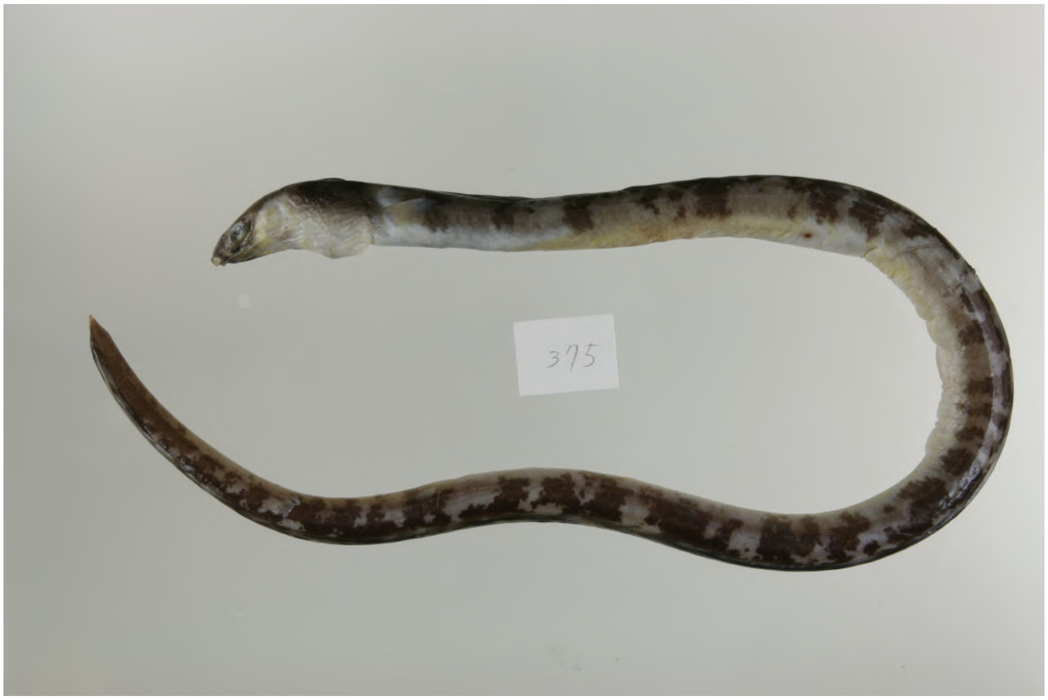

DOS 08623, *Ophichthus lithinus*, OR114188.

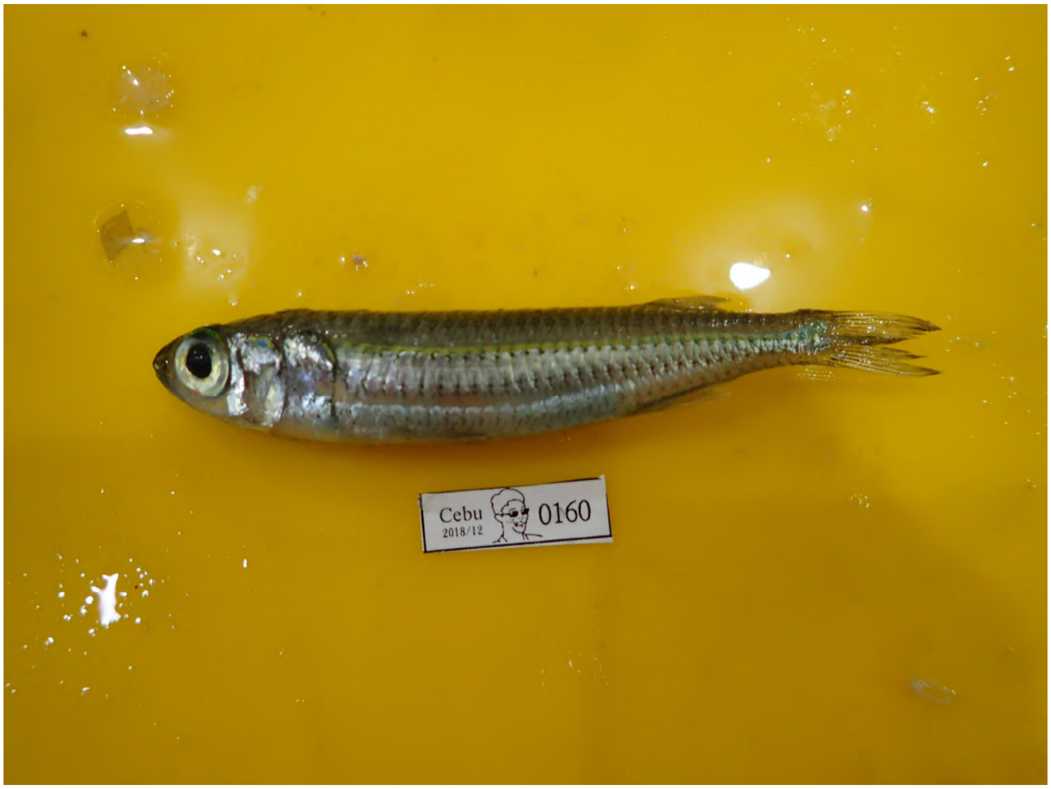

DOS 06603-1, *Doboatherina balabacensis*, OR113794.

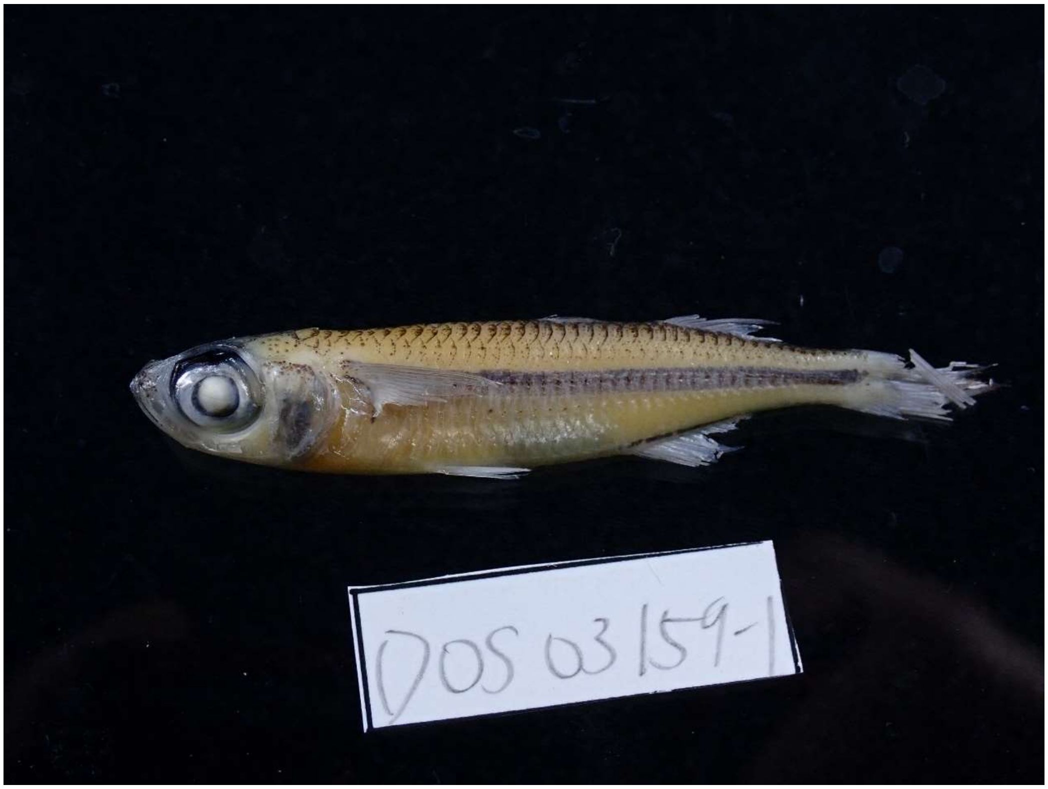

DOS 03159-1, *Doboatherina duodecimalis*, OR113754.

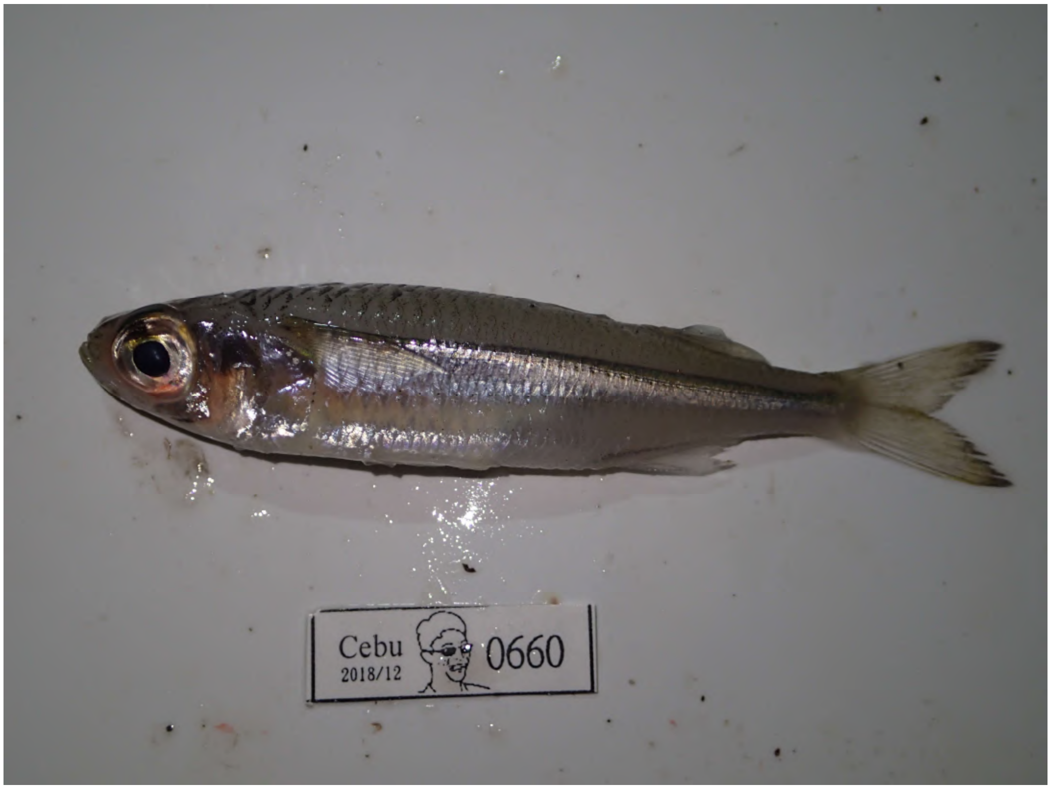

DOS 06602-1, *Doboatherina duodecimalis*, OR113793. (specimen not preserved)

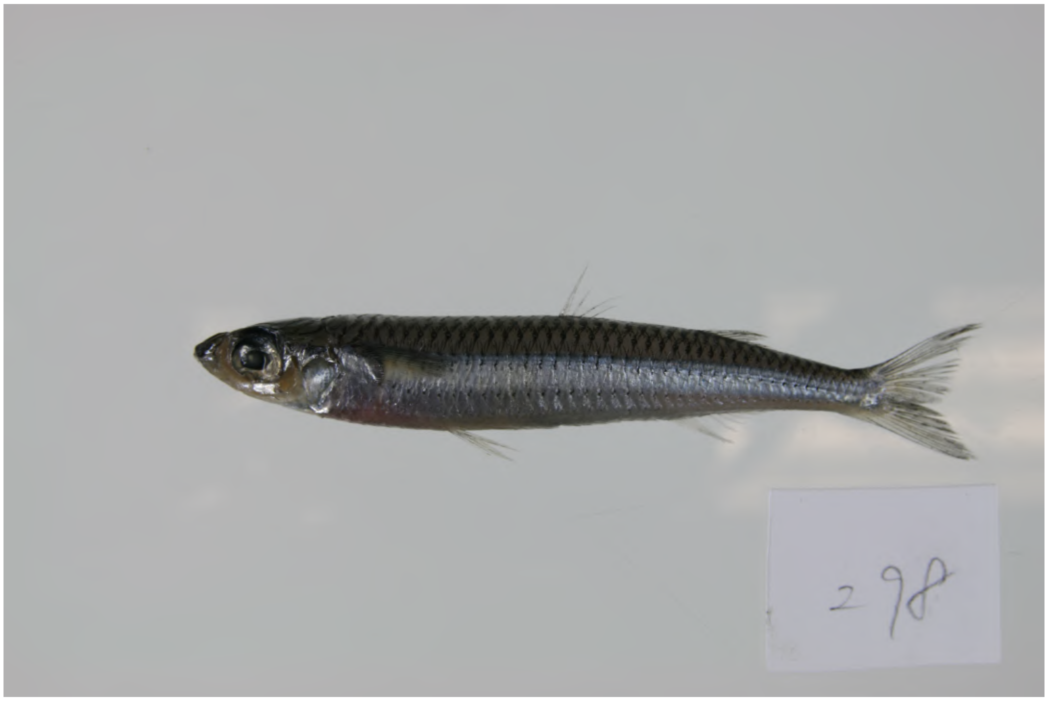

DOS 08624, *Hypoatherina temminckii*, OR114189.

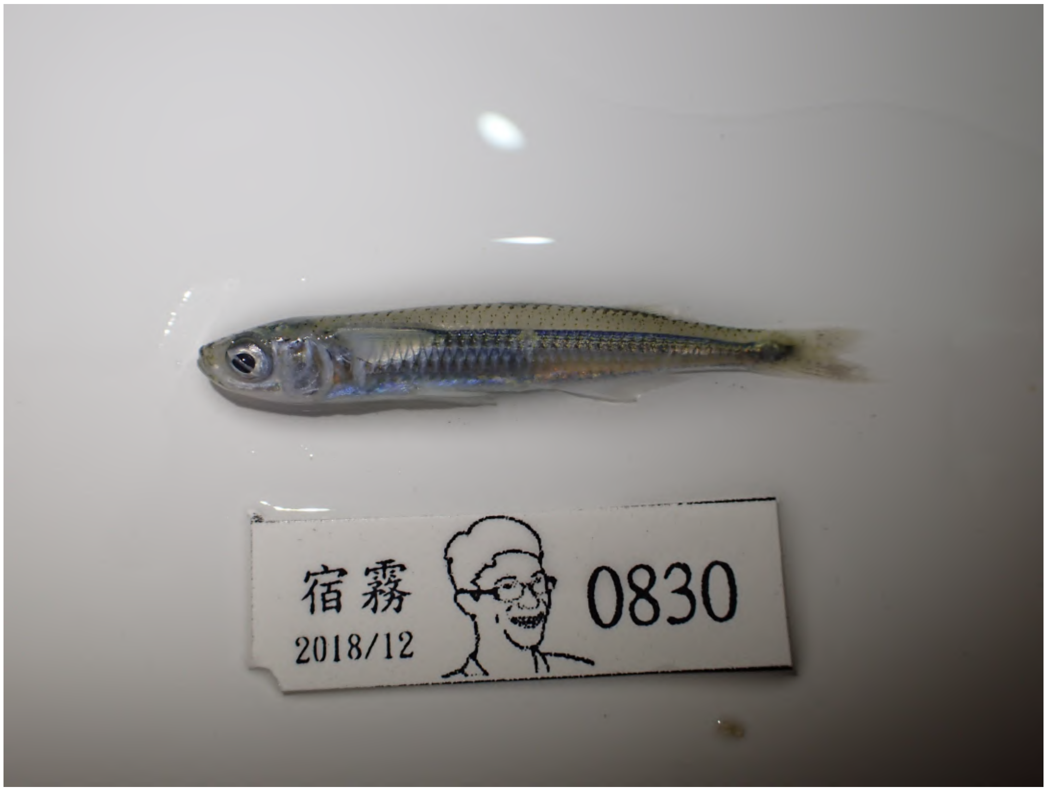

DOS 06604-1, *Atherion elymus*, OR113795.

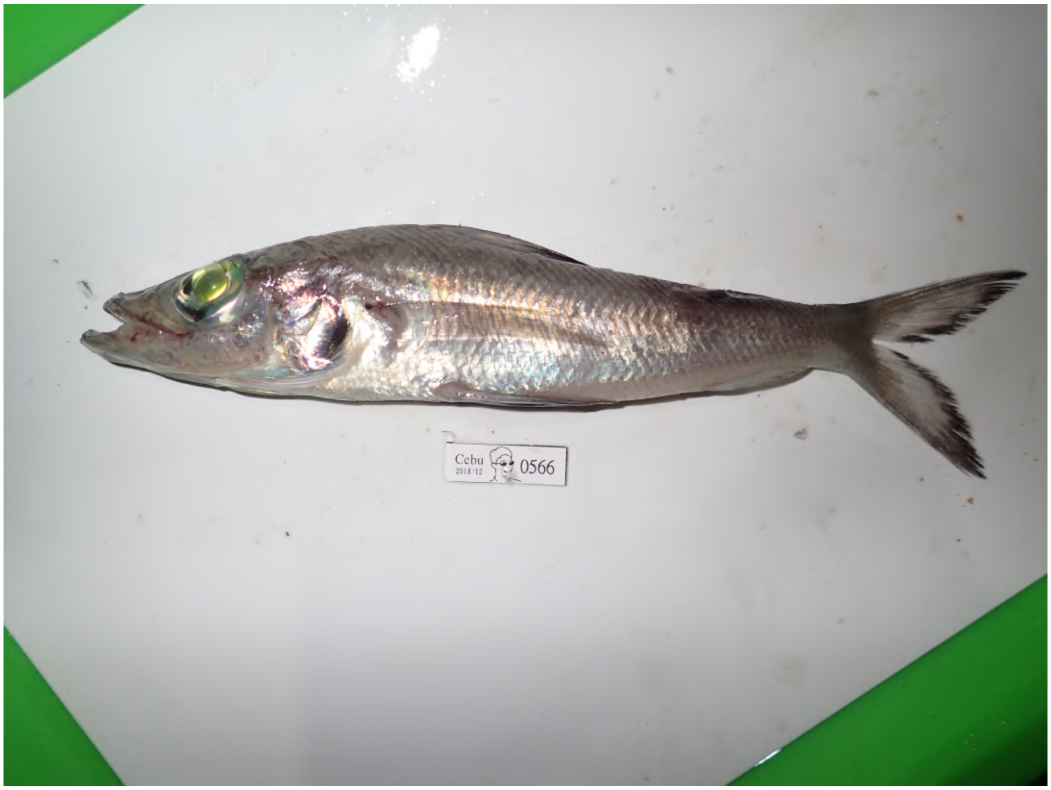

DOS 06674-1, *Chlorophthalmus* sp., OR113865.

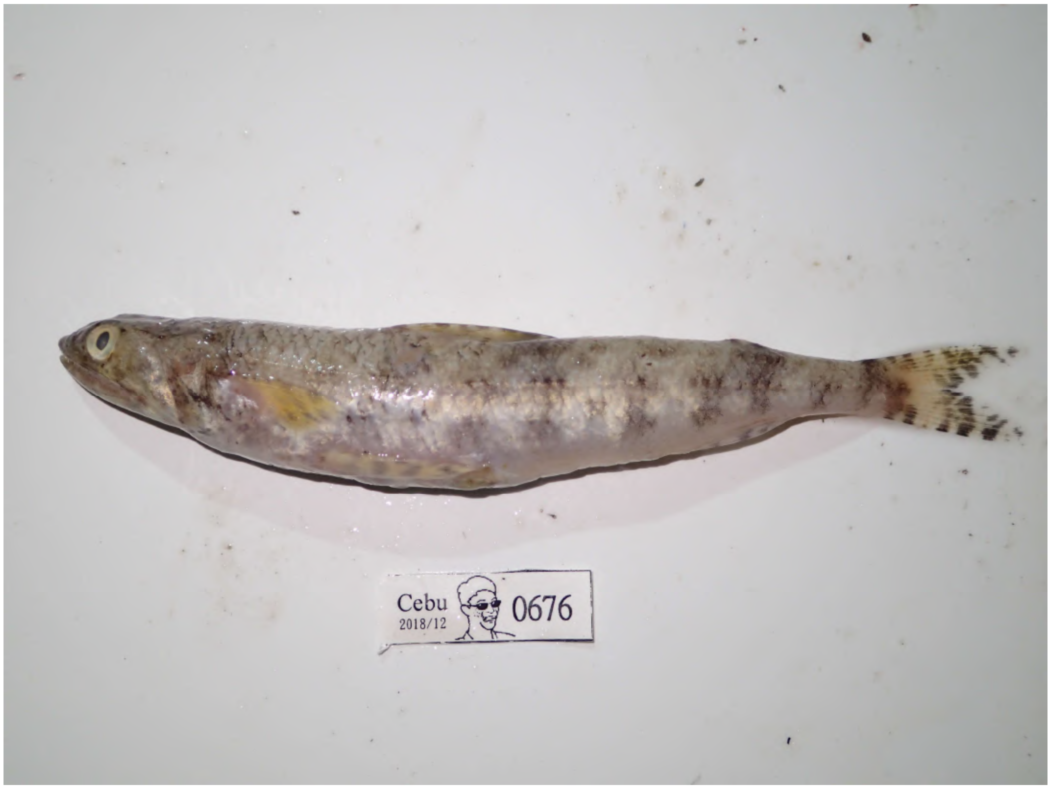

DOS 06975, *Saurida gracilis*, OR114147.

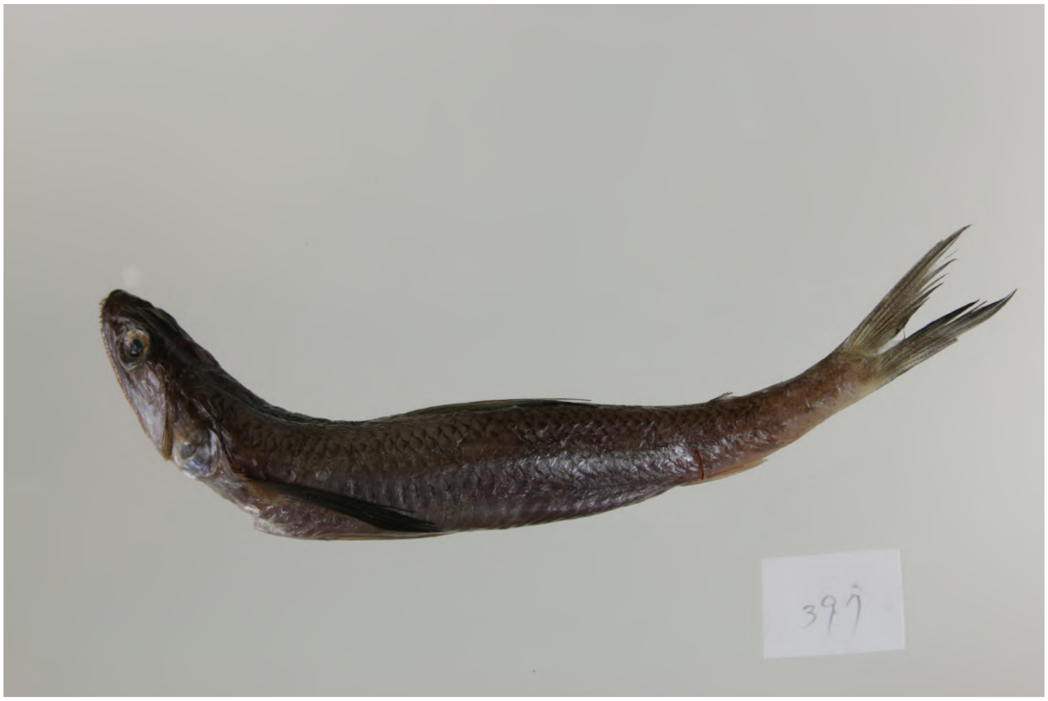

DOS 08625-1, *Saurida longimanus*, OR114190.

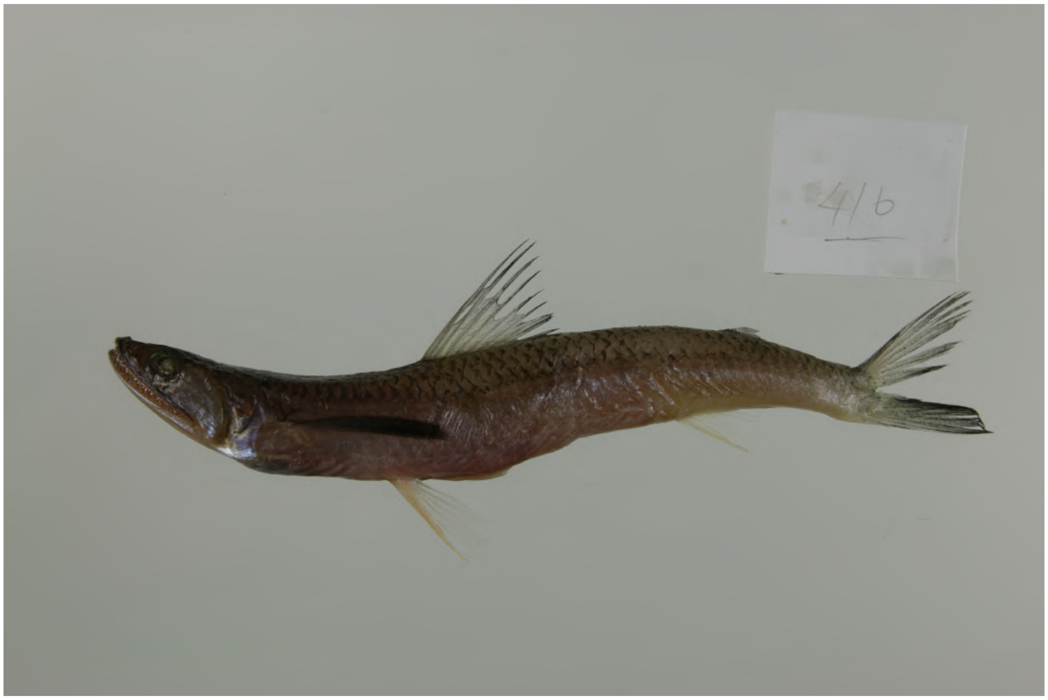

DOS 08625-2, *Saurida longimanus*, OR114191.

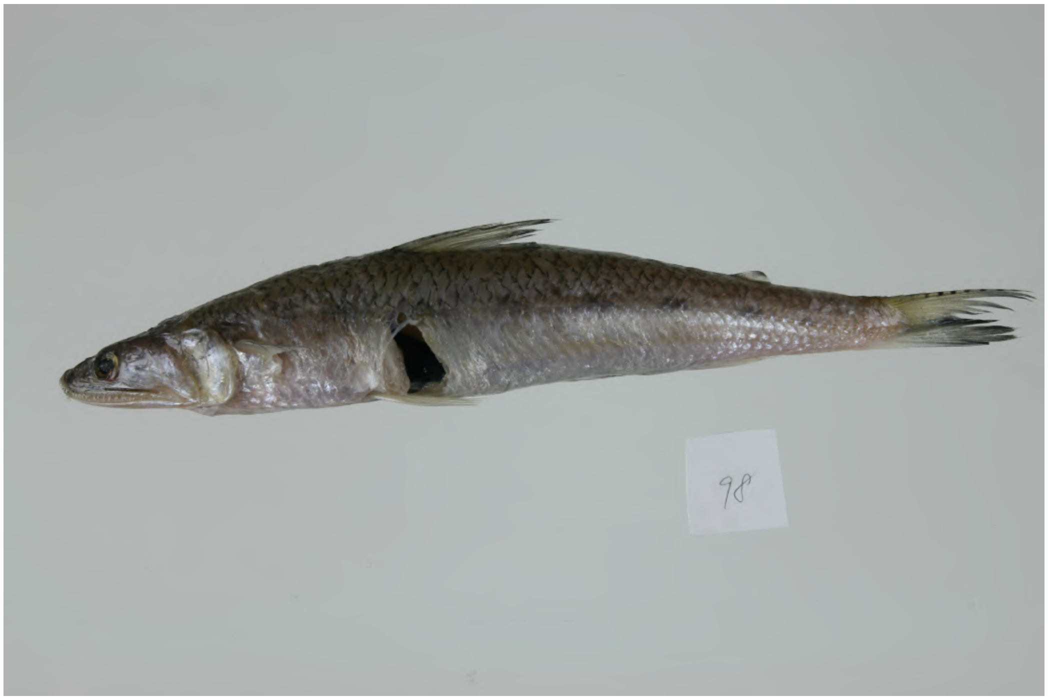

DOS 08626-1, *Saurida undosquamis*, OR114192.

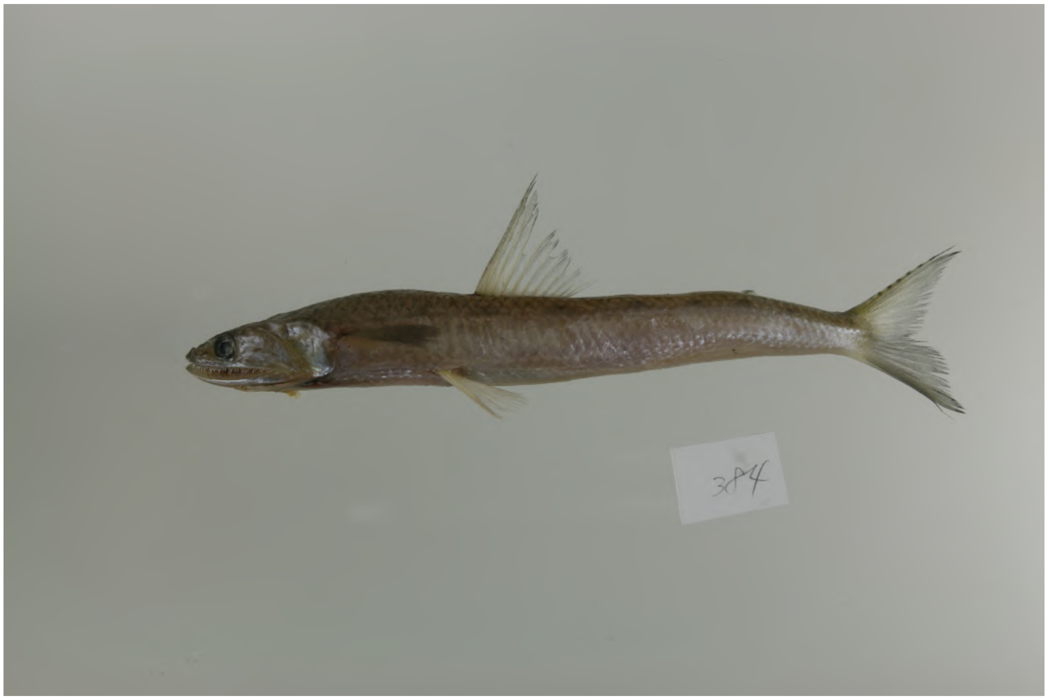

DOS 08626-2, *Saurida undosquamis*, OR114193.

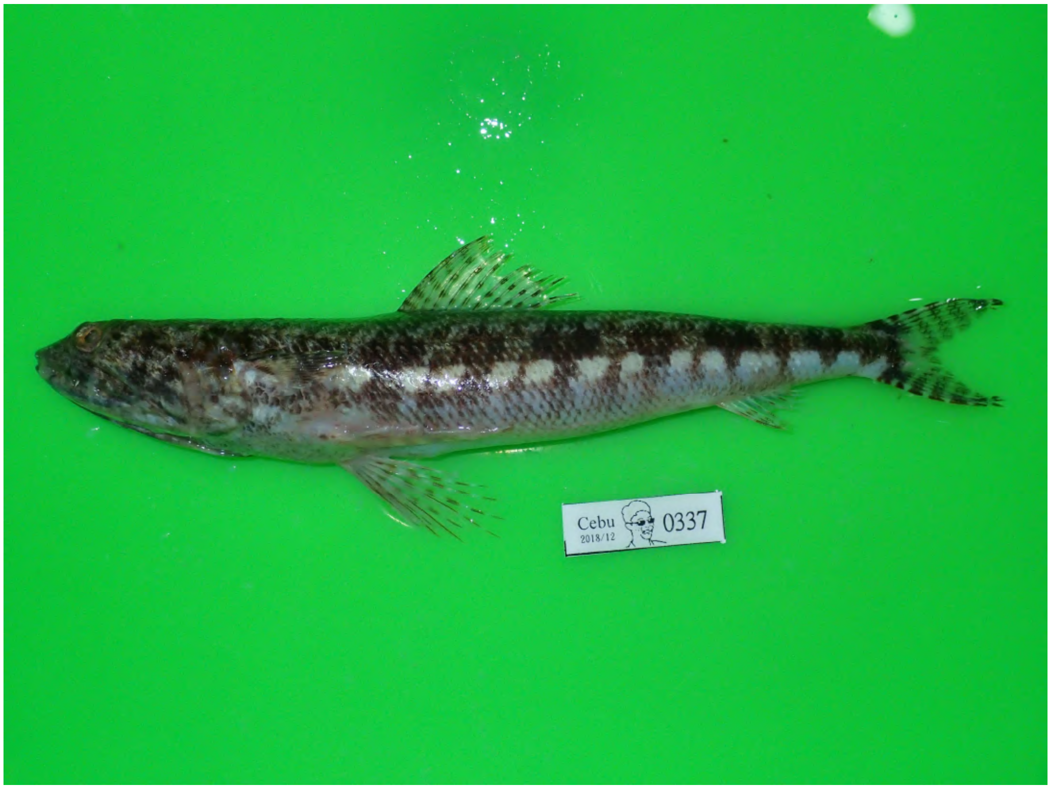

DOS 06973-1, *Synodus variegatus*, OR114145. (specimen not preserved)

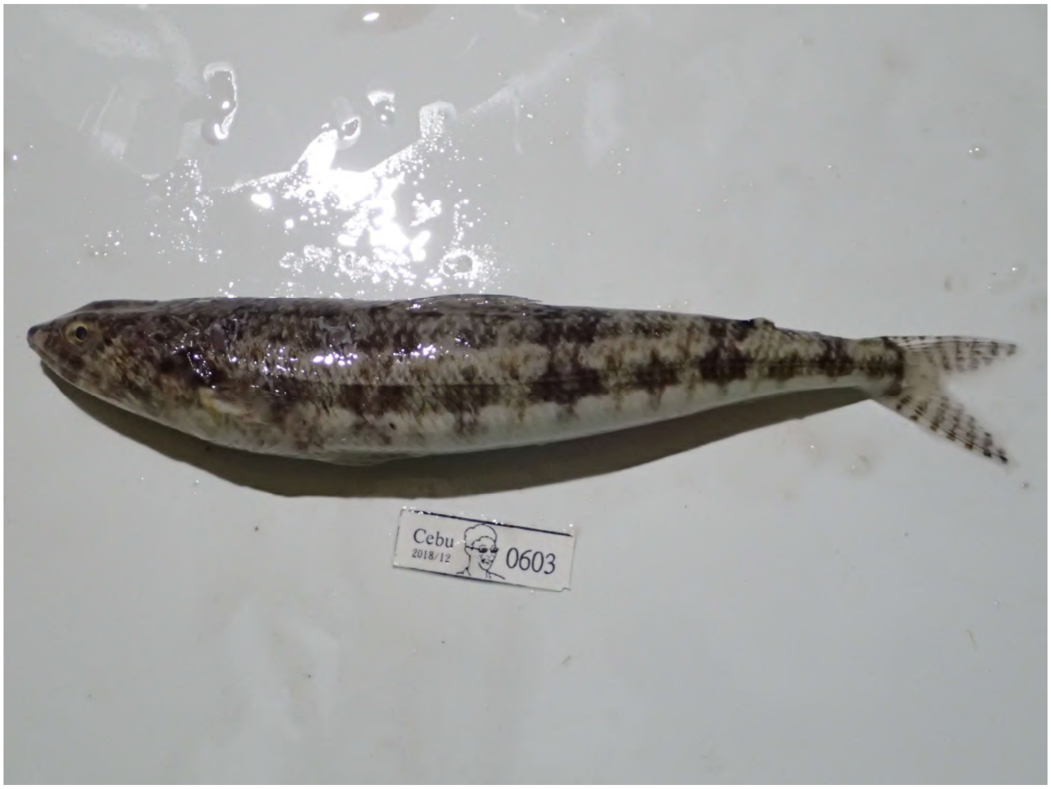

DOS 06974-1, *Synodus variegatus*, OR114146.

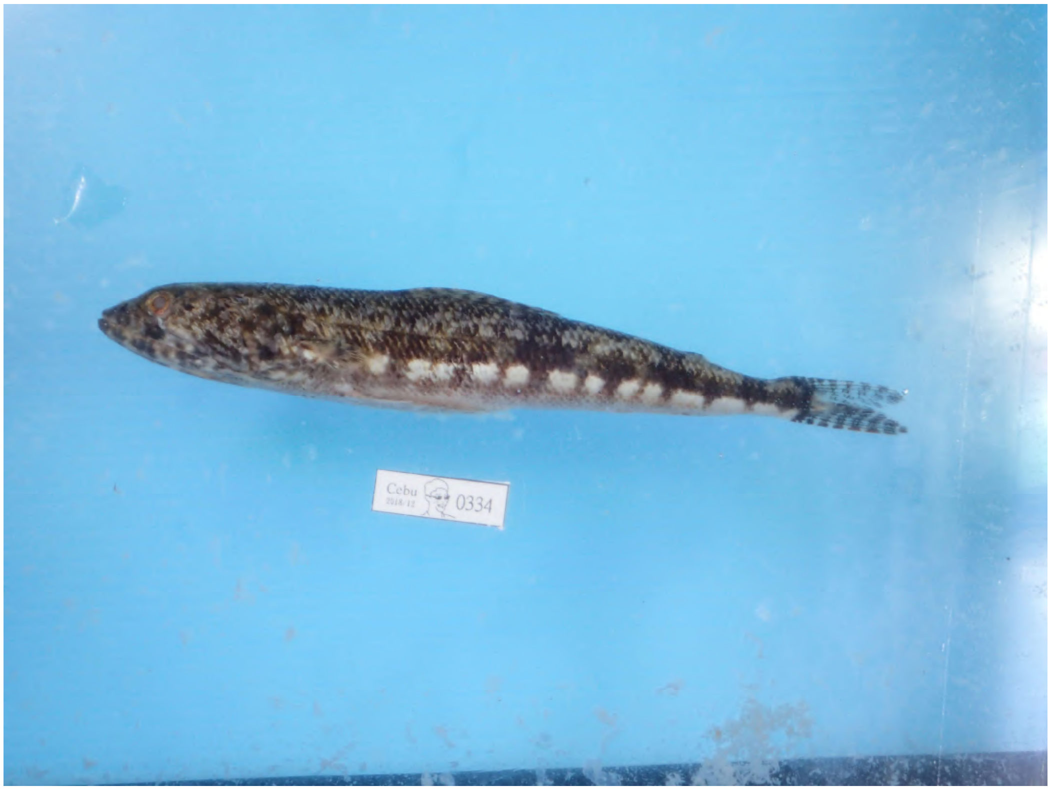

DOS 06978-1, *Synodus variegatus*, OR114148.

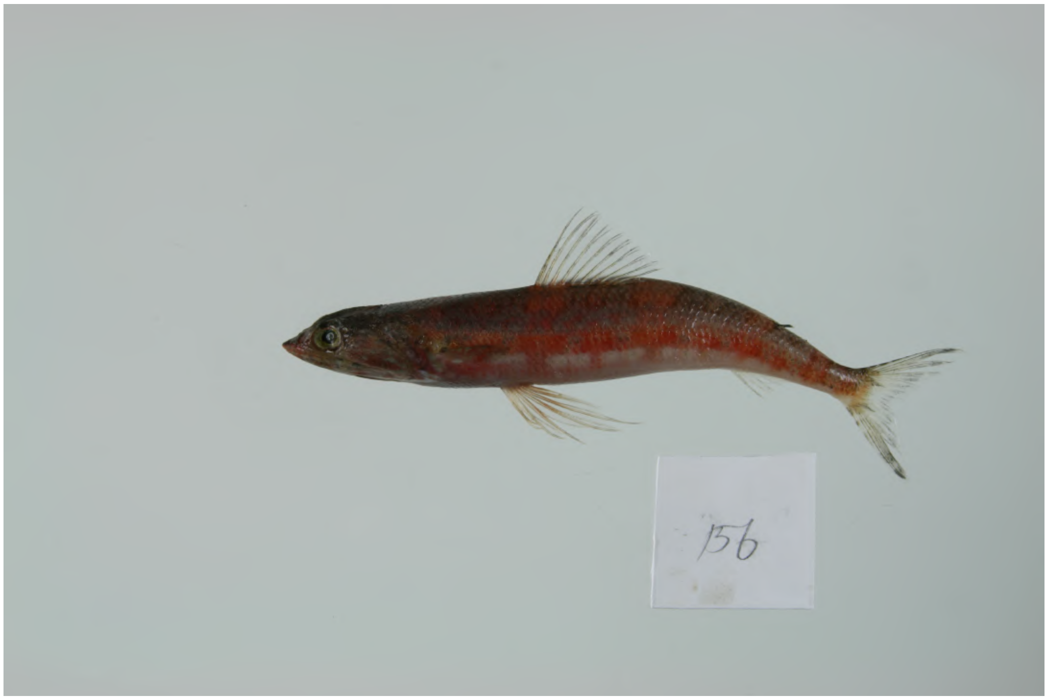

DOS 08627-1, *Synodus variegatus*, OR114194.

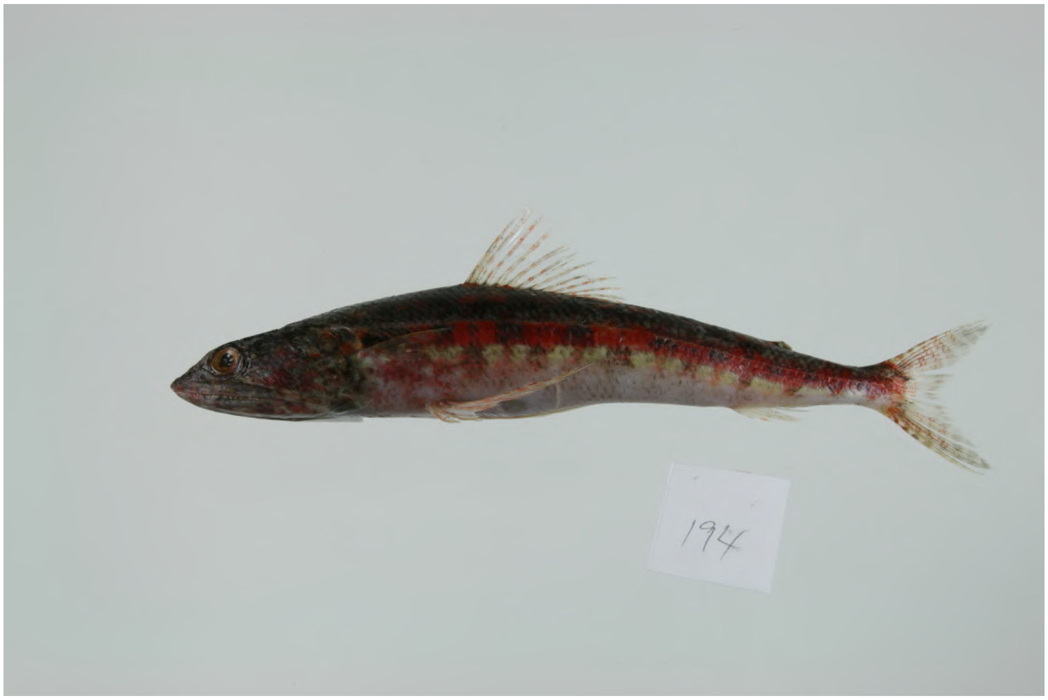

DOS 08627-2, *Synodus variegatus*, OR114195.

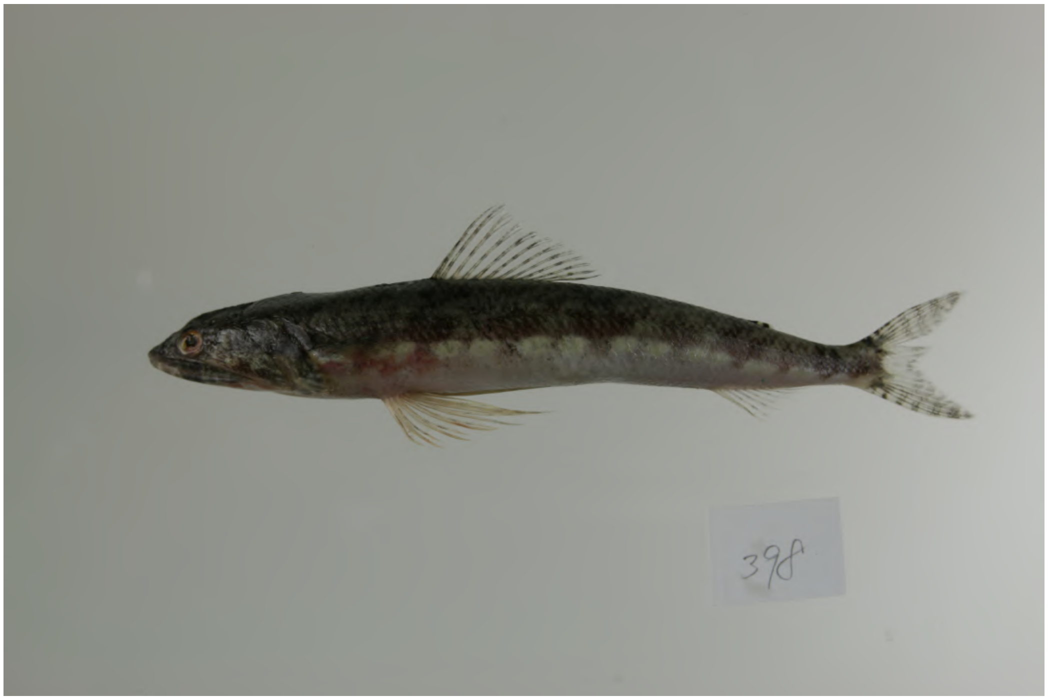

DOS 08627-3, *Synodus variegatus*, OR114196.

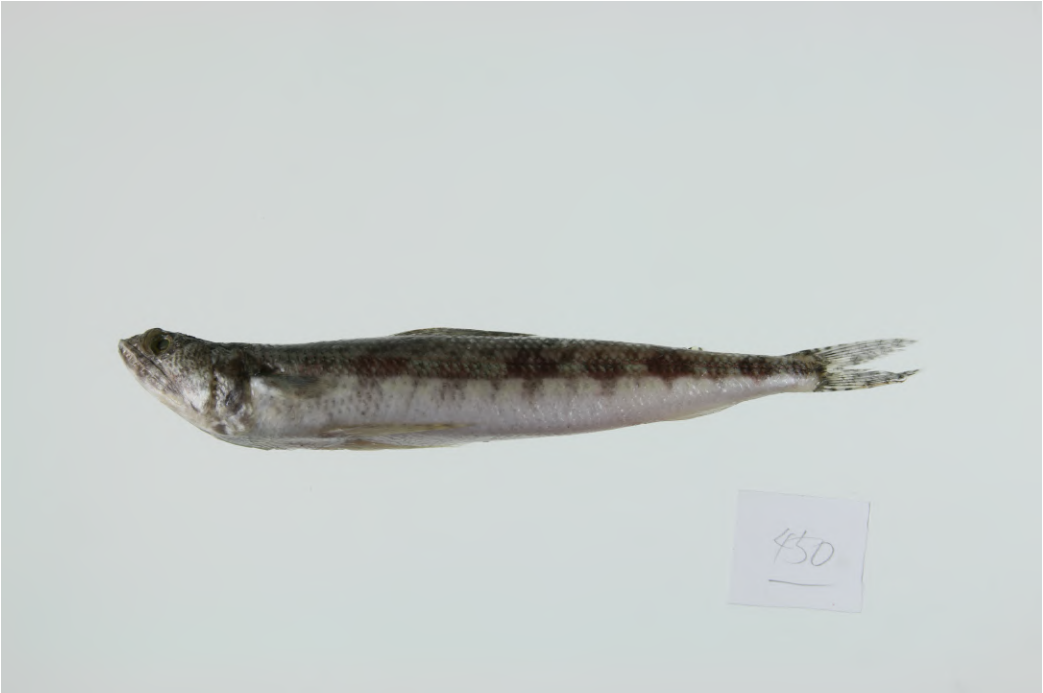

DOS 08627-4, *Synodus variegatus*, OR114197.

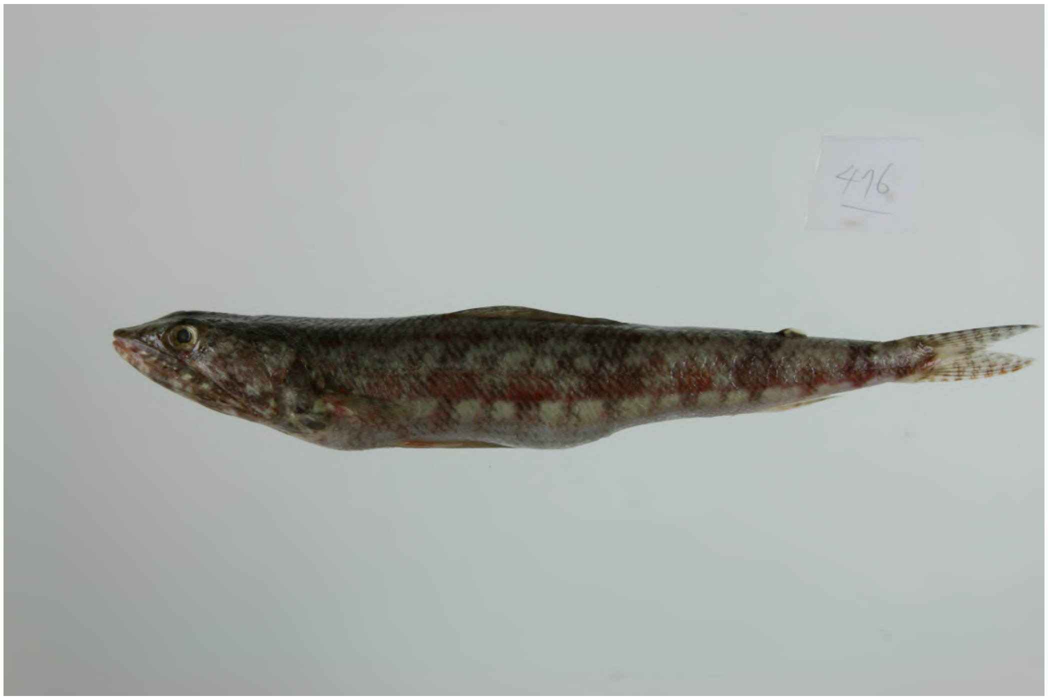

DOS 08627-5, *Synodus variegatus*, OR114198.

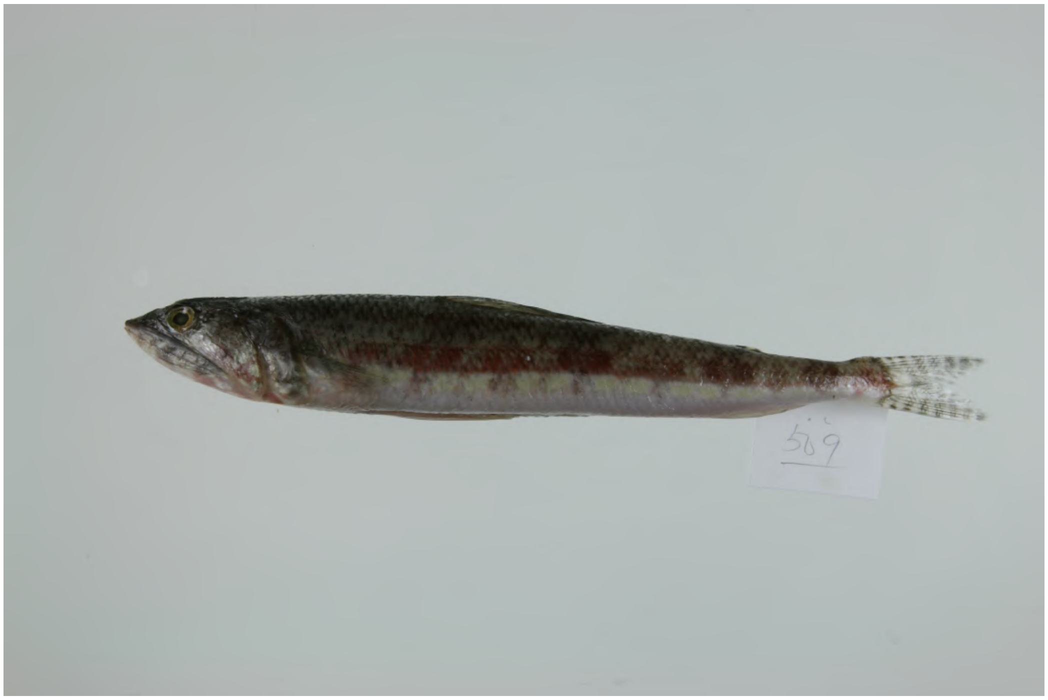

DOS 08627-6, *Synodus variegatus*, OR114199.

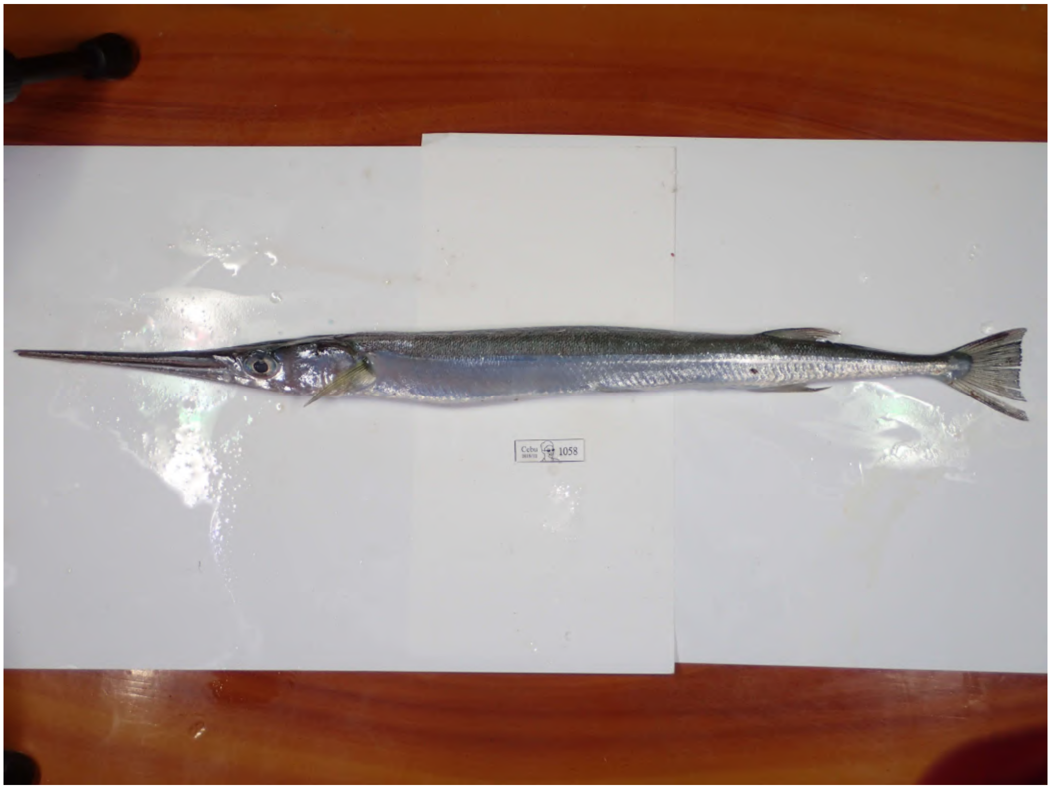

DOS 06618, *Strongylura incisa*, OR113809.

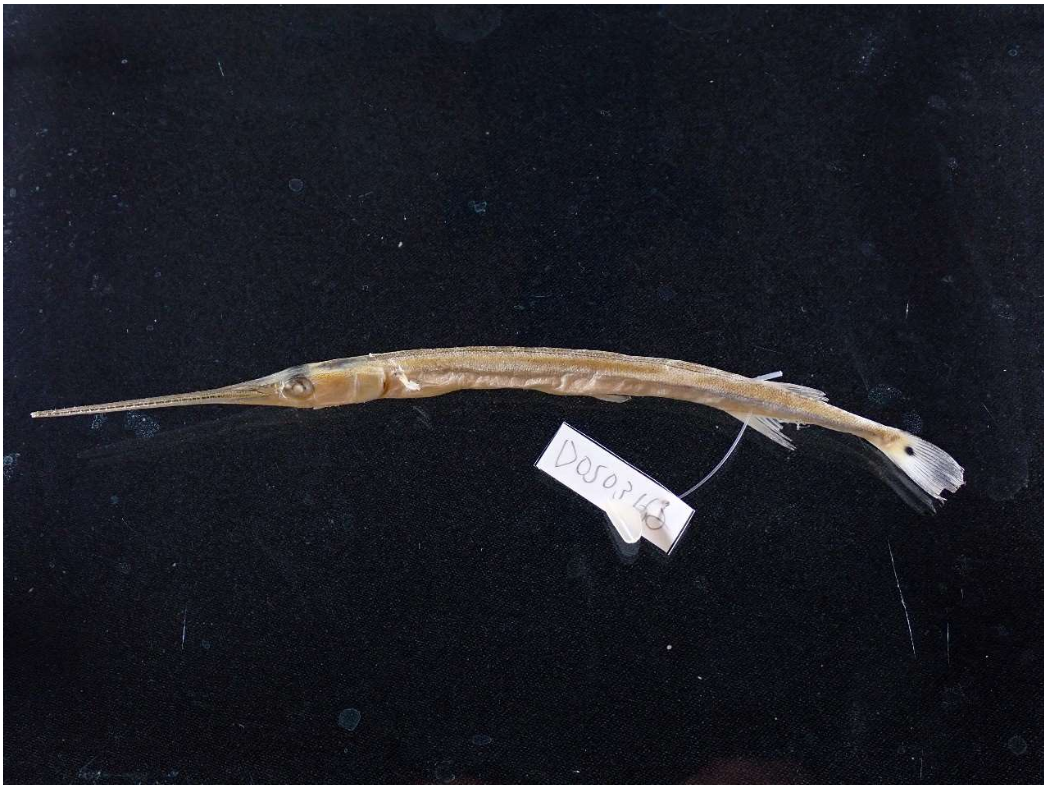

DOS 03162, *Strongylura strongylura*, OR113757.

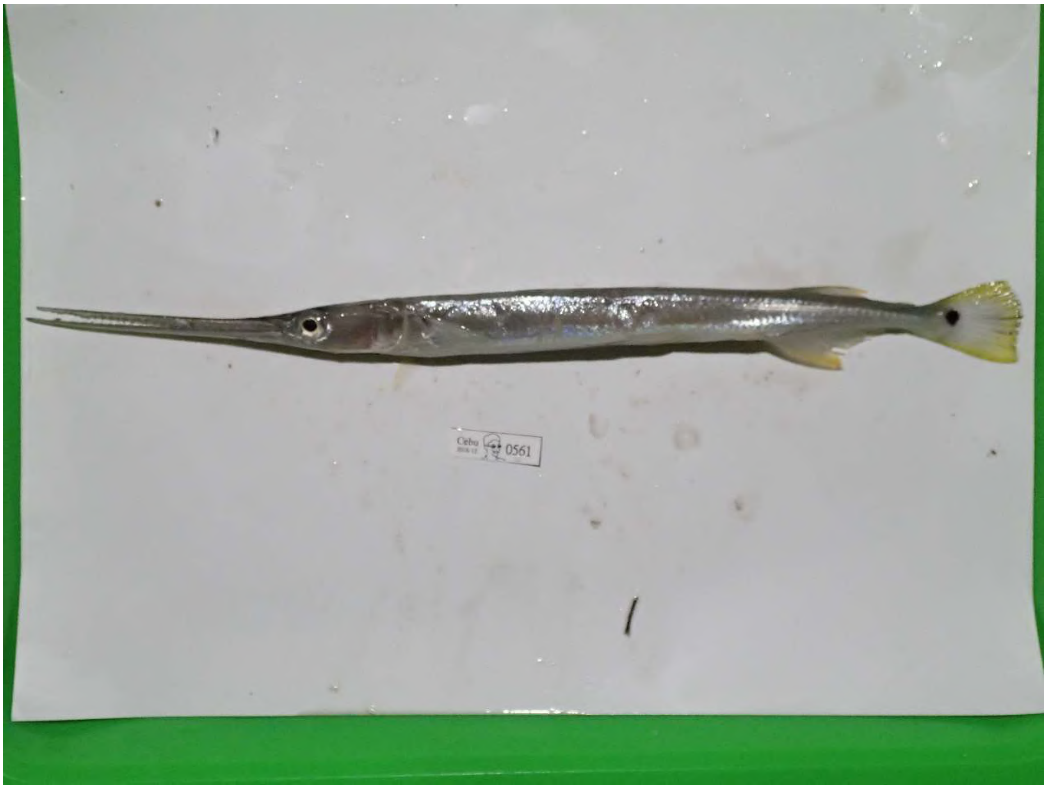

DOS 06617-7, *Strongylura strongylura*, OR113808.

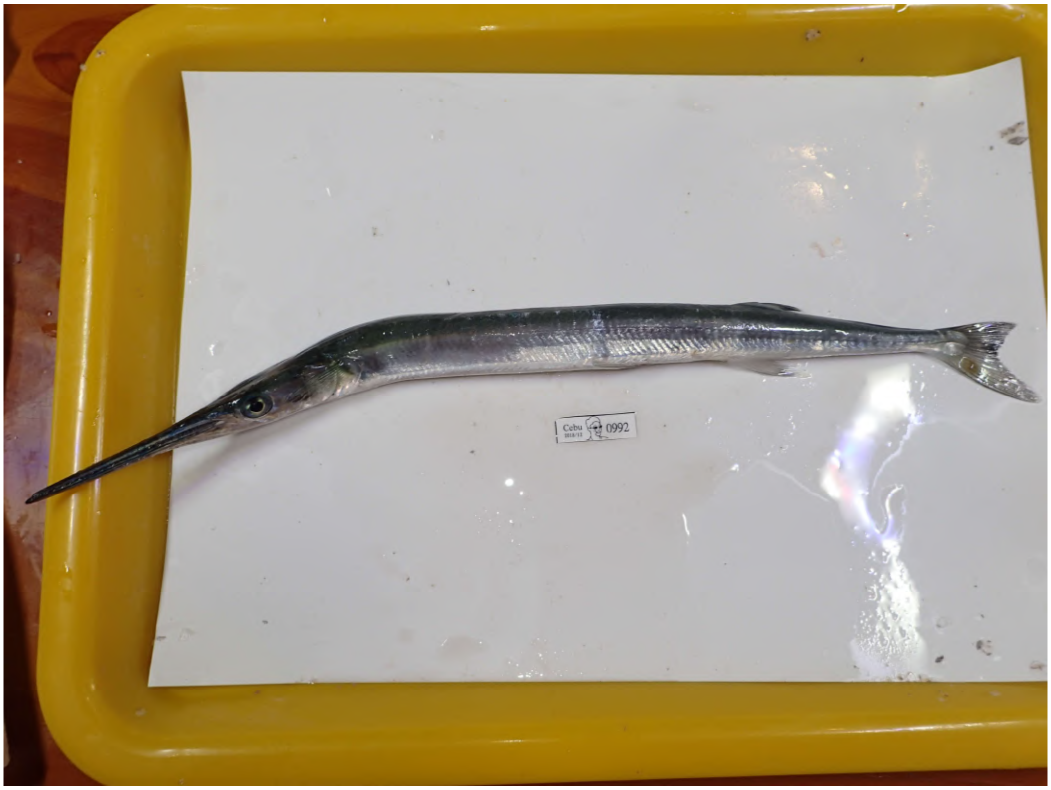

DOS 06616-1, *Tylosurus crocodilus*, OR113807. (specimen not preserved)

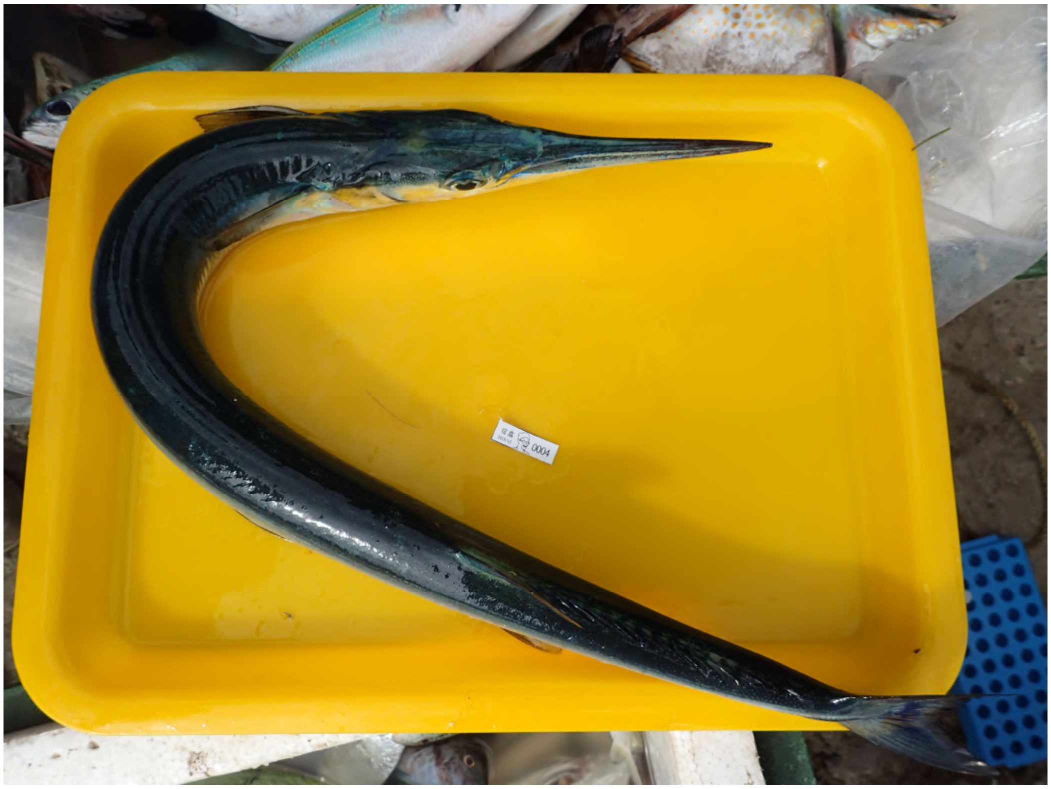

DOS 06619-1, *Tylosurus crocodilus*, OR113810. (specimen not preserved)

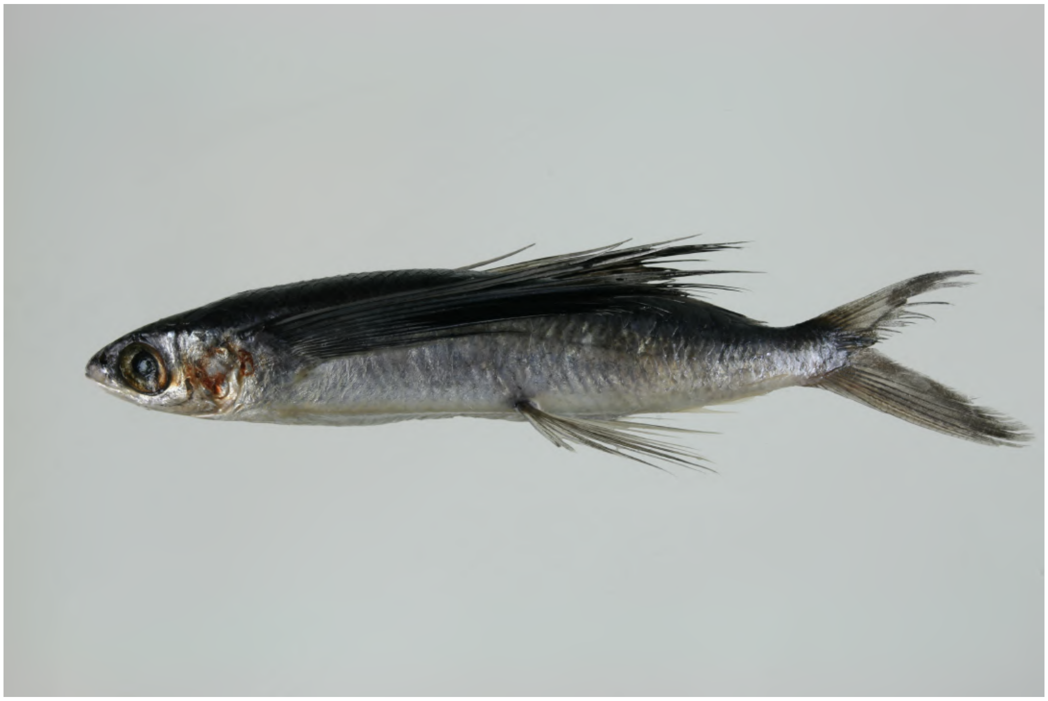

DOS 08628, *Cheilopogon spilonotopterus*, OR114200.

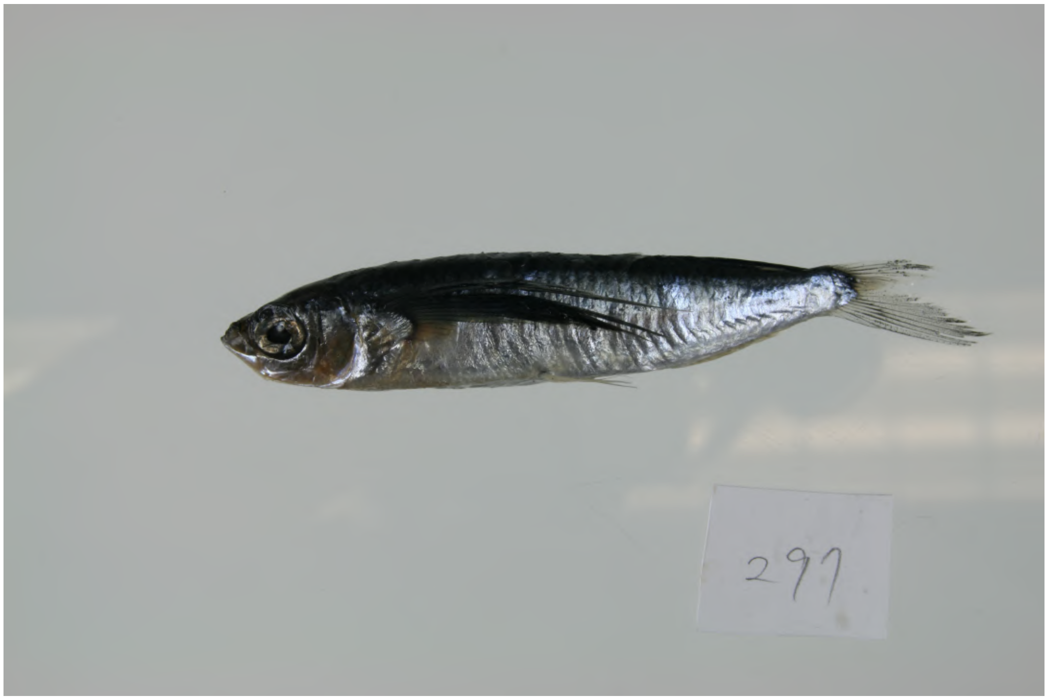

DOS 08629, *Parexocoetus mento*, OR114201.

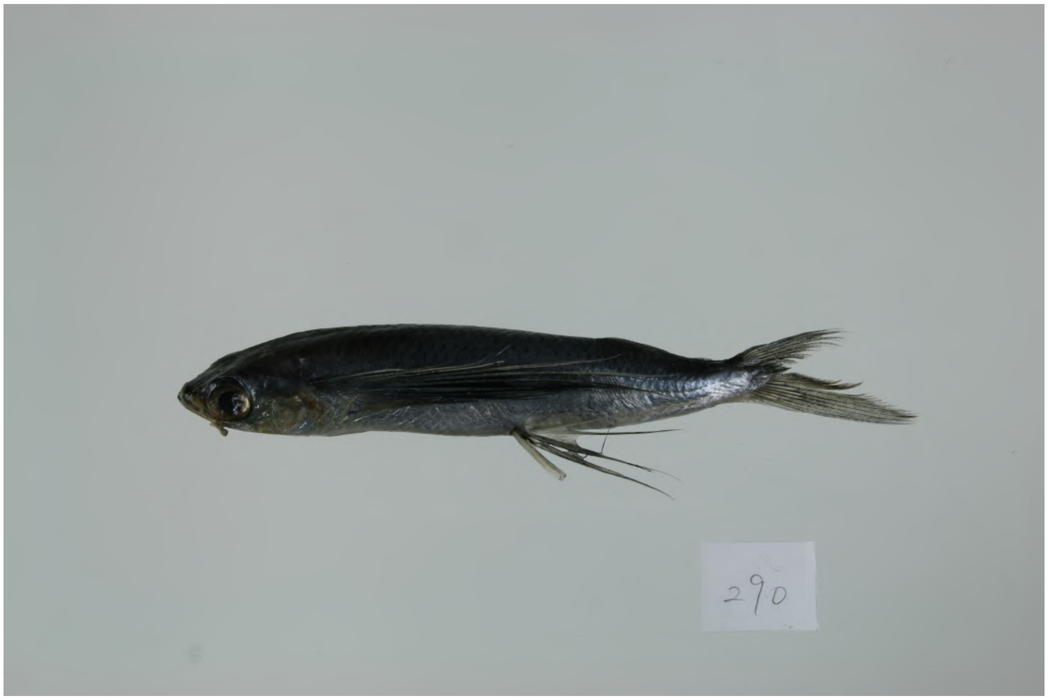

DOS 08630, *Prognichthys brevipinnis*, OR114202.

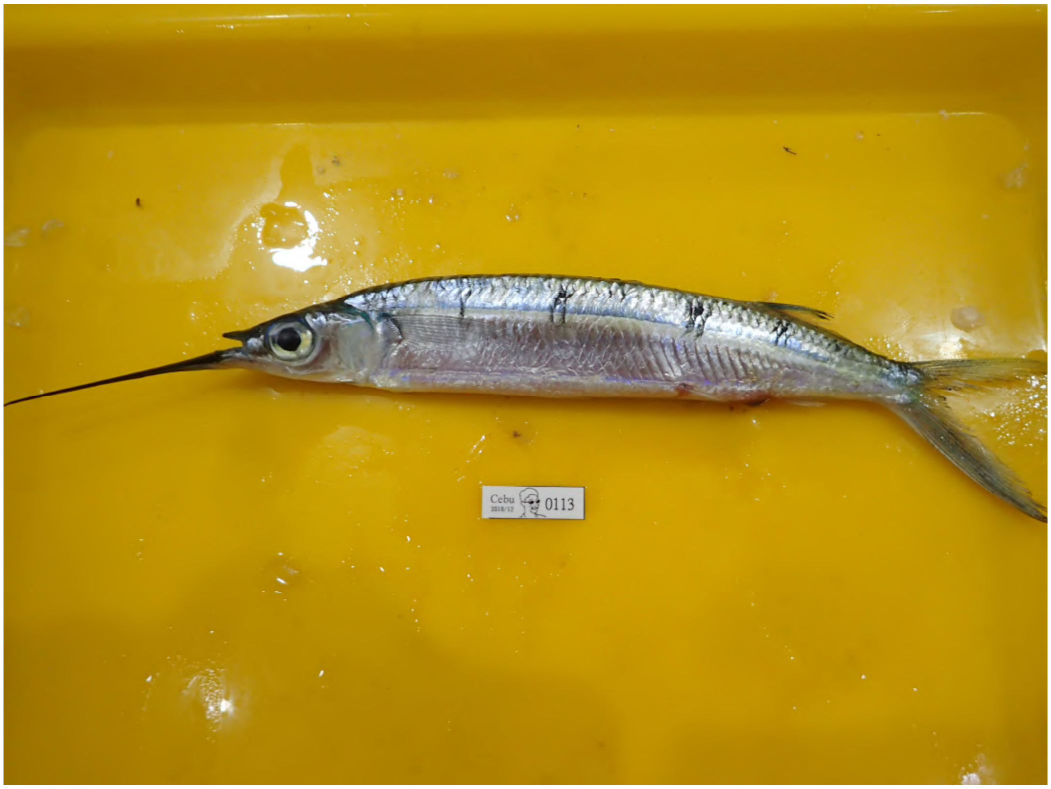

DOS 06722-1, *Hemiramphus far*, OR113910.

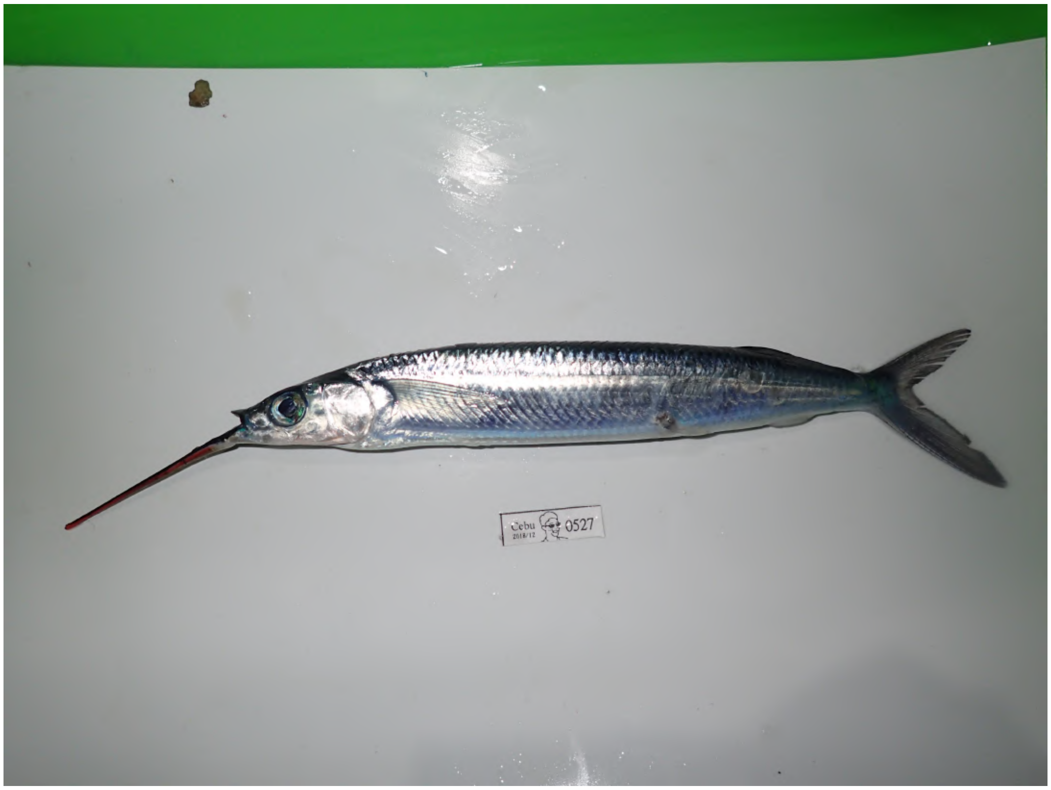

DOS 06721-1, *Hemiramphus lutkei*, OR113909.

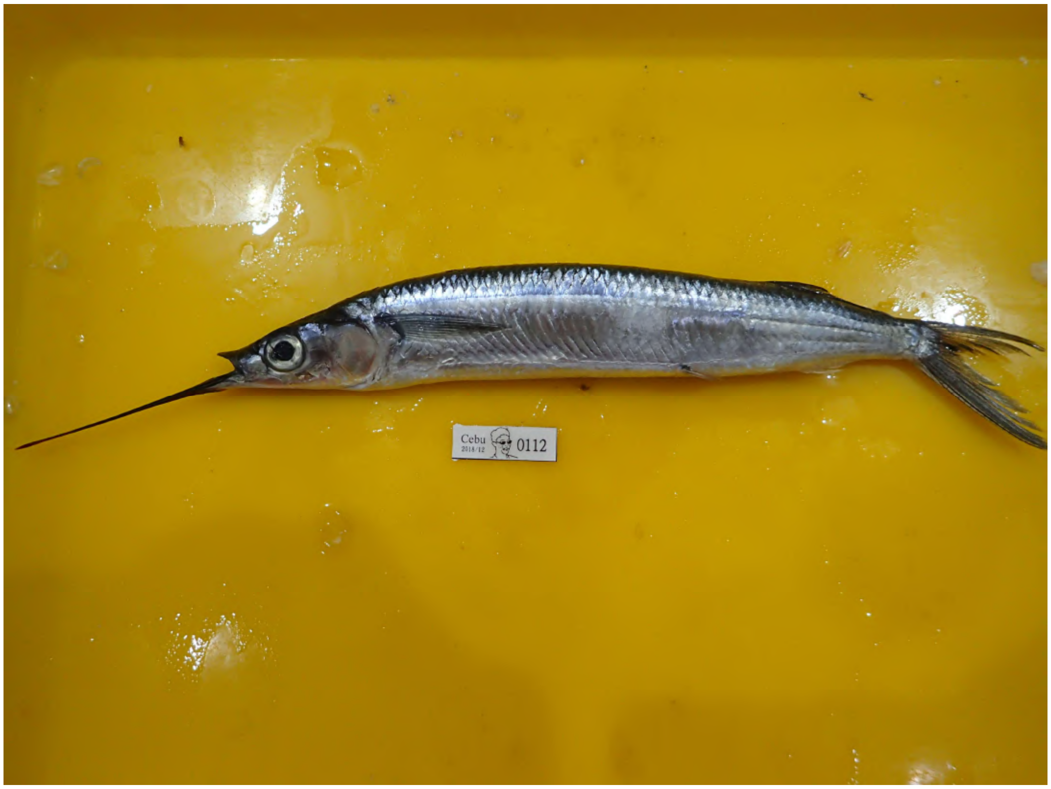

DOS 06723-1, *Hemiramphus lutkei*, OR113911.

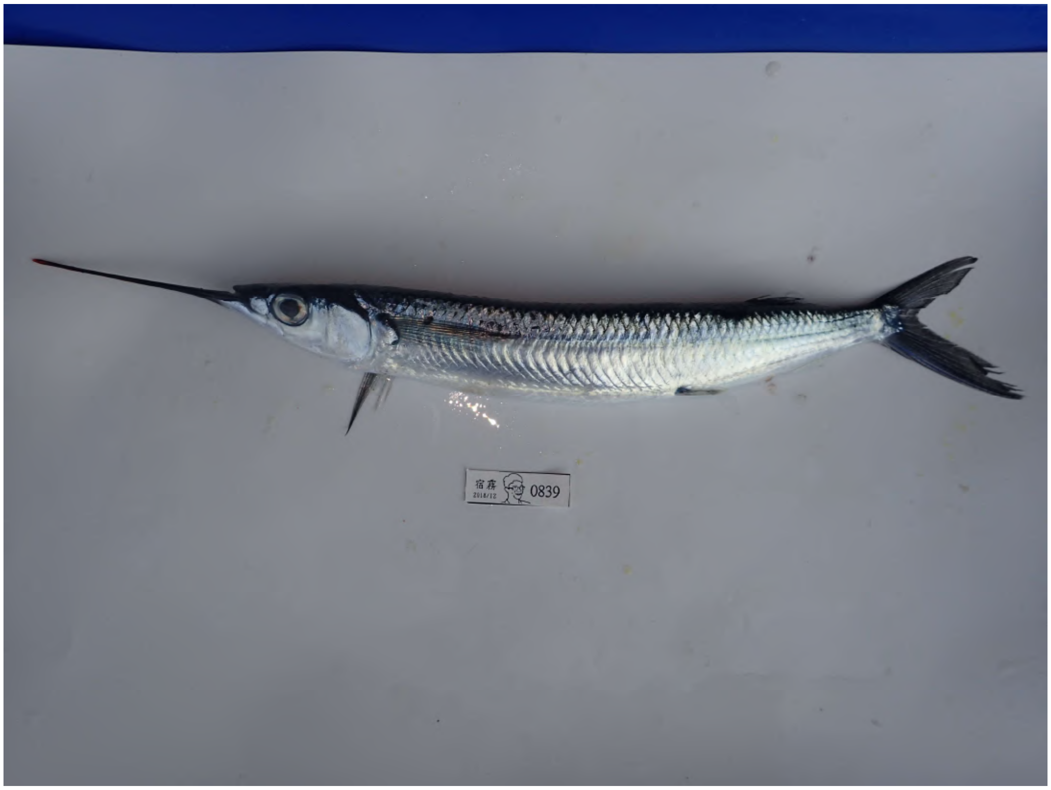

DOS 06726-1, *Hemiramphus lutkei*, OR113914. (specimen not preserved)

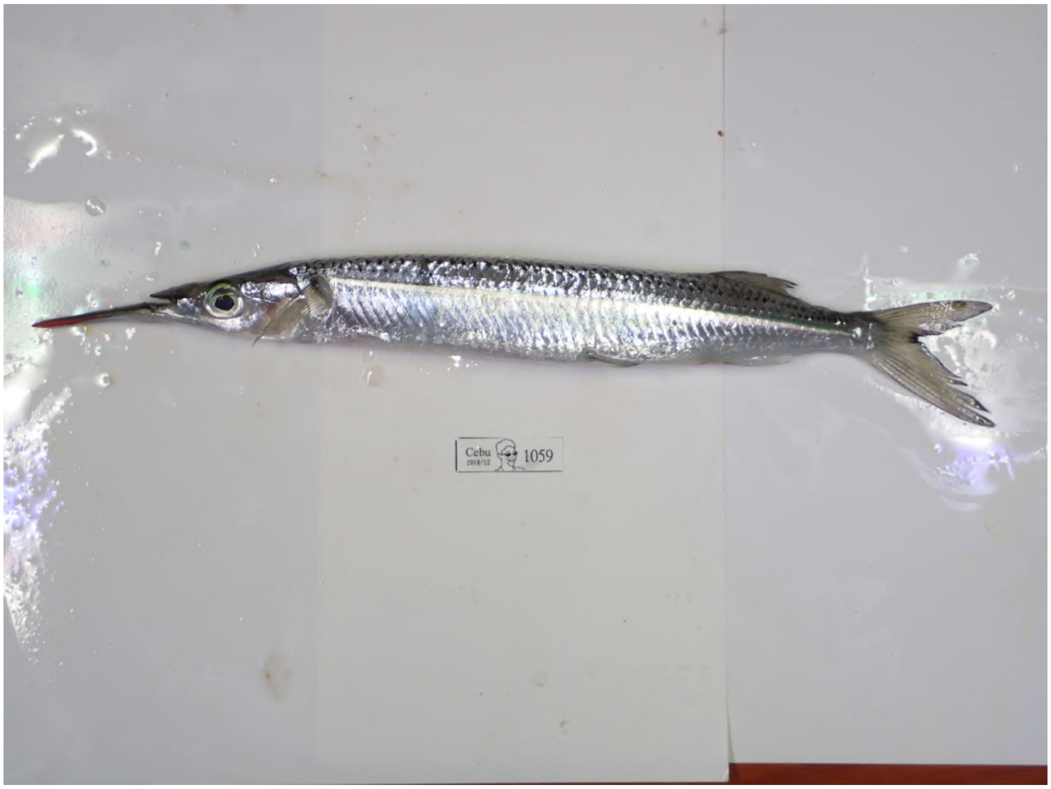

DOS 06725-1, *Hyporhamphus quoyi*, OR113913. (specimen not preserved)

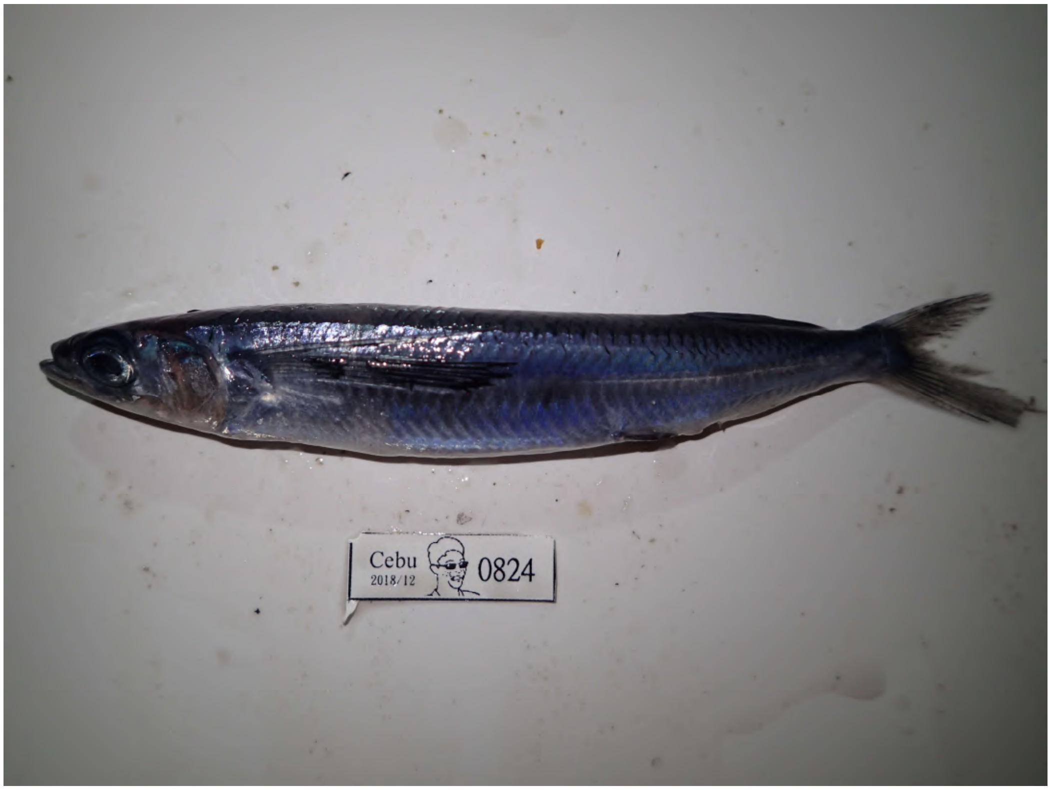

DOS 06695-1, *Oxyporhamphus convexus*, OR113884.

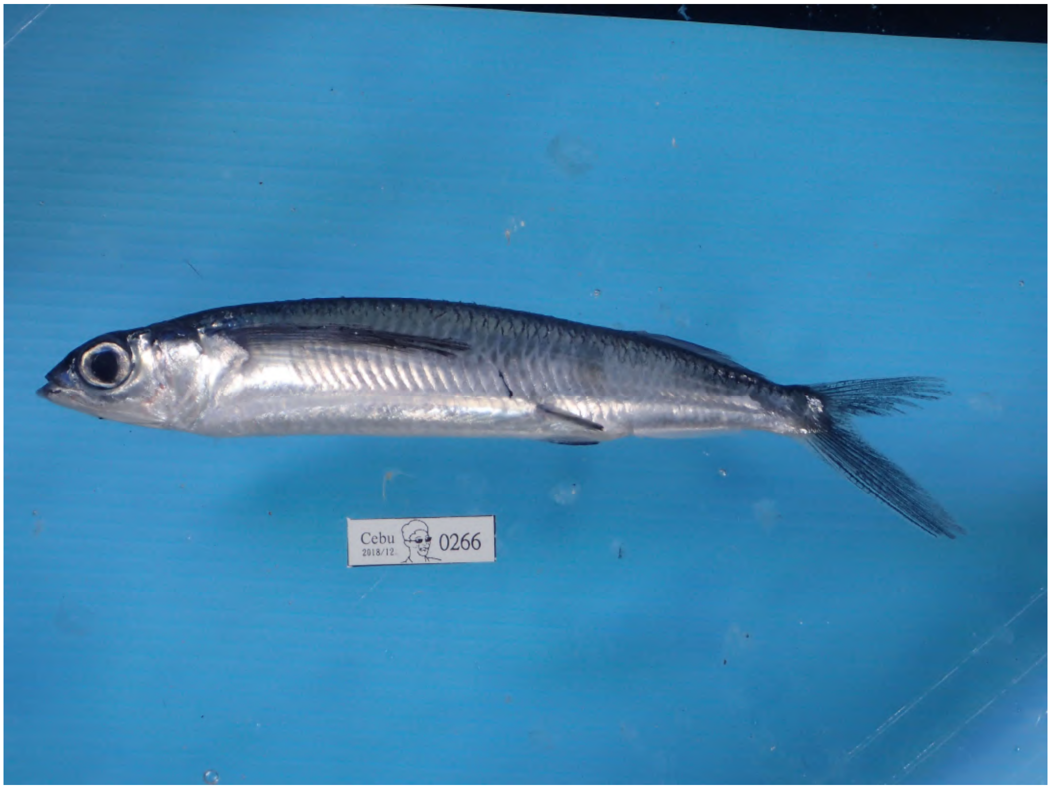

DOS 06697-1, *Oxyporhamphus convexus*, OR113885.

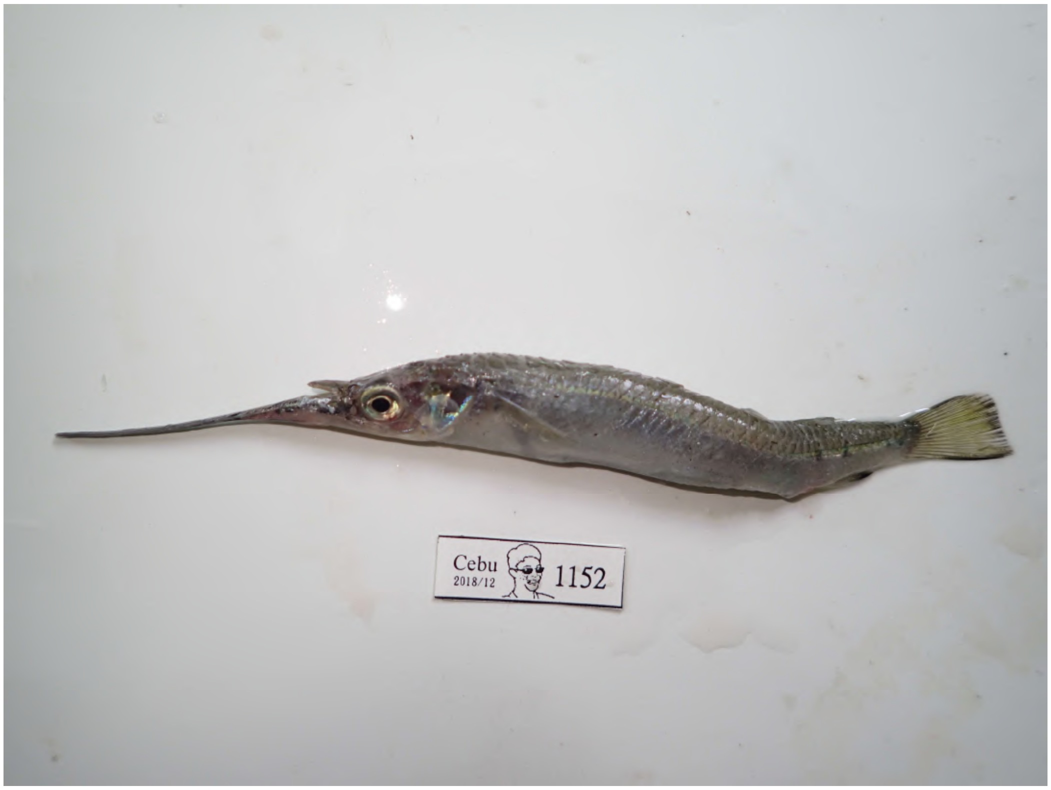

DOS 06724, *Zenarchopterus dispar*, OR113912.

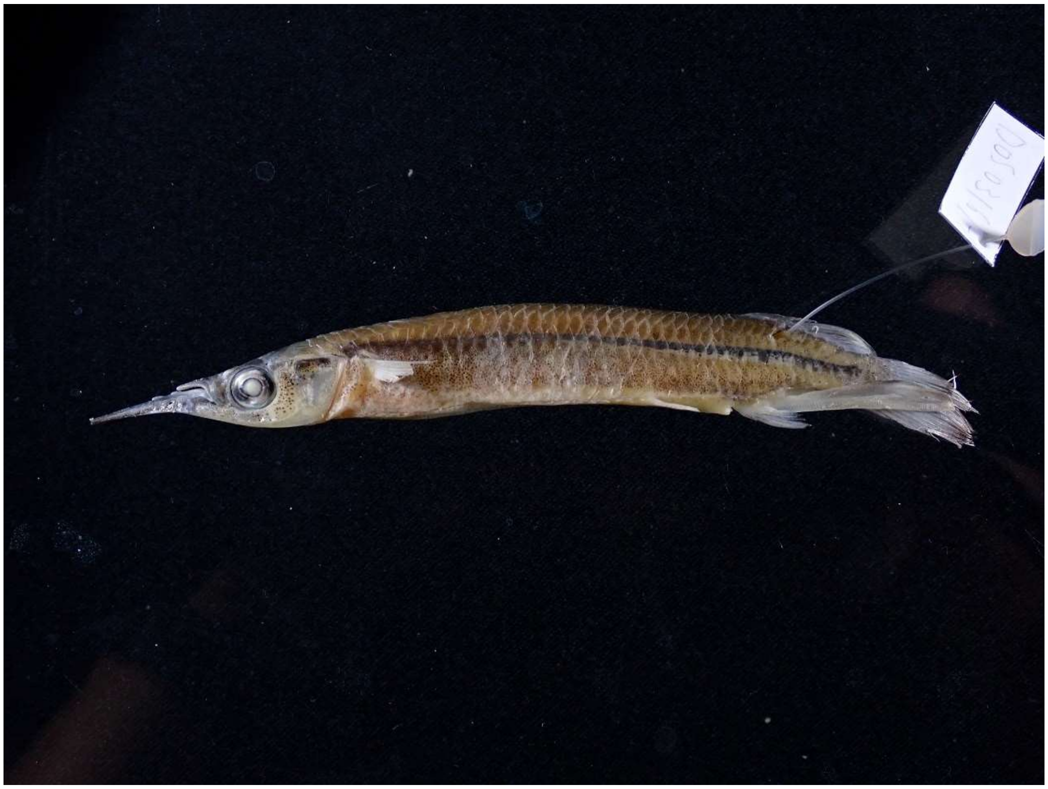

DOS 03161-1, *Zenarchopterus* sp., OR113756.

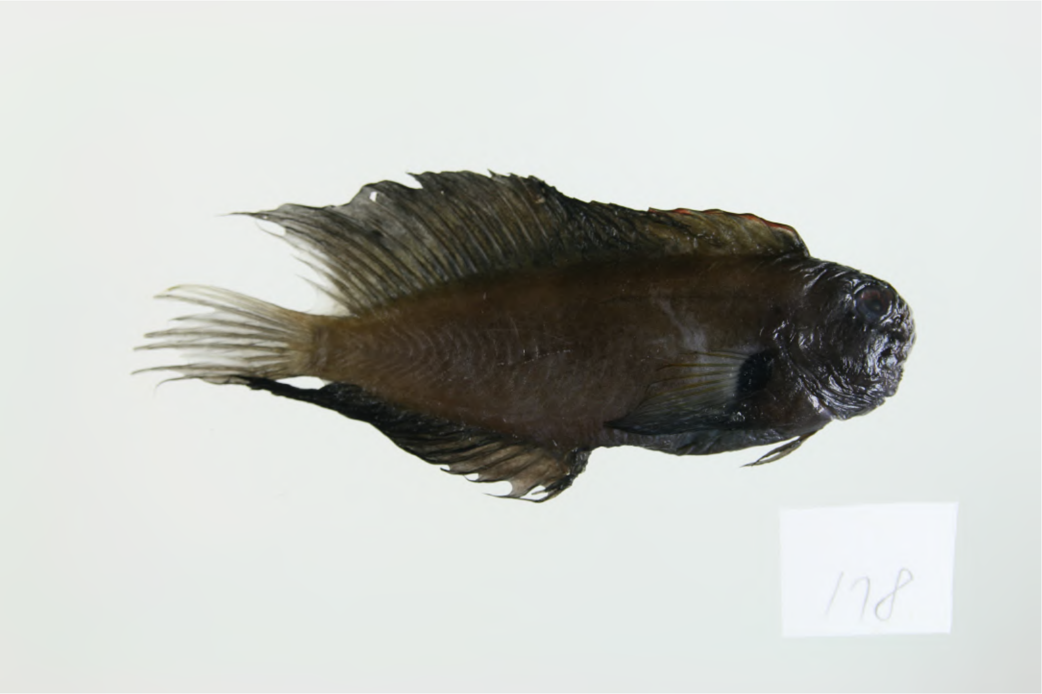

DOS 08631, *Atrosalarias holomelas*, OR114203.

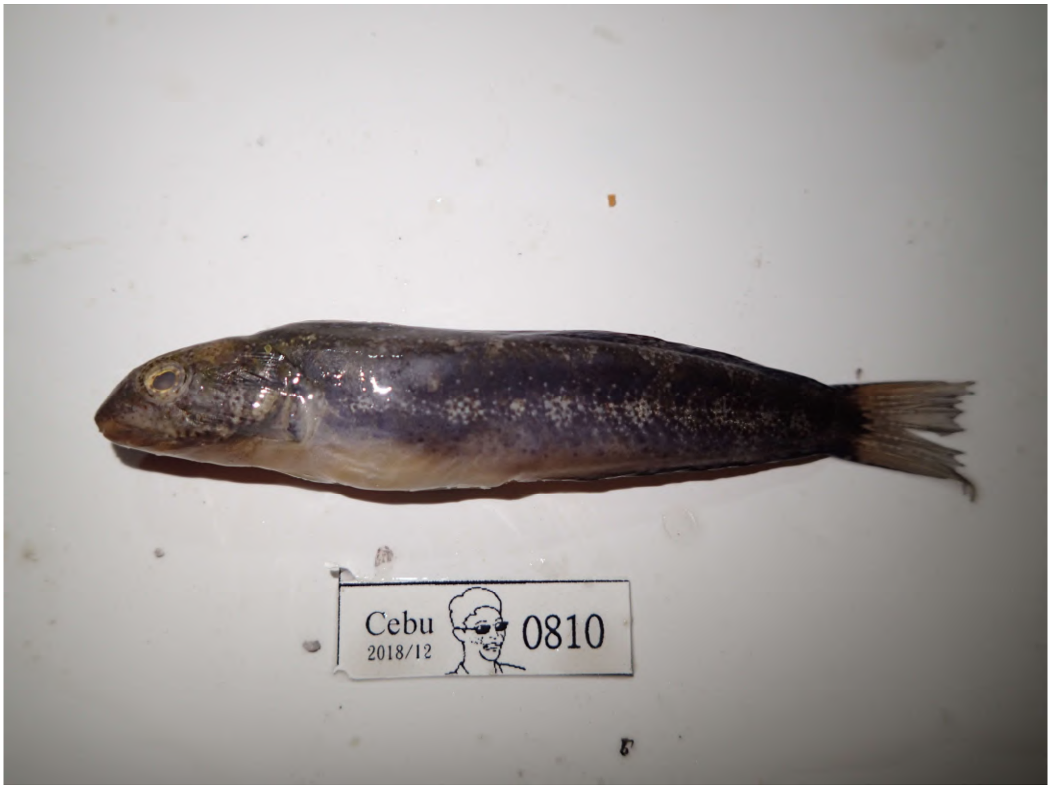

DOS 06621-2, *Petroscirtes variabilis*, OR113812.

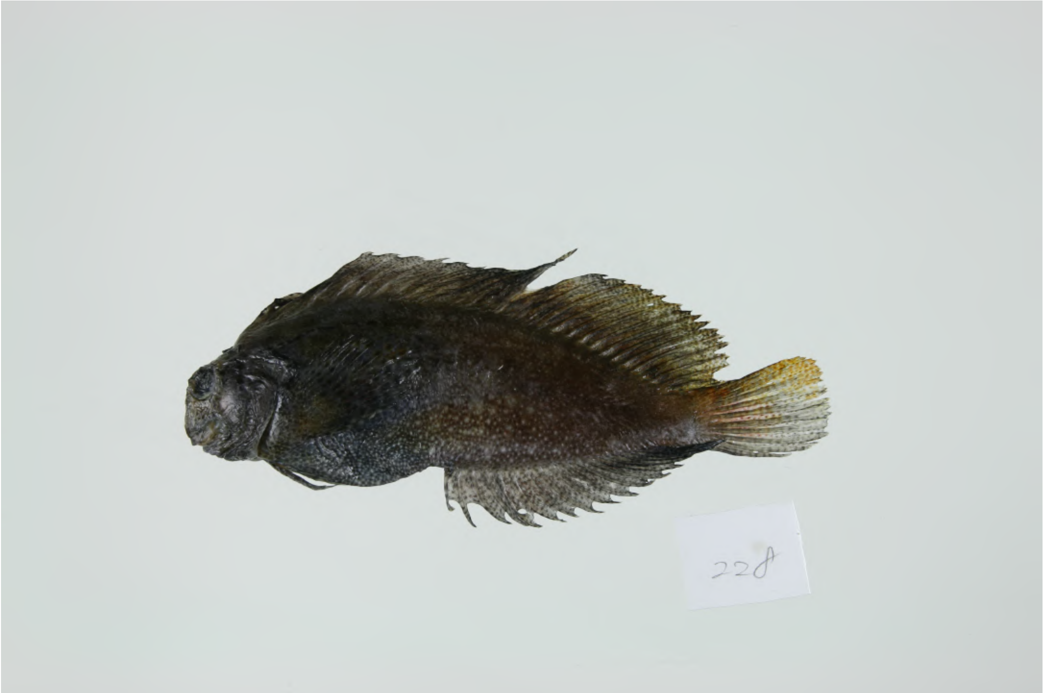

DOS 08632, *Salarias ceramensis*, OR114204.

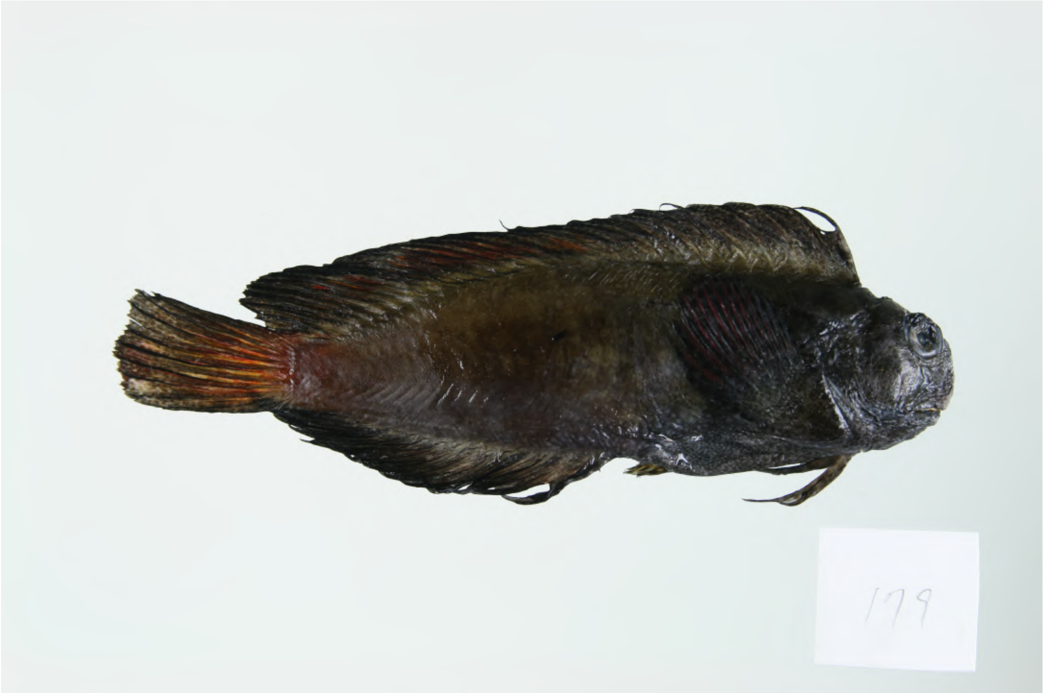

DOS 08633, *Salarias ceramensis*, OR114205.

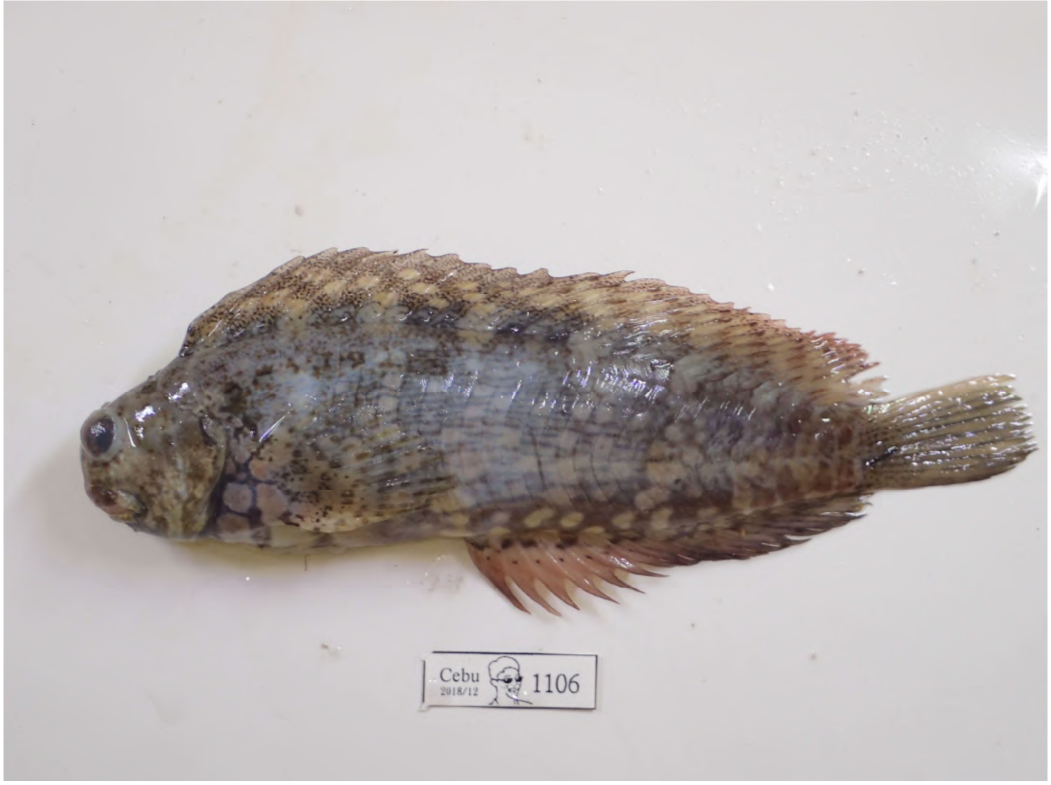

DOS 06620-1, *Salarias fasciatus*, OR113811.

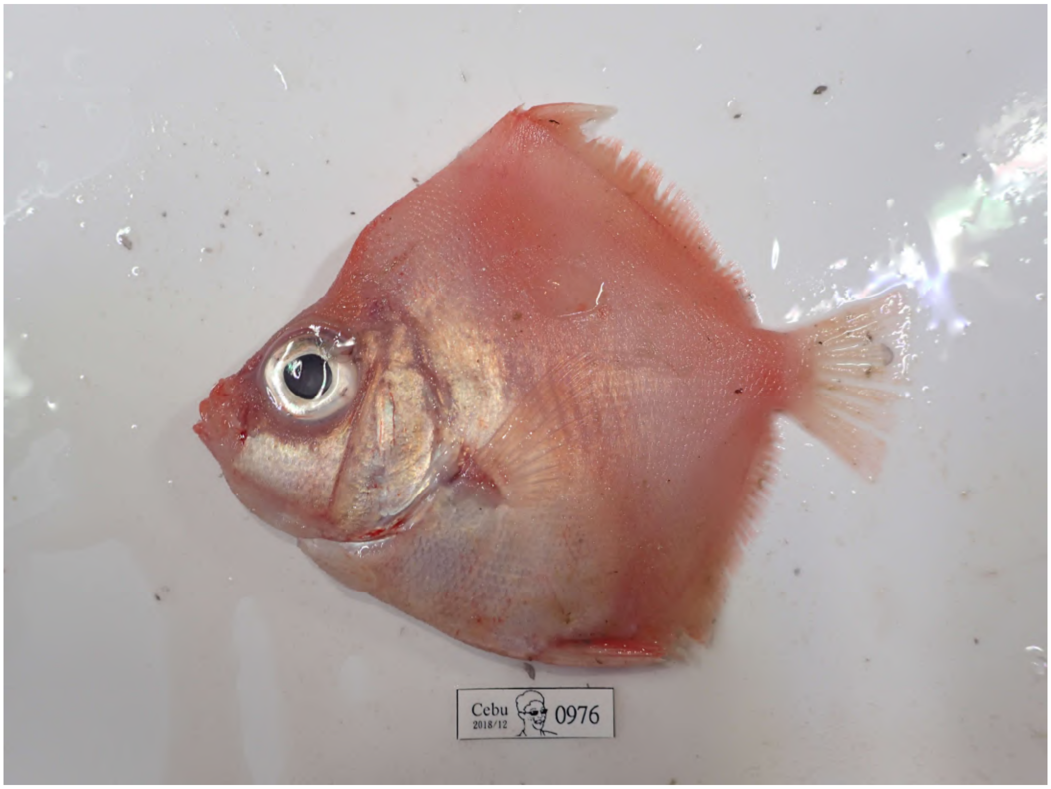

DOS 06632, *Antigonia rubescens*, OR113824.

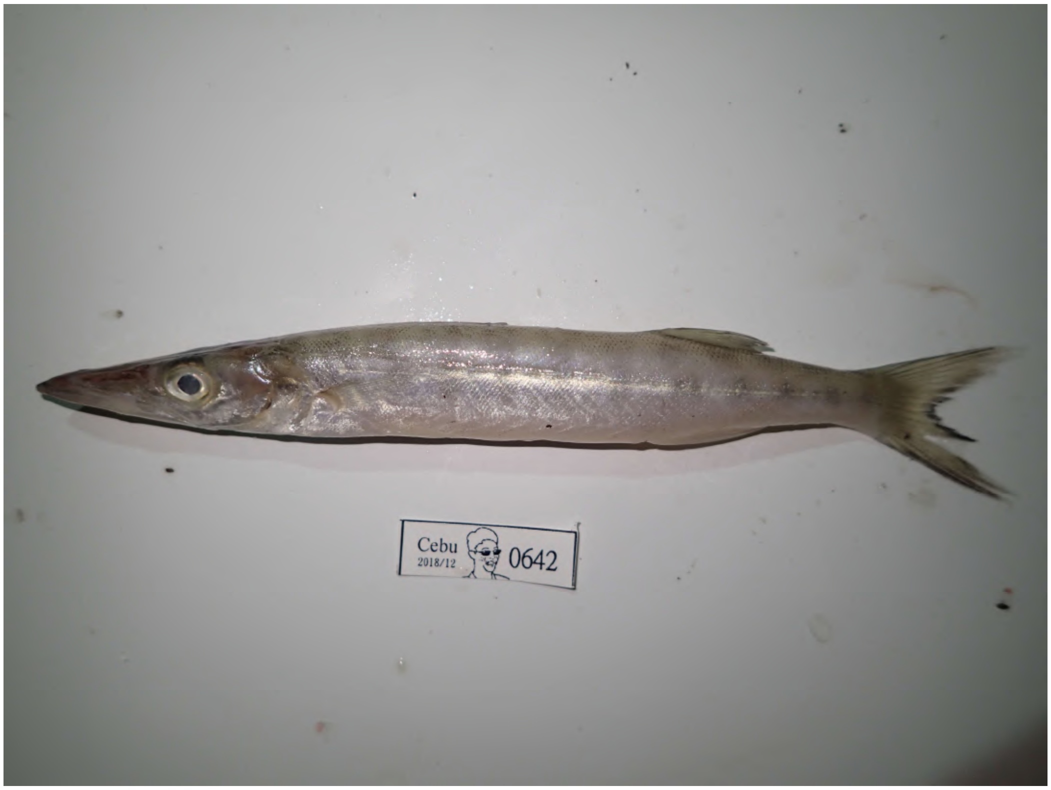

DOS 06971, *Sphyraena* cf. *jello*, OR114144.

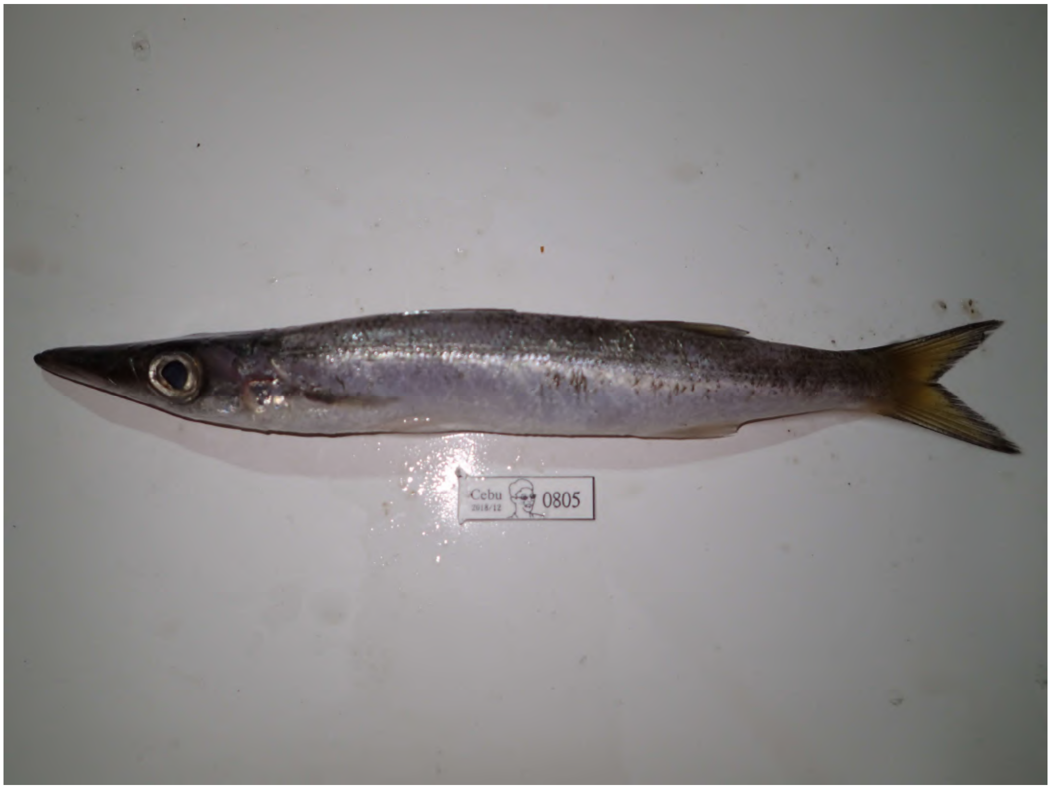

DOS 06967-1, *Sphyraena flavicauda*, OR114141.

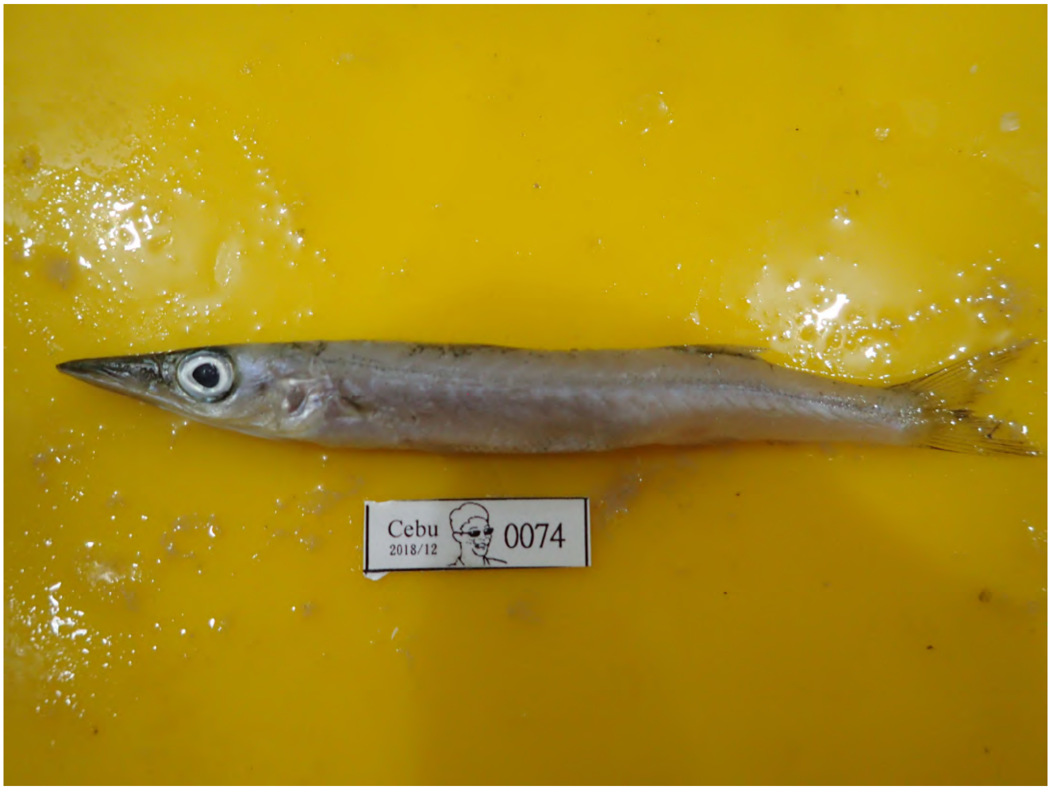

DOS 06970, *Sphyraena flavicauda*, OR114143.

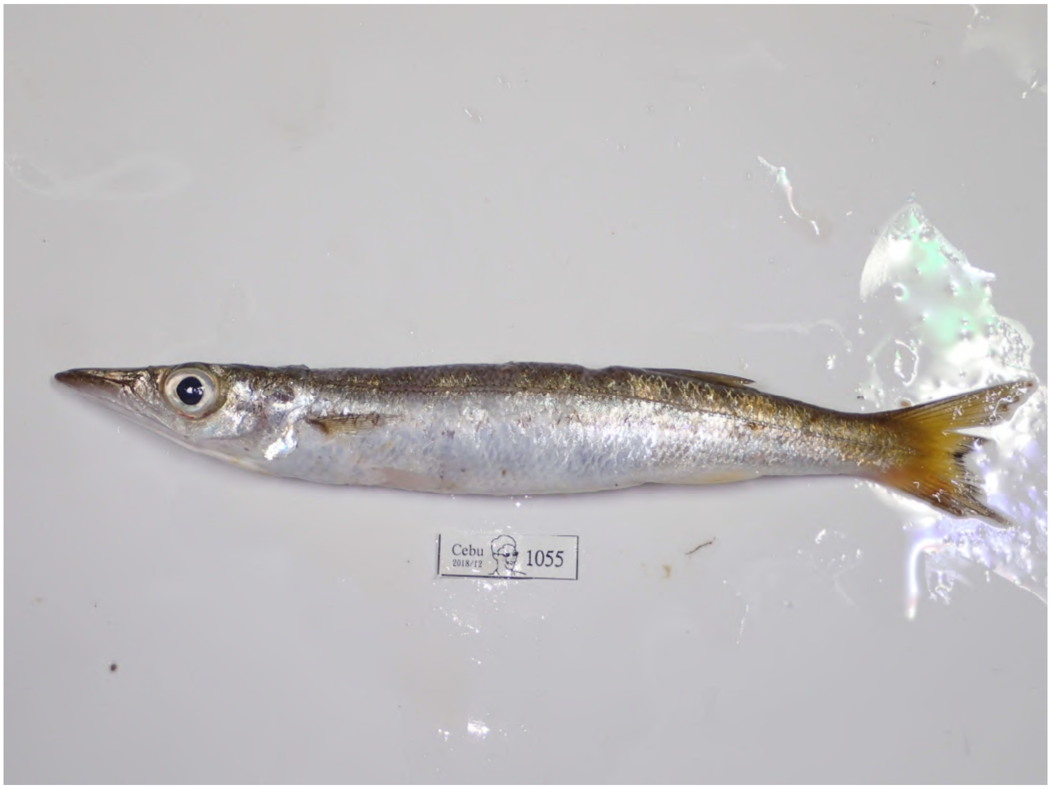

DOS 06969, *Sphyraena* sp., OR114142.

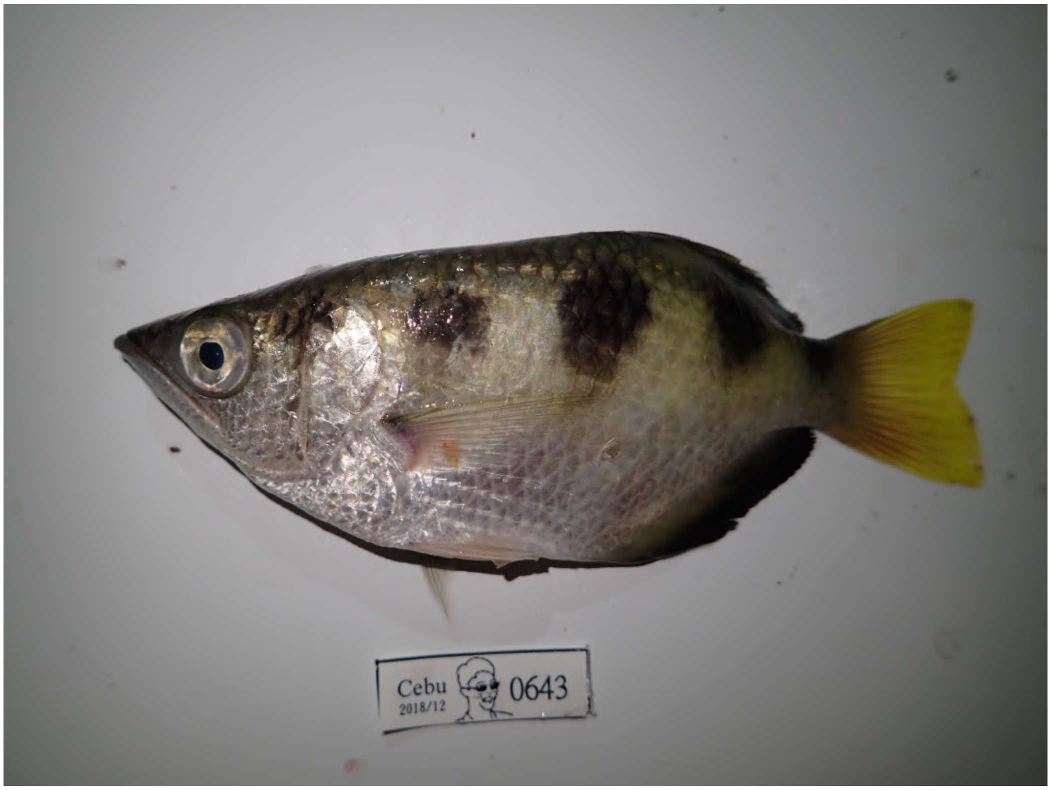

DOS 06990, *Toxotes jaculatrix*, OR114159.

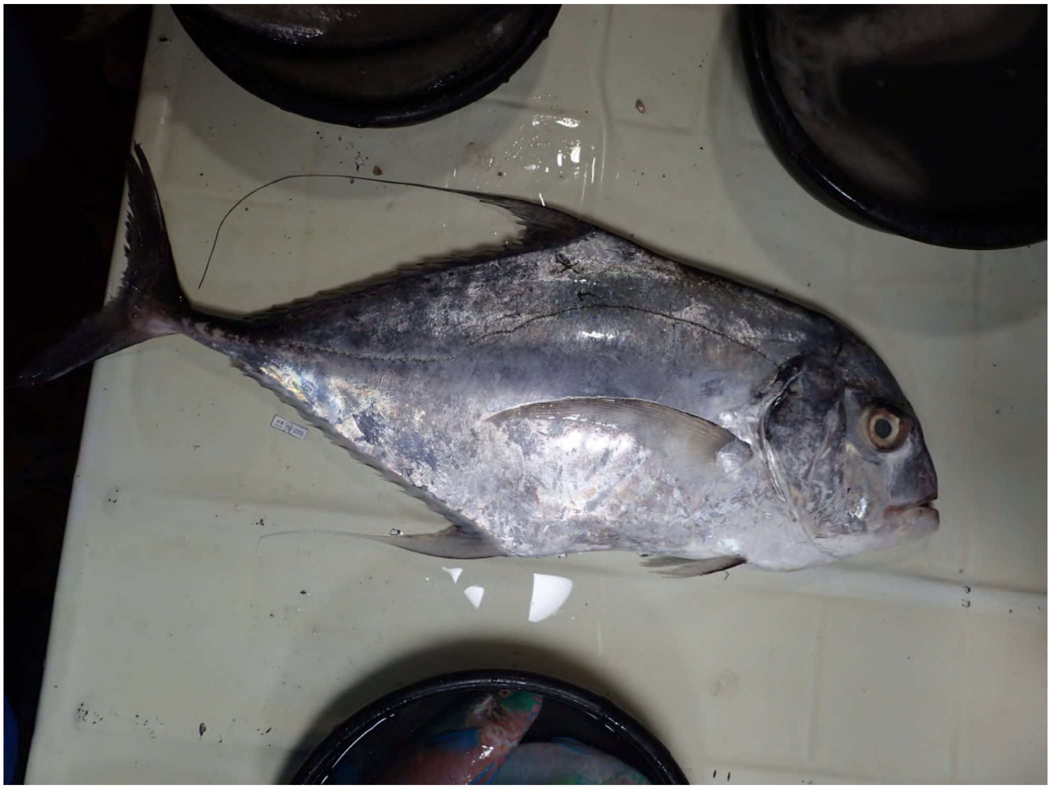

DOS 06633, *Alectis ciliaris*, OR113825. (specimen not preserved)

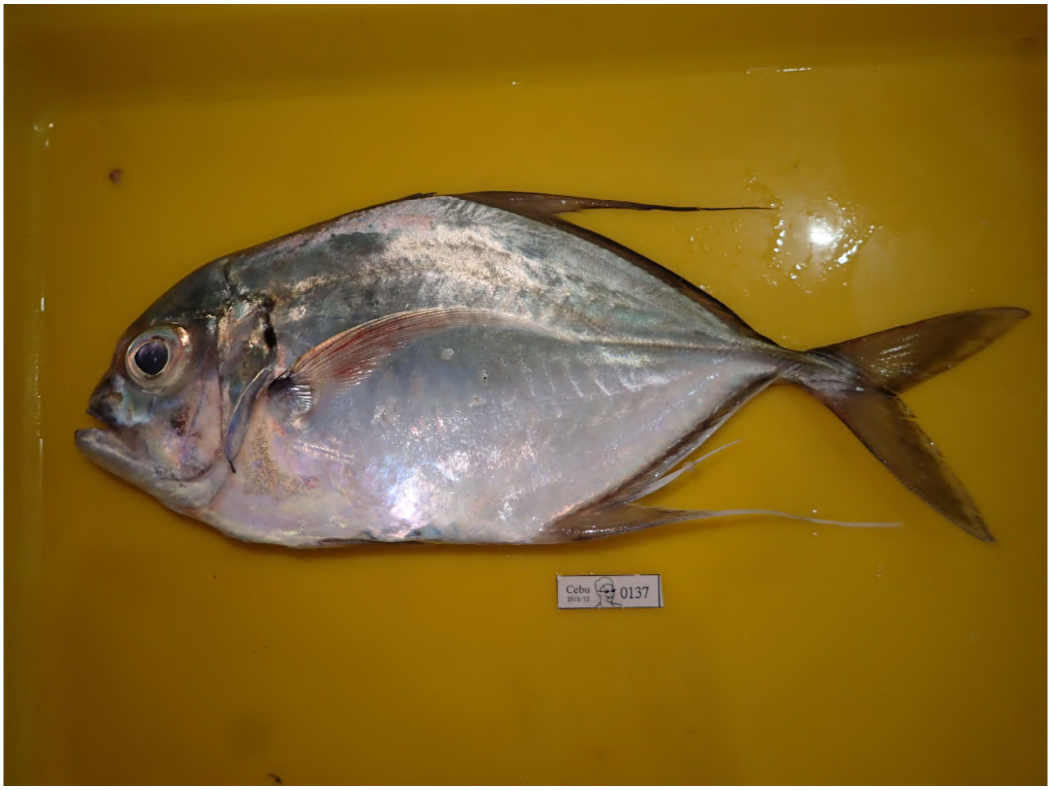

DOS 06653, *Atropus hedlandensis*, OR113844.

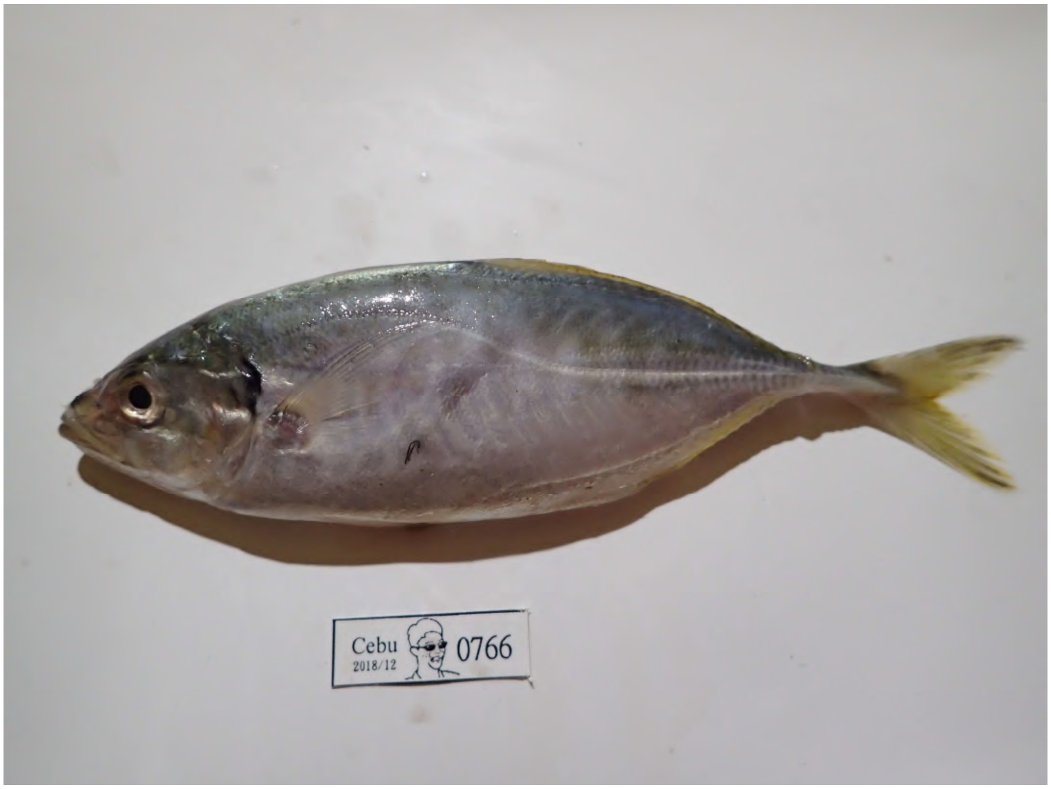

DOS 06634-1, *Atule mate*, OR113826. (specimen not preserved)

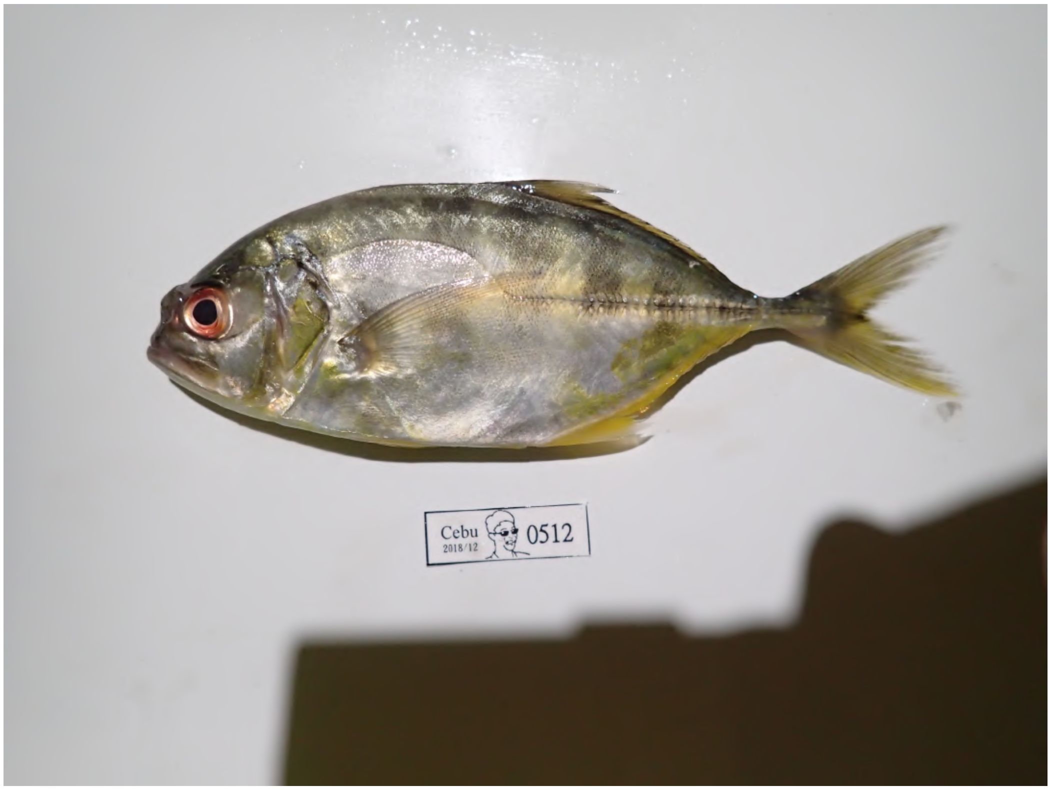

DOS 06639, *Caranx sexfasciatus*, OR113831.

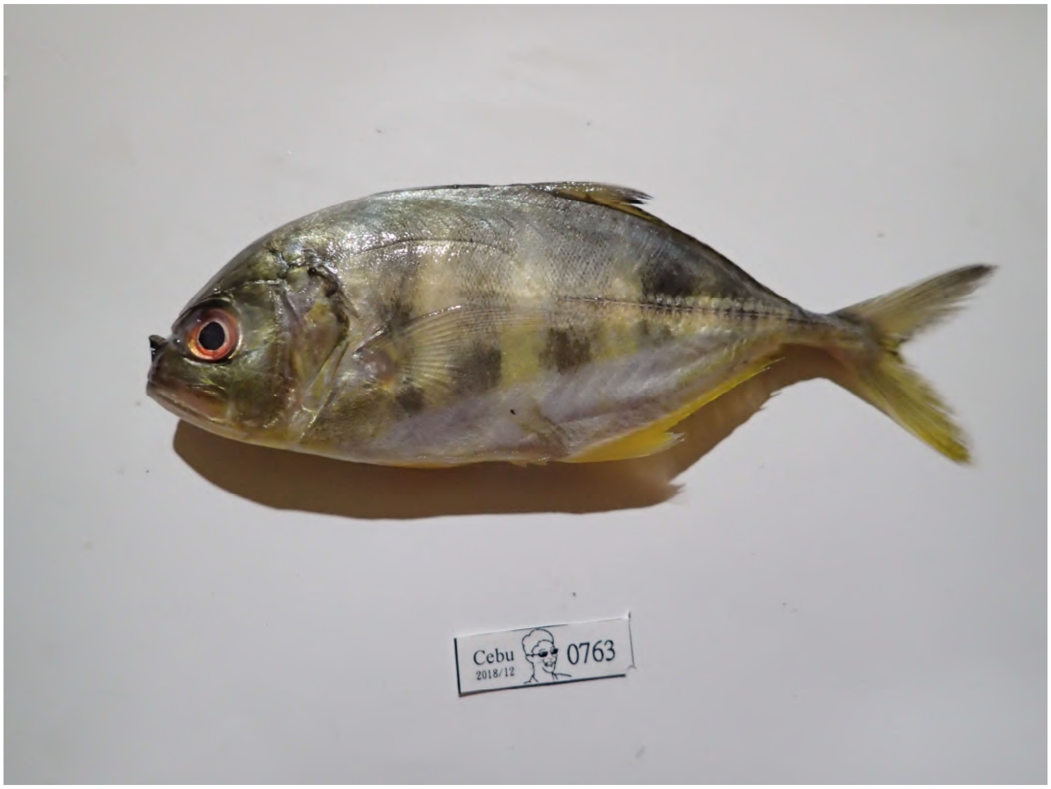

DOS 06640-1, *Caranx sexfasciatus*, OR113832.

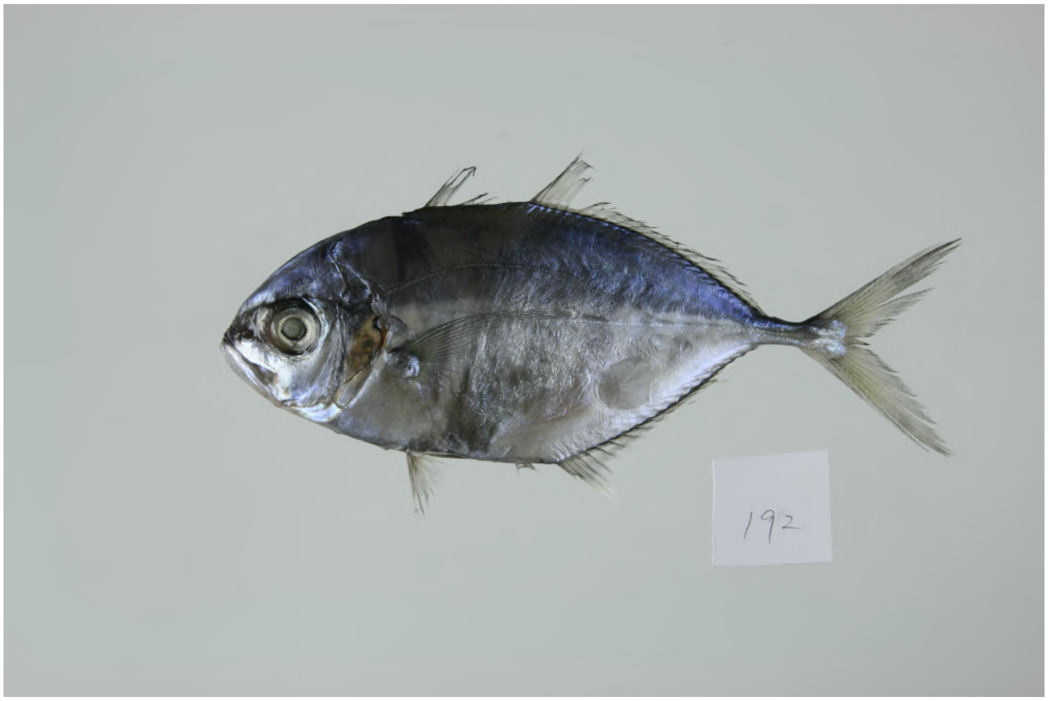

DOS 08635, *Craterognathus plagiotaenia*, OR114207.

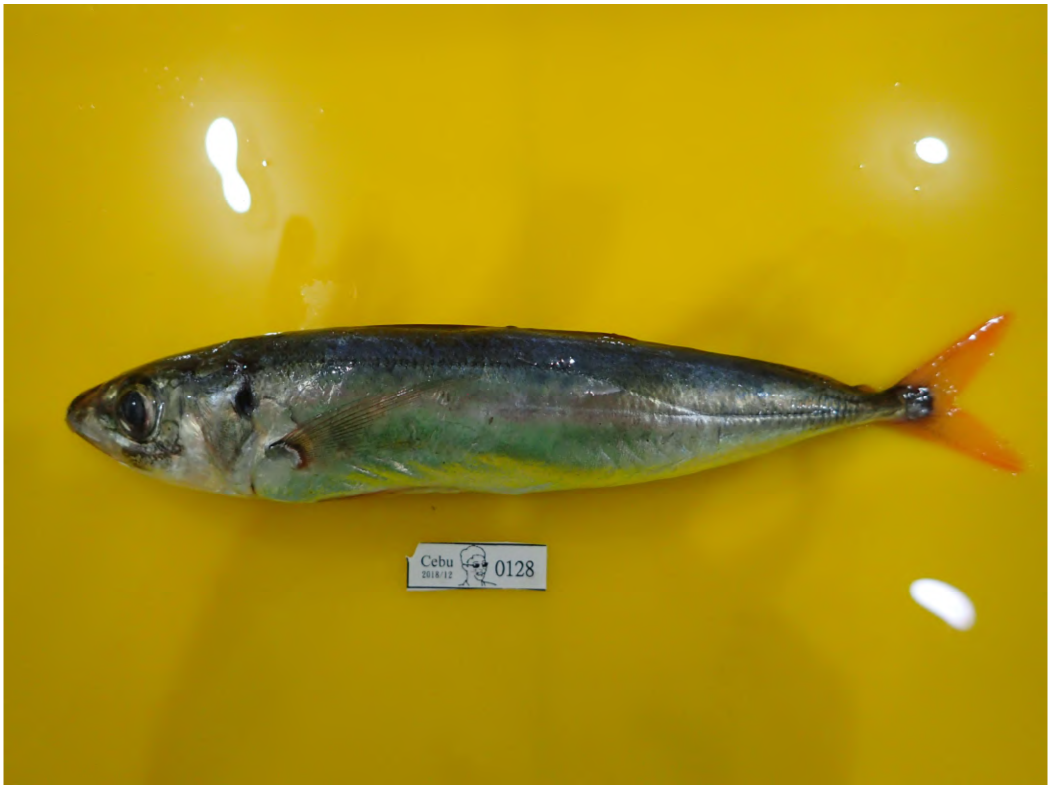

DOS 06641-1, *Decapterus kurroides*, OR113833. (specimen not preserved)

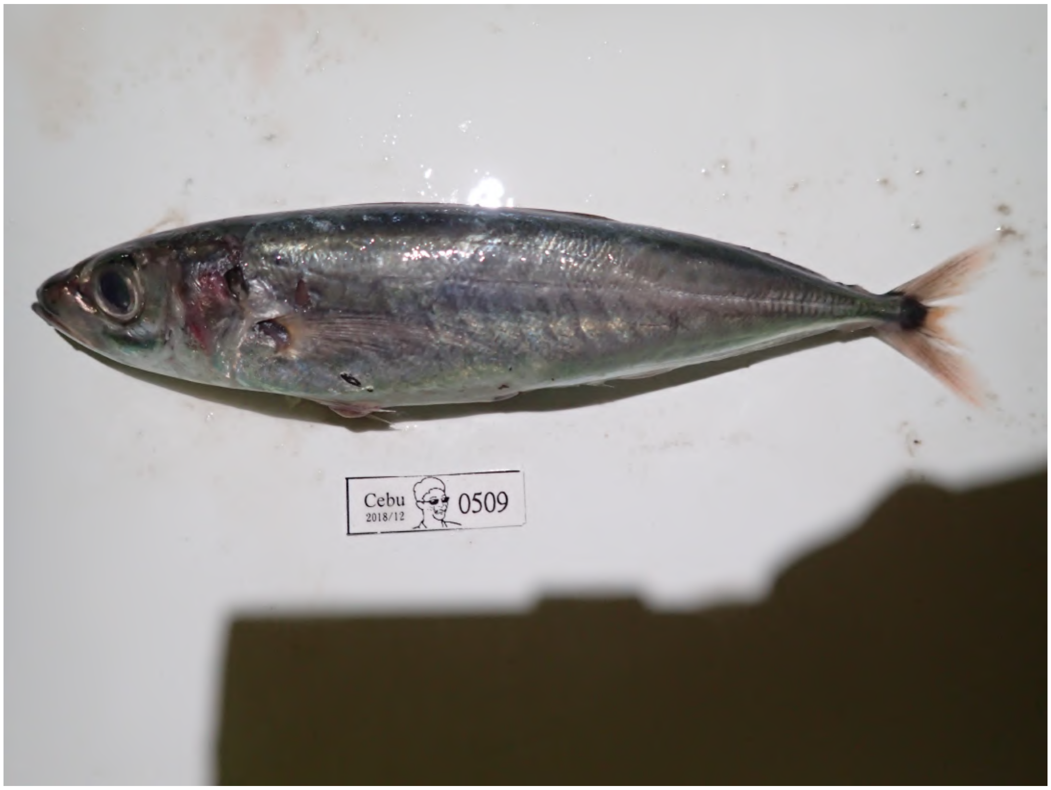

DOS 06643-1, *Decapterus kurroides*, OR113835.

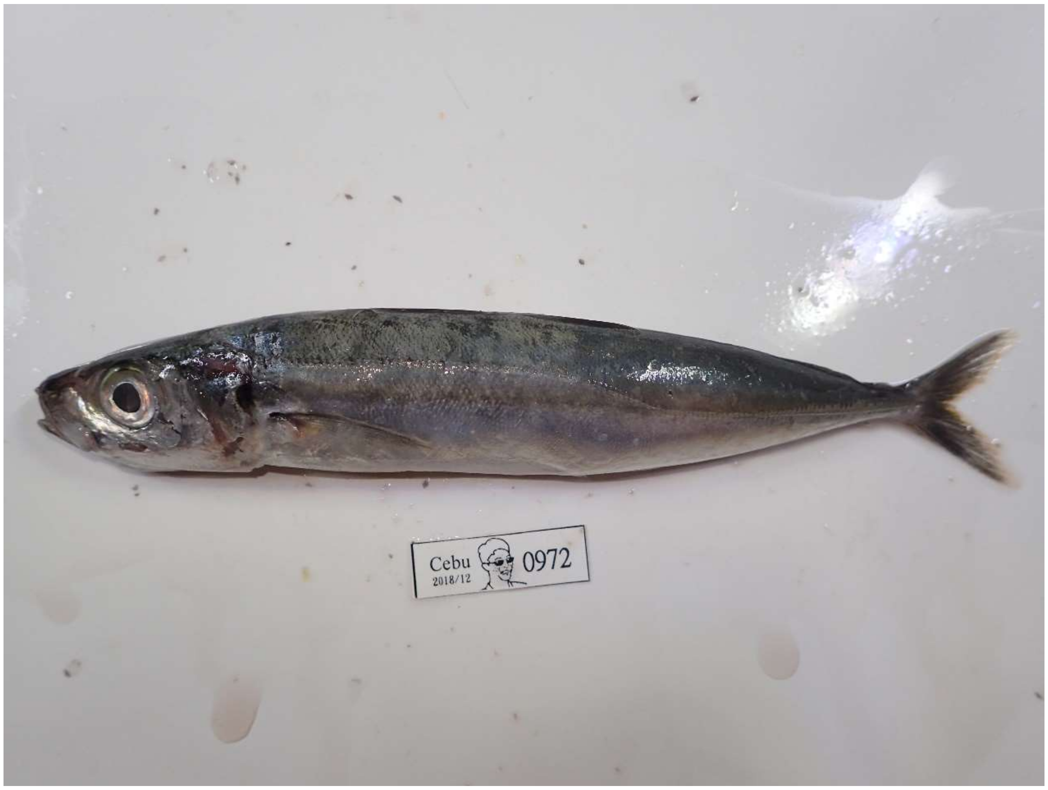

DOS 06651-1, *Decapterus macrosoma*, OR113842.

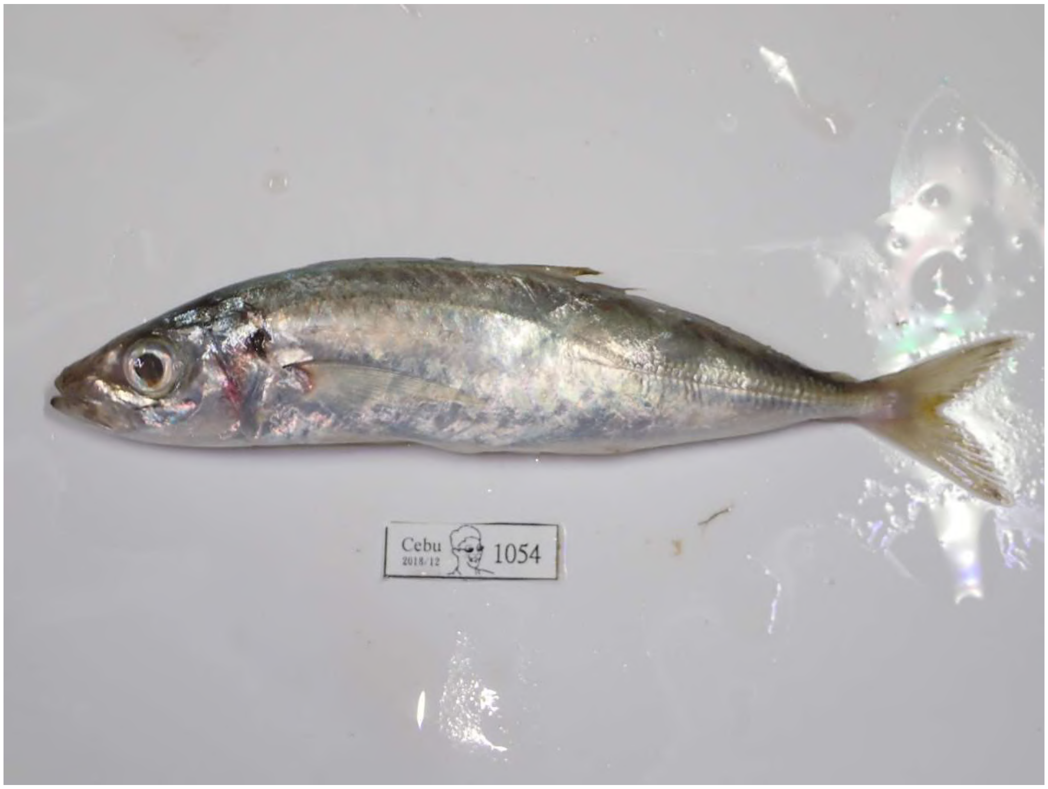

DOS 06642, *Decapterus russelli*, OR113834.

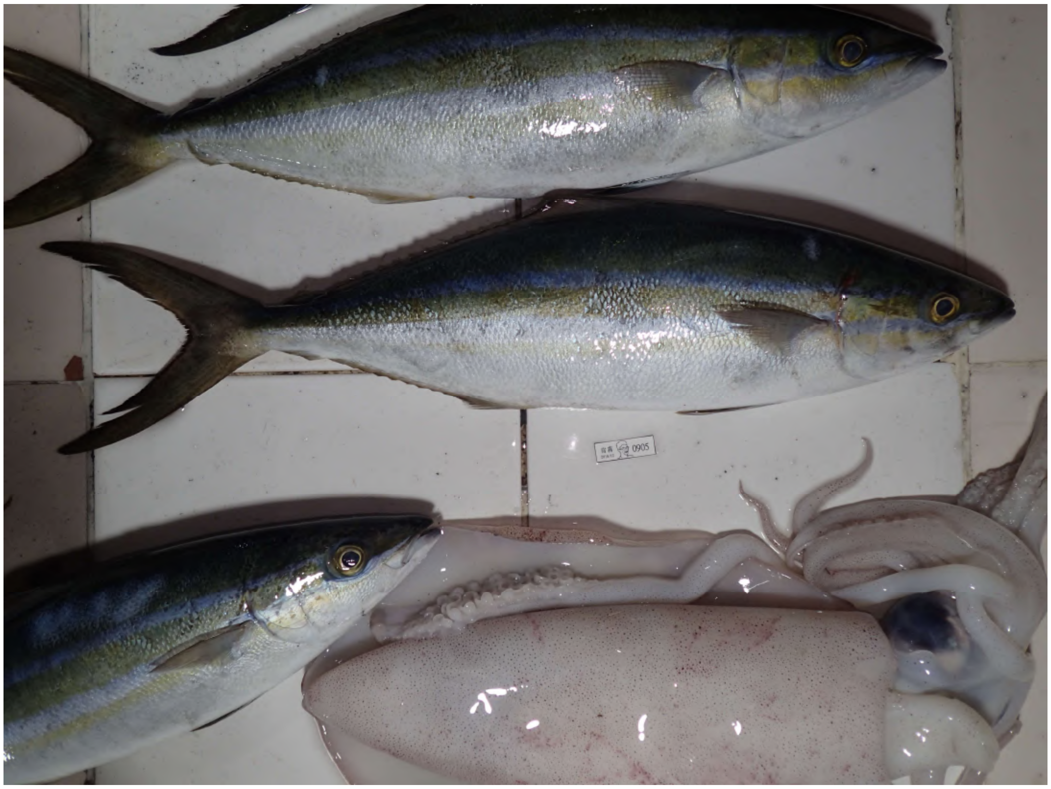

DOS 06644-1, *Elagatis bipinnulata*, OR113836. (specimen not preserved)

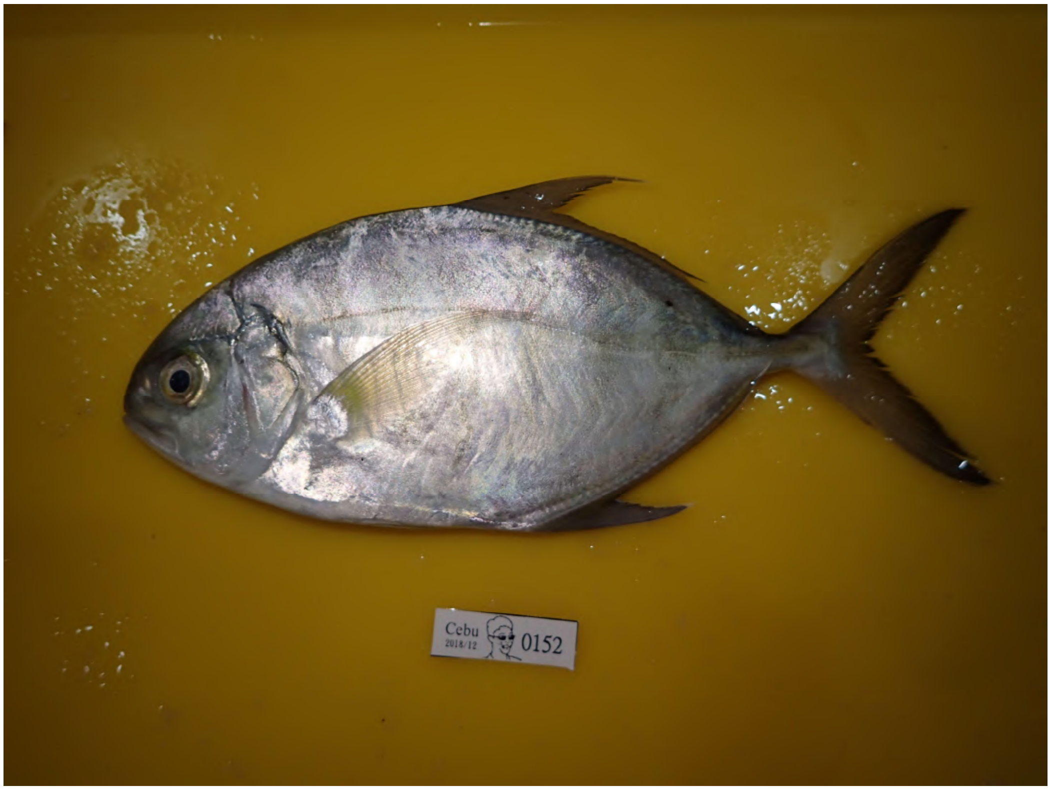

DOS 06638-1, *Ferdauia ferdau*, OR113830. (specimen not preserved)

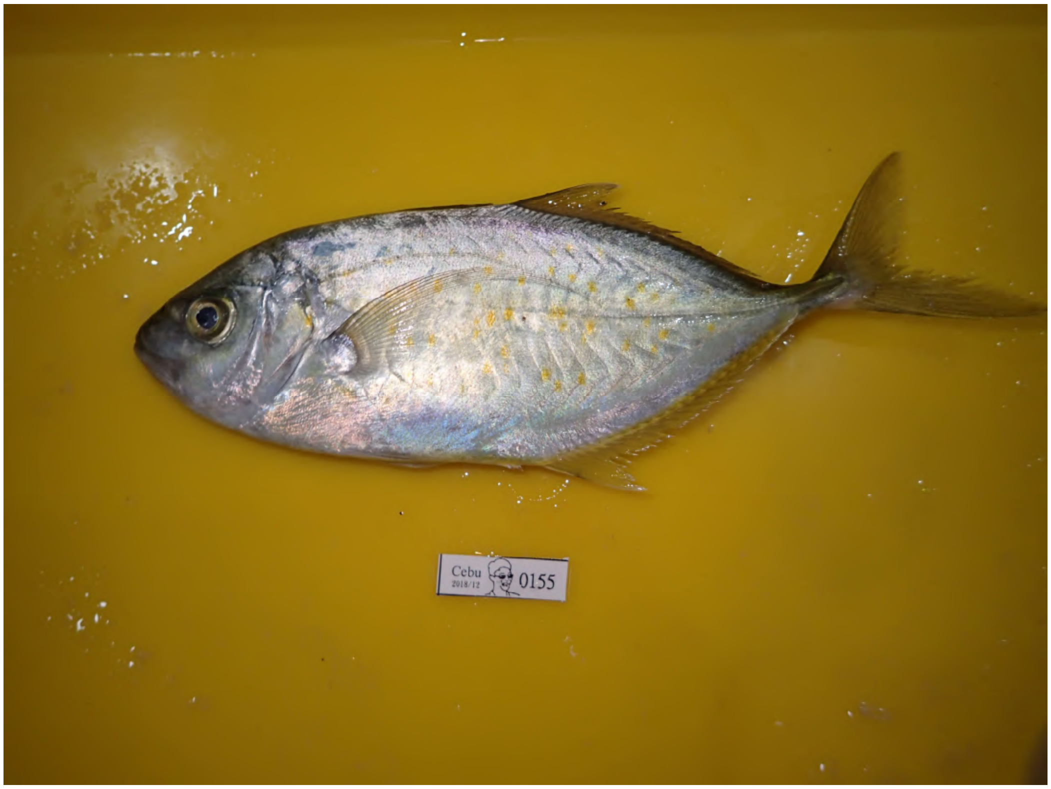

DOS 06637, *Flavocaranx bajad*, OR113829.

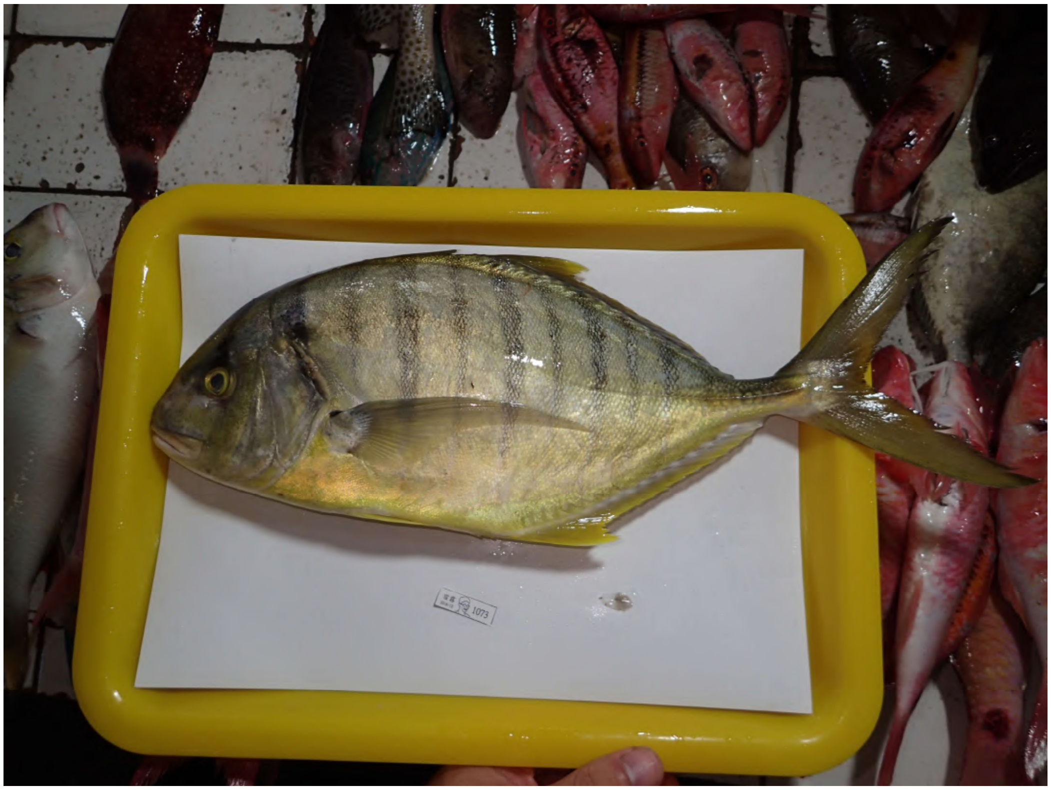

DOS 06645-1, *Gnathanodon speciosus*, OR113837. (specimen not preserved)

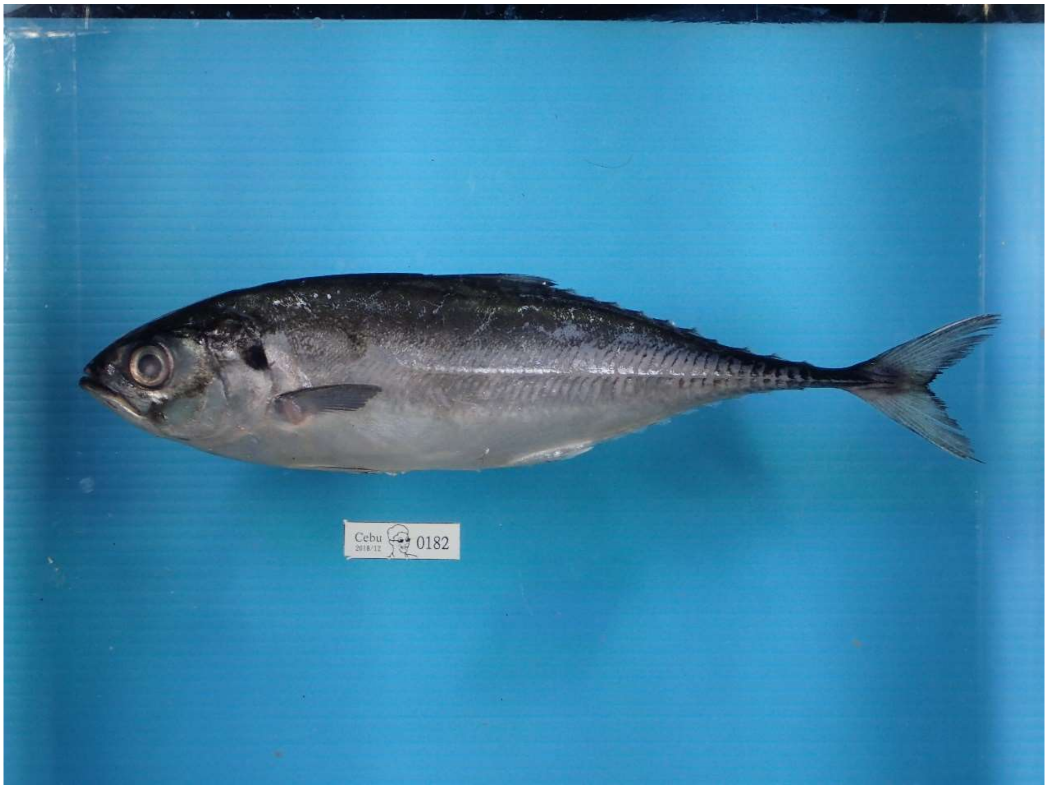

DOS 06646-2, *Megalaspis cordyla*, OR113838. (specimen not preserved)

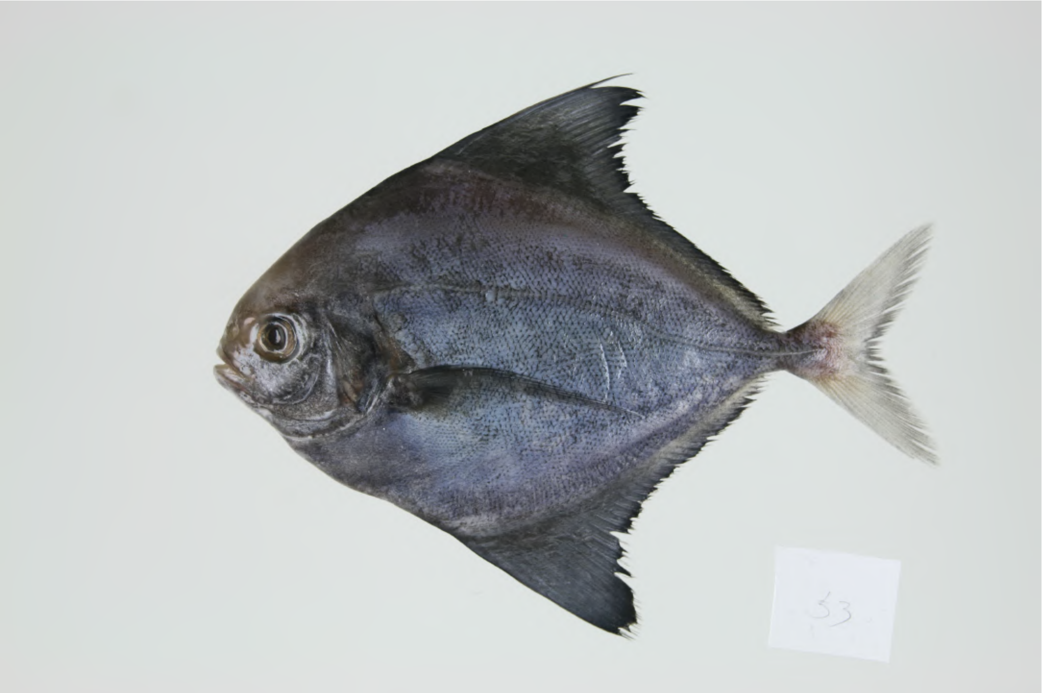

DOS 08636, *Parastromateus niger*, OR114208.

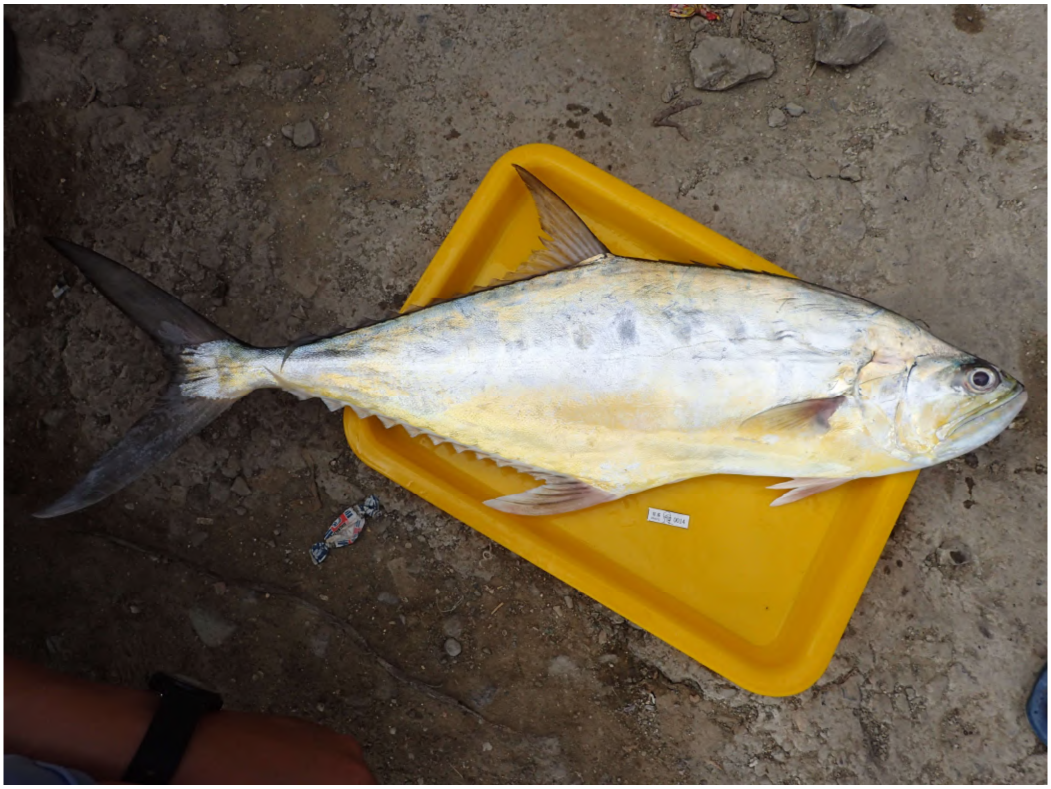

DOS 06649, *Scomberoides commersonnianus*, OR113841. (specimen not preserved)

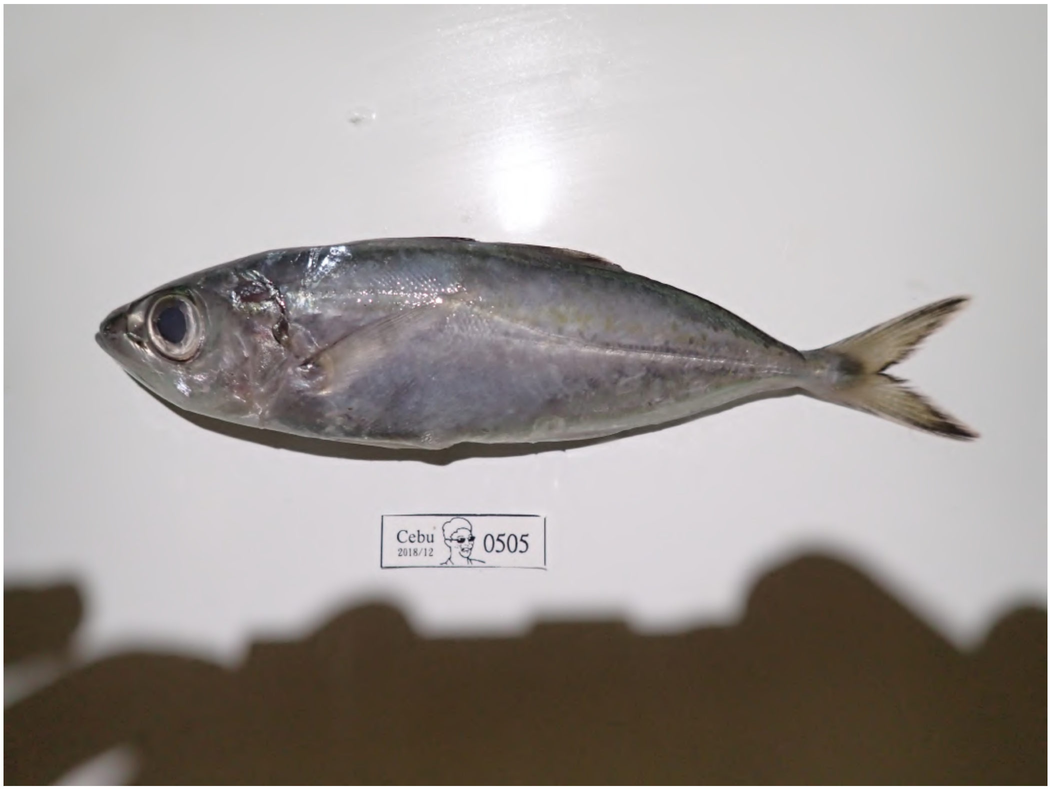

DOS 06652-1, *Selar crumenophthalmus*, OR113843.

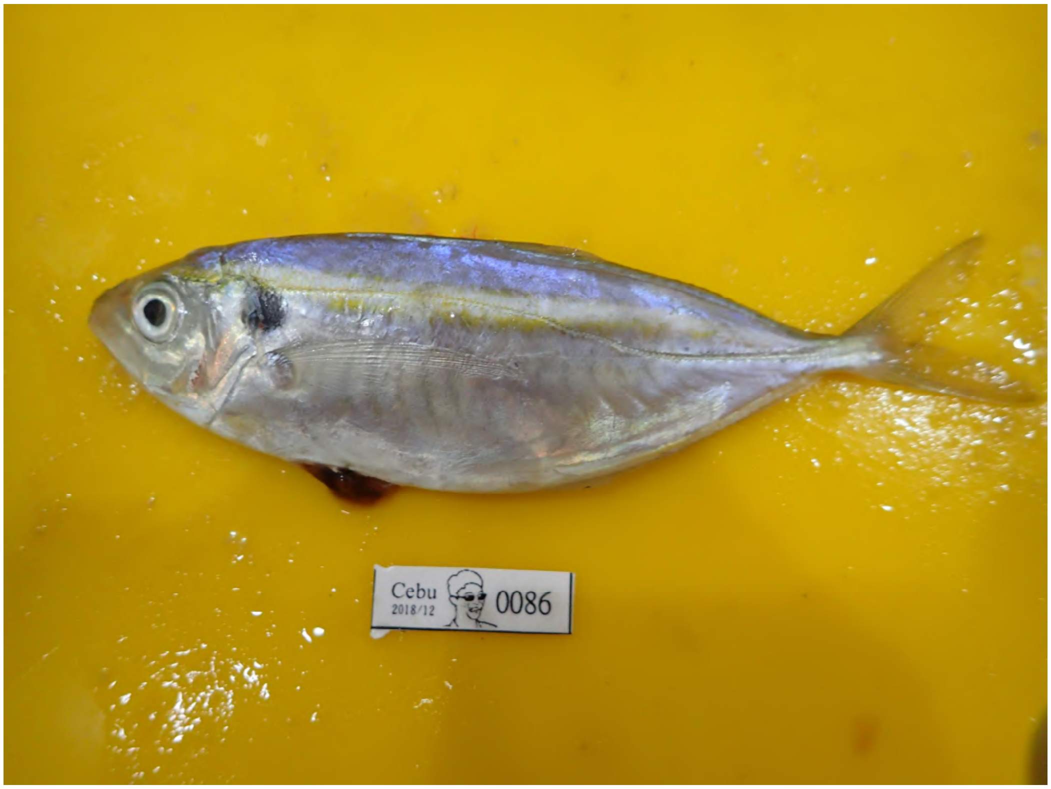

DOS 06648-1, *Selaroides leptolepis*, OR113840. (specimen not preserved)

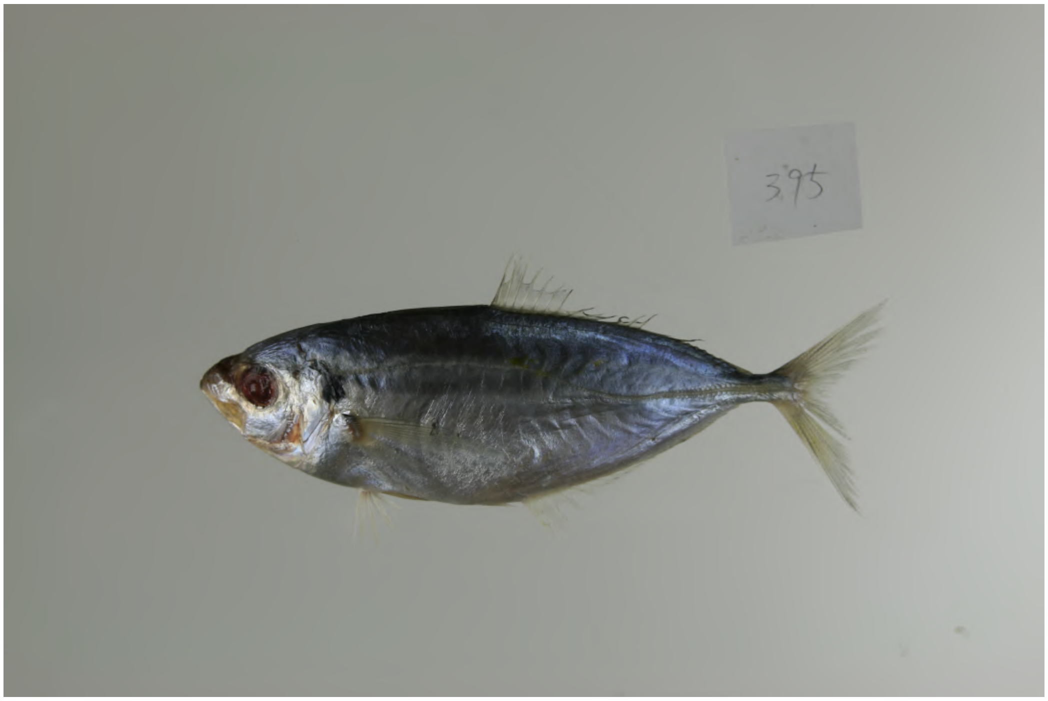

DOS 08637, *Selaroides leptolepis*, OR114209.

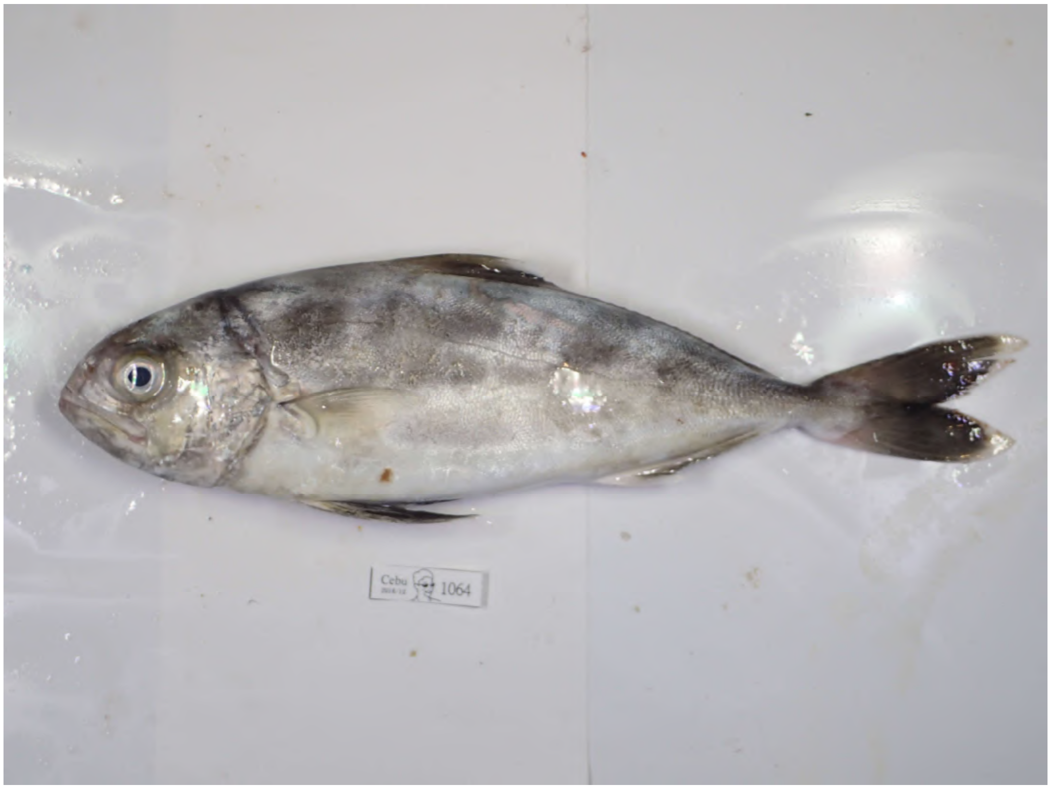

DOS 06647, *Seriolina nigrofasciata*, OR113839.

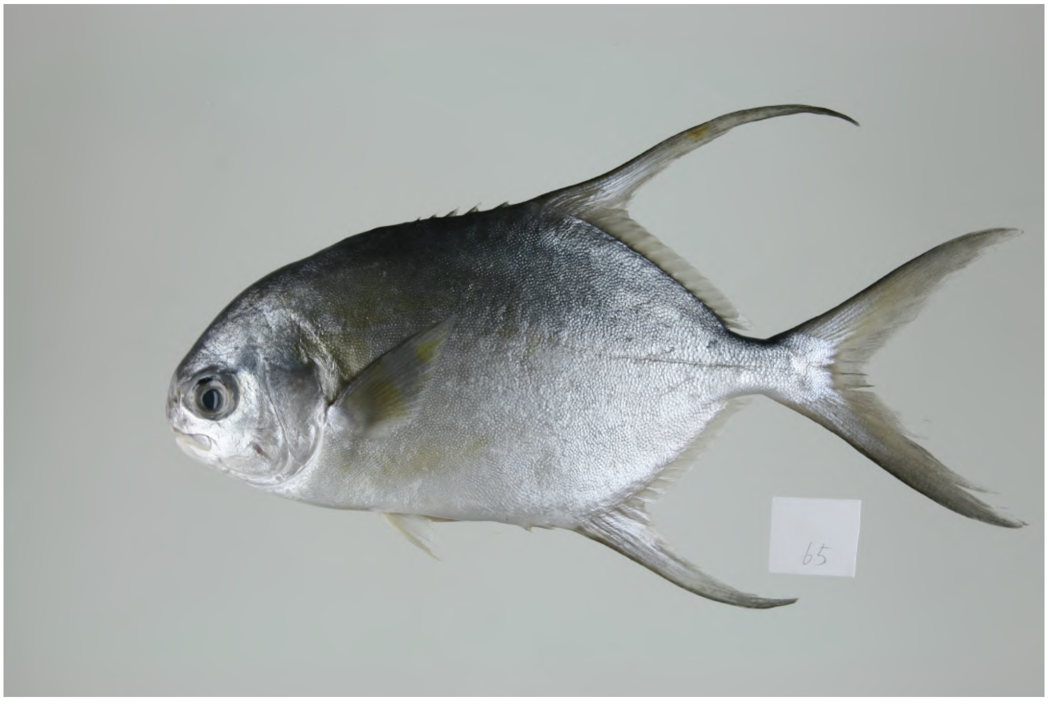

DOS 08638, *Trachinotus blochii*, OR114210.

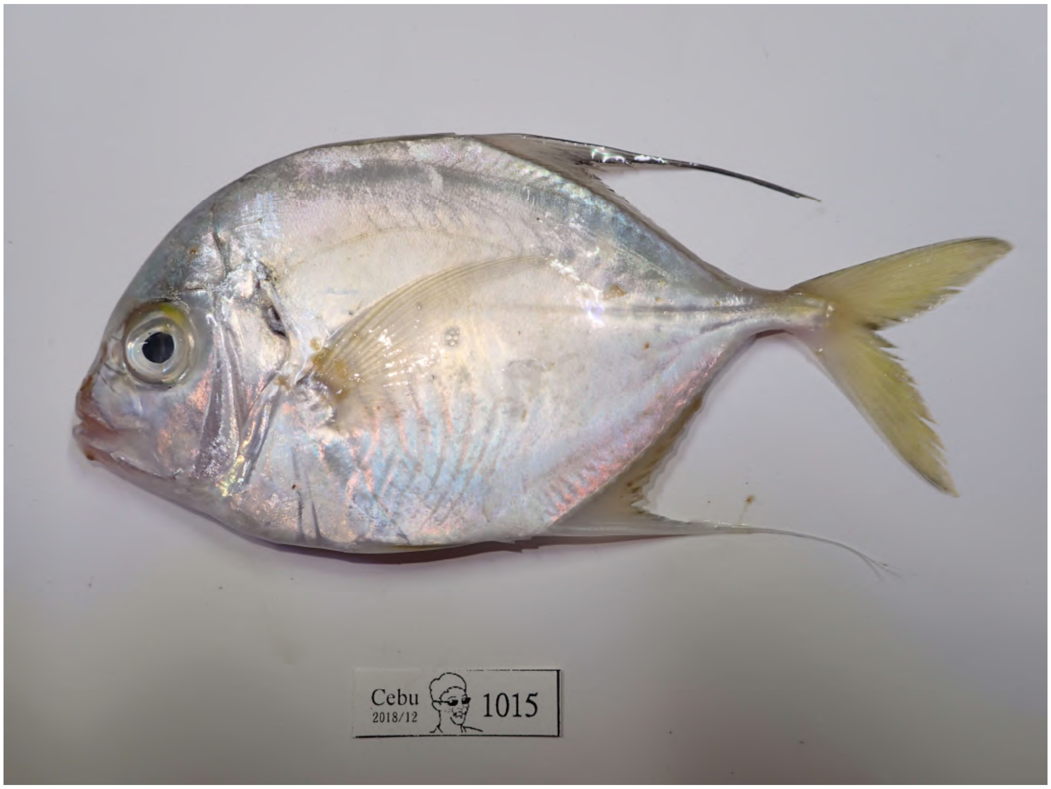

DOS 06635-1, *Turrum coeruleopinnatum*, OR113827. (specimen not preserved)

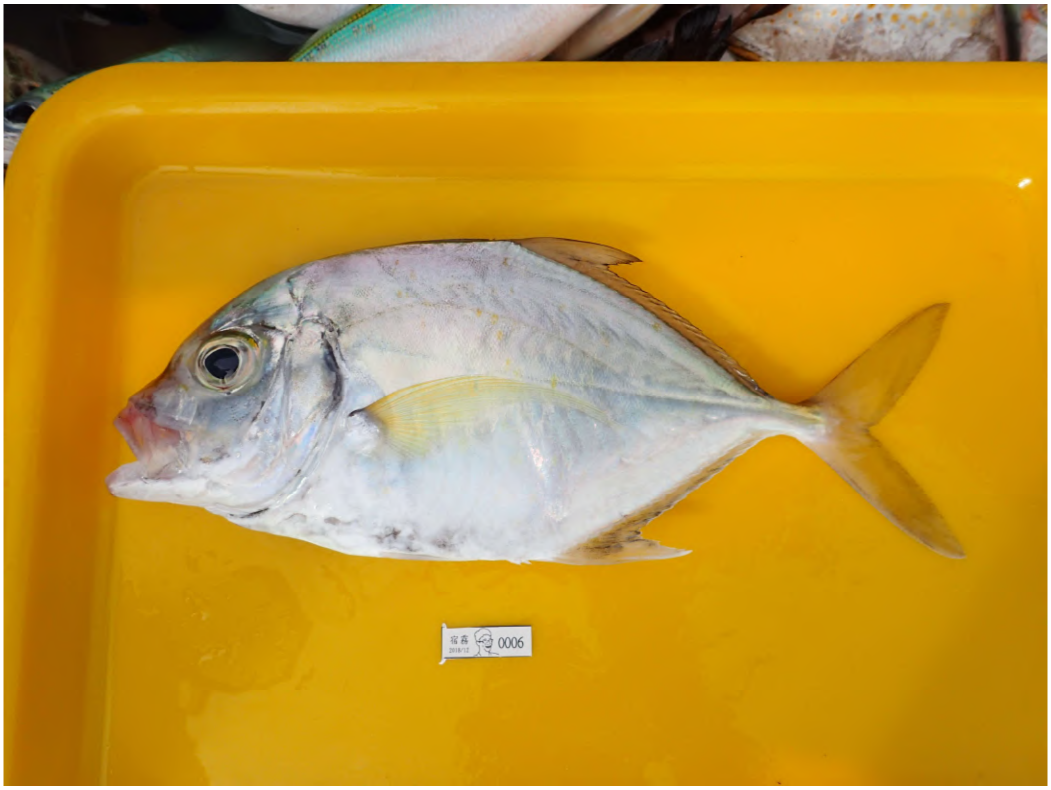

DOS 06636-1, *Turrum coeruleopinnatum*, OR113828. (specimen not preserved)

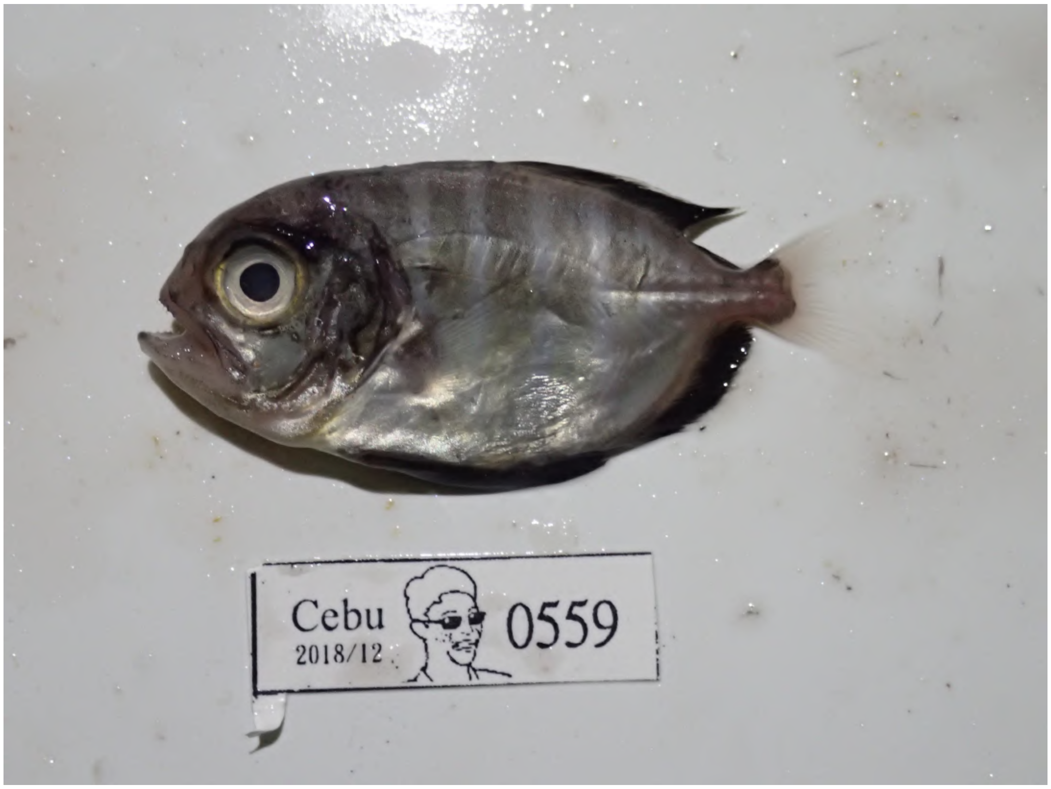

DOS 06654, *Uraspis uraspis*, OR113845.

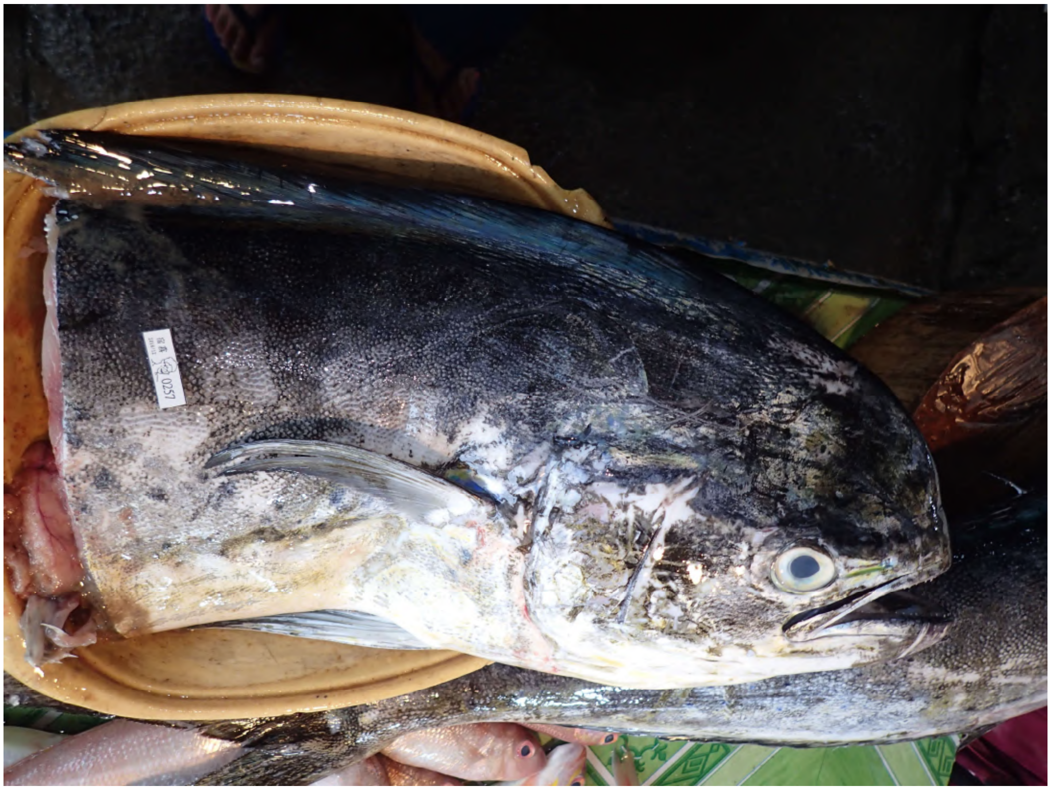

DOS 06682, *Coryphaena hippurus*, OR113872. (specimen not preserved)

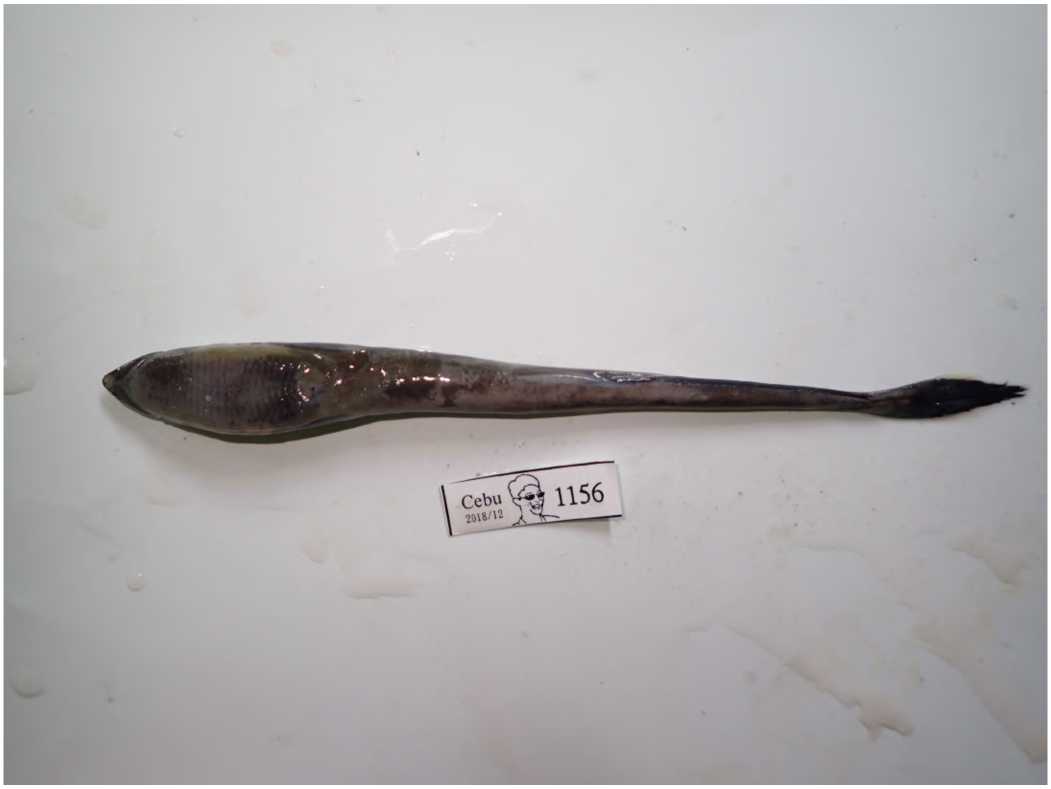

DOS 06688, *Echeneis naucrates*, OR113878.

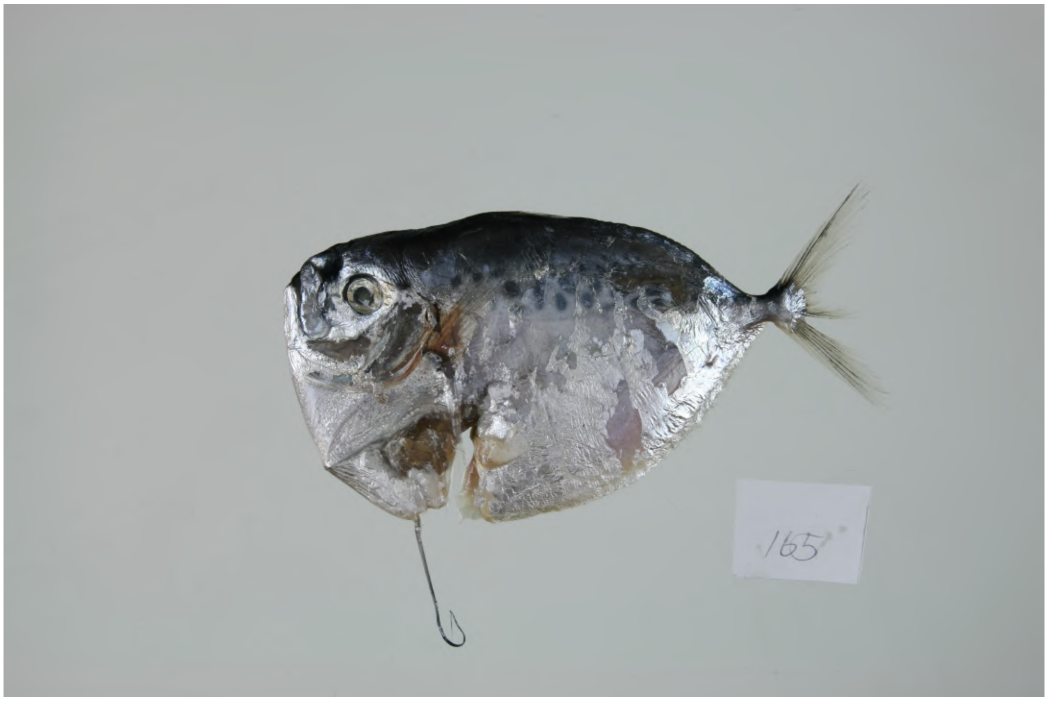

DOS 08639, *Mene maculata*, OR114211.

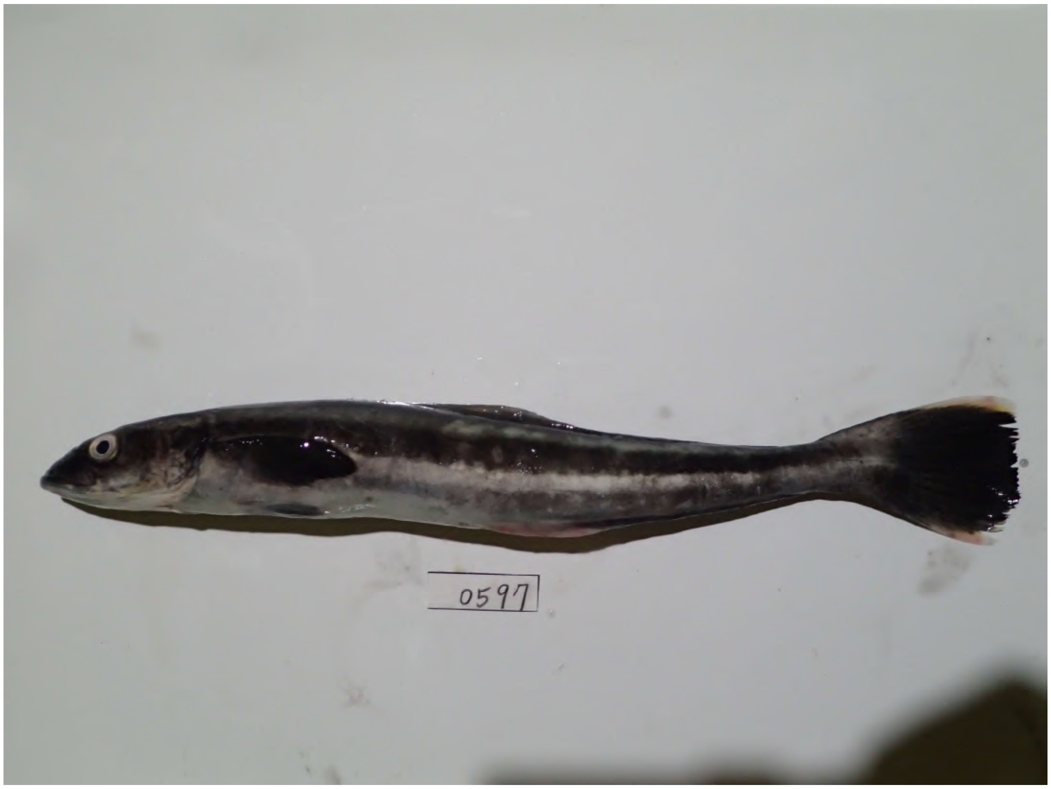

DOS 07007-1, *Rachycentron canadum*, OR114174.

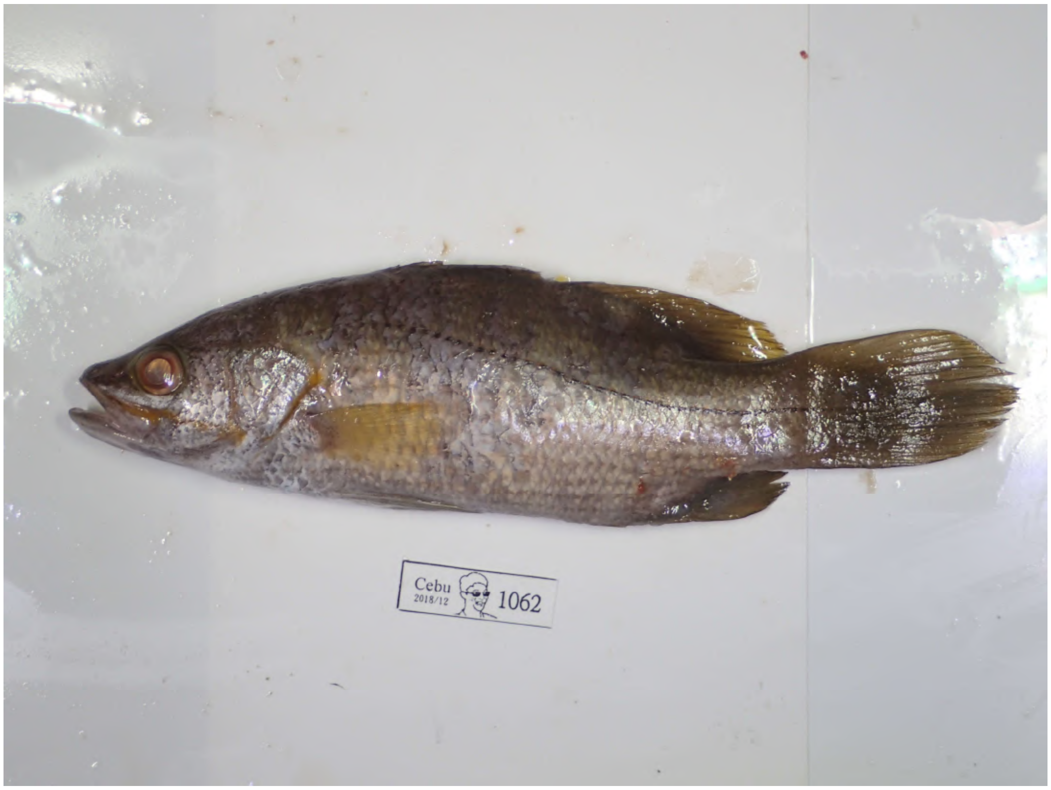

DOS 06764-1, *Psammoperca waigiensis*, OR113949. (specimen not preserved)

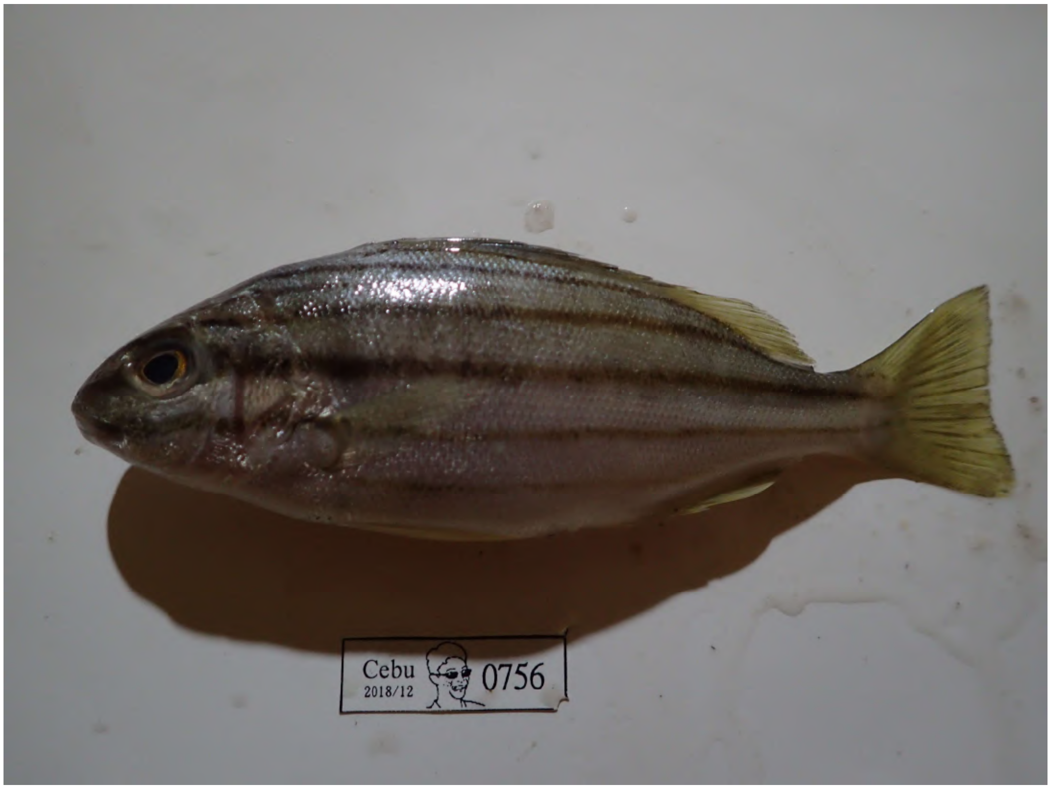

DOS 06979, *Pelates quadrilineatus*, OR114149.

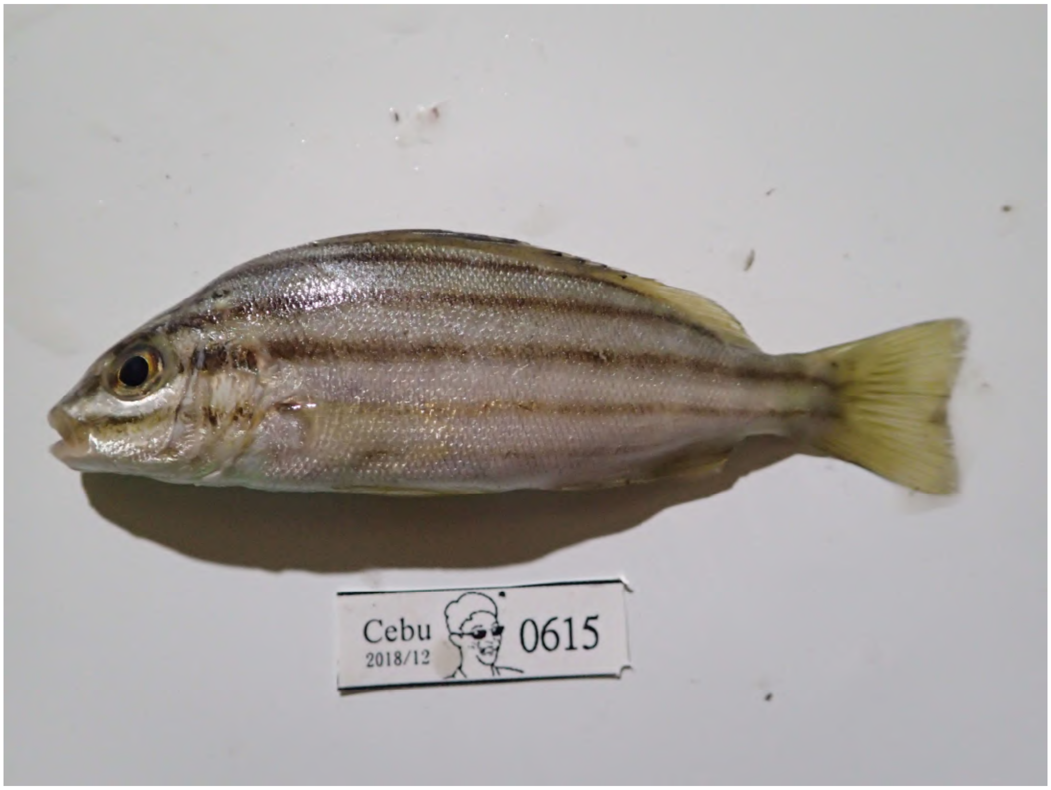

DOS 06980-1, *Pelates quadrilineatus*, OR114150.

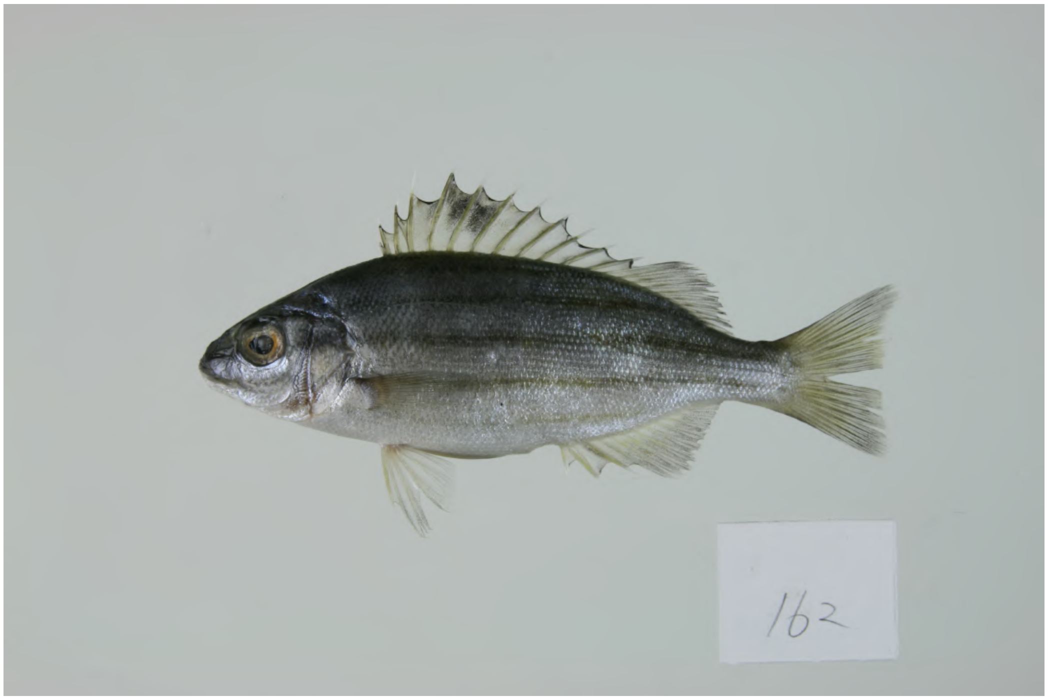

DOS 08668-1, *Pelates quadrilineatus*, OR114245.

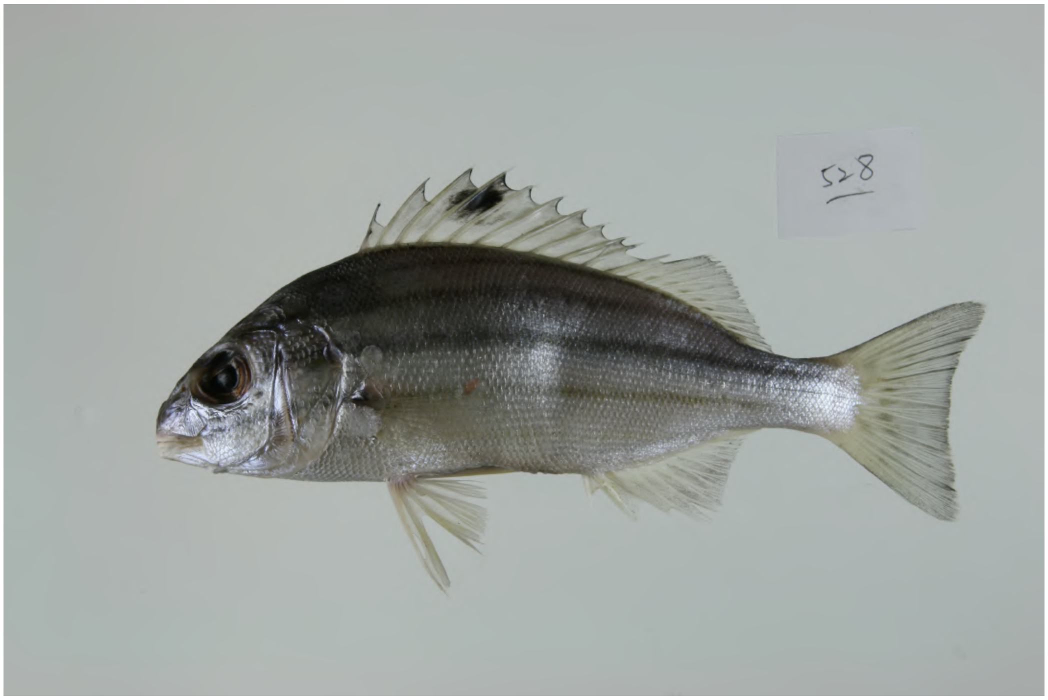

DOS 08668-2, *Pelates quadrilineatus*, OR114246.

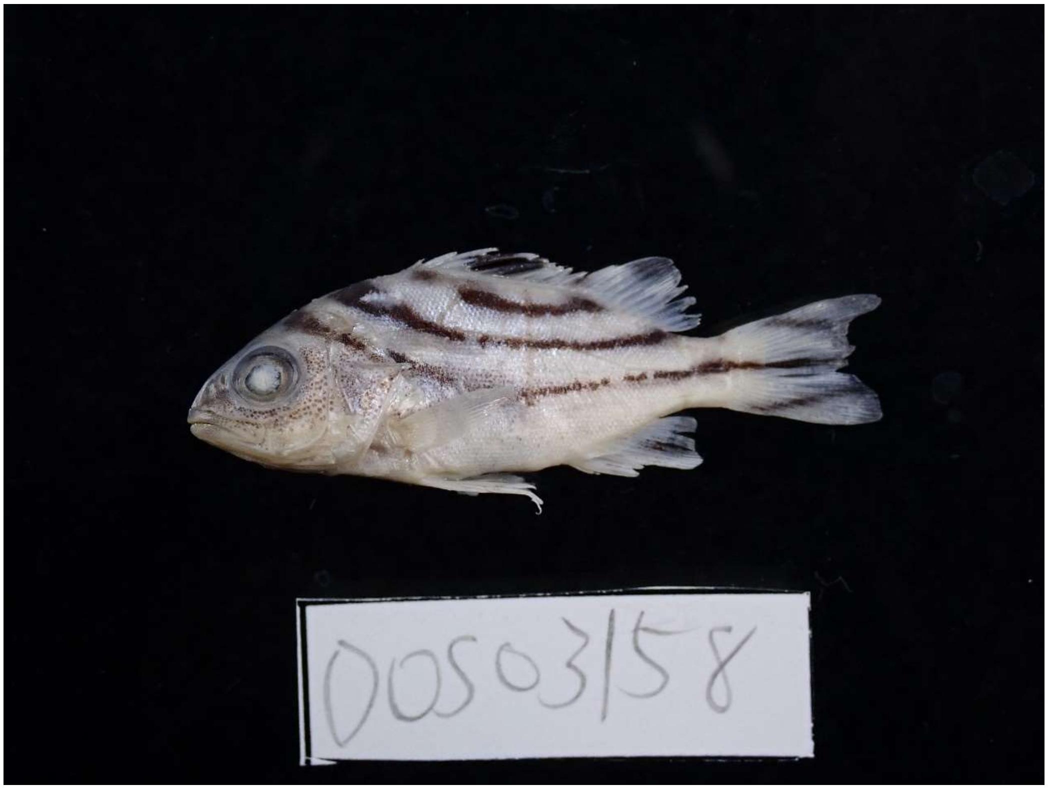

DOS 03158, *Terapon jarbua*, OR113753.

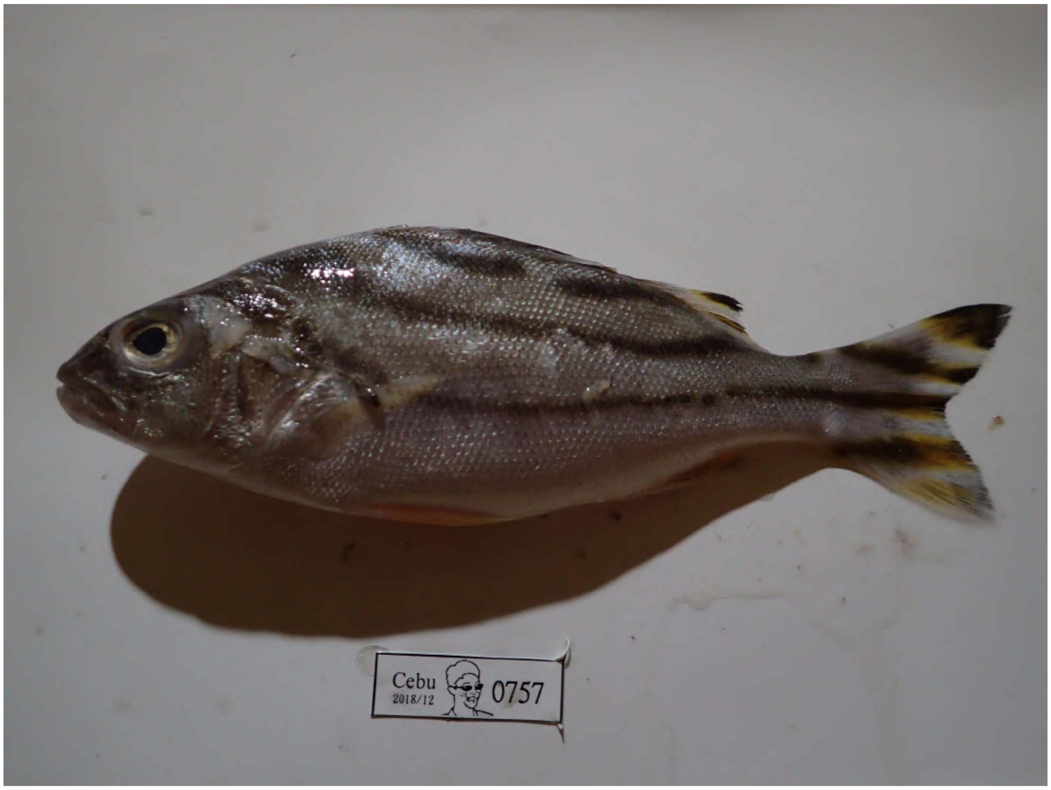

DOS 06981-1, *Terapon jarbua*, OR114151. (specimen not preserved)

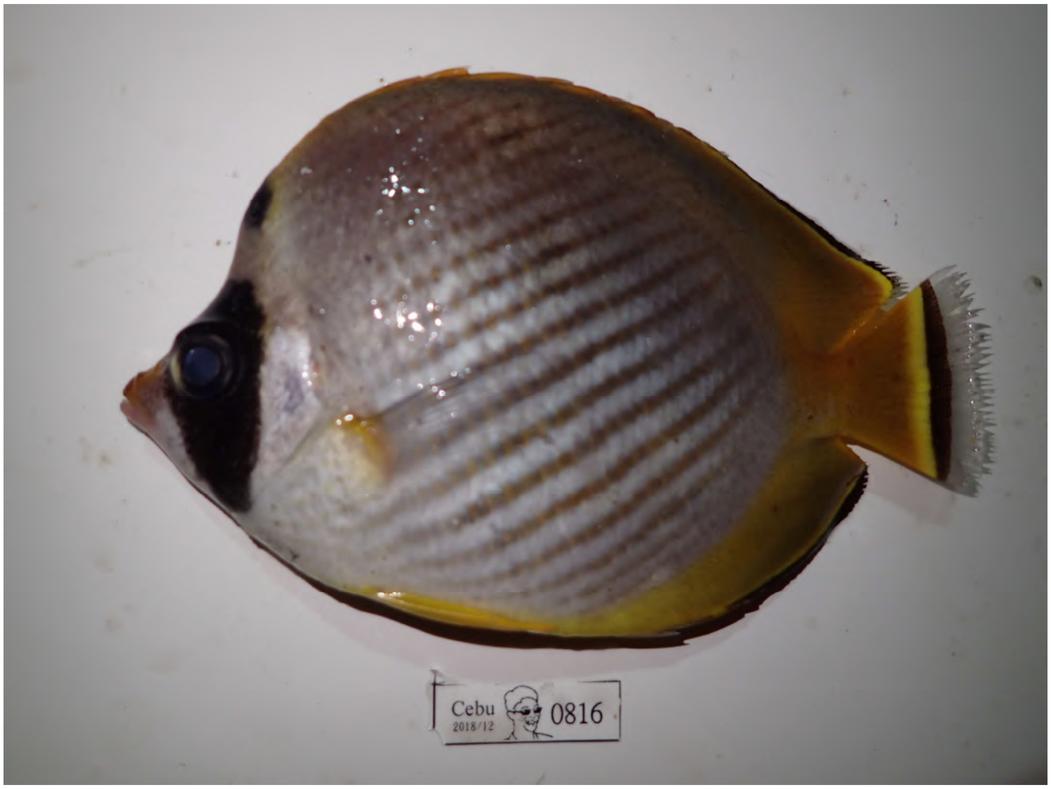

DOS 06657-1, *Chaetodon adiergastos*, OR113848.

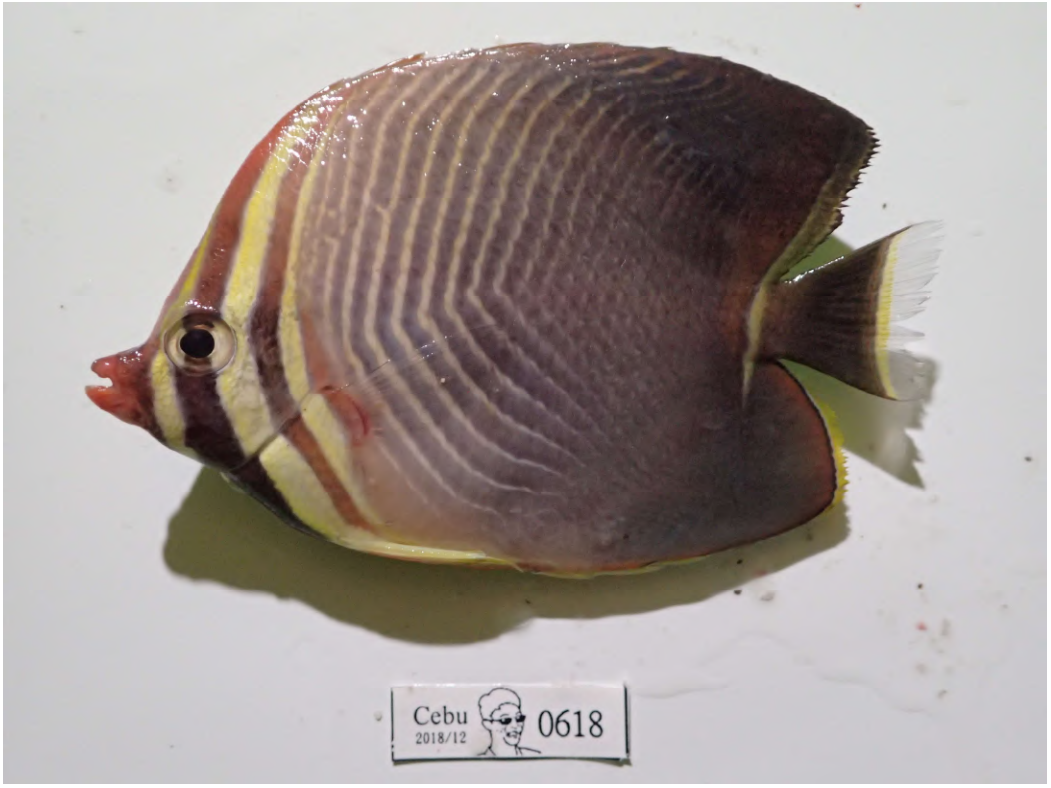

DOS 06658-1, *Chaetodon baronessa*, OR113849.

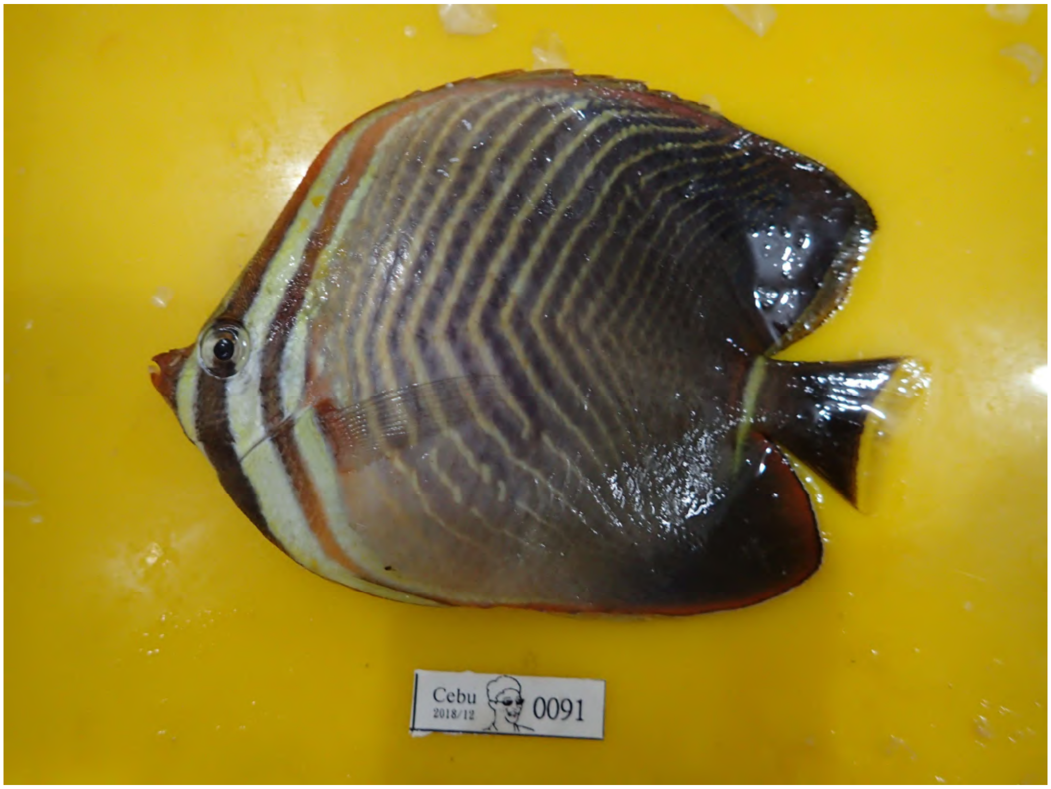

DOS 06664-1, *Chaetodon baronessa*, OR113855. (specimen not preserved)

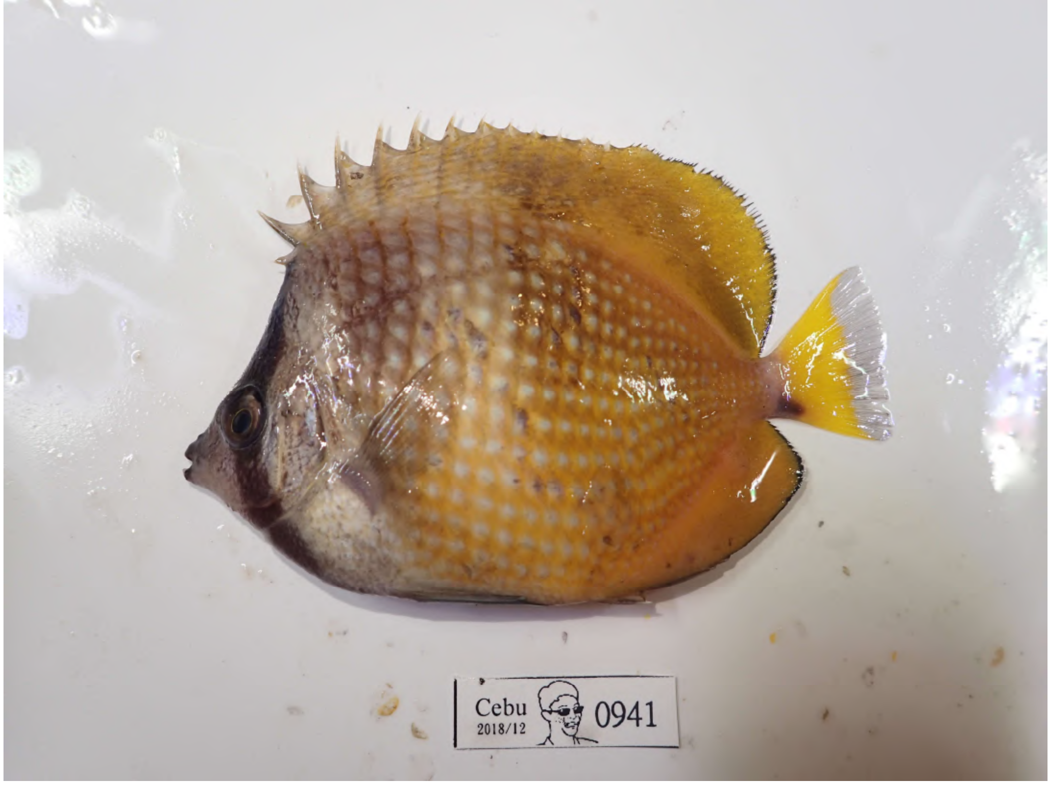

DOS 06659, *Chaetodon kleinii*, OR113850.

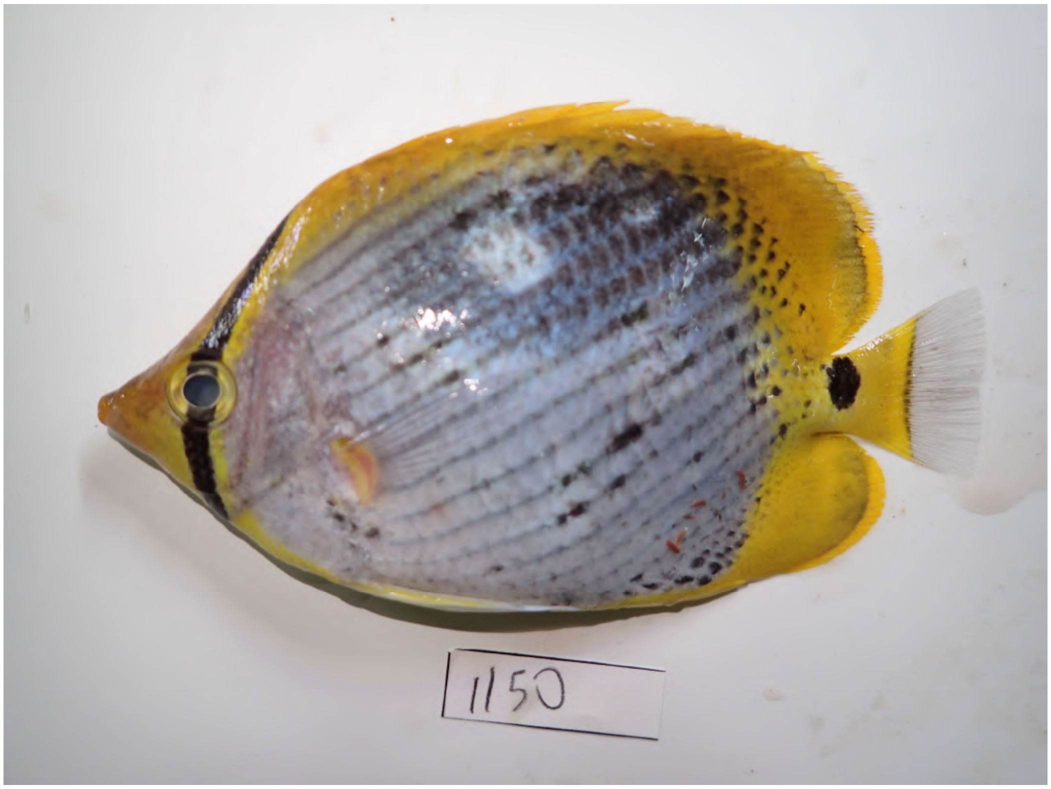

DOS 06660, *Chaetodon ocellicaudus*, OR113851.

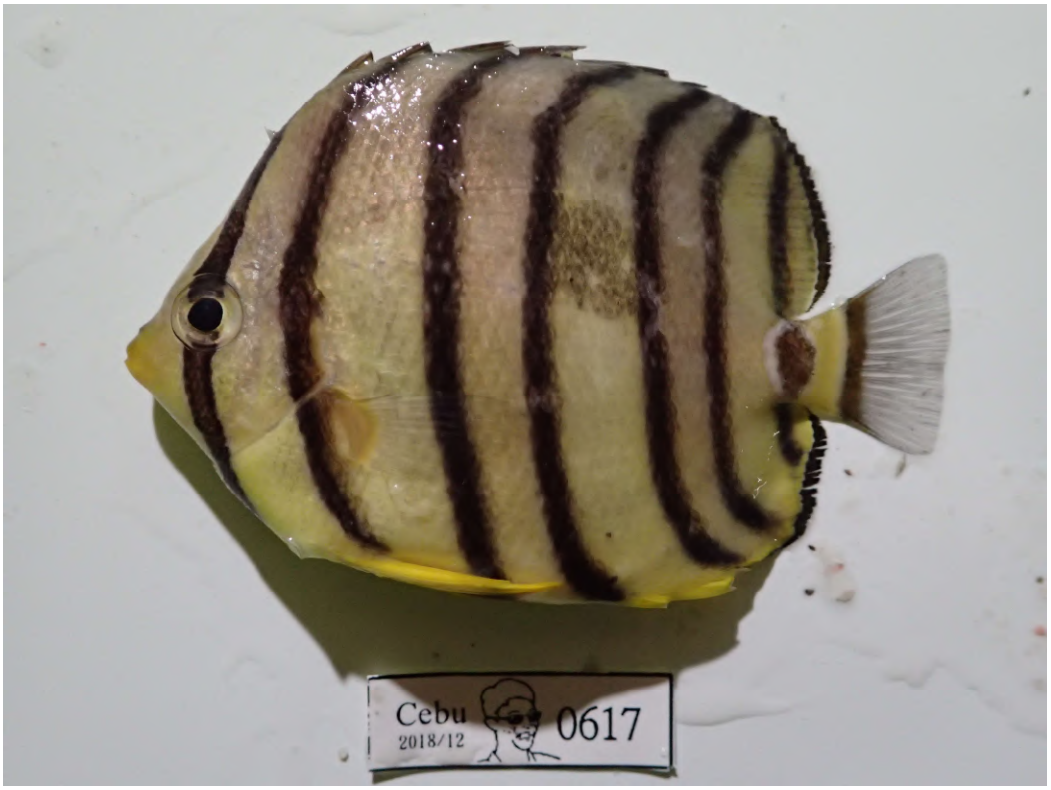

DOS 06661-1, *Chaetodon octofasciatus*, OR113852.

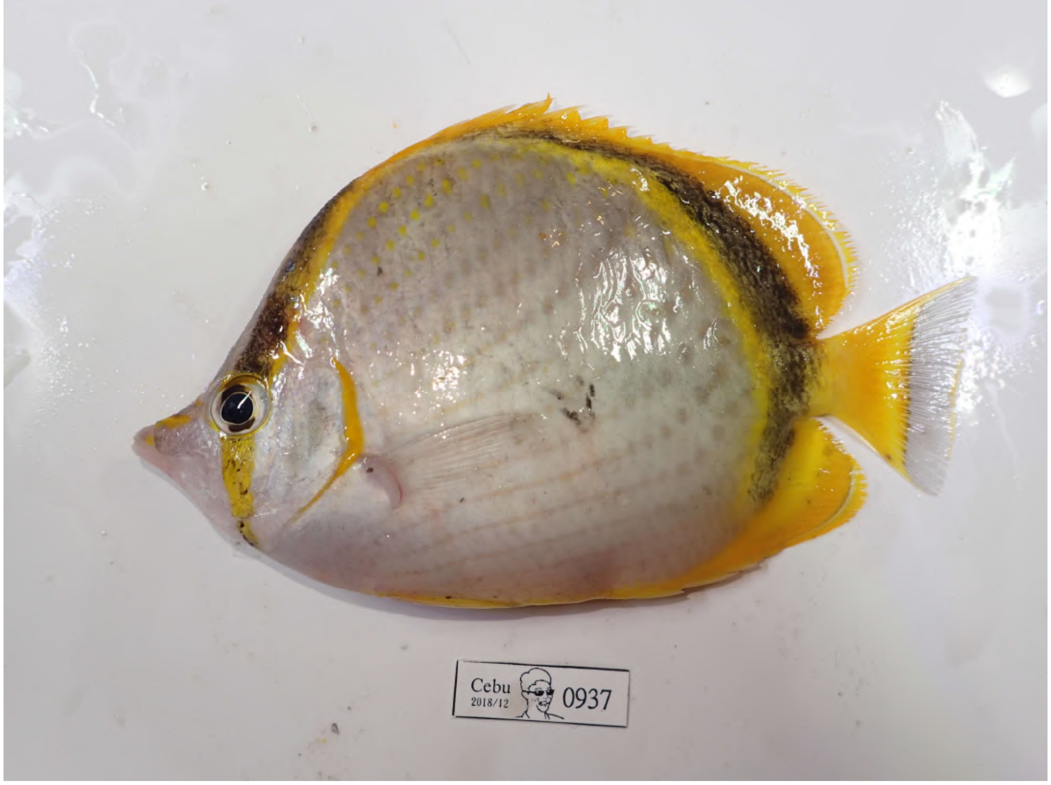

DOS 06662, *Chaetodon selene*, OR113853.

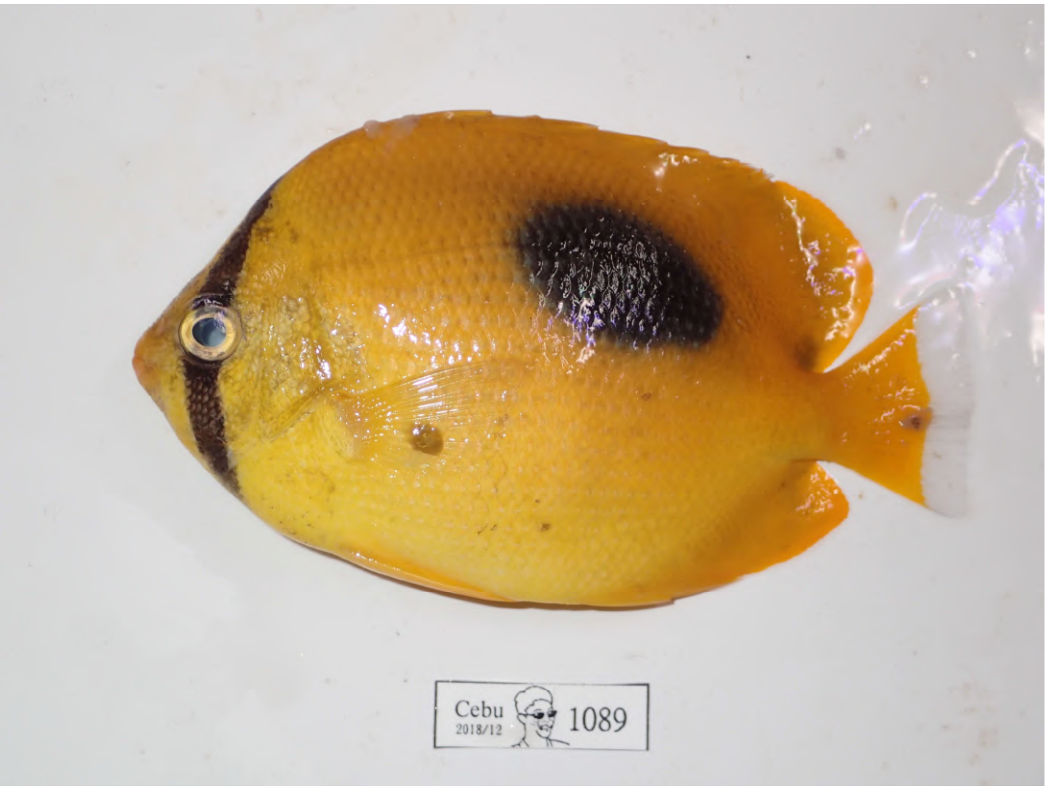

DOS 06665, *Chaetodon speculum*, OR113856.

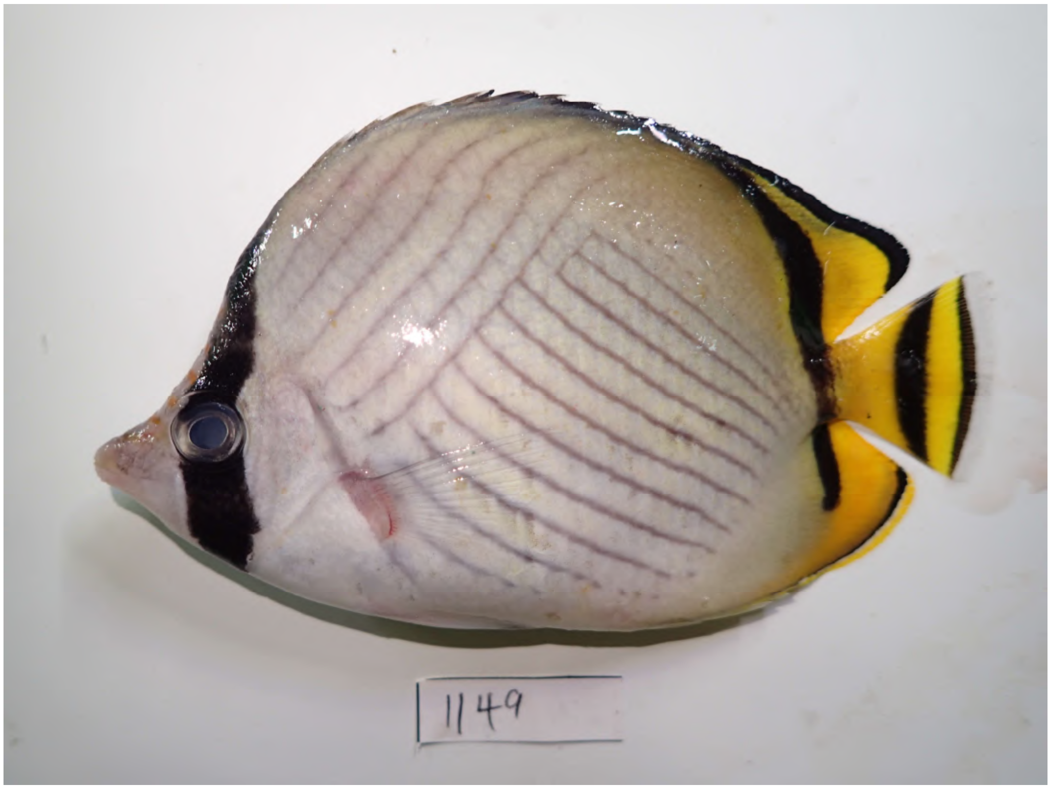

DOS 06666, *Chaetodon vagabundus*, OR113857.

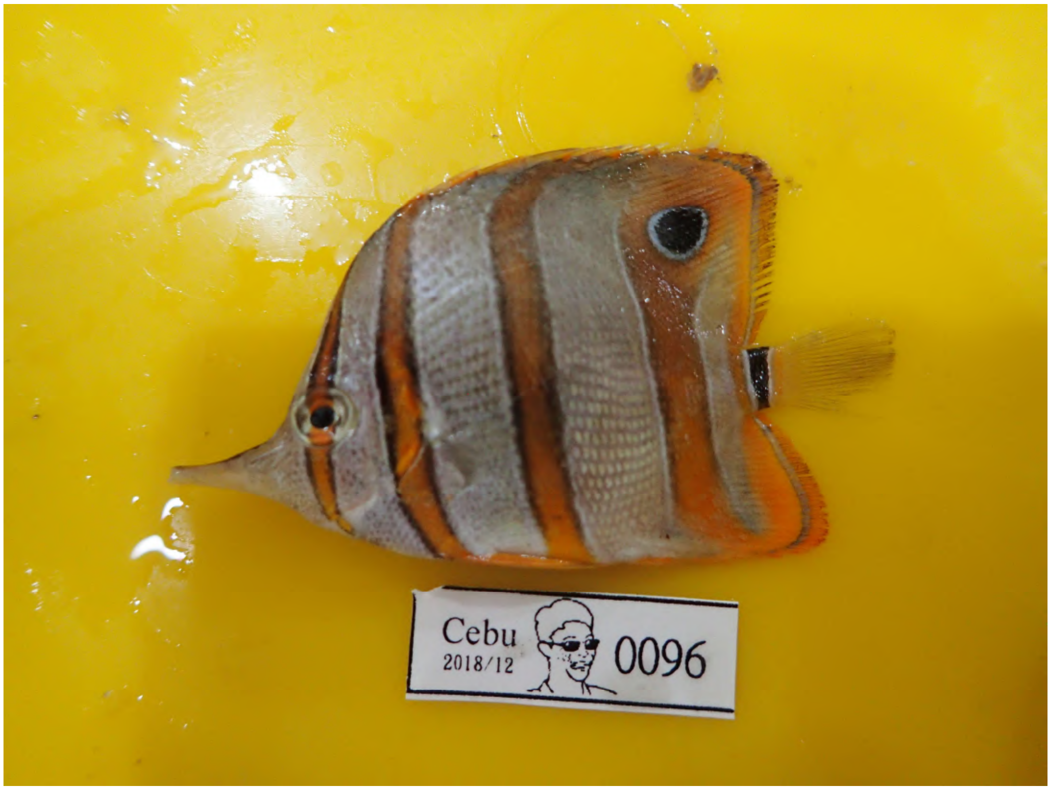

DOS 06668, *Chelmon rostratus*, OR113859.

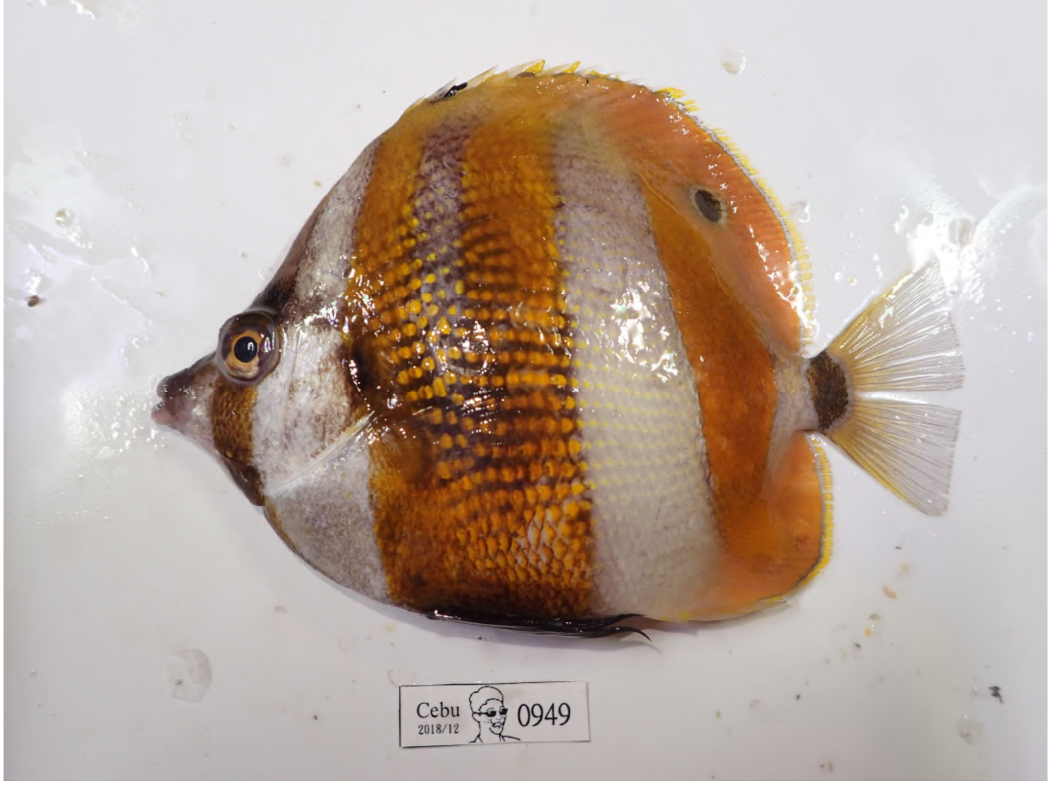

DOS 06669-1, *Coradion chrysozonus*, OR113860.

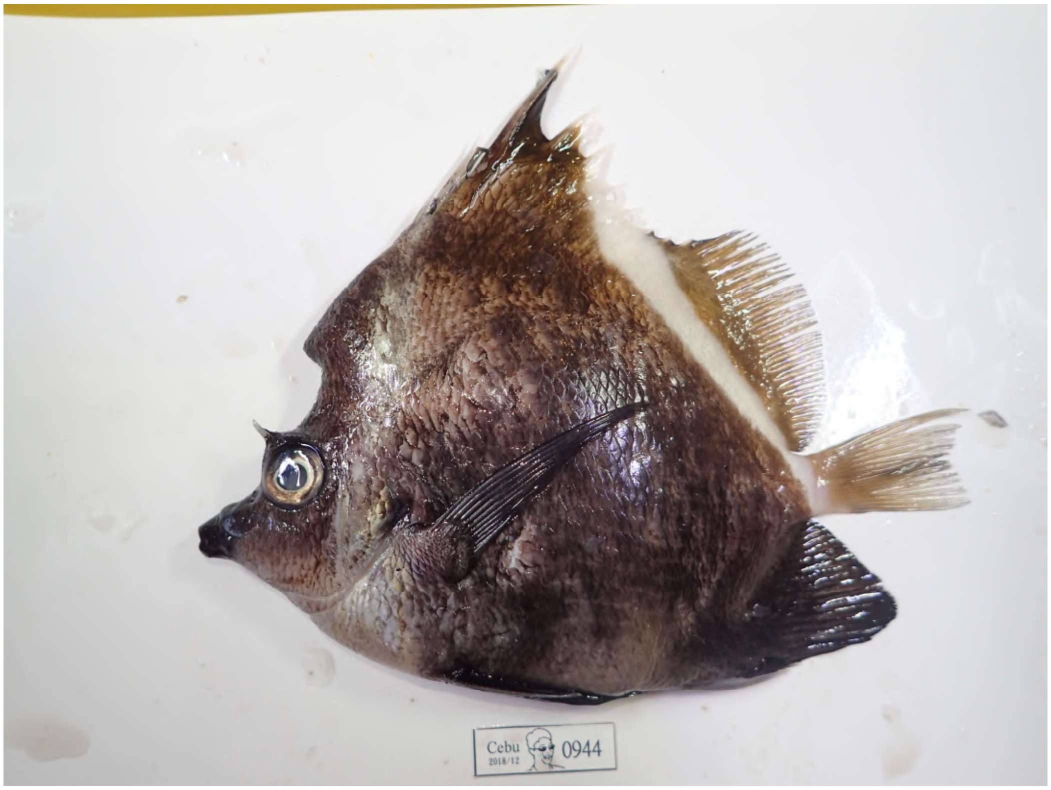

DOS 06670-1, *Heniochus varius*, OR113861.

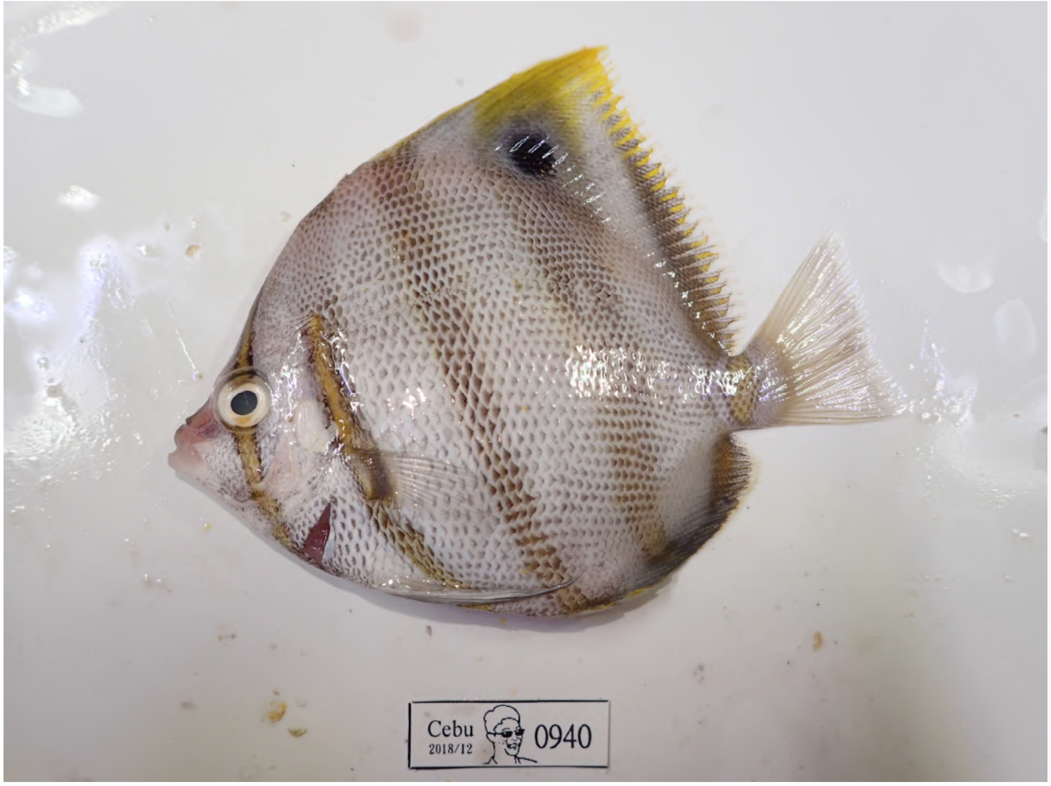

DOS 06663-1, *Parachaetodon ocellatus*, OR113854. (specimen not preserved)

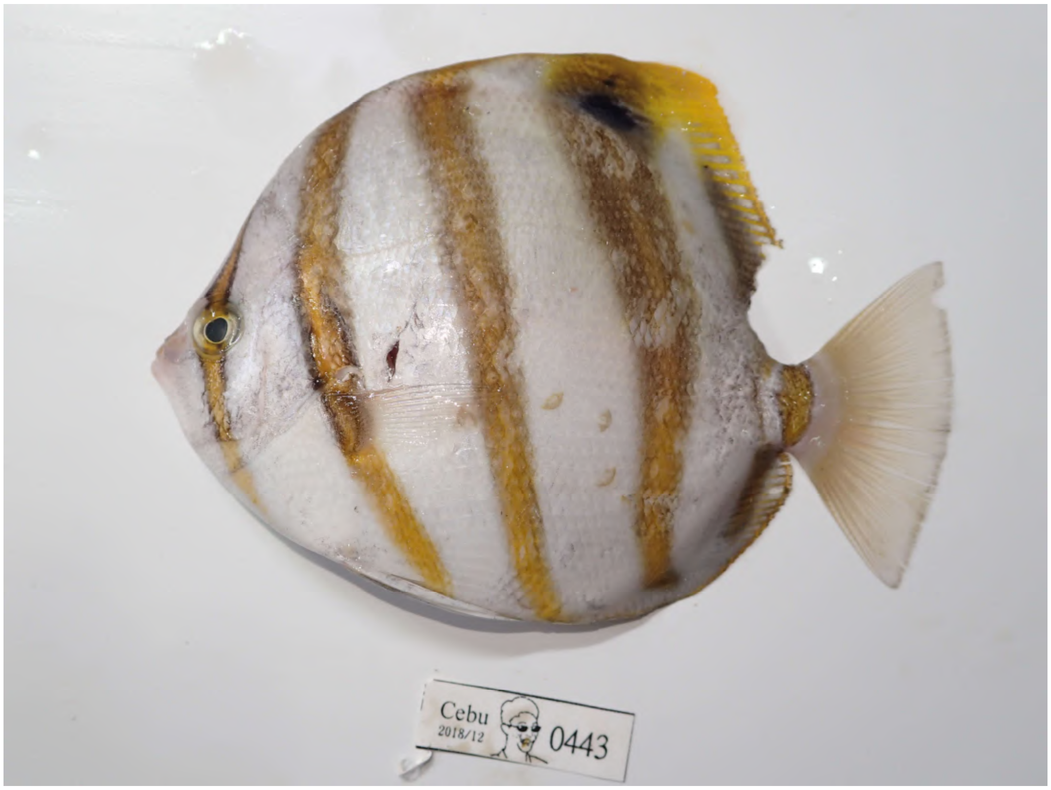

DOS 06667-1, *Parachaetodon ocellatus*, OR113858.

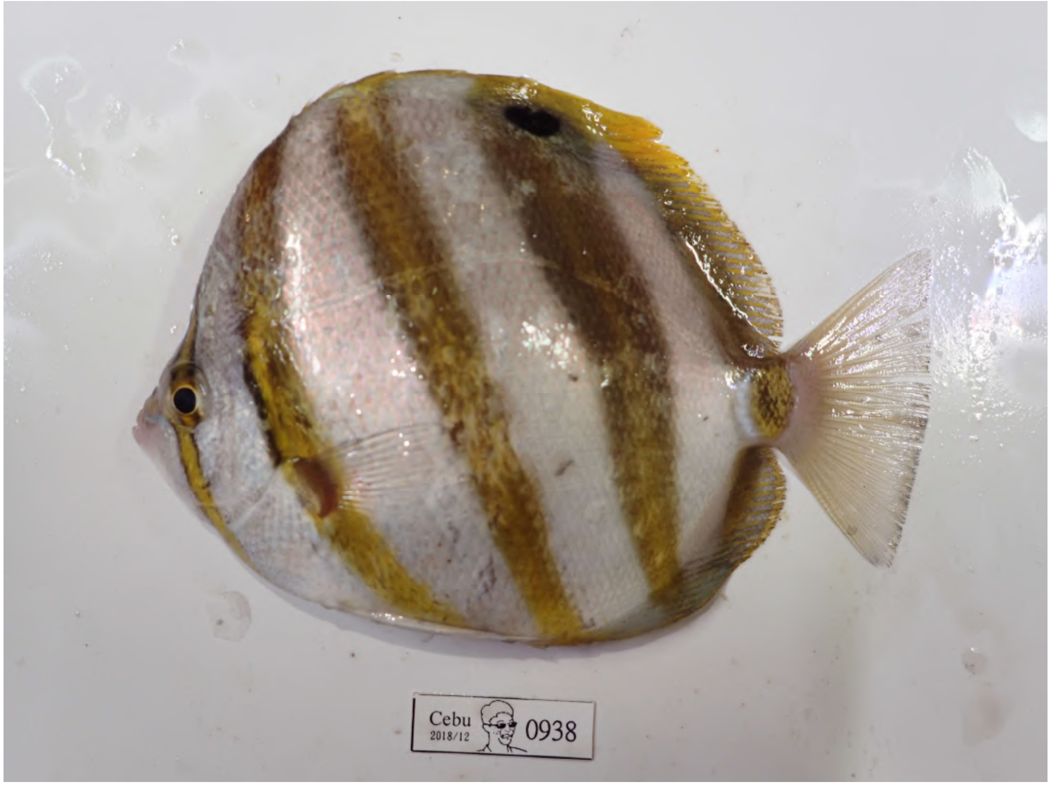

DOS 06671-1, *Parachaetodon ocellatus*, OR113862. (specimen not preserved)

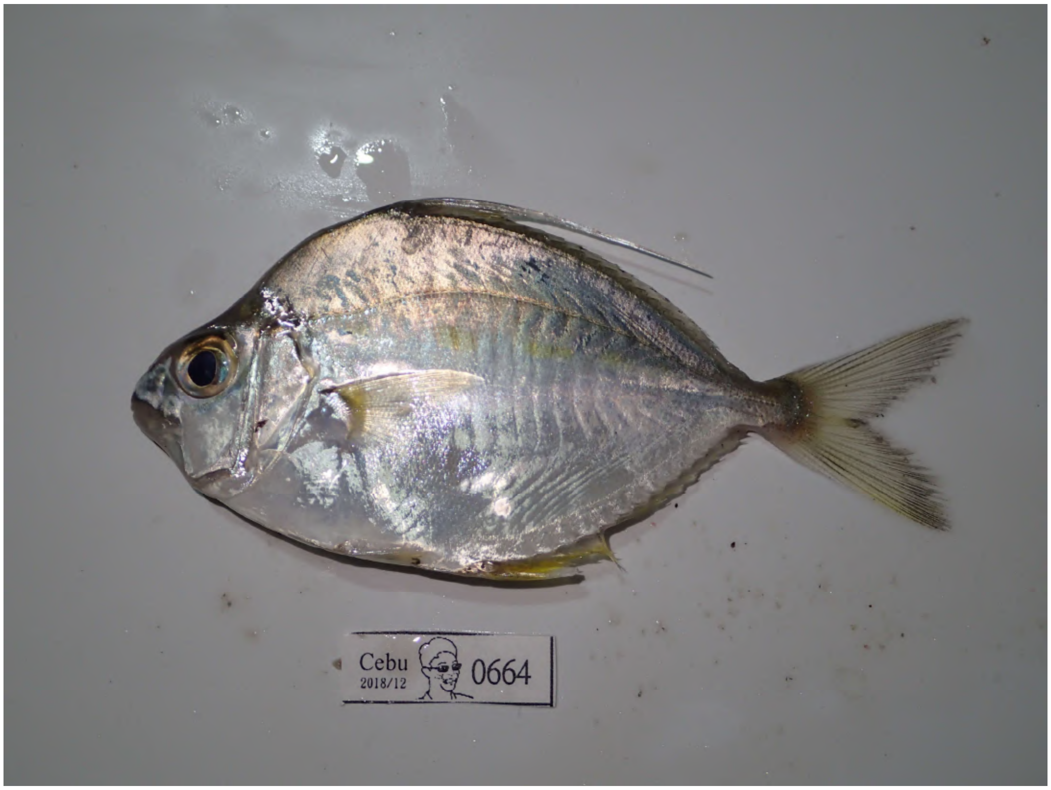

DOS 06765-1, *Aurigequula fasciata,* OR113950.

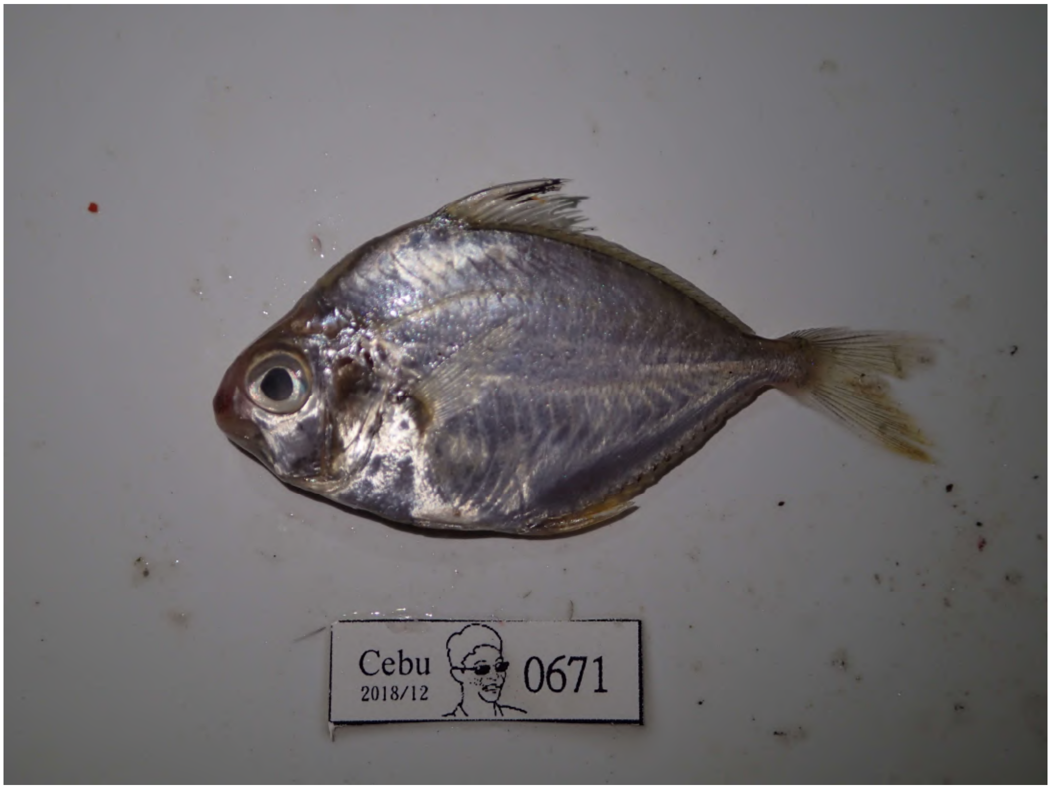

DOS 06766, *Eubleekeria* sp., OR113951.

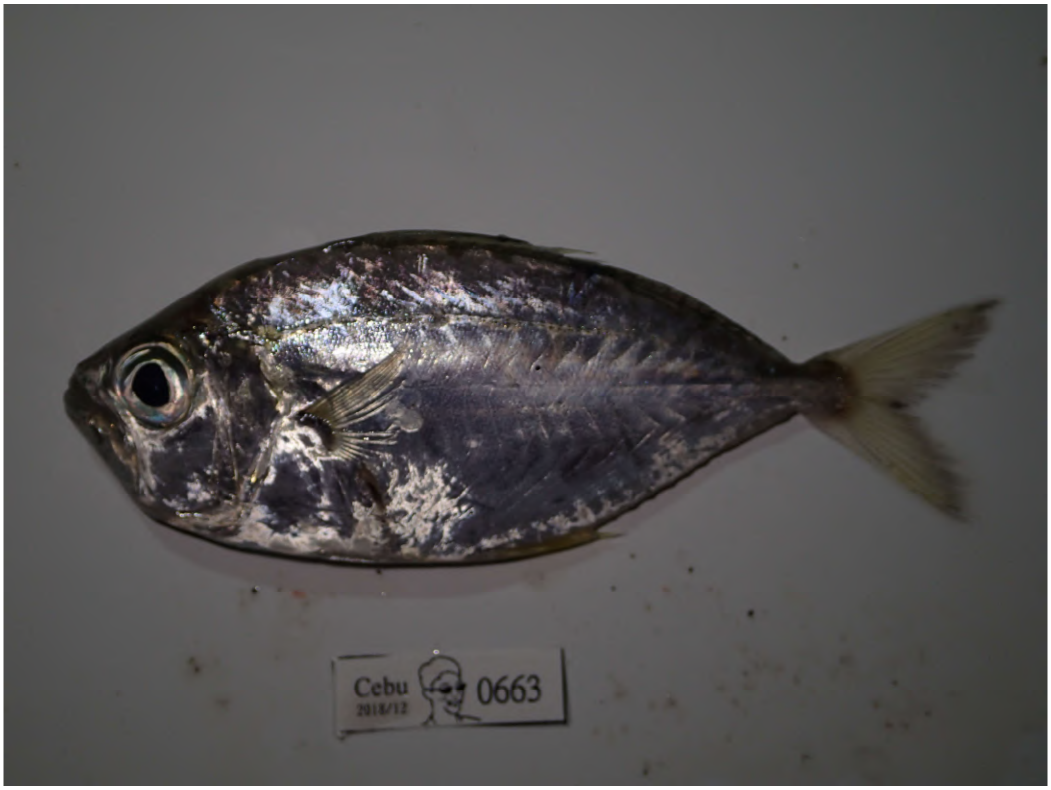

DOS 06768-1, *Gazza minuta*, OR113952.

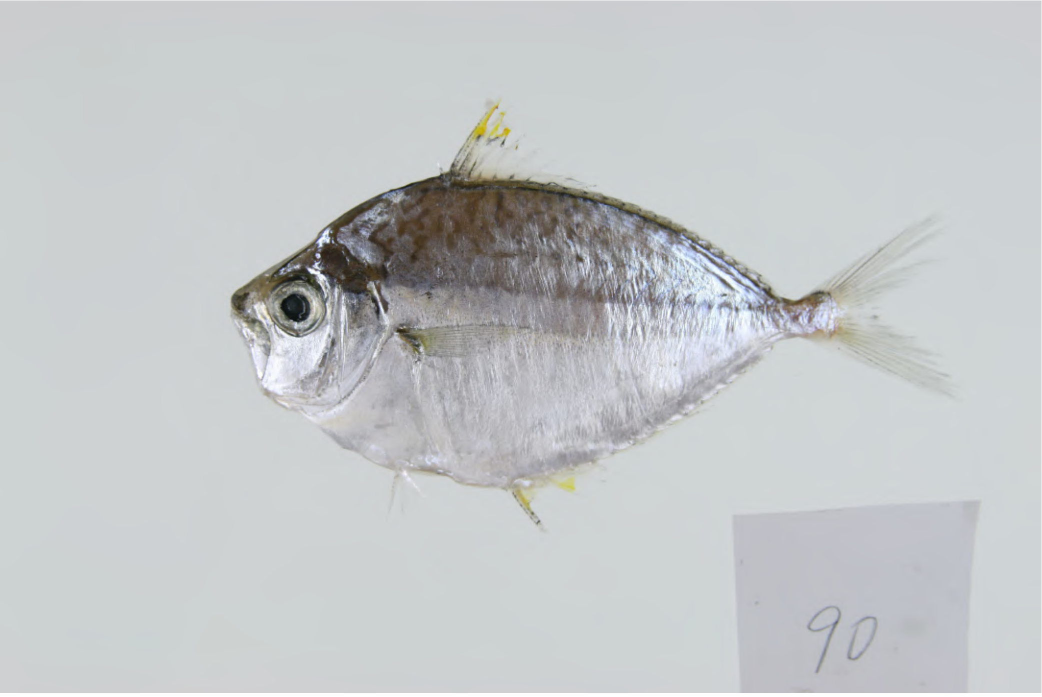

DOS 08655, *Photopectoralis bindus*, OR114231.

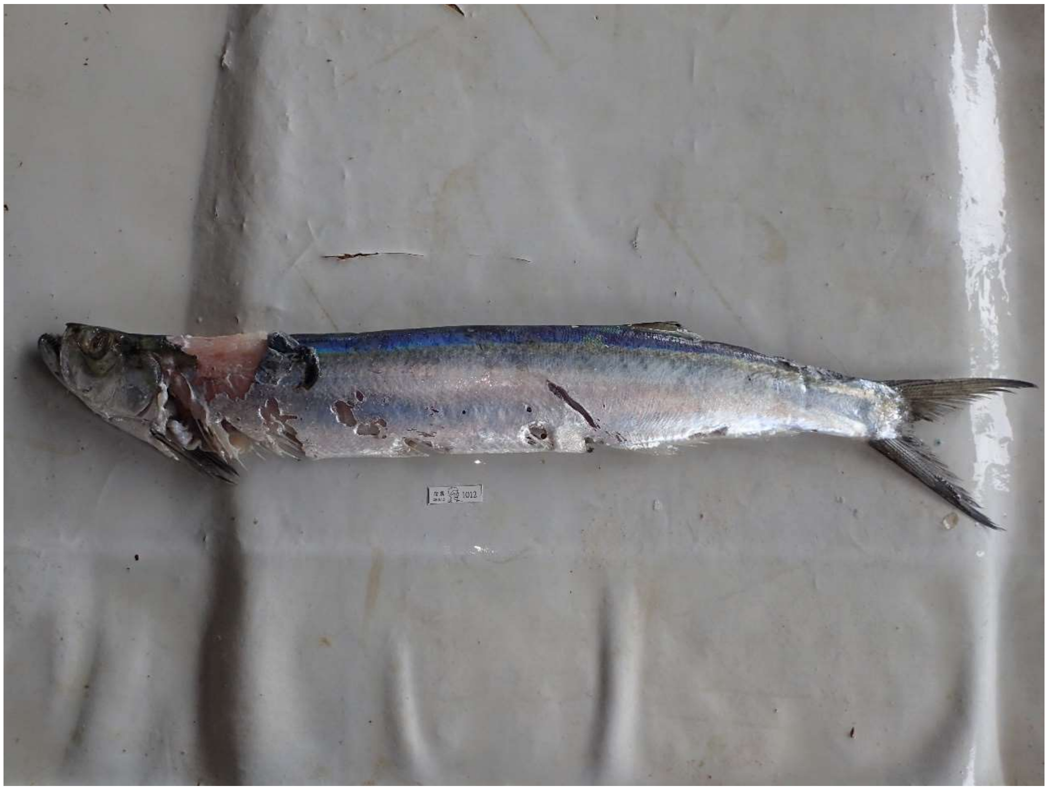

DOS 06673-1, *Chirocentrus dorab*, OR113864. (specimen not preserved)

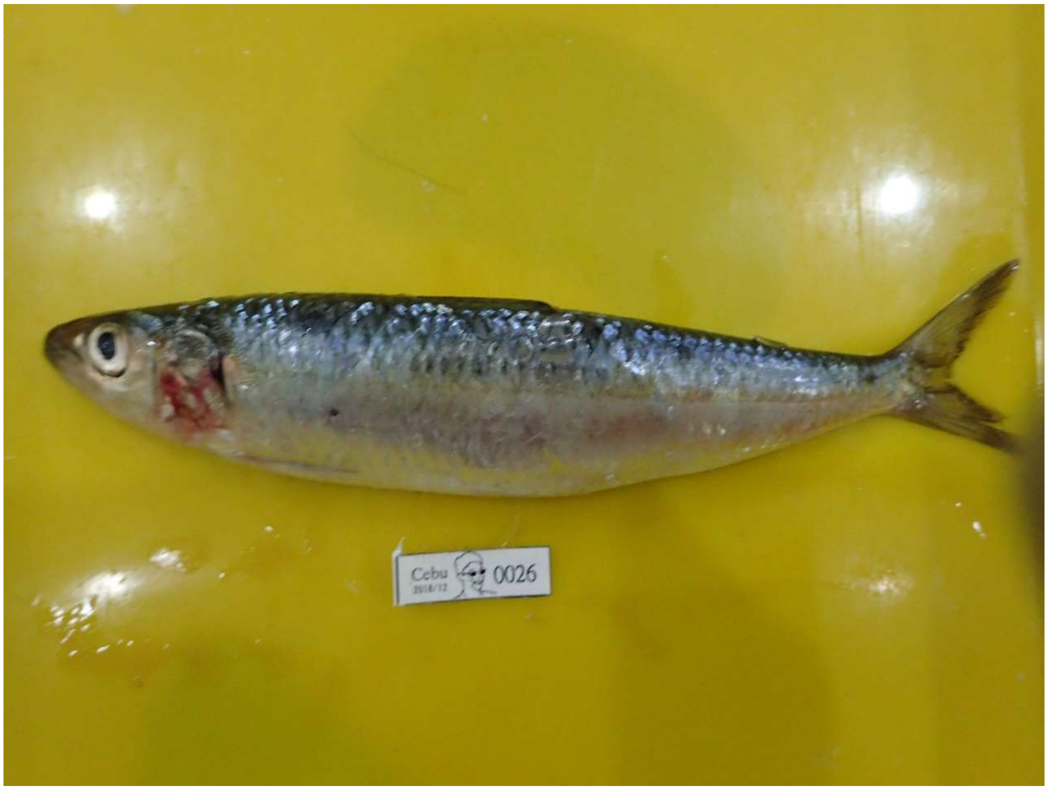

DOS 06675, *Sardinella lemuru*, OR113866.

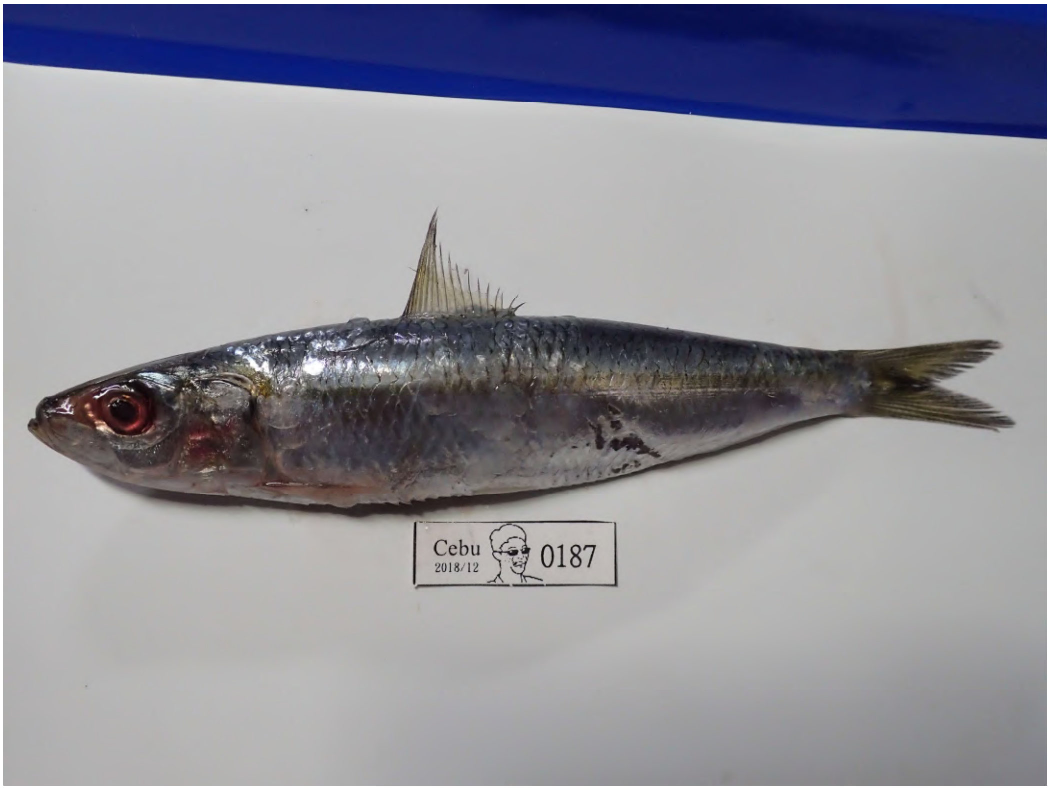

DOS 06676-1, *Sardinella lemuru*, OR113867.

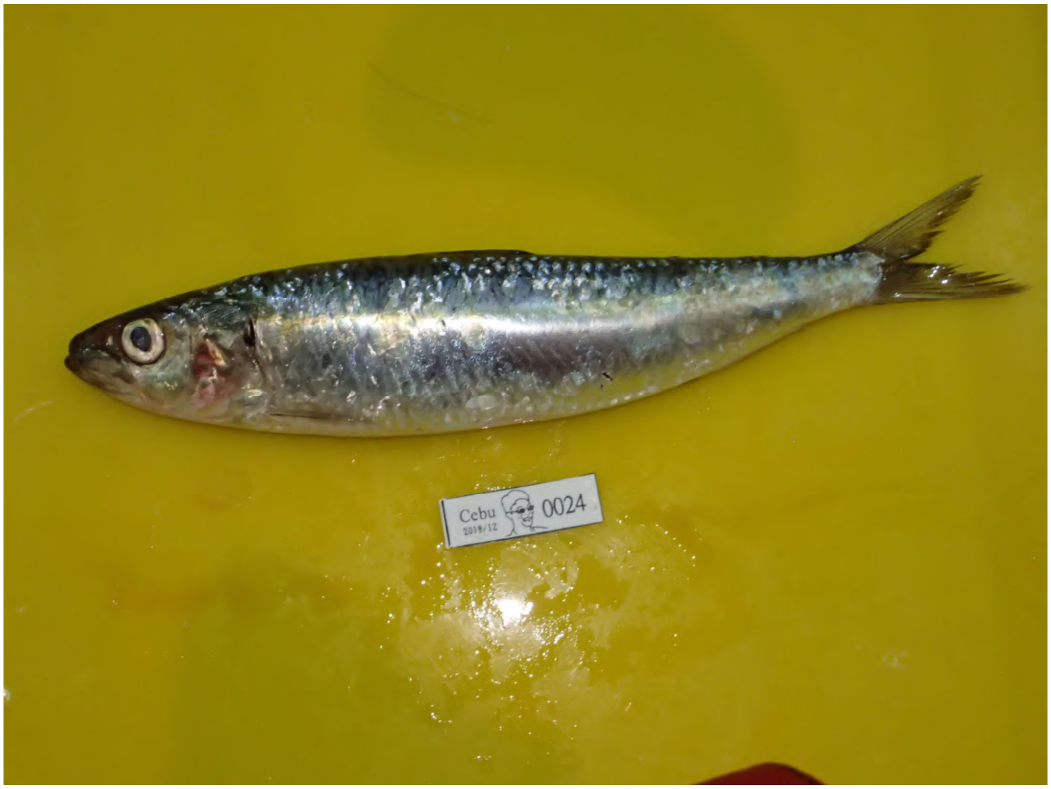

DOS 06677, *Sardinella lemuru*, OR113868.

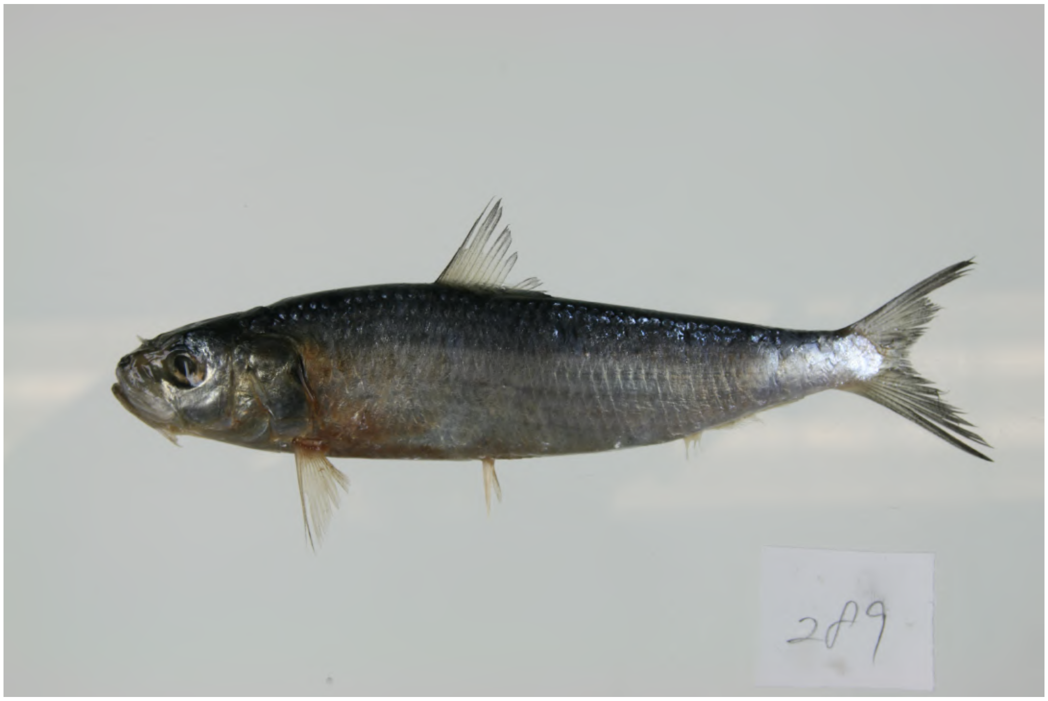

DOS 08640, *Sardinella lemuru*, OR114212.

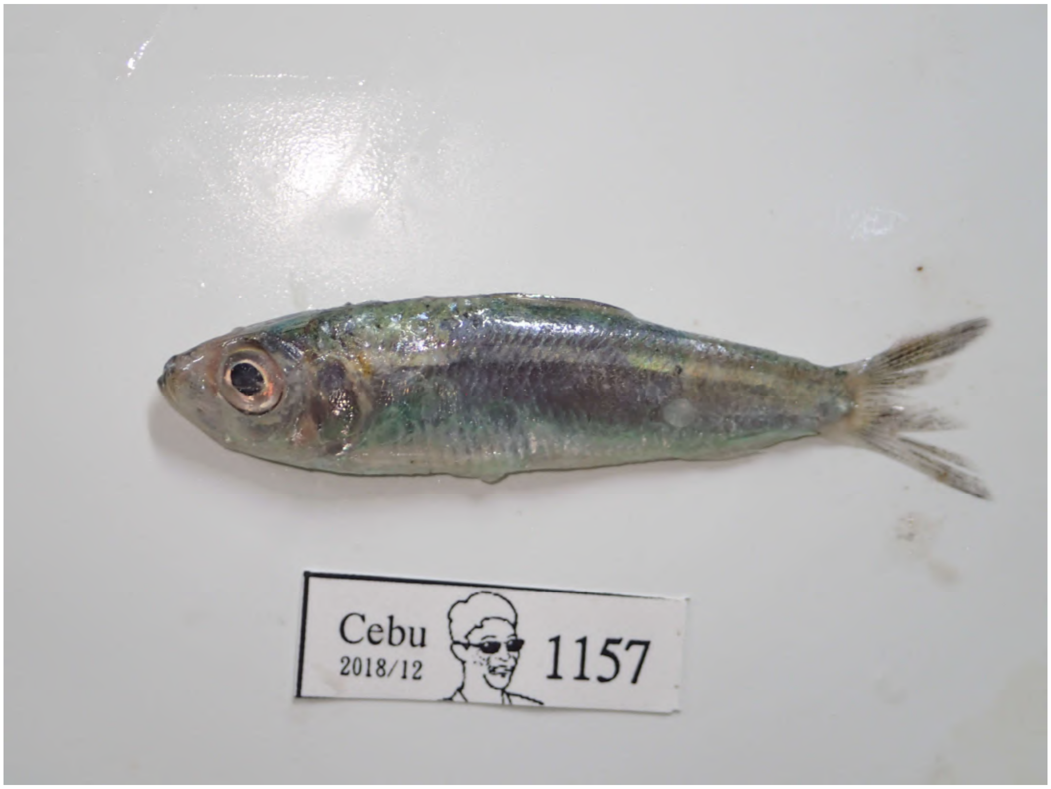

DOS 06690-3, *Stolephorus commersonnii*, OR113880.

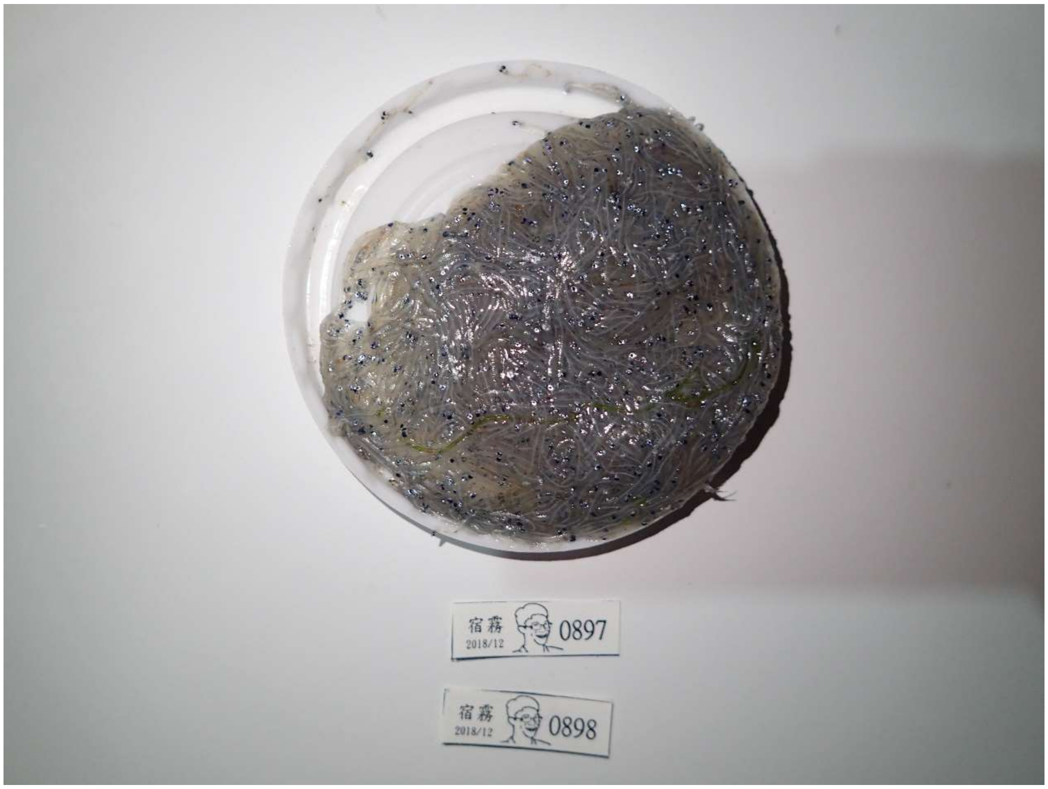

DOS 06994, *Spratelloides* cf. *delicatulus*, OR114163.

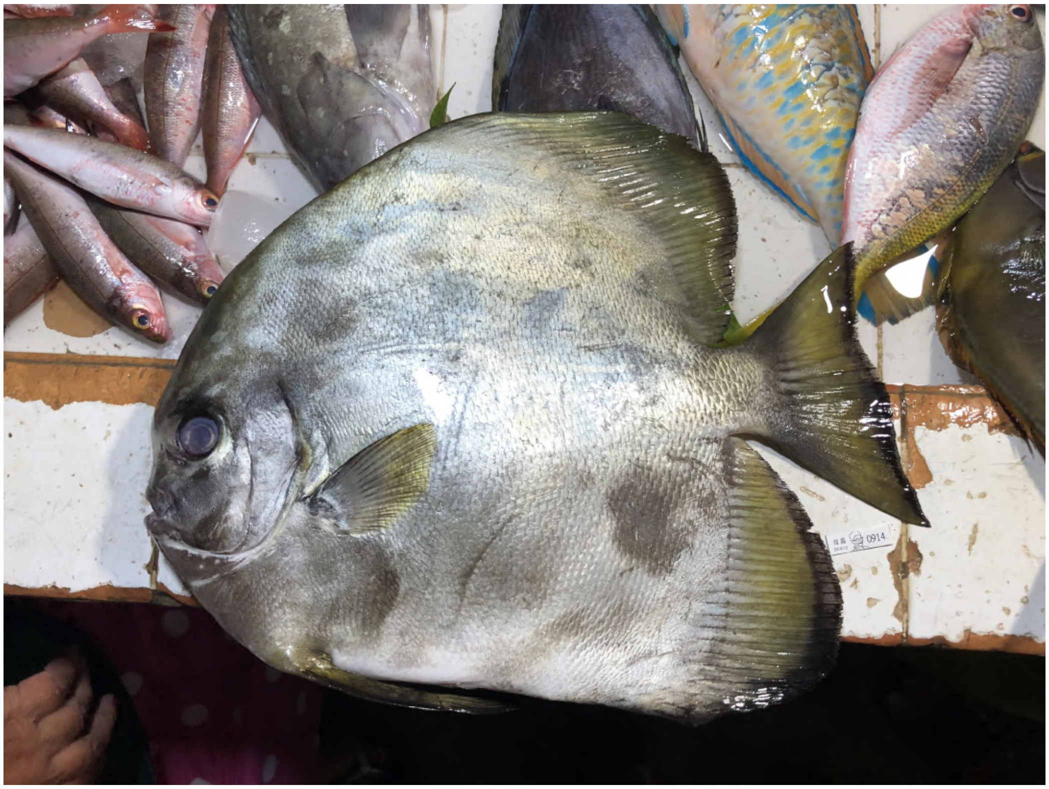

DOS 06691, *Platax boersii*, OR113881. (specimen not preserved)

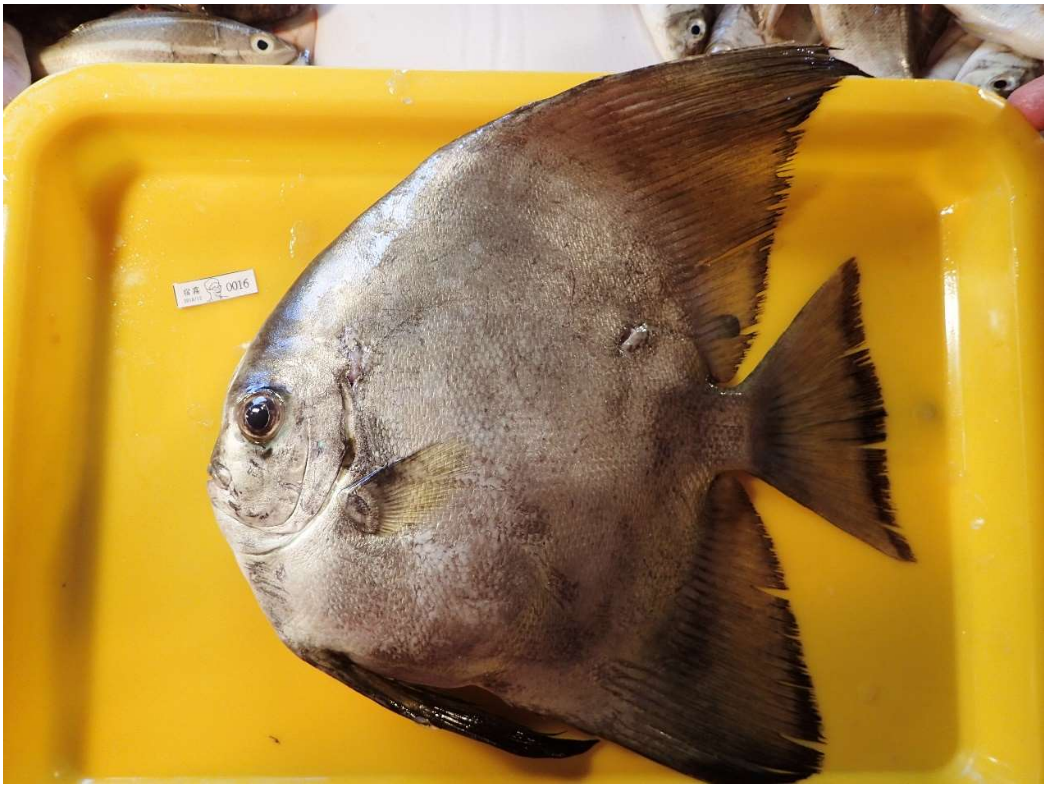

DOS 06692, *Platax boersii*, OR113882. (specimen not preserved)

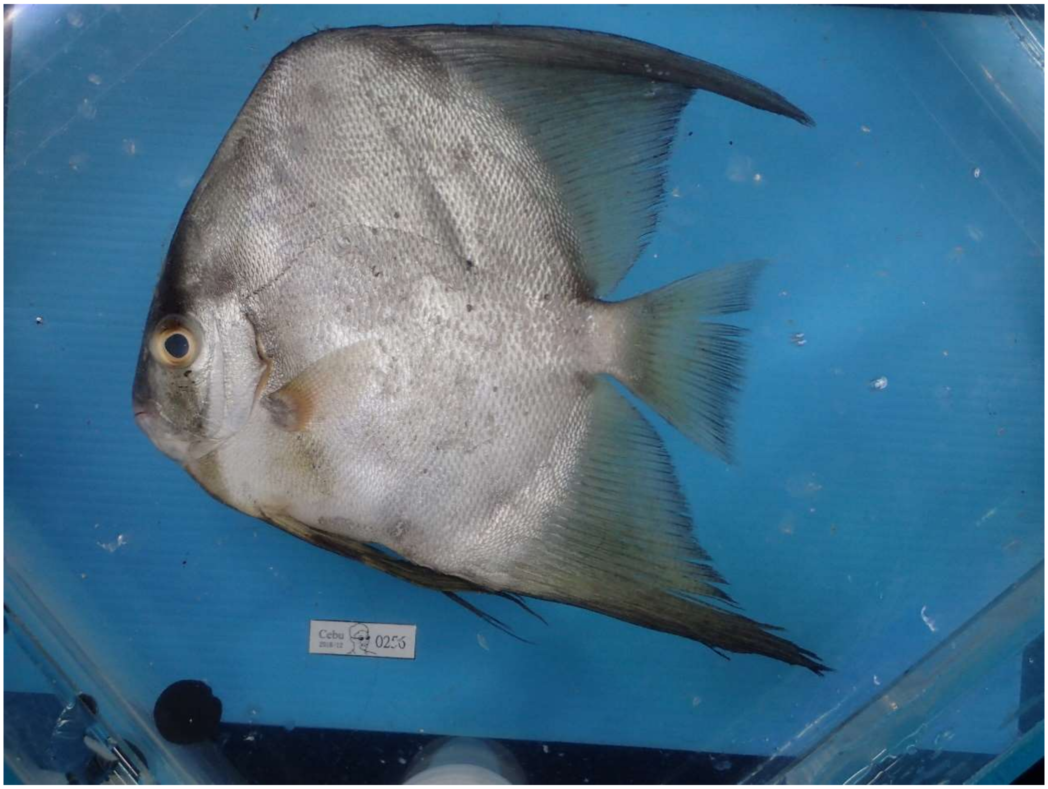

DOS 06693-1, *Platax orbicularis*, OR113883.

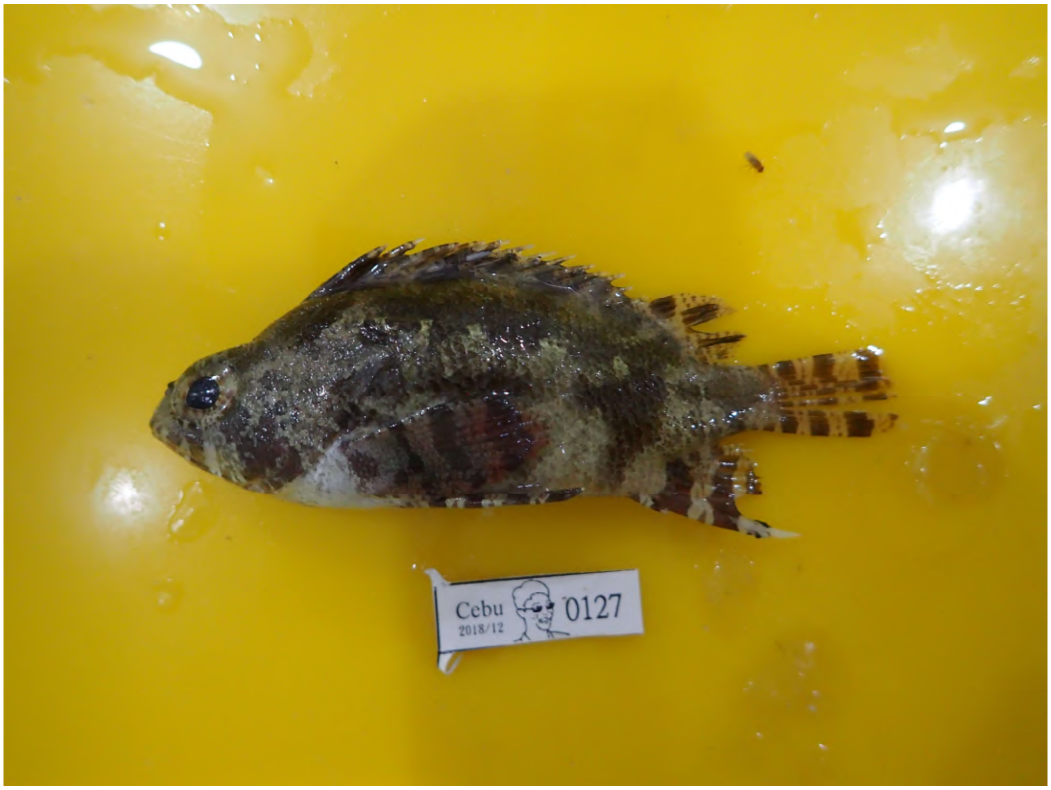

DOS 06655-1, *Centrogenys vaigiensis*, OR113846. (specimen not preserved)

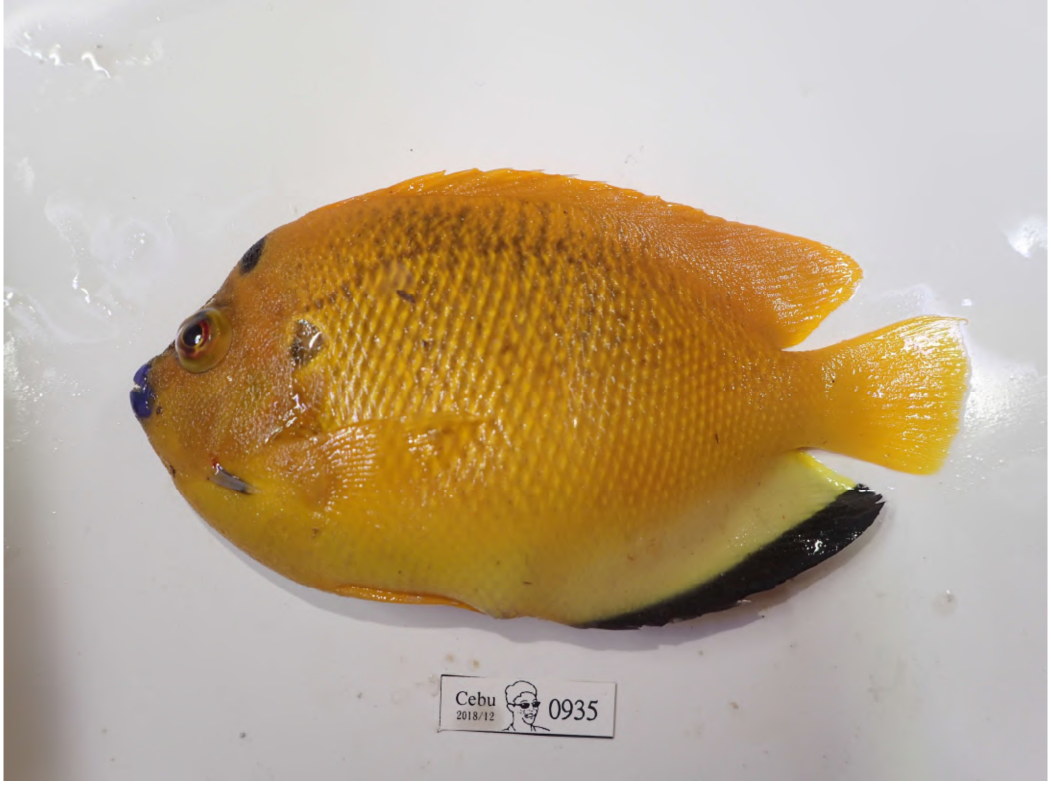

DOS 06874, *Apolemichthys trimaculatus*, OR114051.

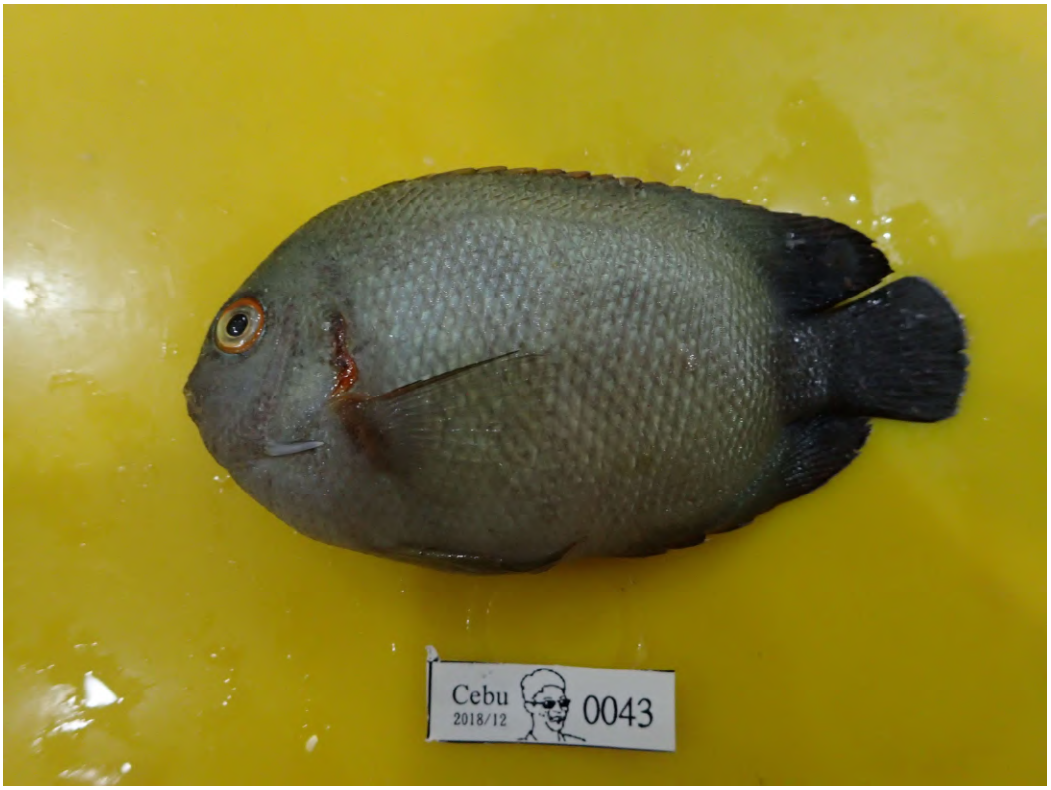

DOS 06875-1, *Centropyge vrolikii*, OR114052. (specimen not preserved)

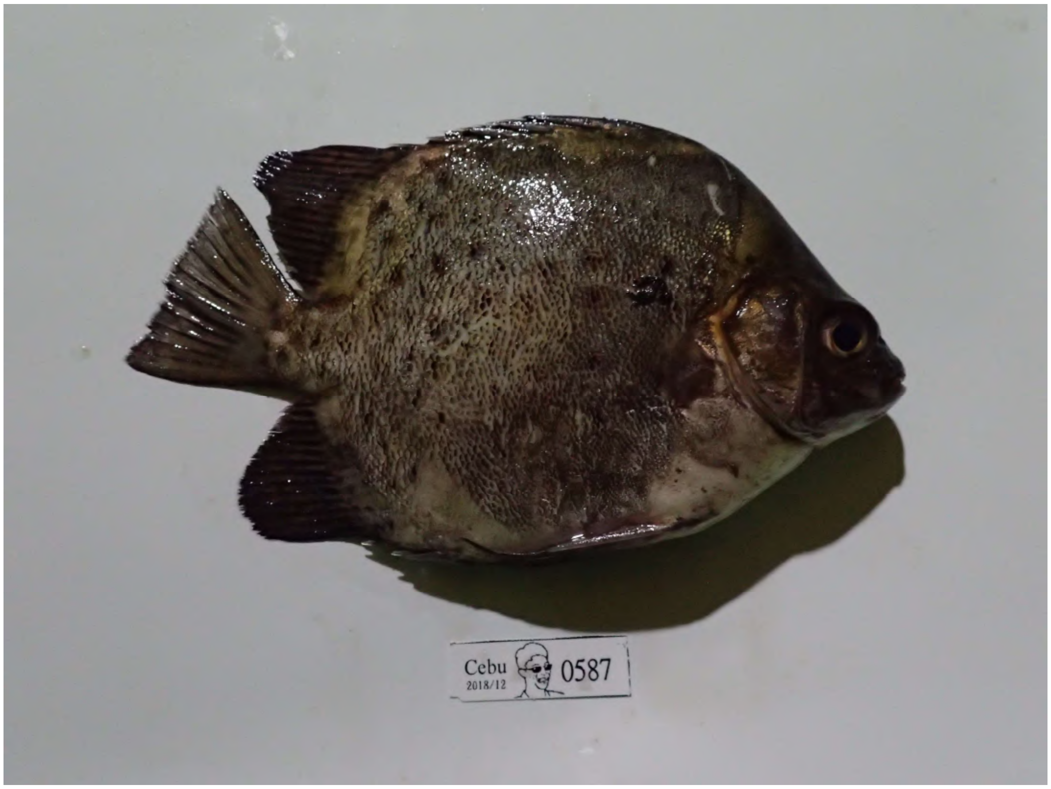

DOS 06923-1, *Scatophagus argus*, OR114099.

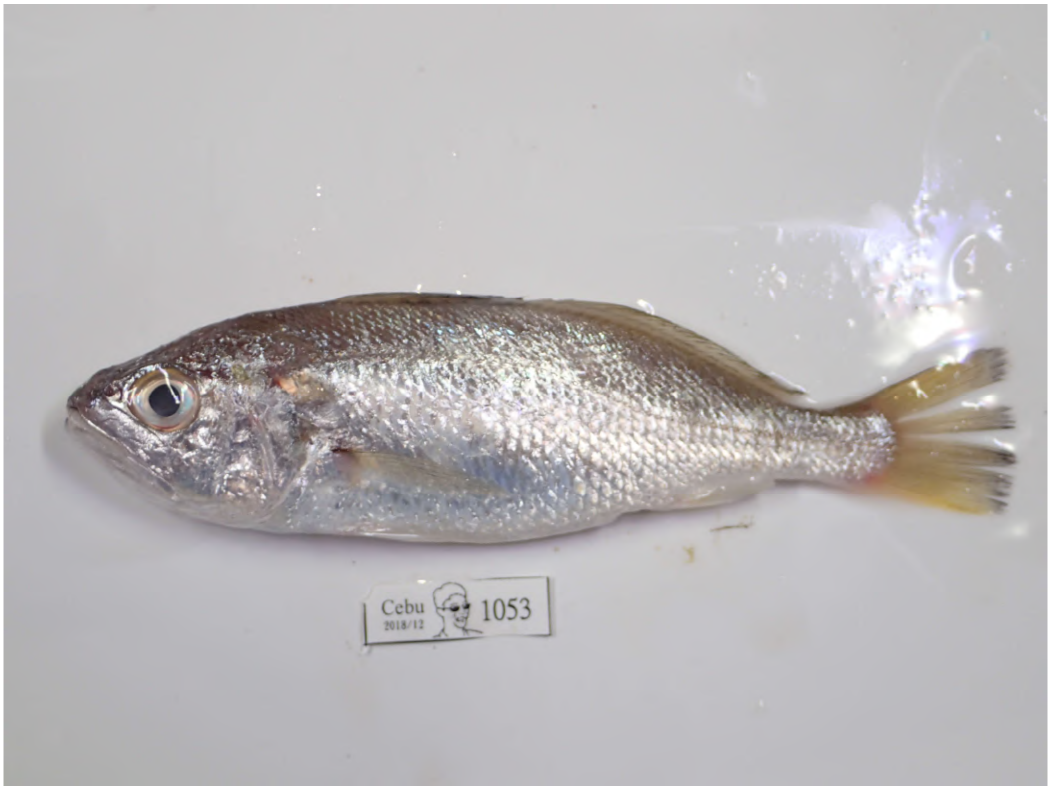

DOS 06924-3, *Pennahia aneus*, OR114100.

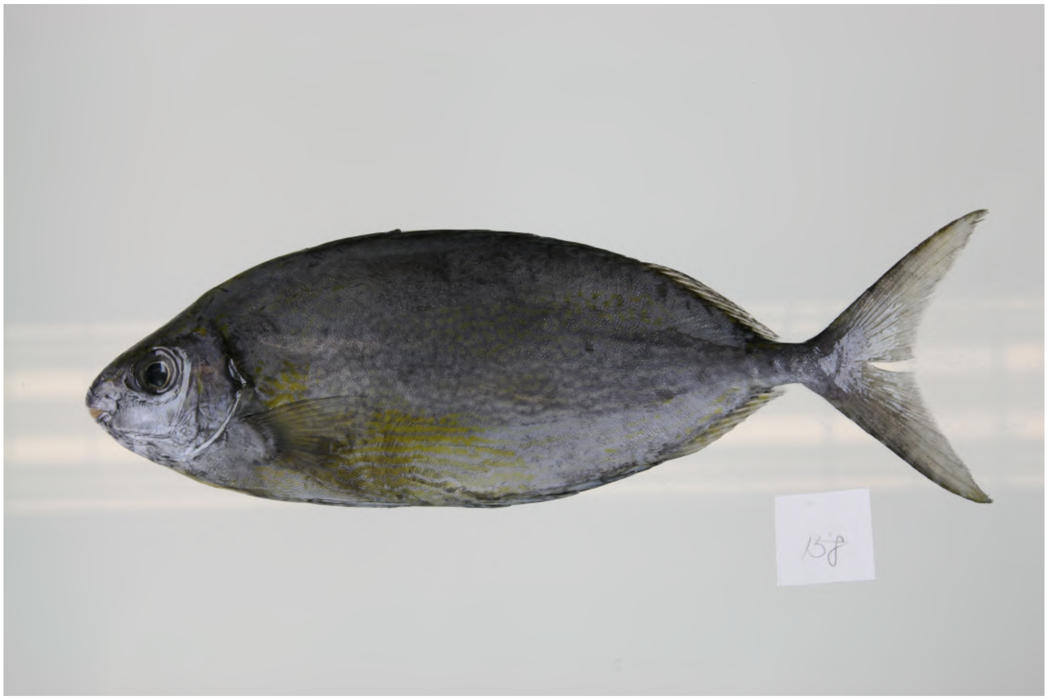

DOS 08664, *Siganus argenteus*, OR114240.

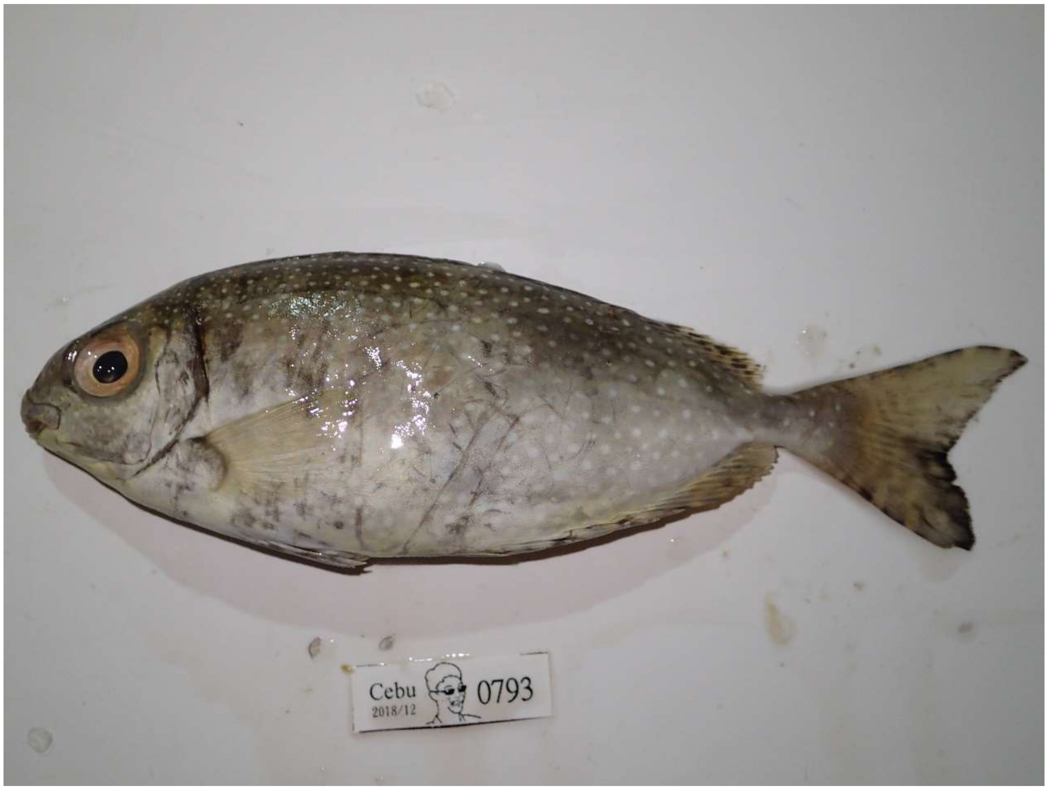

DOS 06957-1, *Siganus fuscescens*, OR114133.

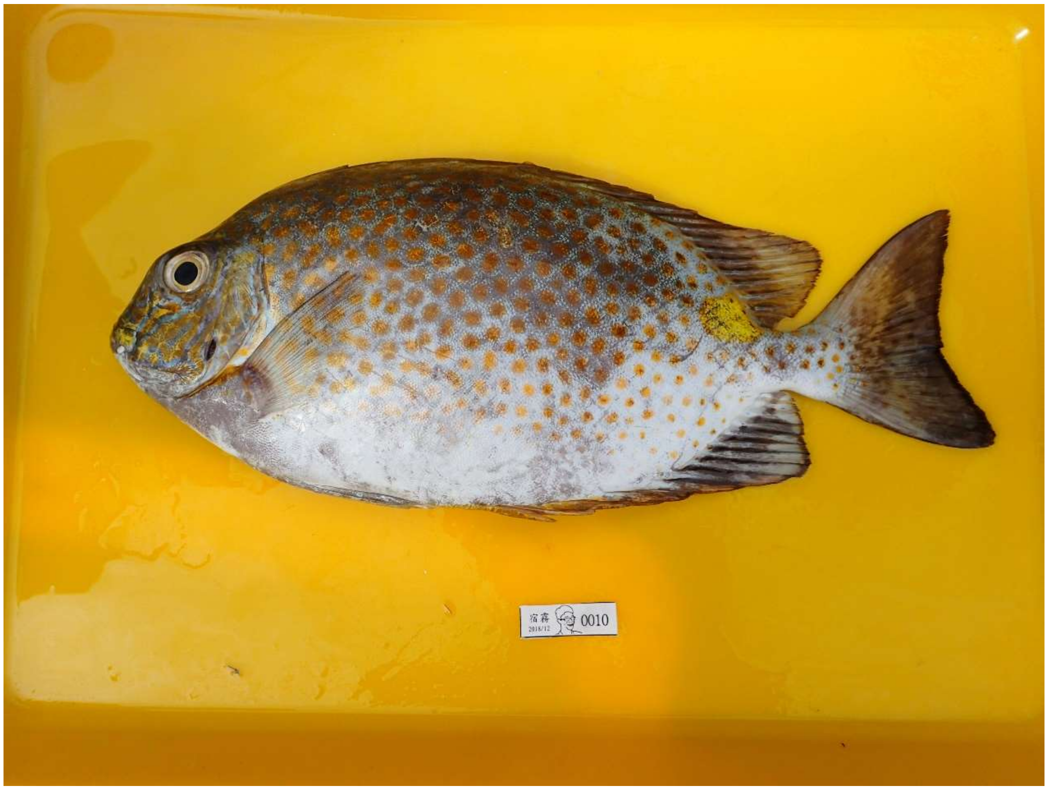

DOS 06958-1, *Siganus guttatus*, OR114134. (specimen not preserved)

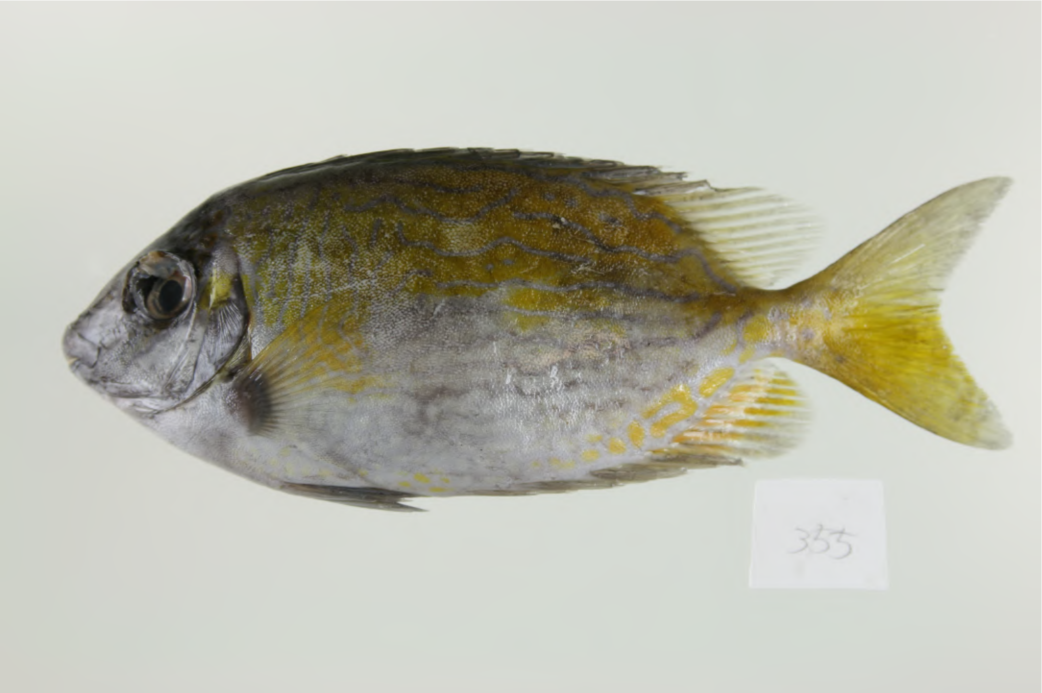

DOS 08665, *Siganus puellus*, OR114241.

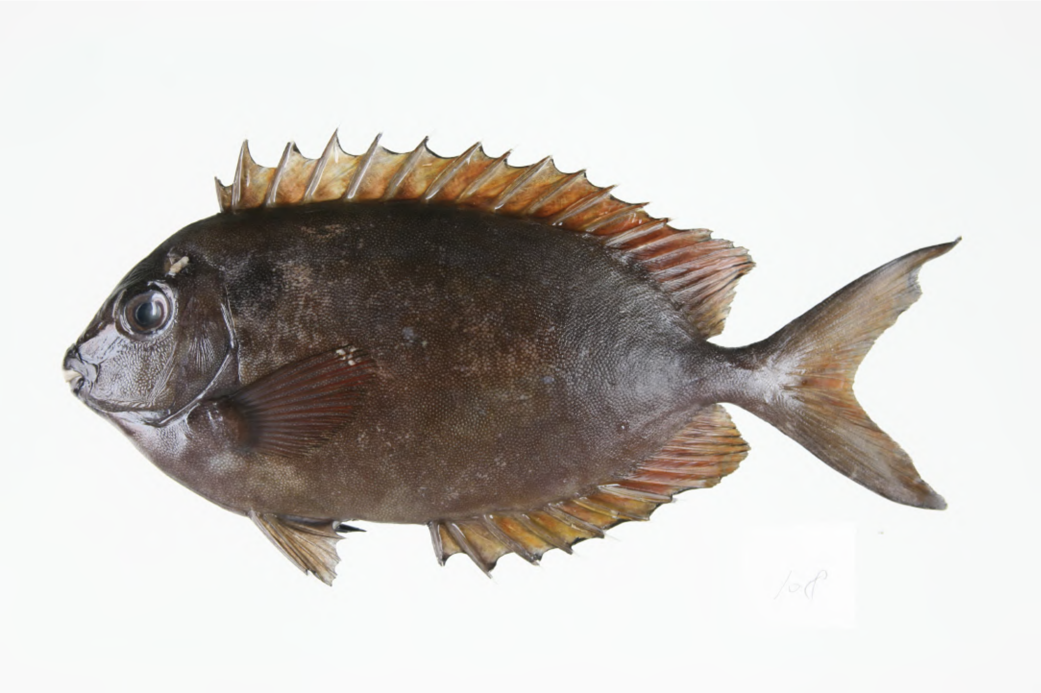

DOS 08666-1, *Siganus punctatissimus*, OR114242.

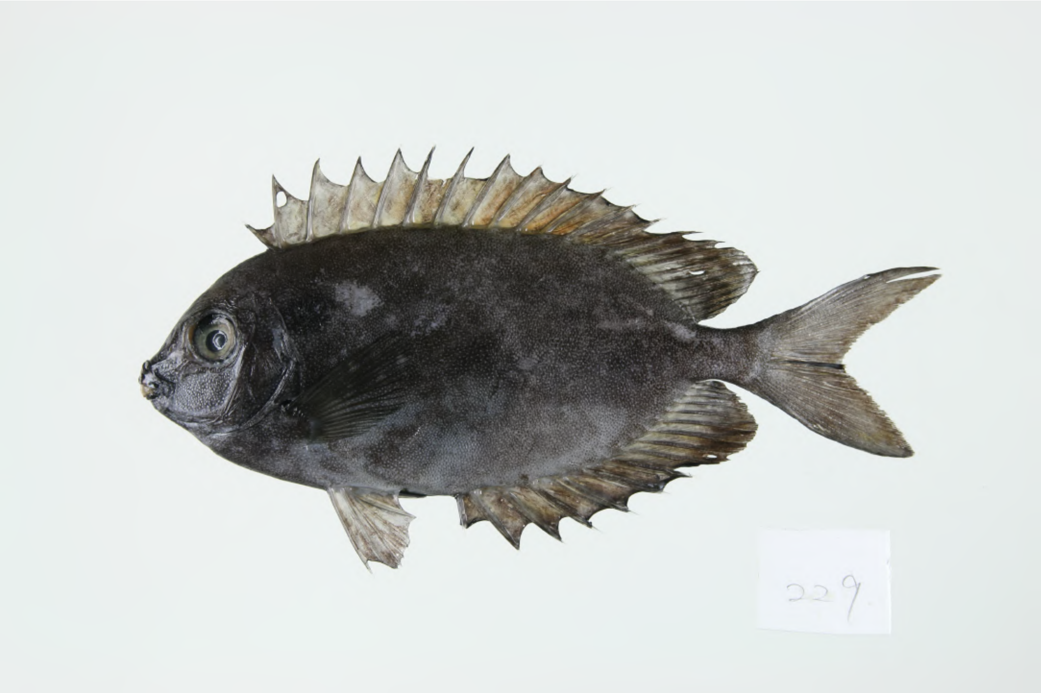

DOS 08666-2, *Siganus punctatissimus*, OR114243.

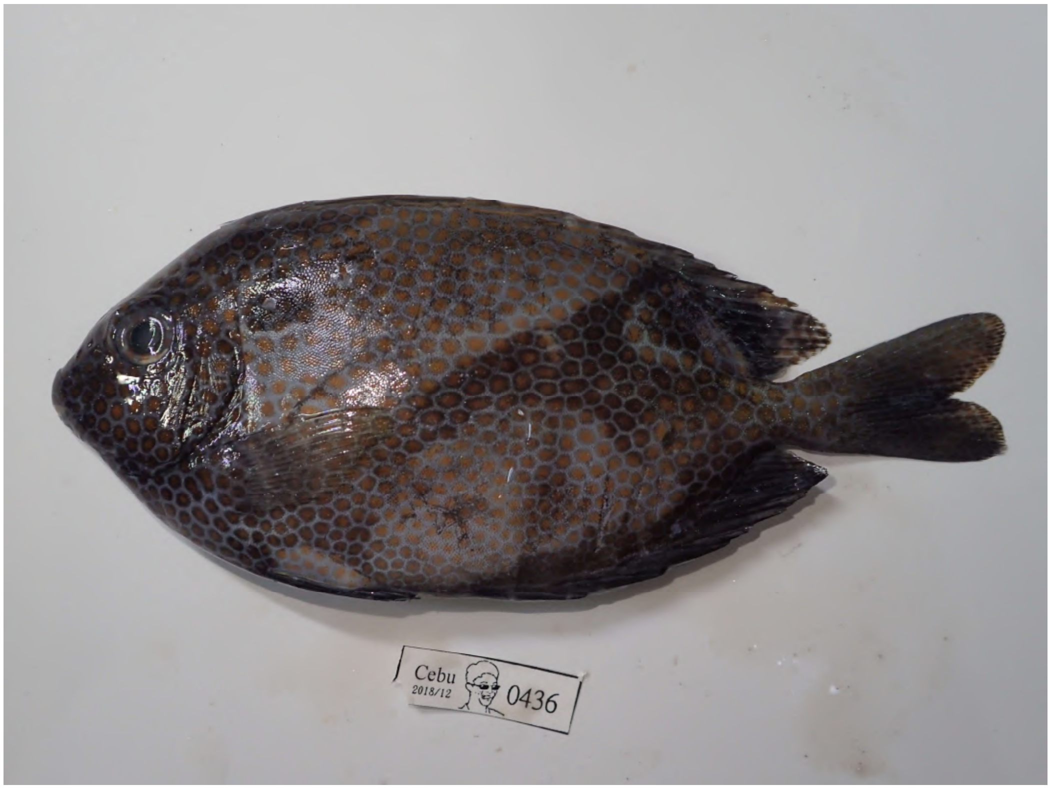

DOS 06959-2, *Siganus punctatus*, OR114135.

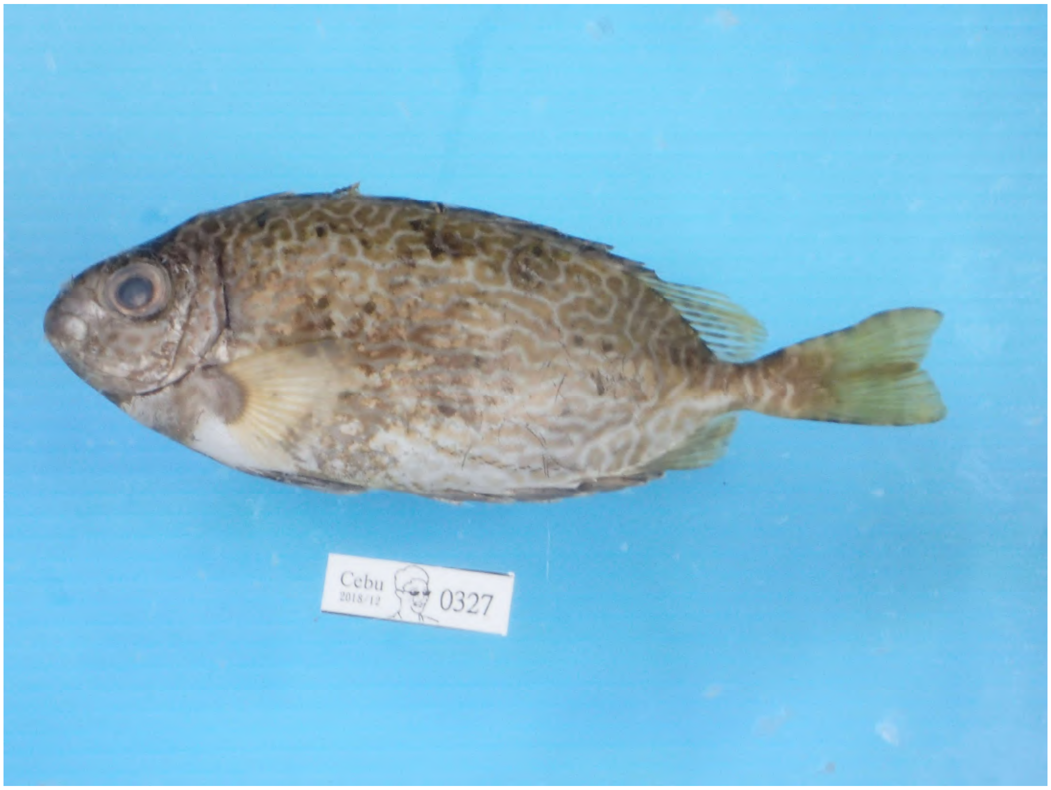

DOS 06961-1, *Siganus spinus*, OR114137. (specimen not preserved)

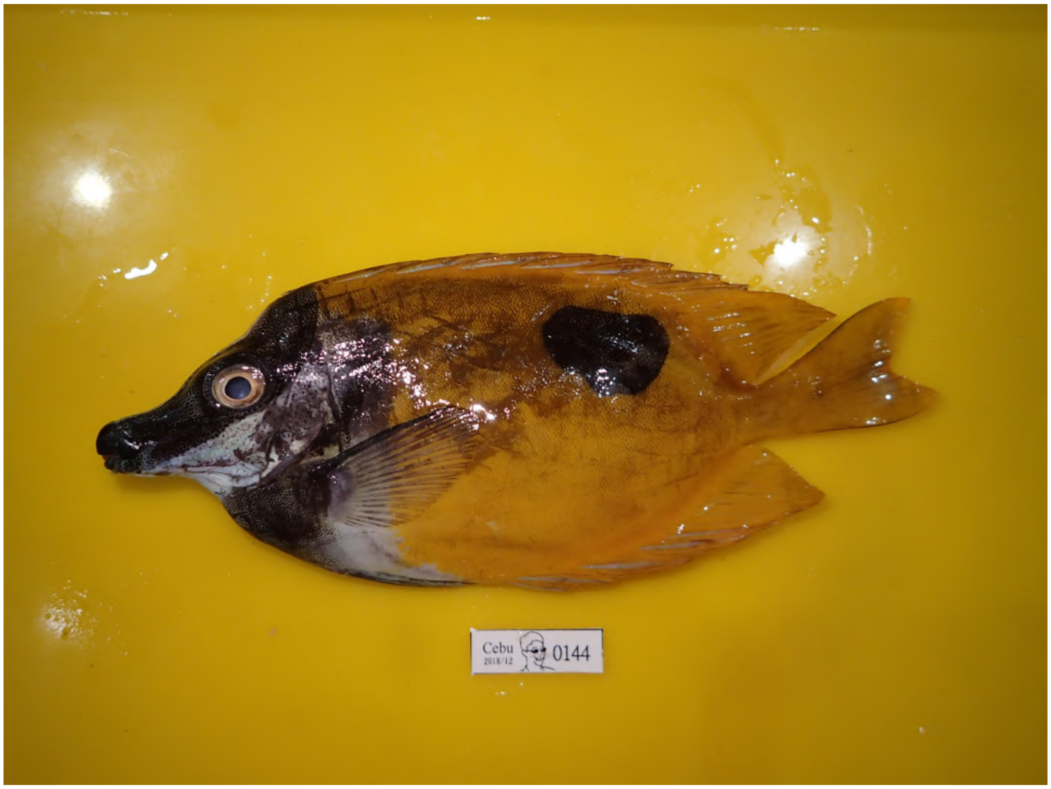

DOS 06960-2, *Siganus unimaculatus*, OR114136. (specimen not preserved)

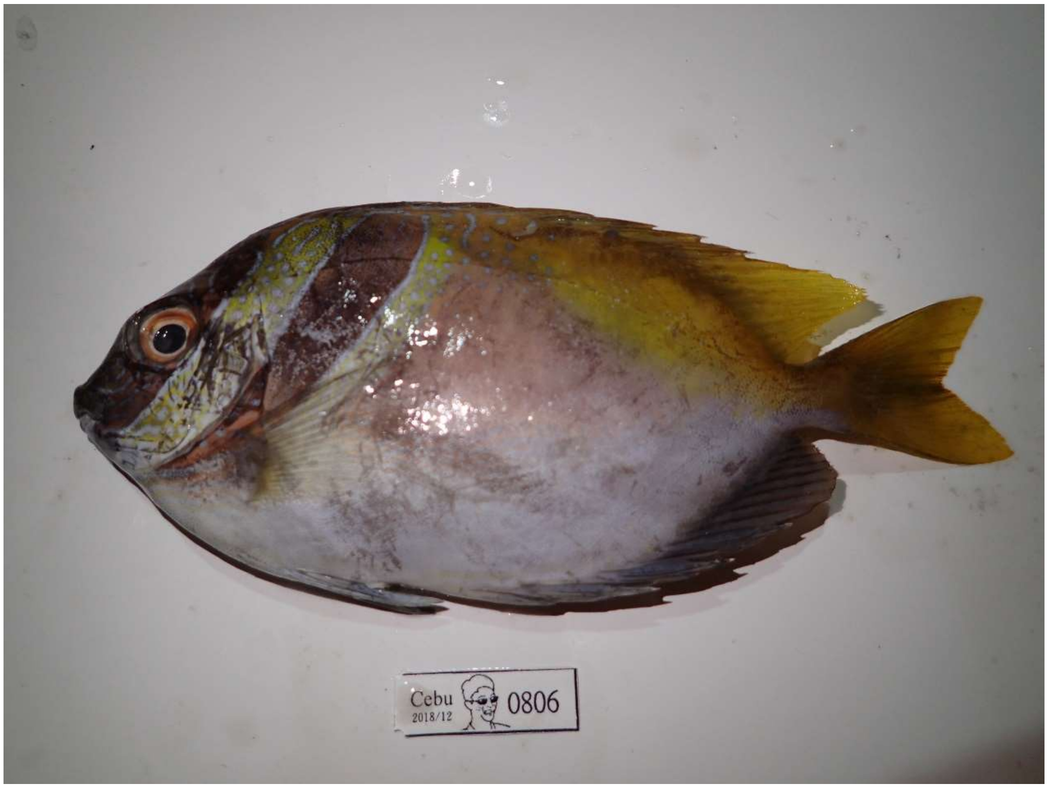

DOS 06962-3, *Siganus virgatus*, OR114138.

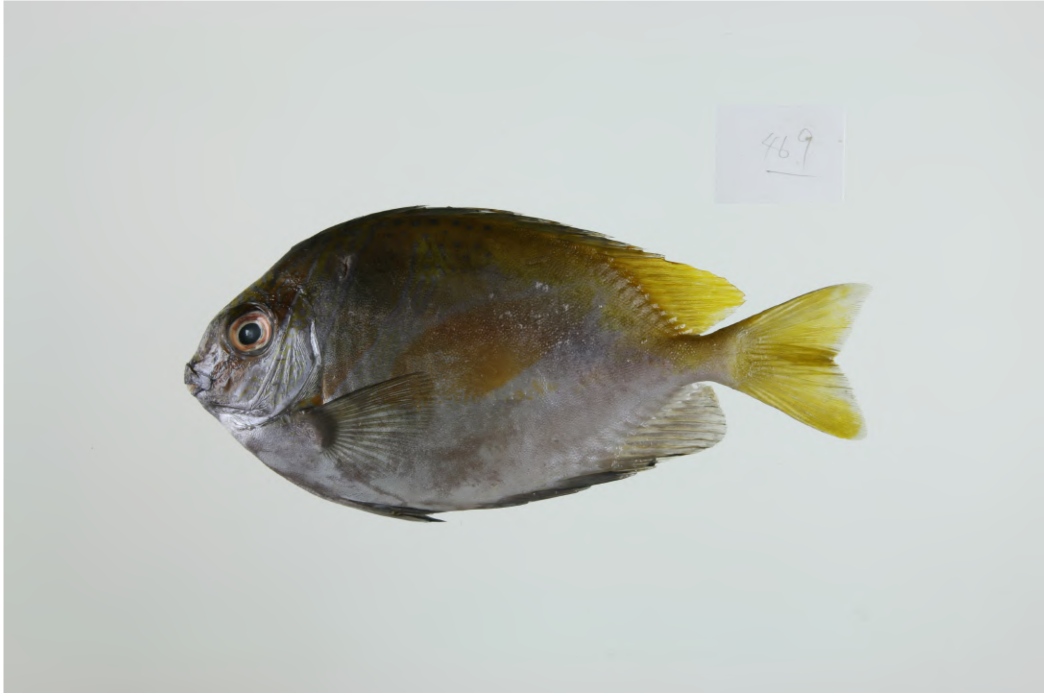

DOS 08667, *Siganus virgatus*, OR114244.

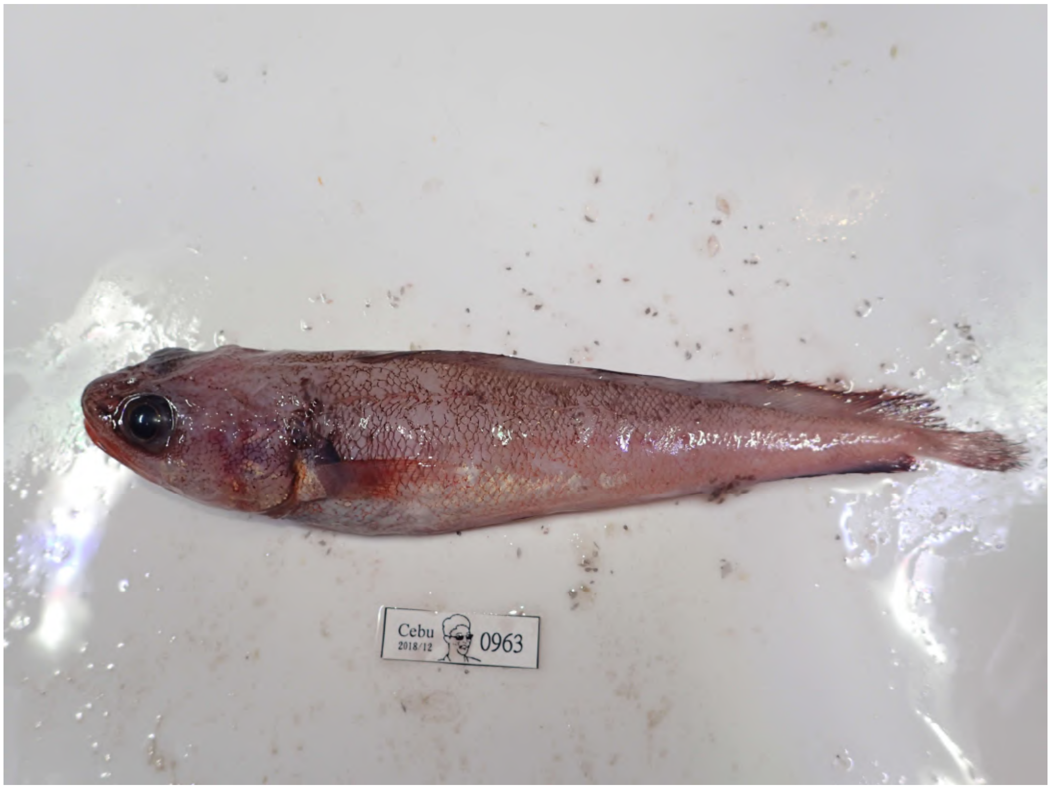

DOS 06804-1, *Physiculus chigodarana*, OR113986.

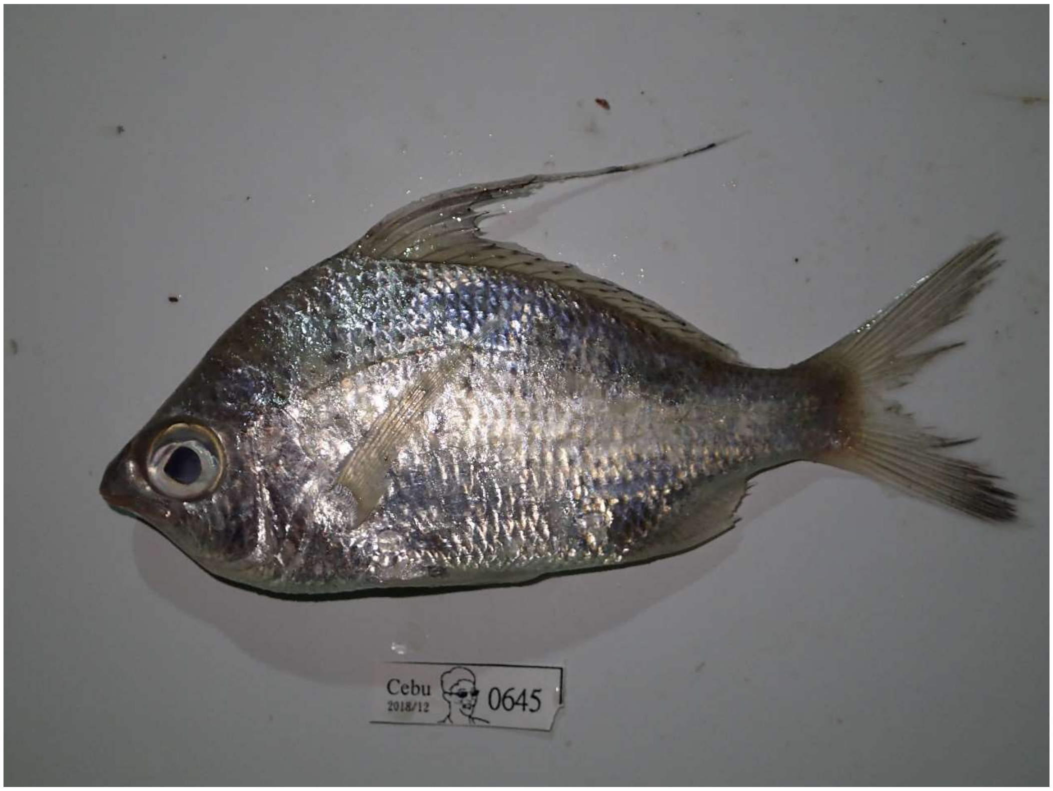

DOS 06702-1, *Gerres filamentosus*, OR113890.

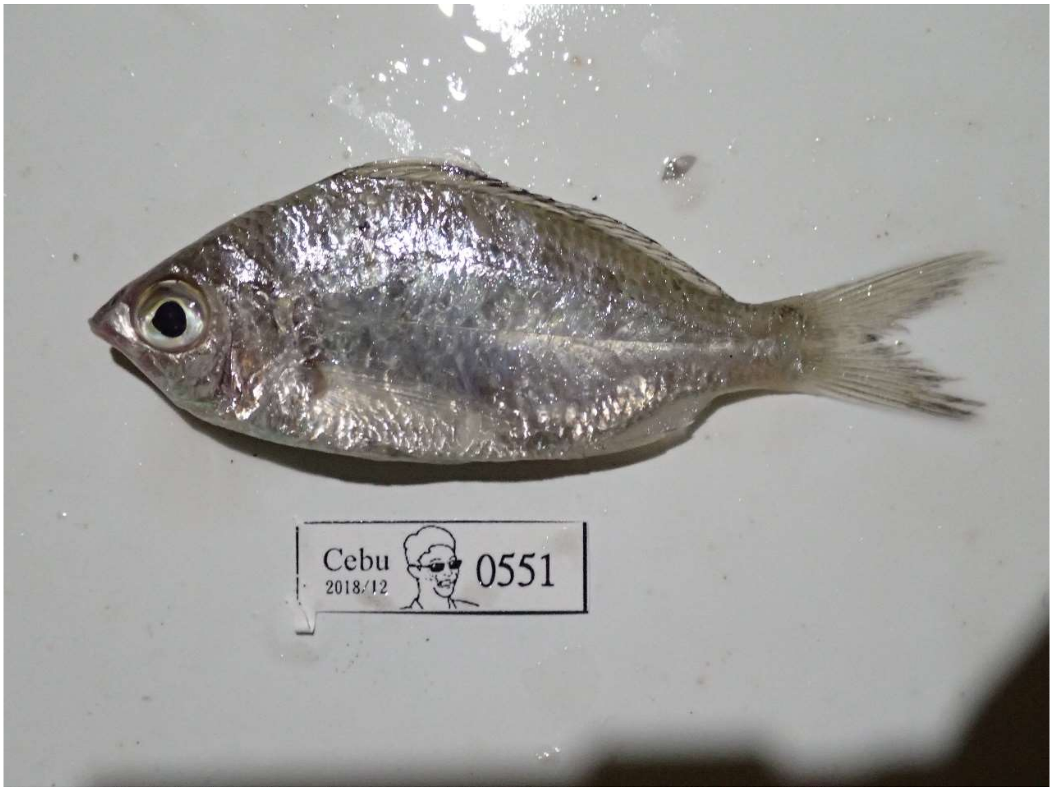

DOS 06704-1, *Gerres macracanthus*, OR113892.

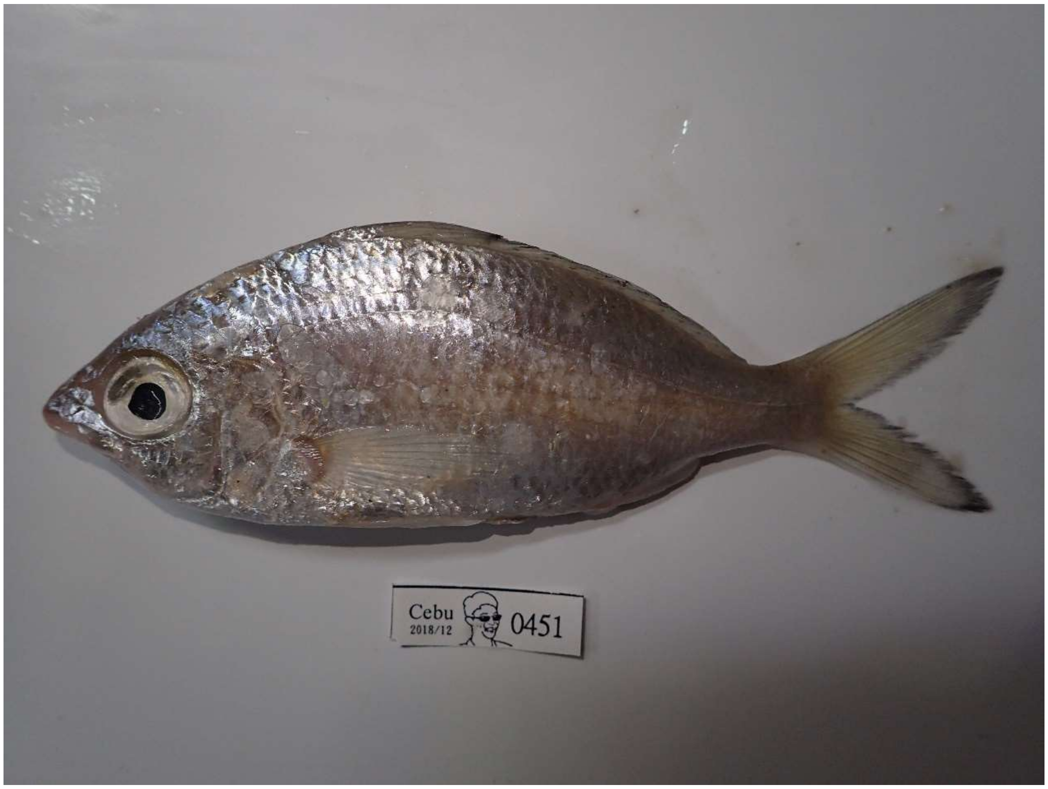

DOS 06701-1, *Gerres oyena*, OR113889.

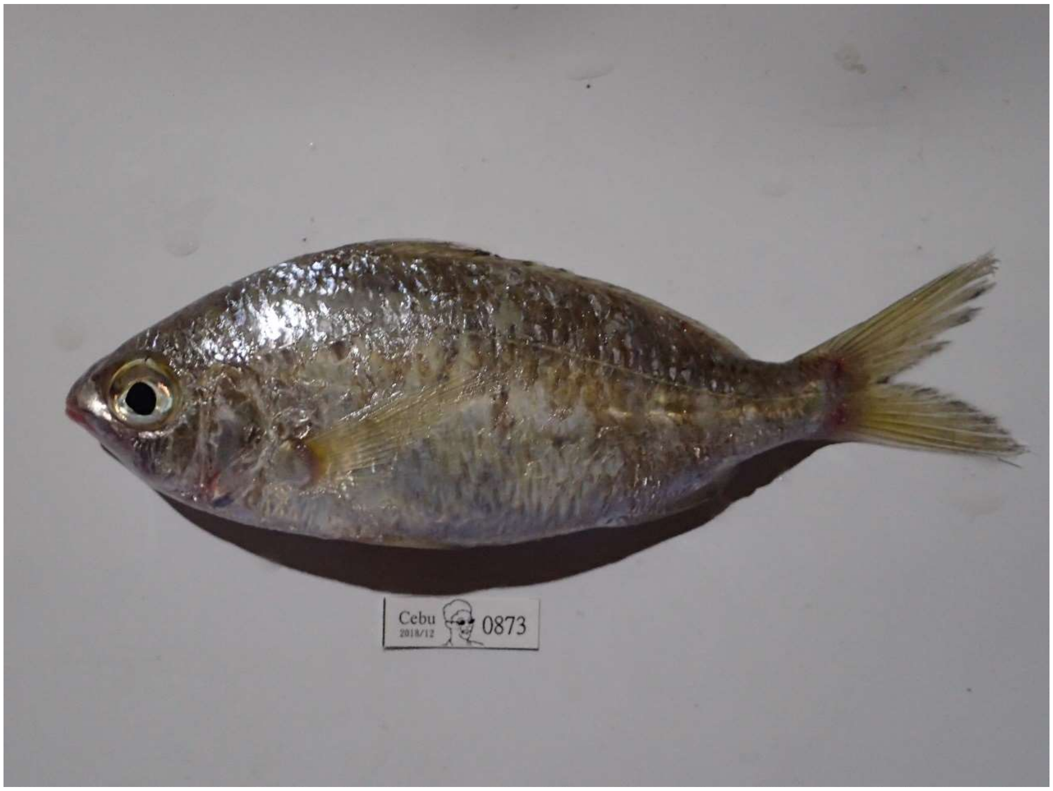

DOS 06703, *Gerres oyena*, OR113891.

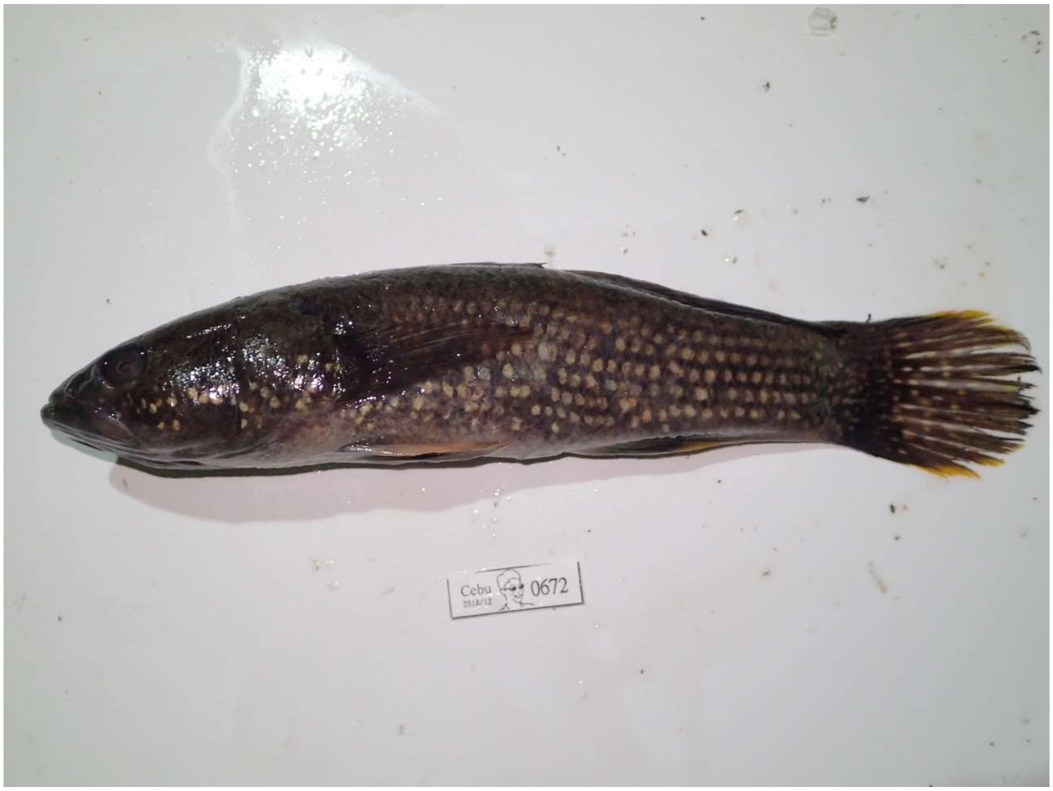

DOS 06689-1, *Ophiocara porocephala*, OR113879. (specimen not preserved)

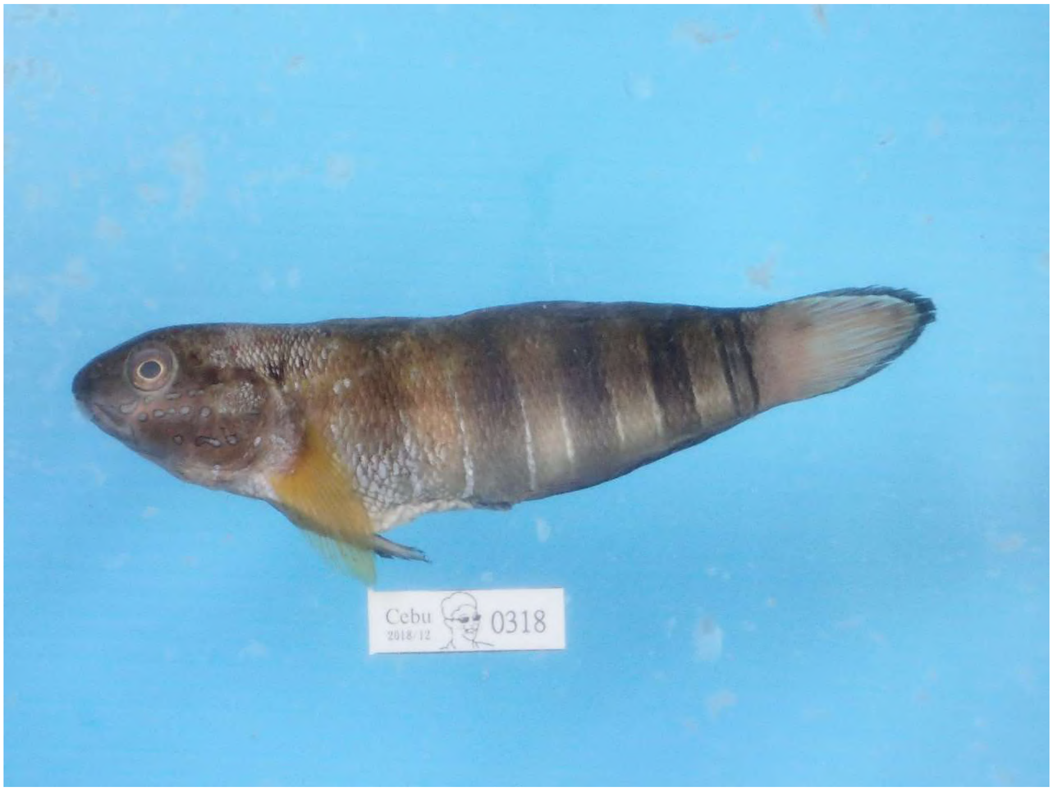

DOS 06705-1, *Amblygobius phalaena*, OR113893.

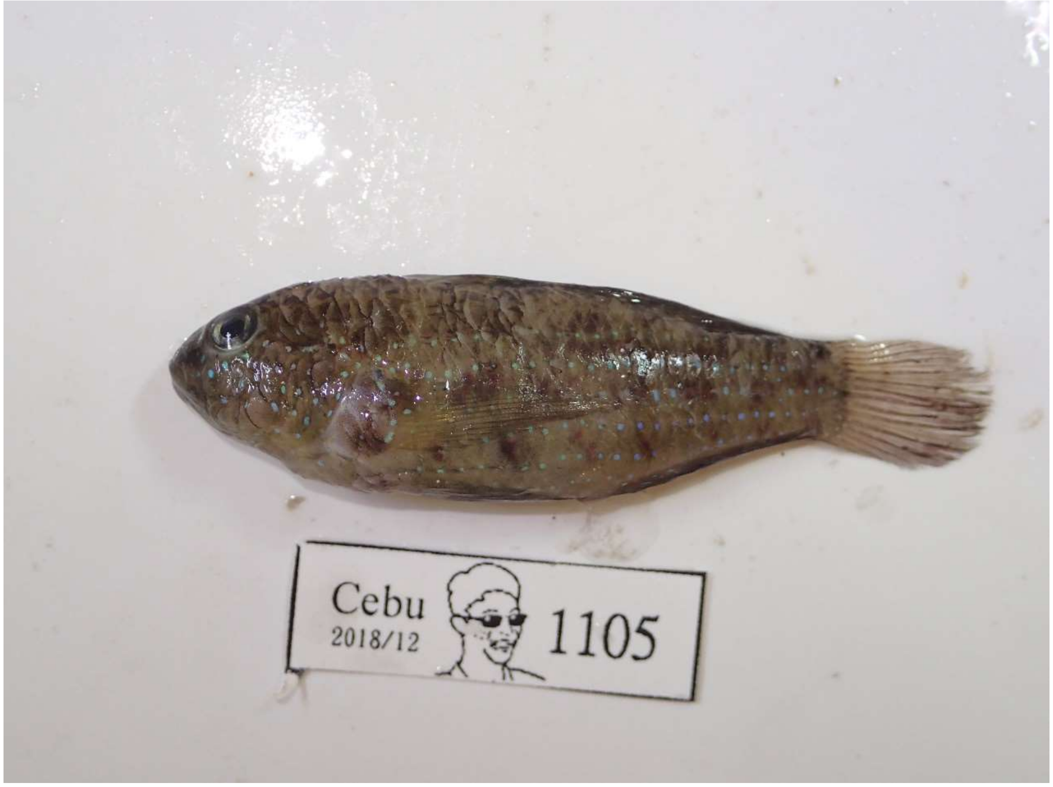

DOS 06914, *Asterropteryx semipunctata*, OR114091.

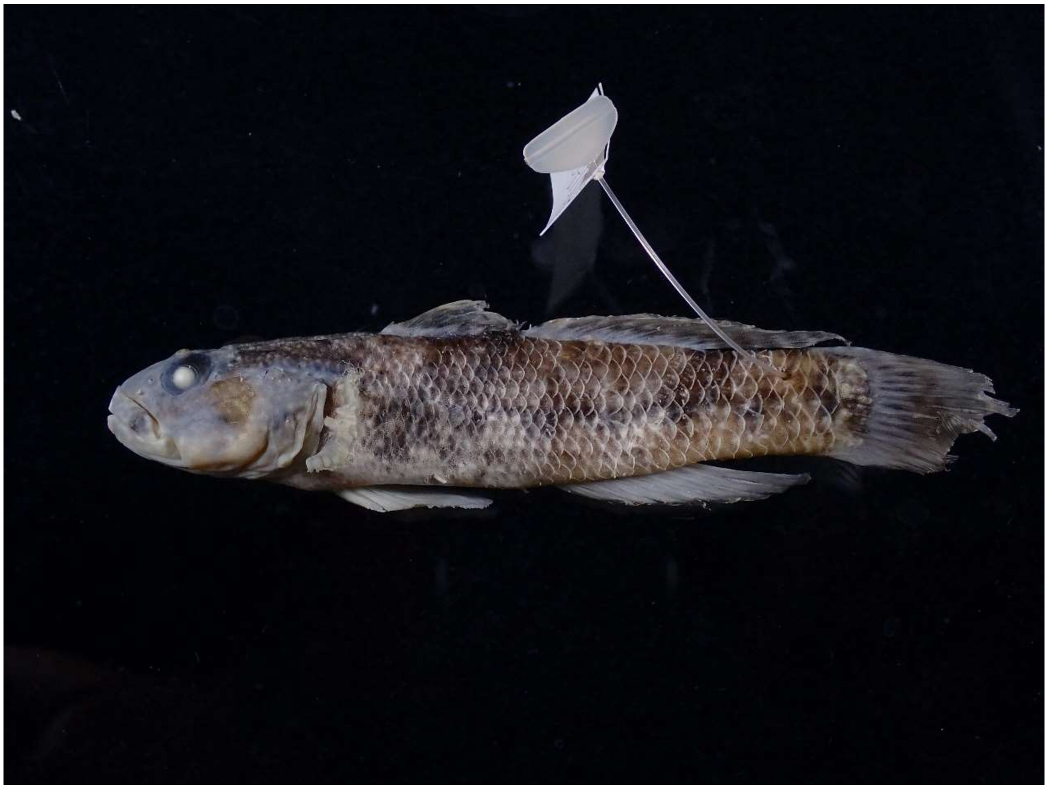

DOS 03156-1, *Bathygobius coalitus*, OR113751.

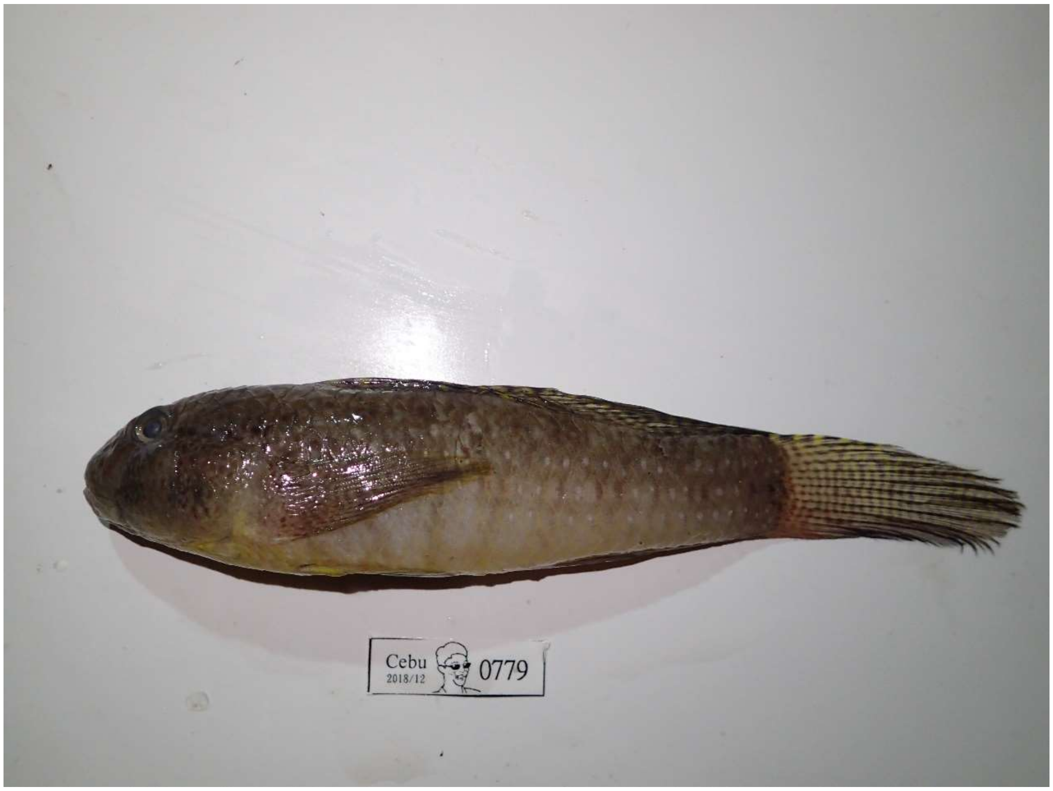

DOS 06707-1, *Exyrias puntang*, OR113895.

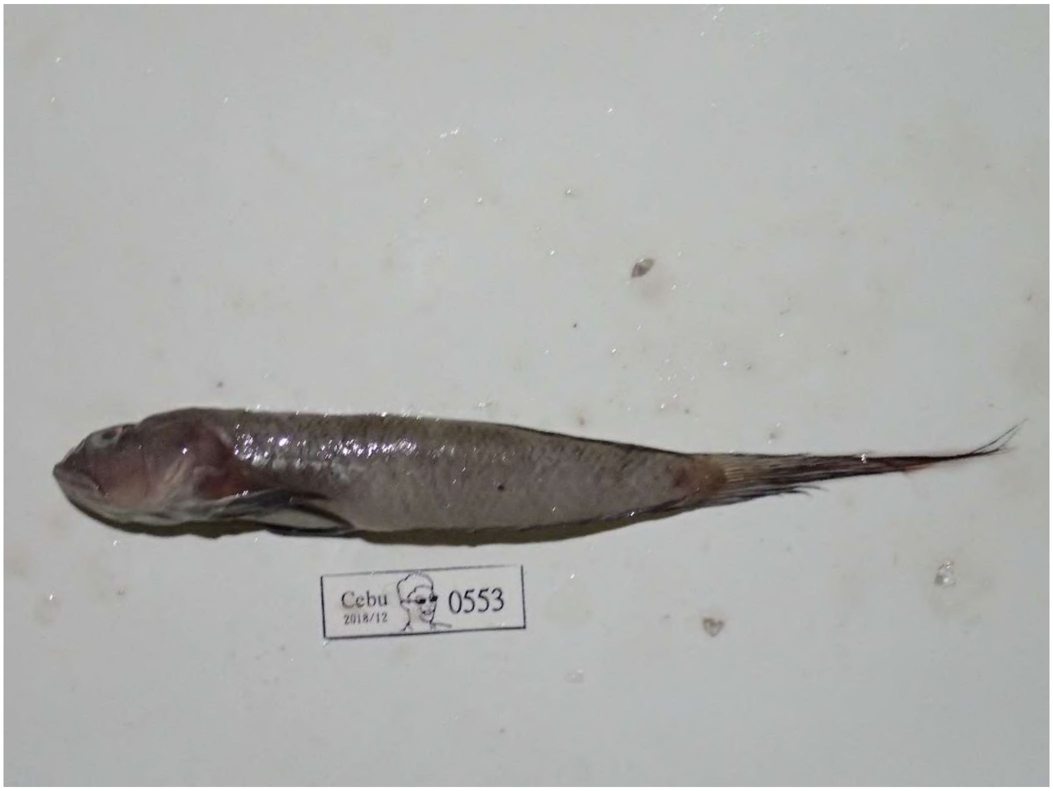

DOS 06862, *Oxyurichthys ophthalmonema*, OR114038.

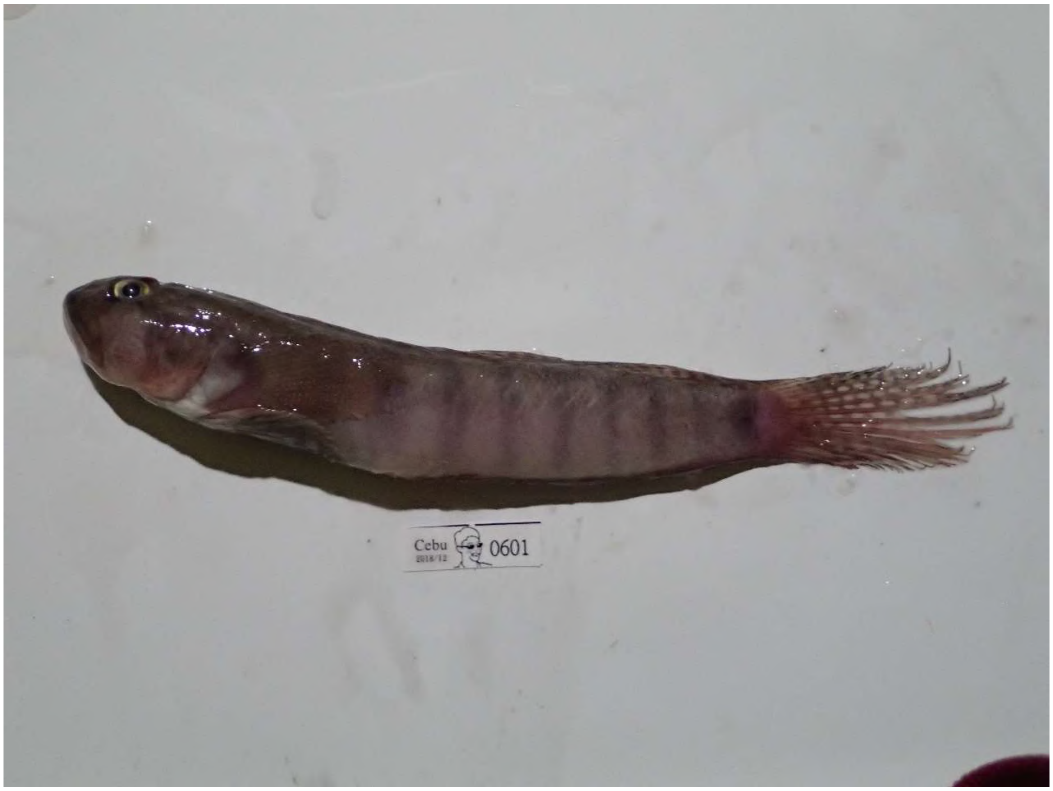

DOS 06710-1, *Oxyurichthys papuensis*, OR113898.

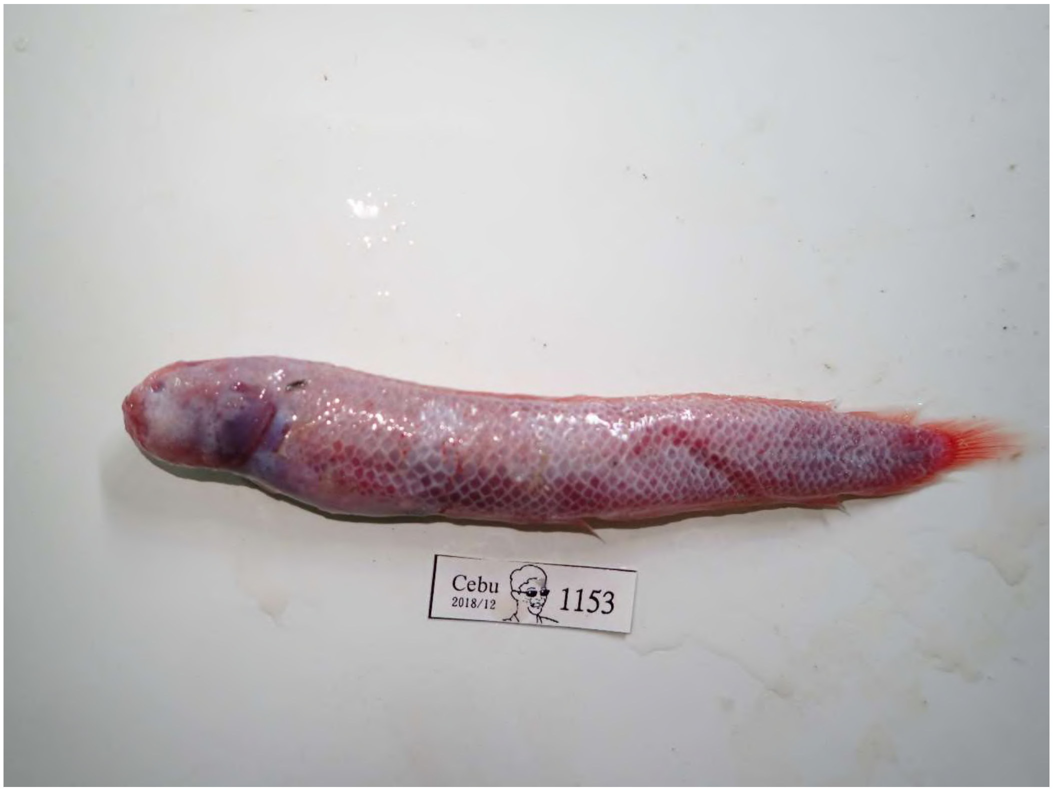

DOS 06711, *Paratrypauchen* sp., OR113899.

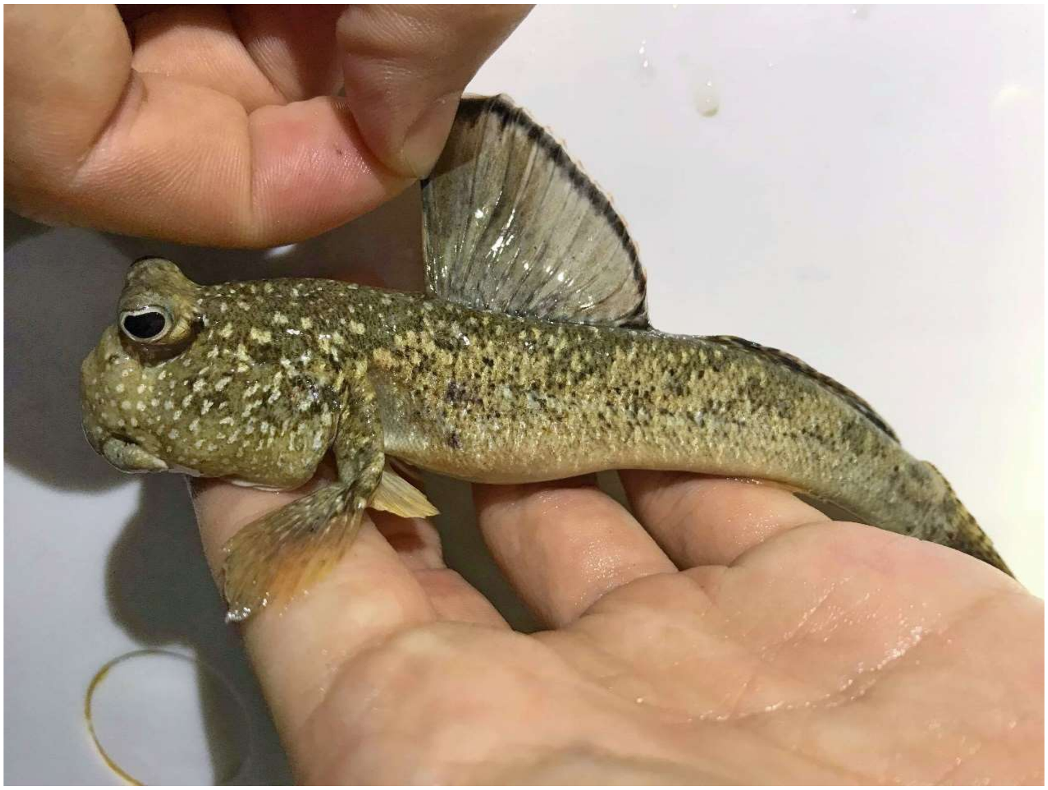

DOS 06566, *Periophthalmus kalolo*, OR113769.

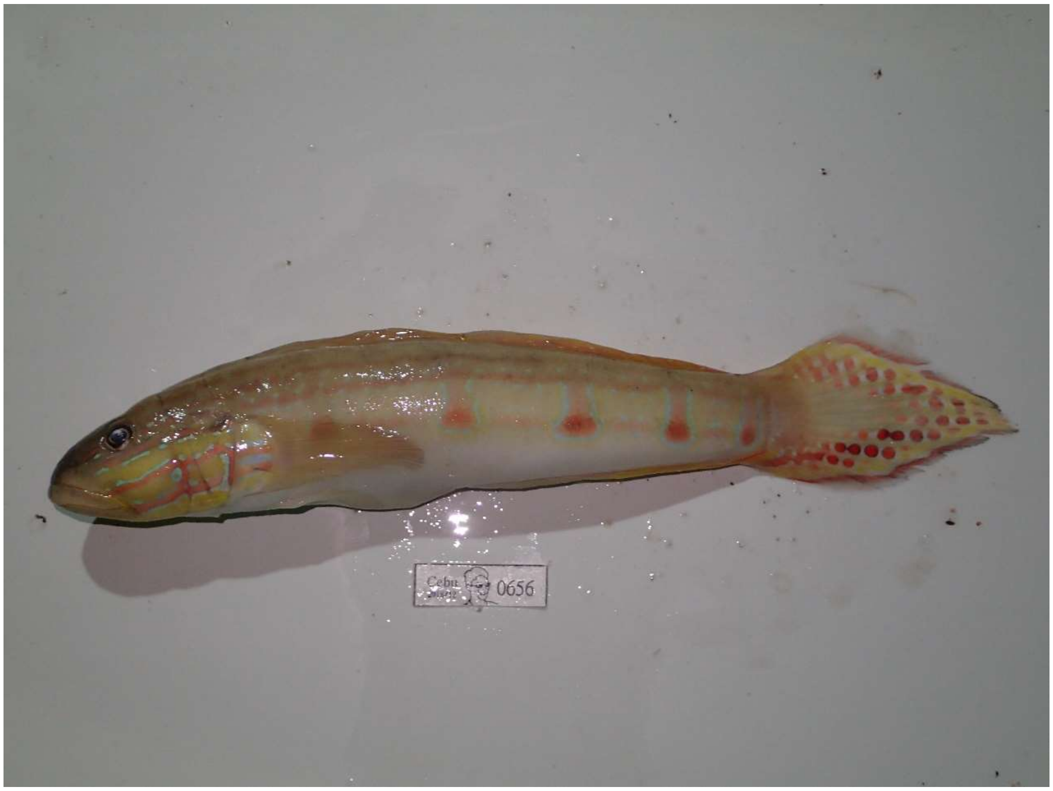

DOS 06709, *Valenciennea longipinnis*, OR113897.

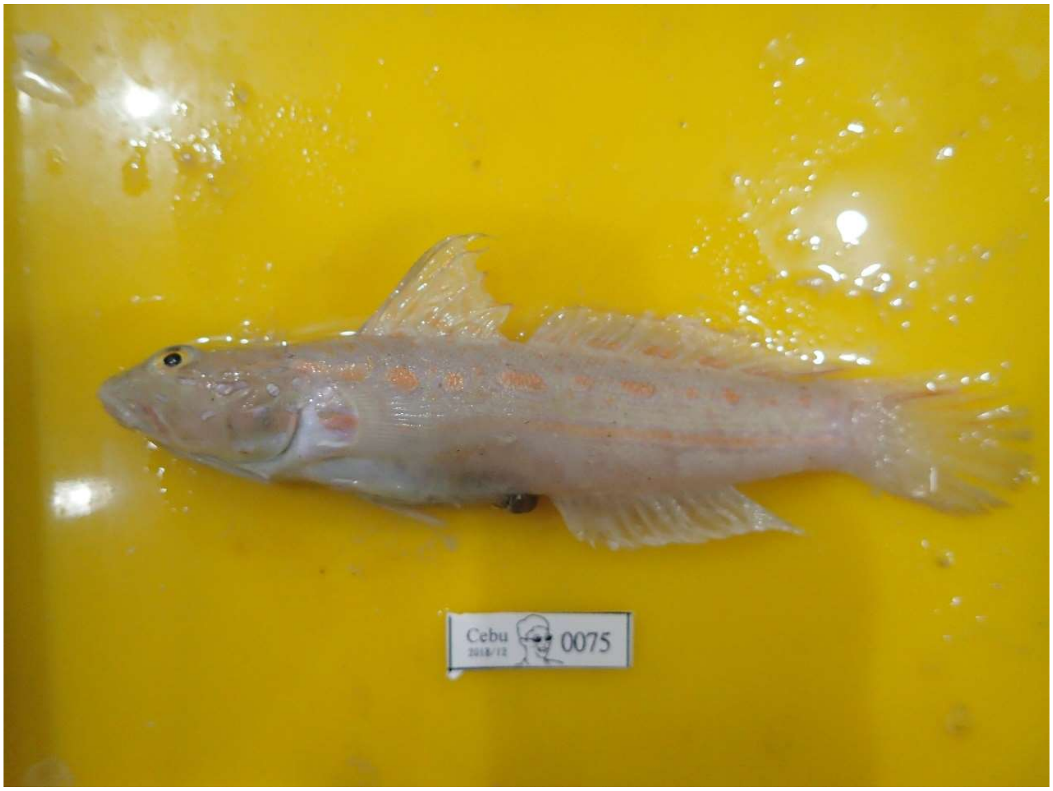

DOS 06706, *Valenciennea puellaris*, OR113894. (specimen not preserved)

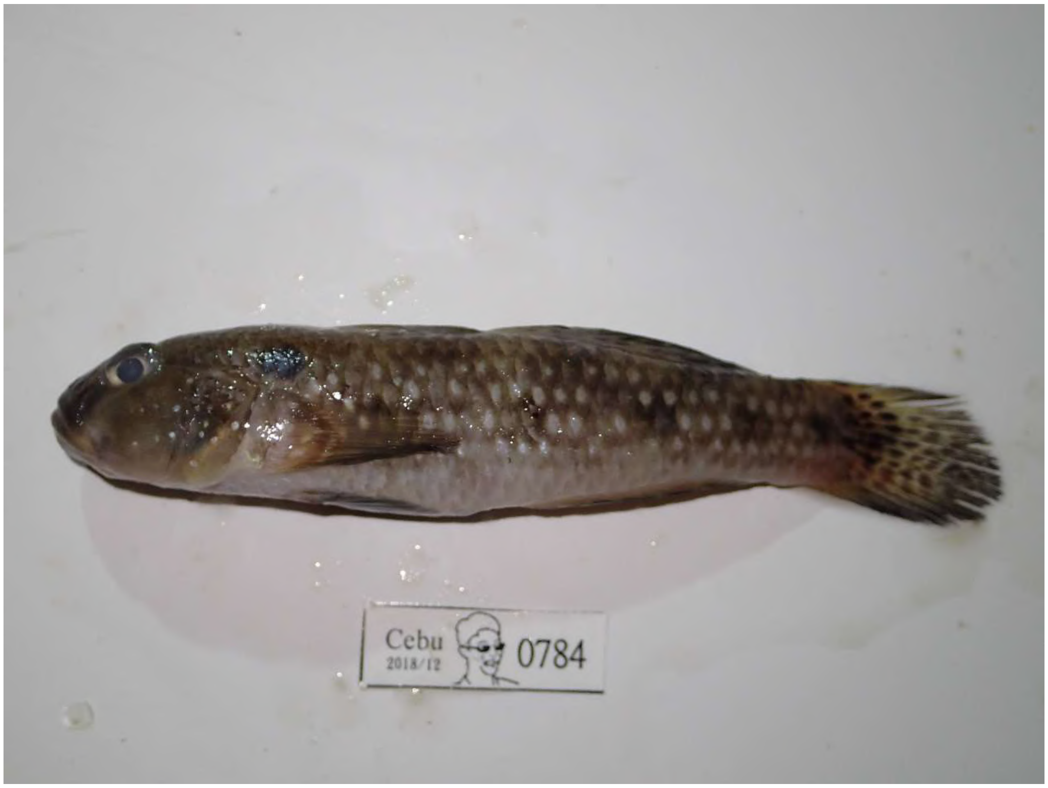

DOS 06712-1, *Yongeichthys nebulosus*, OR113900.

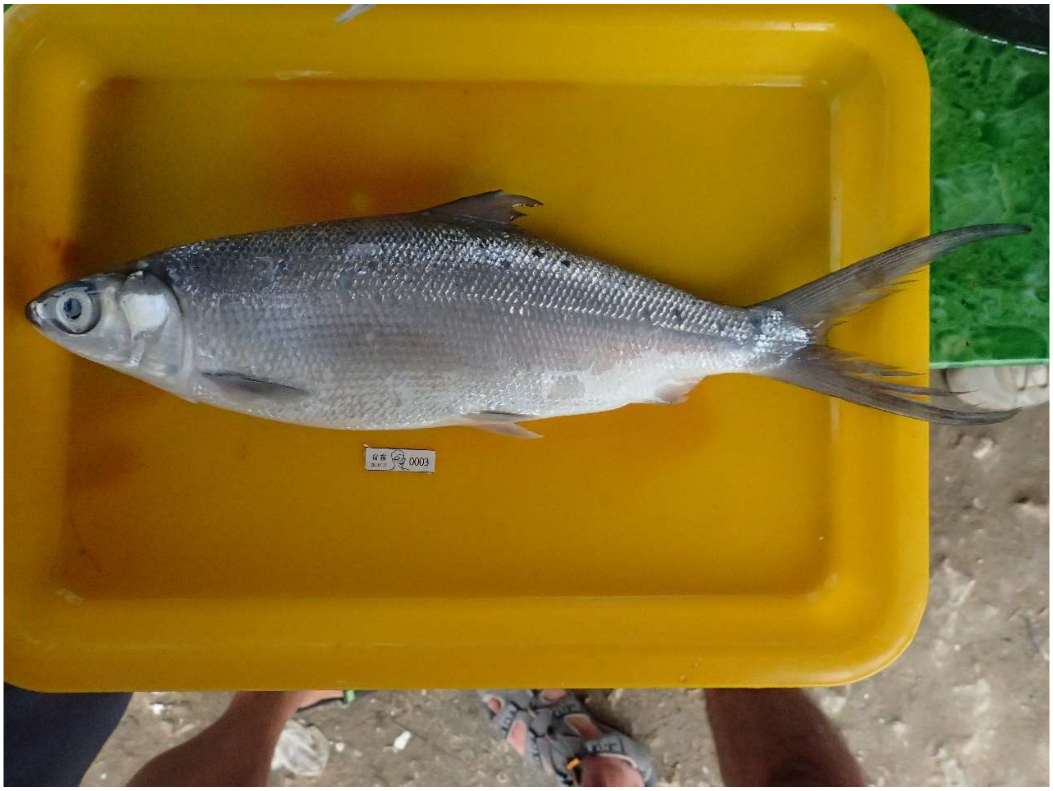

DOS 06672, *Chanos chanos*, OR113863. (specimen not preserved)

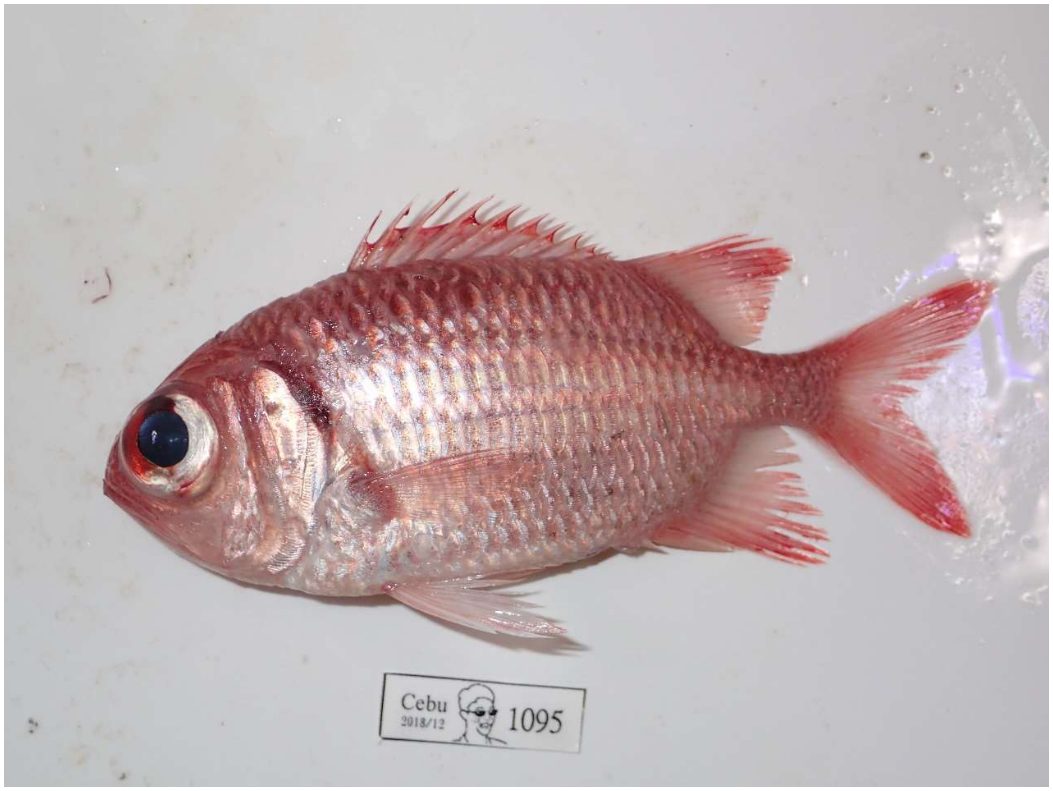

DOS 06727-6, *Myripristis hexagona*, OR113915.

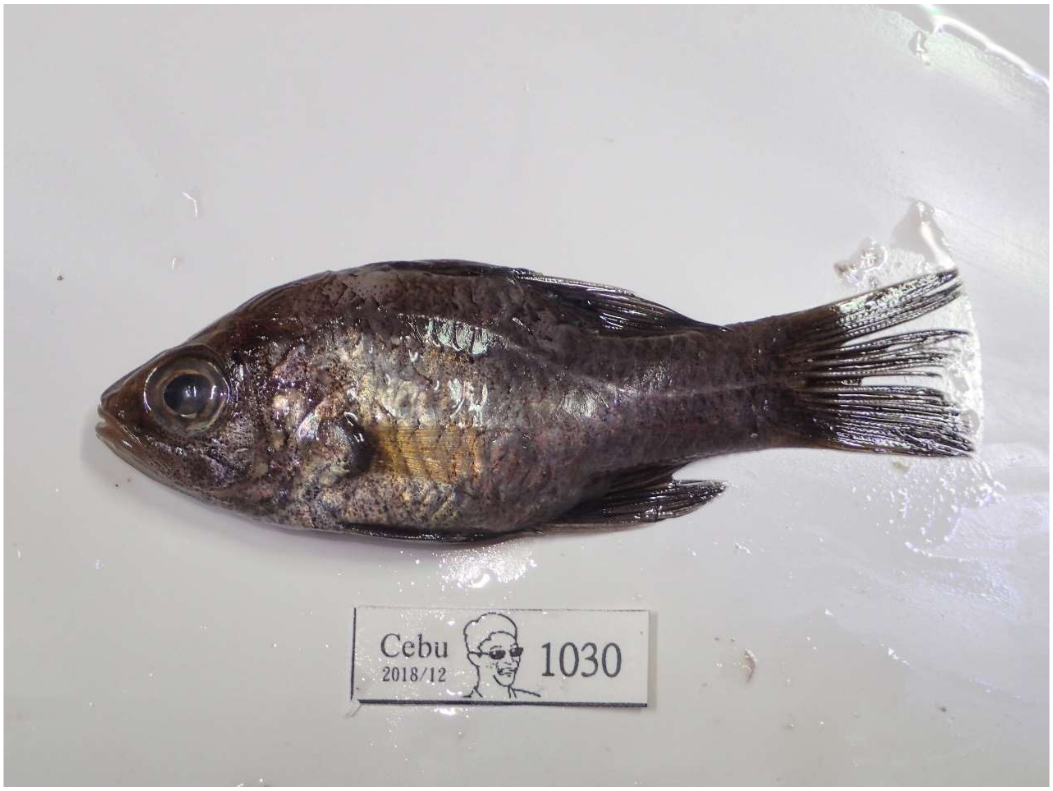

DOS 07004-1, *Apogonichthyoides melas*, OR114171.

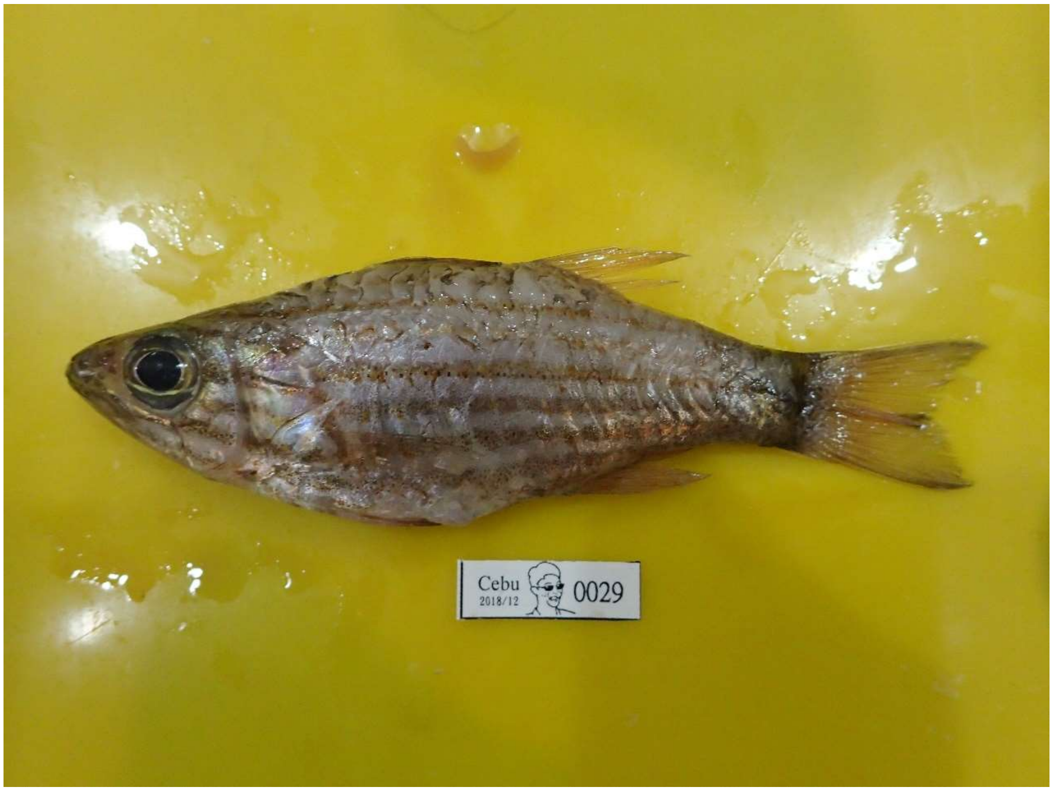

DOS 06999, *Cheilodipterus artus*, OR114166. (specimen not preserved)

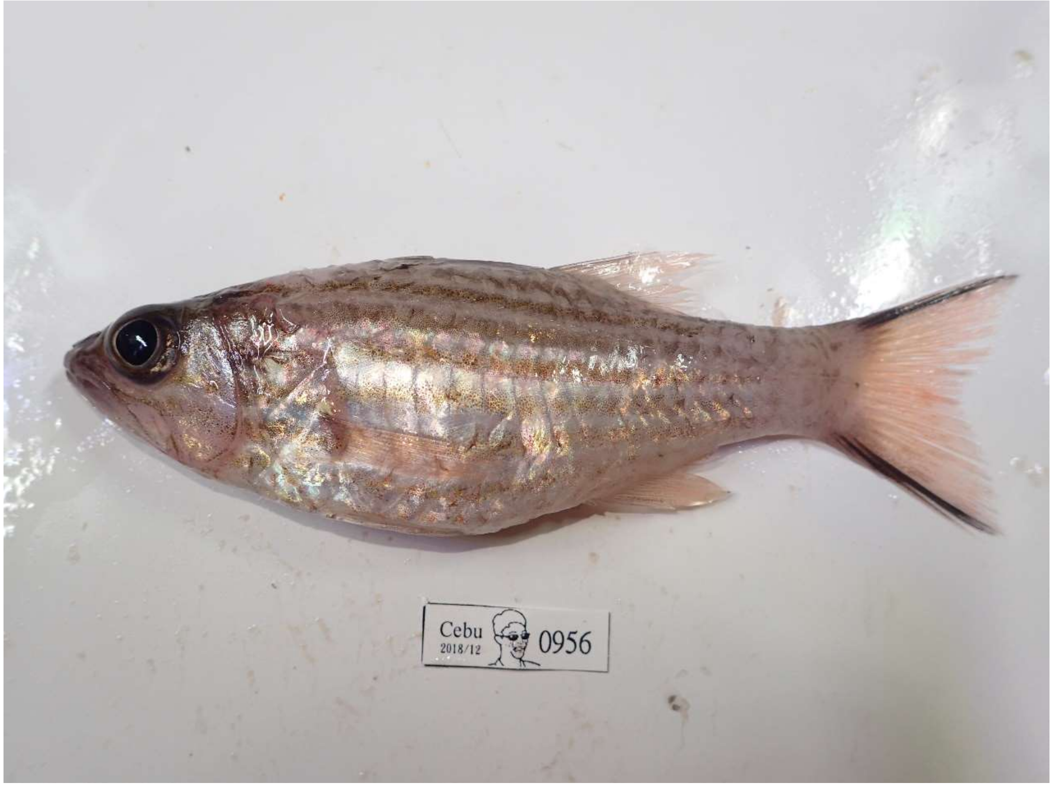

DOS 07005, *Cheilodipterus macrodon*, OR114172.

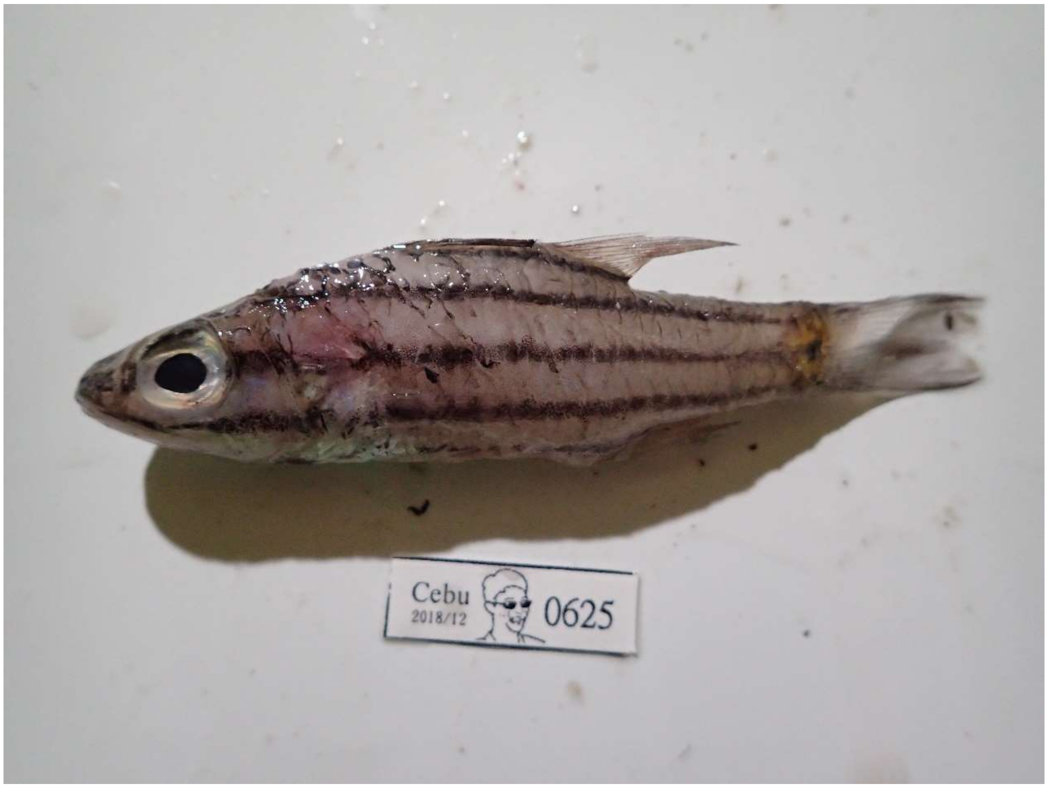

DOS 07006-1, *Cheilodipterus quinquelineatus*, OR114173.

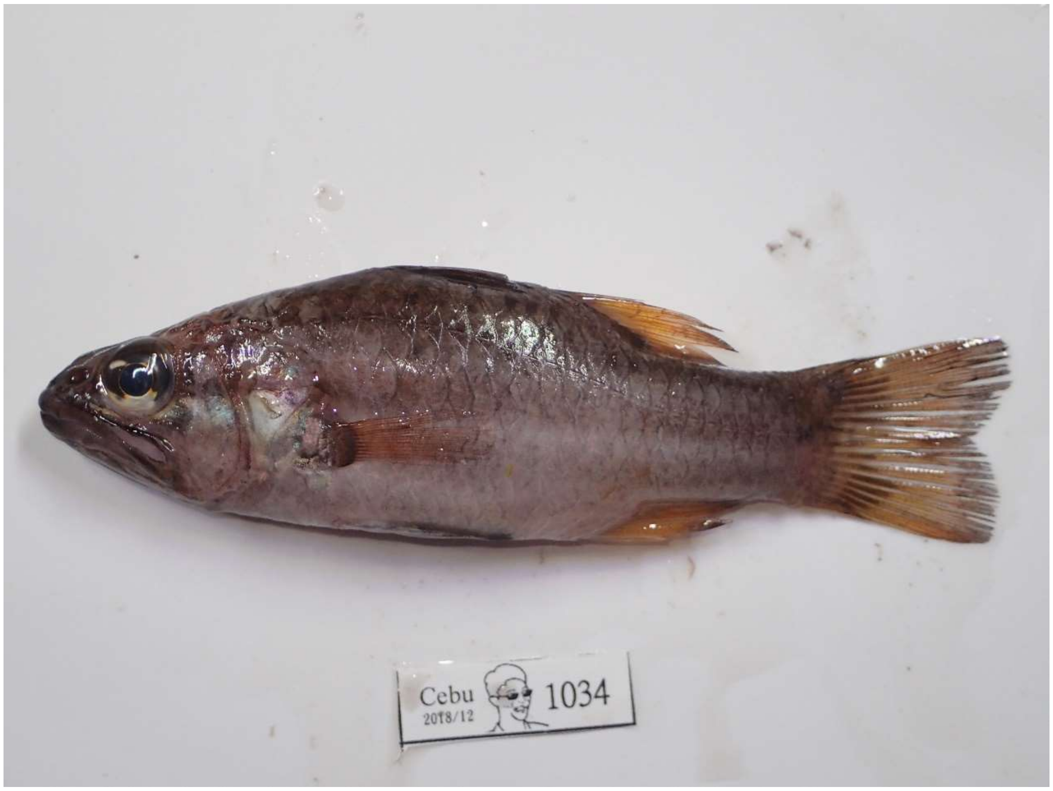

DOS 06589-1, *Cheilodipterus singapurensis*, OR113780.

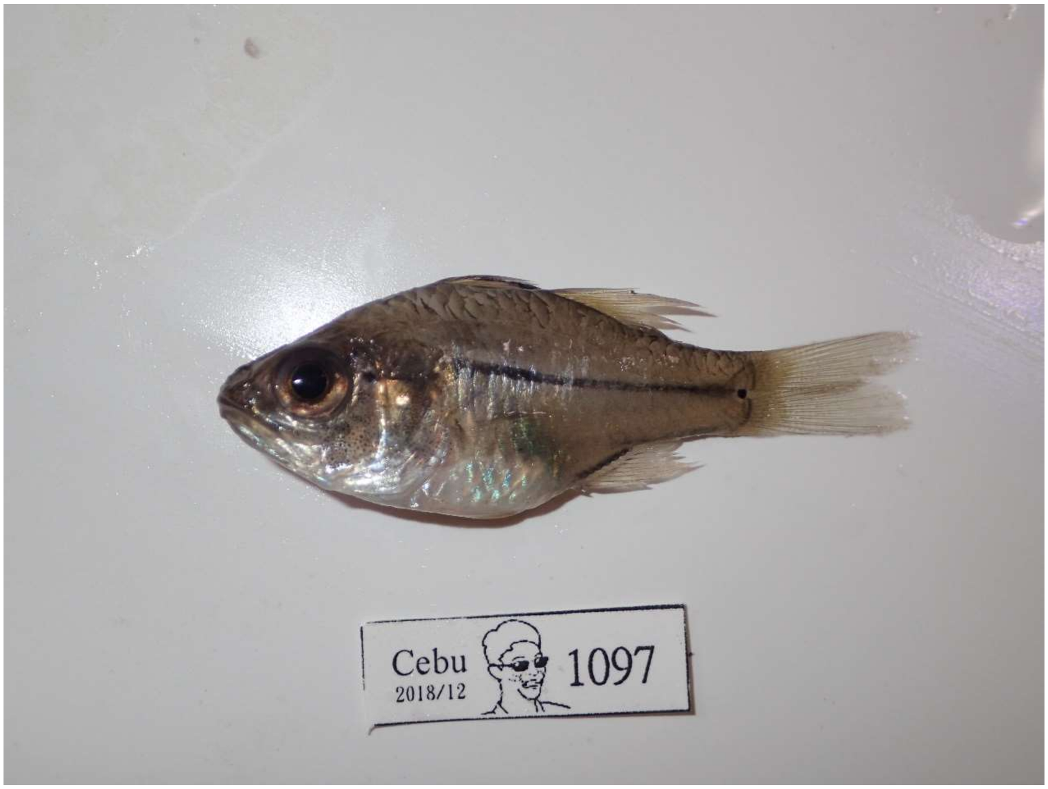

DOS 06599-1, *Fibramia lateralis*, OR113790. (specimen not preserved)

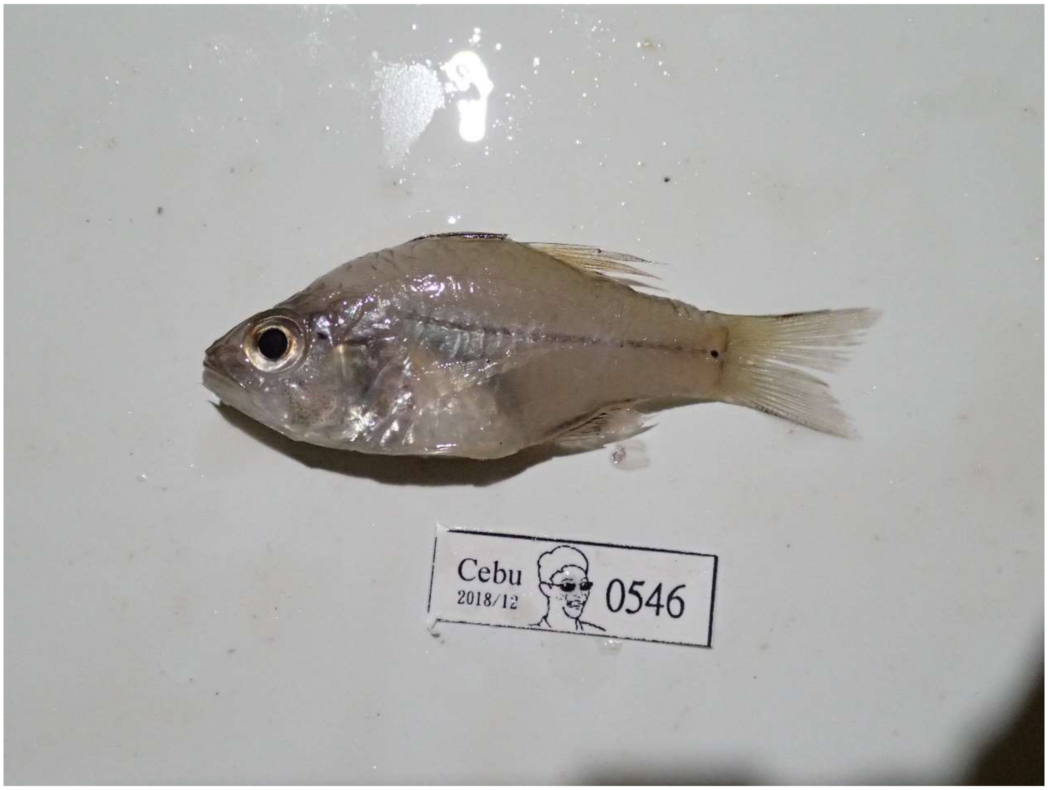

DOS 07002-1, *Fibramia lateralis*, OR114169.

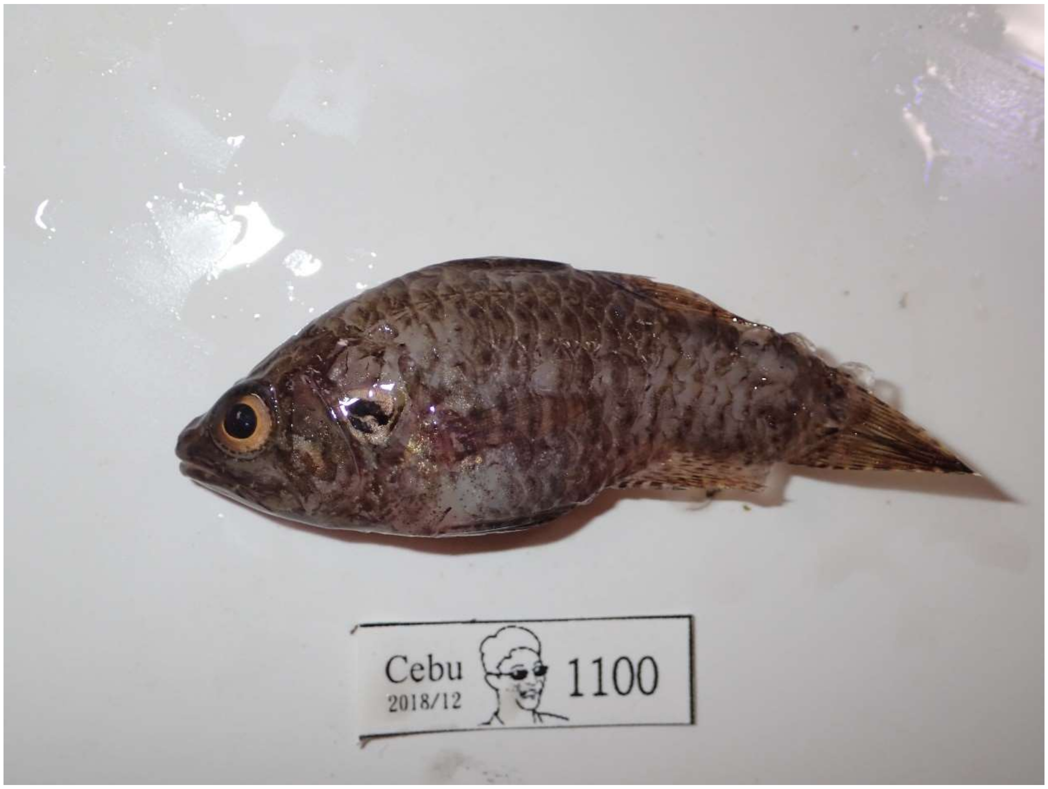

DOS 06591-1, *Fowleria variegata*, OR113783.

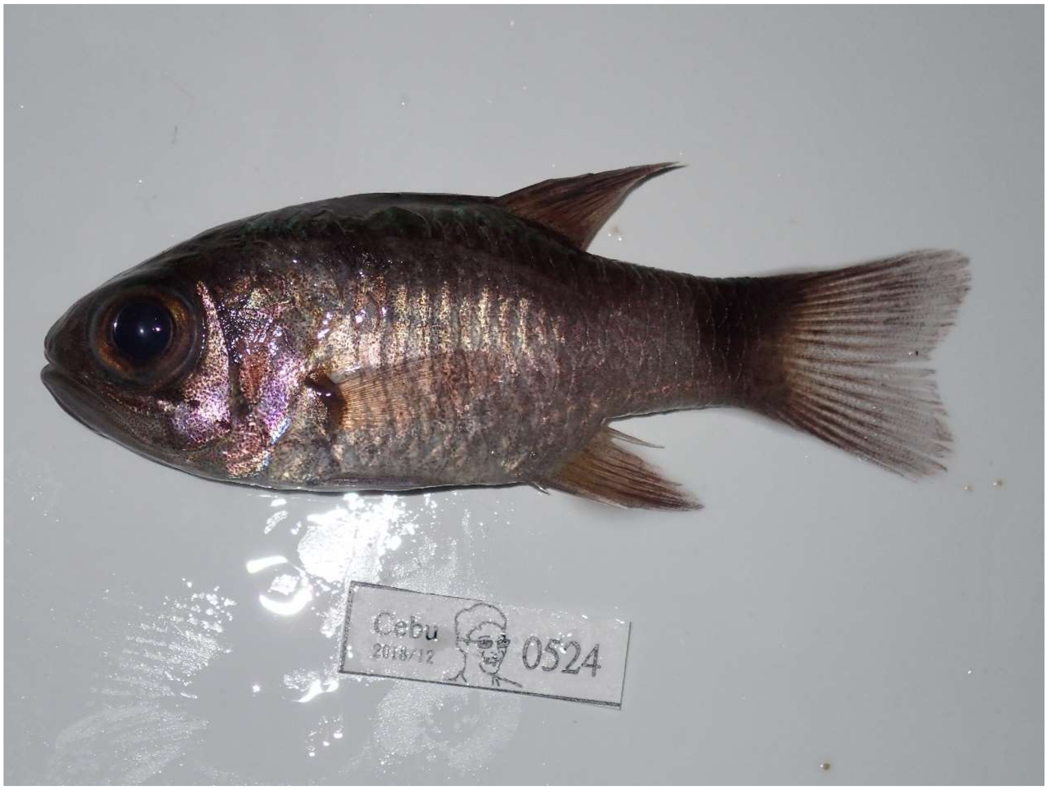

DOS 06594-2, *Nectamia fusca*, OR113784.

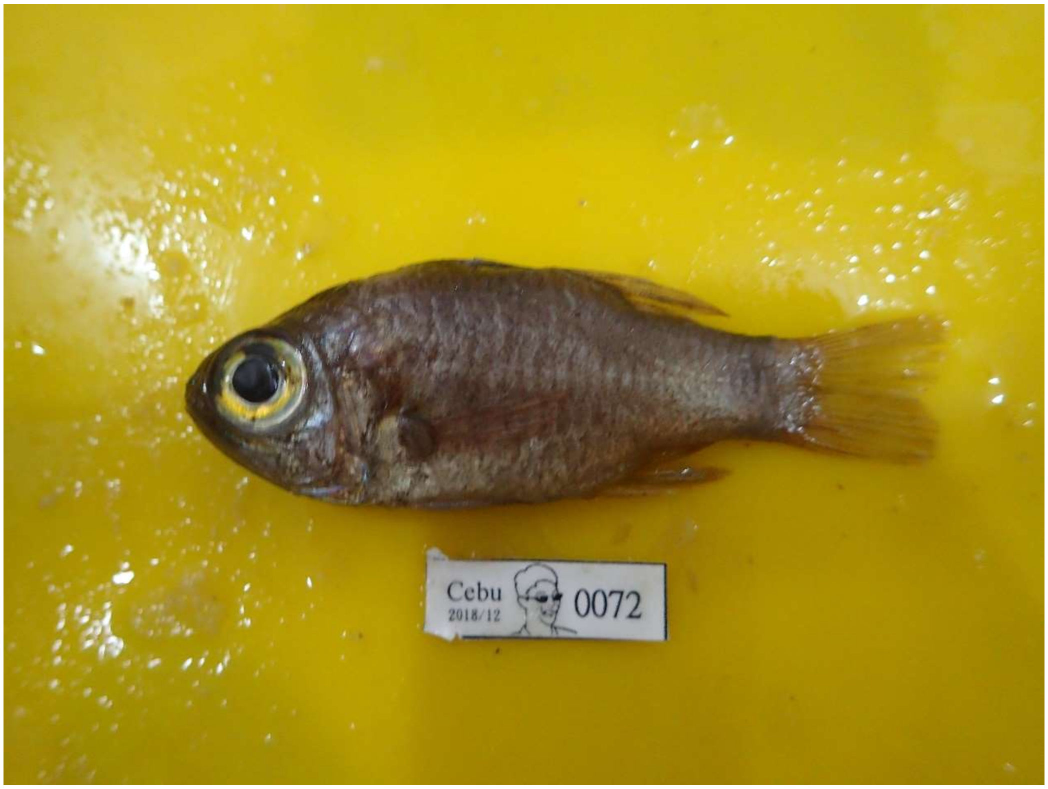

DOS 07001, *Nectamia fusca*, OR114168.

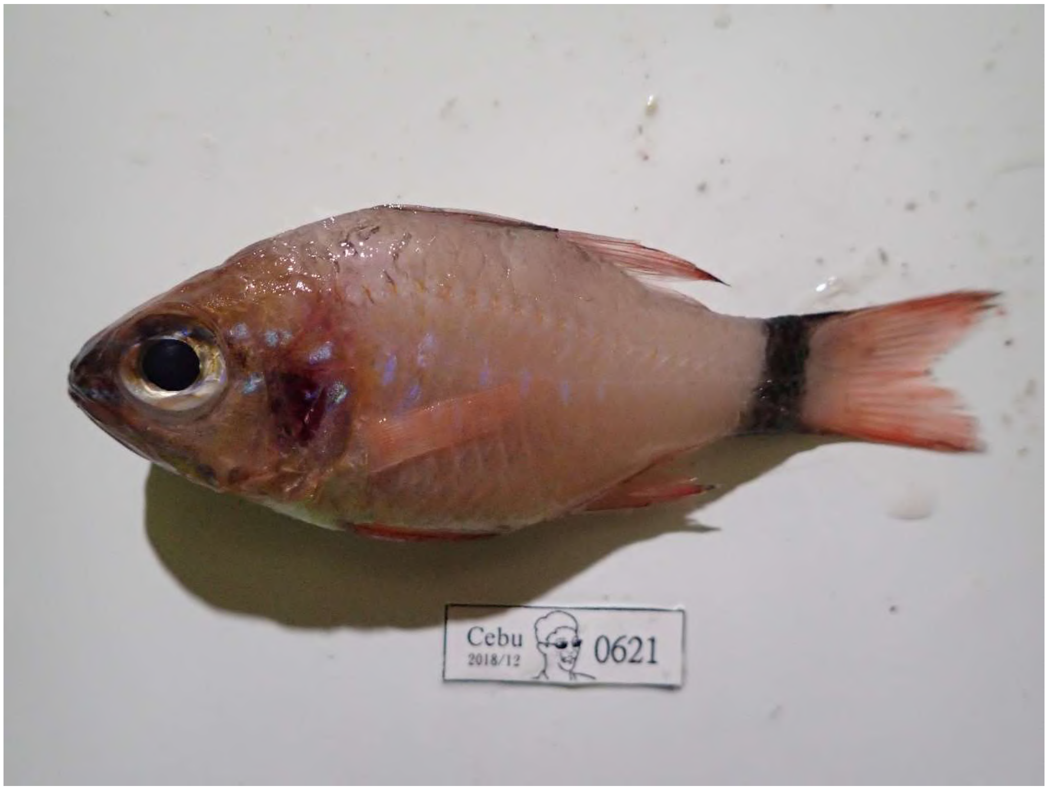

DOS 06595-1, *Ostorhinchus aureus*, OR113785.

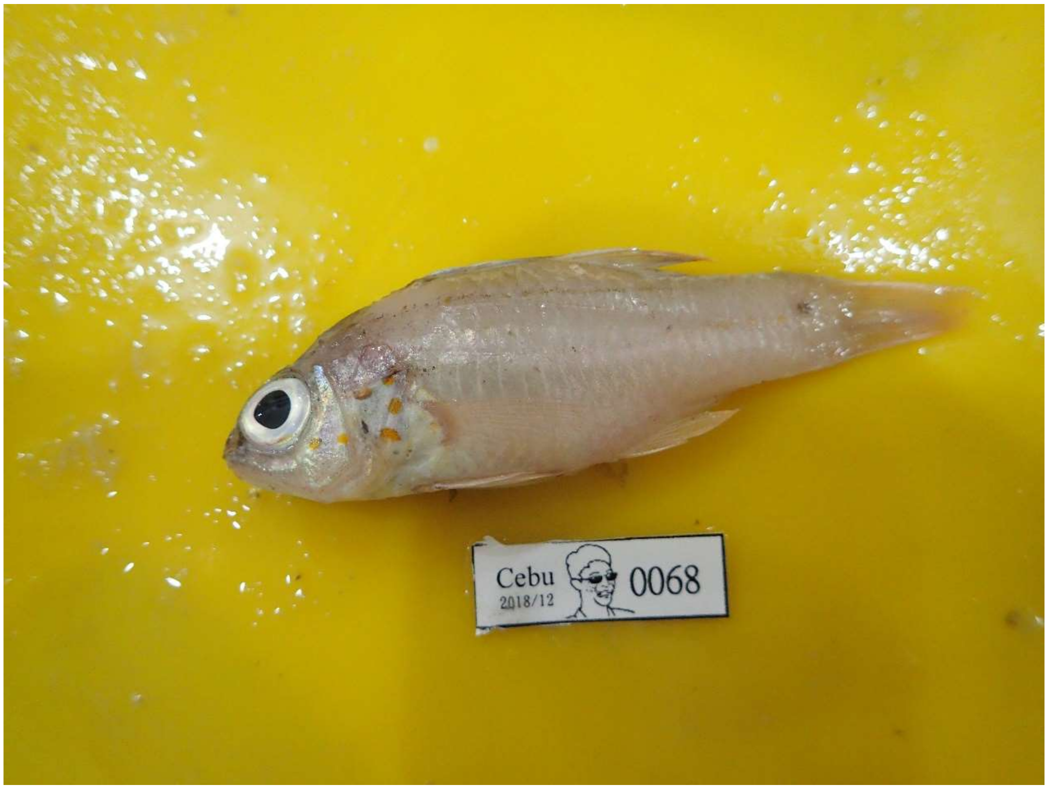

DOS 06597-2, *Ostorhinchus chrysopomus*, OR113787. (specimen not preserved)

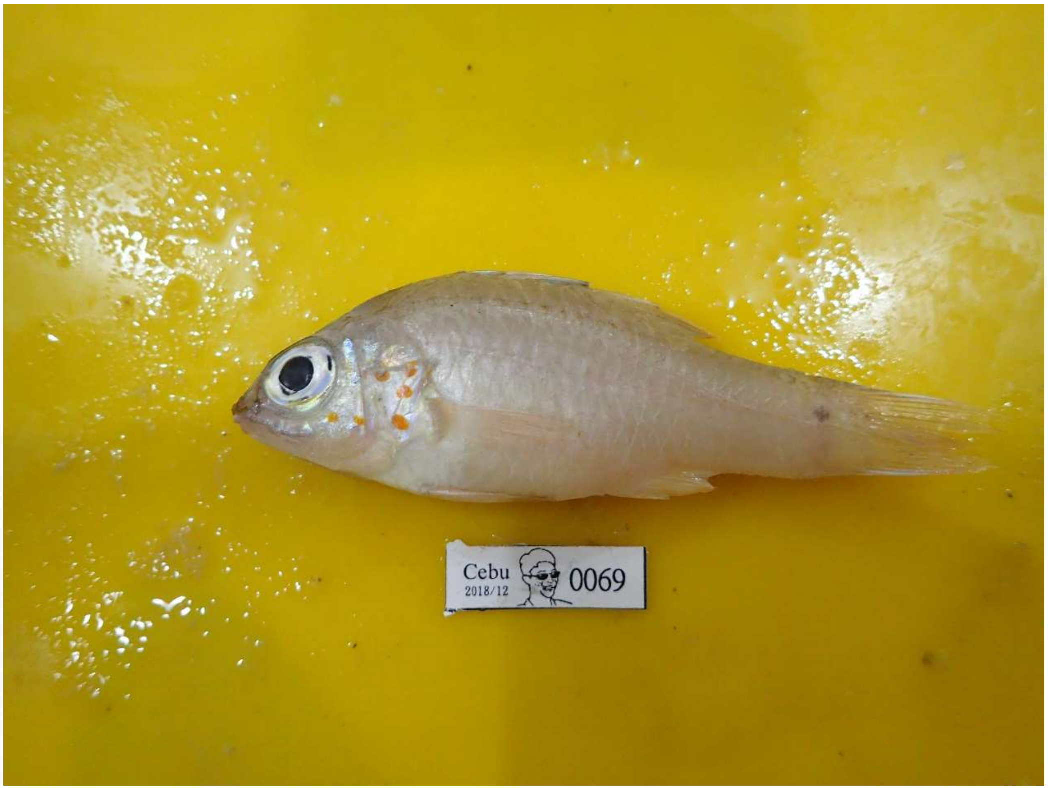

DOS 06597-3, *Ostorhinchus chrysopomus*, OR113788.

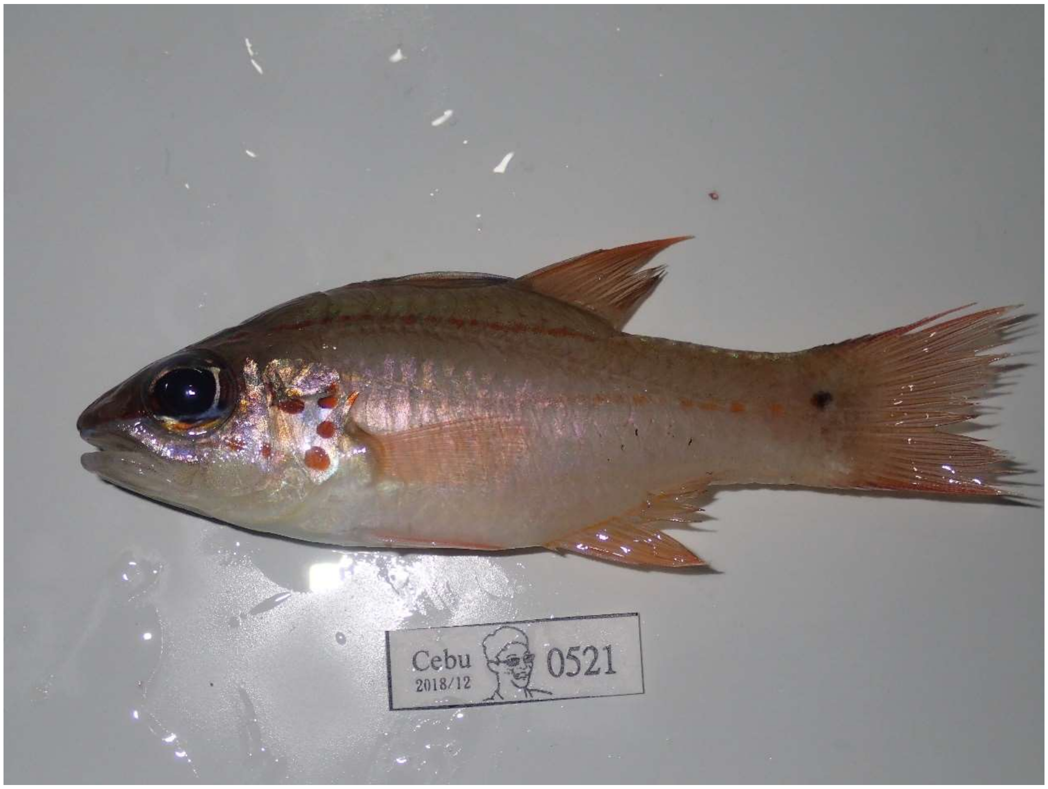

DOS 06598-1, *Ostorhinchus chrysopomus*, OR113789.

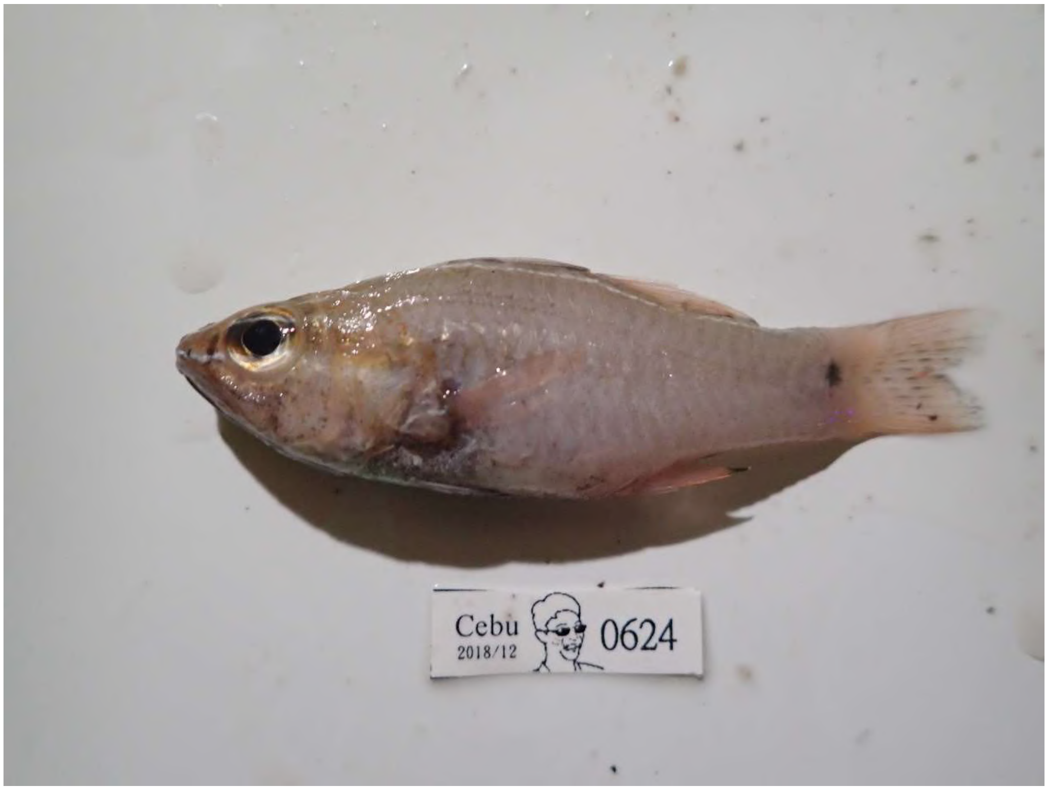

DOS 06590-3, *Ostorhinchus hartzfeldii*, OR113782.

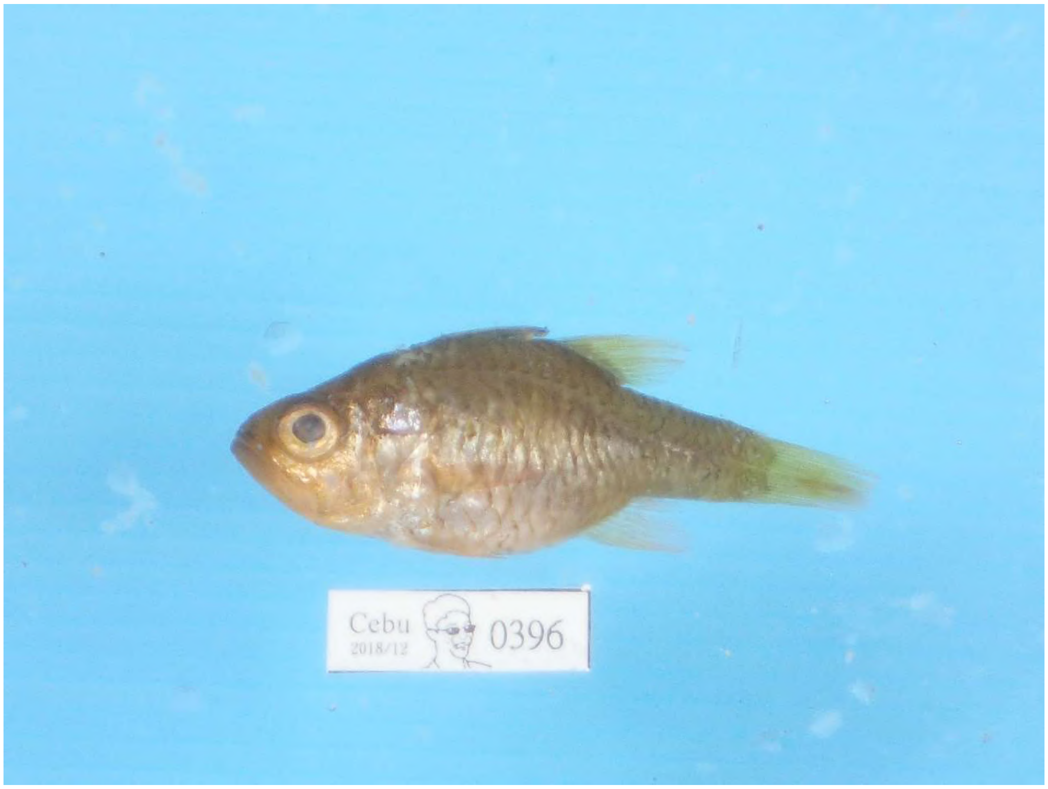

DOS 07003-1, *Ostorhinchus ishigakiensis*, OR114170.

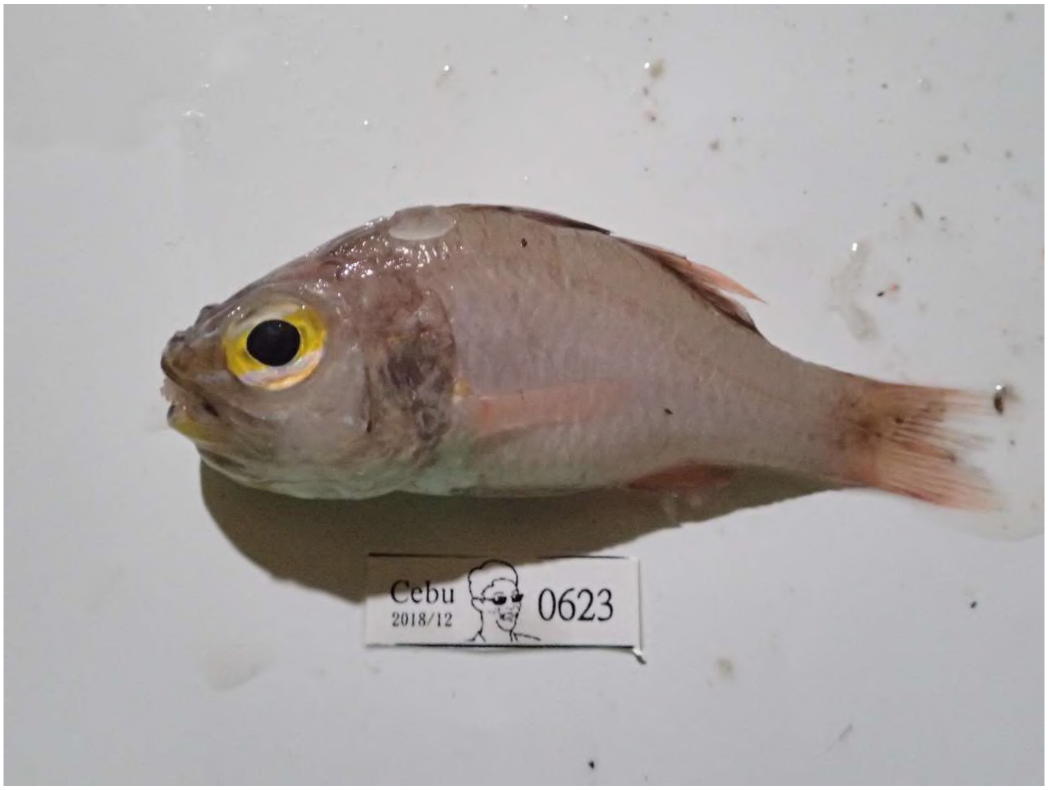

DOS 06590-2, *Ostorhinchus monospilus*, OR113781.

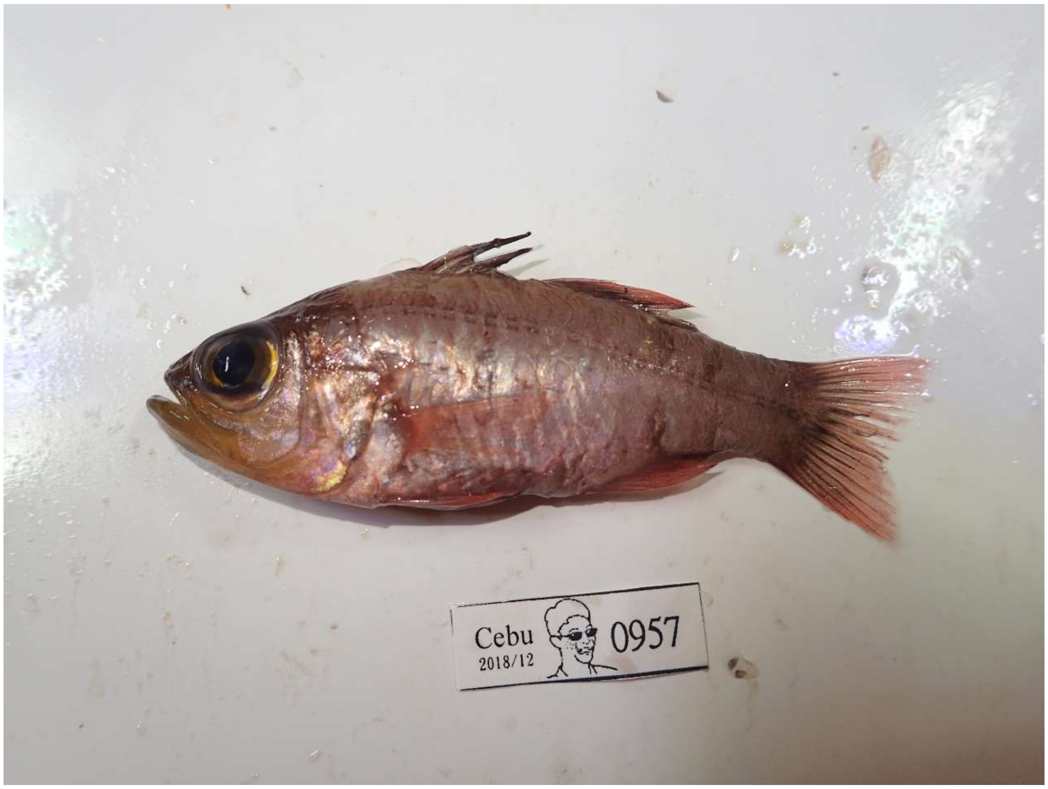

DOS 07000, *Ostorhinchus monospilus*, OR114167.

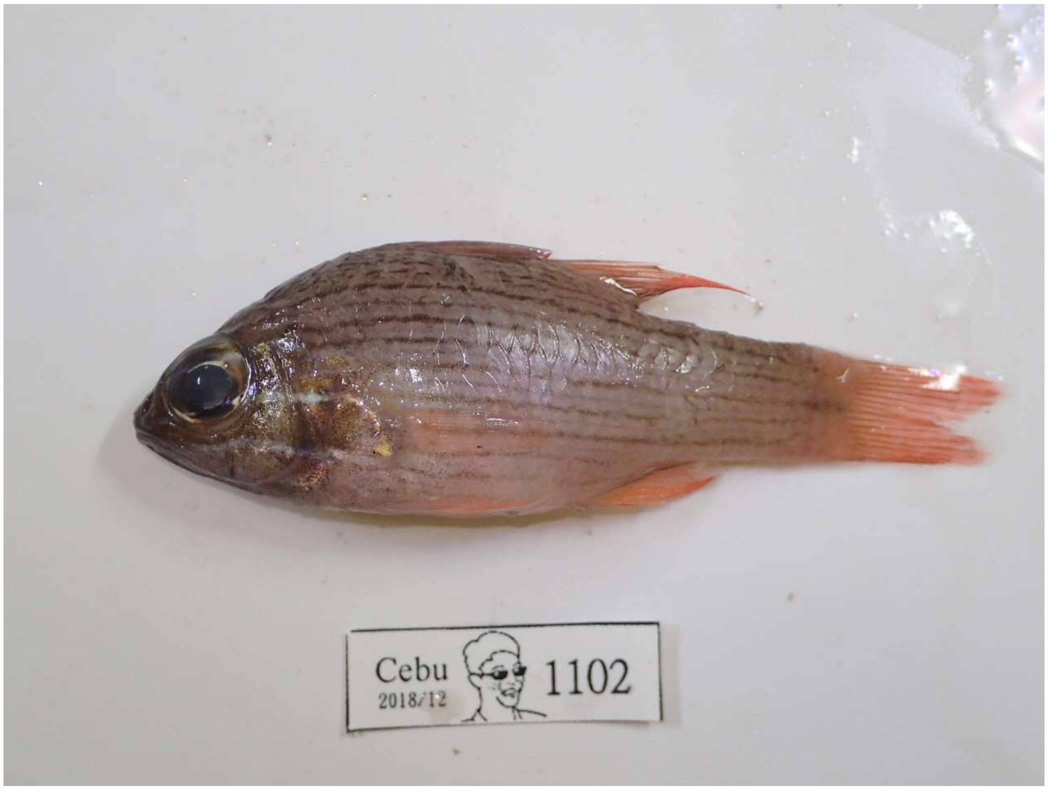

DOS 06596, *Ostorhinchus multilineatus*, OR113786.

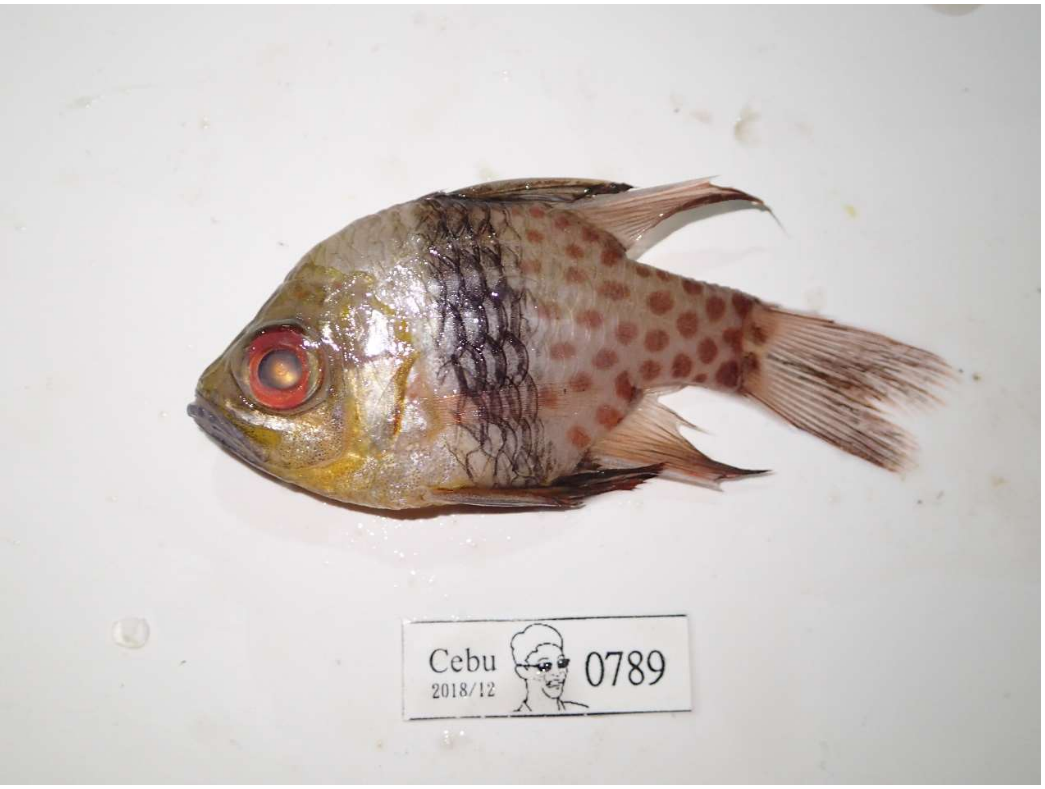

DOS 06600, *Sphaeramia nematoptera*, OR113791.

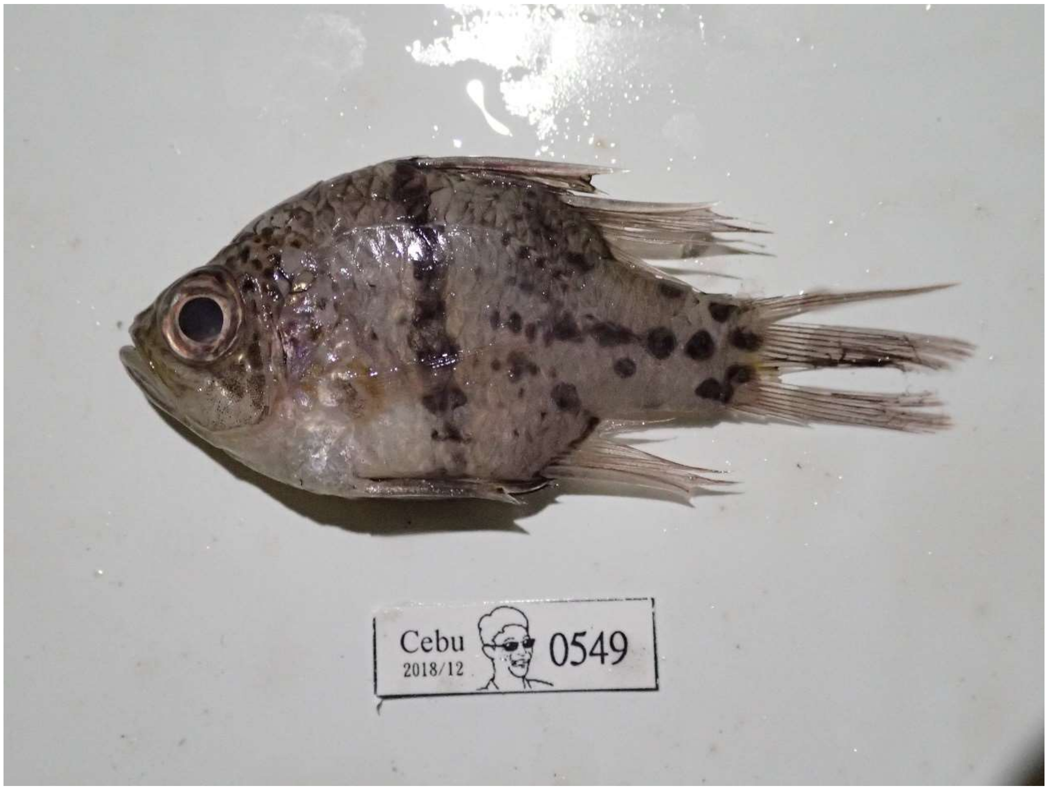

DOS 06601-1, *Sphaeramia orbicularis*, OR113792.

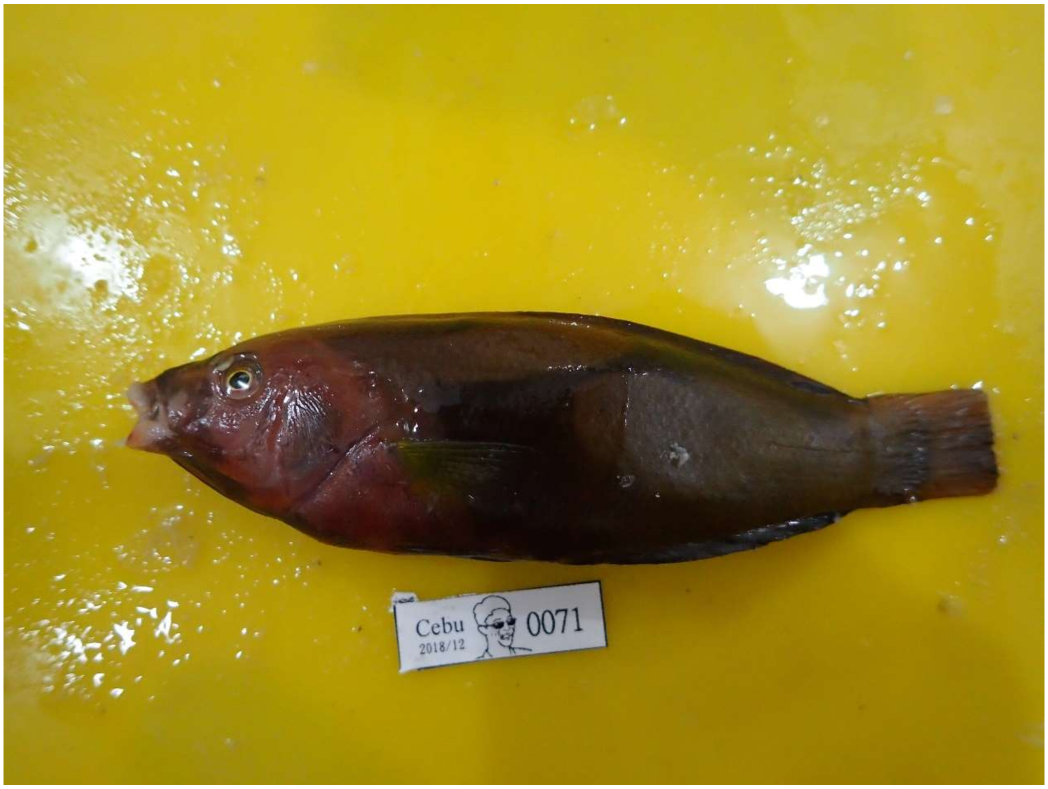

DOS 06729, *Anampses geographicus*, OR113916. (specimen not preserved)

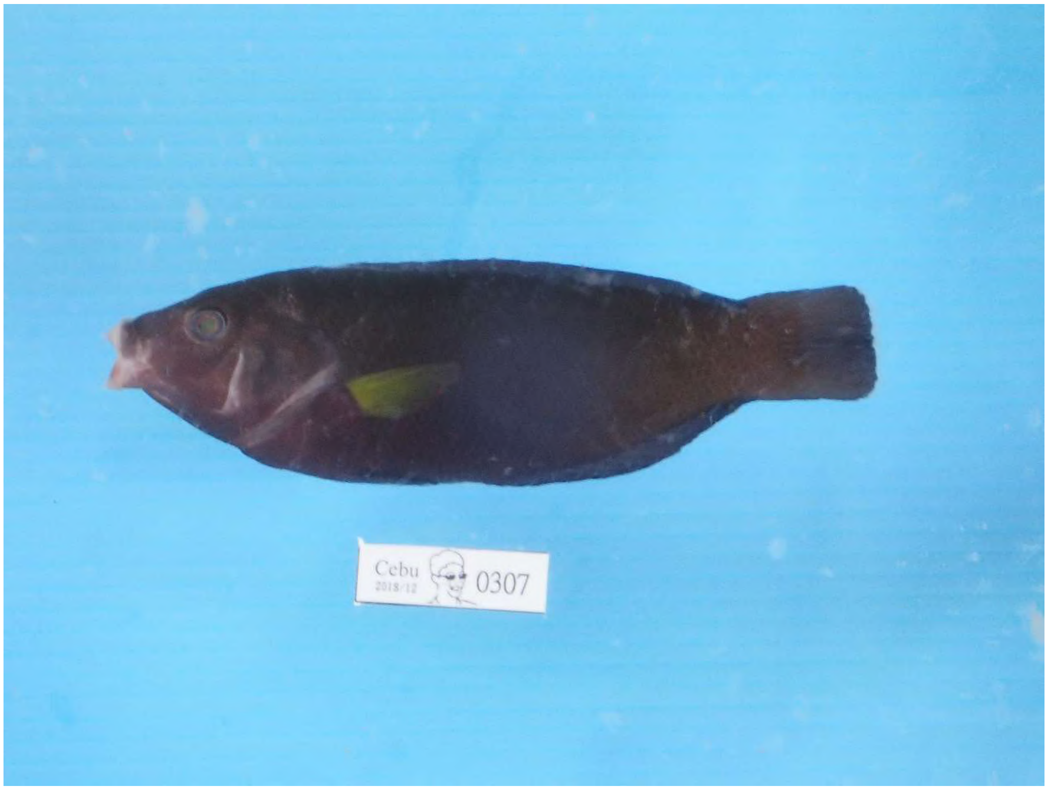

DOS 06922, *Anampses geographicus*, OR114098.

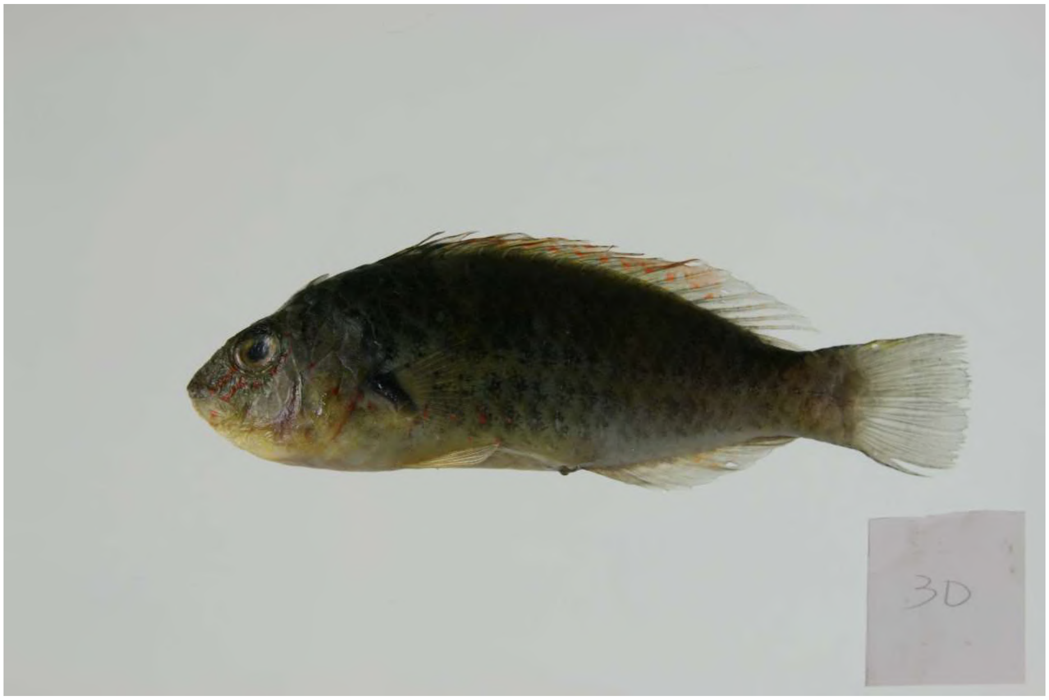

DOS 08648-1, *Calotomus spinidens*, OR114222.

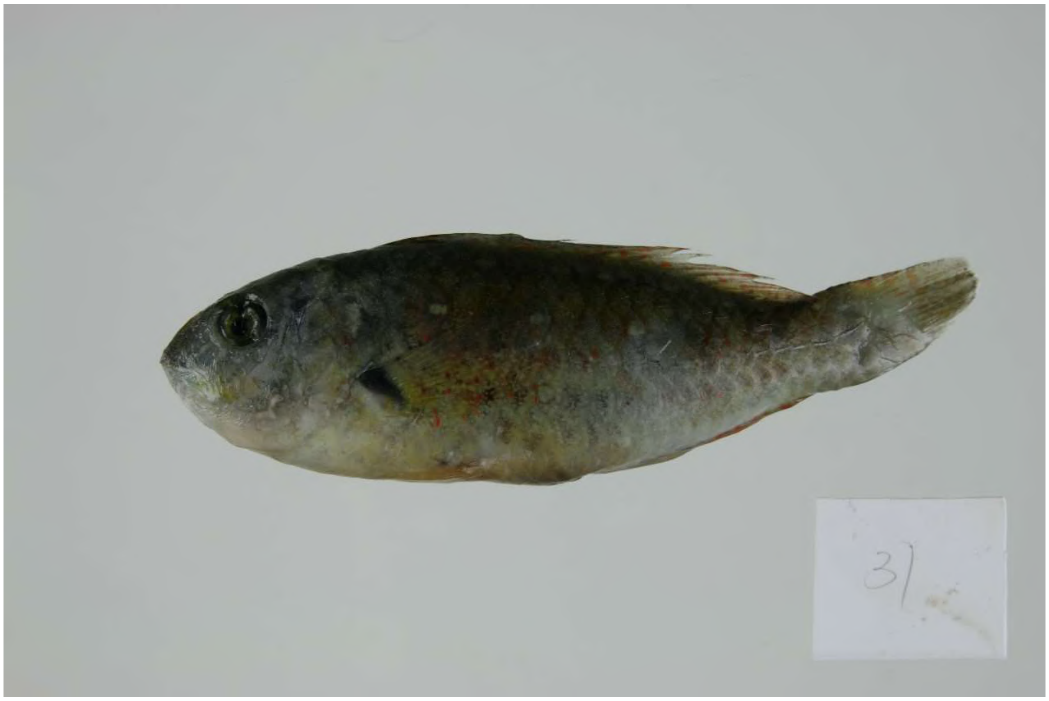

DOS 08648-2, *Calotomus spinidens*, OR114223.

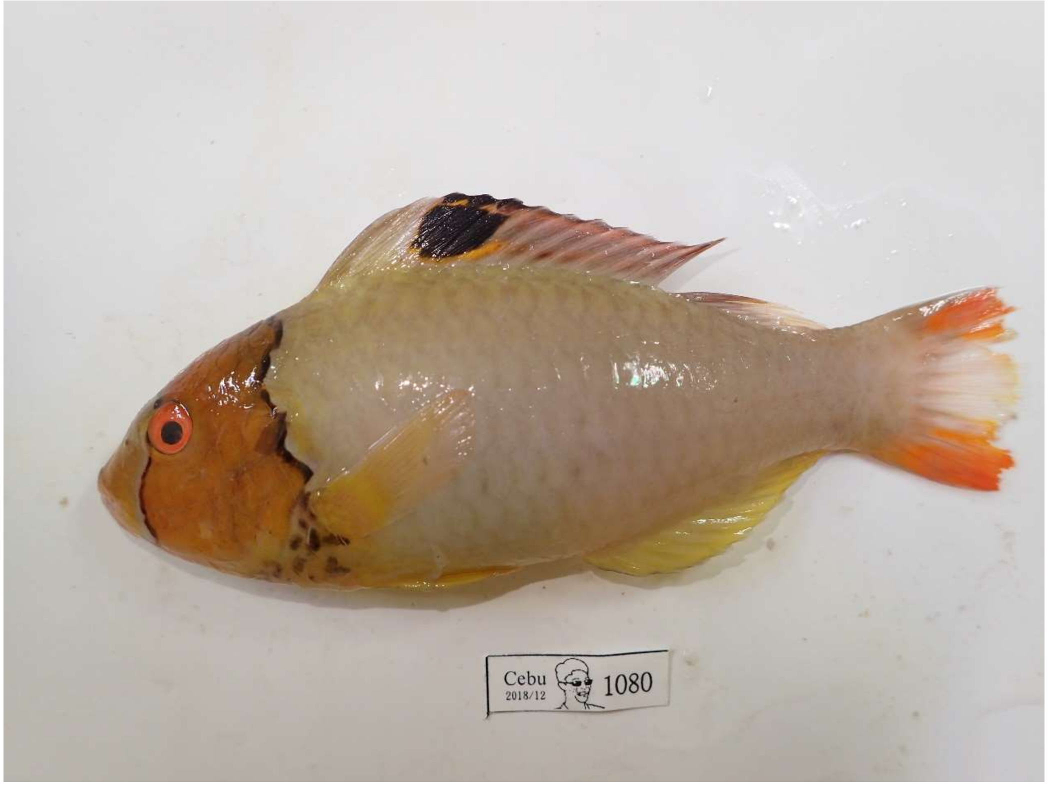

DOS 06913, *Cetoscarus ocellatus*, OR114090.

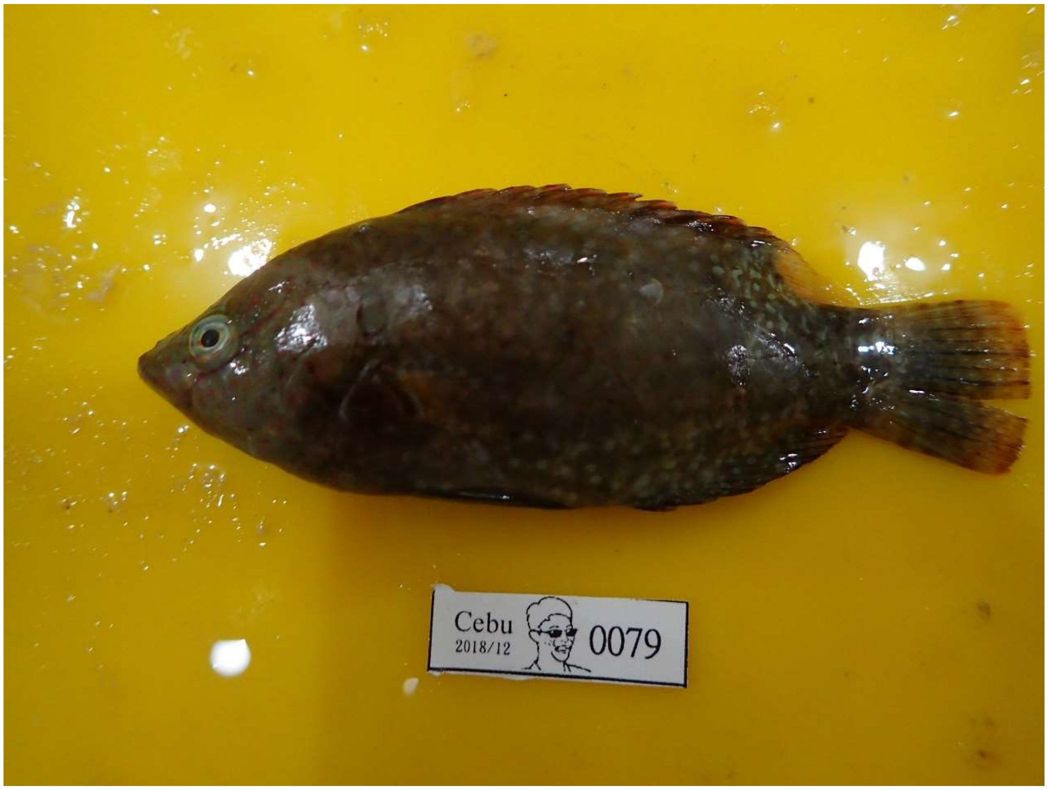

DOS 06732, *Cheilinus chlorourus*, OR113918. (specimen not preserved)

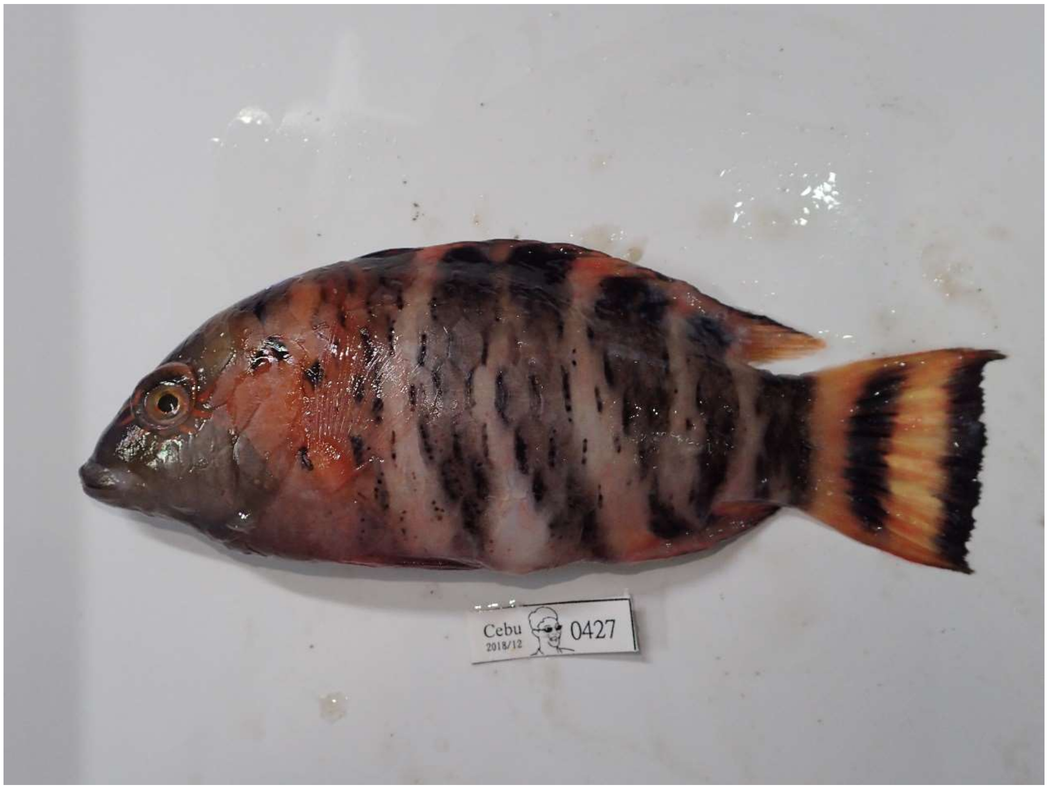

DOS 06731-1, *Cheilinus fasciatus*, OR113917. (specimen not preserved)

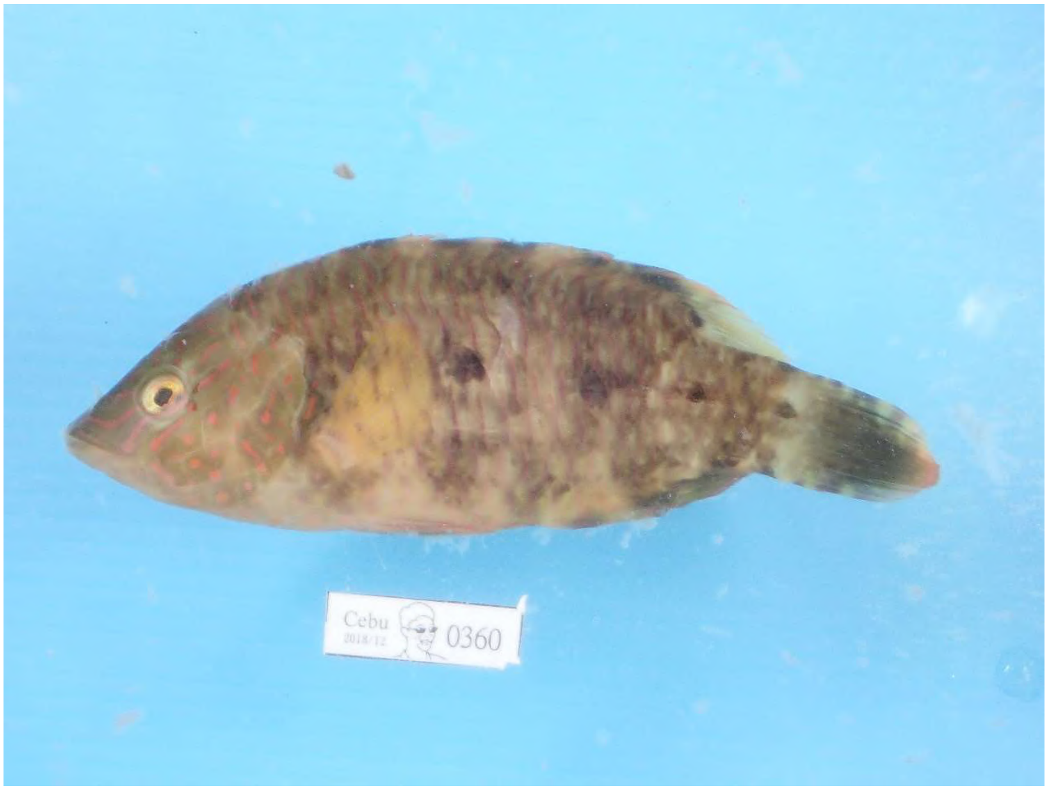

DOS 06733-1, *Cheilinus trilobatus*, OR113919.

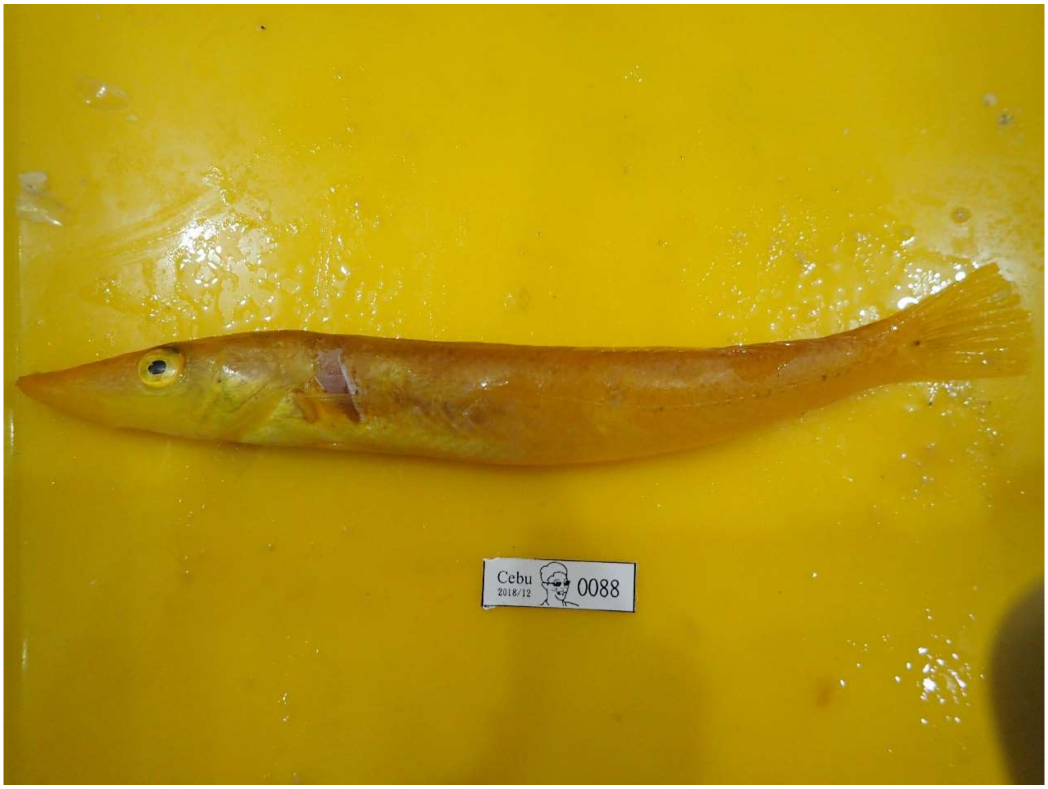

DOS 06735, *Cheilio inermis*, OR113920. (specimen not preserved)

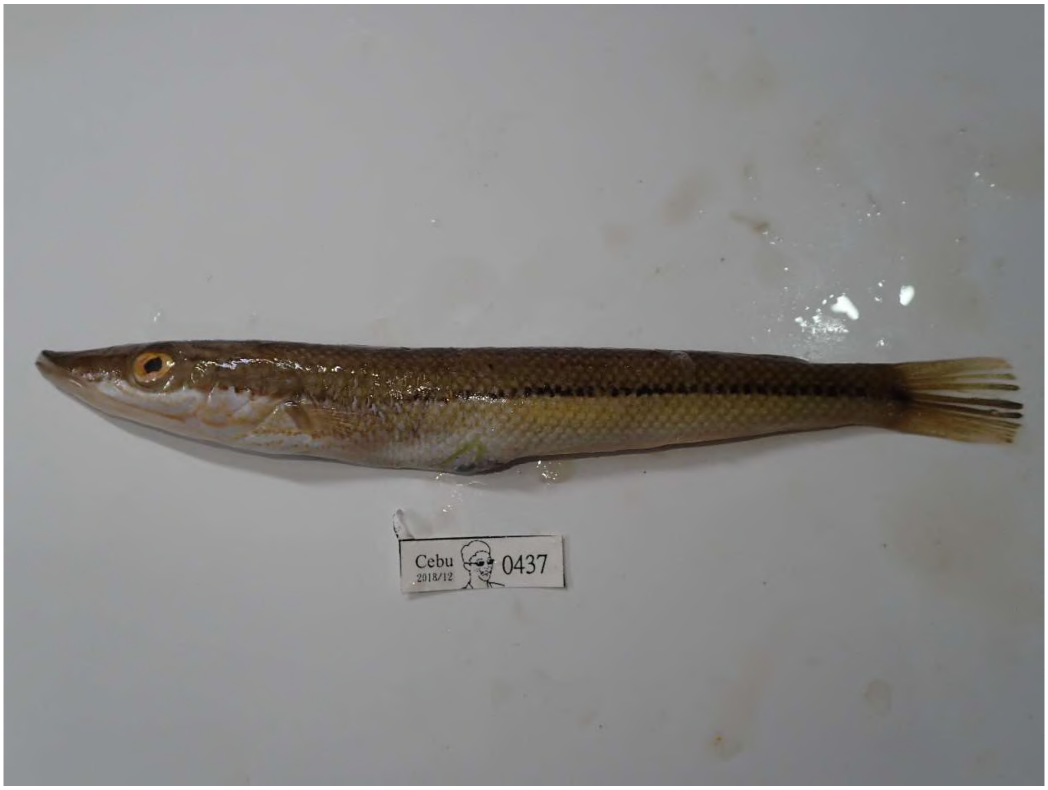

DOS 06736-1, *Cheilio inermis*, OR113921.

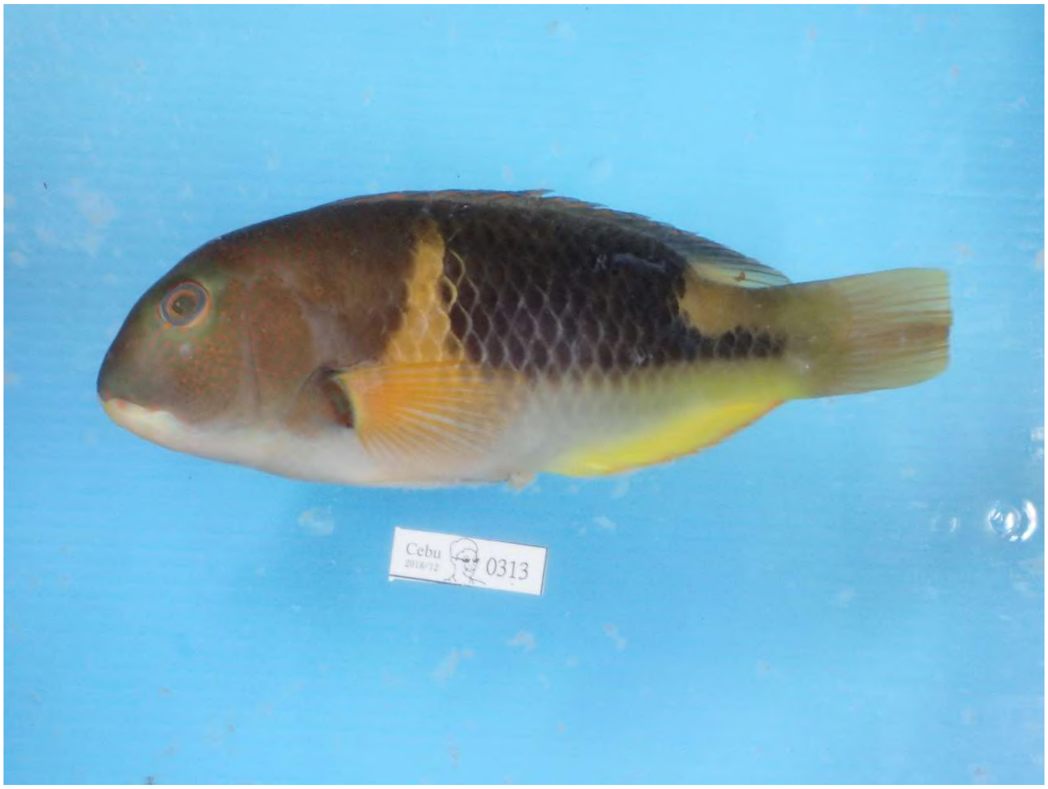

DOS 06738-1, *Choerodon anchorago*, OR113923. (specimen not preserved)

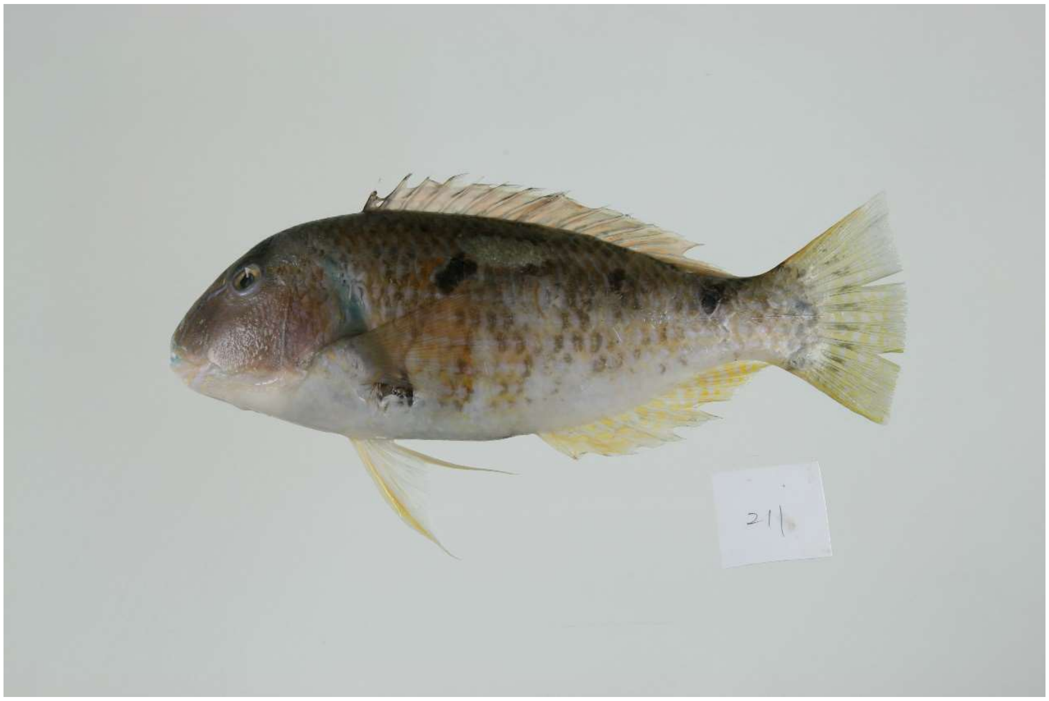

DOS 08641-1, *Choerodon oligacanthus*, OR114213.

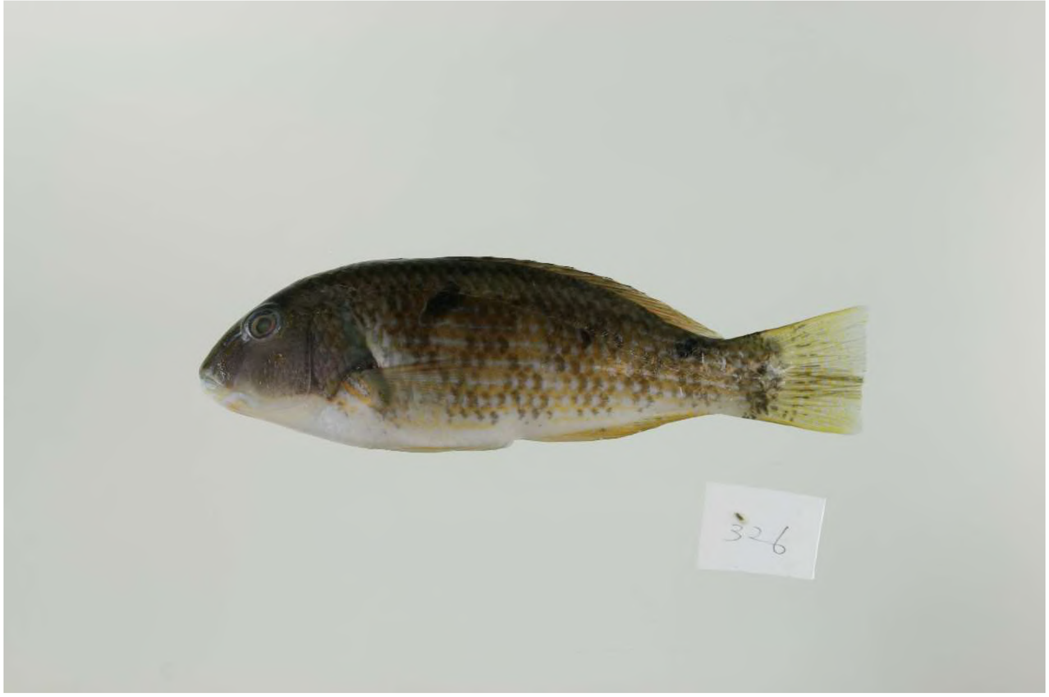

DOS 08641-2, *Choerodon oligacanthus*, OR114214.

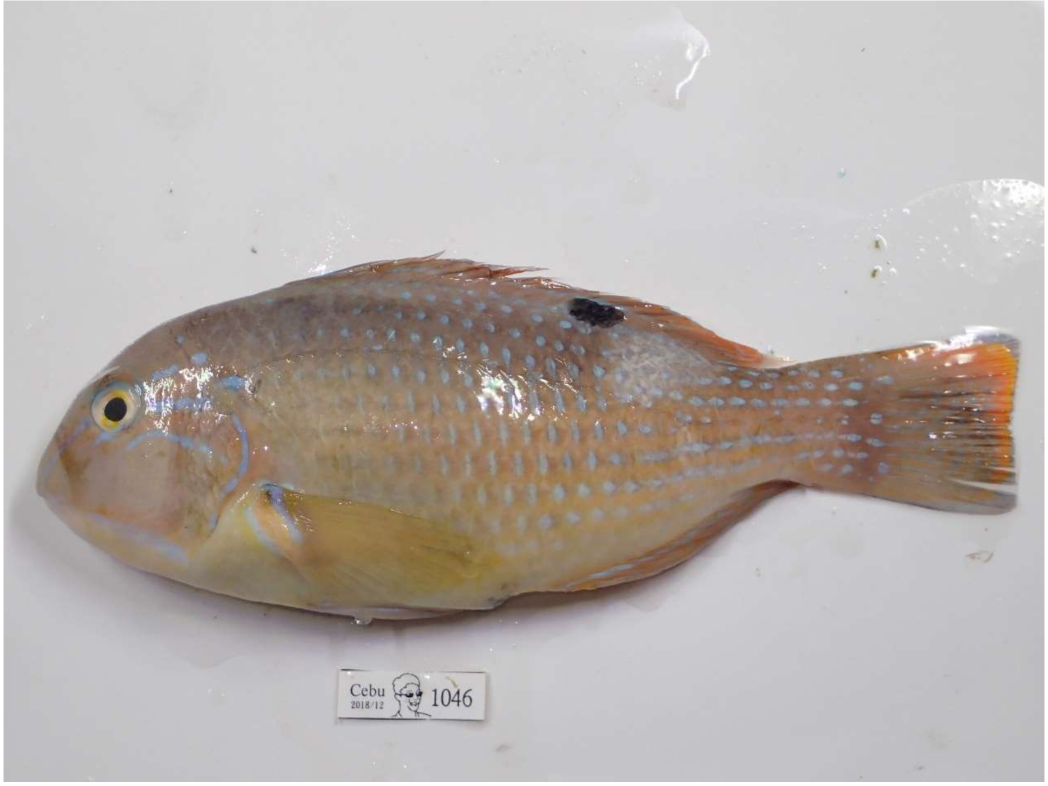

DOS 06920, *Choerodon schoenleinii*, OR114096.

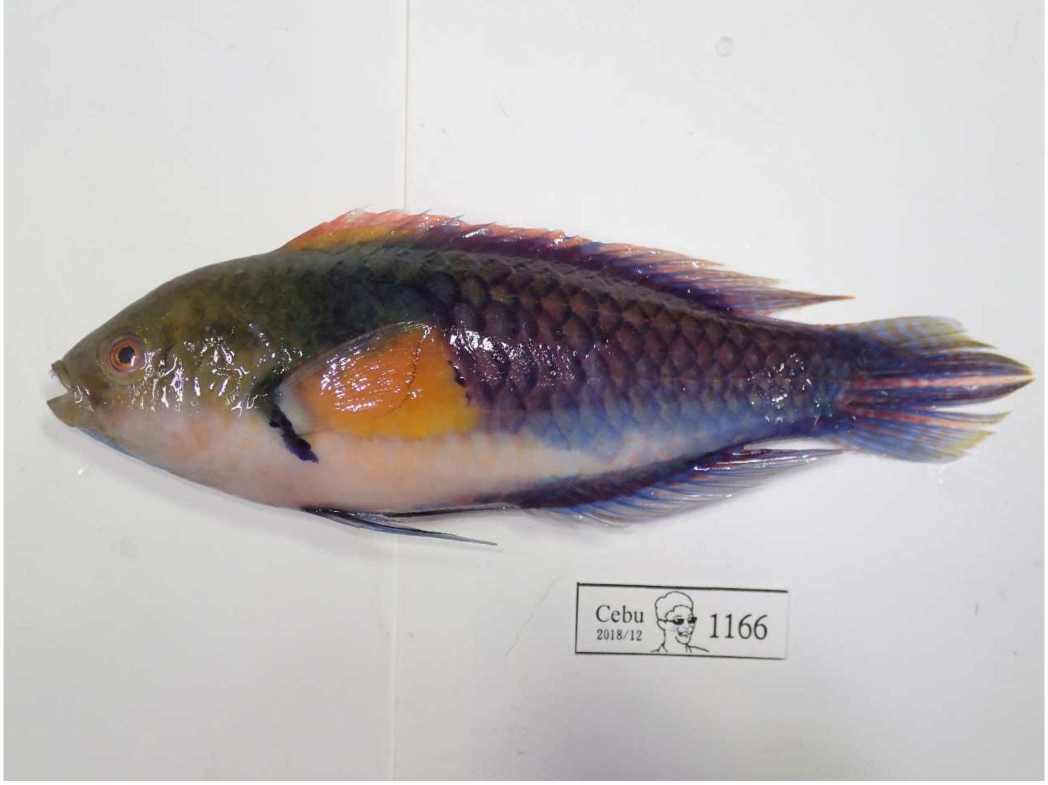

DOS 06739-1, *Cirrhilabrus cyanopleura*, OR113924.

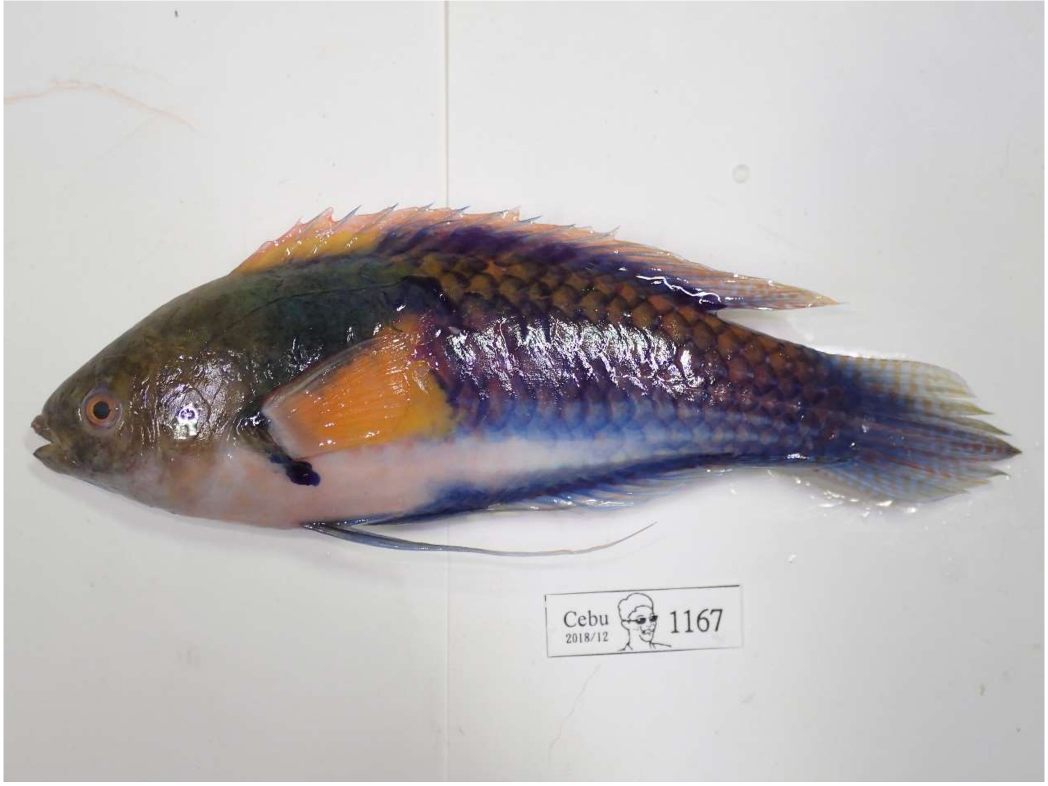

DOS 06739-2, *Cirrhilabrus cyanopleura*, OR113925.

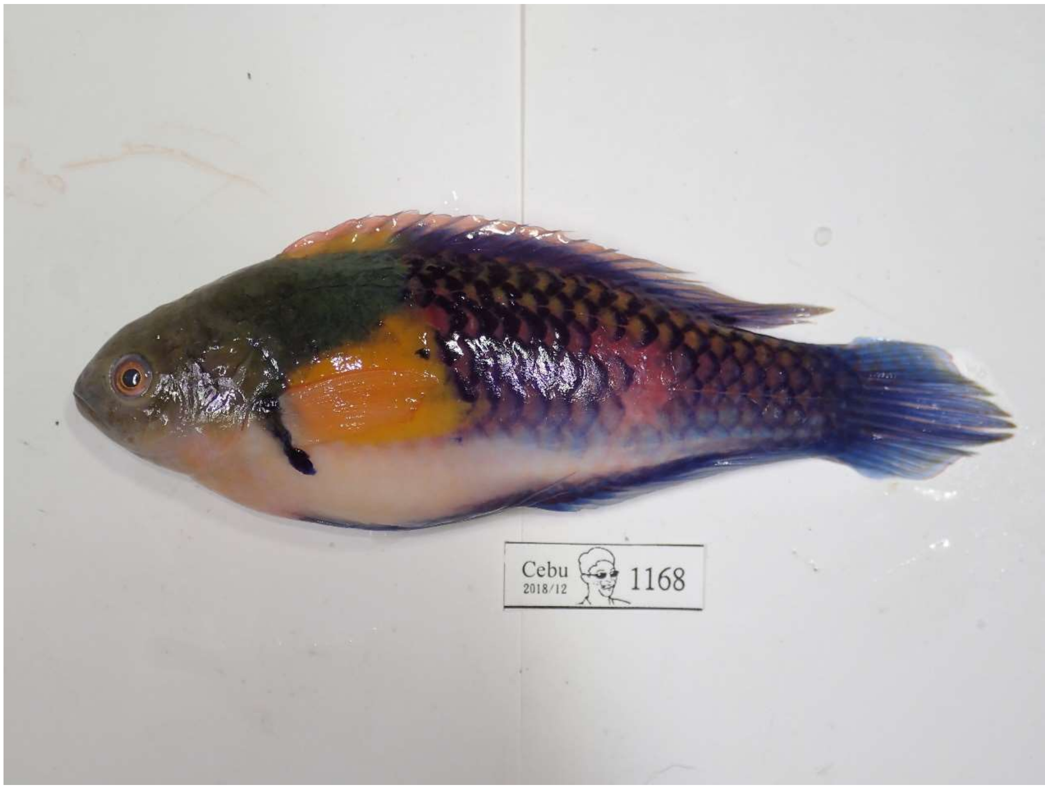

DOS 06739-3, *Cirrhilabrus cyanopleura*, OR113926.

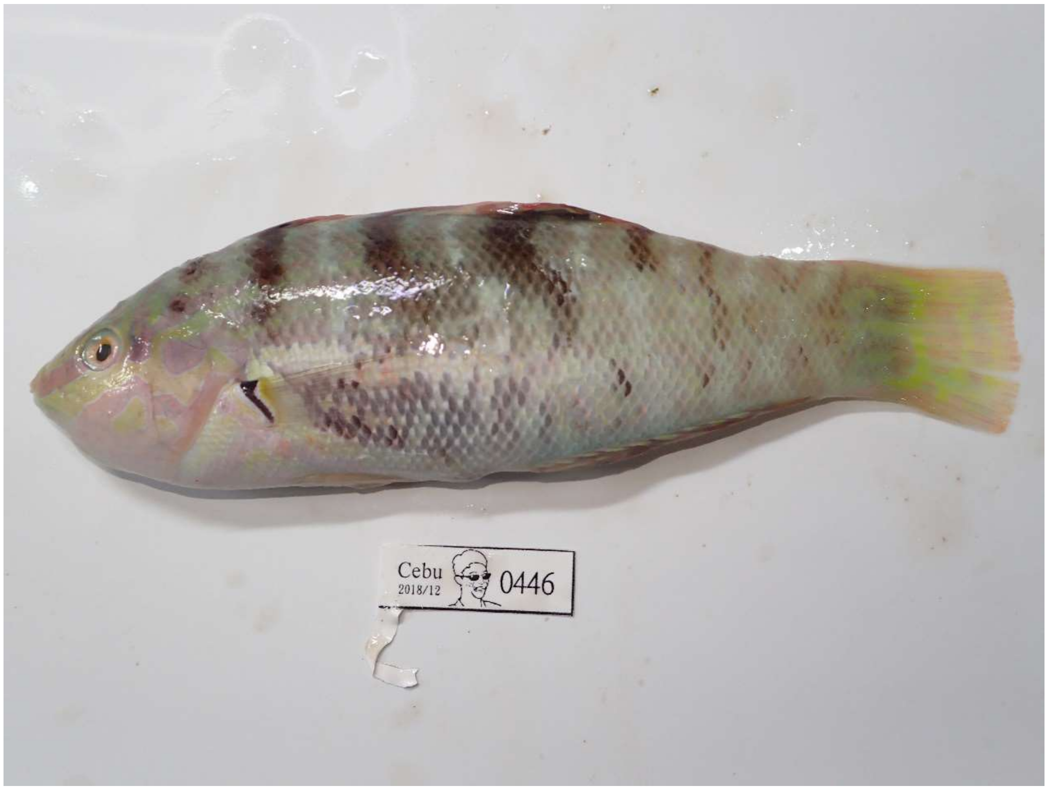

DOS 06740-1, *Coris batuensis*, OR113927.

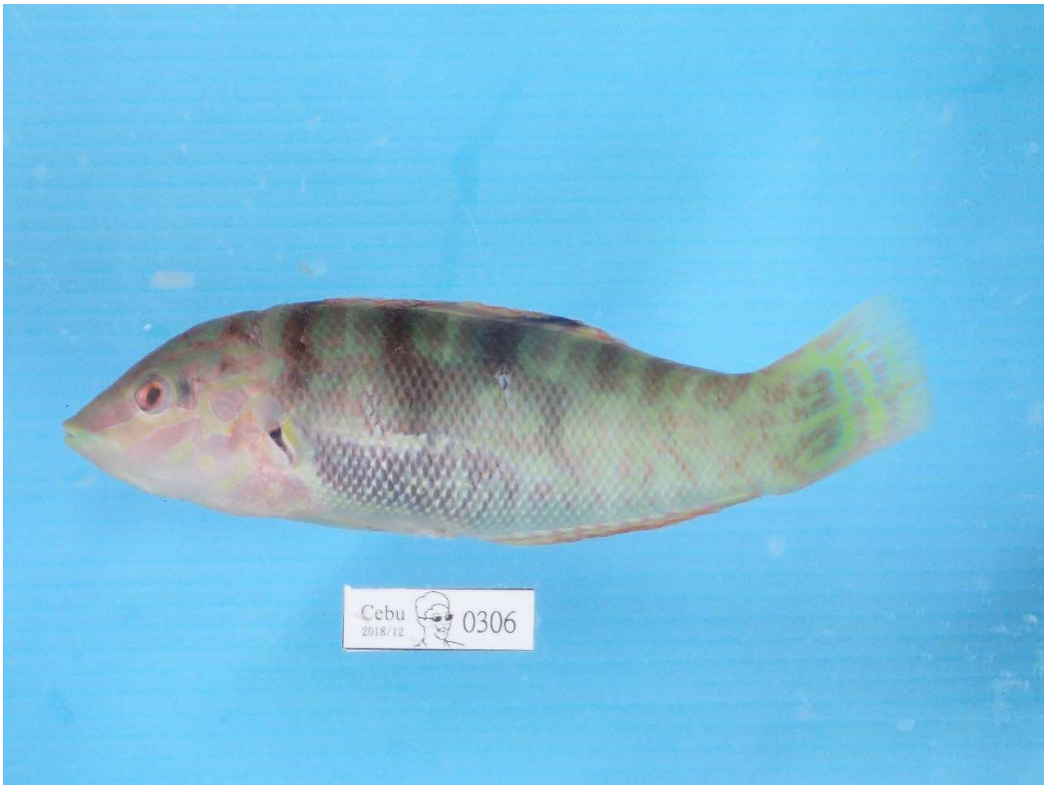

DOS 06745-2, *Coris batuensis*, OR113932.

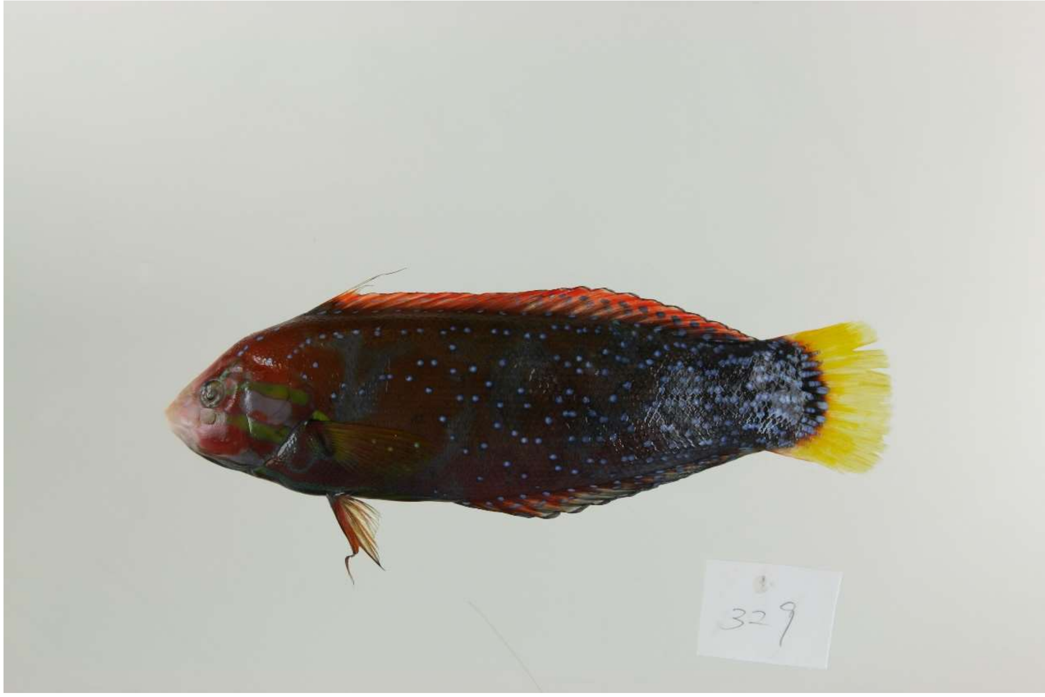

DOS 08642, *Coris gaimard*, OR114215.

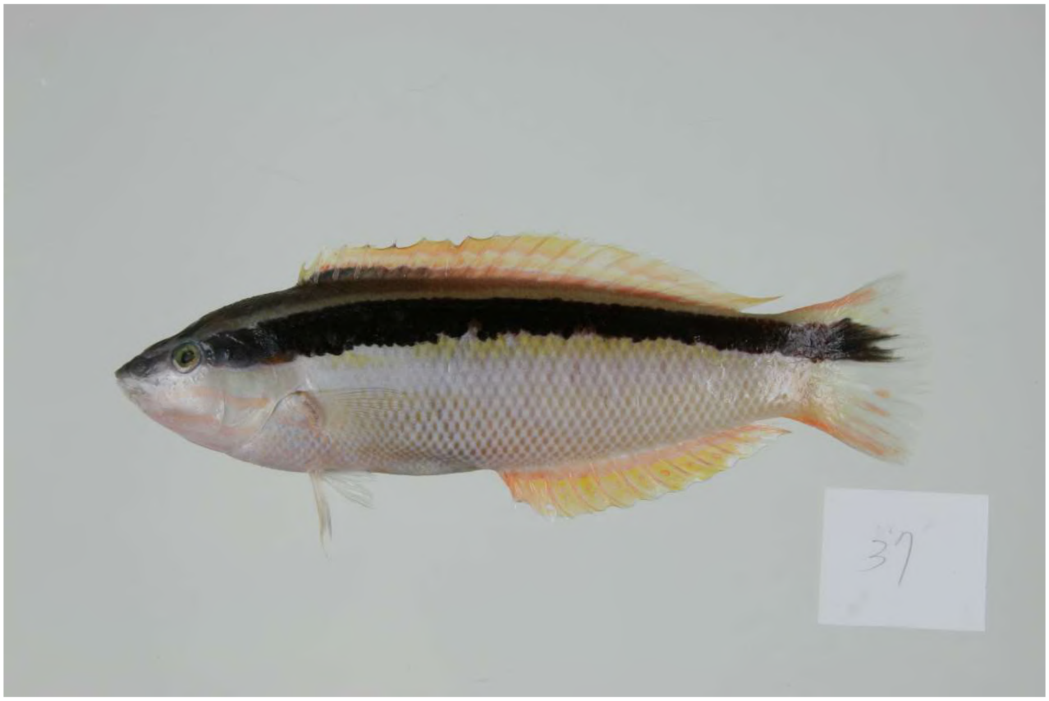

DOS 08643, *Coris pictoides*, OR114216.

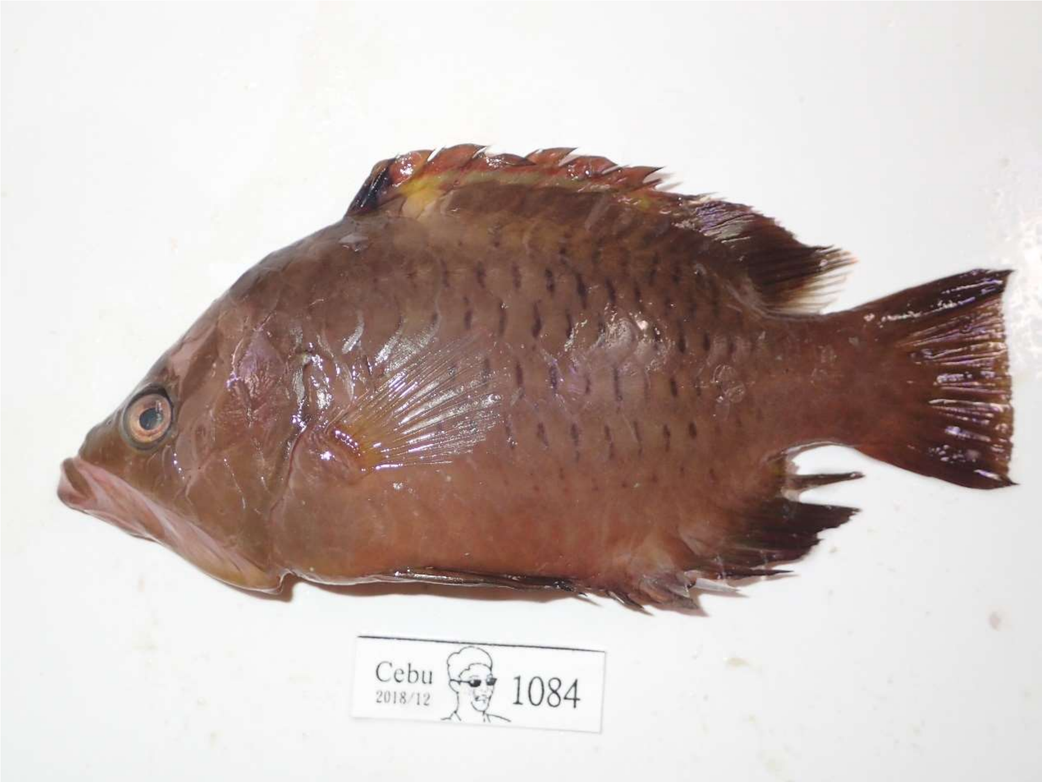

DOS 06742-1, *Epibulus brevis*, OR113929.

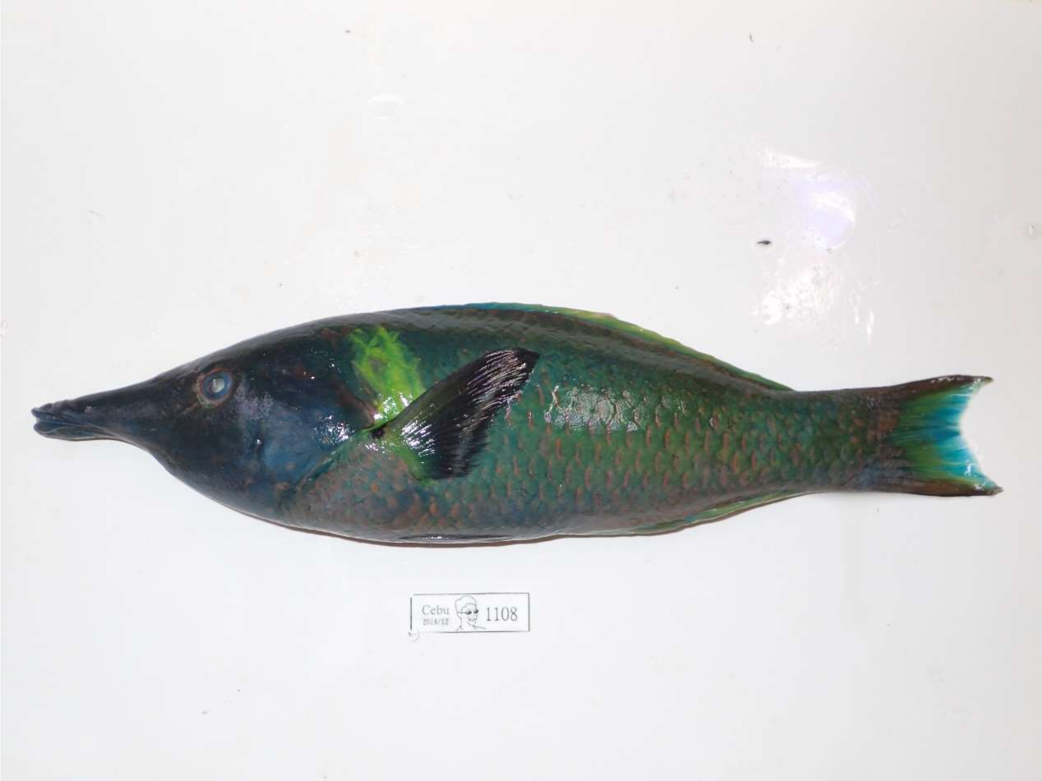

DOS 06743, *Gomphosus varius*, OR113930.

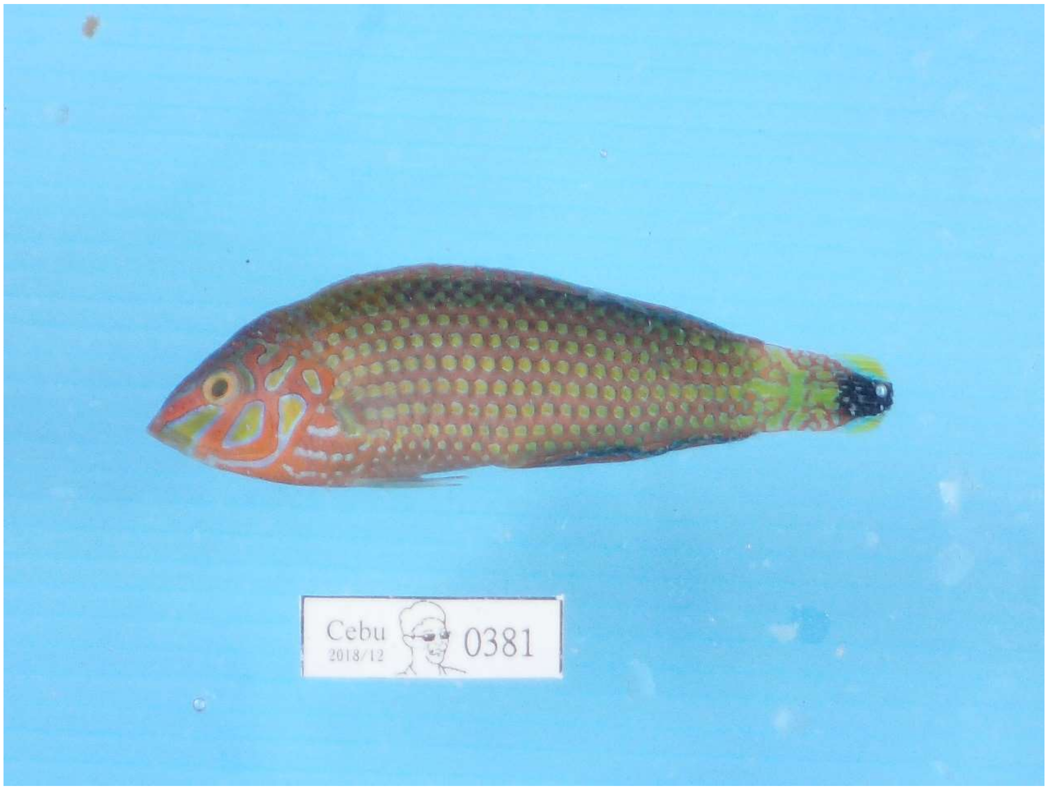

DOS 06744, *Halichoeres argus*, OR113931.

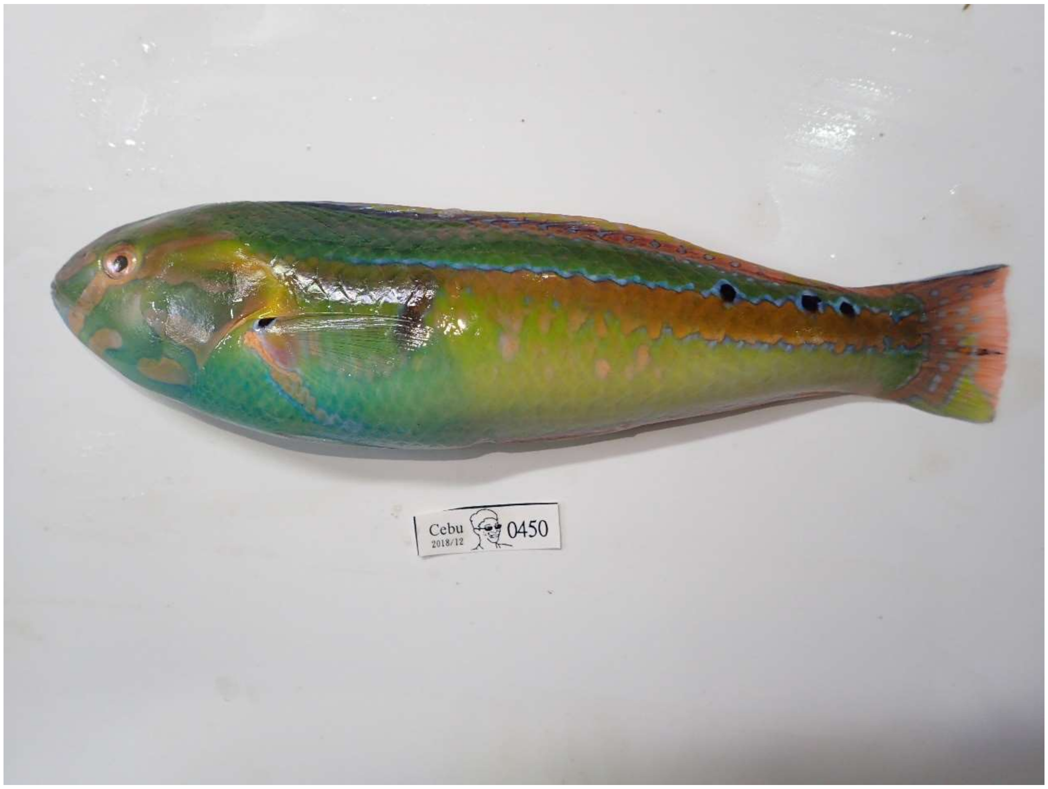

DOS 06751, *Halichoeres hartzfeldii*, OR113937.

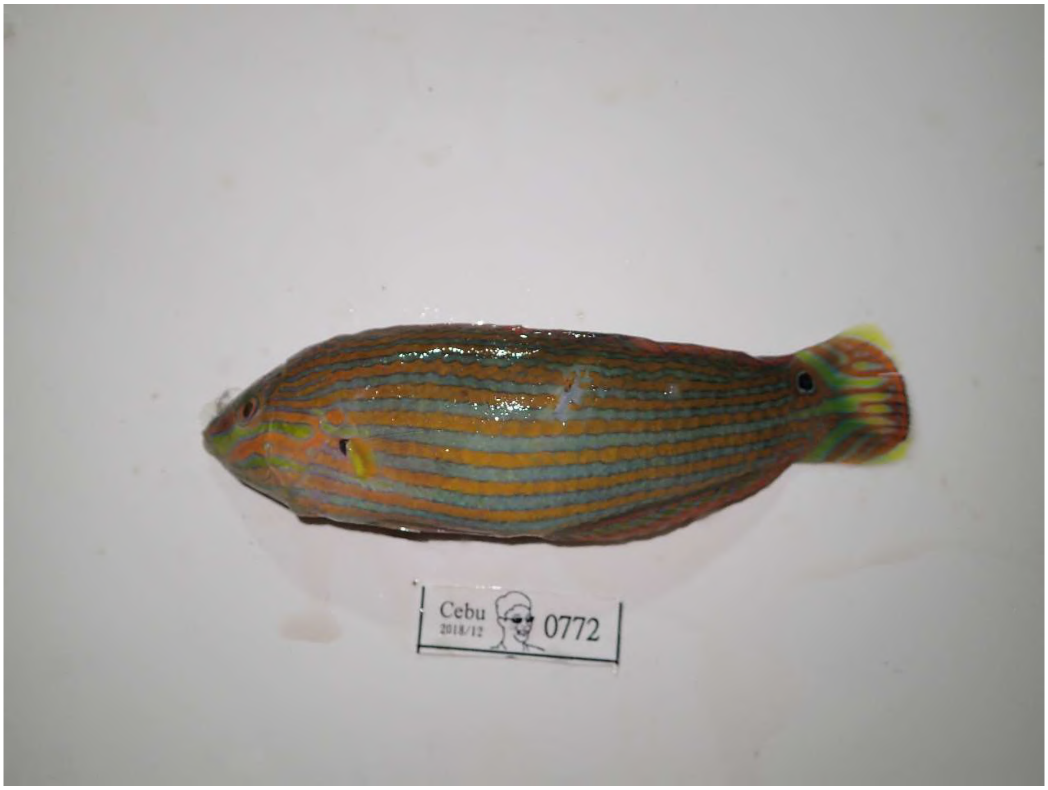

DOS 06746-1, *Halichoeres melanurus*, OR113933.

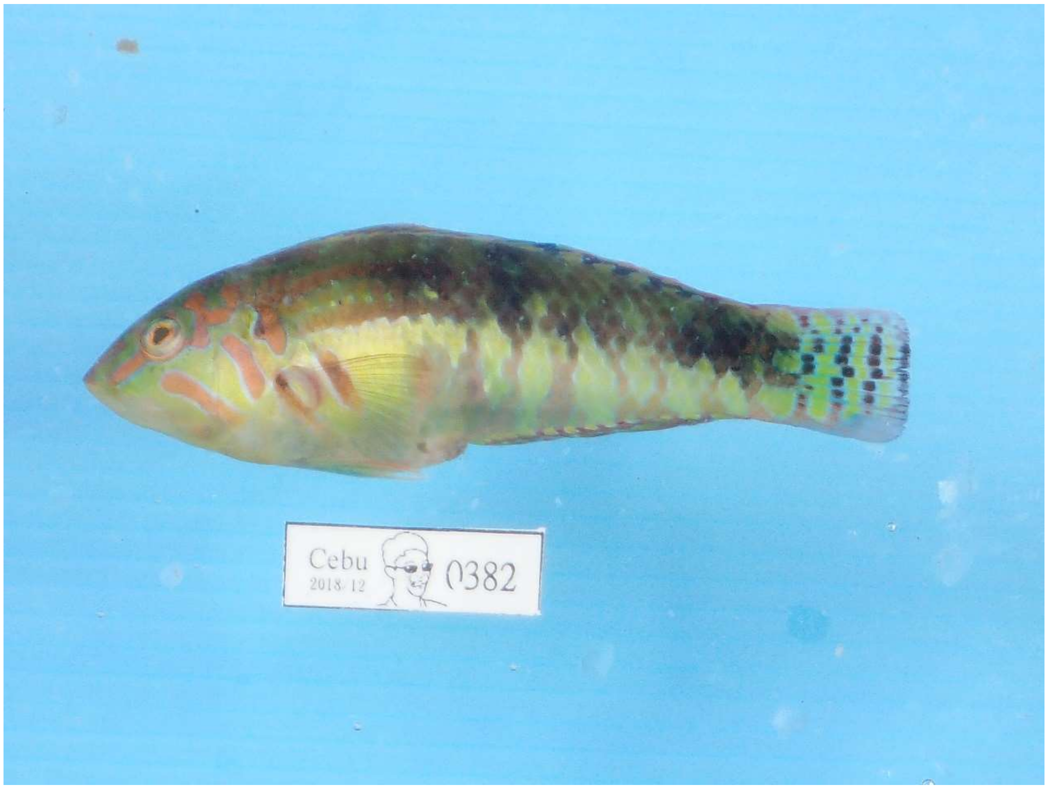

DOS 06747-1, *Halichoeres nebulosus*, OR113934.

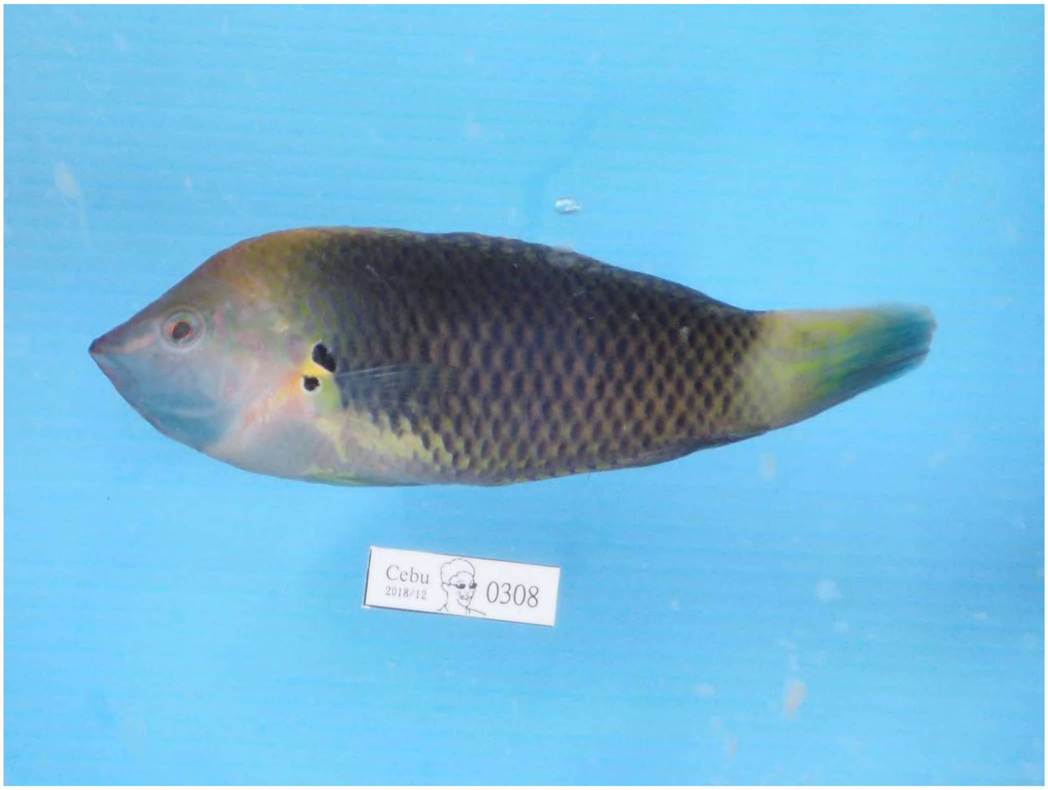

DOS 06741-1, *Halichoeres podostigma*, OR113928.

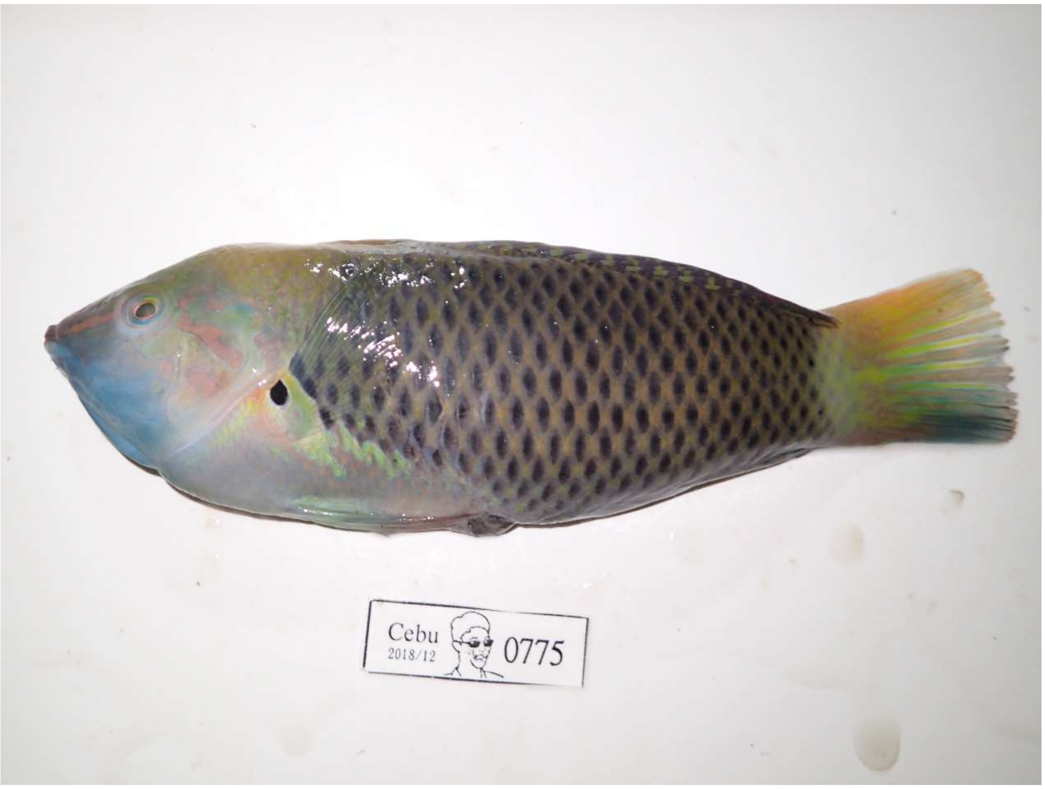

DOS 06749-1, *Halichoeres podostigma*, OR113936.

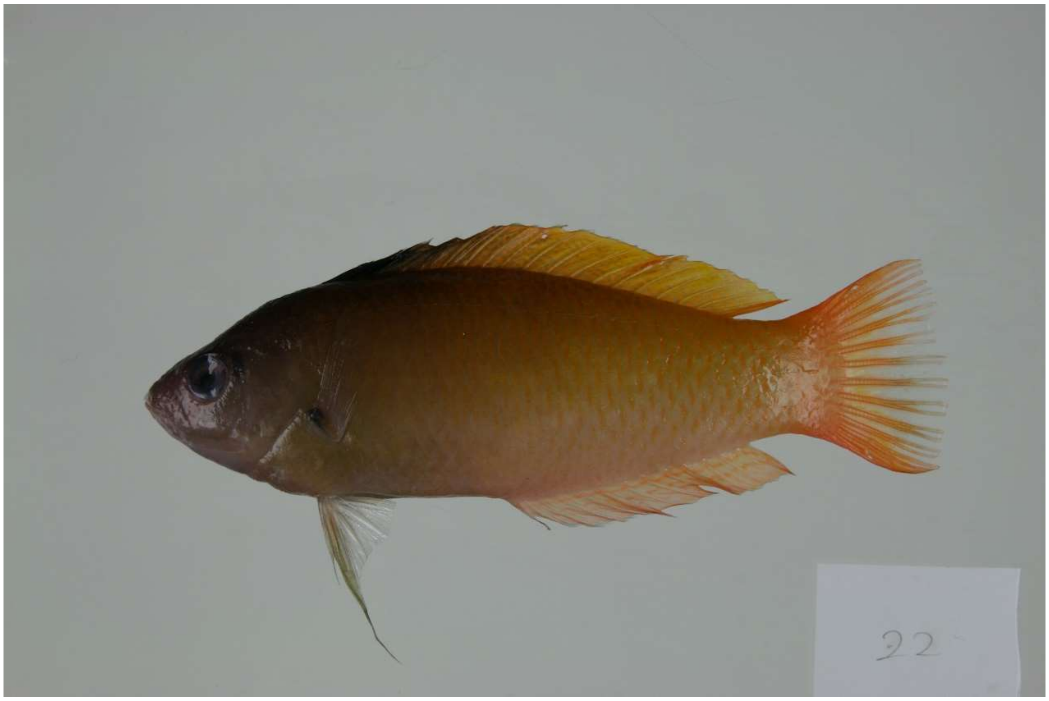

DOS 08644, *Halichoeres prosopeion*, OR114217.

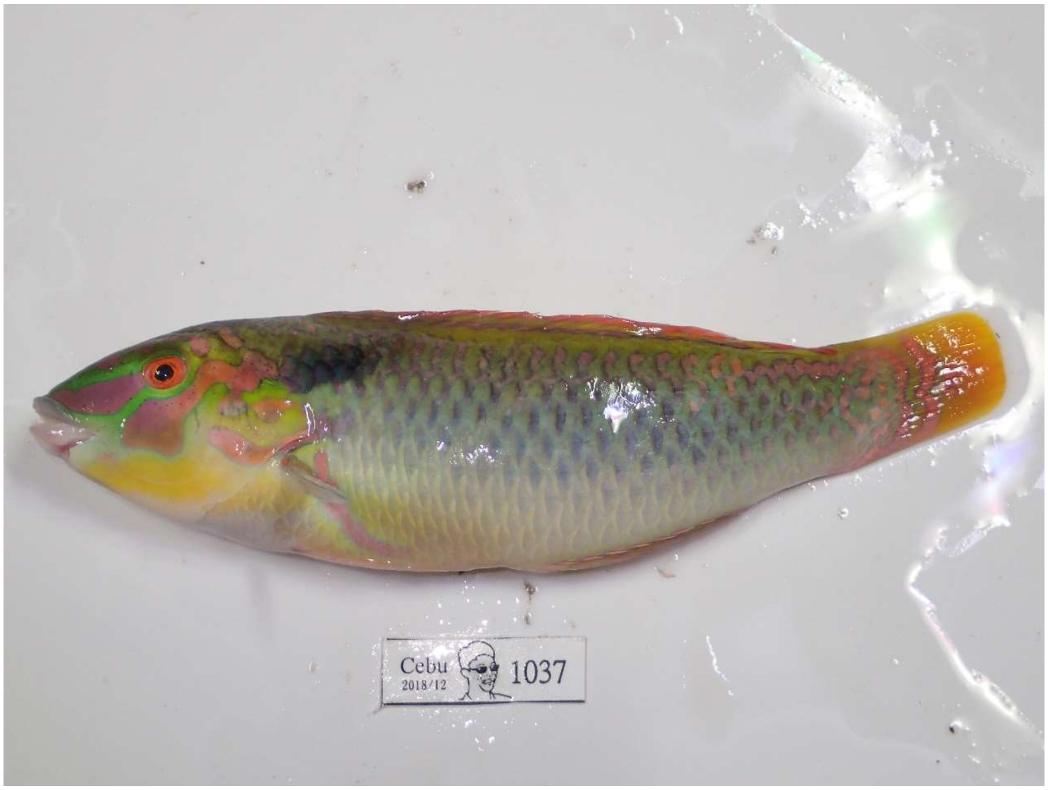

DOS 06737-1, *Halichoeres scapularis*, OR113922.

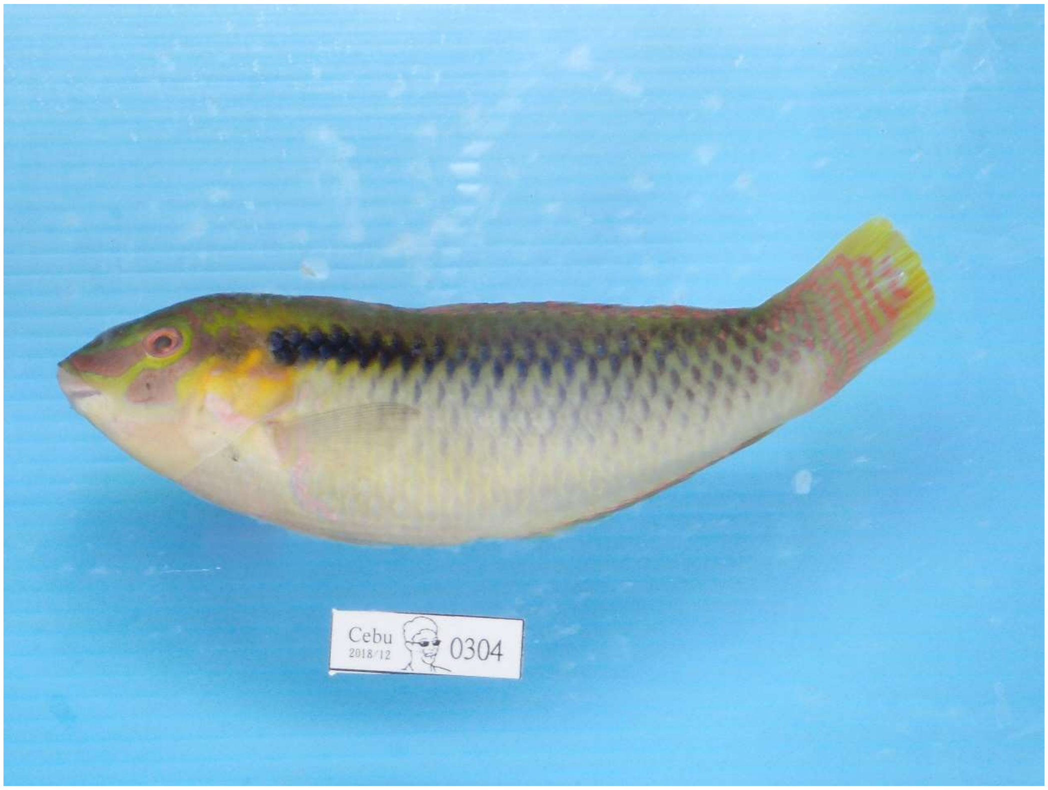

DOS 06748-1, *Halichoeres scapularis*, OR113935. (specimen not preserved)

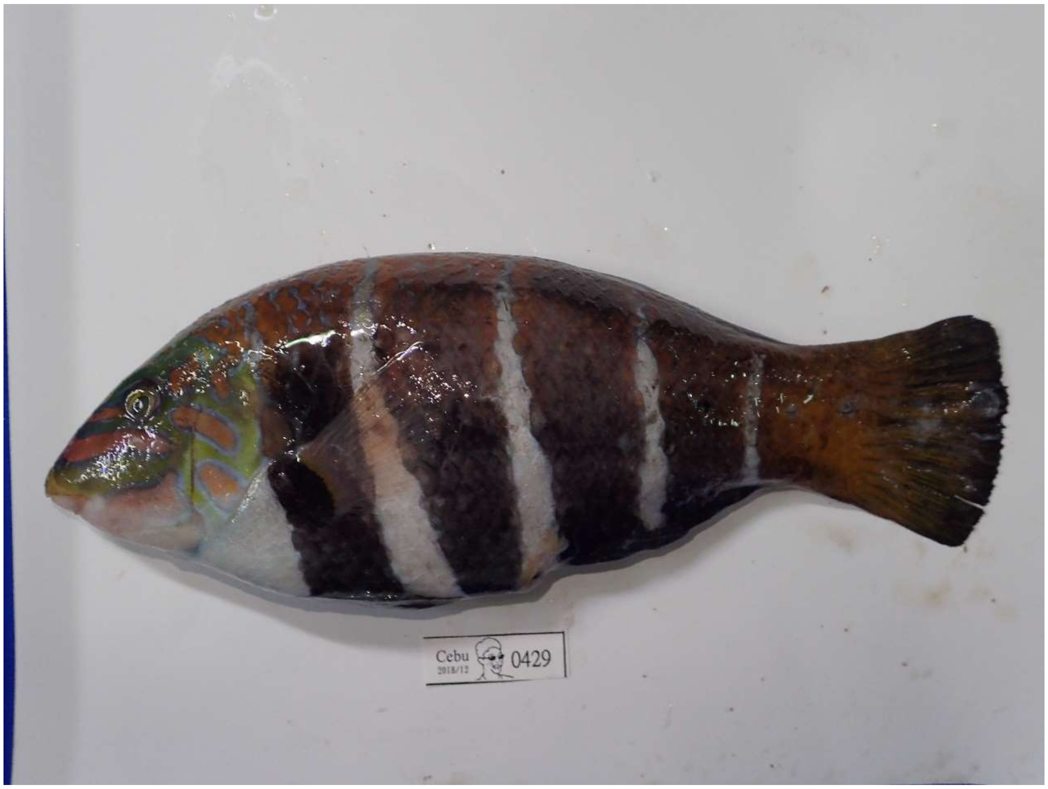

DOS 06752, *Hemigymnus fasciatus*, OR113938.

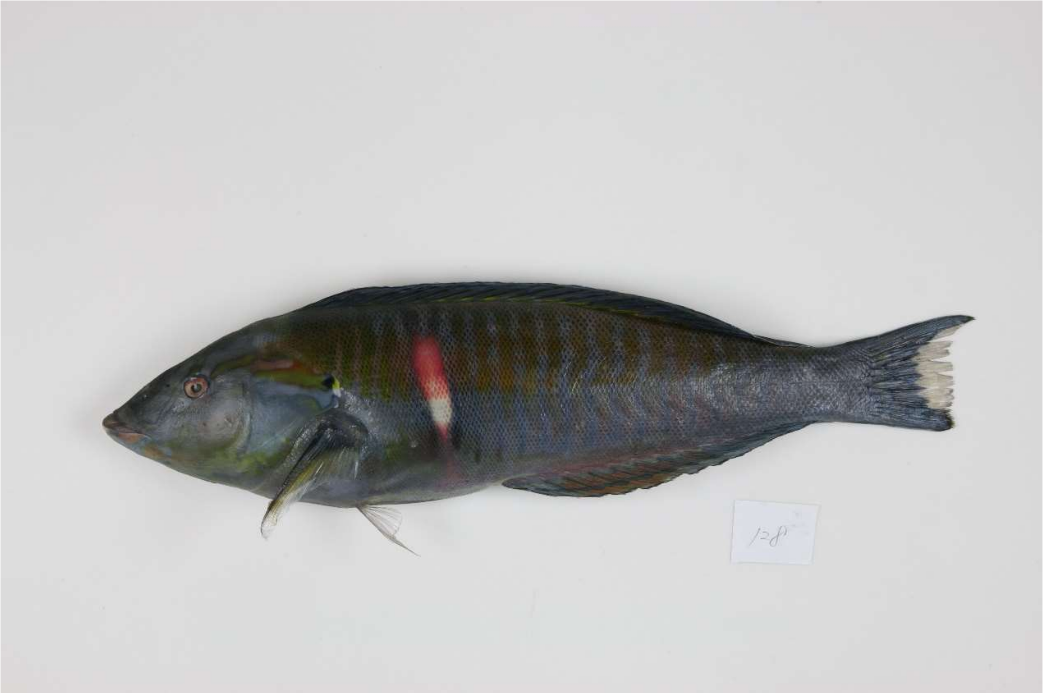

DOS 08645, *Hologymnosus doliatus*, OR114218.

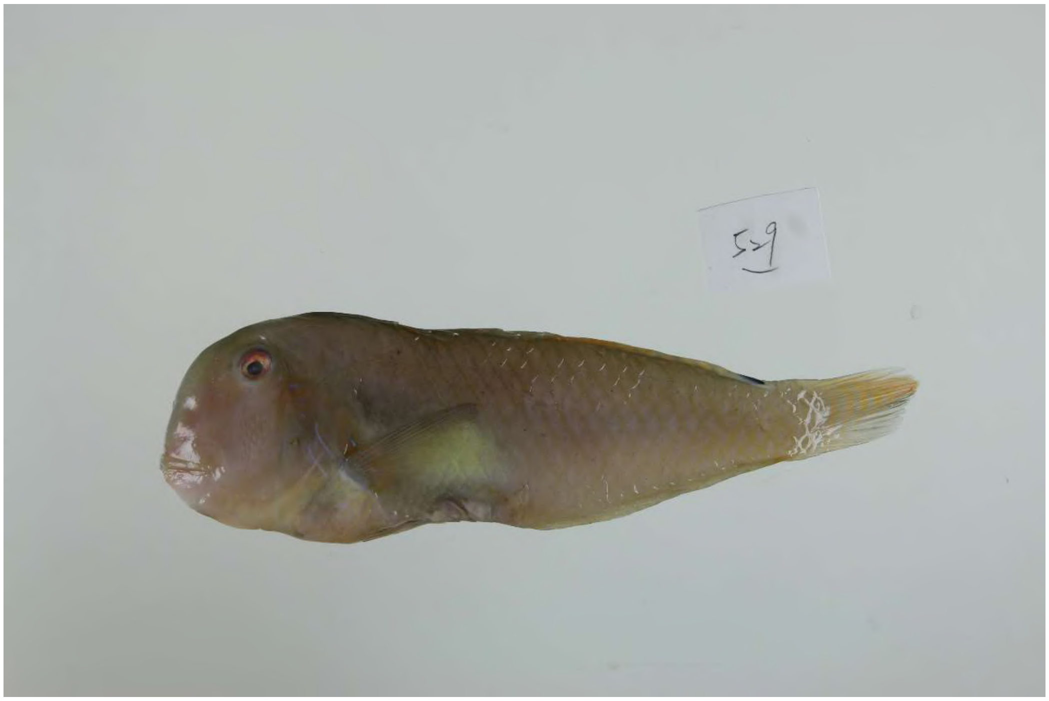

DOS 08646-1, *Iniistius* sp., OR114219.

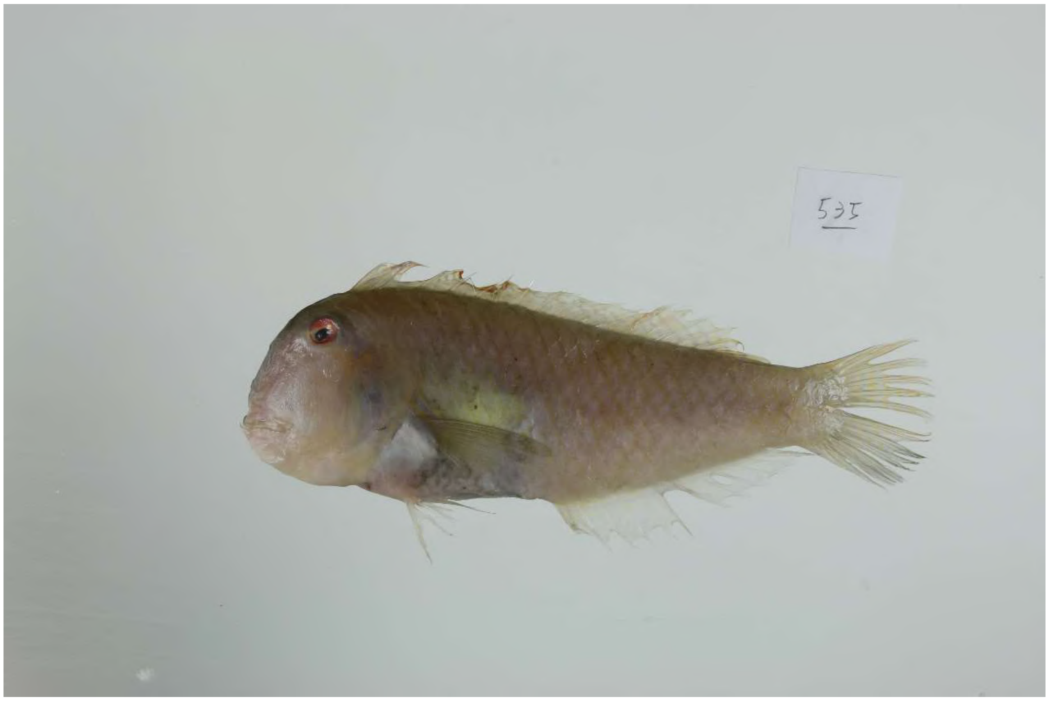

DOS 08646-2, *Iniistius* sp., OR114220.

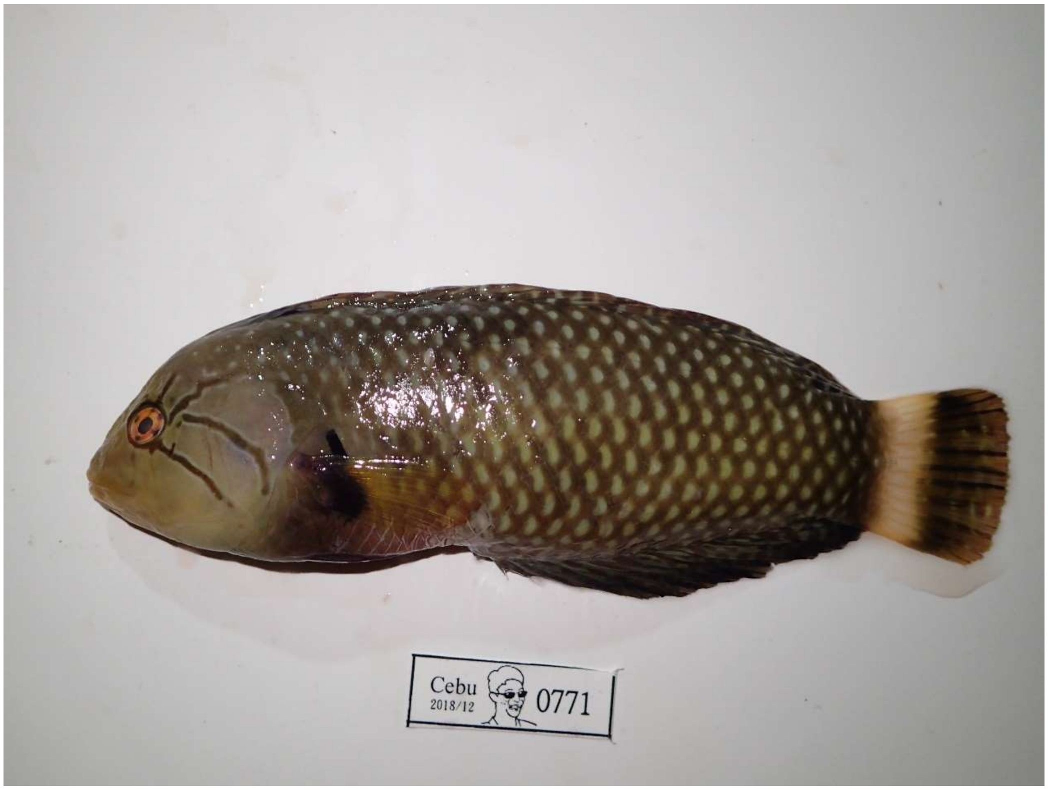

DOS 06753, *Novaculichthys taeniourus*, OR113939.

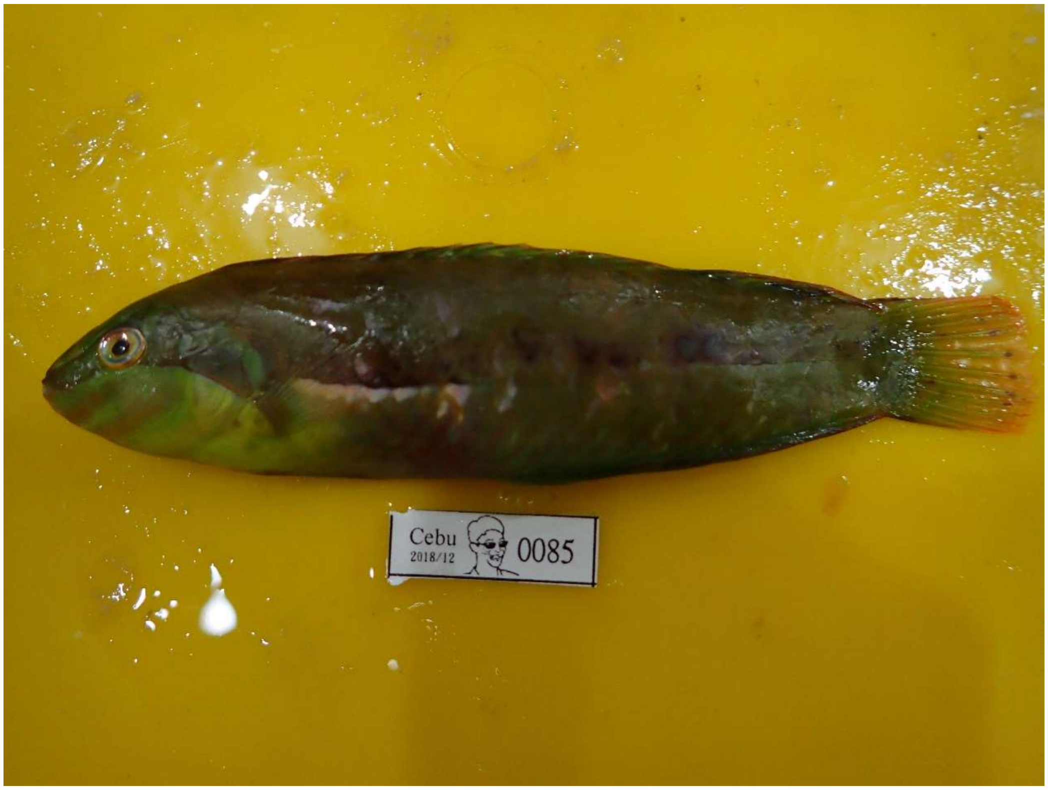

DOS 06763, *Novaculoides macrolepidotus*, OR113948. (specimen not preserved)

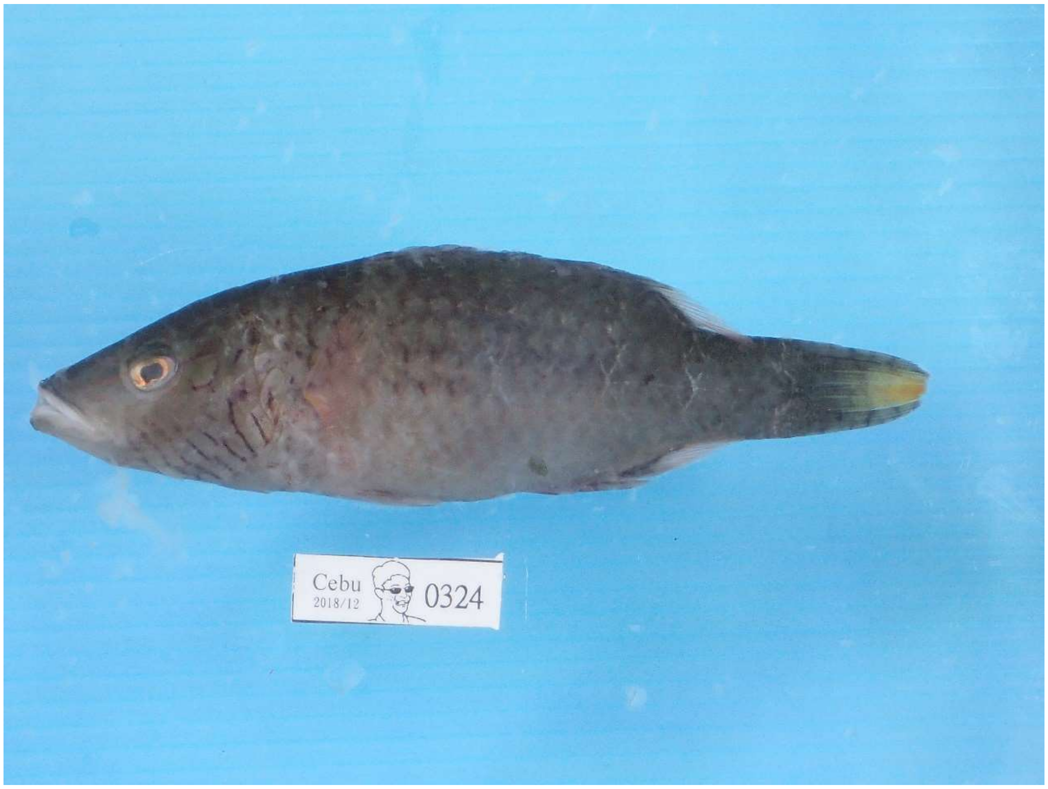

DOS 06755, *Oxycheilinus digramma*, OR113941.

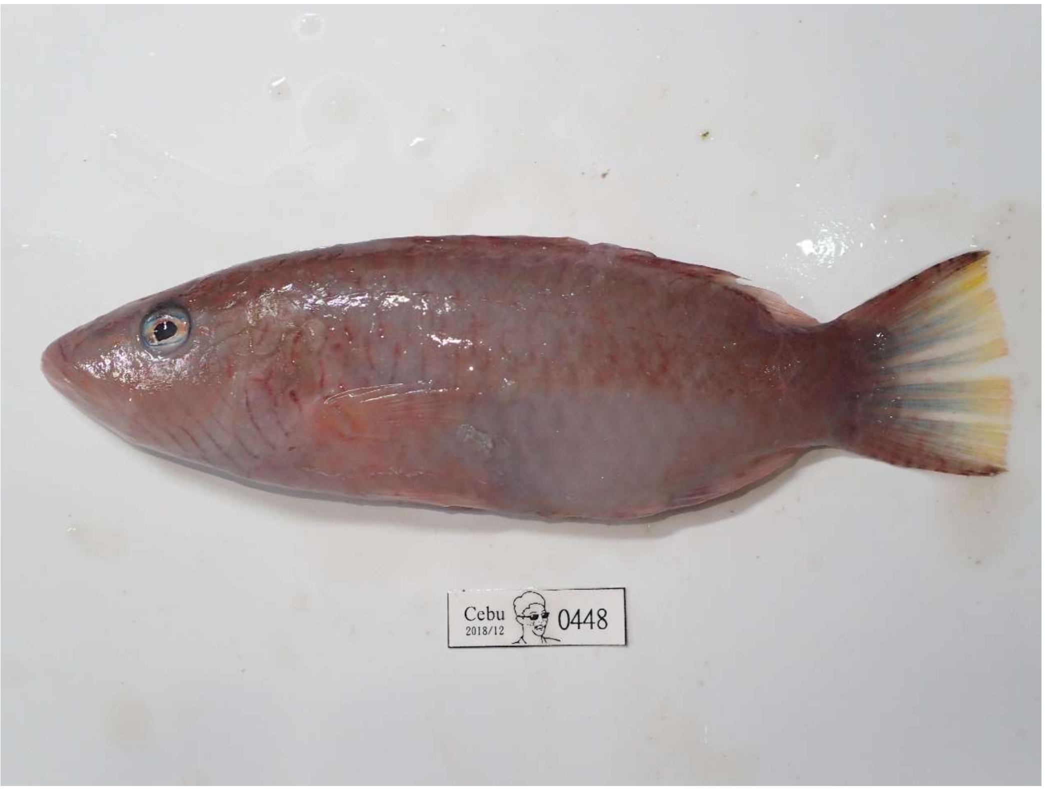

DOS 06757, *Oxycheilinus digramma*, OR113942.

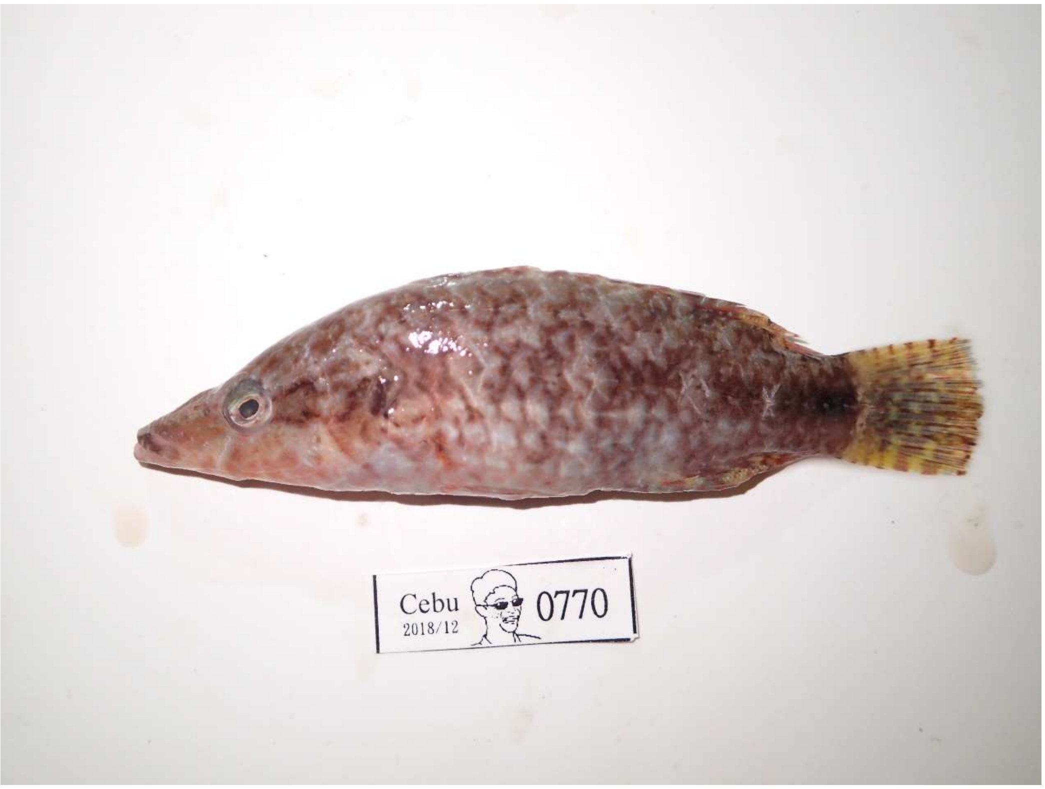

DOS 06754-3, *Oxycheilinus* sp., OR113940.

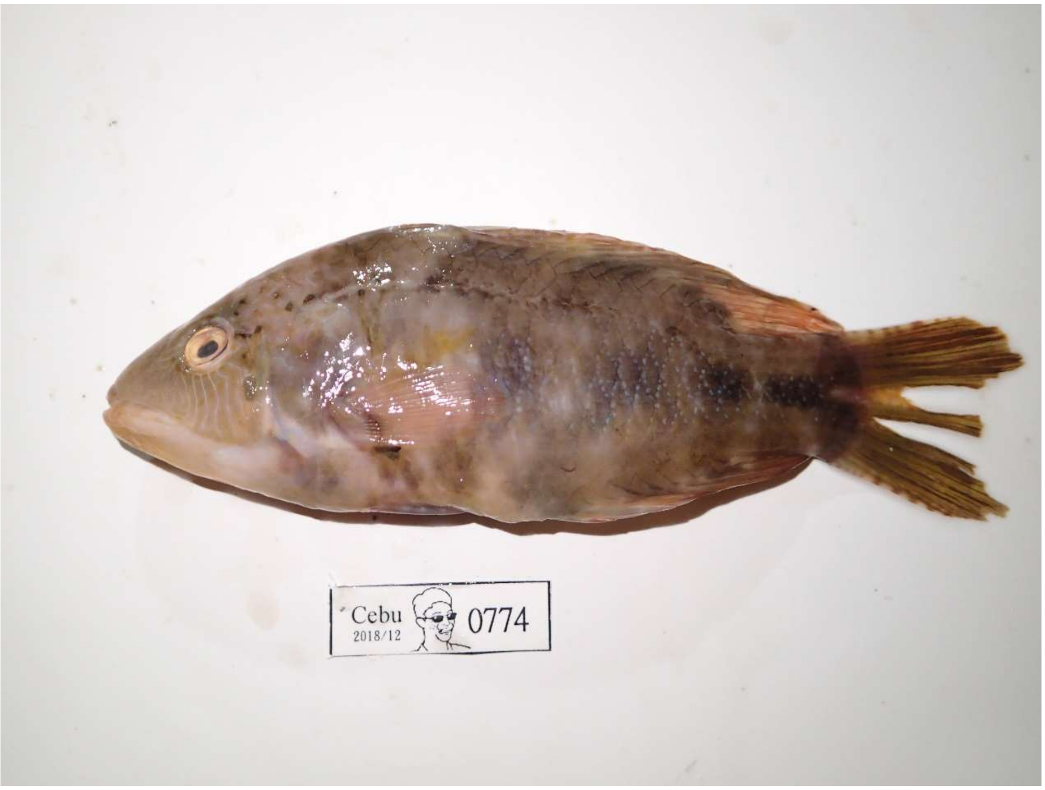

DOS 06758, *Pteragogus* sp., OR113943.

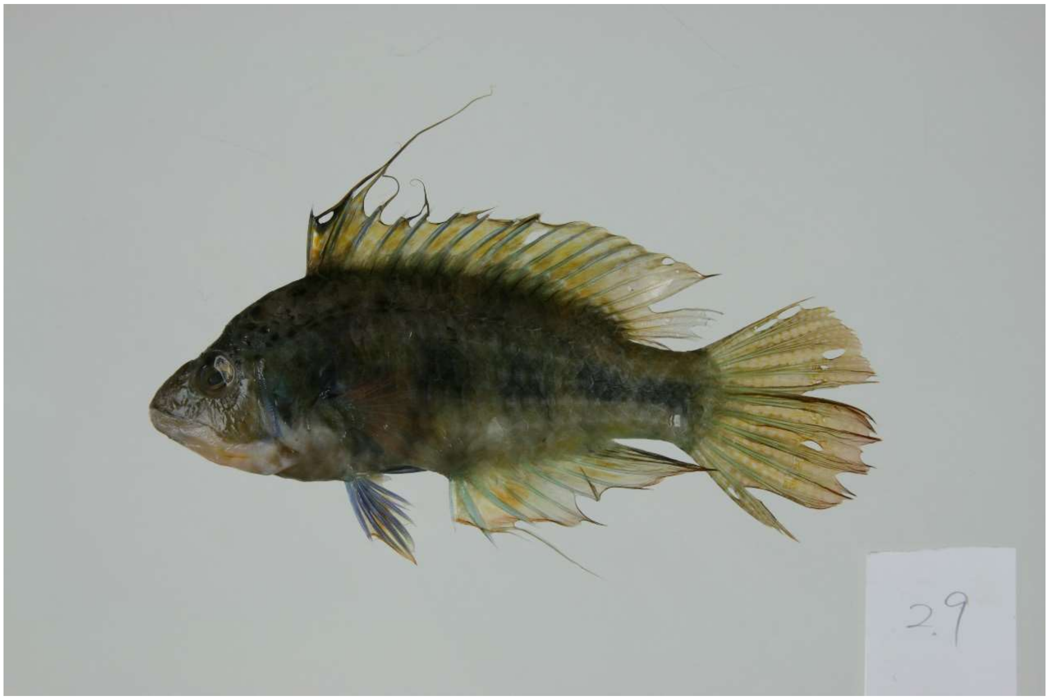

DOS 08647, *Pteragogus* sp., OR114221.

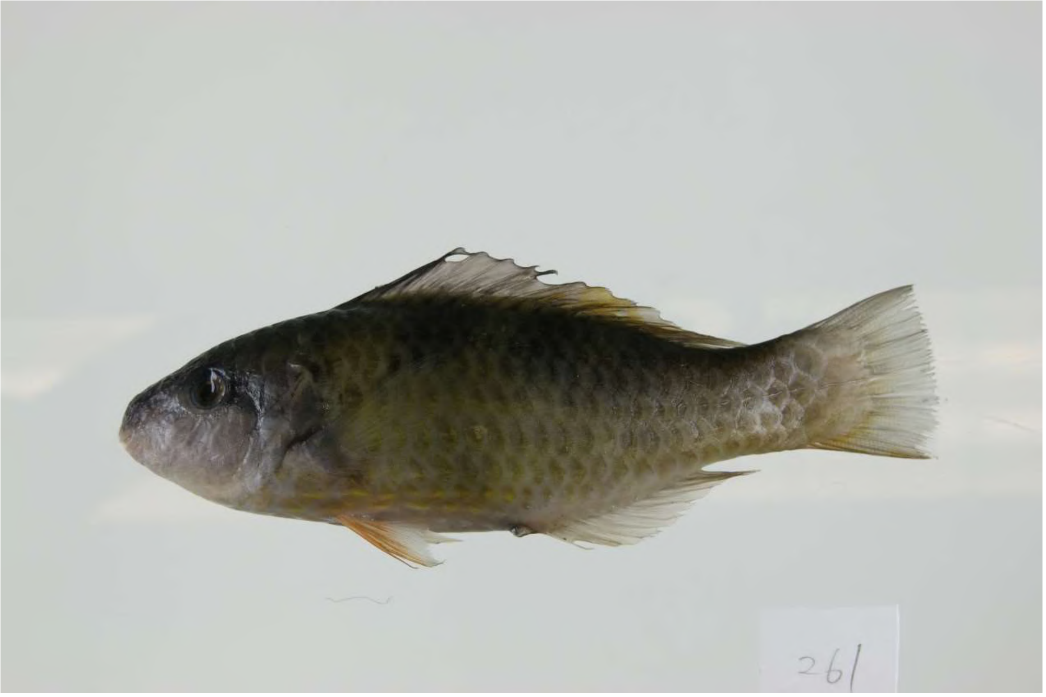

DOS 08649, *Scarus dimidiatus*, OR114224.

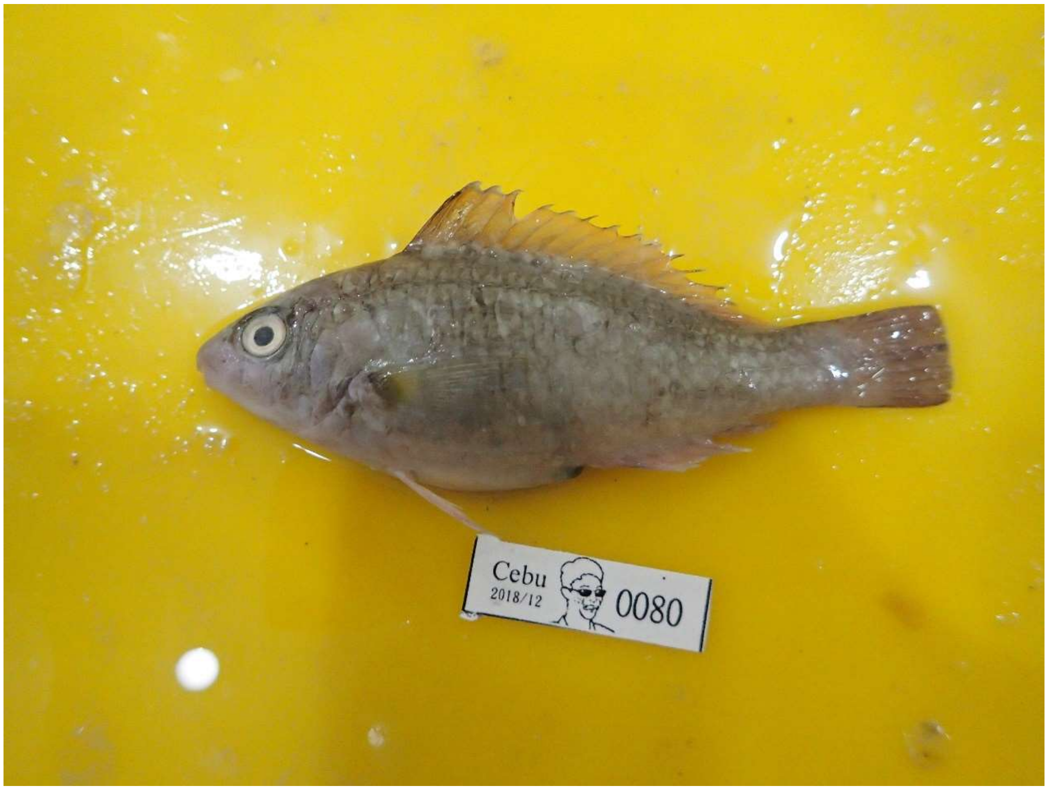

DOS 06921, *Scarus flavipectoralis*, OR114097.

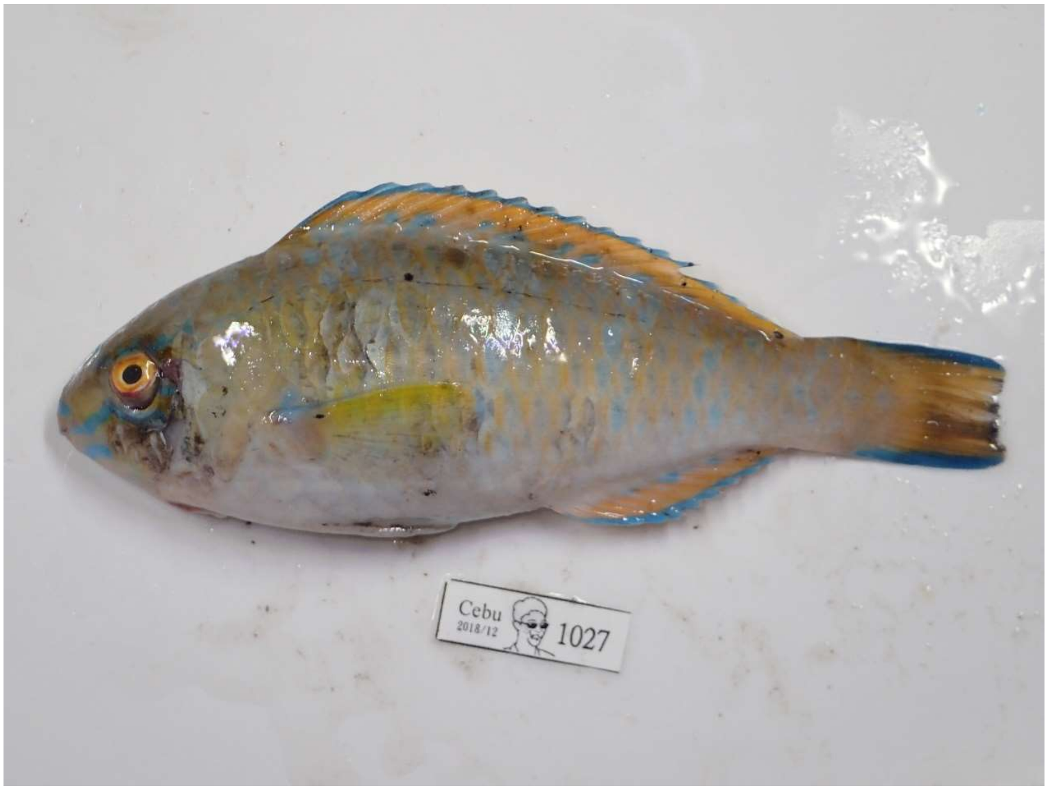

DOS 06917, *Scarus ghobban*, OR114093.

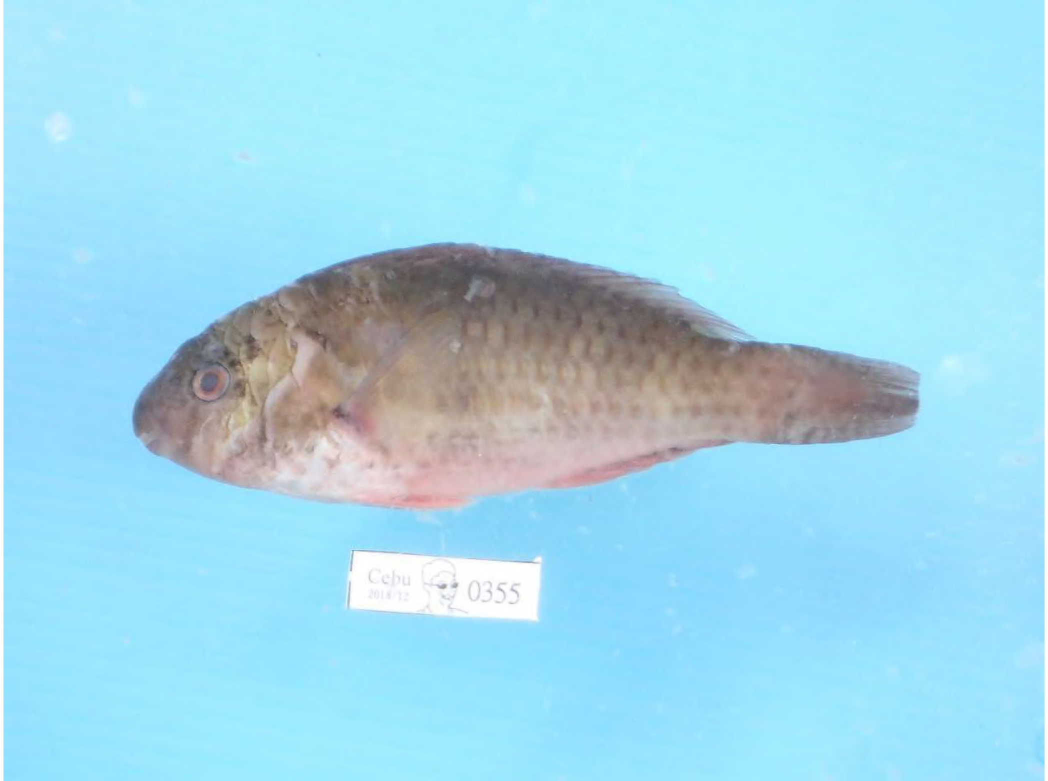

DOS 06919-4, *Scarus globiceps*, OR114095.

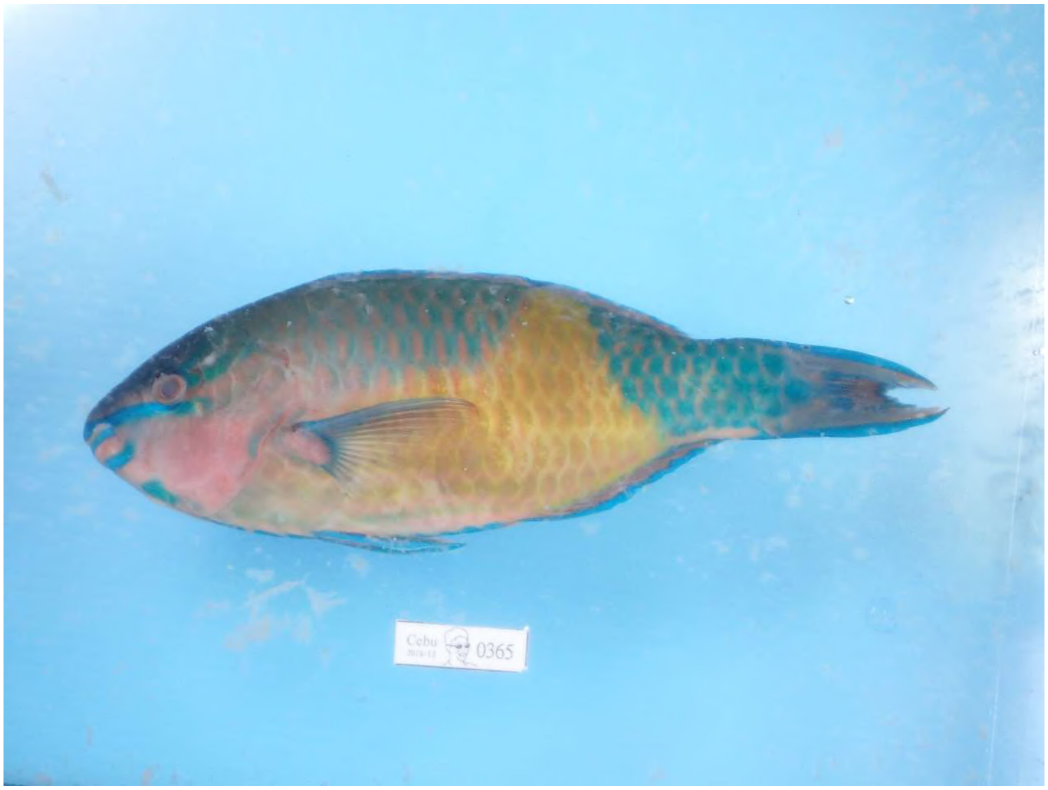

DOS 06918-1, *Scarus psittacus*, OR114094. (specimen not preserved)

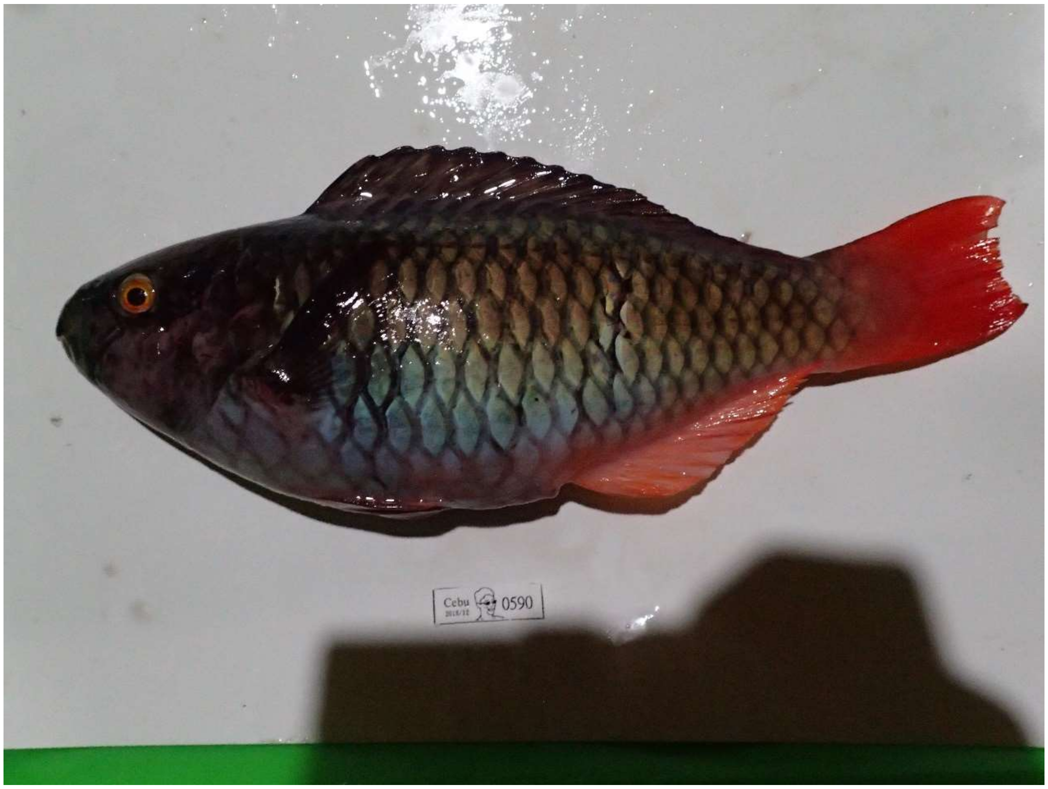

DOS 06916-1, *Scarus tricolor*, OR114092.

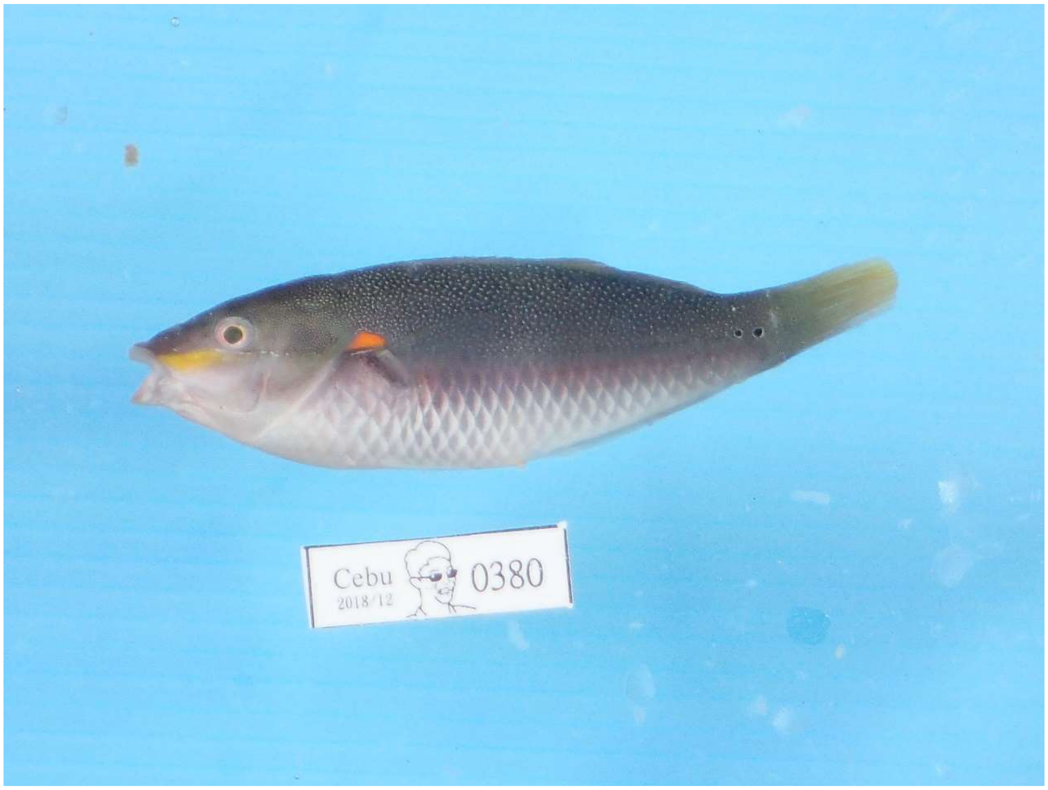

DOS 06759-1, *Stethojulis bandanensis*, OR113944. (specimen not preserved)

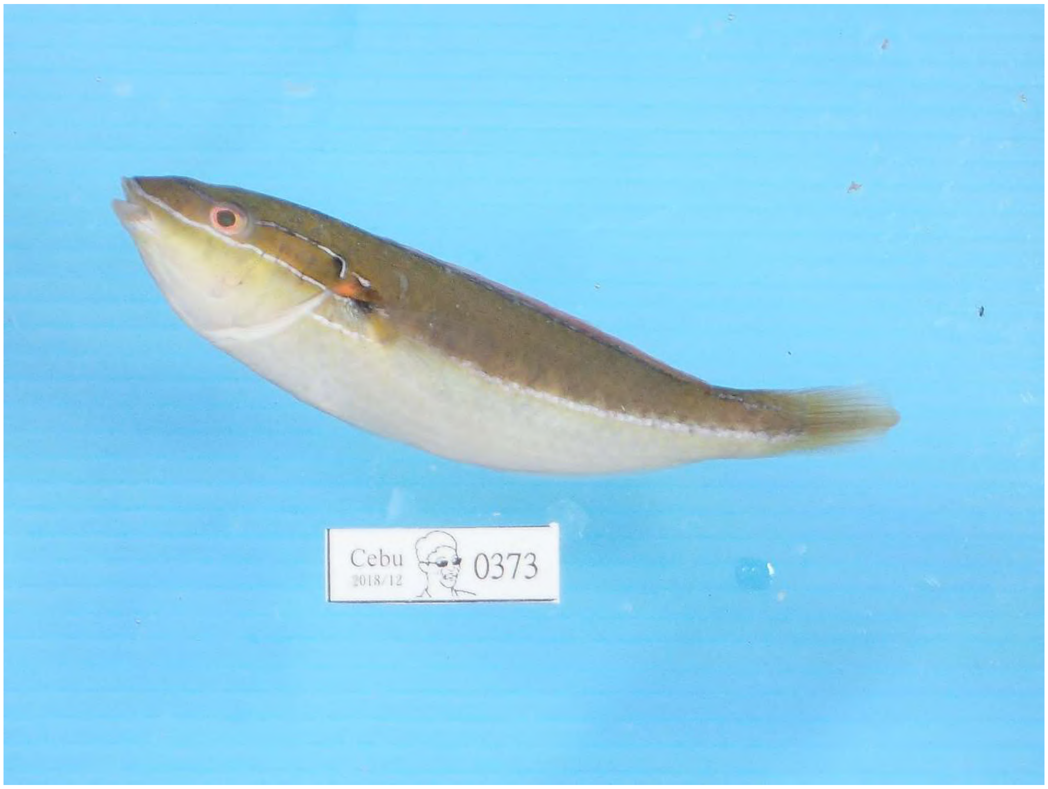

DOS 06760-1, *Stethojulis interrupta*, OR113945. (specimen not preserved)

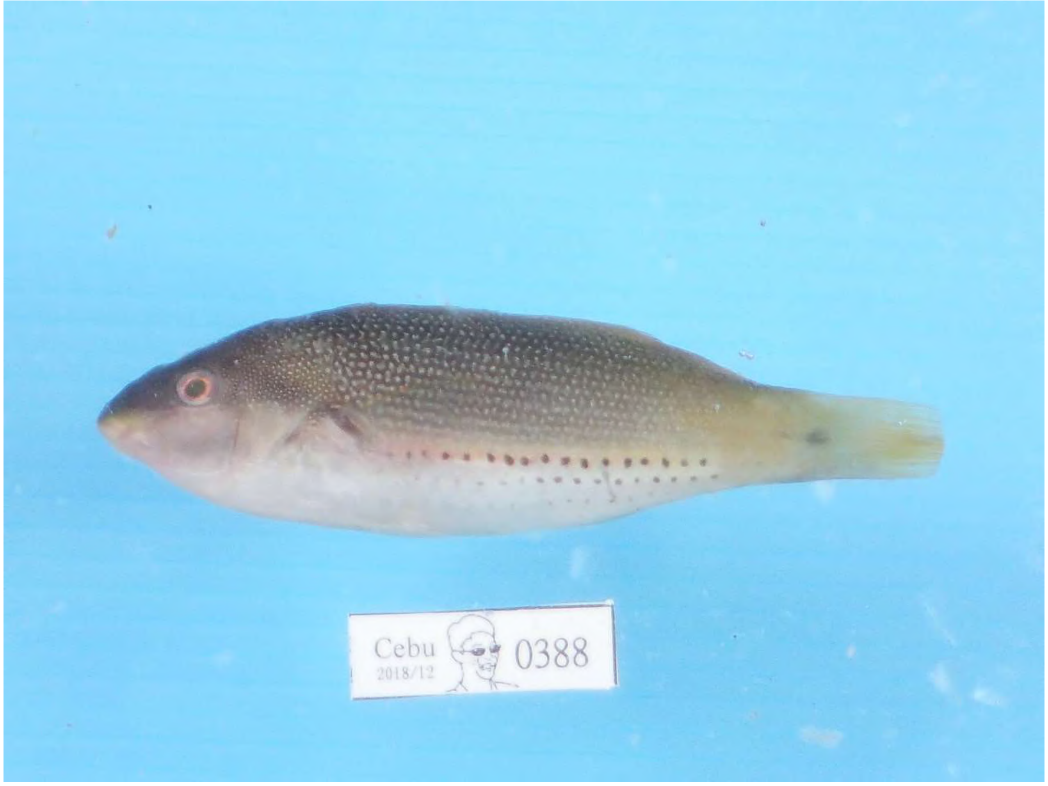

DOS 06761-3, *Stethojulis trilineata*, OR113946.

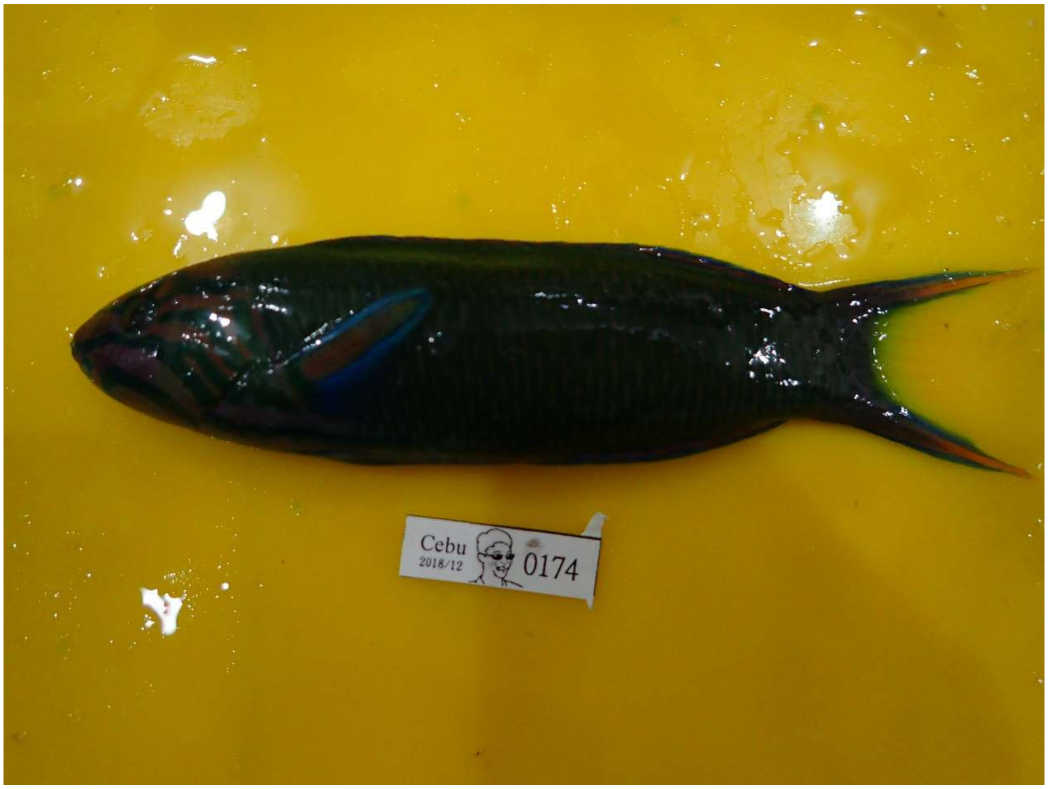

DOS 06762-1, *Thalassoma lunare*, OR113947. (specimen not preserved)

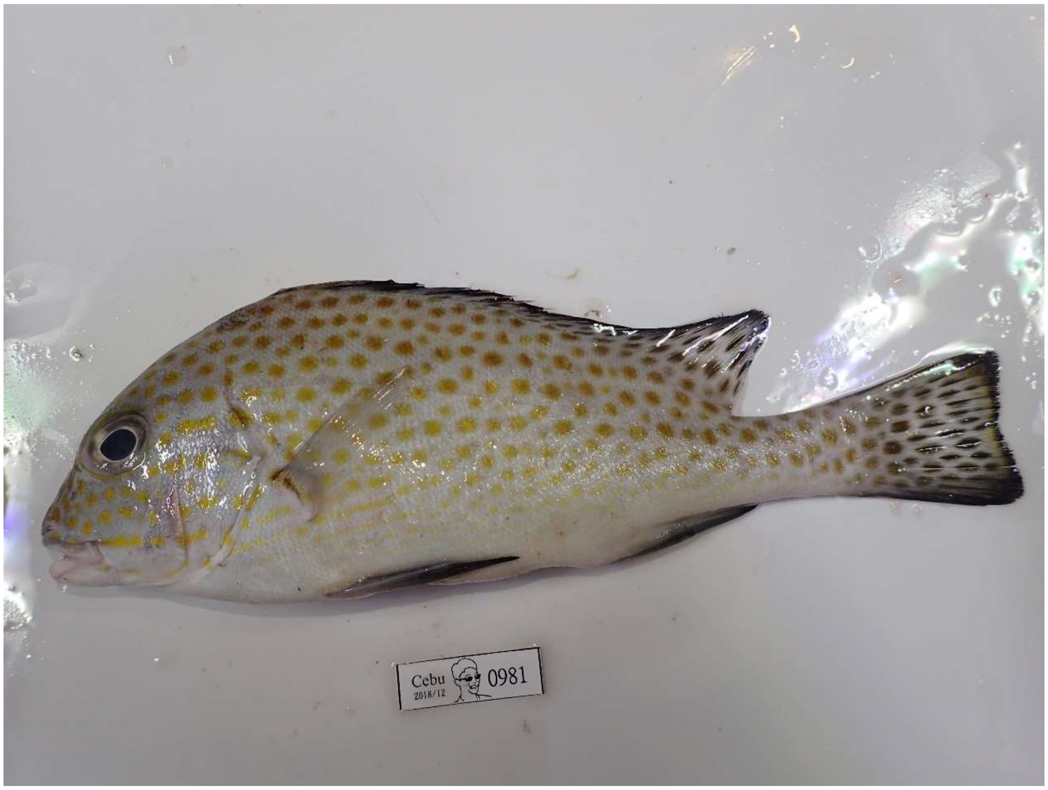

DOS 06714, *Diagramma pictum*, OR113902. (specimen not preserved)

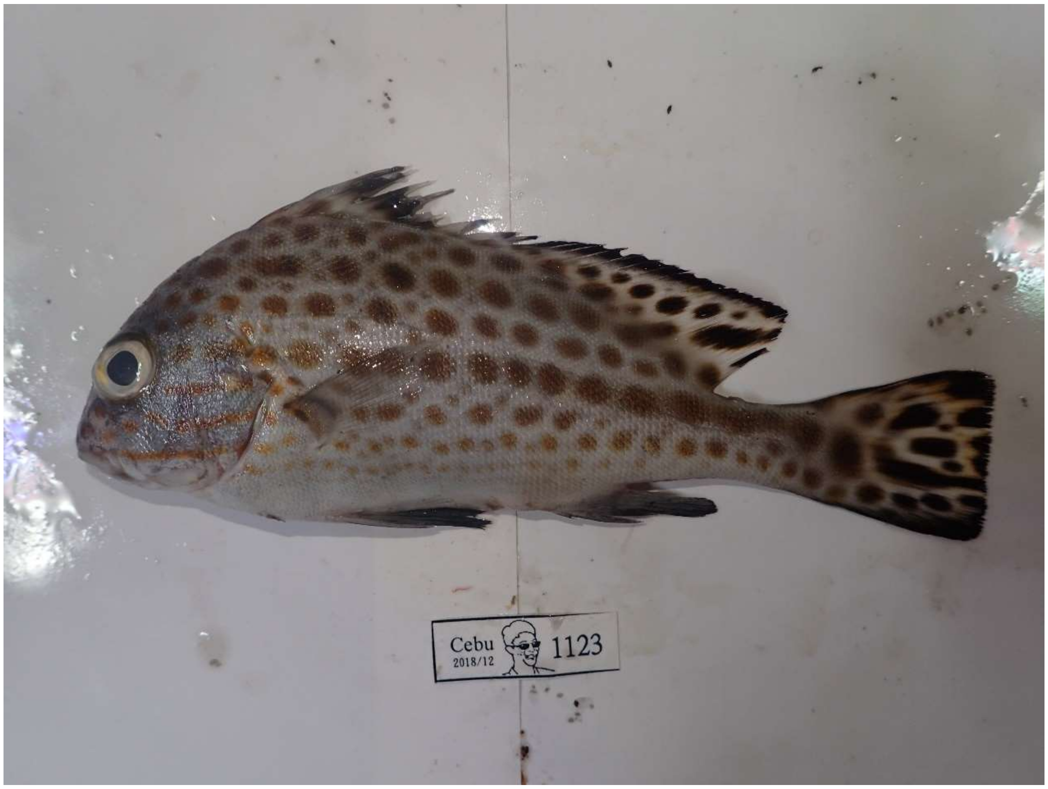

DOS 06718, *Diagramma pictum*, OR113906.

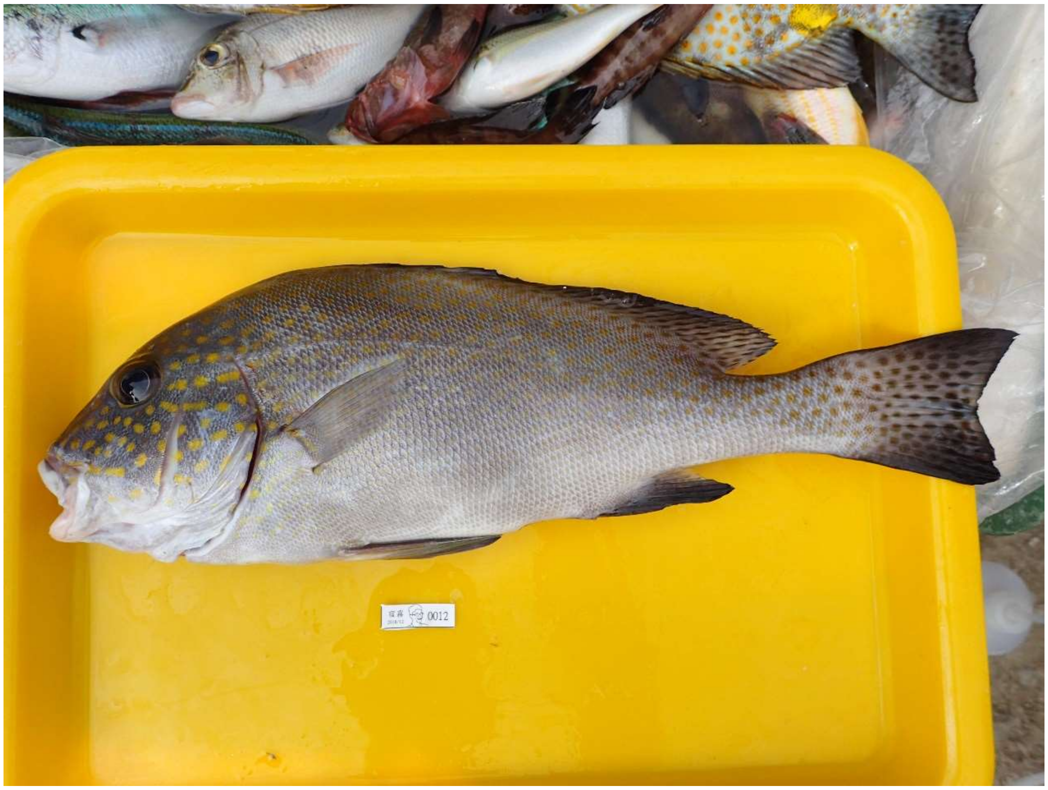

DOS 06720-1, *Diagramma pictum*, OR113908. (specimen not preserved)

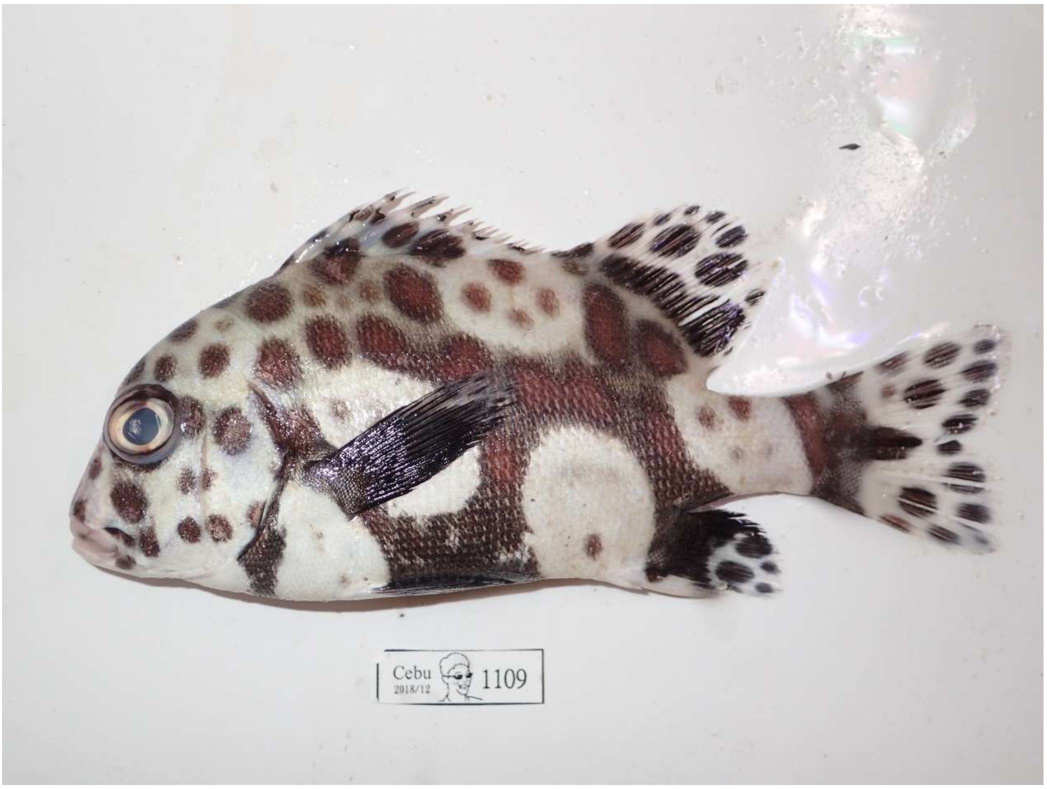

DOS 06713, *Plectorhinchus chaetodonoides*, OR113901.

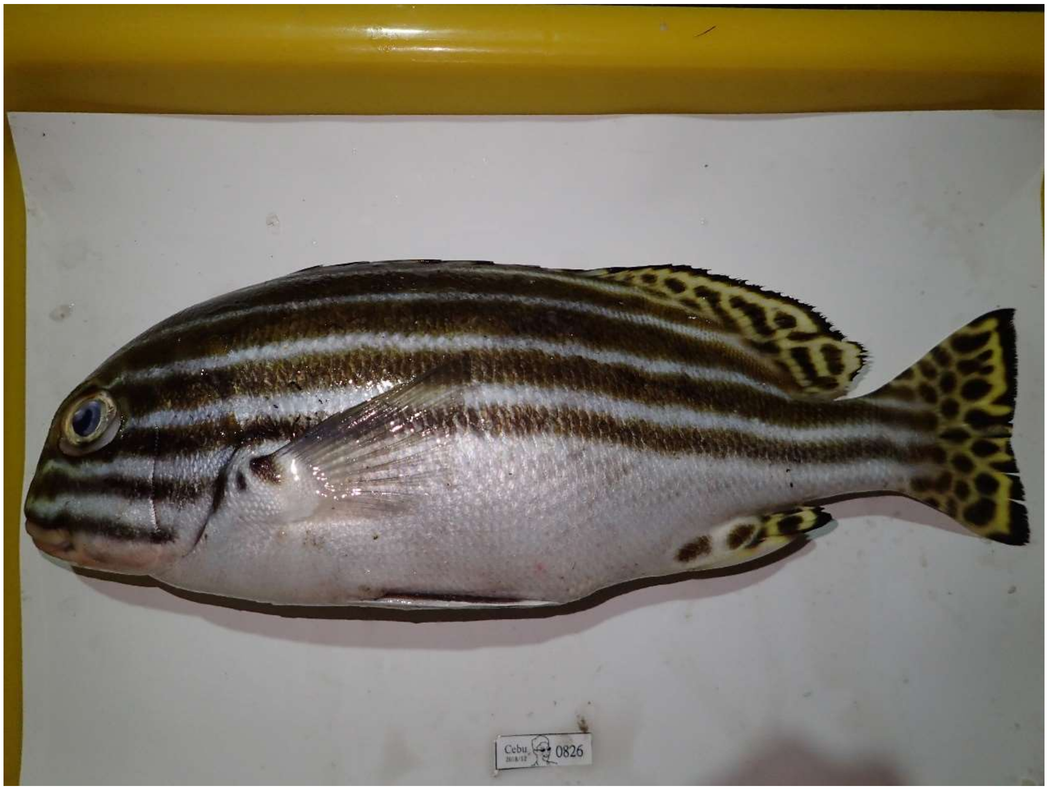

DOS 06715, *Plectorhinchus lessonii*, OR113903. (specimen not preserved)

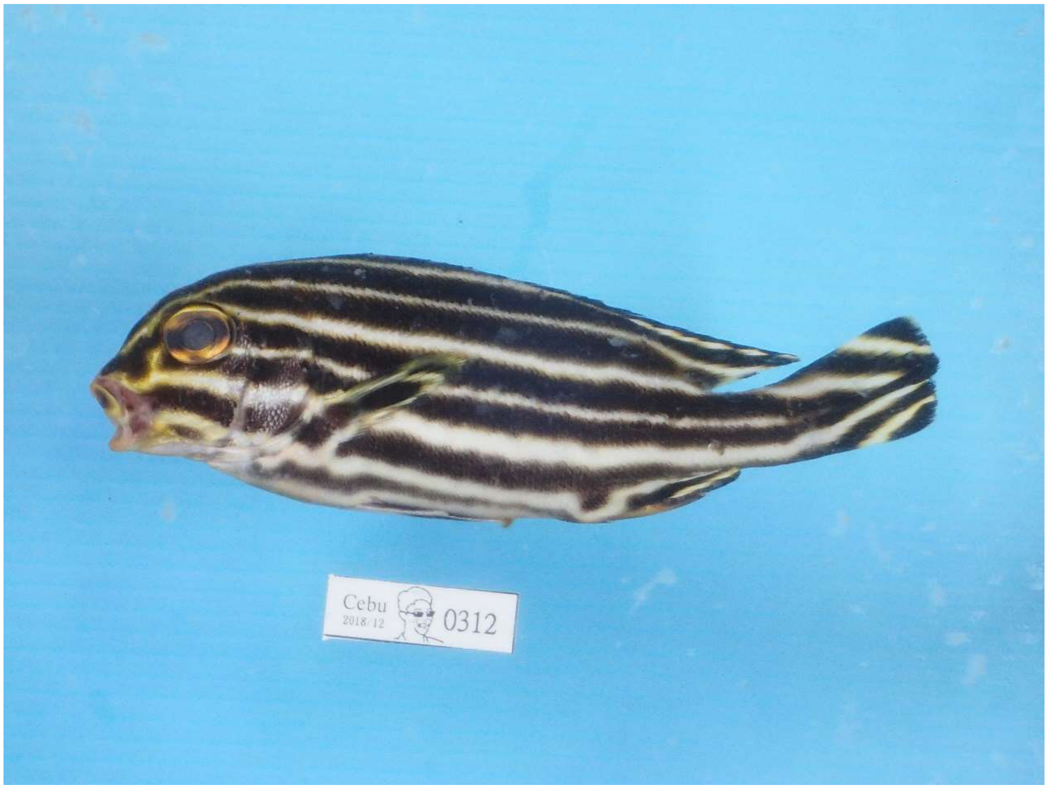

DOS 06716, *Plectorhinchus lessonii*, OR113904.

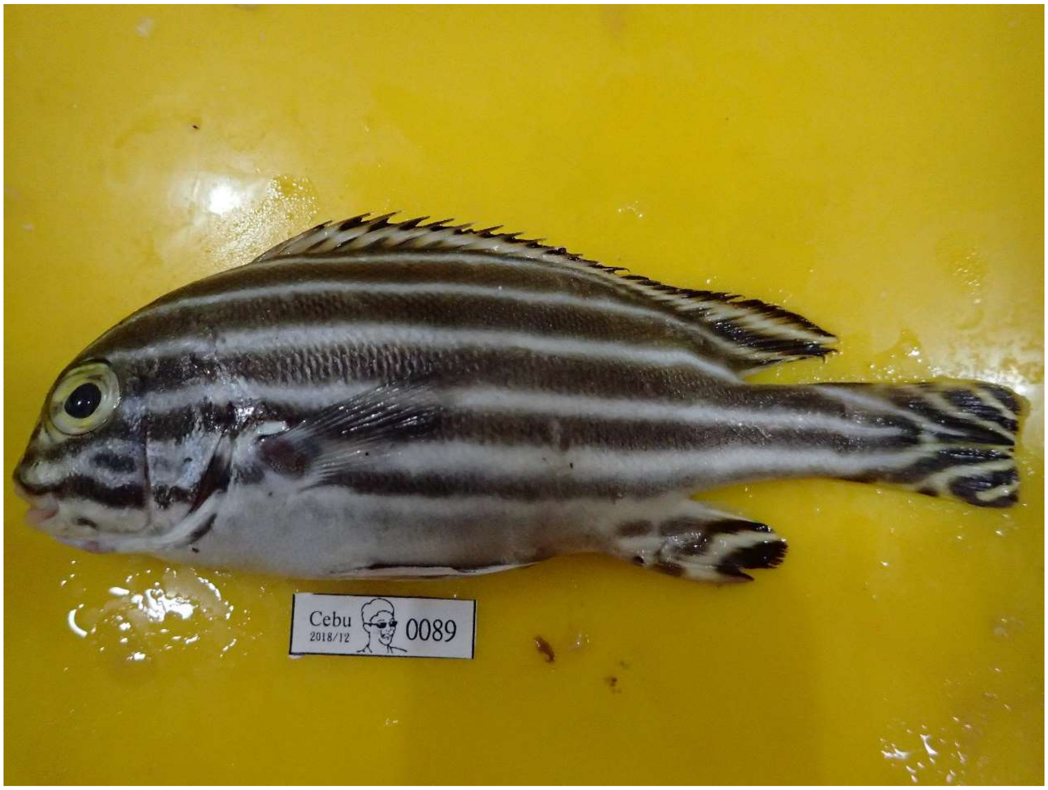

DOS 06719-1, *Plectorhinchus lessonii*, OR113907. (specimen not preserved)

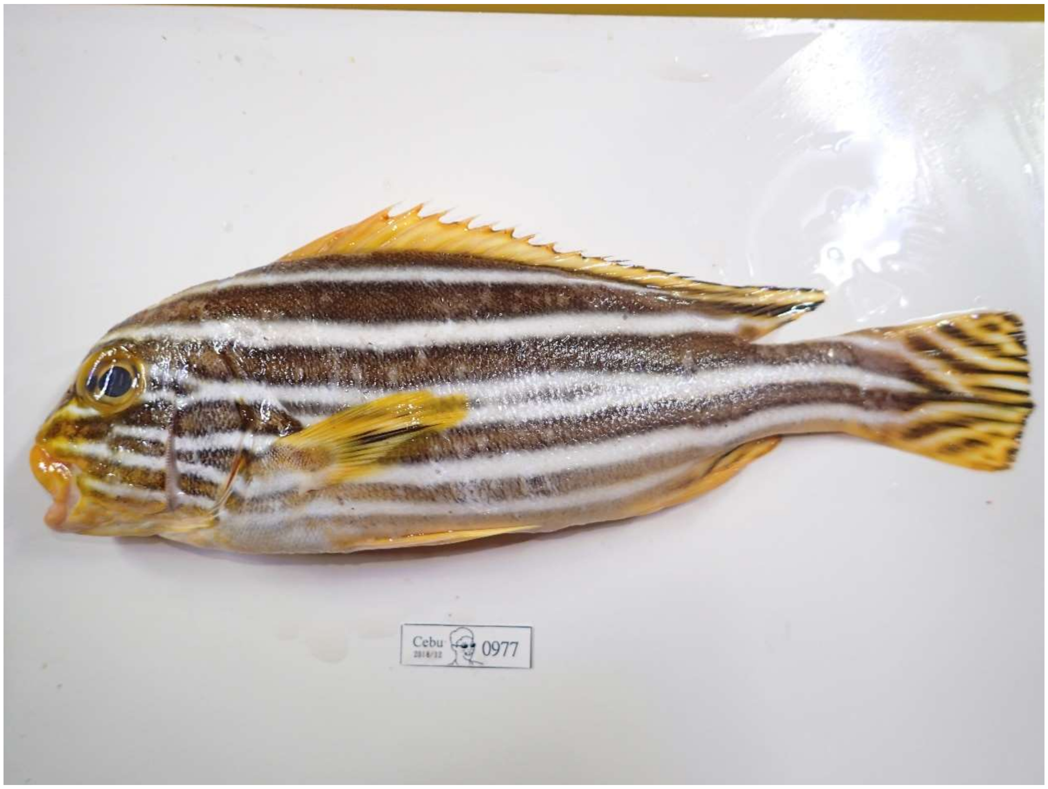

DOS 06717, *Plectorhinchus polytaenia*, OR113905. (specimen not preserved)

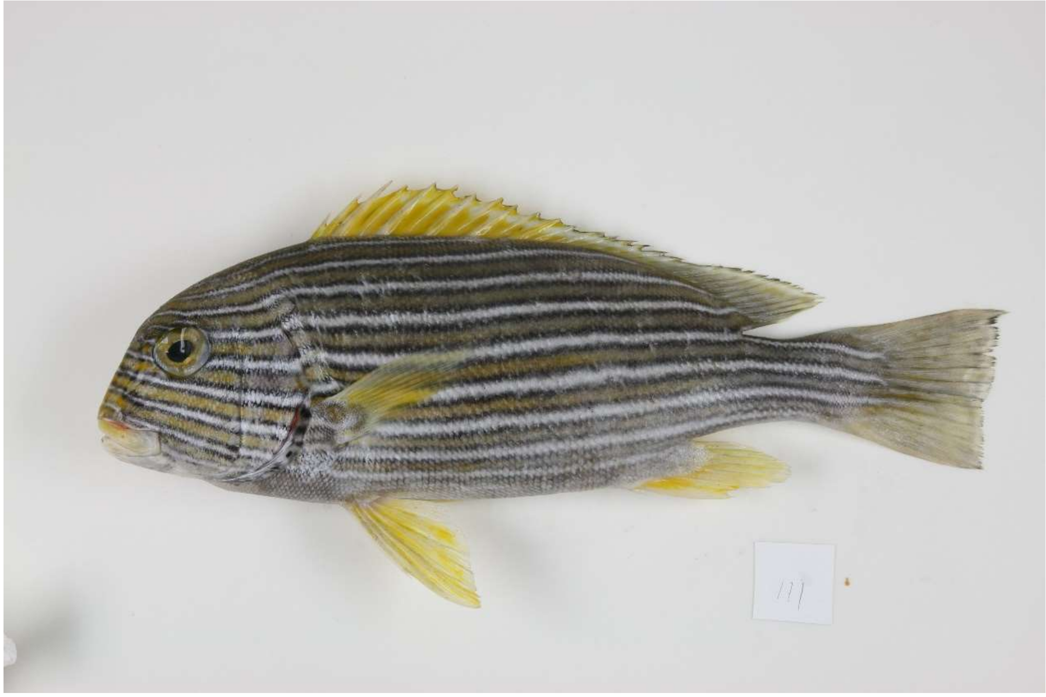

DOS 08654, *Plectorhinchus polytaenia*, OR114230.

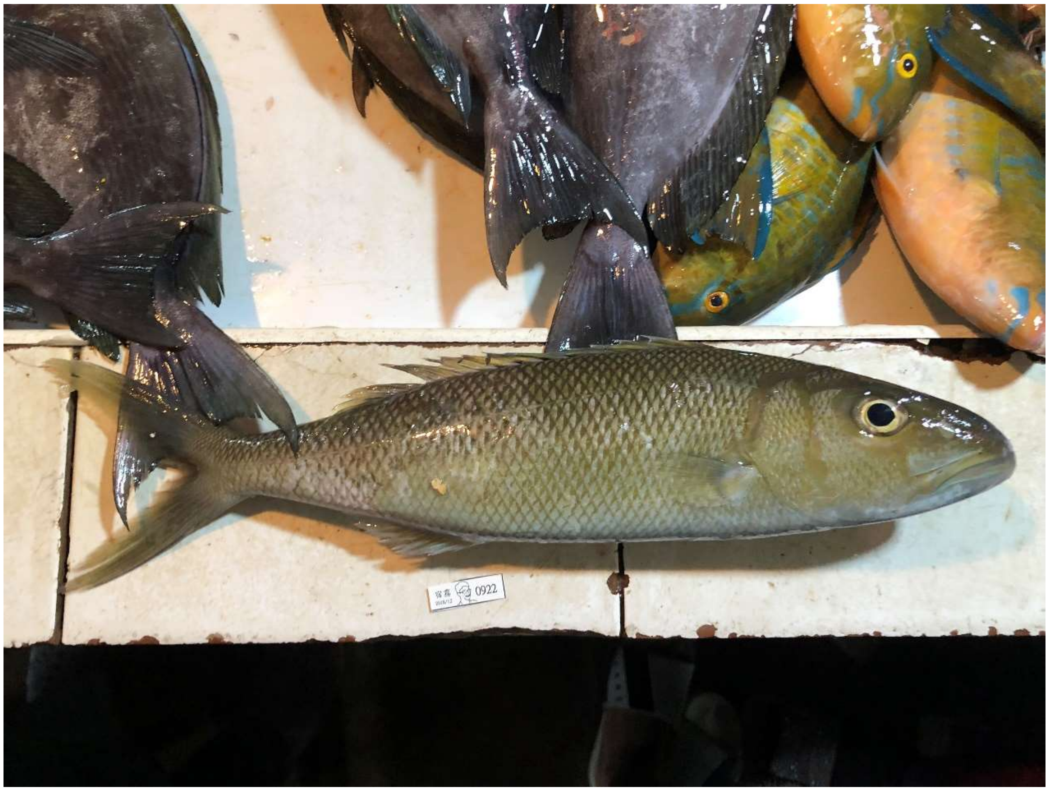

DOS 07009-1, *Aprion virescens*, OR114175. (specimen not preserved)

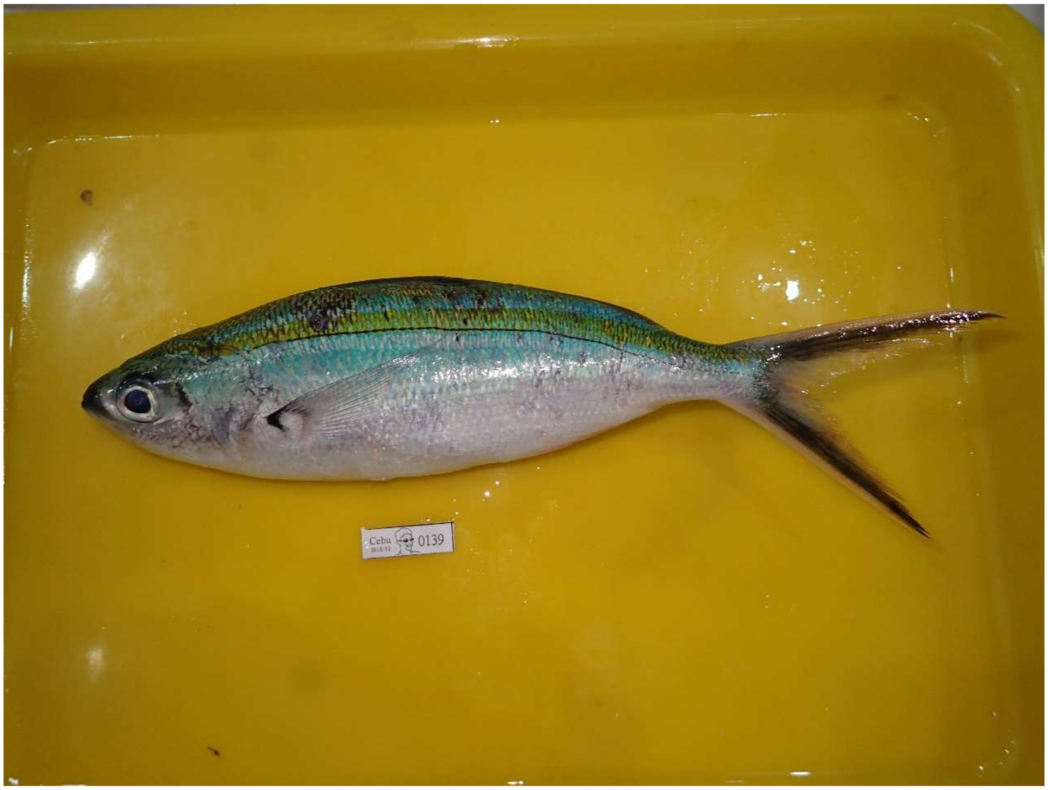

DOS 06624, *Caesio caerulaurea*, OR113815. (specimen not preserved)

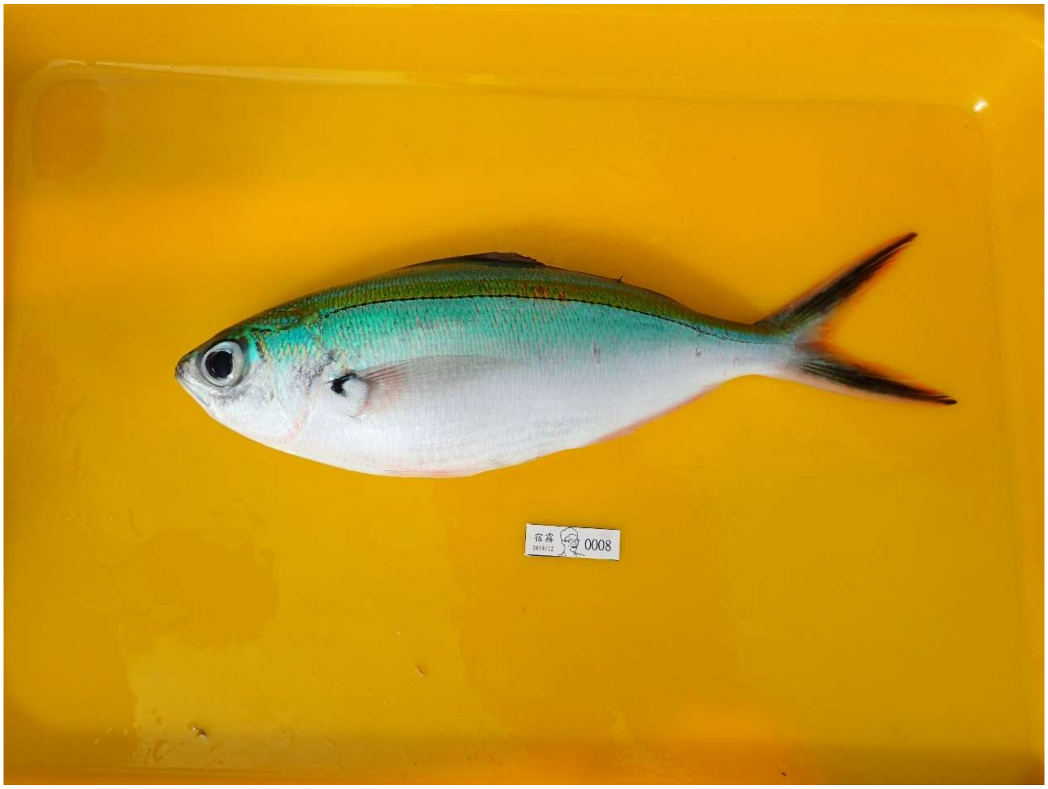

DOS 06630-1, *Caesio caerulaurea*, OR113821. (specimen not preserved)

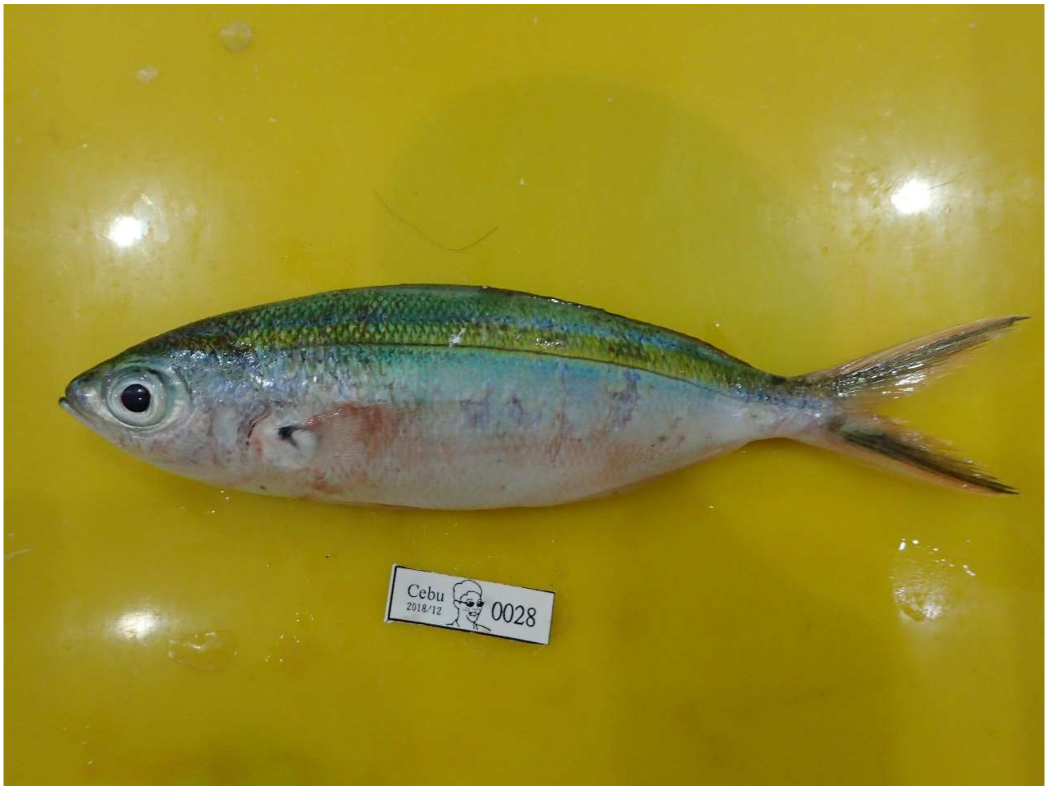

DOS 06630-3, *Caesio caerulaurea*, OR113822. (specimen not preserved)

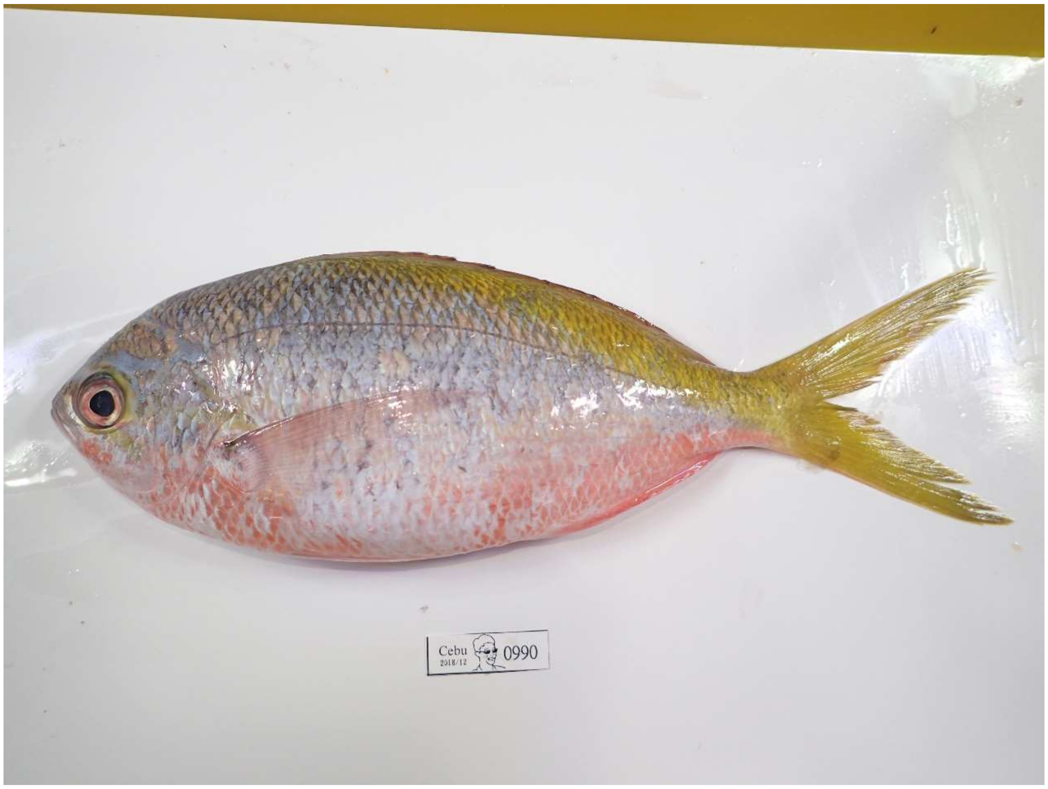

DOS 06625, *Caesio cuning*, OR113816. (specimen not preserved)

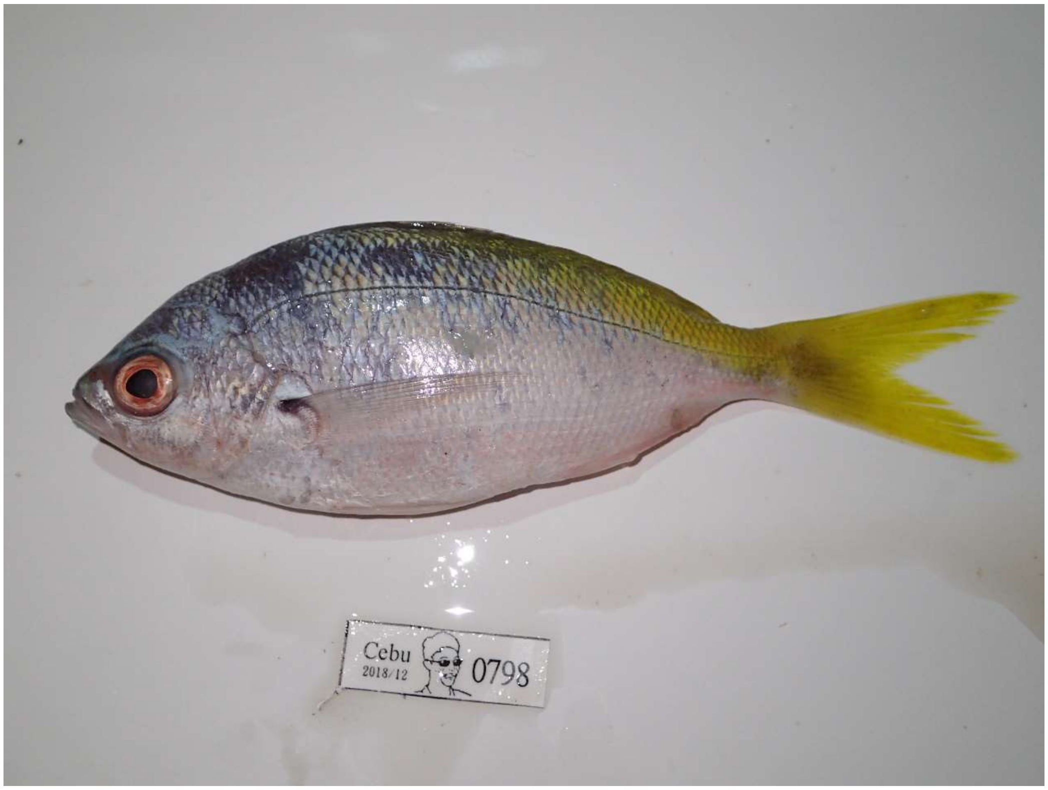

DOS 06626-1, *Caesio cuning*, OR113817.

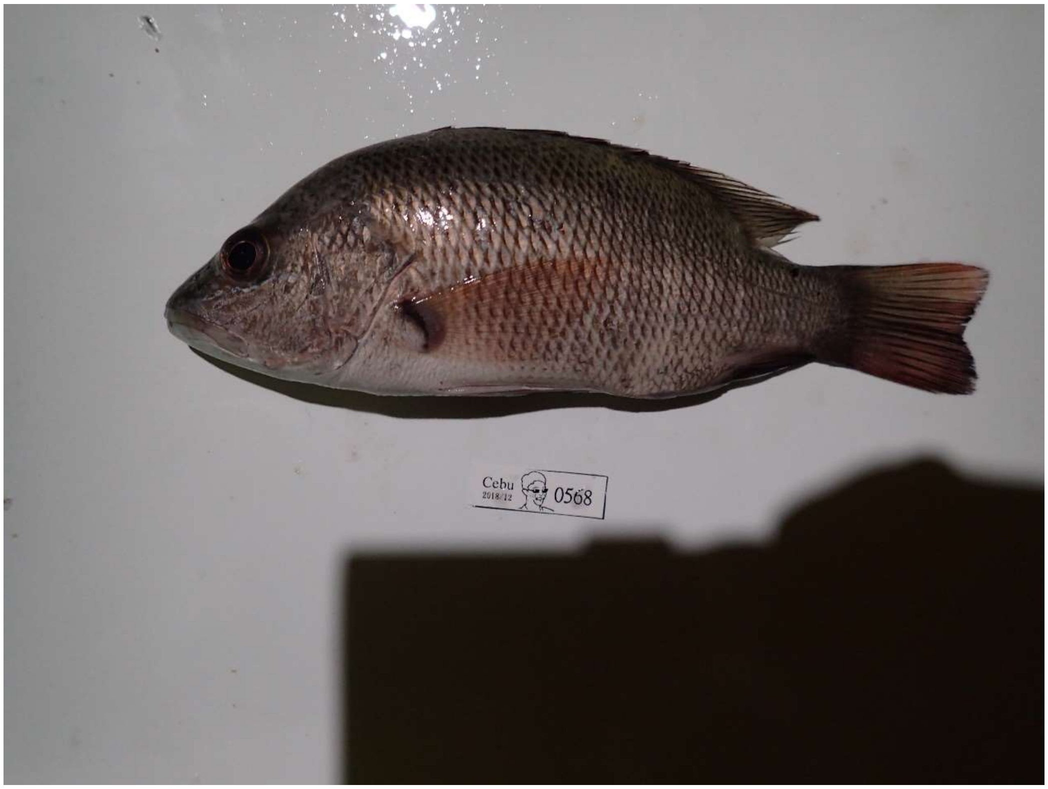

DOS 06780-1, *Lutjanus argentimaculatus*, OR113961.

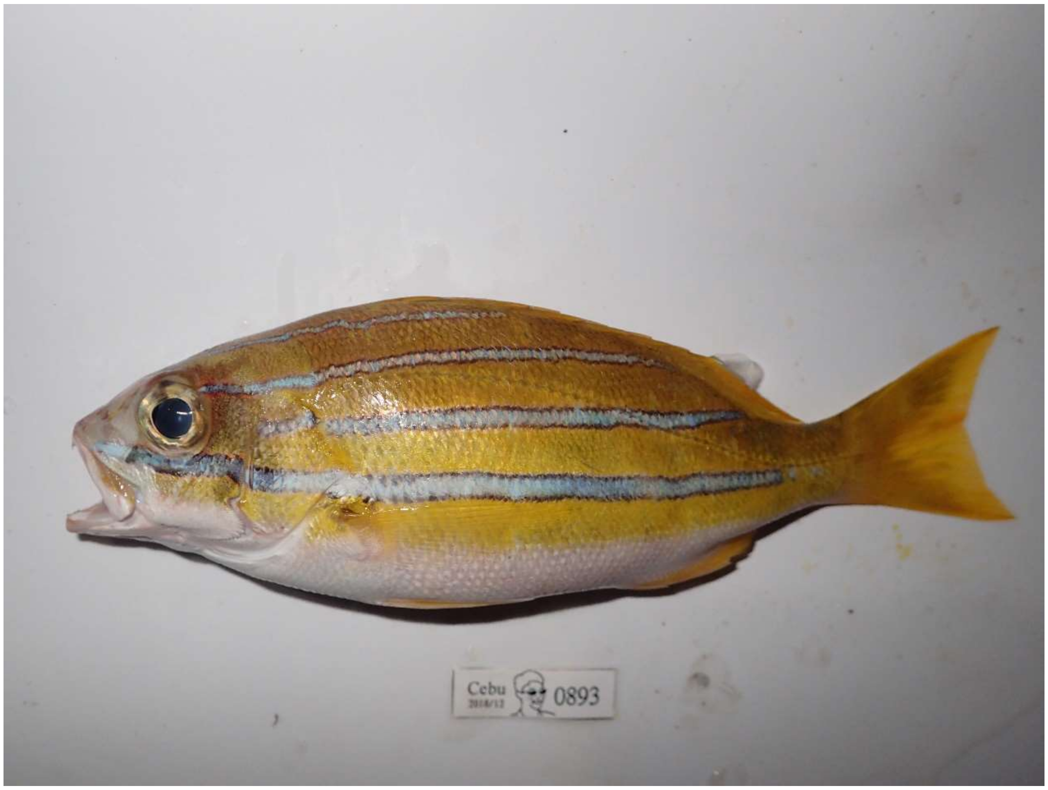

DOS 06790-9, *Lutjanus bengalensis*, OR113971.

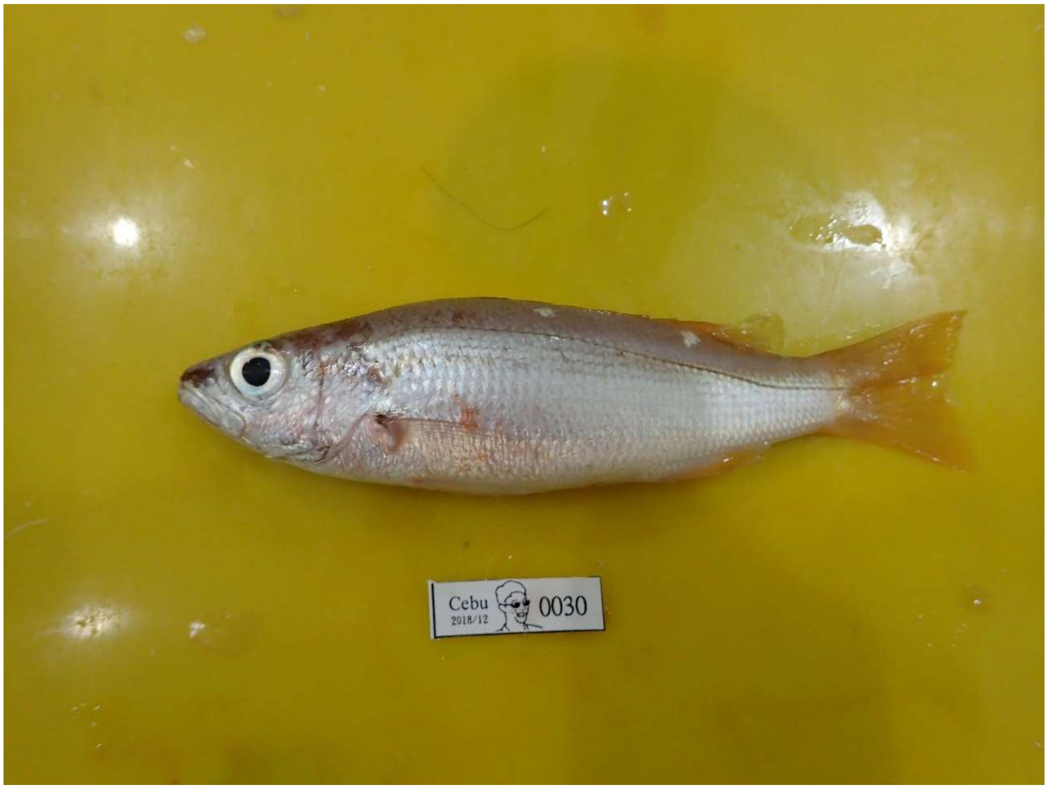

DOS 06791-1, *Lutjanus biguttatus*, OR113972. (specimen not preserved)

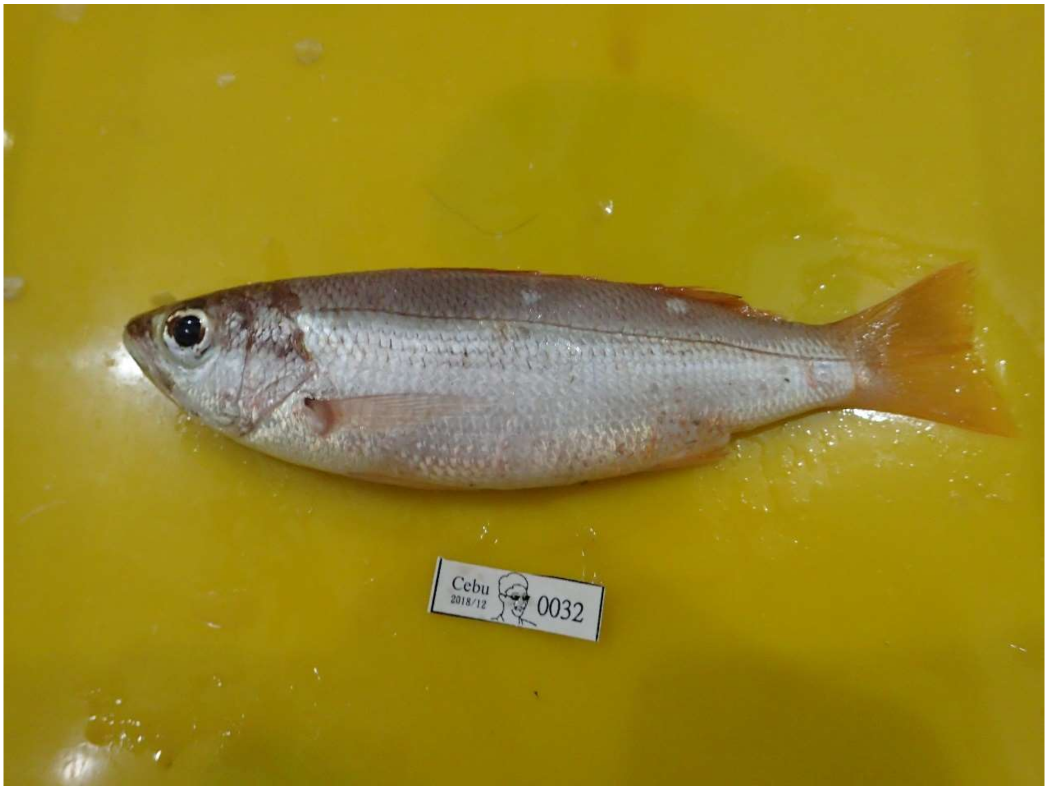

DOS 06791-3, *Lutjanus biguttatus*, OR113973.

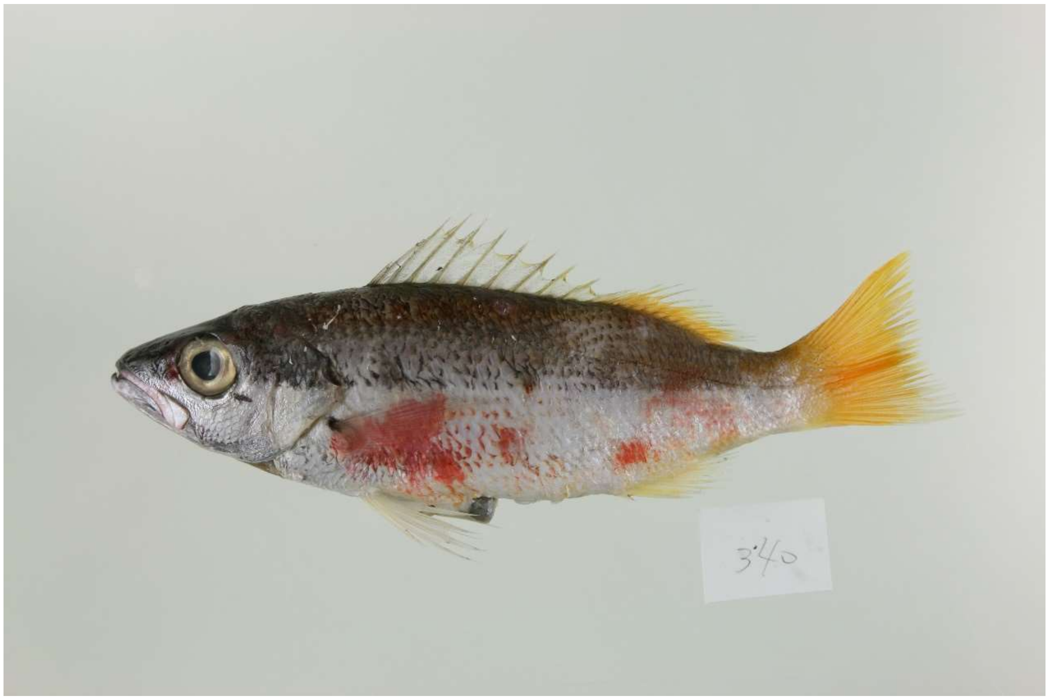

DOS 08656, *Lutjanus biguttatus*, OR114232.

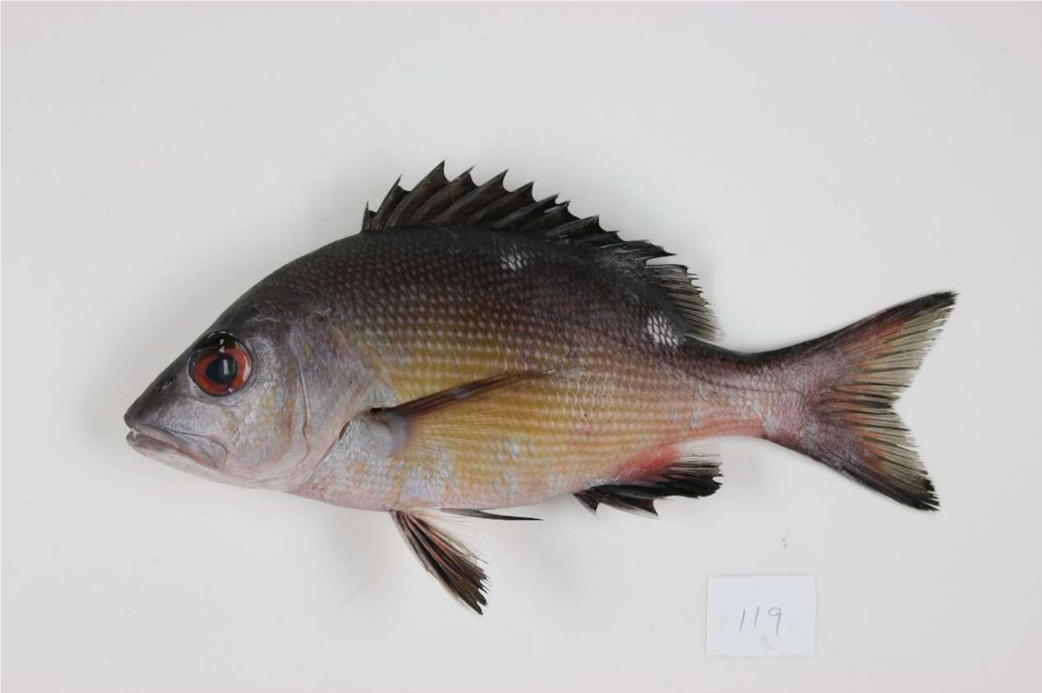

DOS 08657, *Lutjanus bohar*, OR114233.

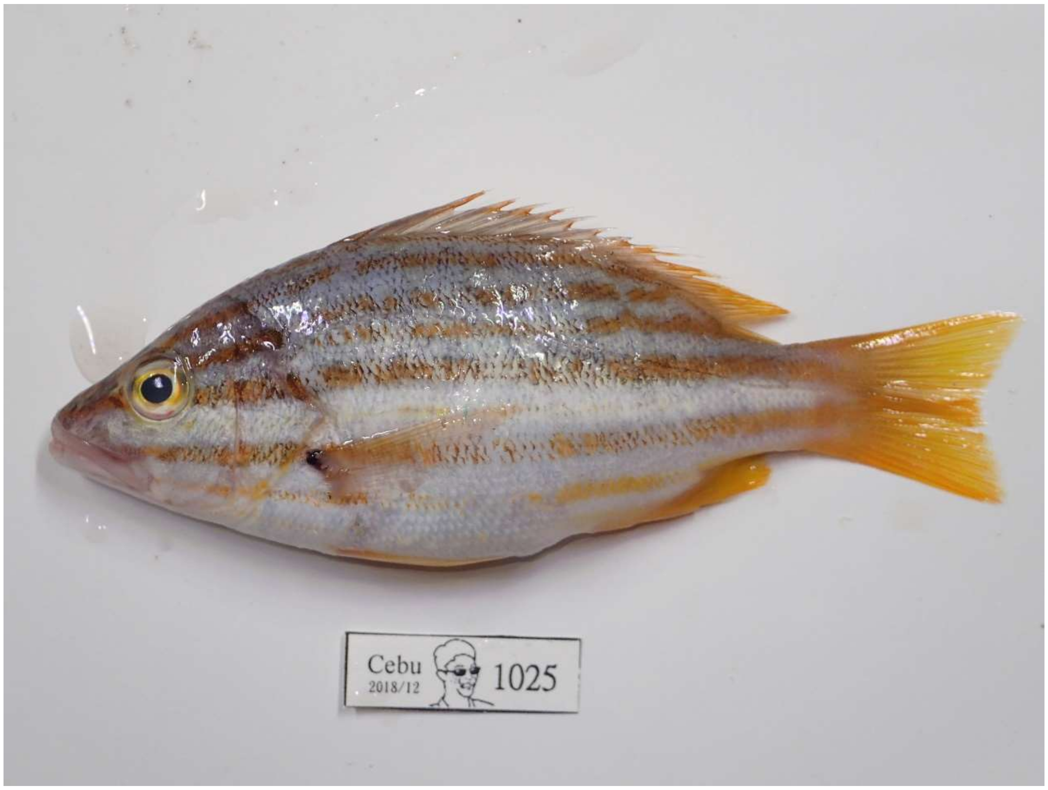

DOS 06781, *Lutjanus carponotatus*, OR113962.

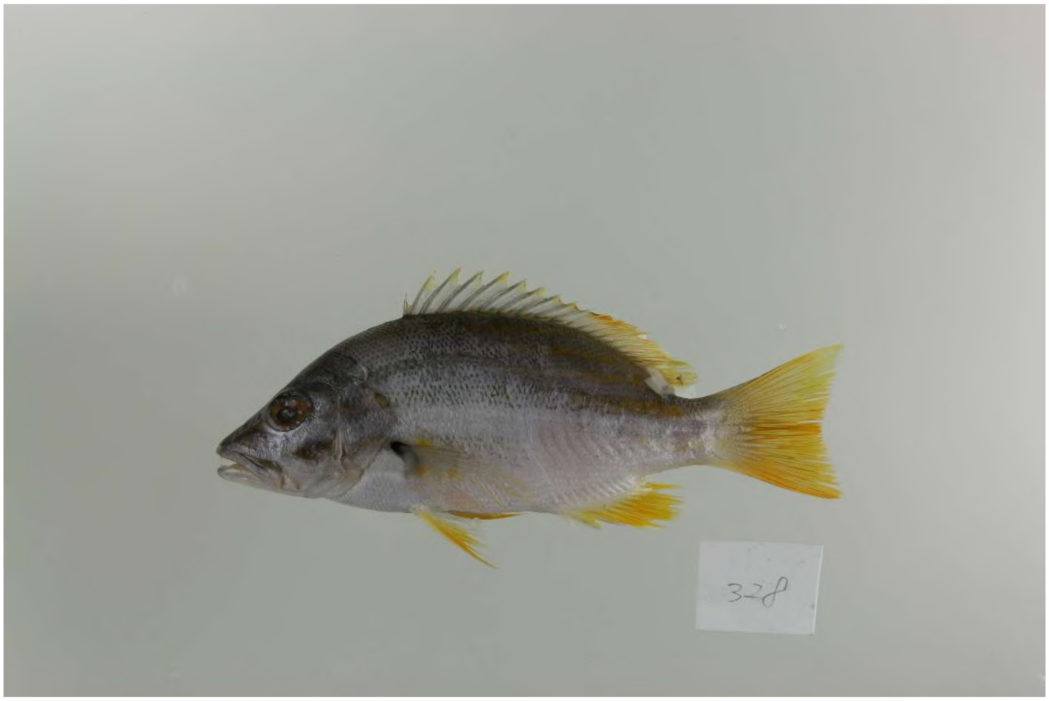

DOS 08658, *Lutjanus carponotatus*, OR114234.

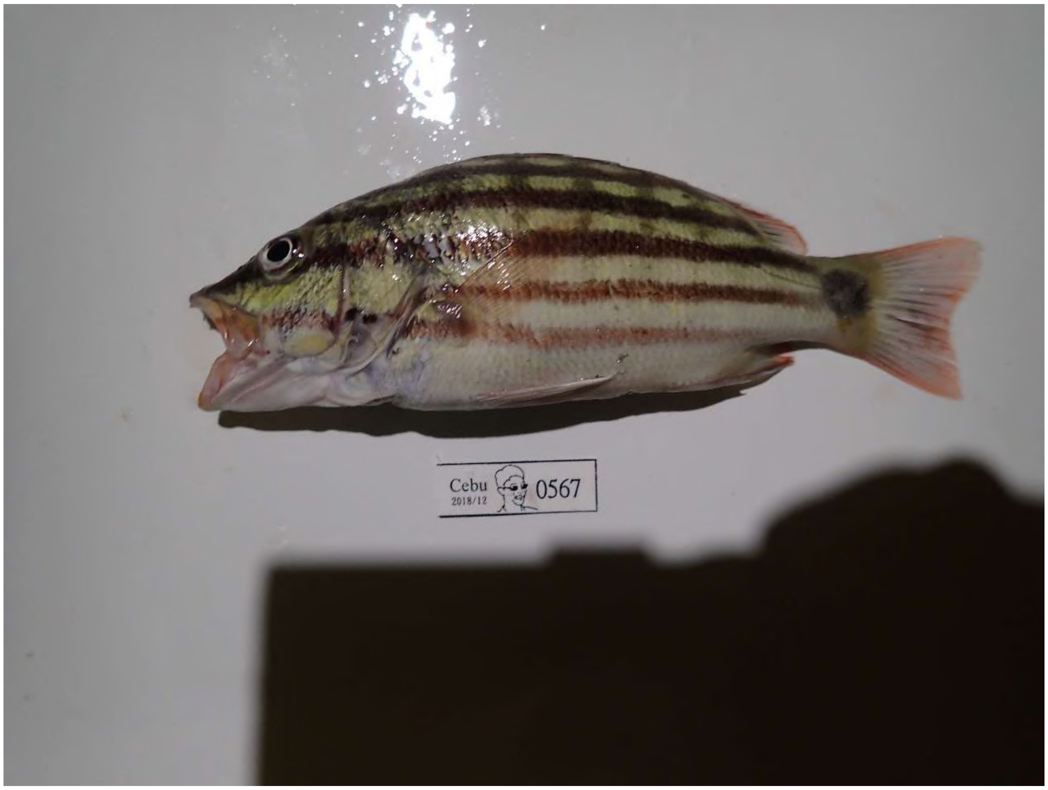

DOS 06782-2, *Lutjanus decussatus*, OR113963.

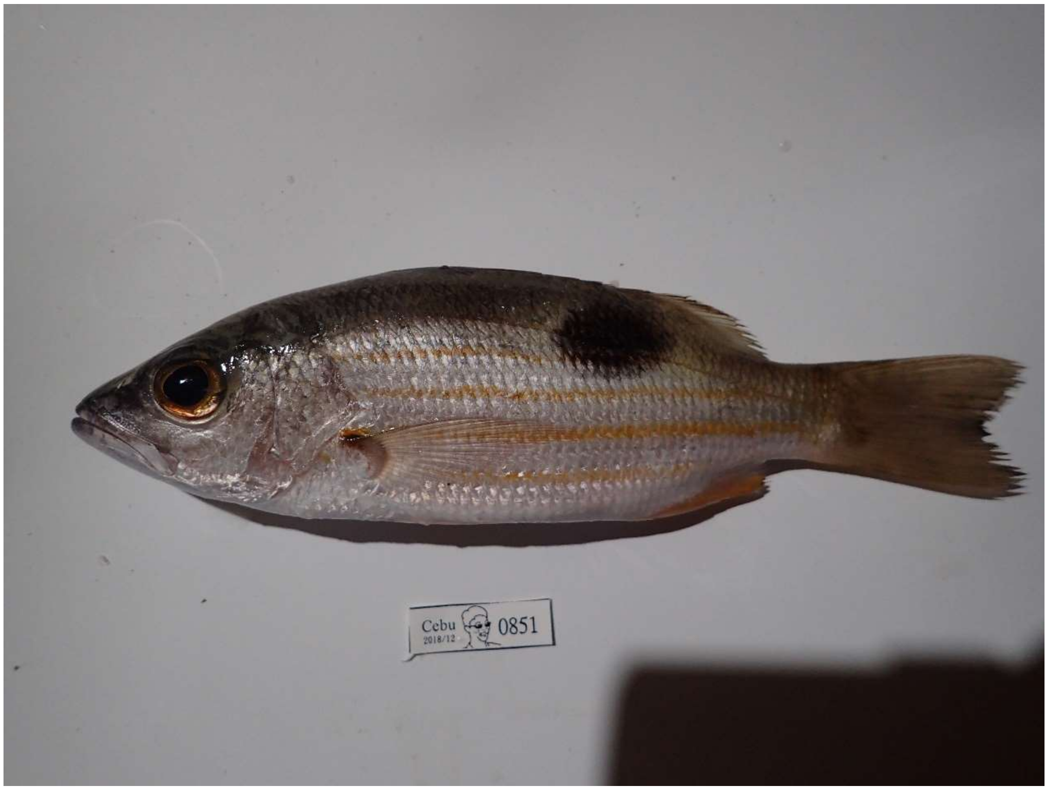

DOS 06783, *Lutjanus ehrenbergii*, OR113964. (specimen not preserved)

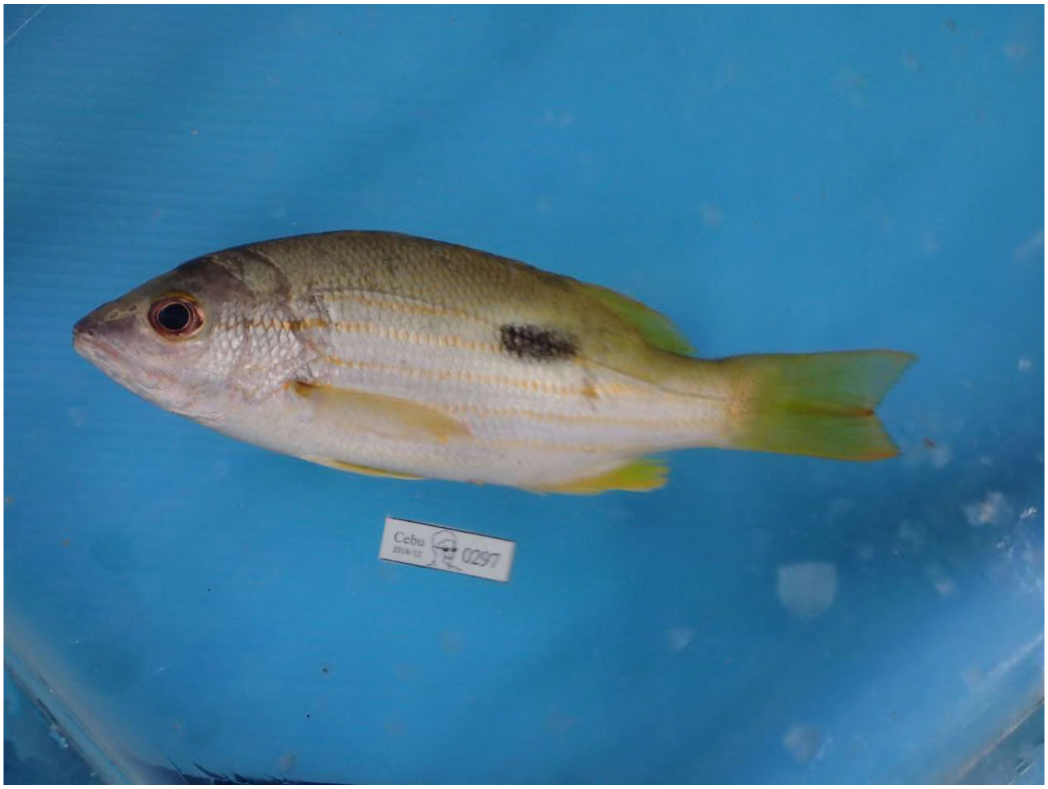

DOS 06788-1, *Lutjanus fulviflamma*, OR113969. (specimen not preserved)

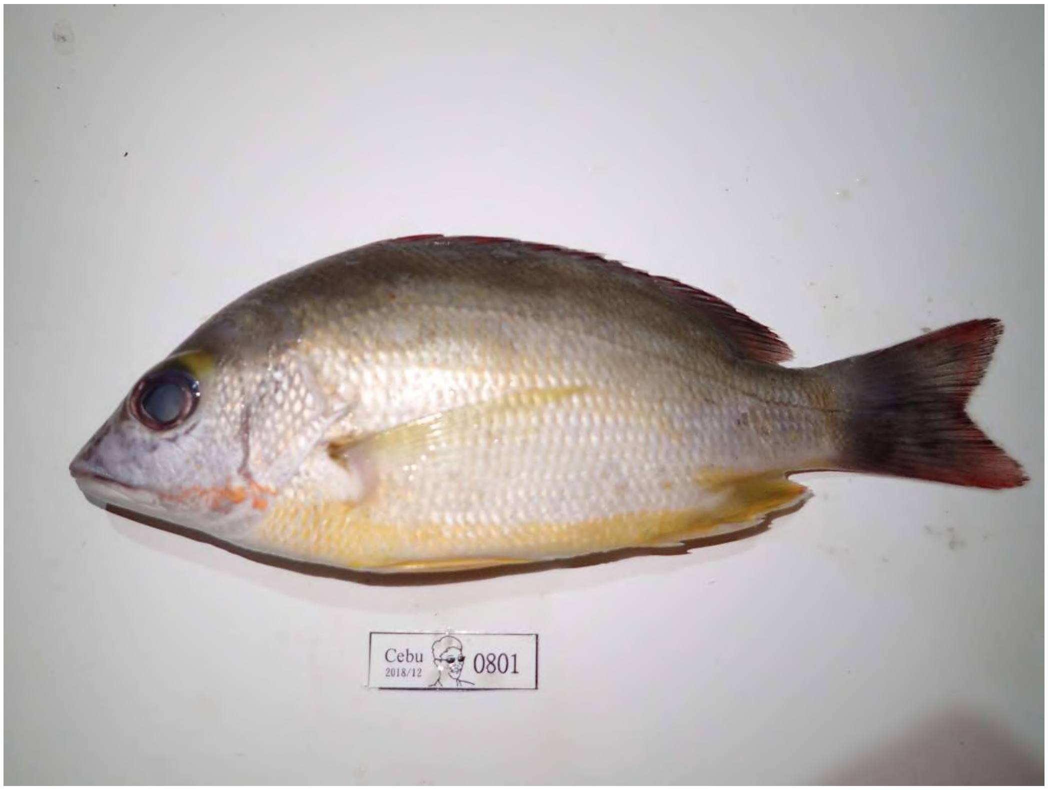

DOS 06784-1, *Lutjanus fulvus*, OR113965.

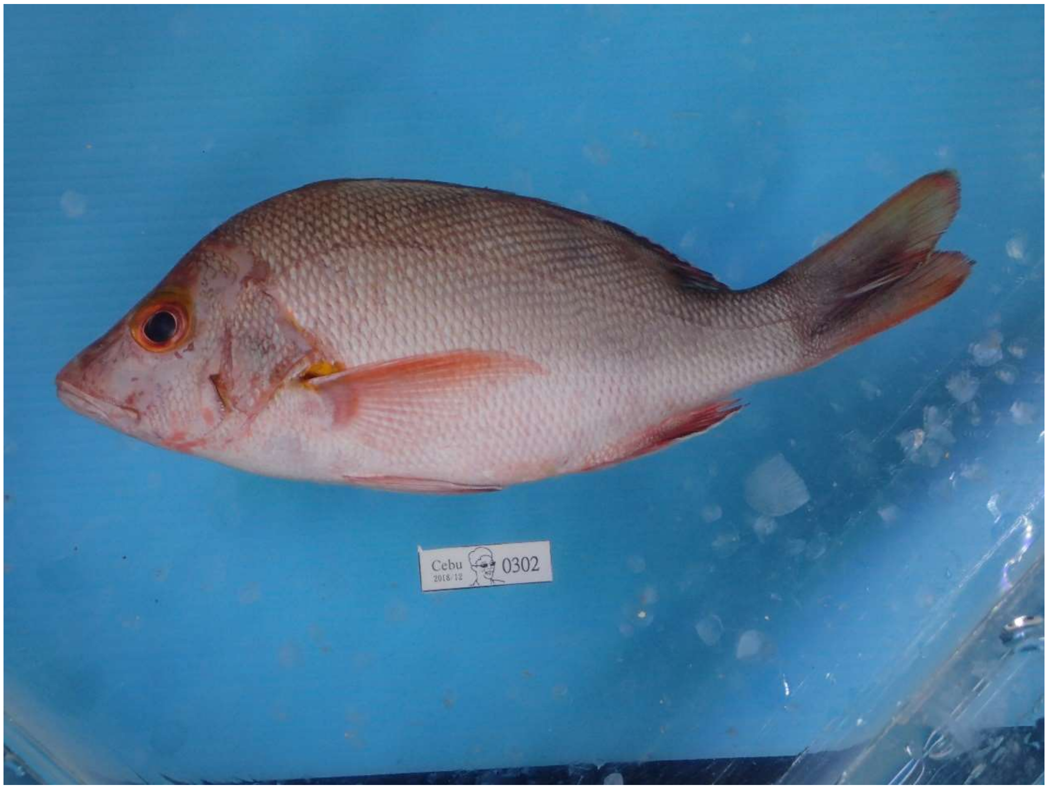

DOS 06785, *Lutjanus gibbus*, OR113966.

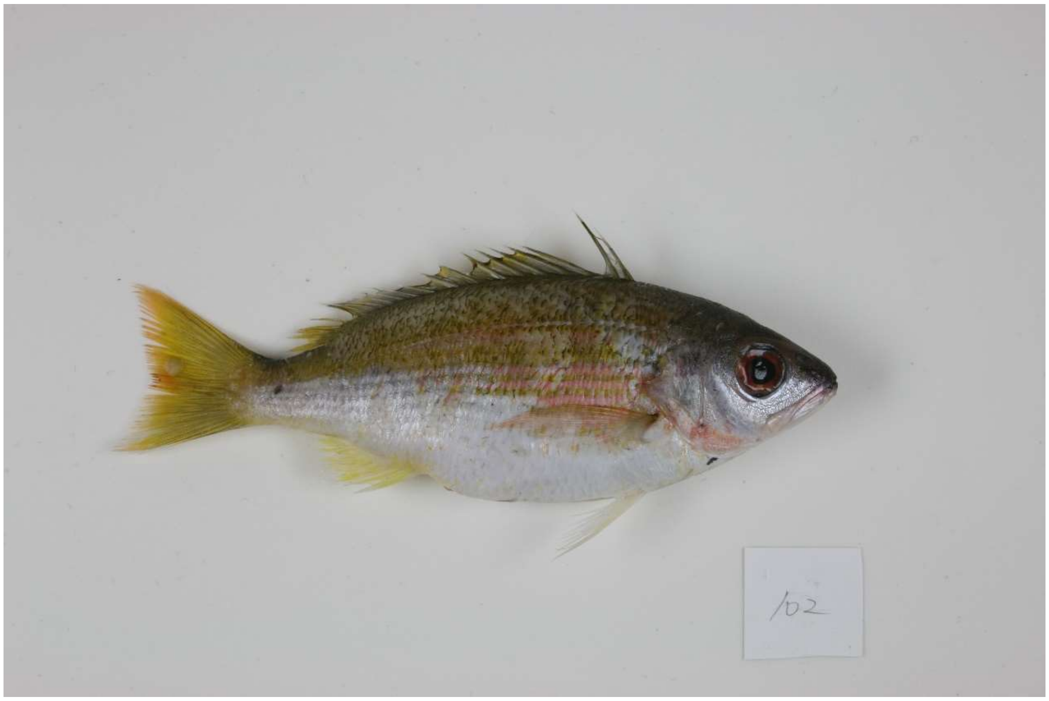

DOS 08659, *Lutjanus lutjanus*, OR114235.

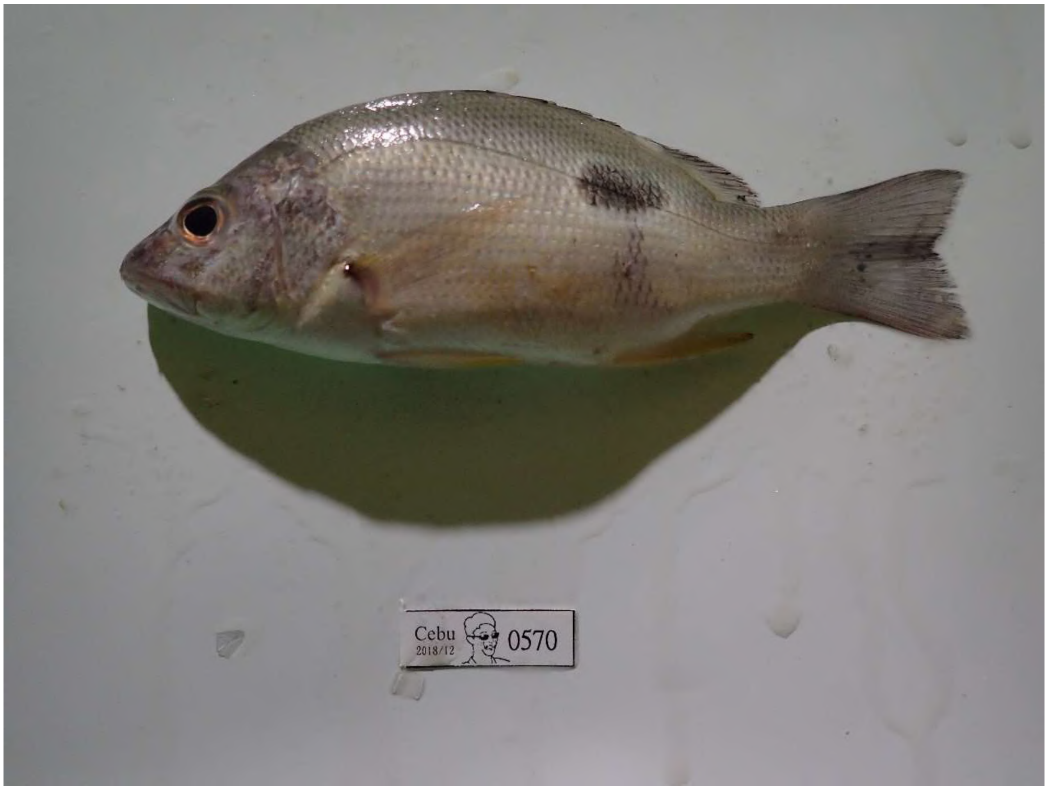

DOS 06786, *Lutjanus russellii*, OR113967.

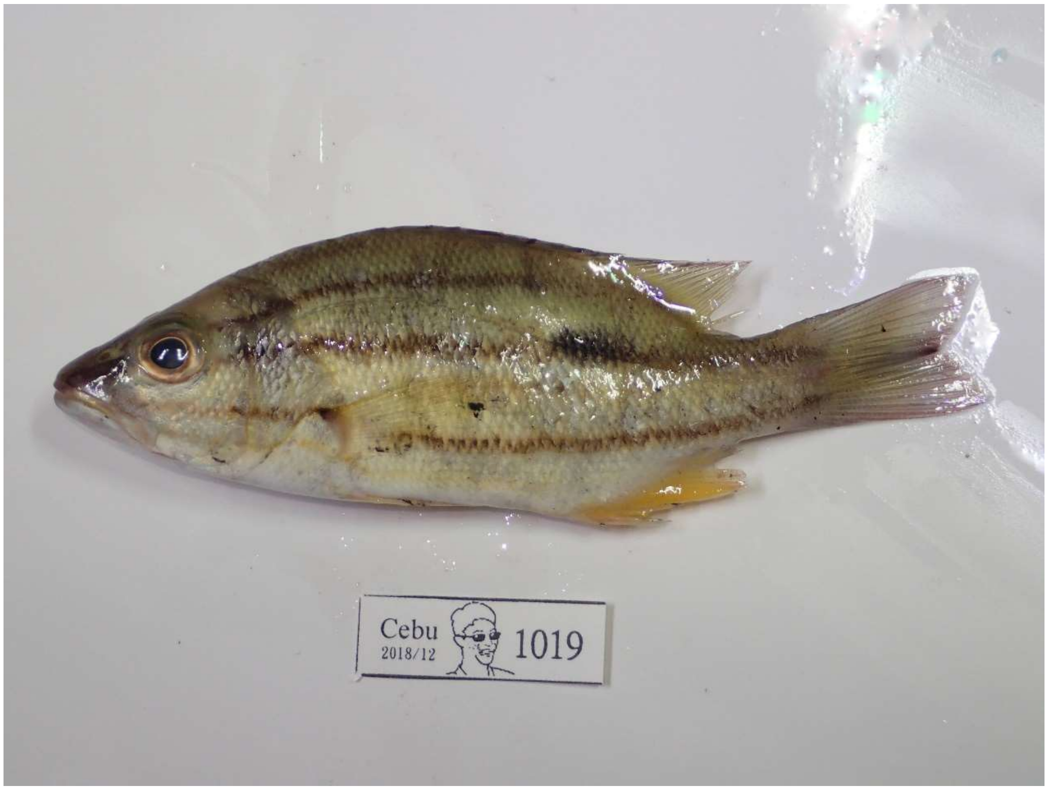

DOS 06789, *Lutjanus russellii*, OR113970.

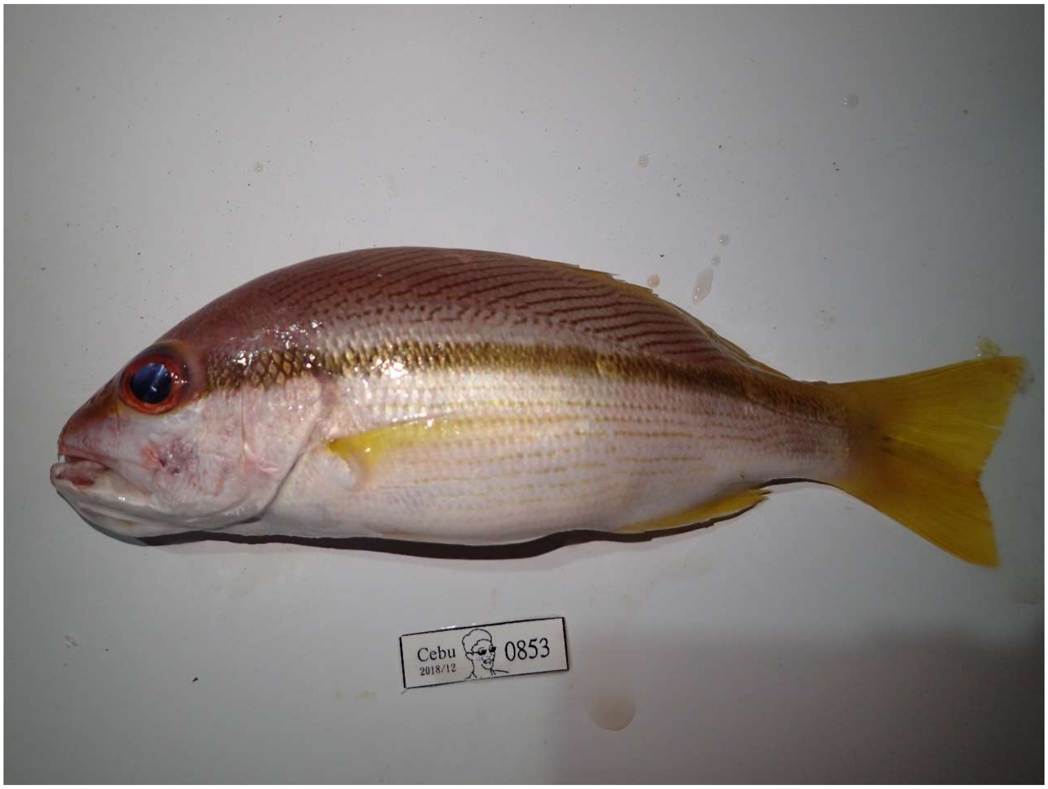

DOS 06787, *Lutjanus vitta*, OR113968.

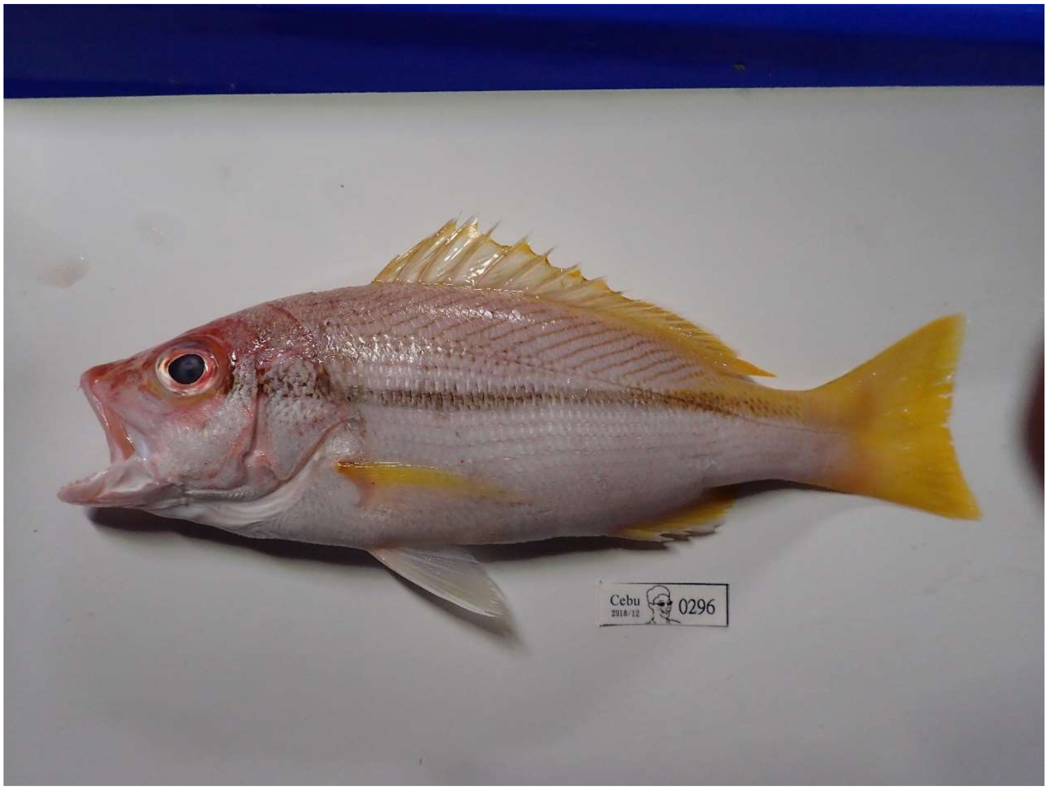

DOS 06792, *Lutjanus vitta*, OR113974.

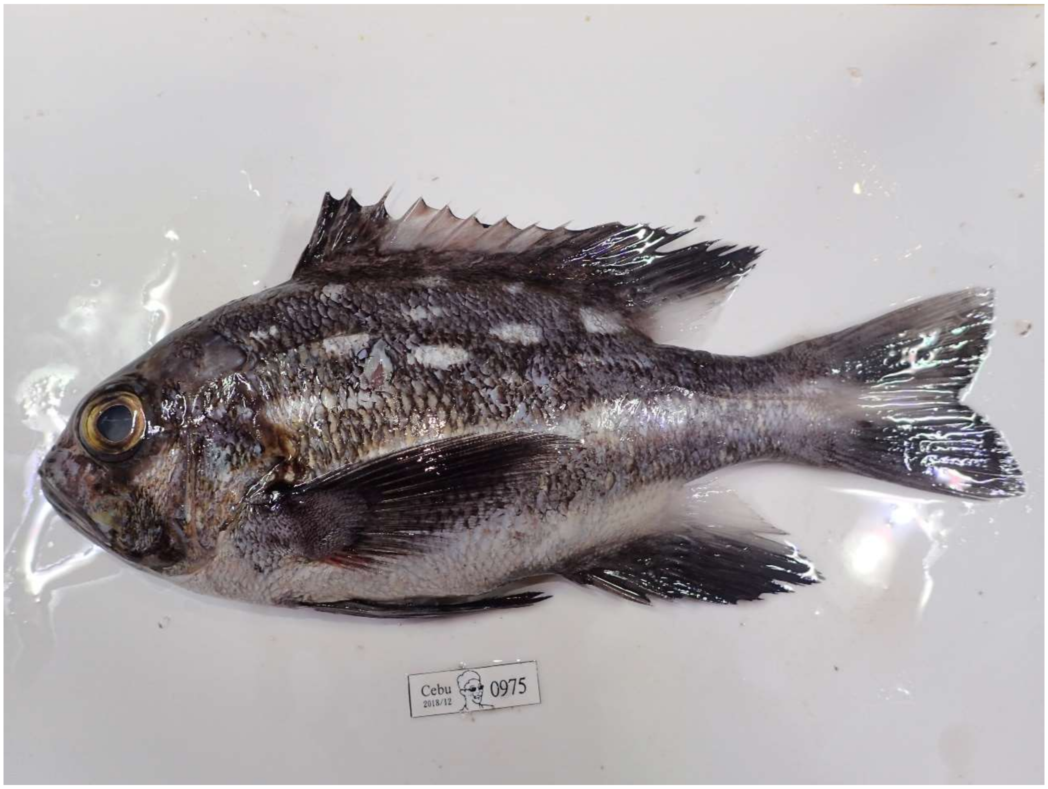

DOS 06793, *Macolor macularis*, OR113975. (specimen not preserved)

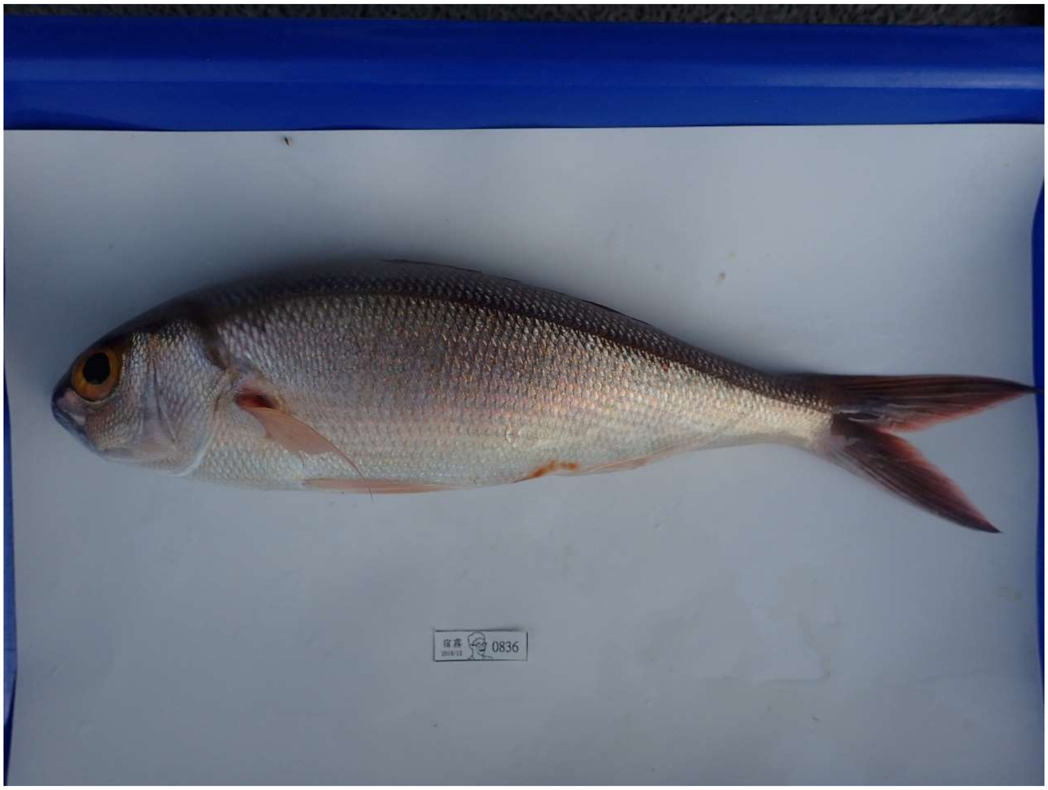

DOS 06779, *Pristipomoides sieboldii*, OR113960. (specimen not preserved)

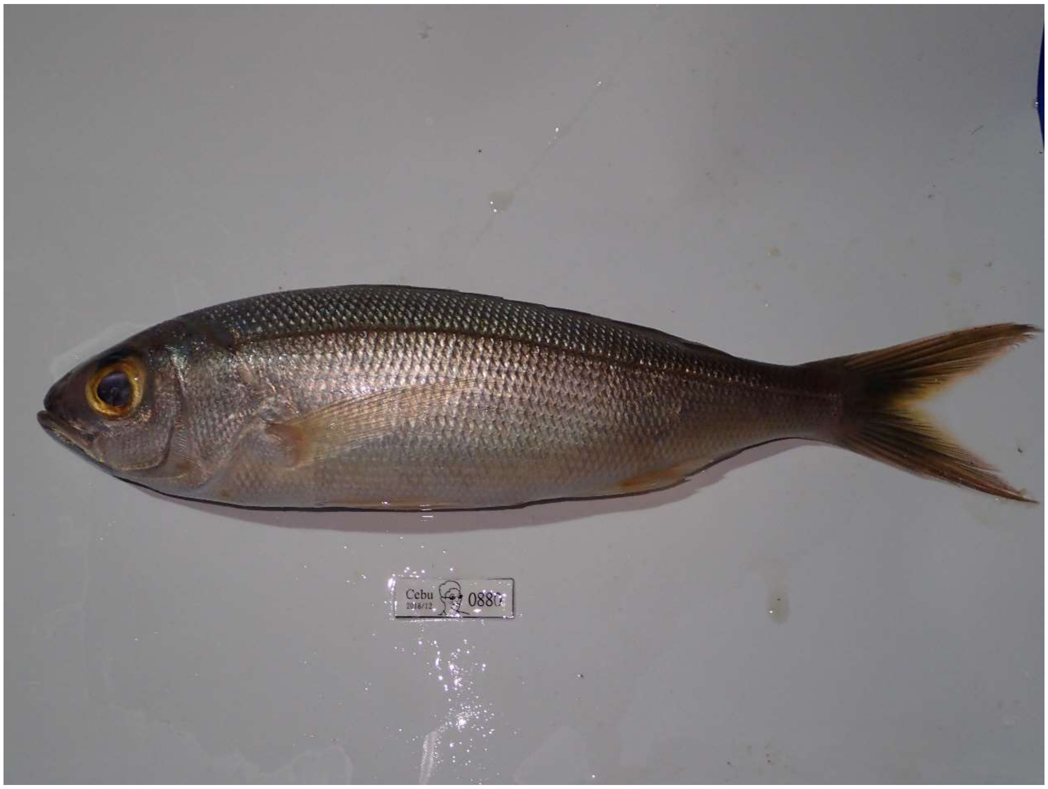

DOS 06794, *Pristipomoides sieboldii*, OR113976.

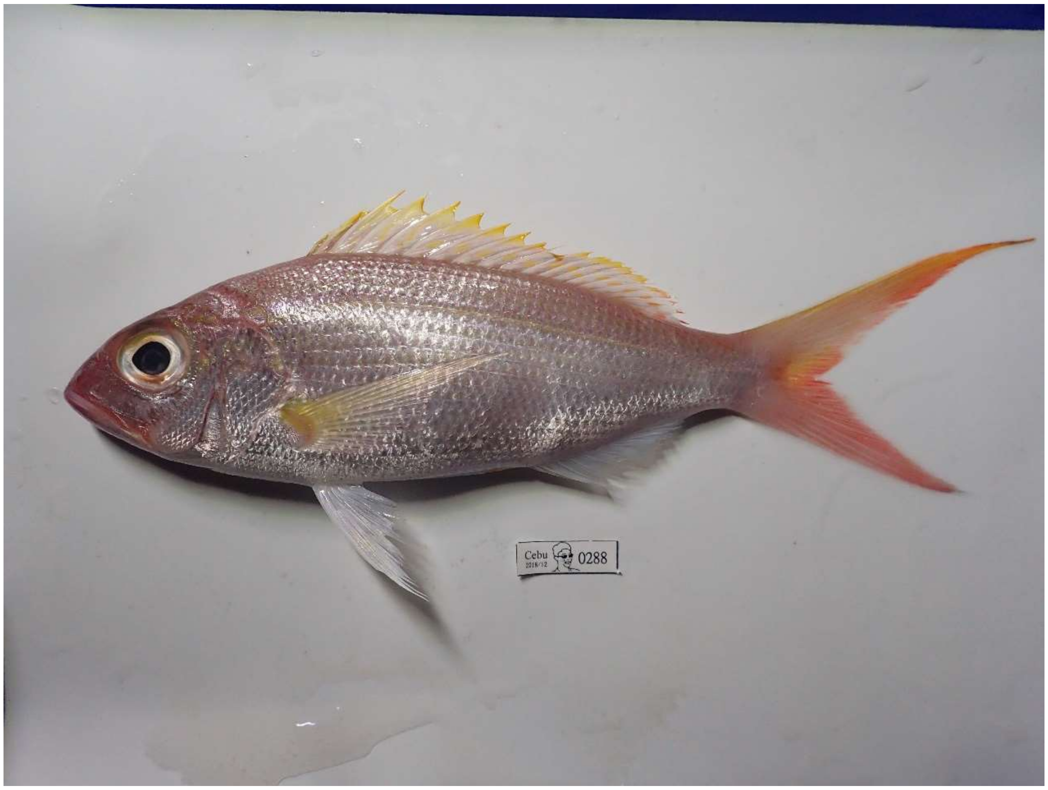

DOS 06837-1, *Pristipomoides typus*, OR114013. (specimen not preserved)

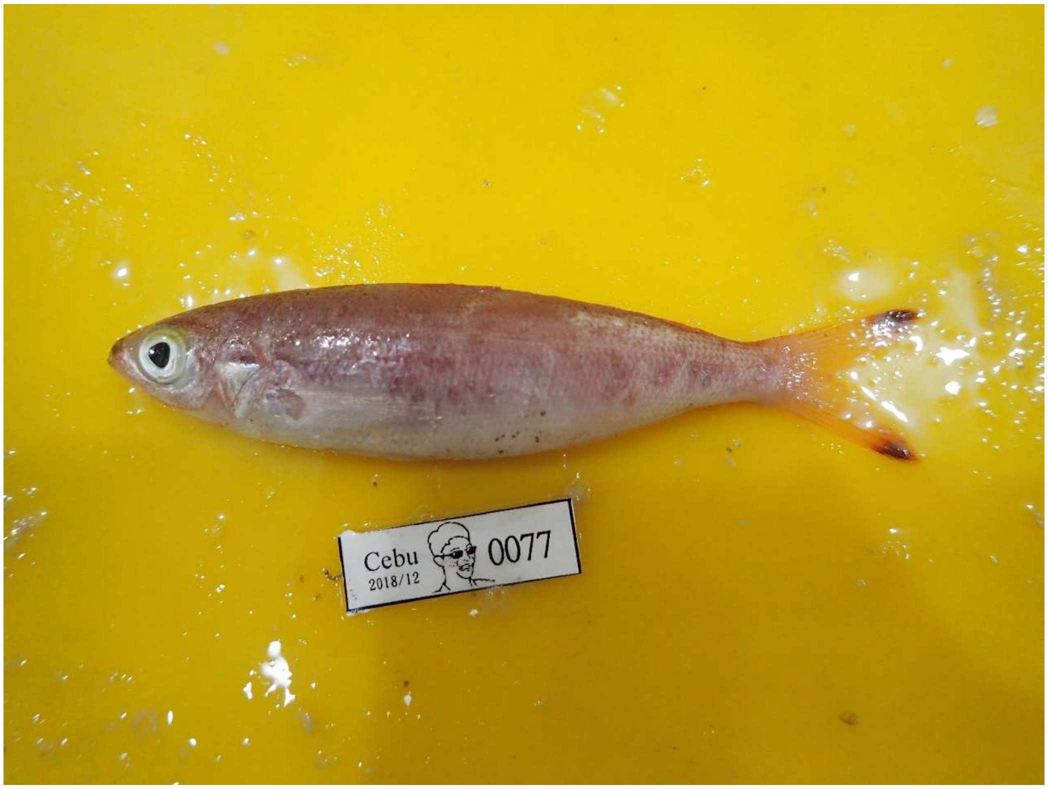

DOS 06629, *Pterocaesio pisang*, OR113820. (specimen not preserved)

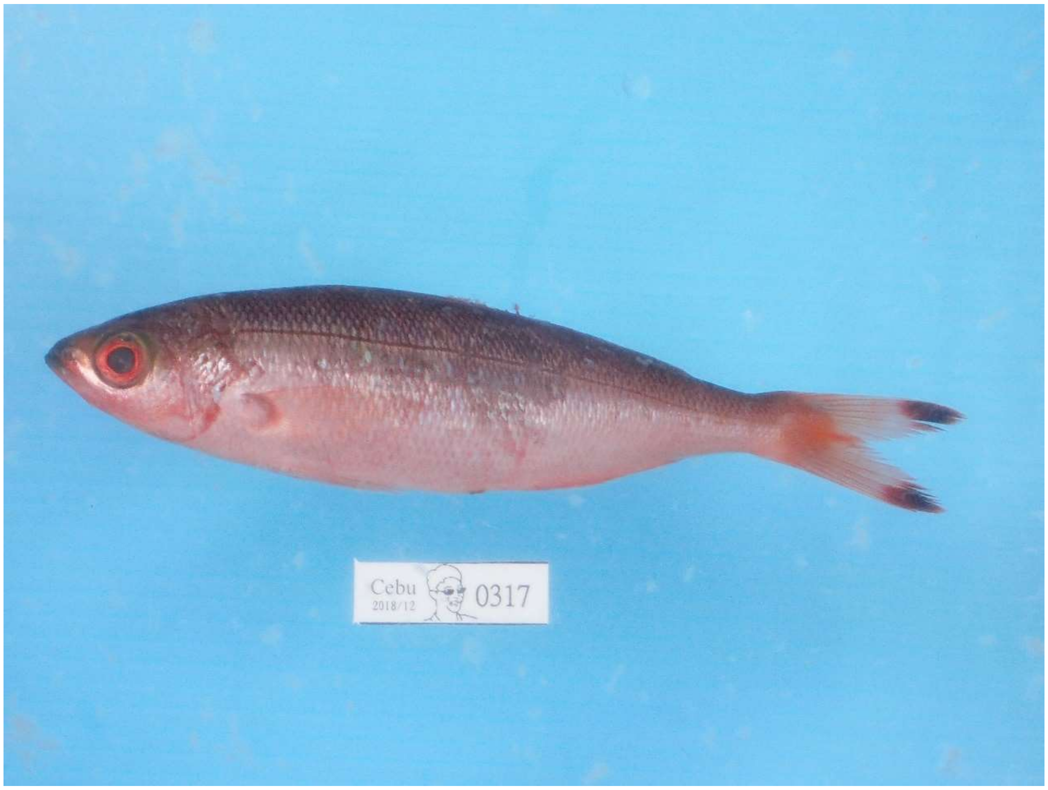

DOS 06631, *Pterocaesio pisang*, OR113823.

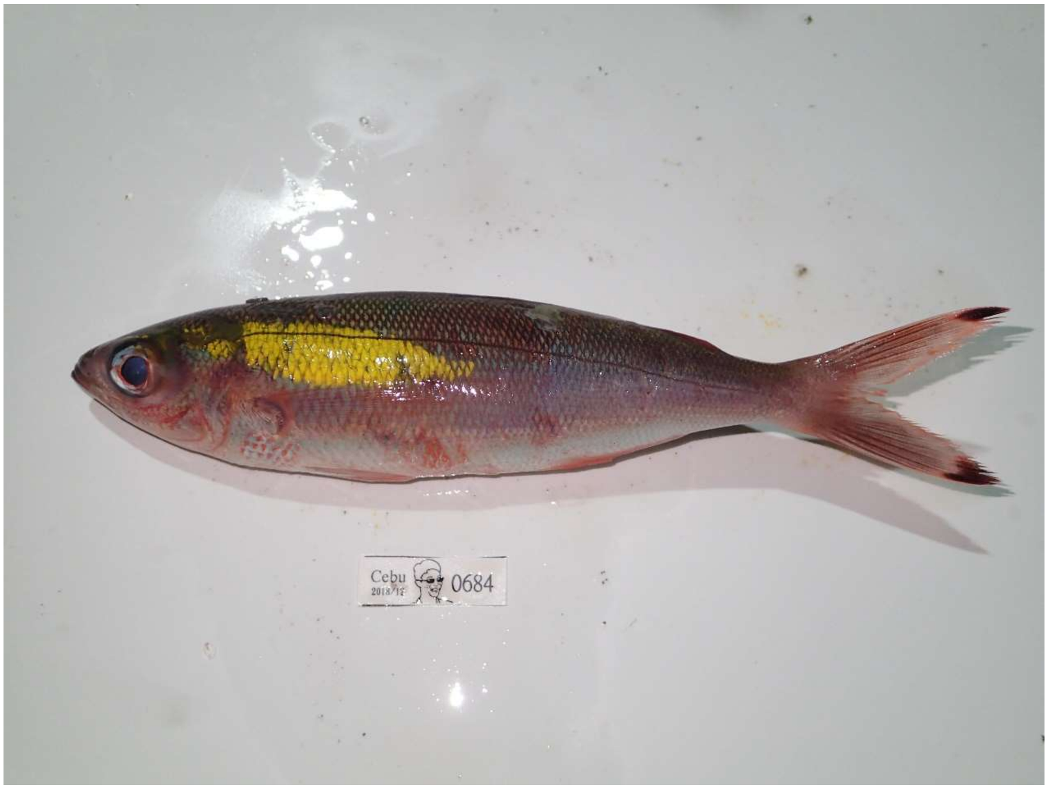

DOS 06628-1, *Pterocaesio randalli*, OR113819.

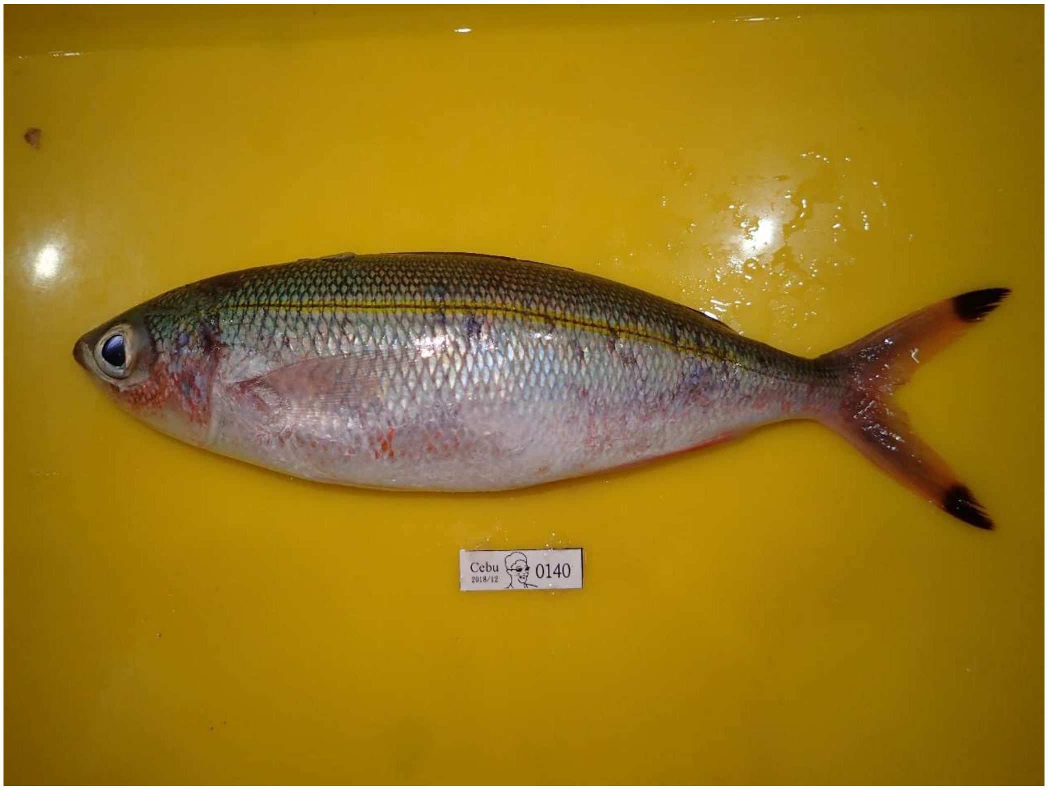

DOS 06627-1, *Pterocaesio tessellata*, OR113818. (specimen not preserved)

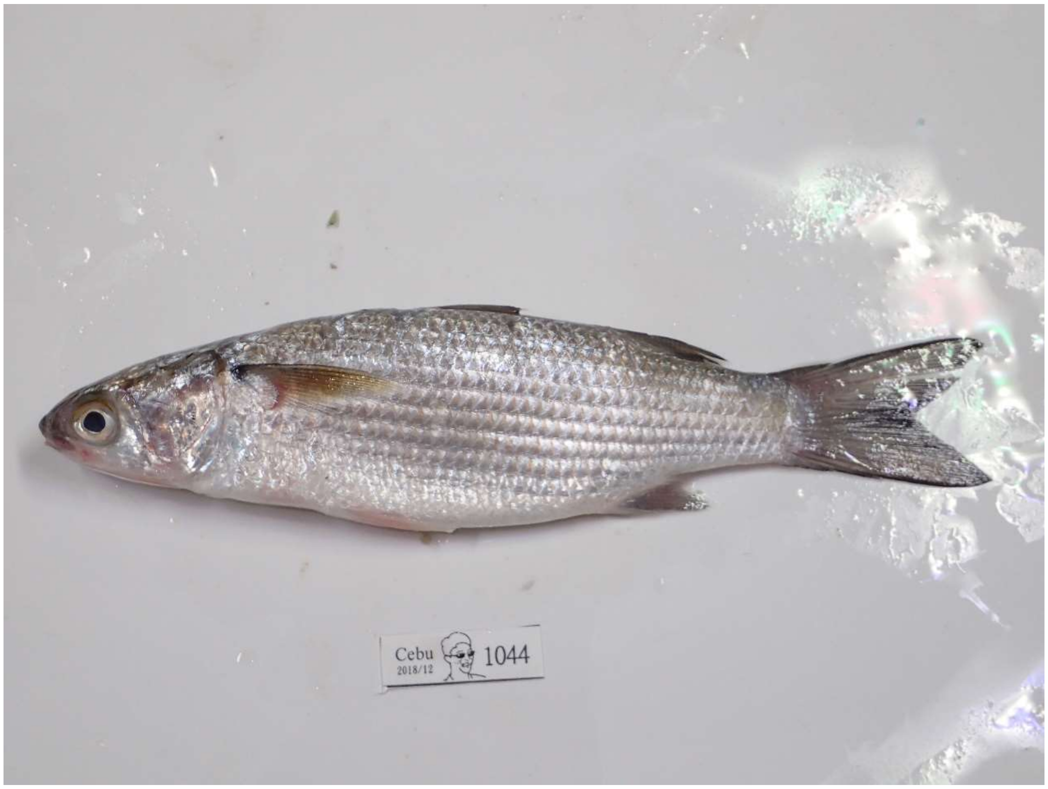

DOS 06807-4, *Crenimugil seheli*, OR113989.

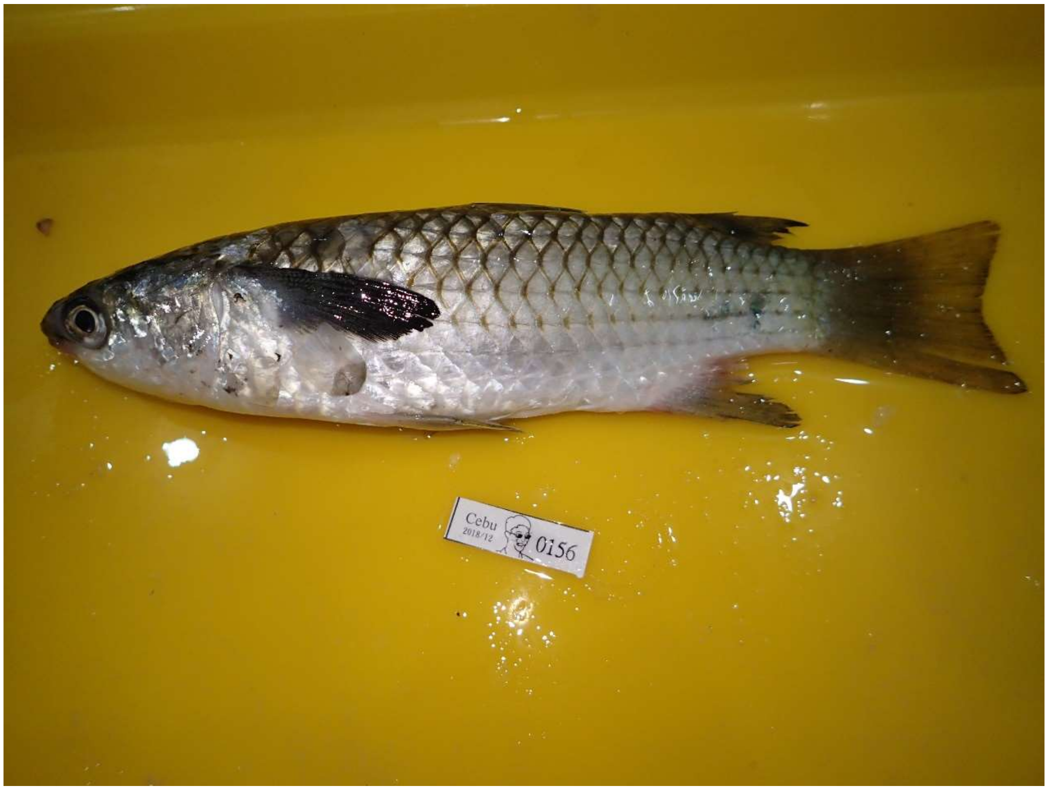

DOS 06806-1, *Ellochelon vaigiensis*, OR113988.

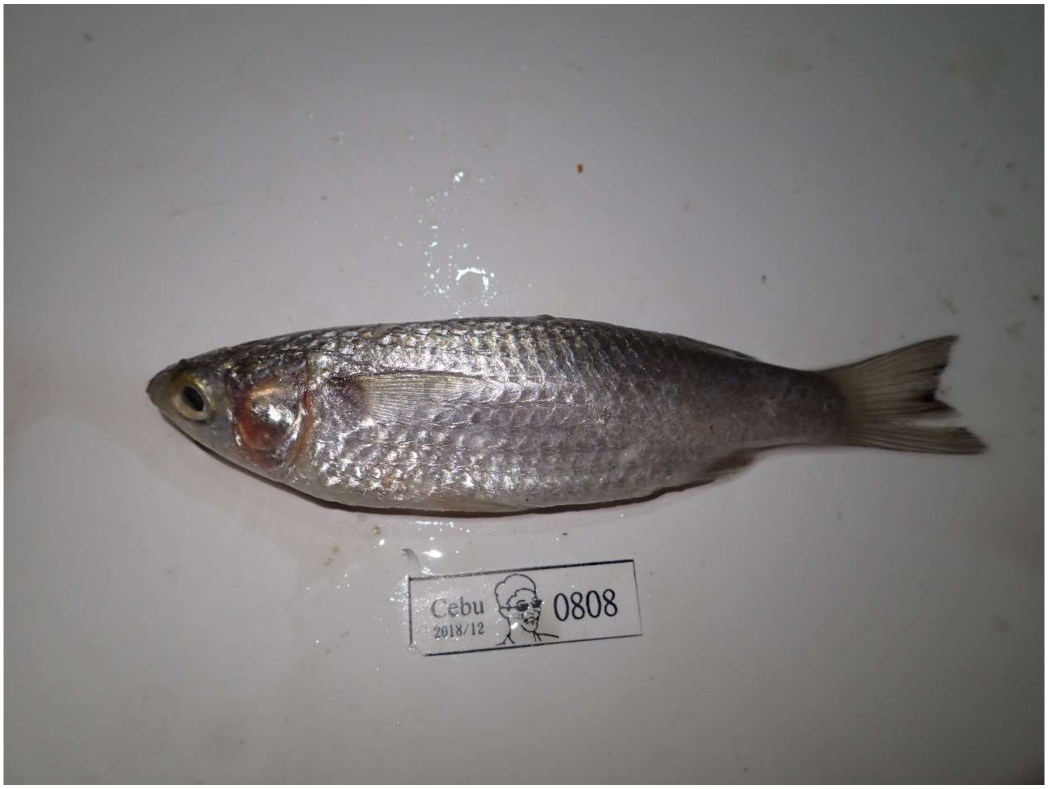

DOS 06808-1, *Osteomugil engeli*, OR113990.

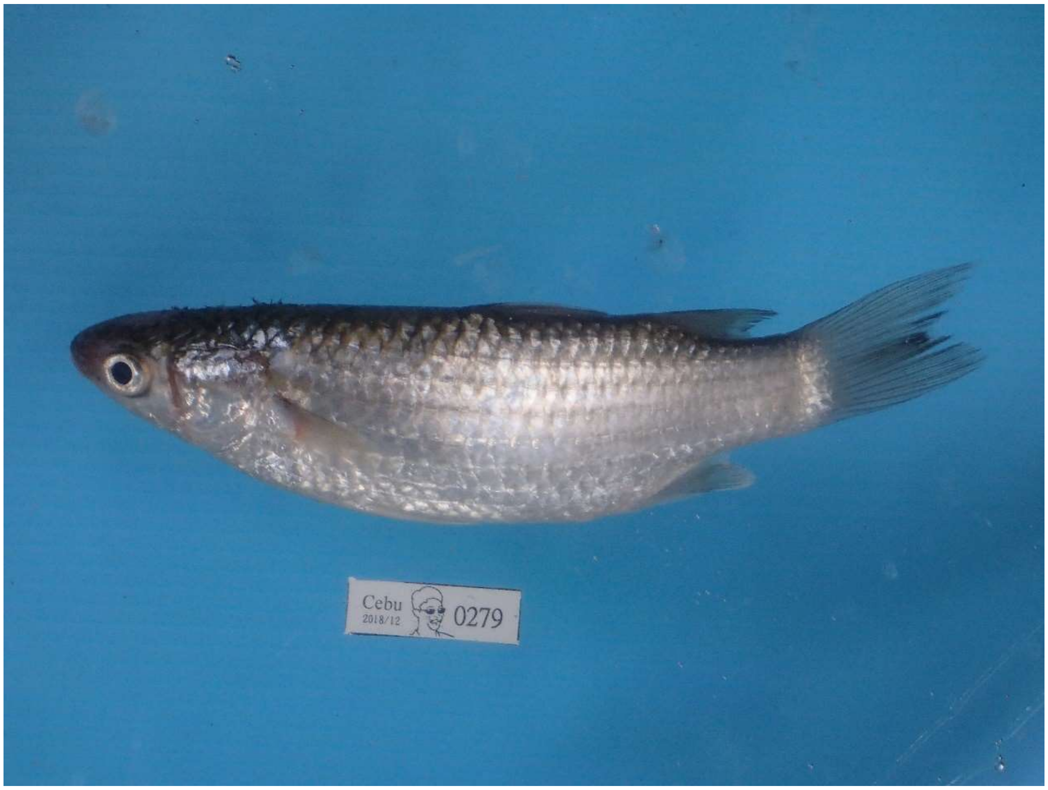

DOS 06805-1, *Planiliza* sp., OR113987. (specimen not preserved)

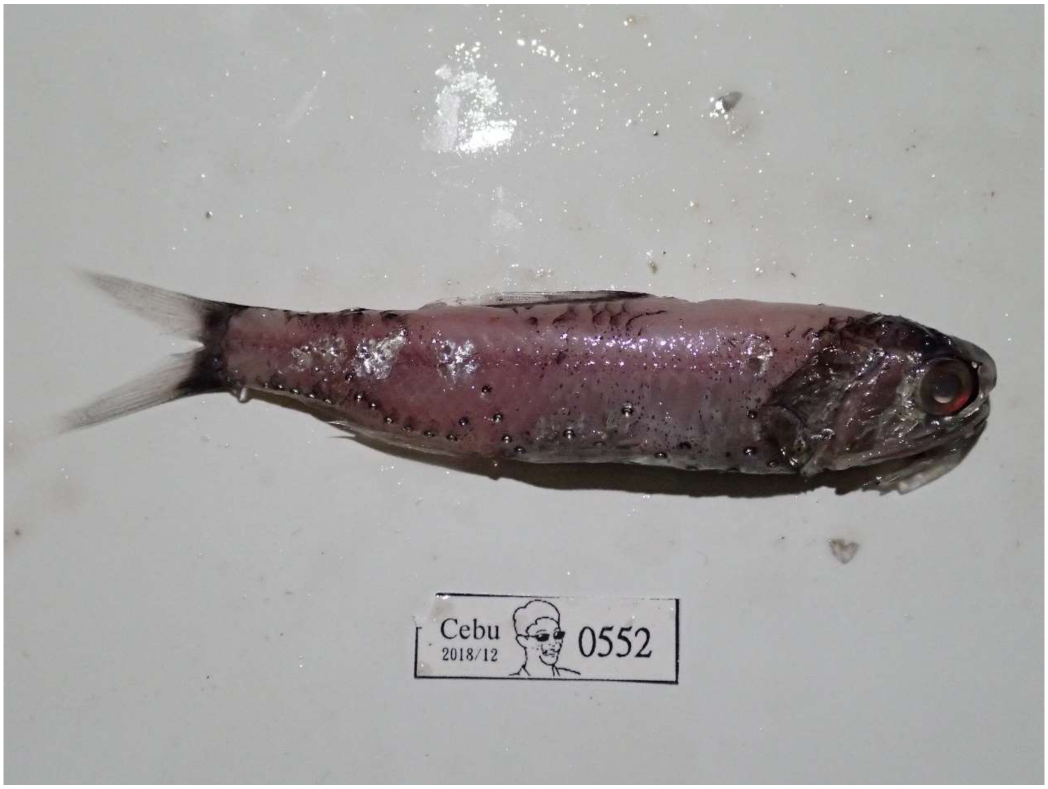

DOS 06835, *Diaphus watasei*, OR114011.

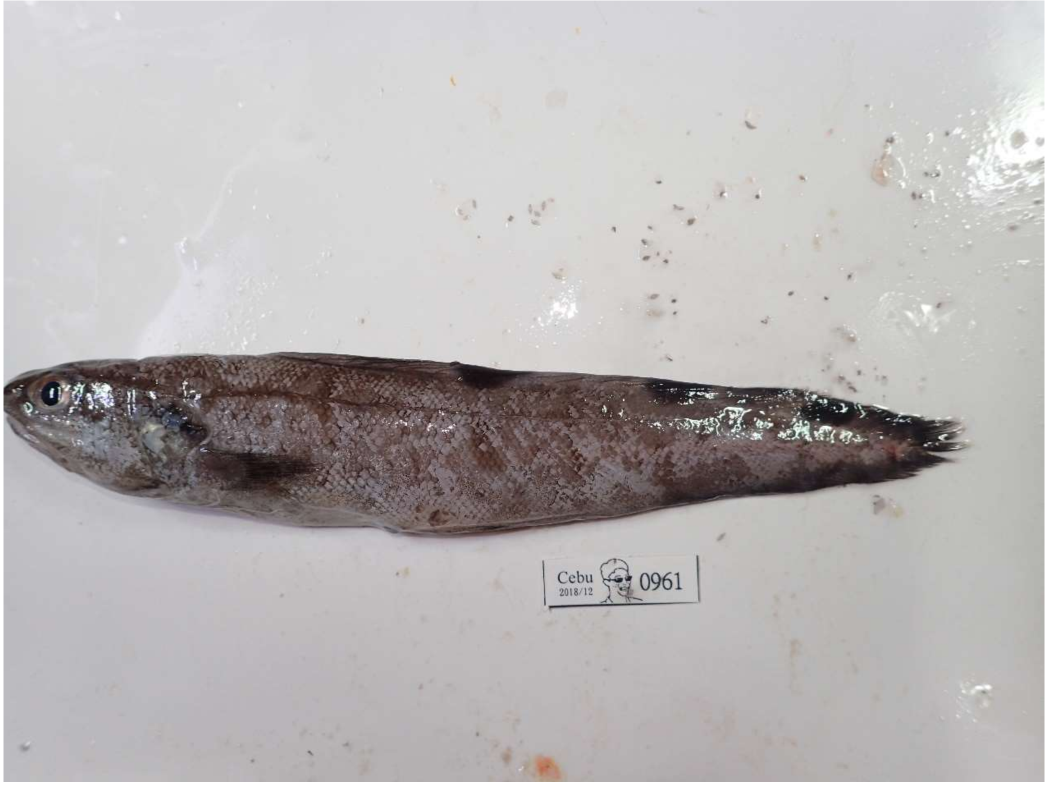

DOS 06861, *Neobythites bimaculatus*, OR114037.

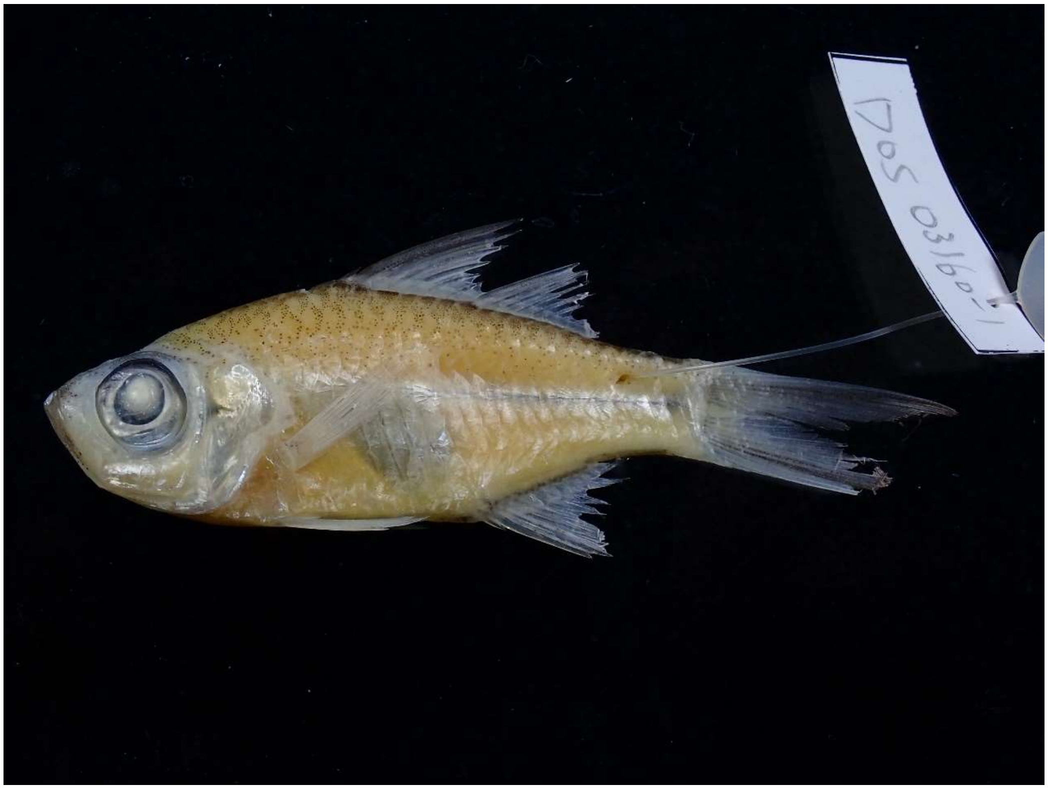

DOS 03160-1, *Ambassis urotaenia*, OR113755.

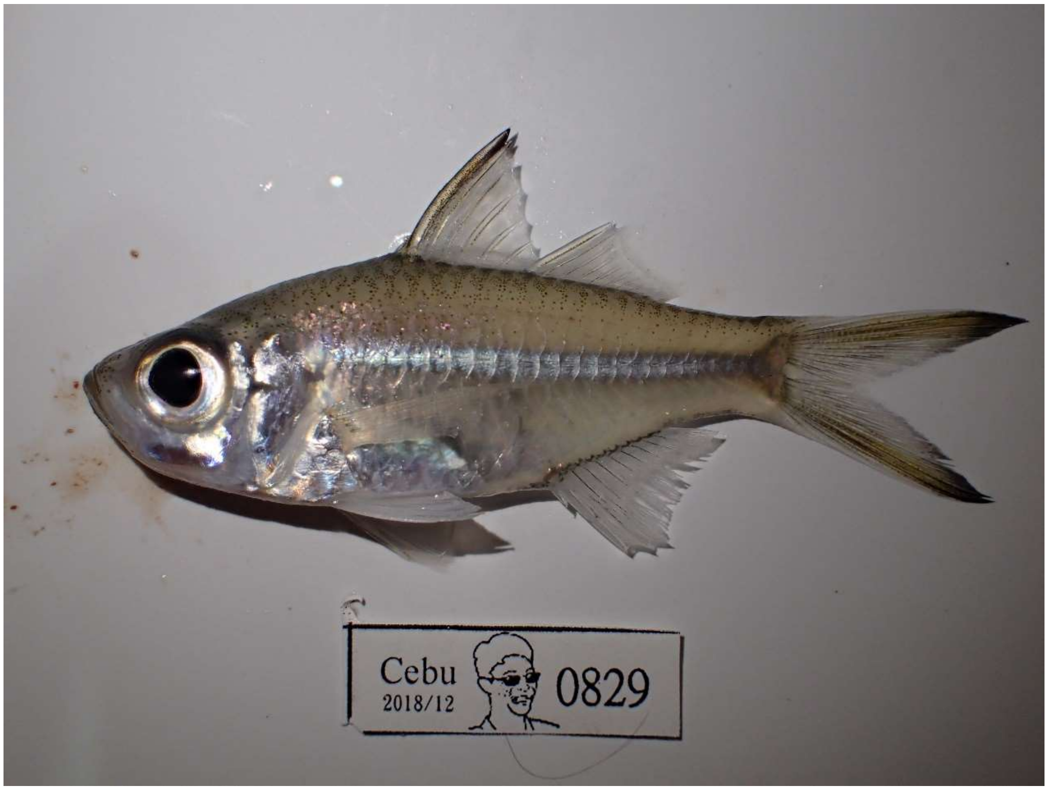

DOS 06998, *Ambassis urotaenia*, OR114165.

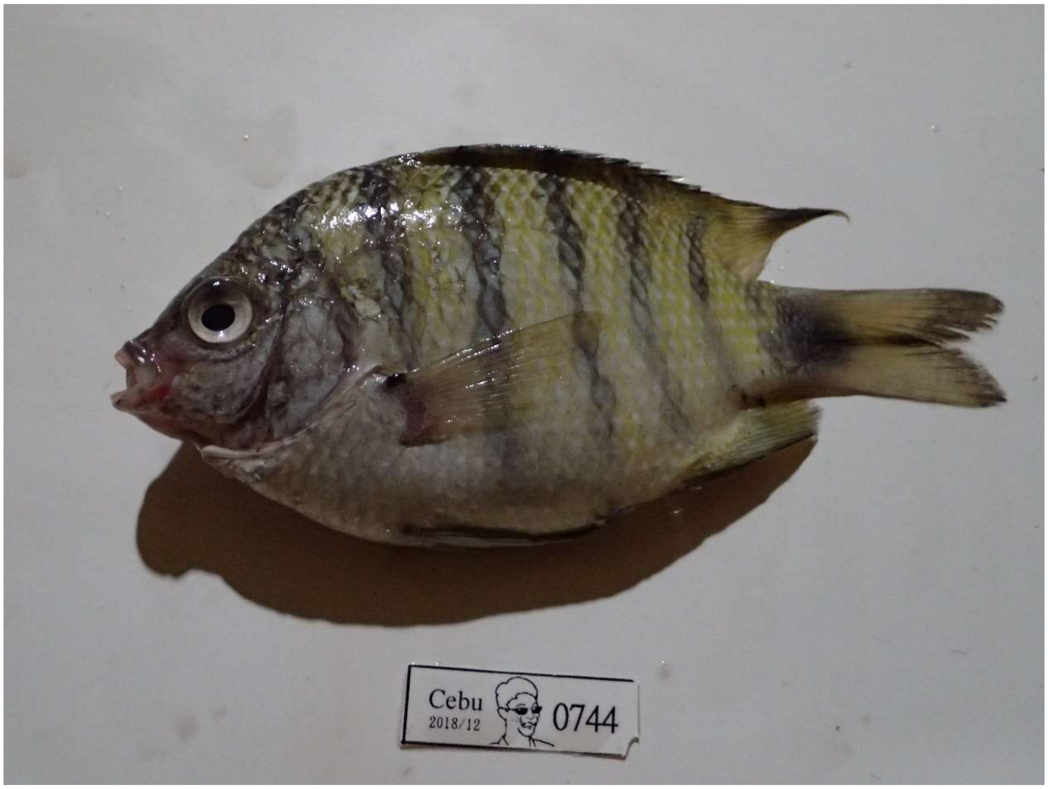

DOS 06884, *Abudefduf bengalensis*, OR114061.

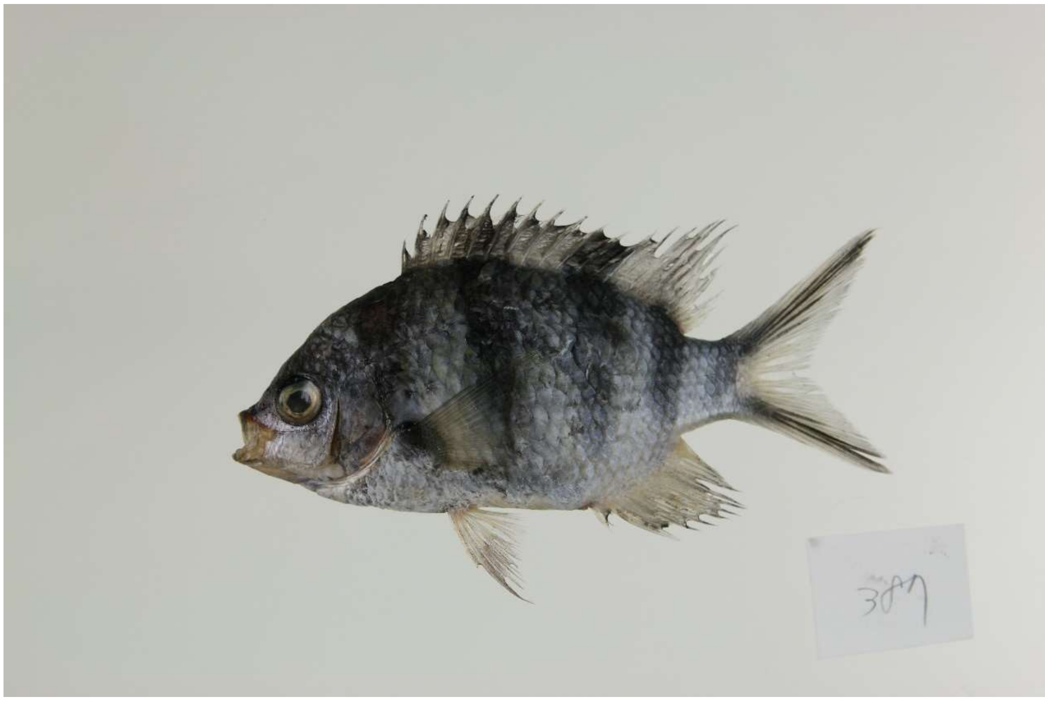

DOS 08650, *Abudefduf sexfasciatus*, OR114225.

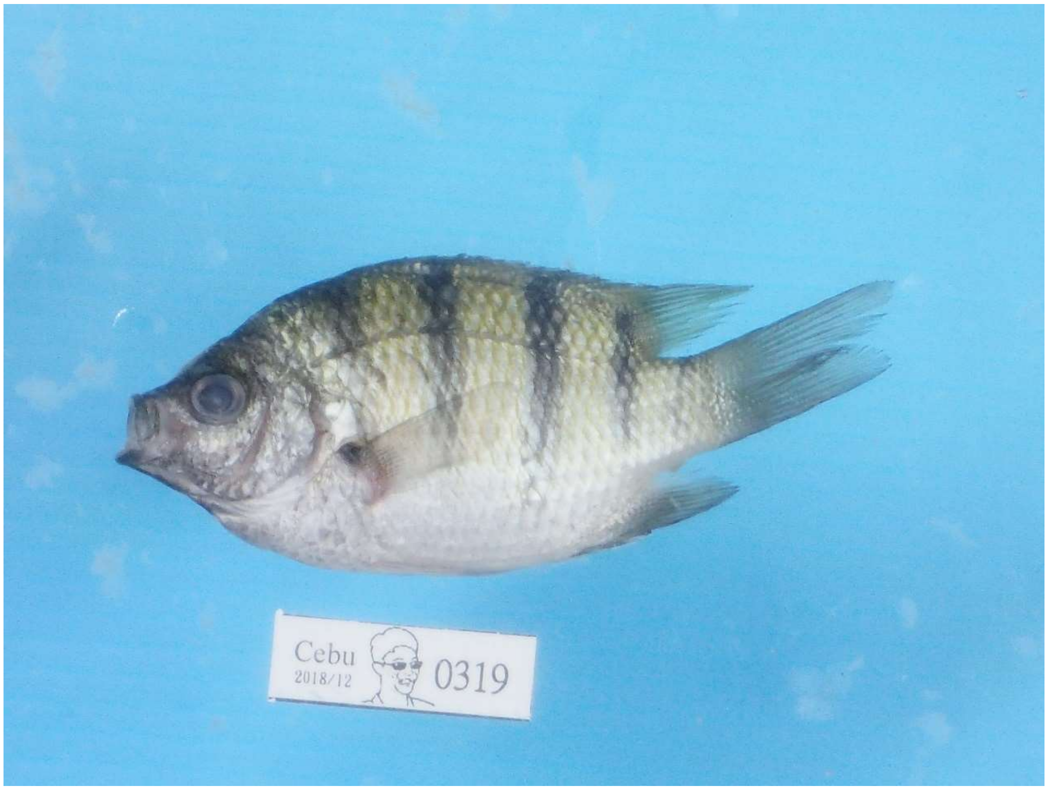

DOS 06887, *Abudefduf* sp., OR114064.

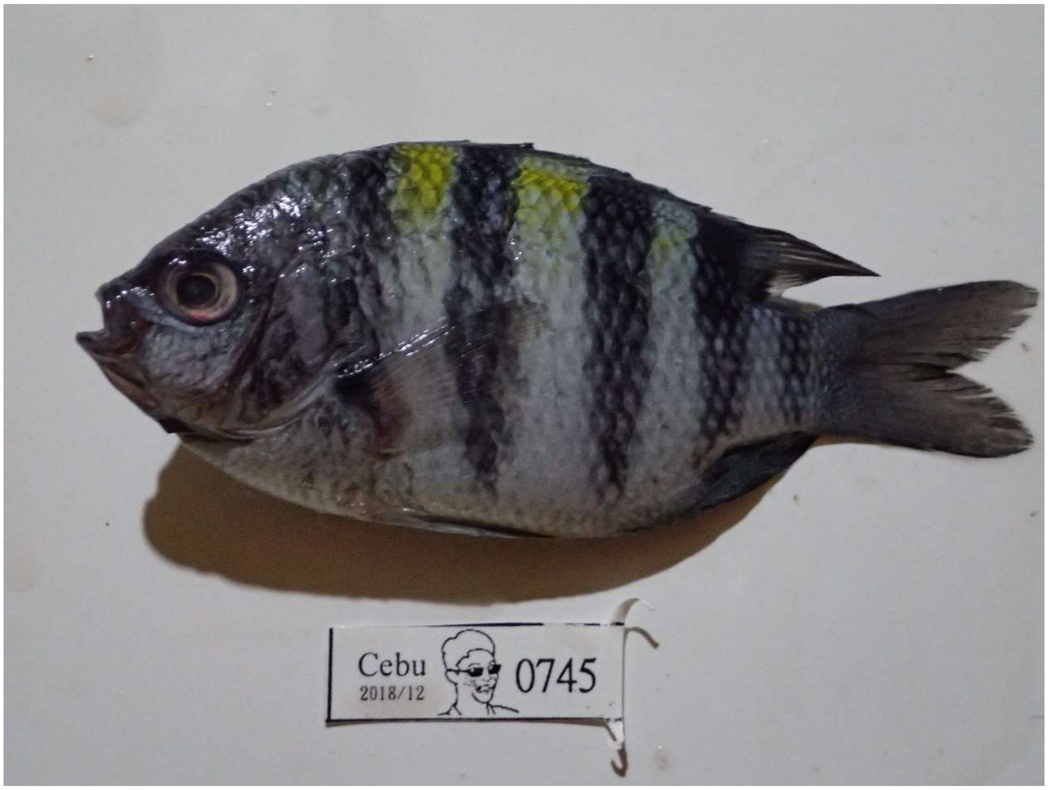

DOS 06885-1, *Abudefduf vaigiensis*, OR114062.

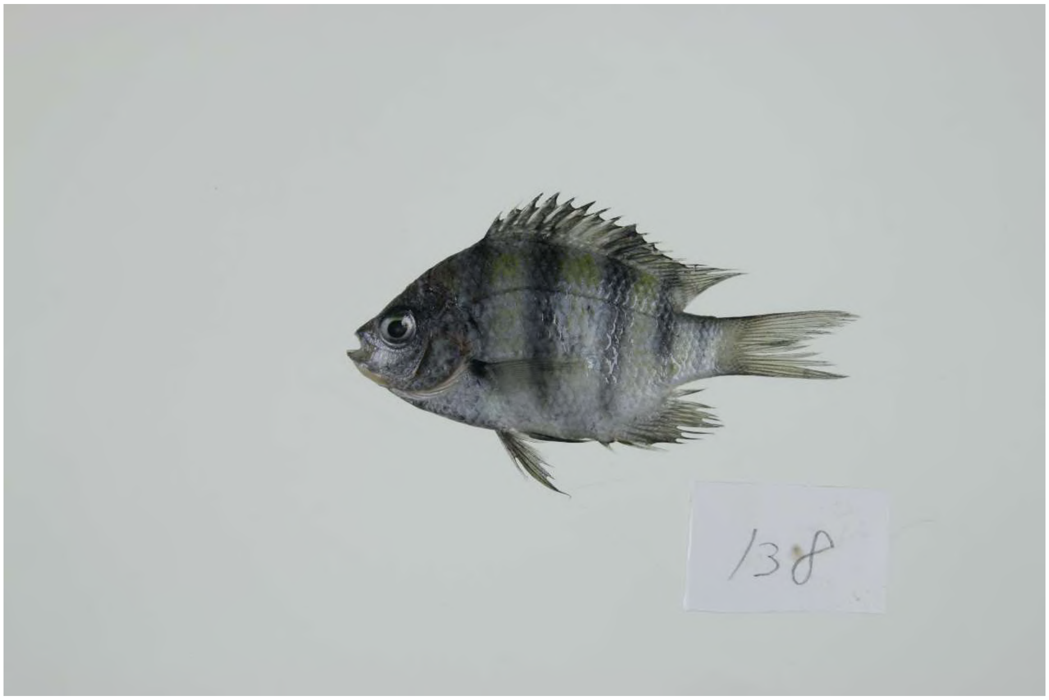

DOS 08651, *Abudefduf vaigiensis*, OR114226.

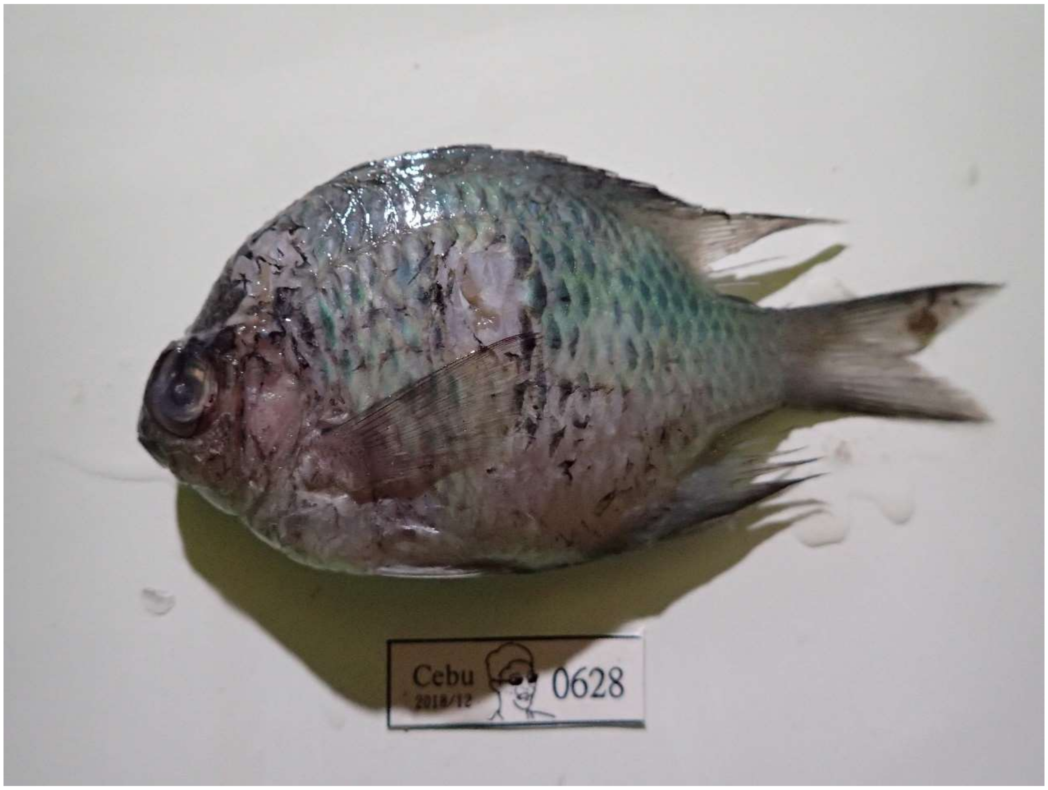

DOS 06901, *Amblyglyphidodon curacao*, OR114078.

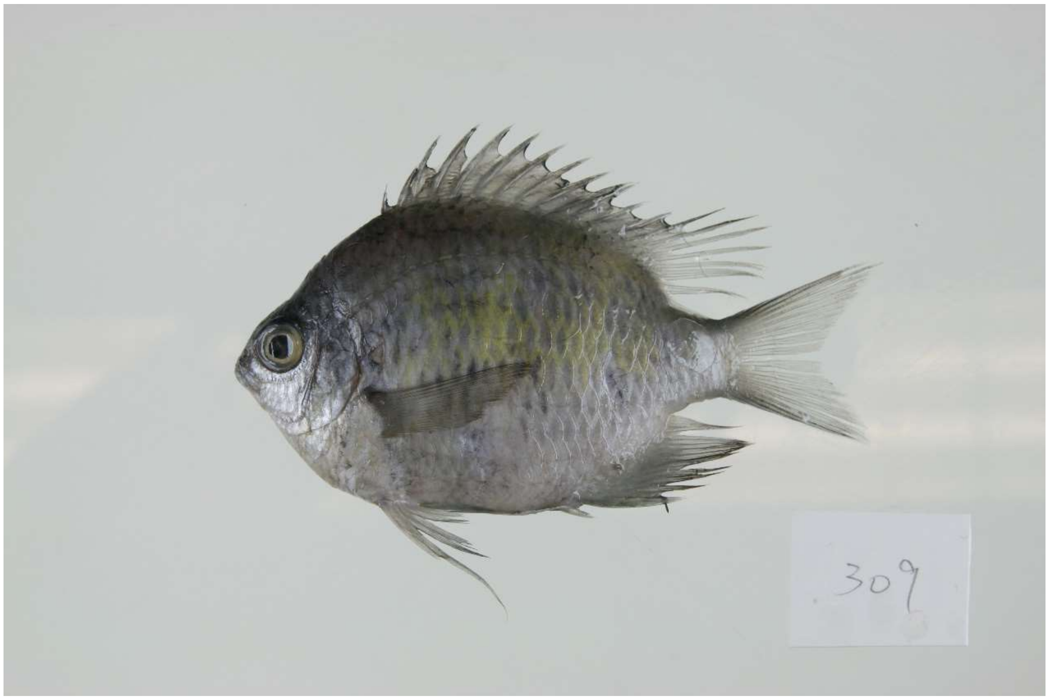

DOS 08652, *Amblyglyphidodon curacao*, OR114227.

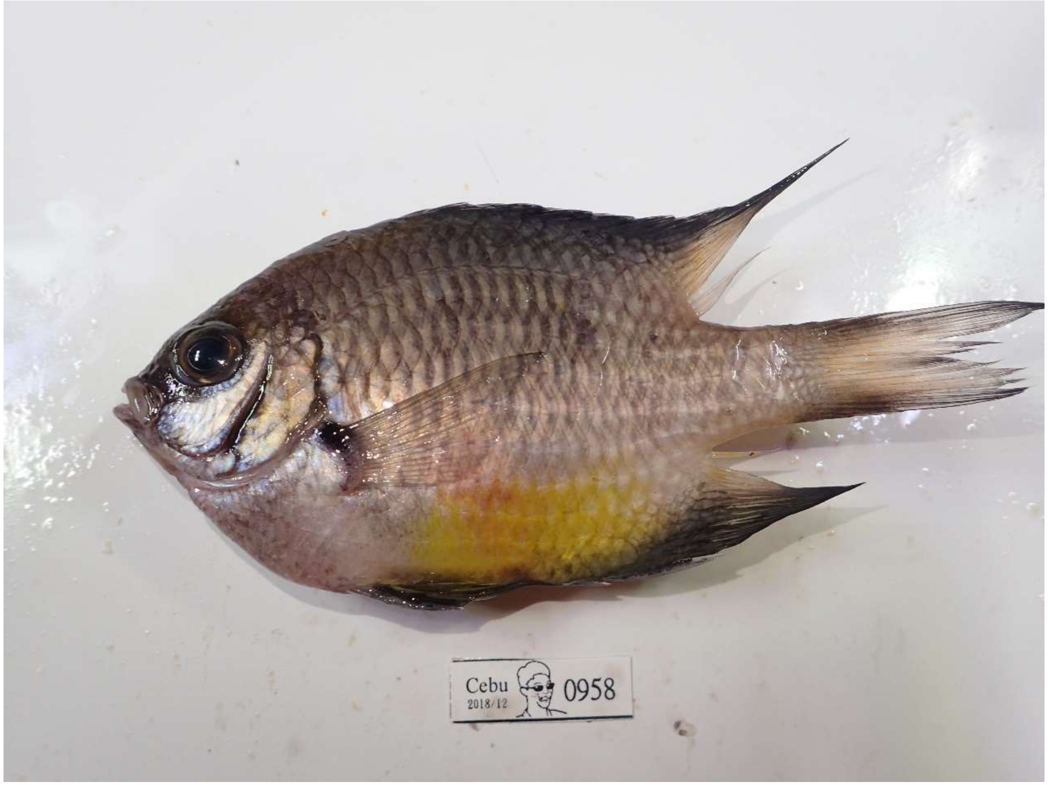

DOS 06888, *Amblyglyphidodon leucogaster*, OR114065.

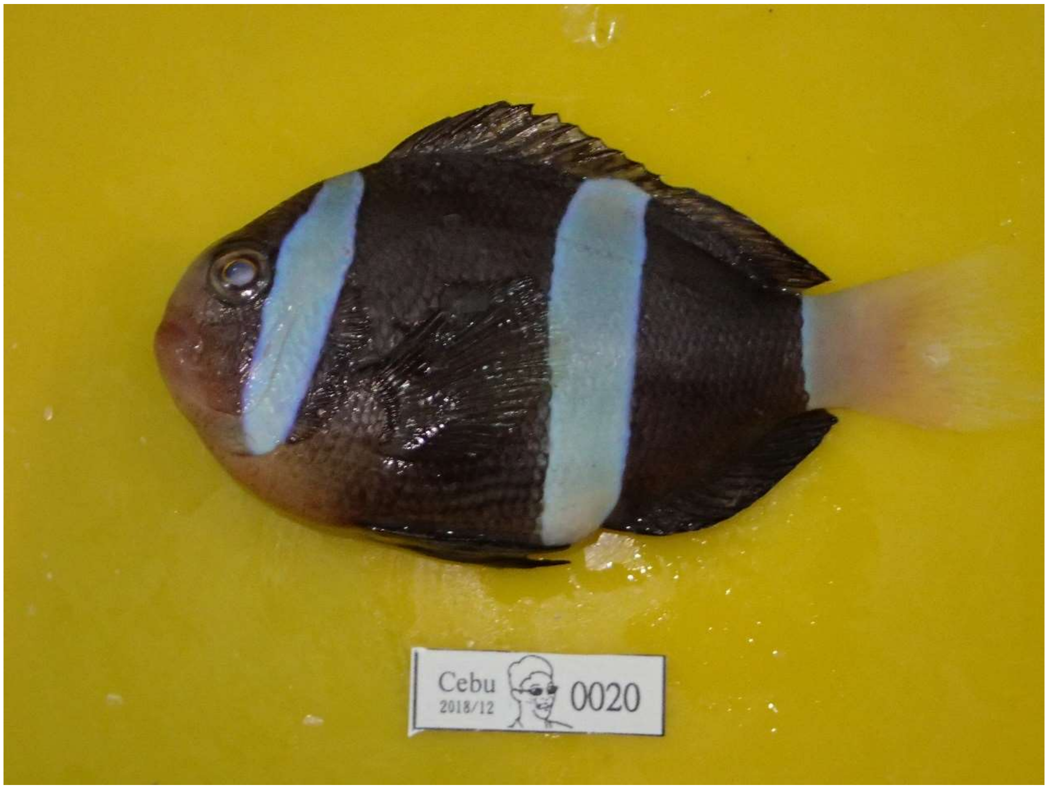

DOS 06889-1, *Amphiprion clarkii*, OR114066. (specimen not preserved)

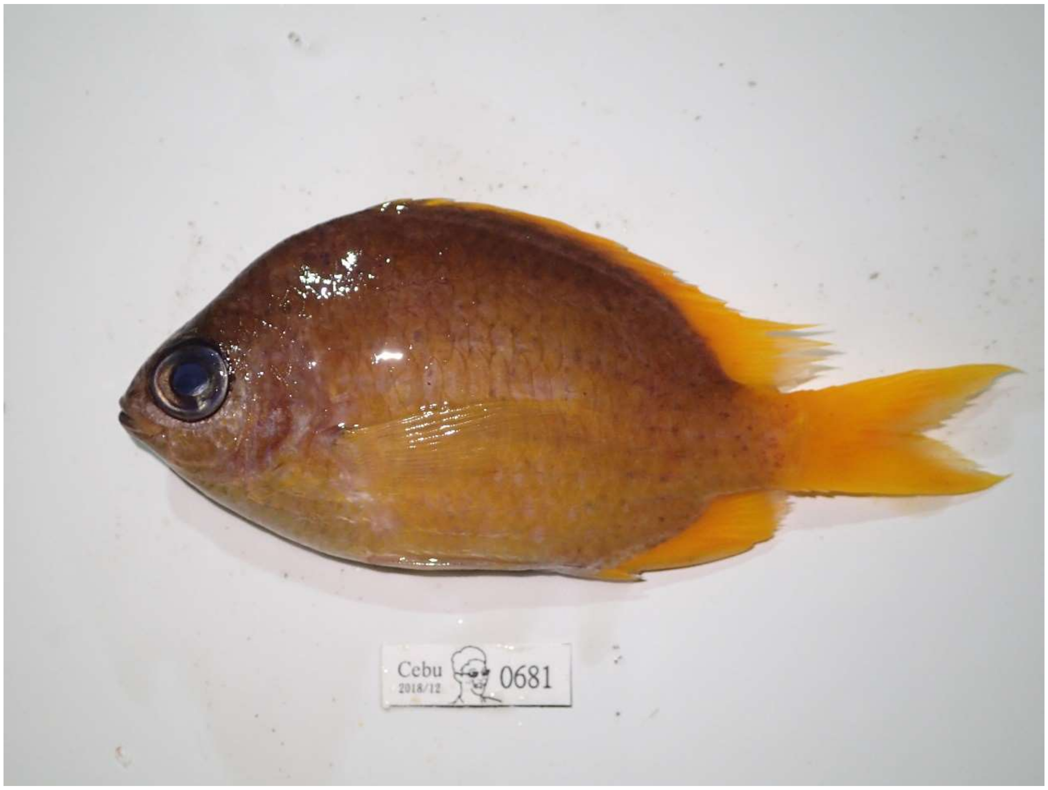

DOS 06890-1, *Chromis analis*, OR114067.

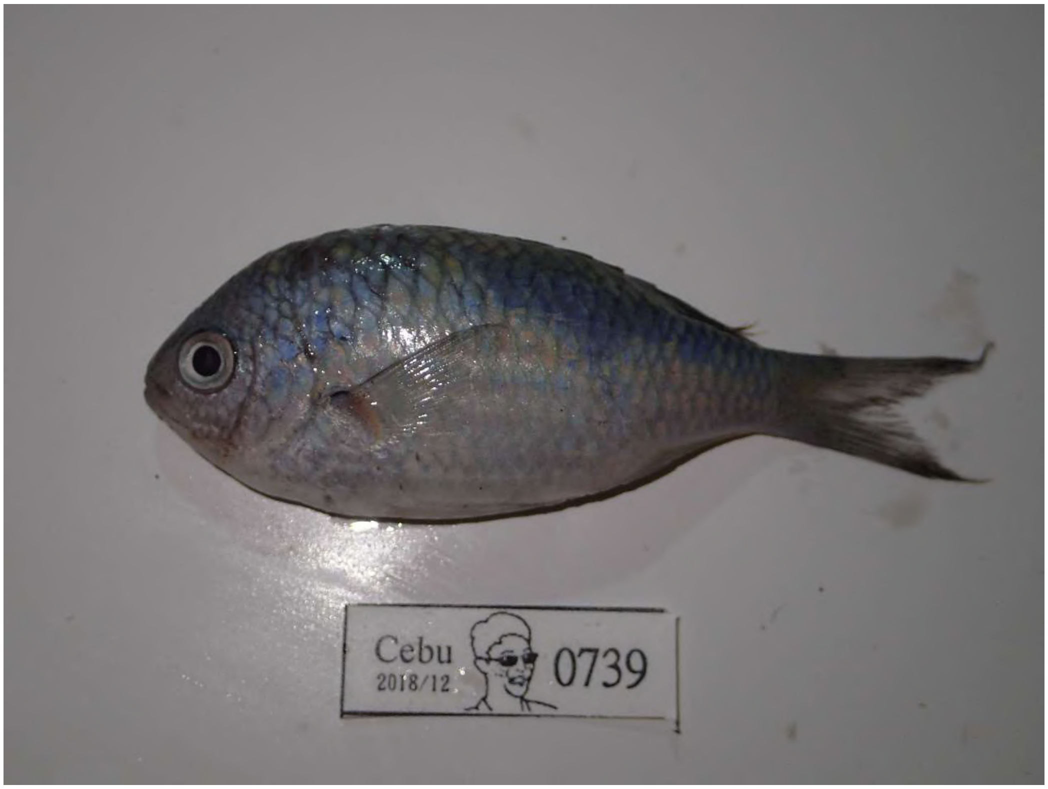

DOS 06892-1, *Chromis ternatensis*, OR114069.

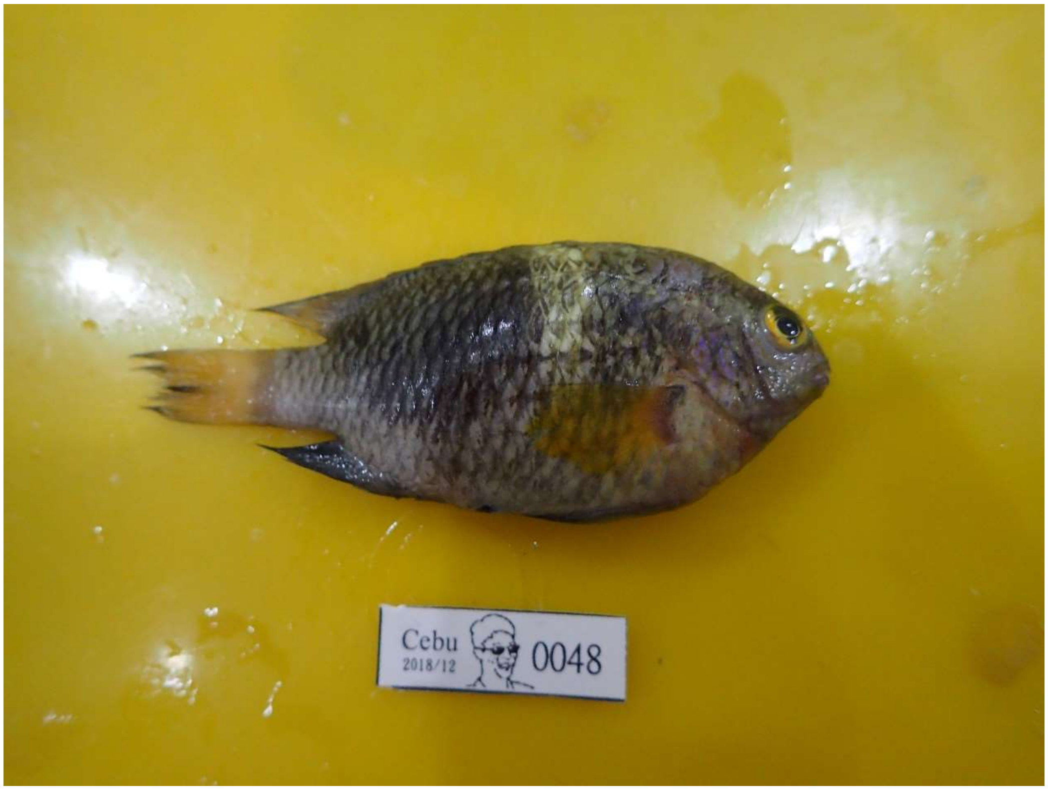

DOS 06878-1, *Chrysiptera biocellata*, OR114055.

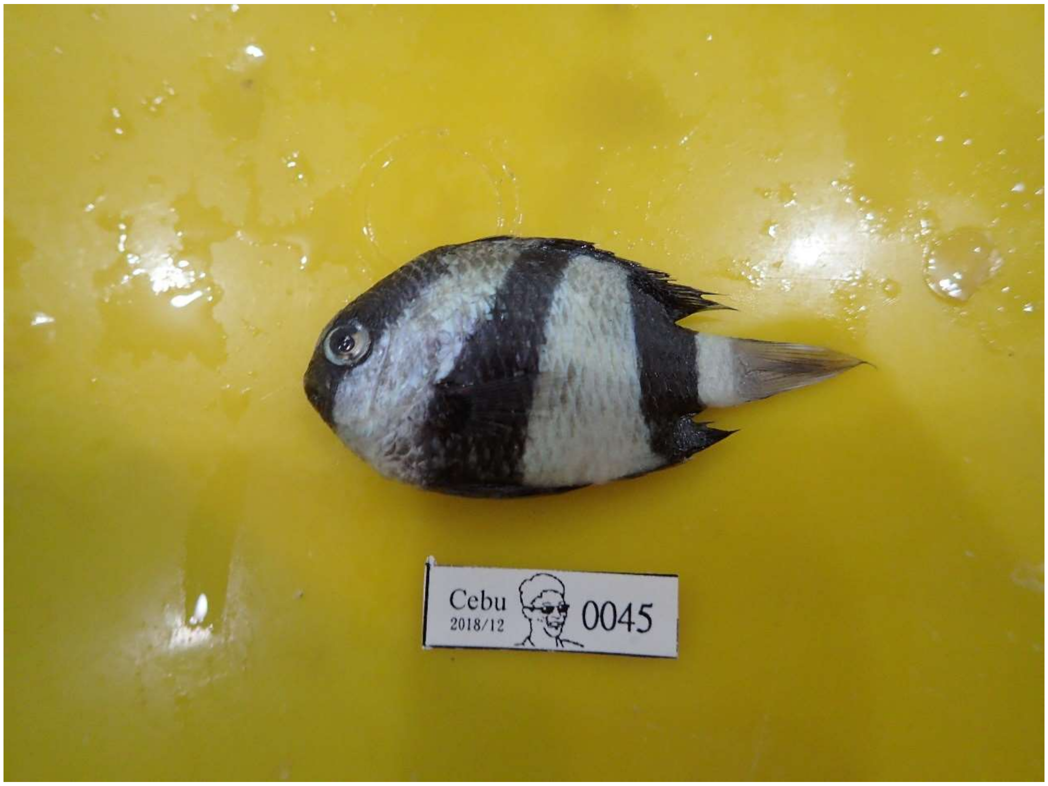

DOS 06880-1, *Dascyllus aruanus*, OR114057. (specimen not preserved)

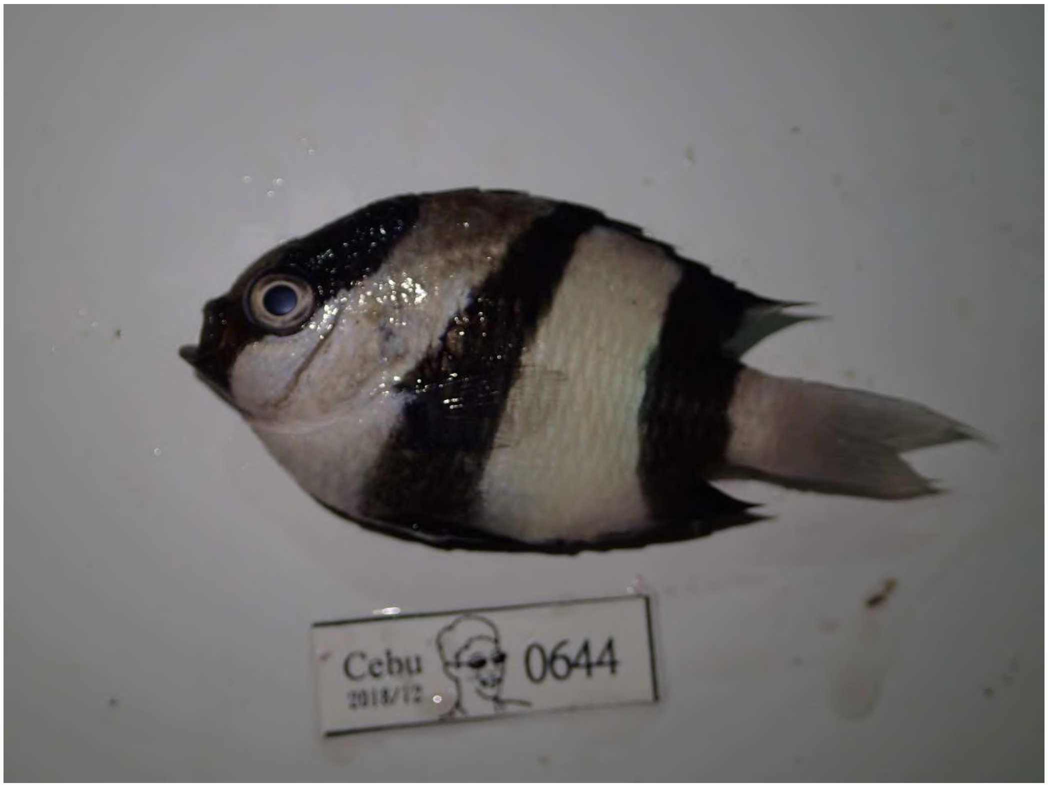

DOS 06896, *Dascyllus aruanus*, OR114073. (specimen not preserved)

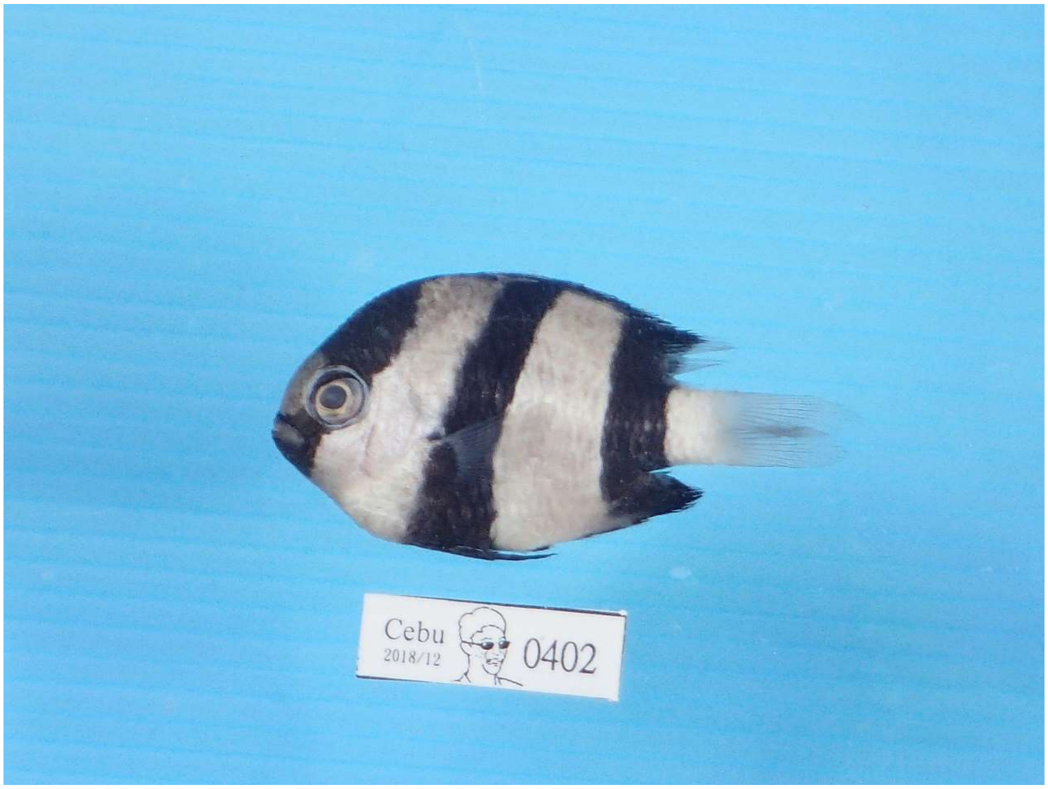

DOS 06897, *Dascyllus aruanus*, OR114074.

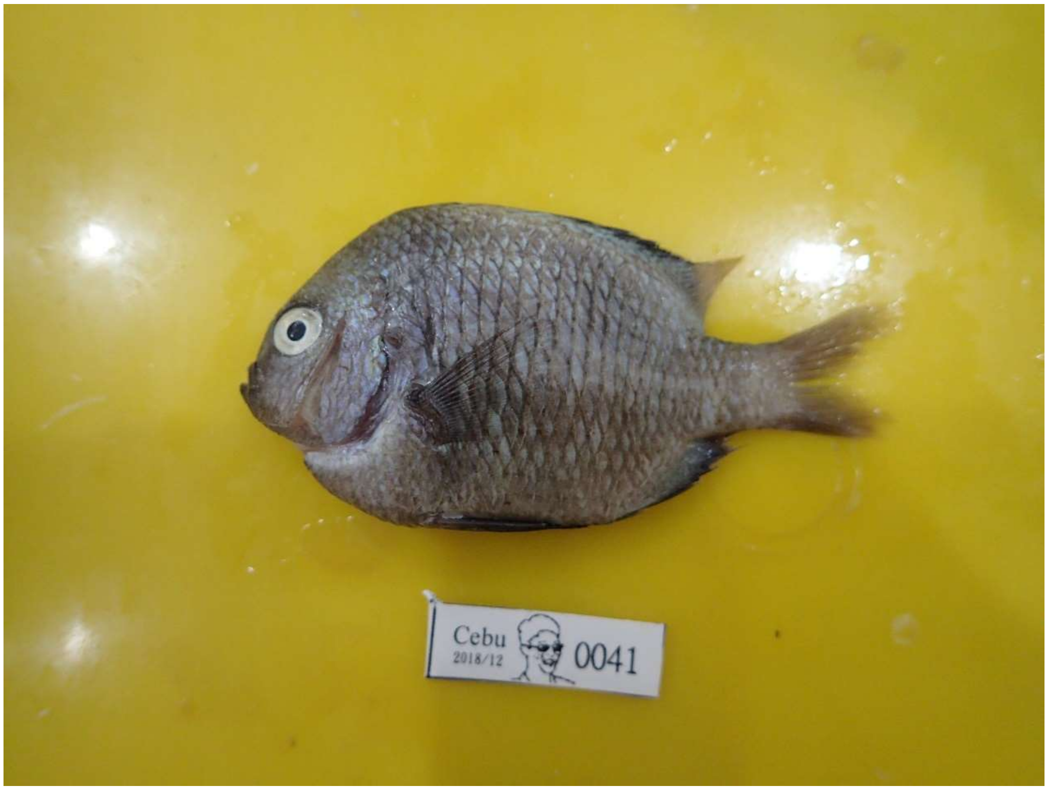

DOS 06898-1, *Dascyllus reticulatus*, OR114075.

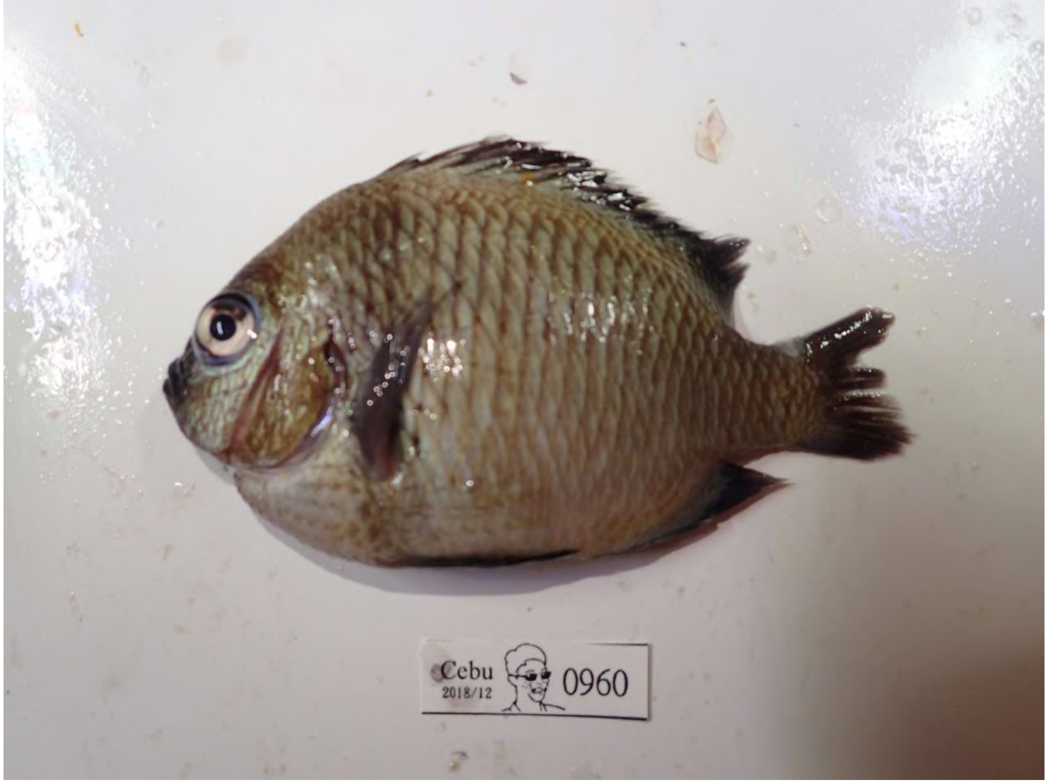

DOS 06899, *Dascyllus reticulatus*, OR114076.

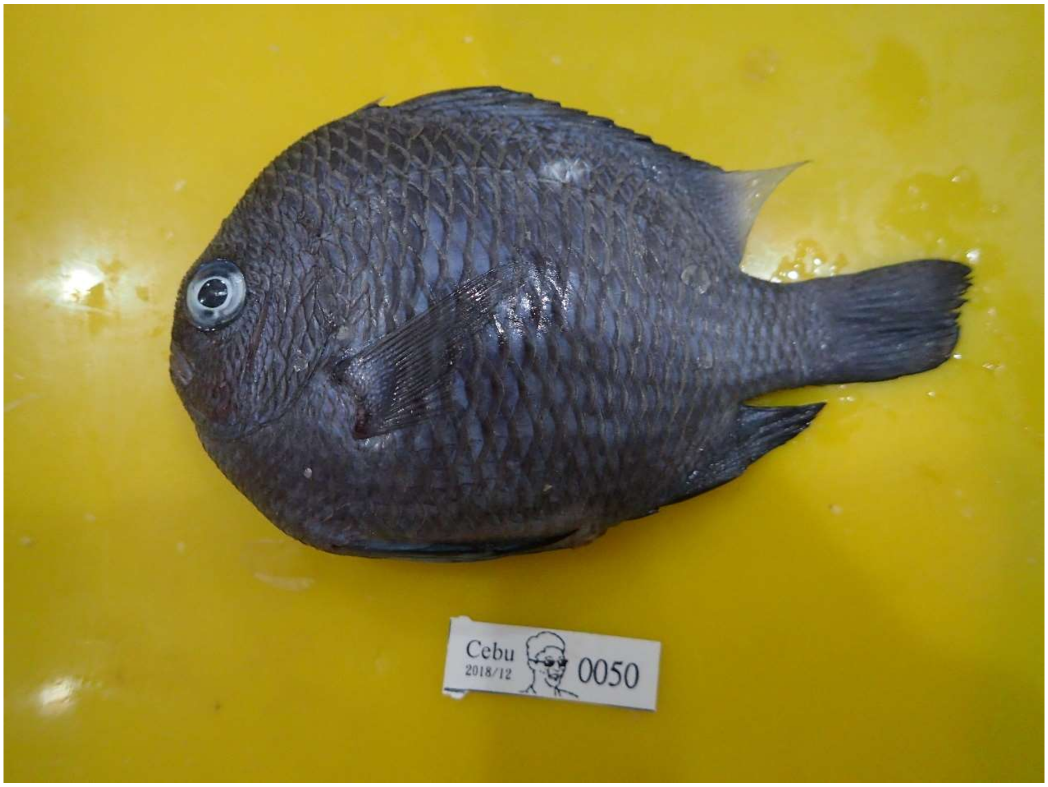

DOS 06881, *Dascyllus trimaculatus*, OR114058. (specimen not preserved)

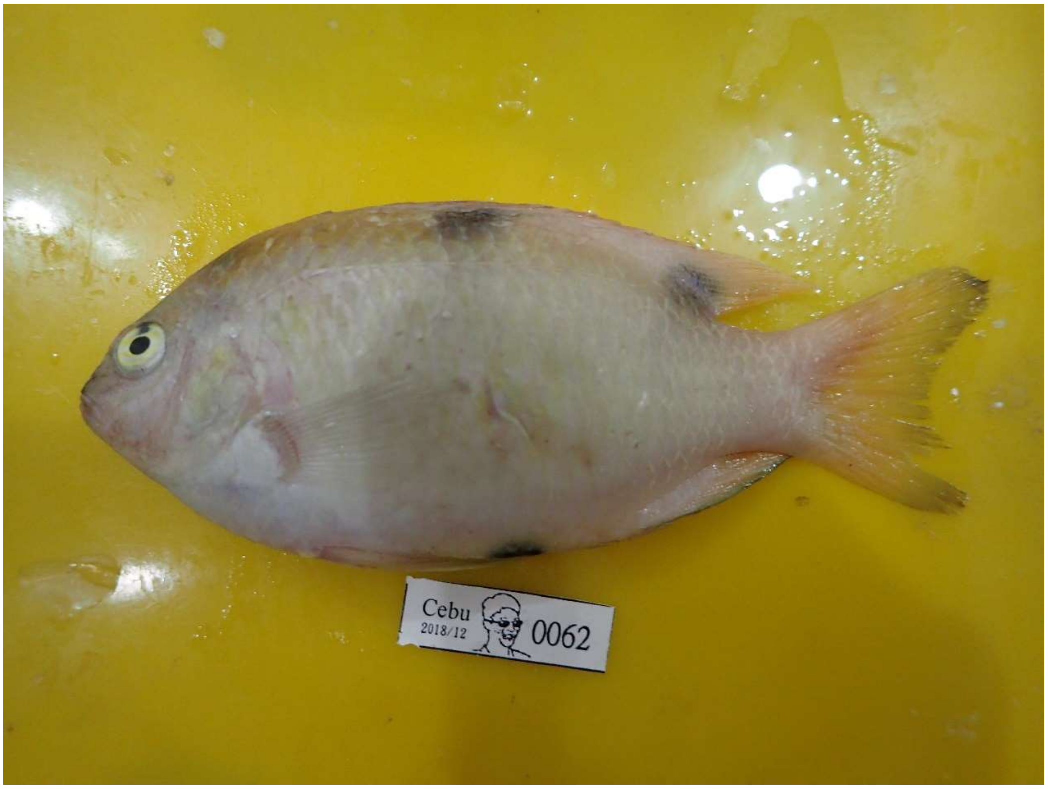

DOS 06882, *Dischistodus perspicillatus*, OR114059.

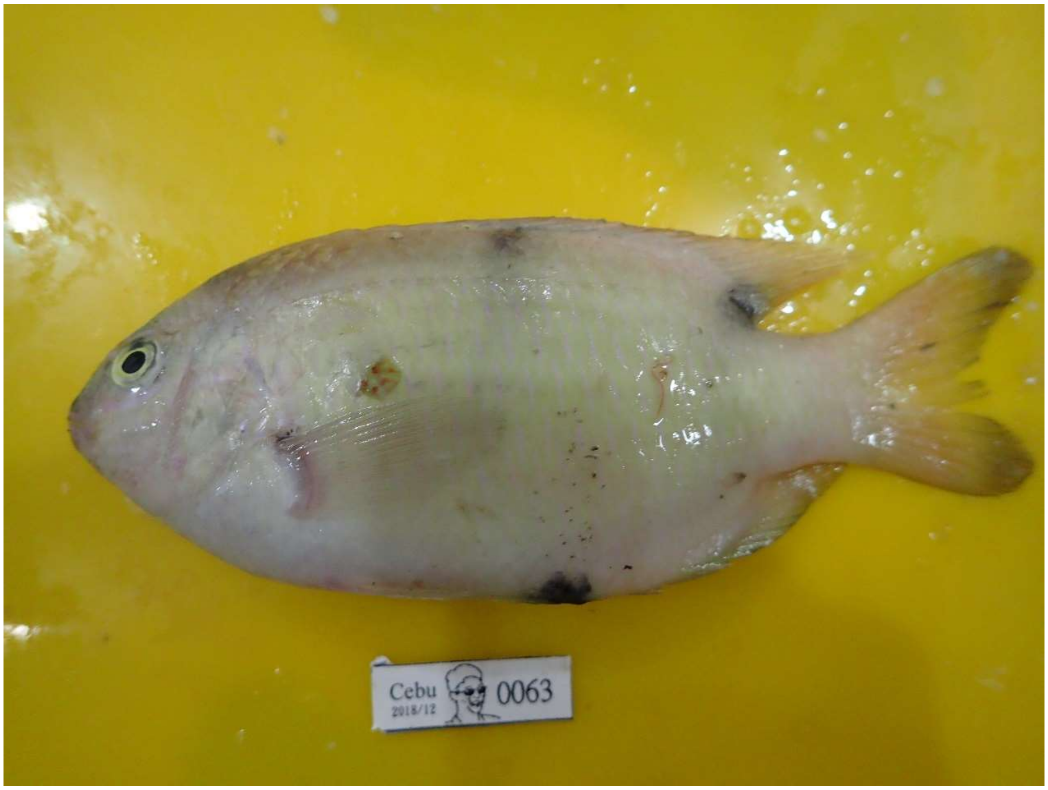

DOS 06883, *Dischistodus perspicillatus*, OR114060.

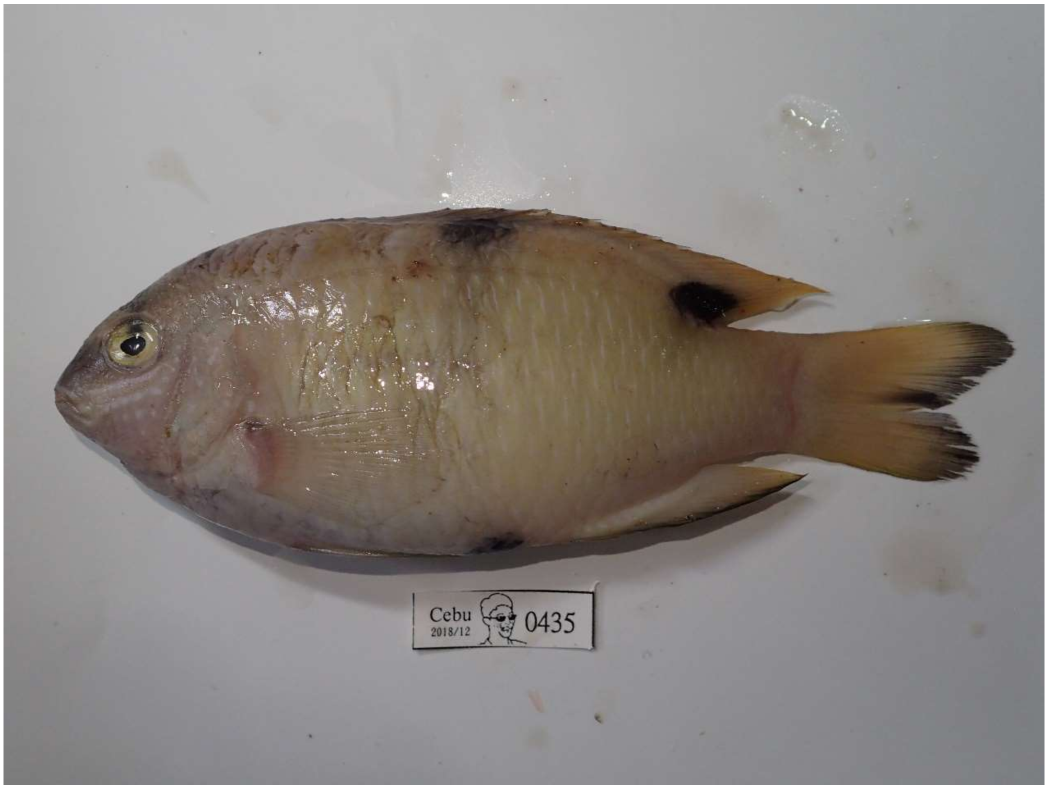

DOS 06907, *Dischistodus perspicillatus*, OR114084.

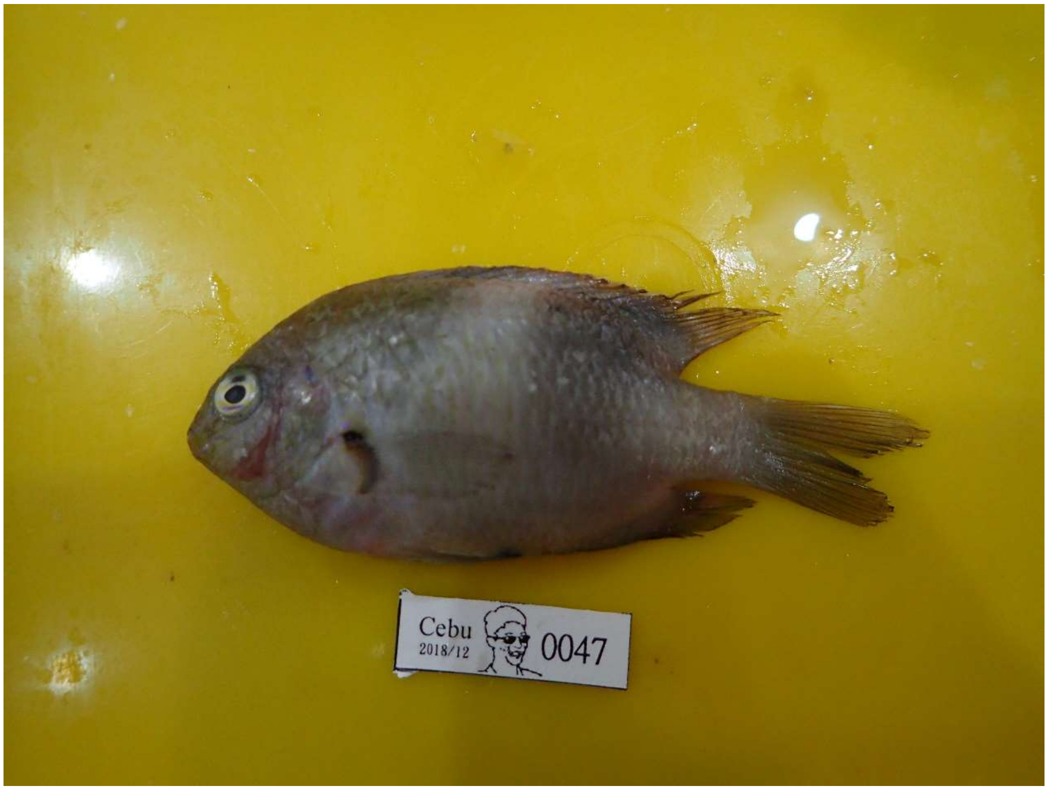

DOS 06879-1, *Dischistodus prosopotaenia,* OR114056.

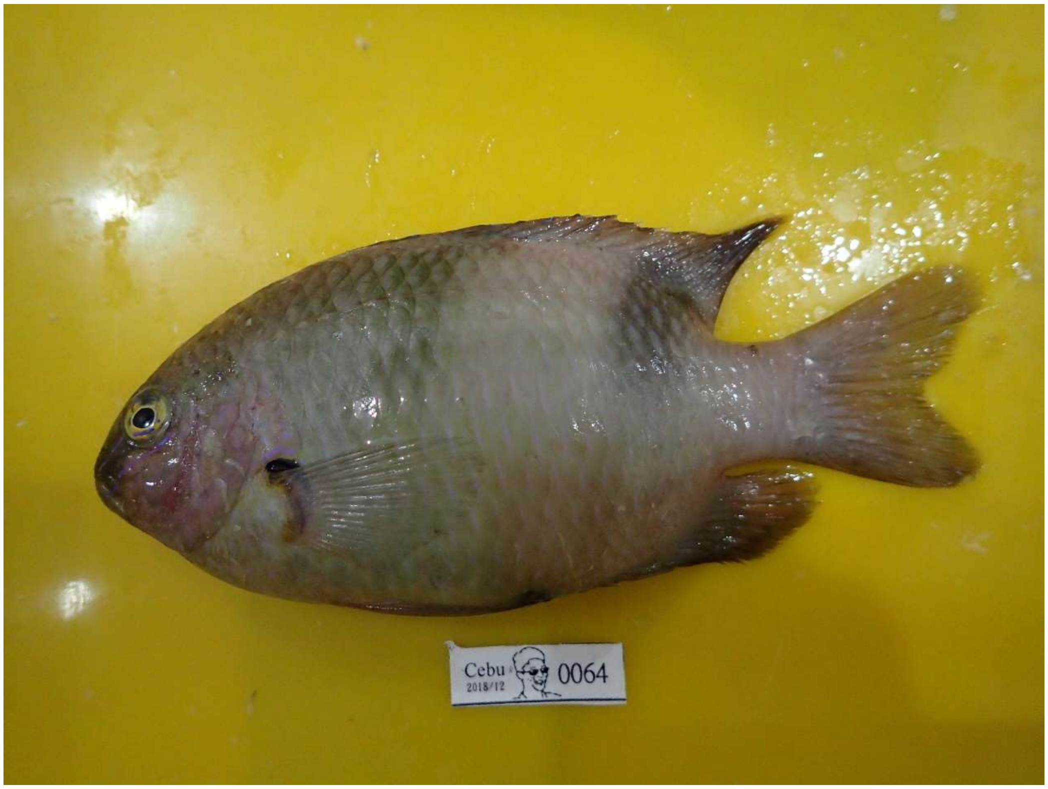

DOS 06894, *Dischistodus prosopotaenia*, OR114071. (specimen not preserved)

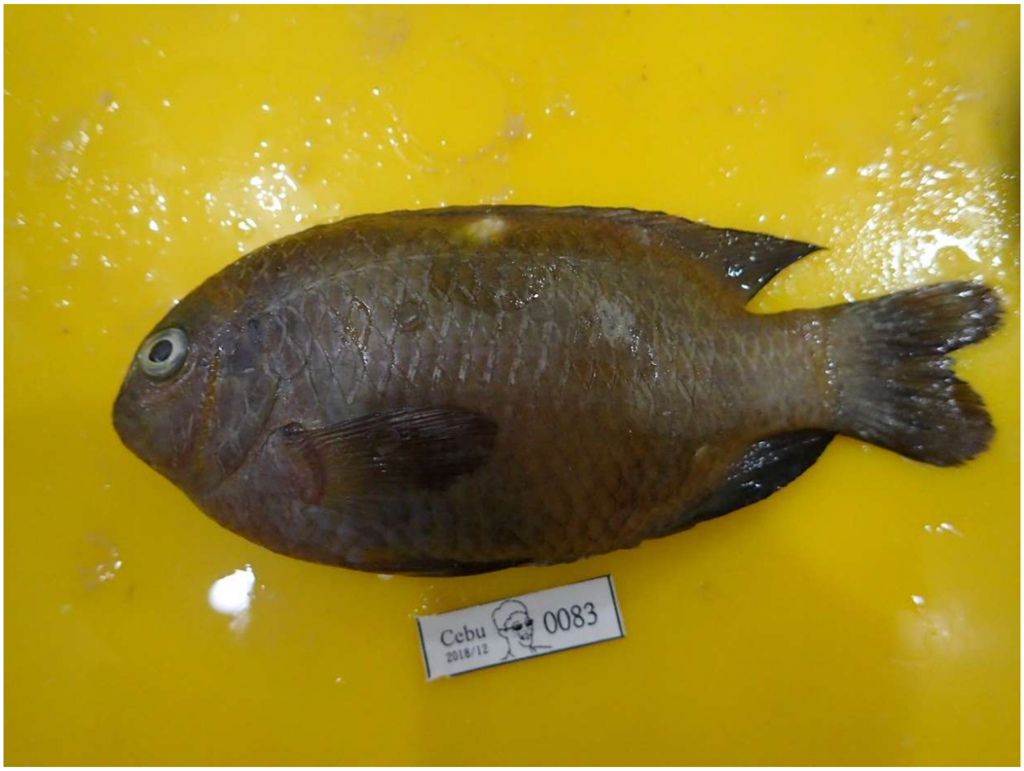

DOS 06903, *Dischistodus pseudochrysopoecilus*, OR114080. (specimen not preserved)

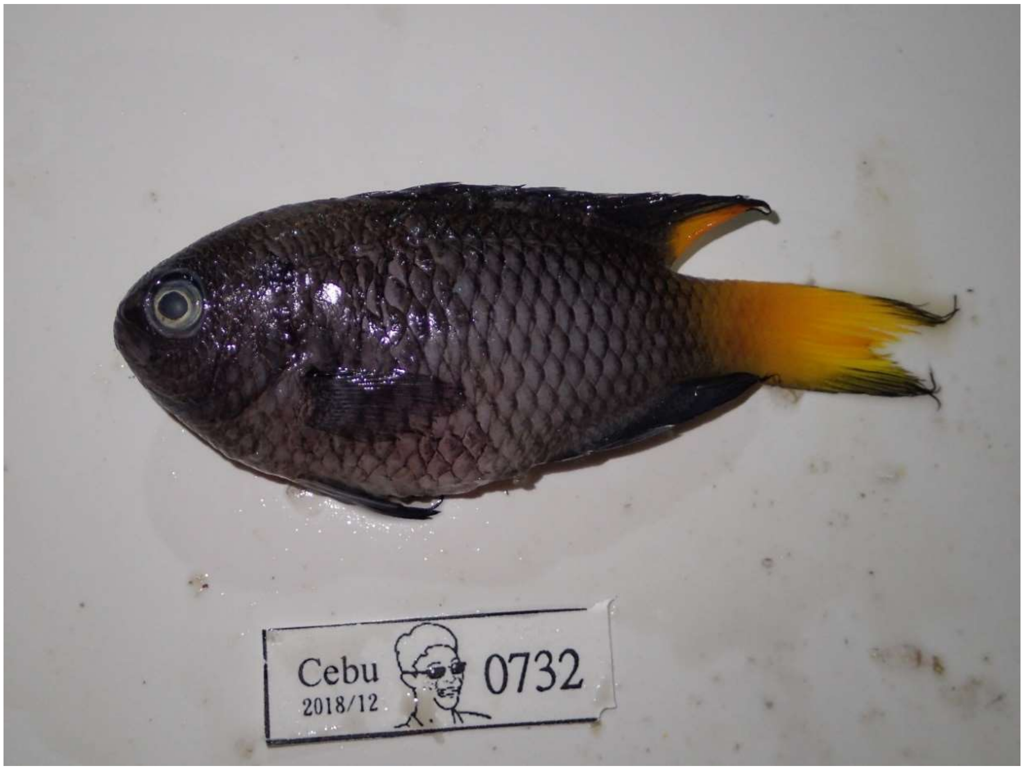

DOS 06905-1, *Neopomacentrus violascens*, OR114082.

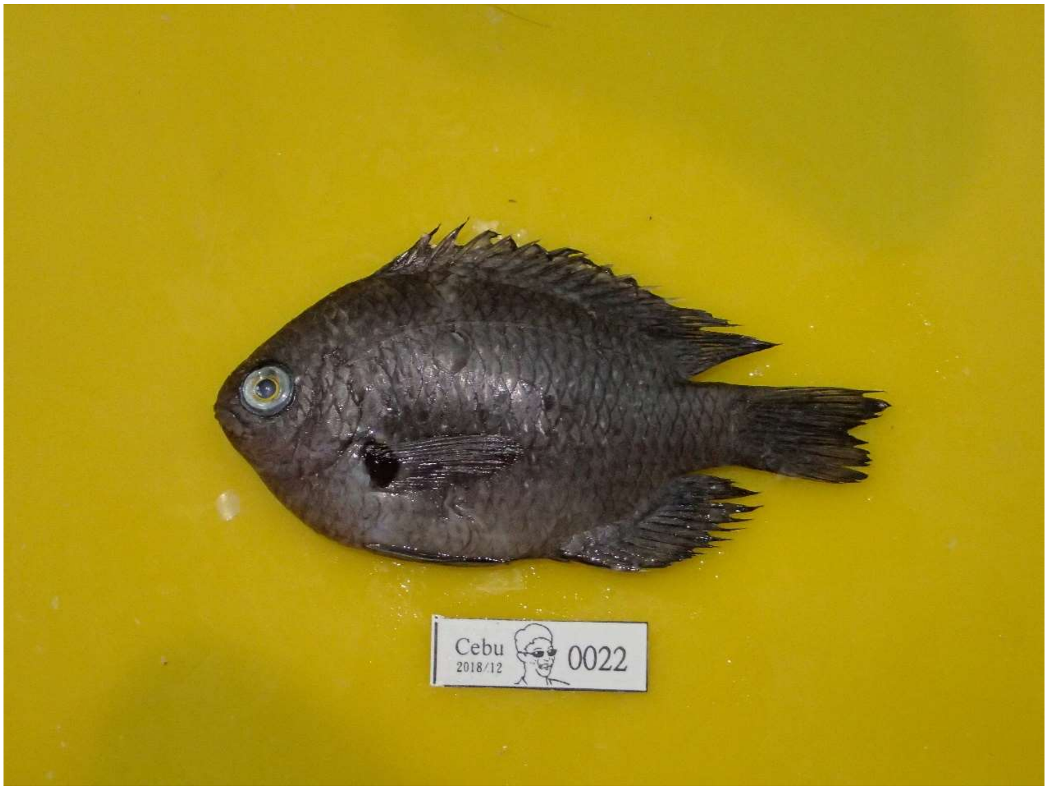

DOS 06902, *Pomacentrus brachialis*, OR114079. (specimen not preserved)

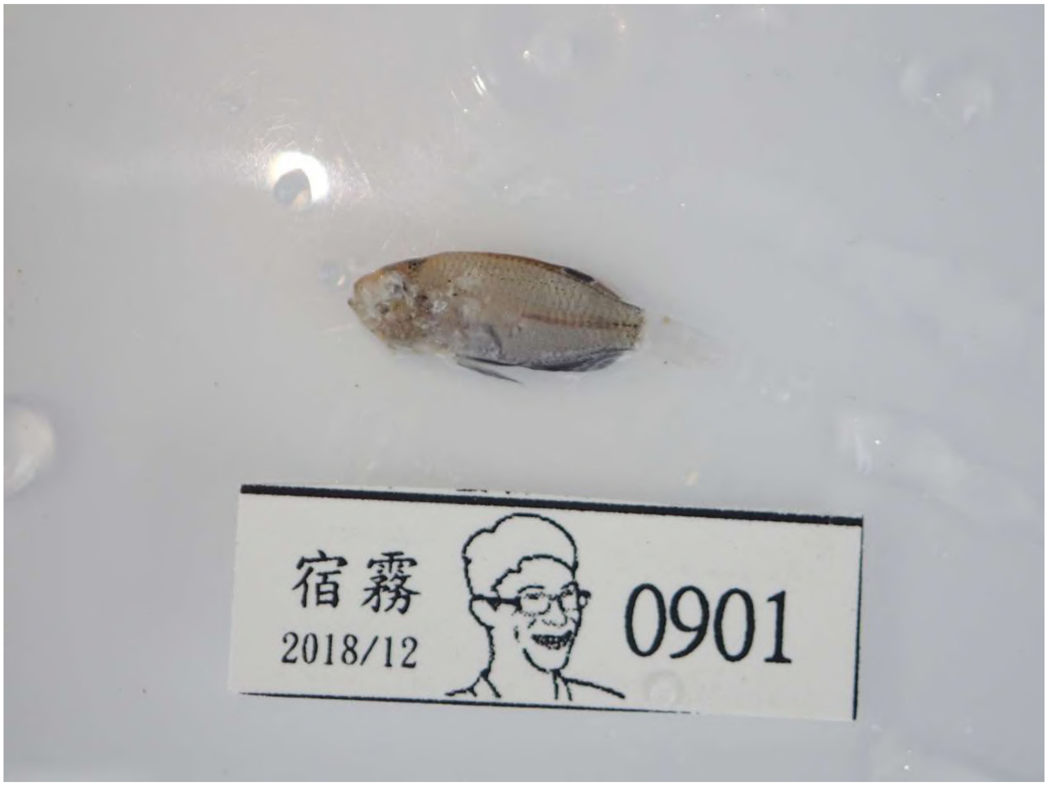

DOS 06891-1, *Pomacentrus chrysurus*, OR114068.

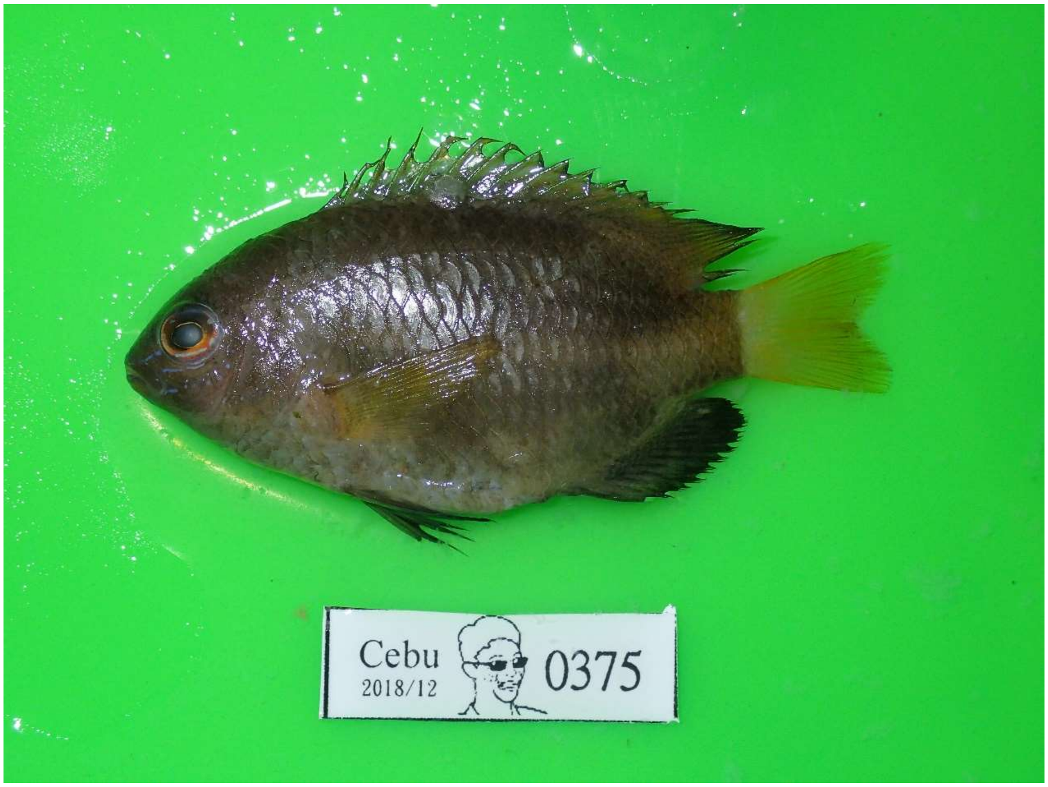

DOS 06895, *Pomacentrus chrysurus*, OR114072.

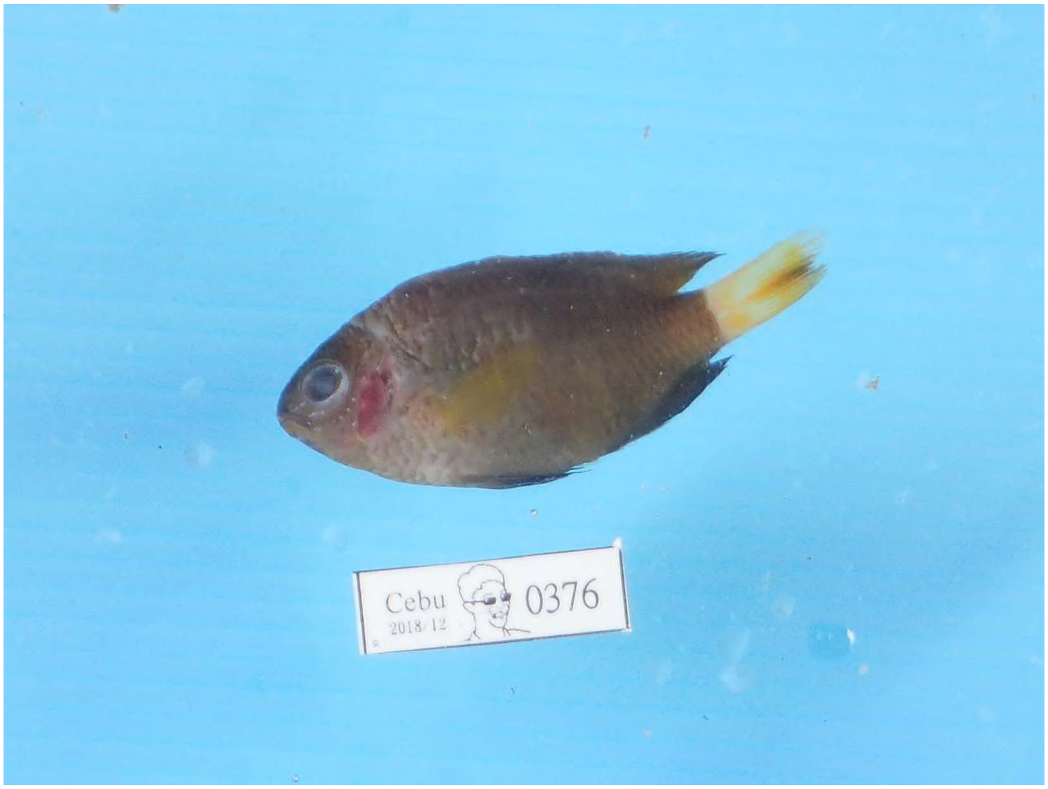

DOS 06904-1, *Pomacentrus chrysurus*, OR114081.

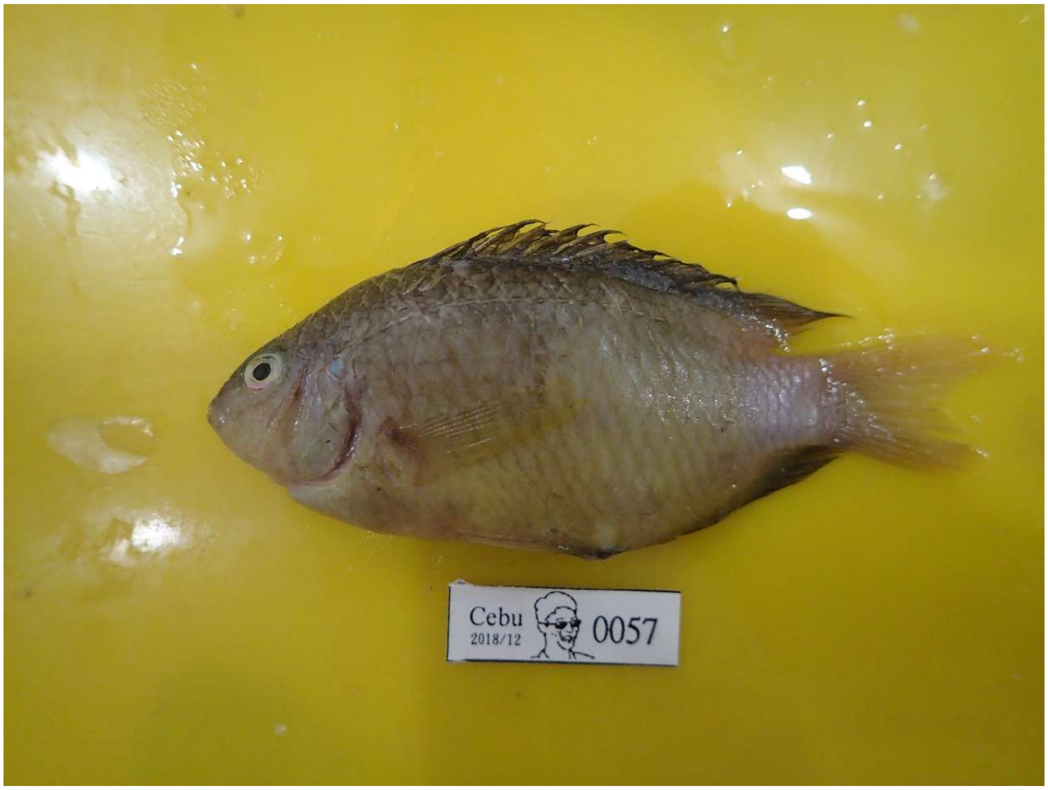

DOS 06876-1, *Pomacentrus grammorhynchus*, OR114053.

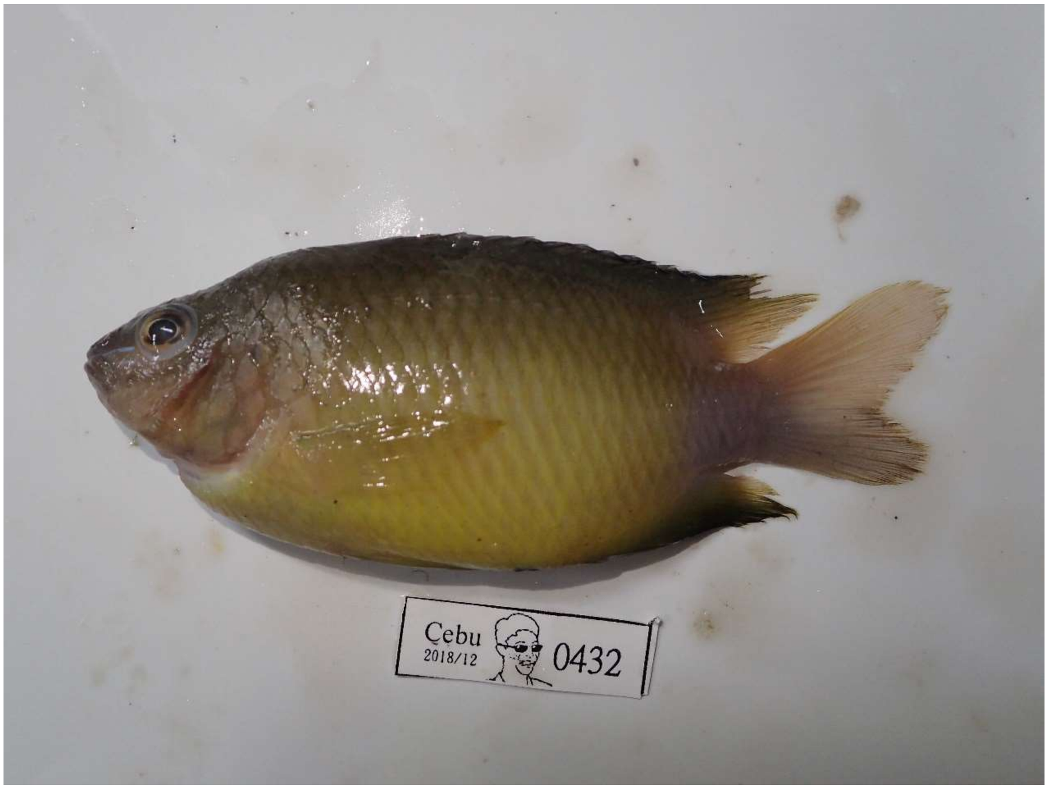

DOS 06893, *Pomacentrus grammorhynchus*, OR114070.

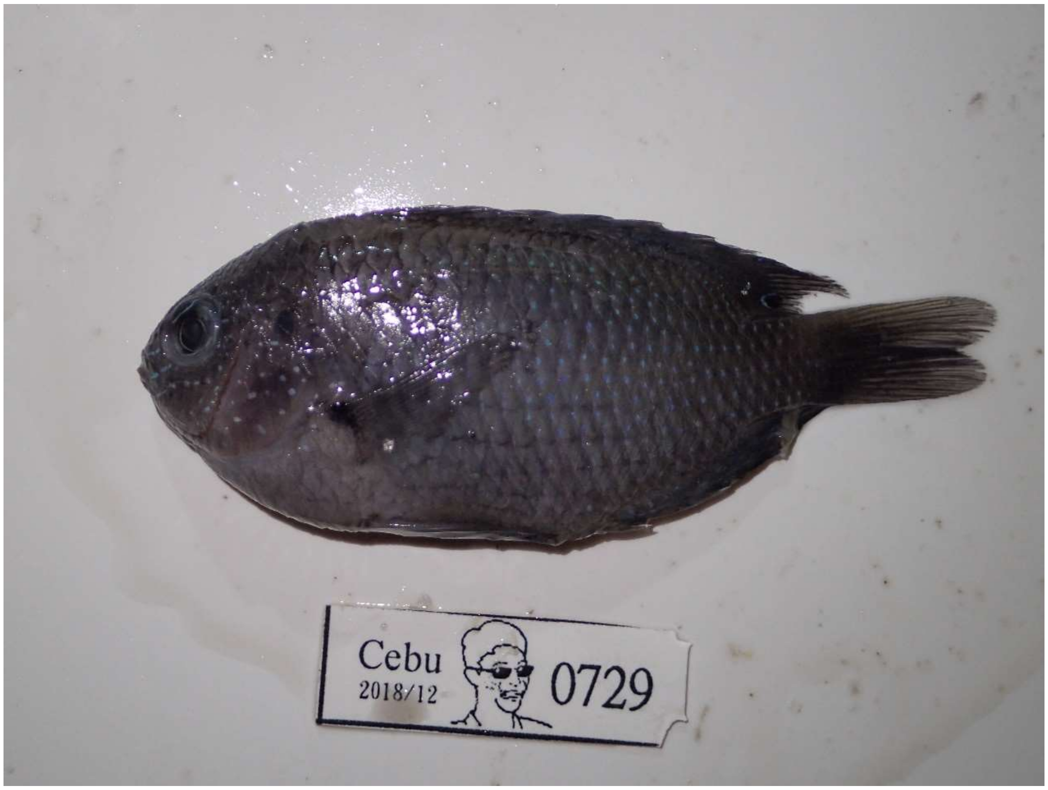

DOS 06906-5, *Pomacentrus littoralis*, OR114083.

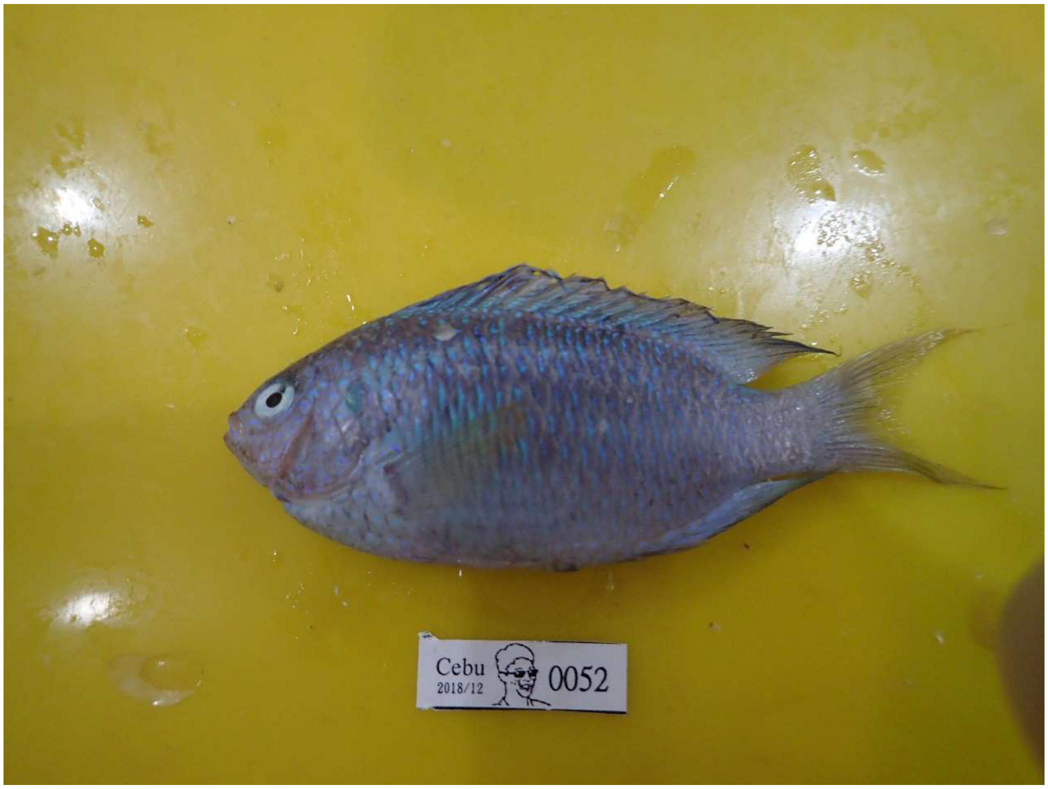

DOS 06877-1, *Pomacentrus pavo*, OR114054.

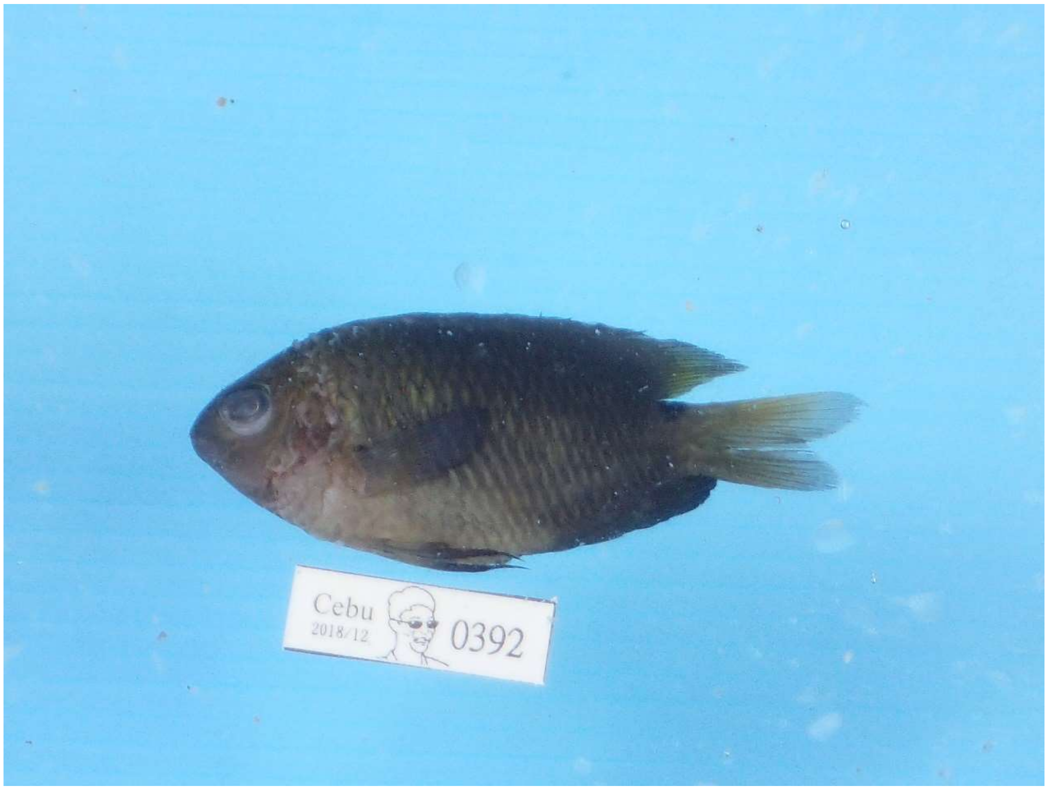

DOS 06886, *Pomacentrus tripunctatus*, OR114063.

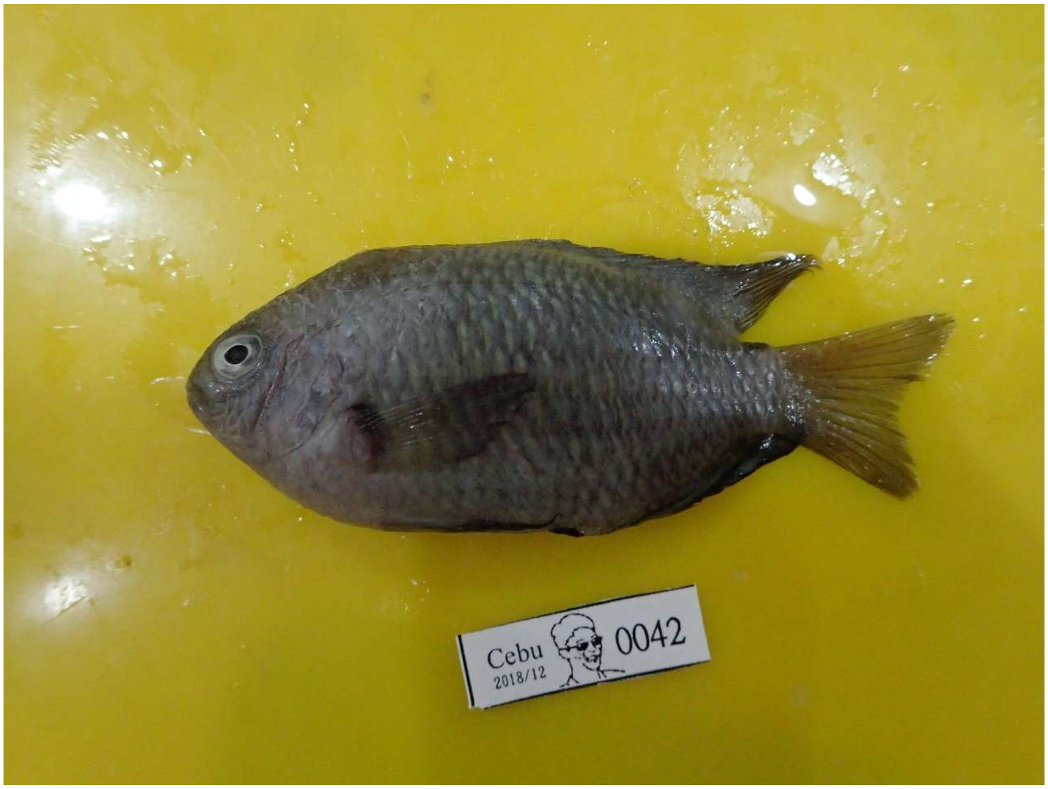

DOS 06900, *Pomacentrus tripunctatus*, OR114077.

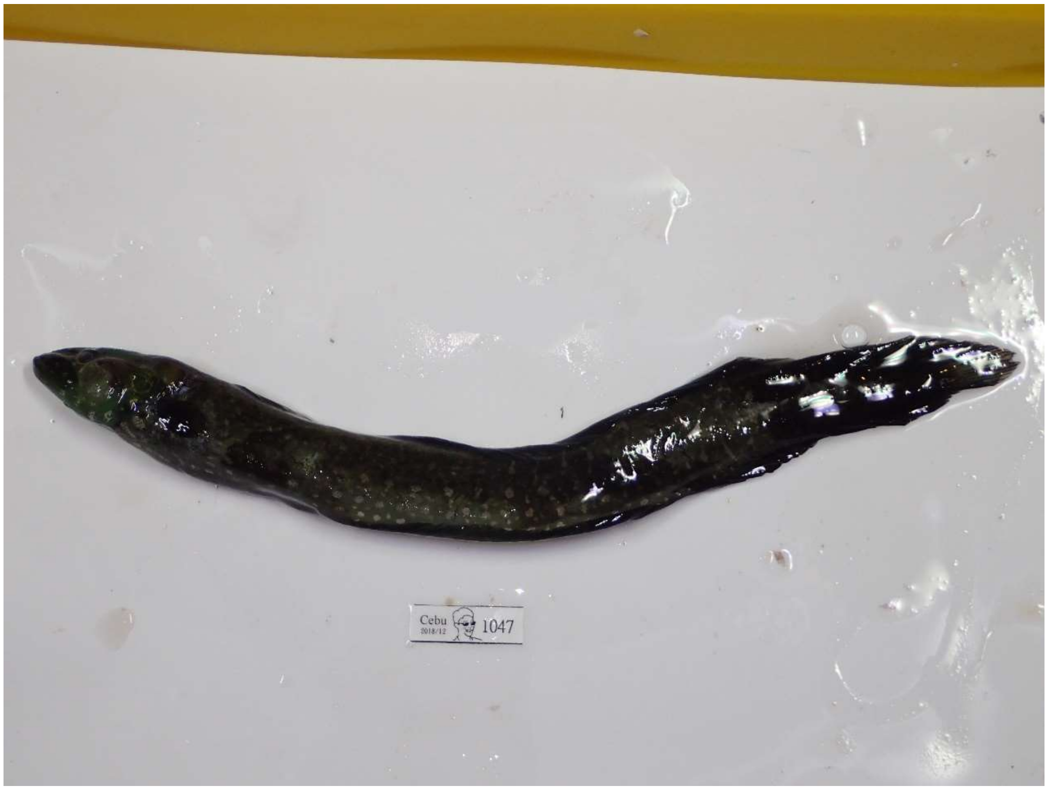

DOS 06910-1, *Congrogadus subducens*, OR114087.

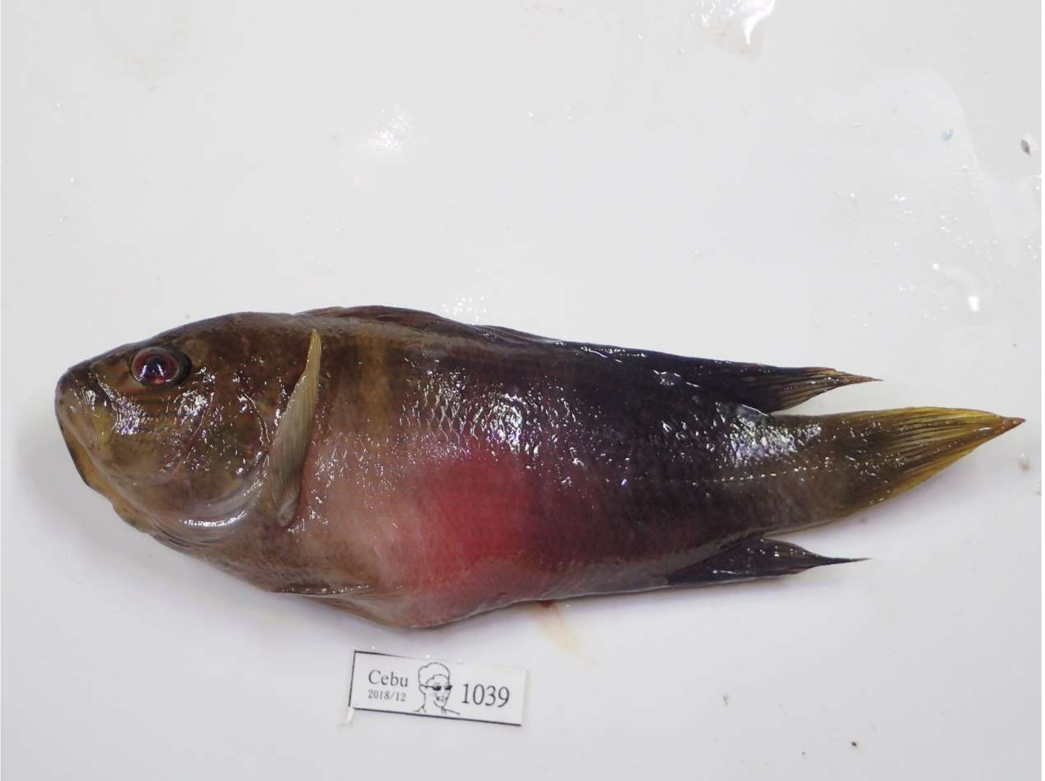

DOS 06911-1, *Labracinus* sp., OR114088. (specimen not preserved)

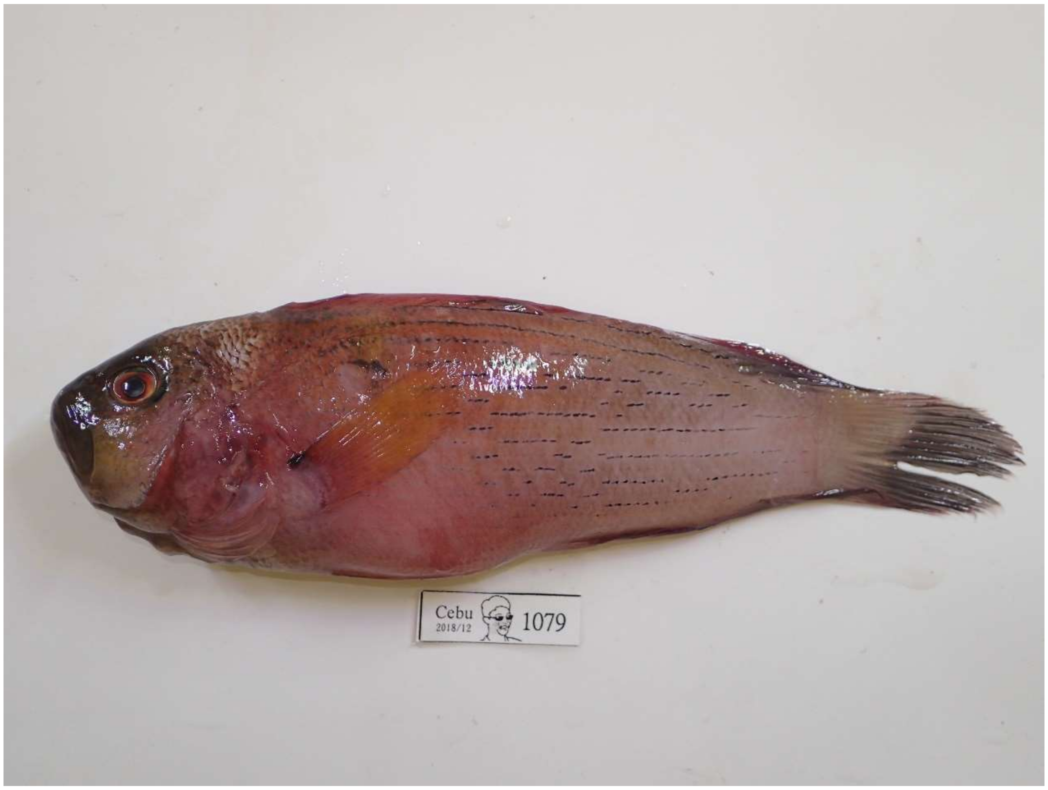

DOS 06912, *Labracinus* sp., OR114089.

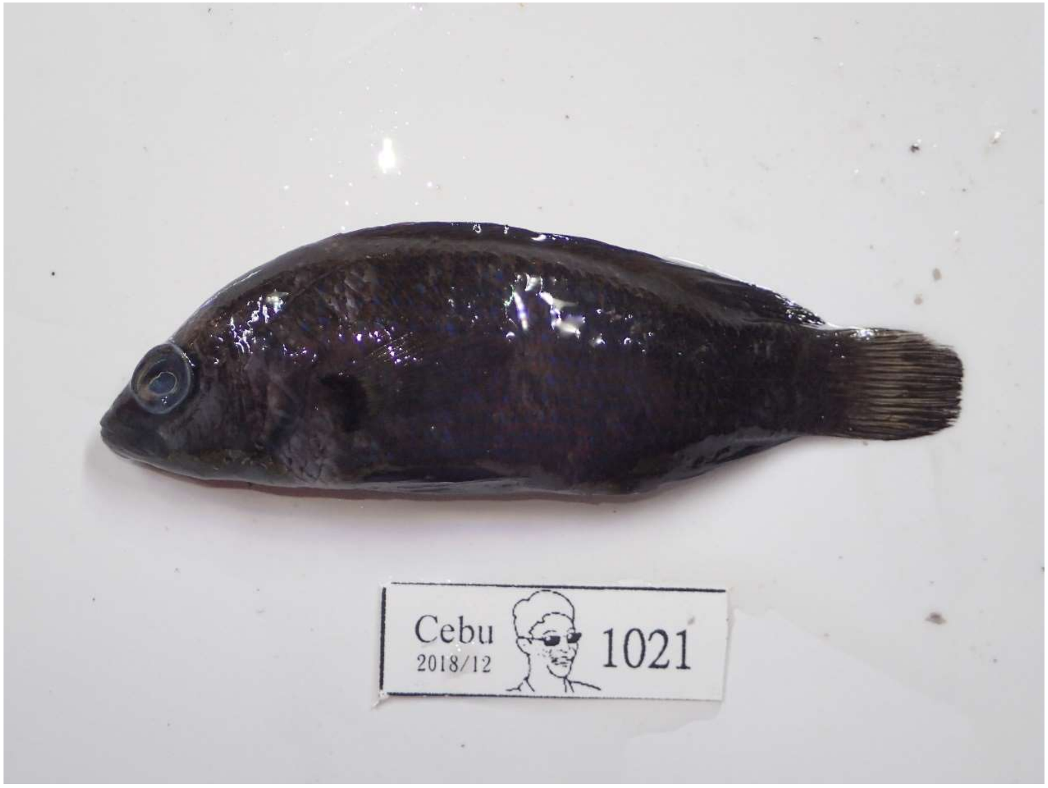

DOS 06954-1, *Pseudochromis fuscus*, OR114130.

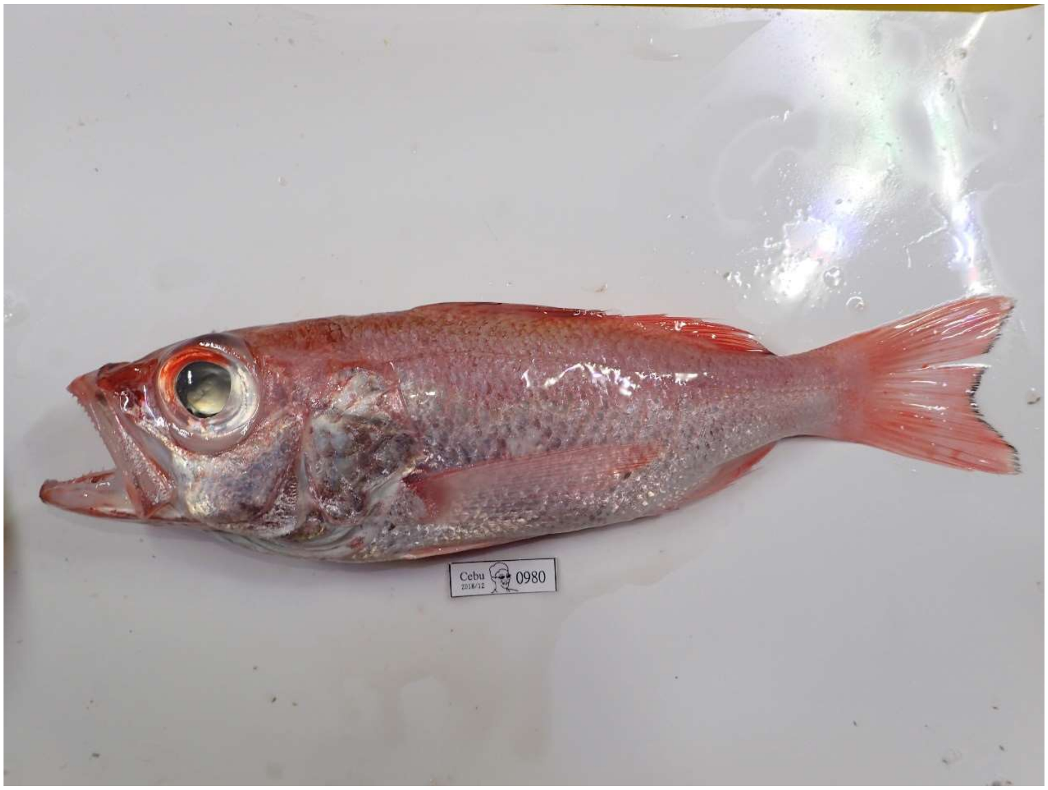

DOS 06577-2, *Doederleinia berycoides*, OR113779. (specimen not preserved)

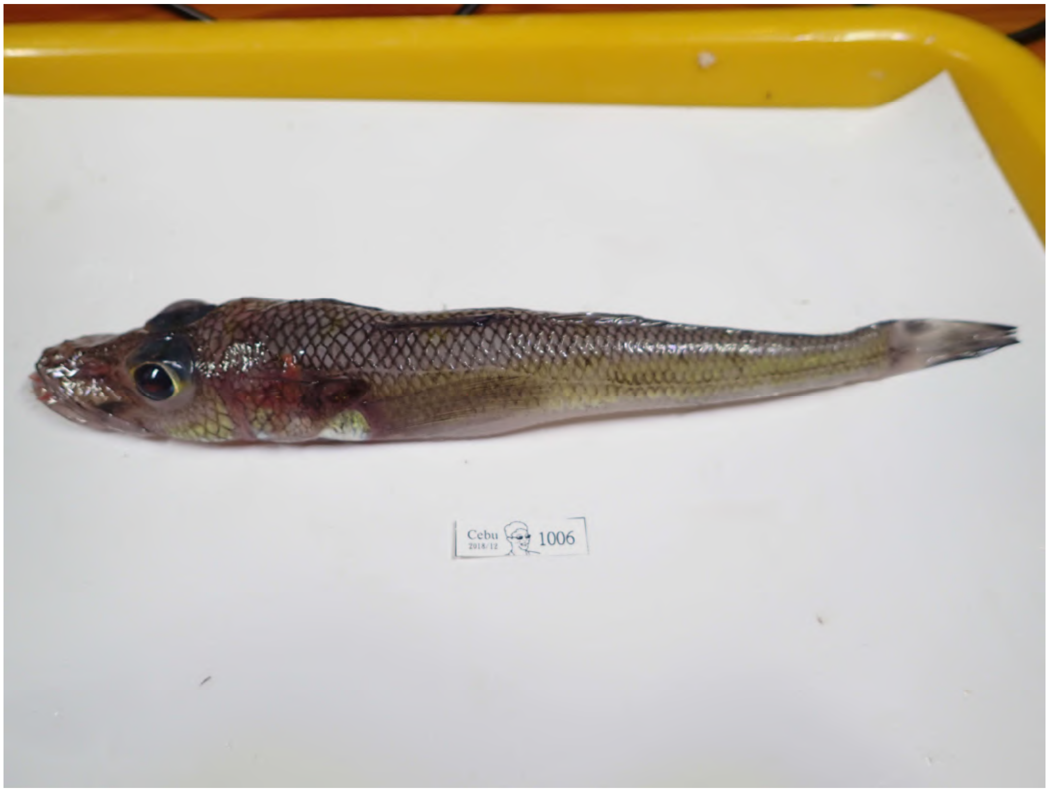

DOS 06708-1, *Bembrops caudimacula*, OR113896.

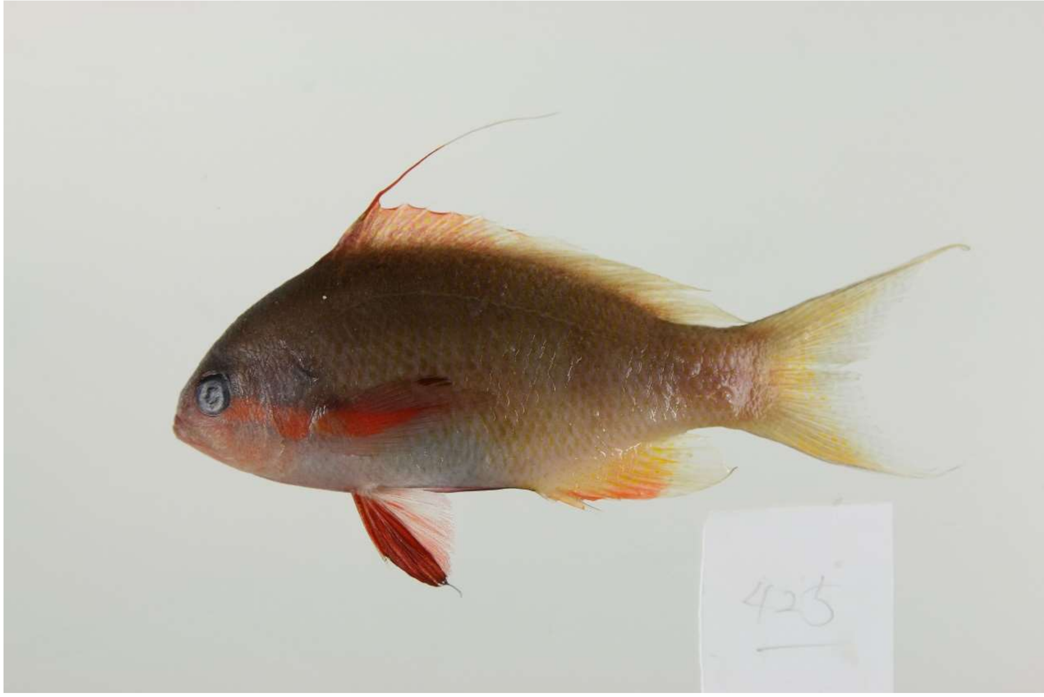

DOS 08663, *Pseudanthias huchtii*, OR114239.

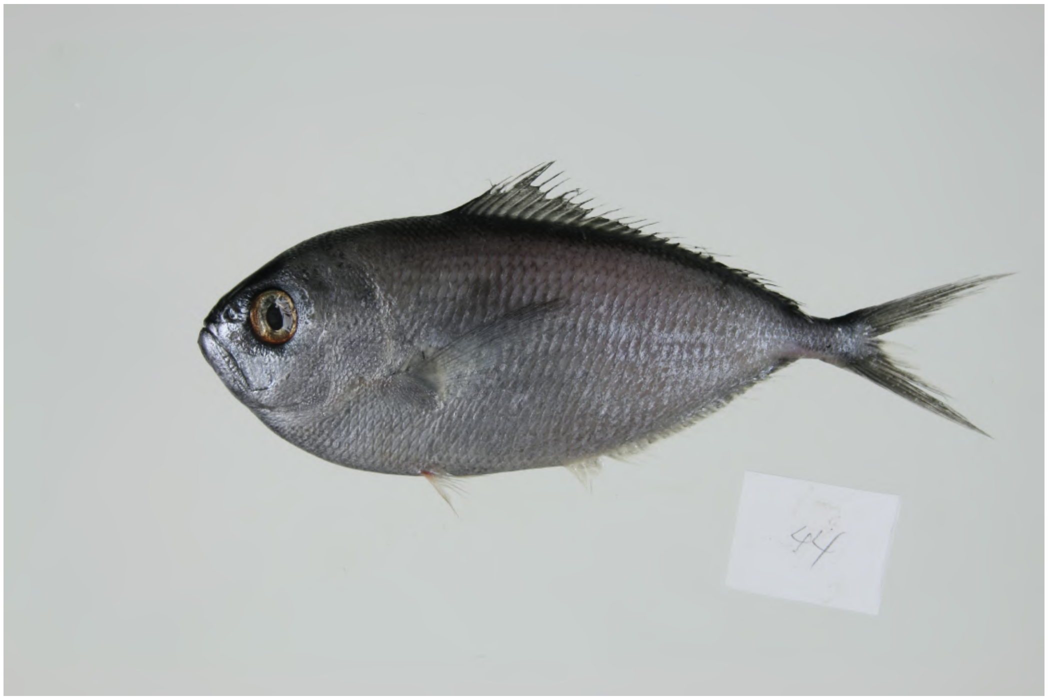

DOS 08653-1, *Brama pauciradiata*, OR114228.

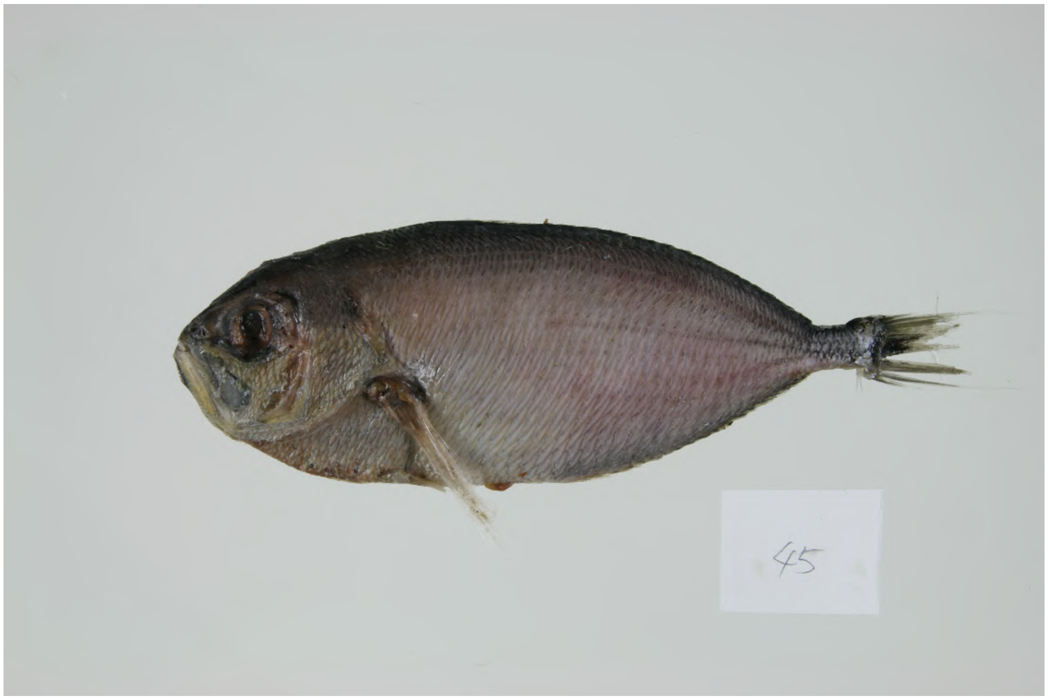

DOS 08653-2, *Brama pauciradiata*, OR114229.

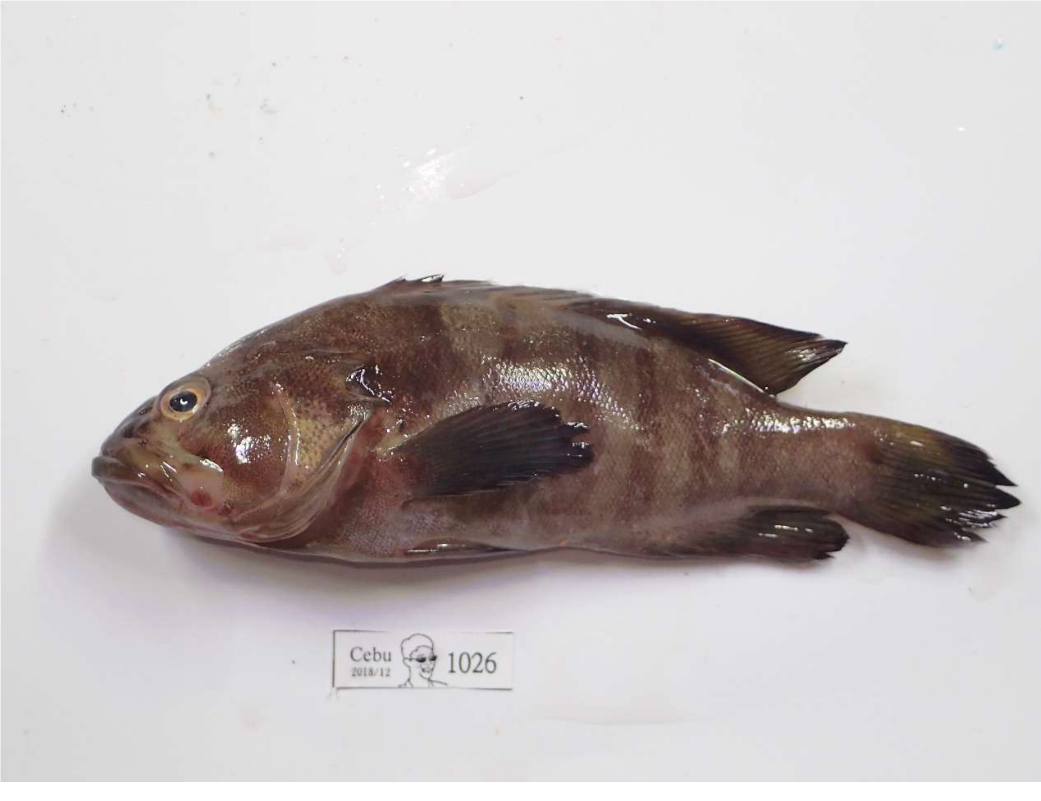

DOS 06949, *Cephalopholis boenak*, OR114125.

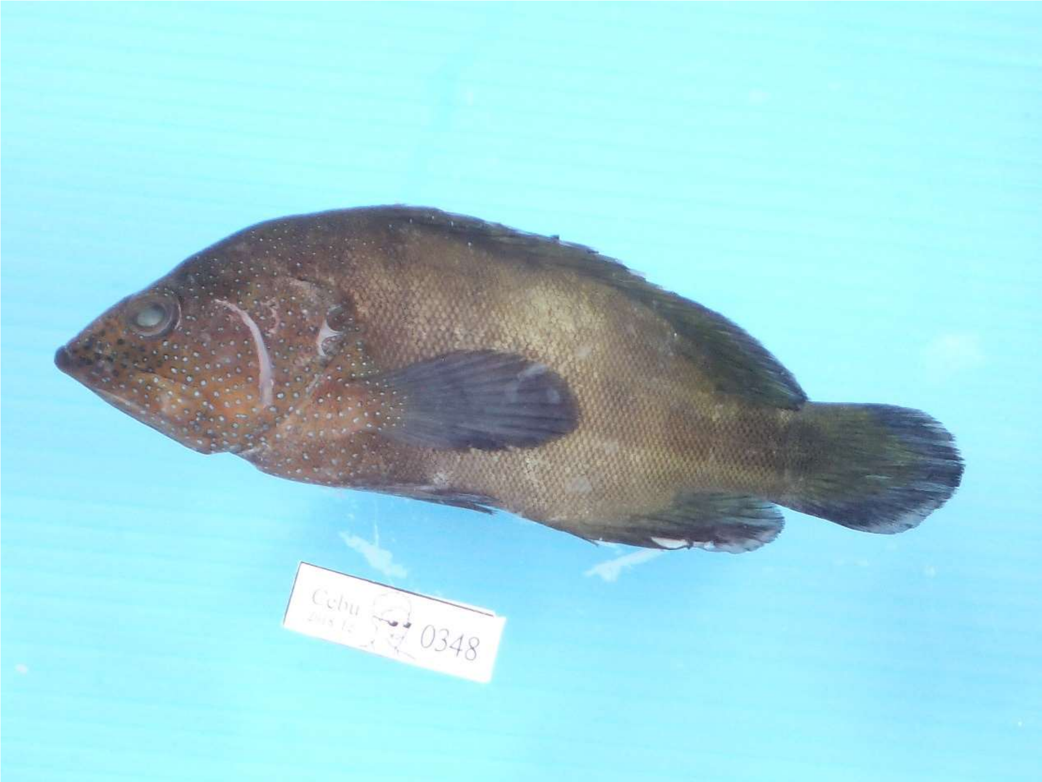

DOS 06939-1, *Cephalopholis microprion*, OR114115.

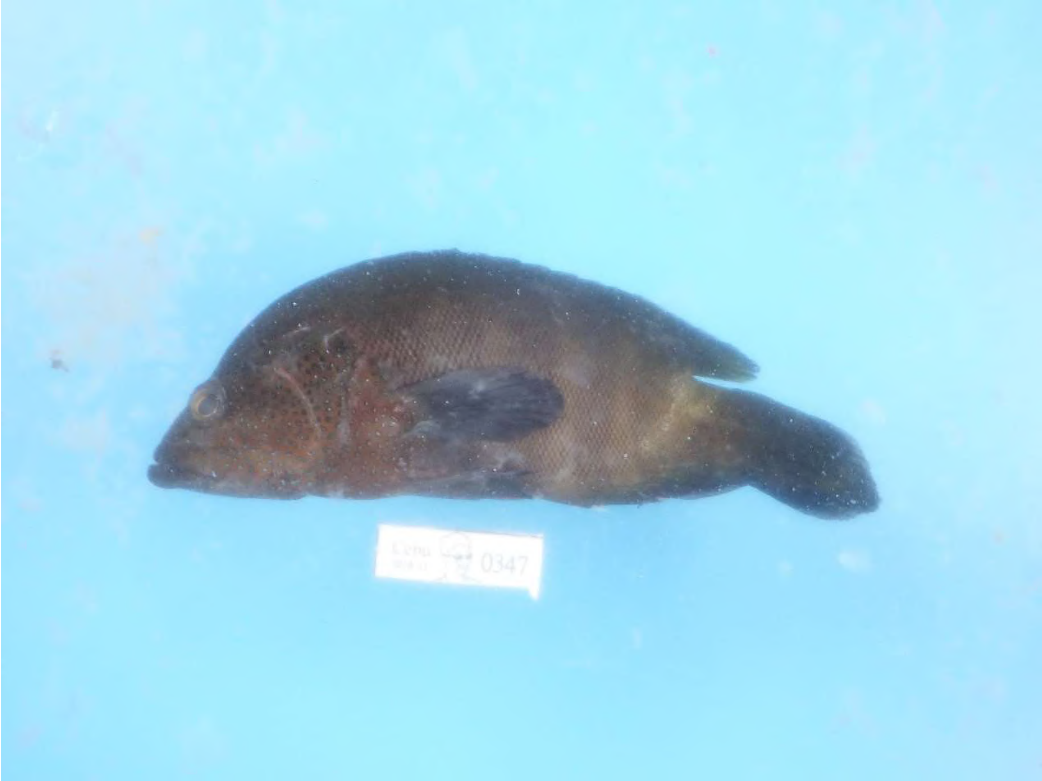

DOS 06940-1, *Cephalopholis microprion*, OR114116.

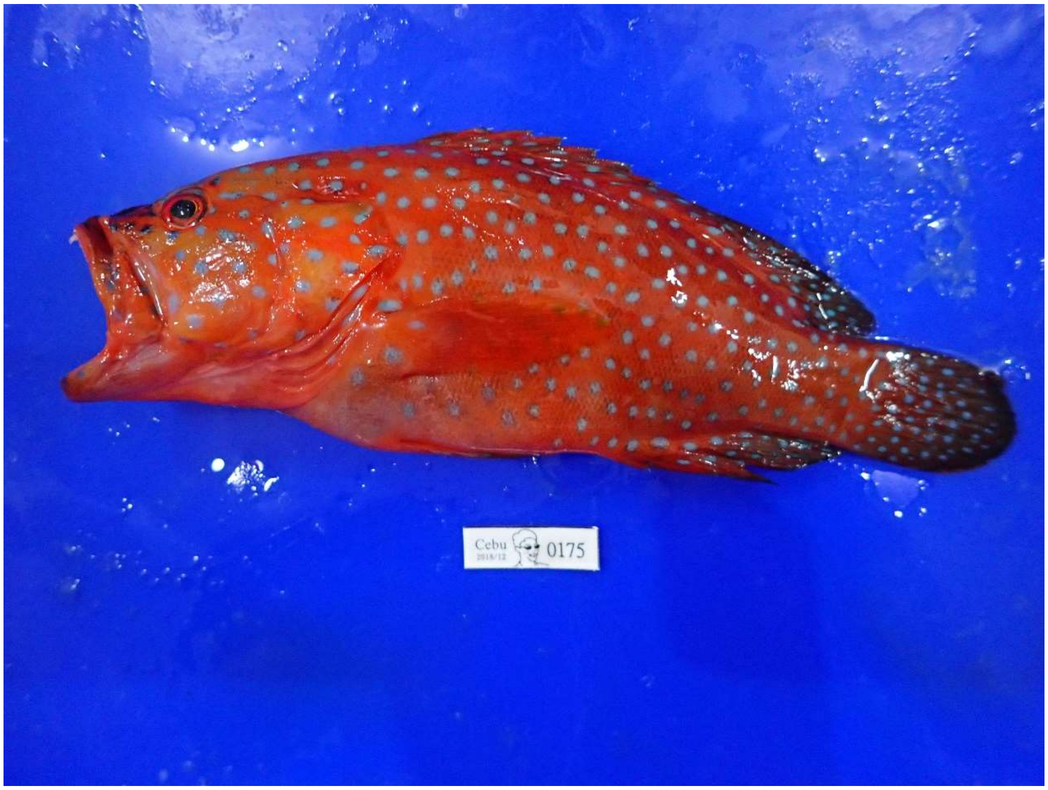

DOS 06941-1, *Cephalopholis miniata*, OR114117. (specimen not preserved)

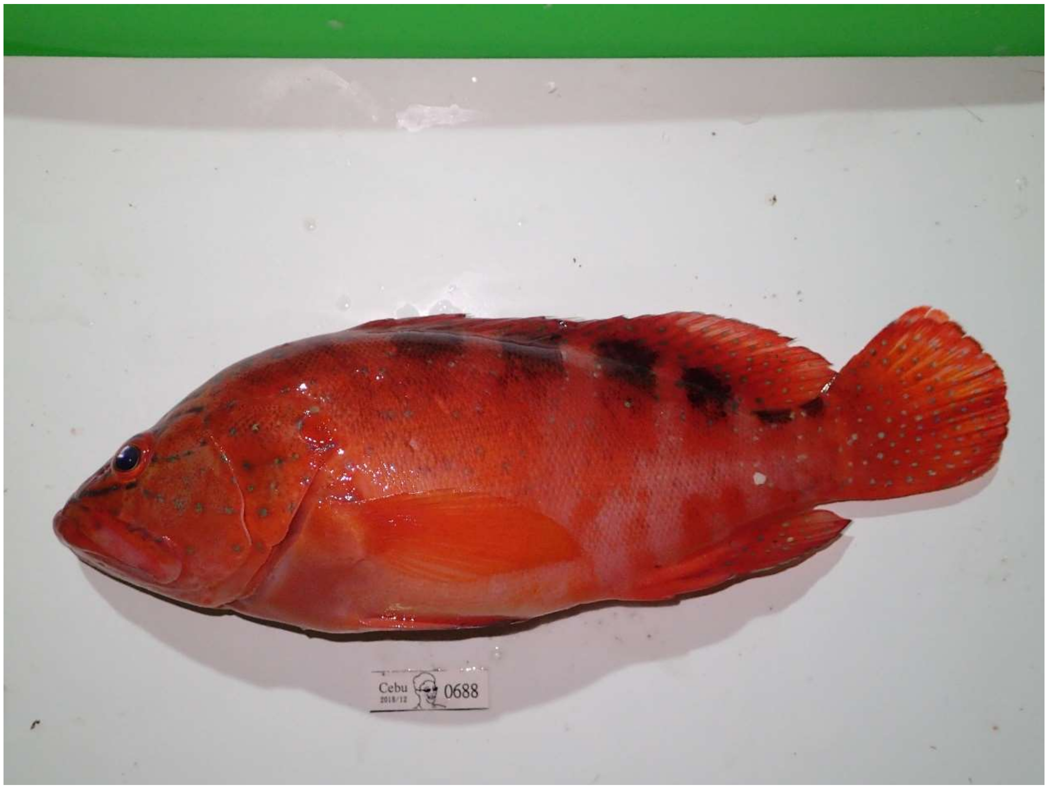

DOS 06943-1, *Cephalopholis sexmaculata*, OR114119.

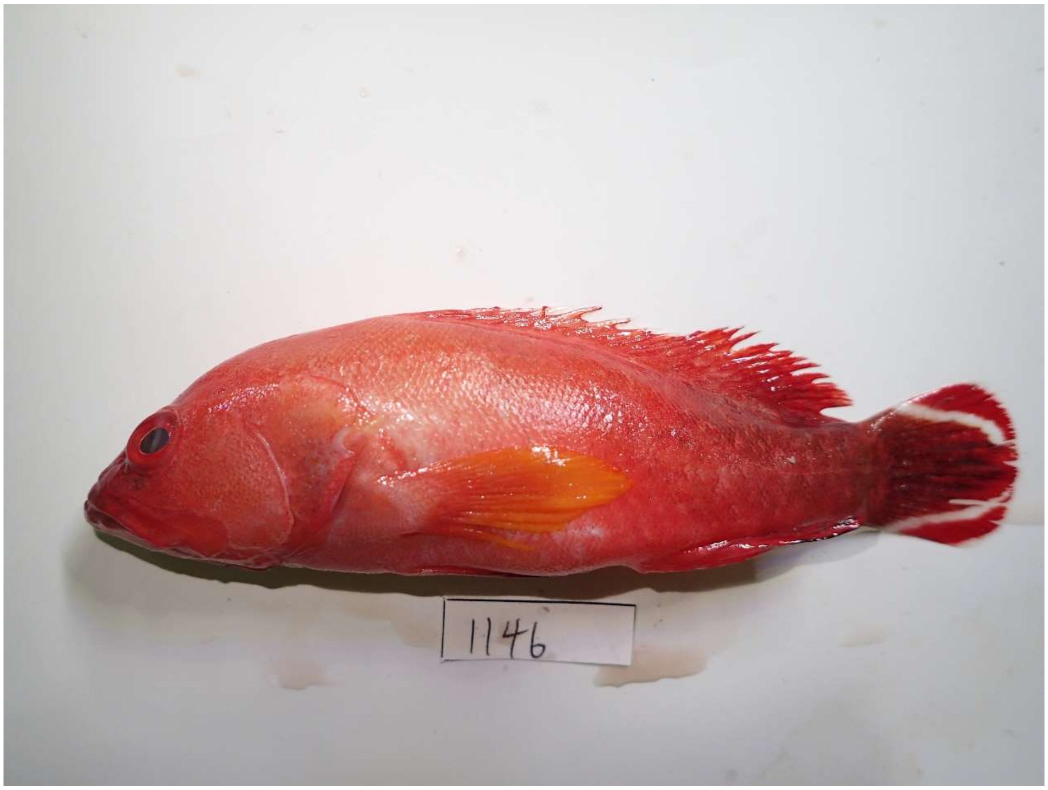

DOS 06944-1, *Cephalopholis urodeta*, OR114120.

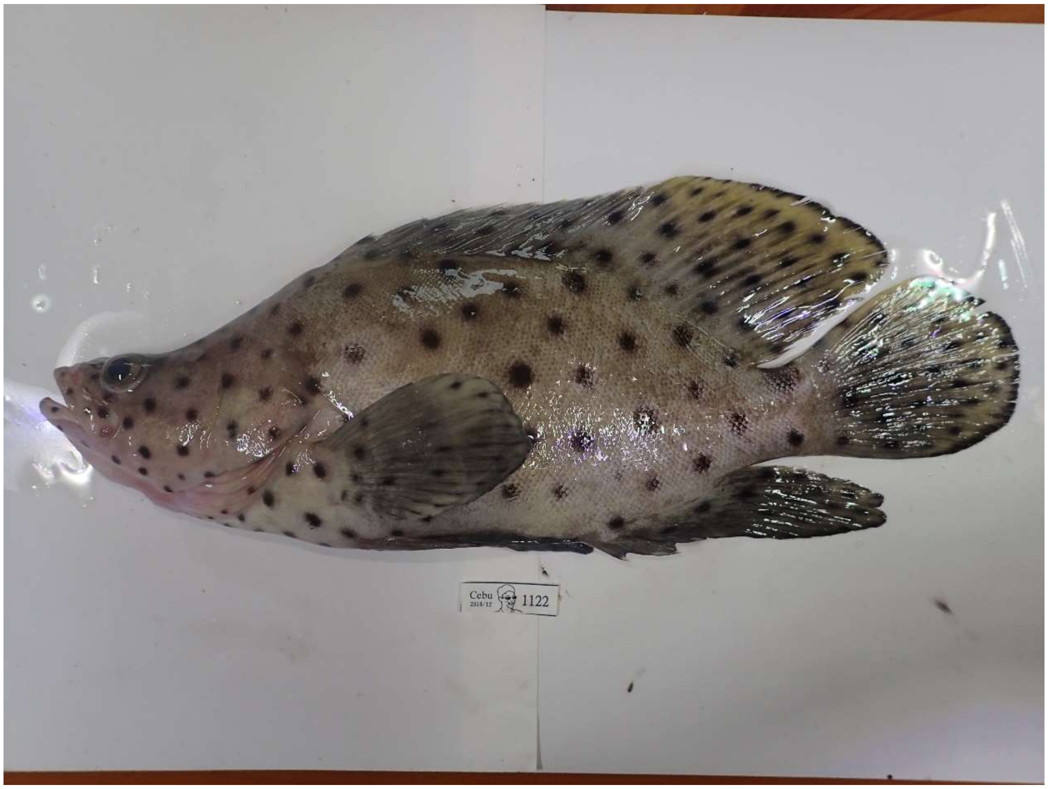

DOS 06945, *Chromileptes altivelis*, OR114121. (specimen not preserved)

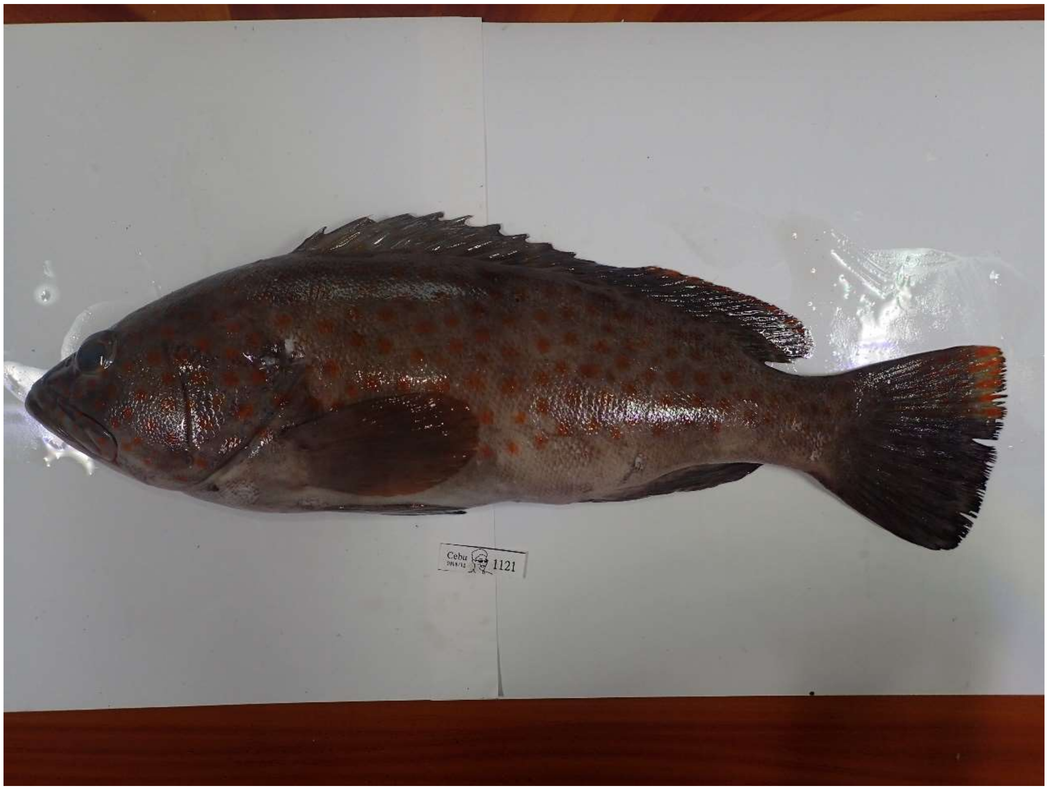

DOS 06938, *Epinephelus bleekeri*, OR114114. (specimen not preserved)

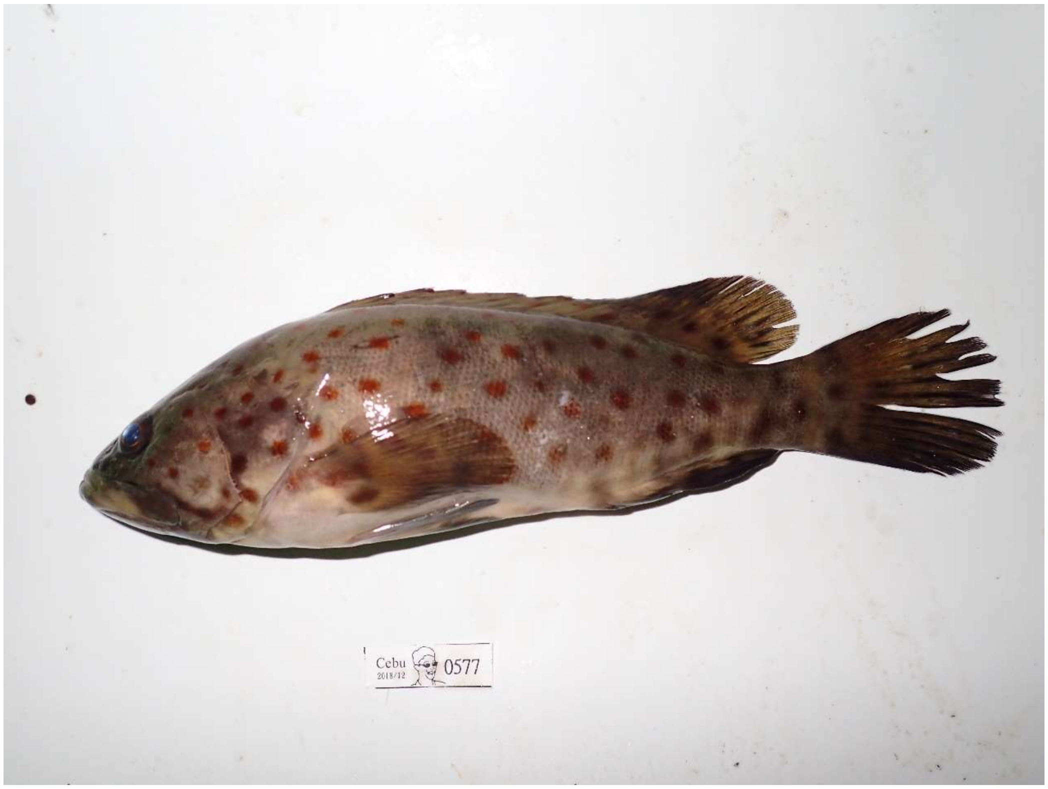

DOS 06946, *Epinephelus coioides*, OR114122.

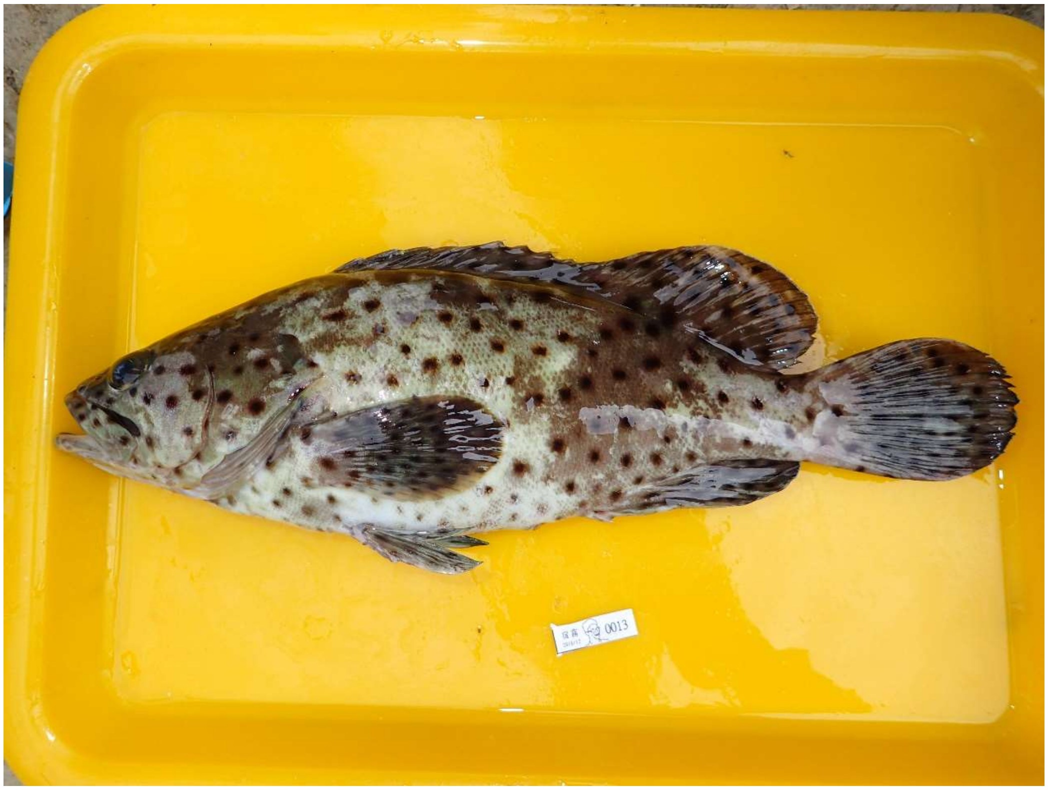

DOS 06948-1, *Epinephelus corallicola*, OR114124. (specimen not preserved)

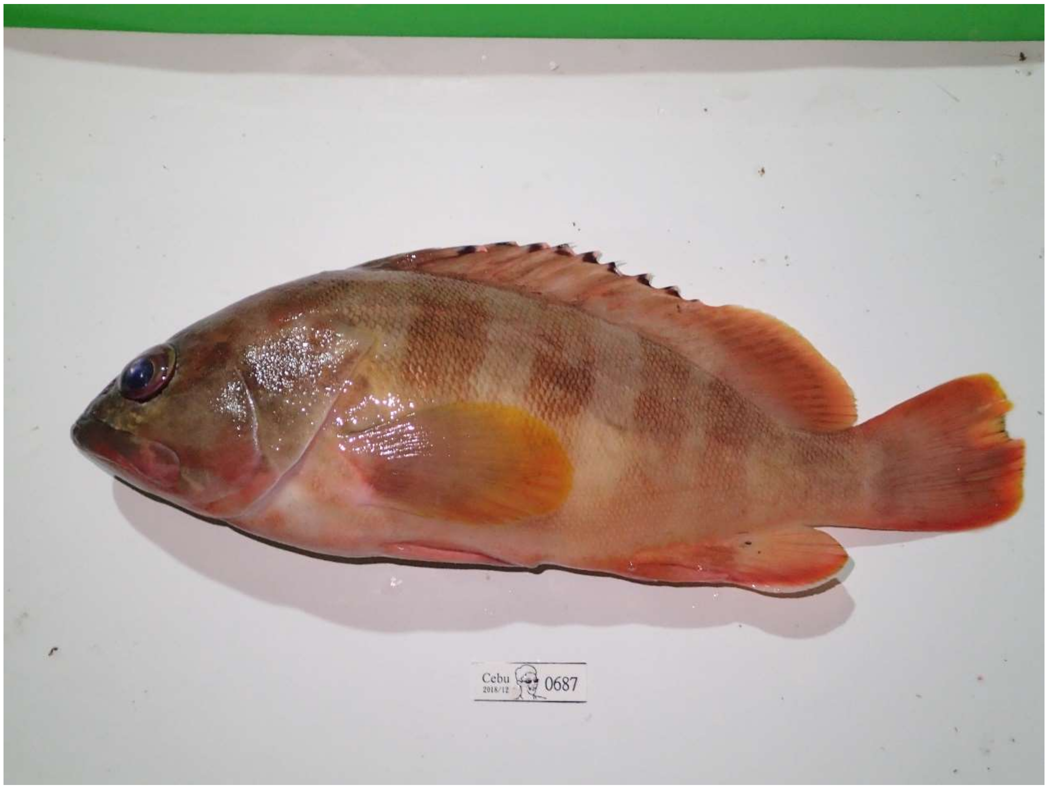

DOS 06950, *Epinephelus fasciatus*, OR114126.

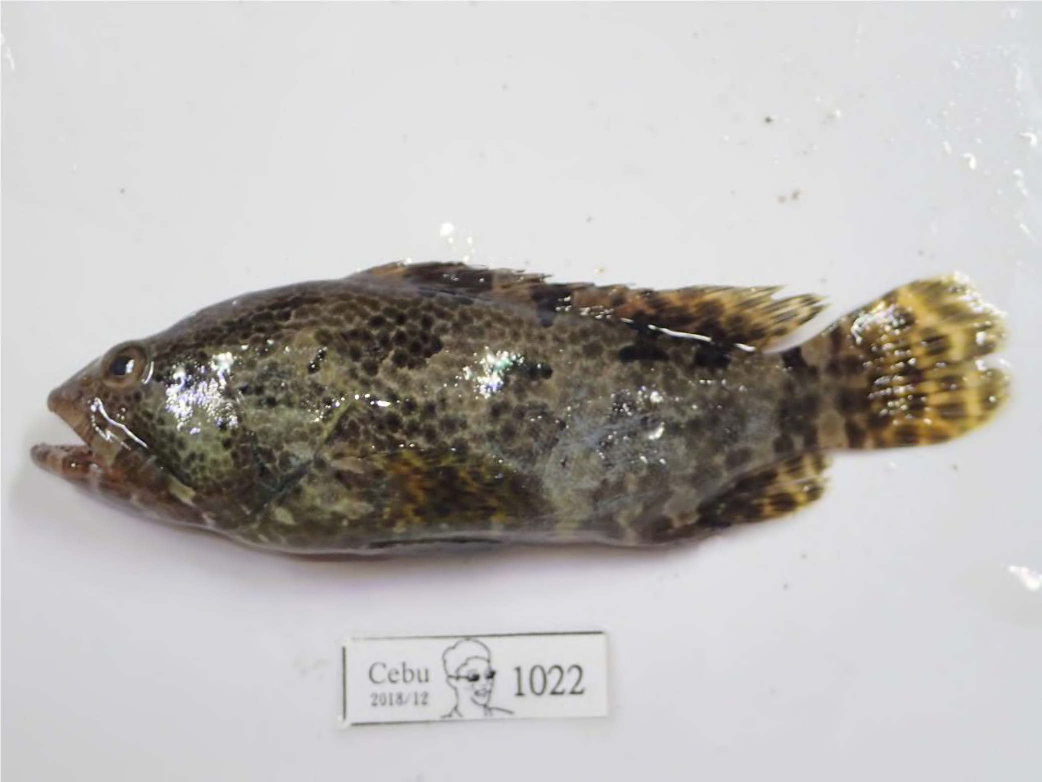

DOS 06951, *Epinephelus fuscoguttatus*, OR114127.

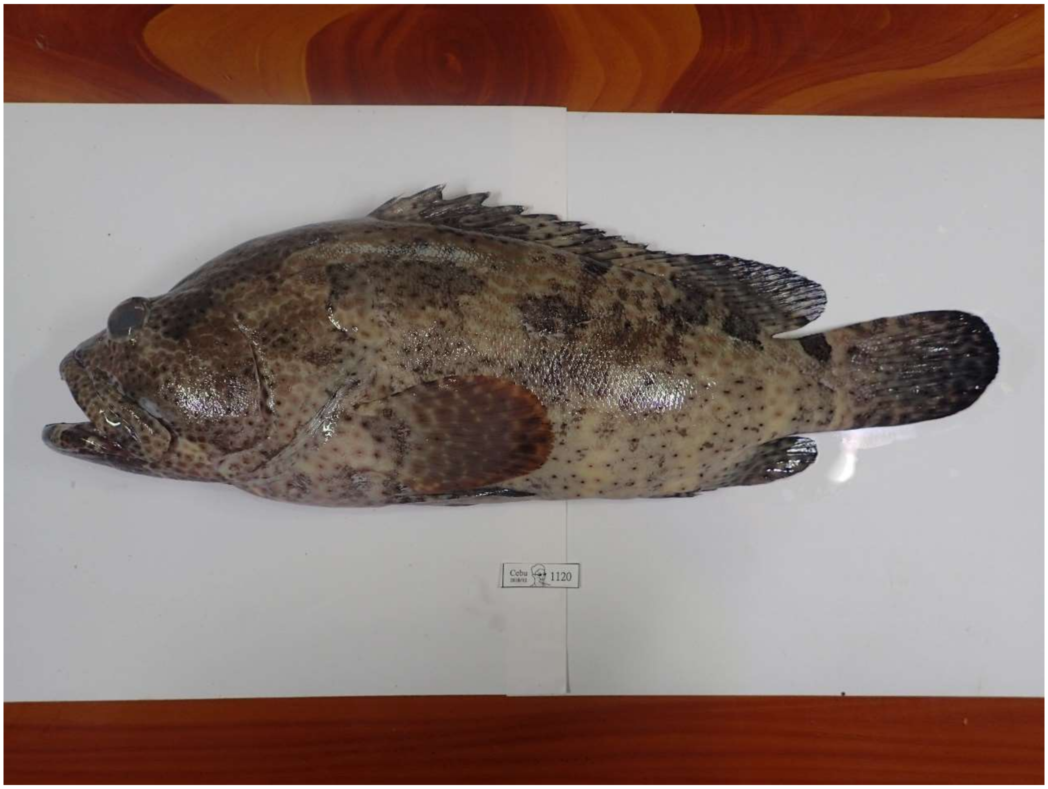

DOS 06953, *Epinephelus fuscoguttatus*, OR114129. (specimen not preserved)

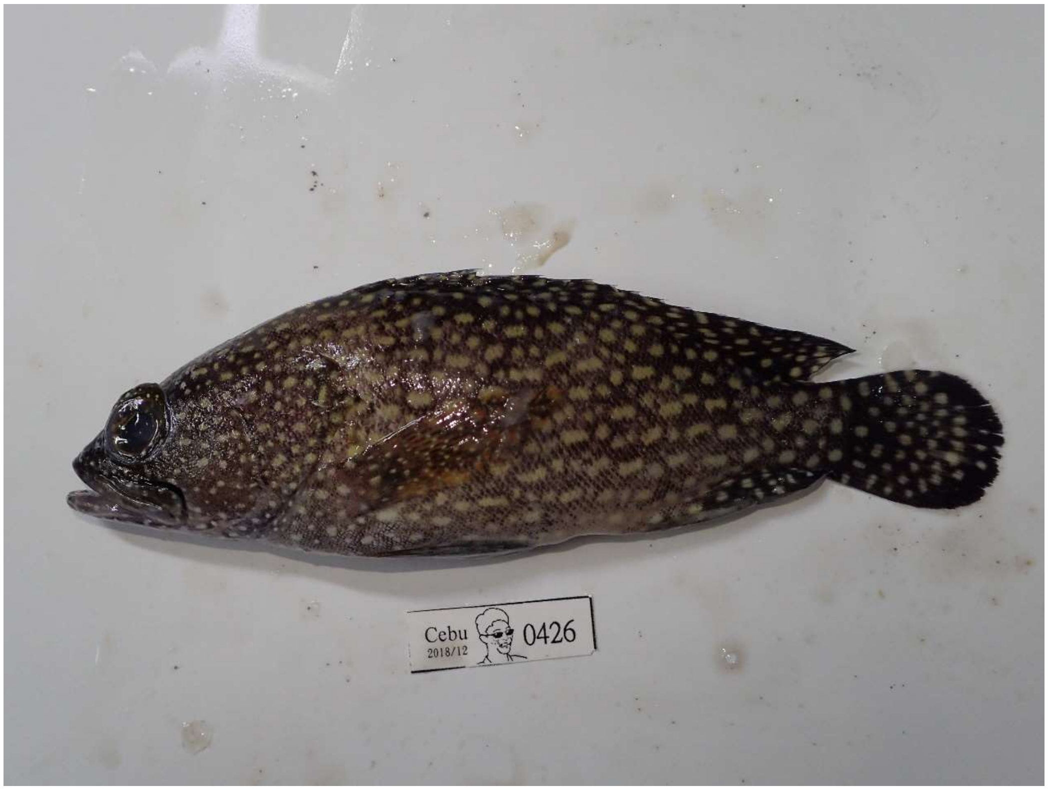

DOS 06942, *Epinephelus ongus*, OR114118.

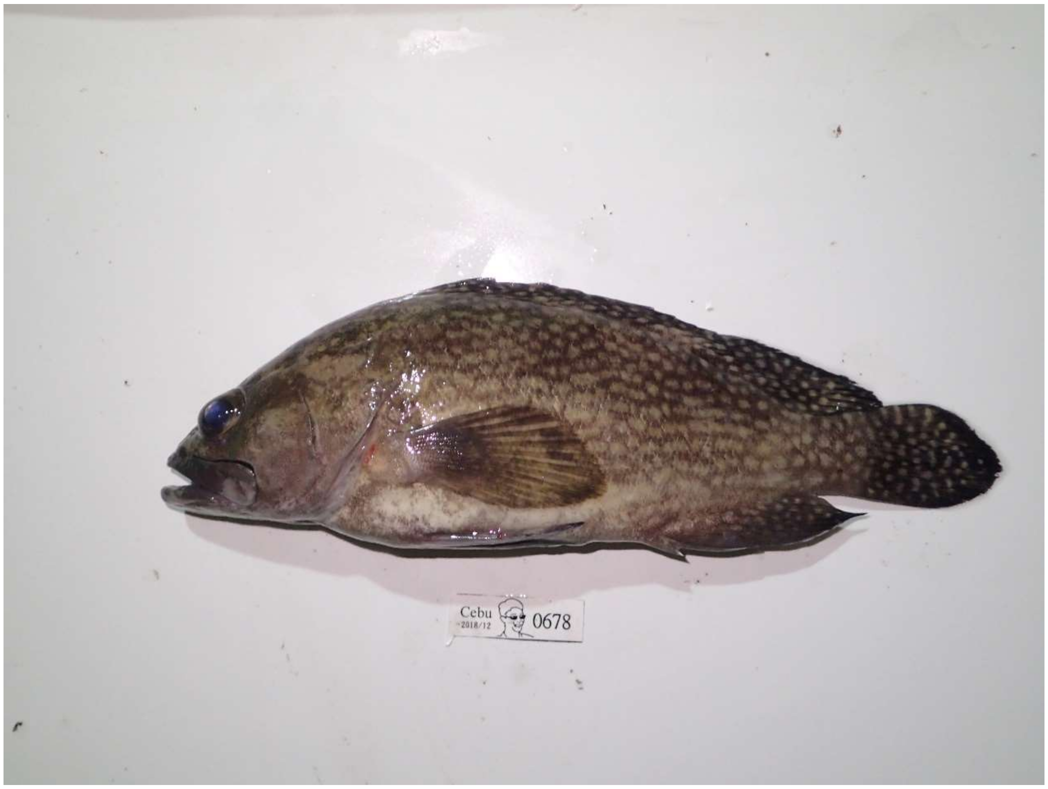

DOS 06947-1, *Epinephelus ongus*, OR114123.

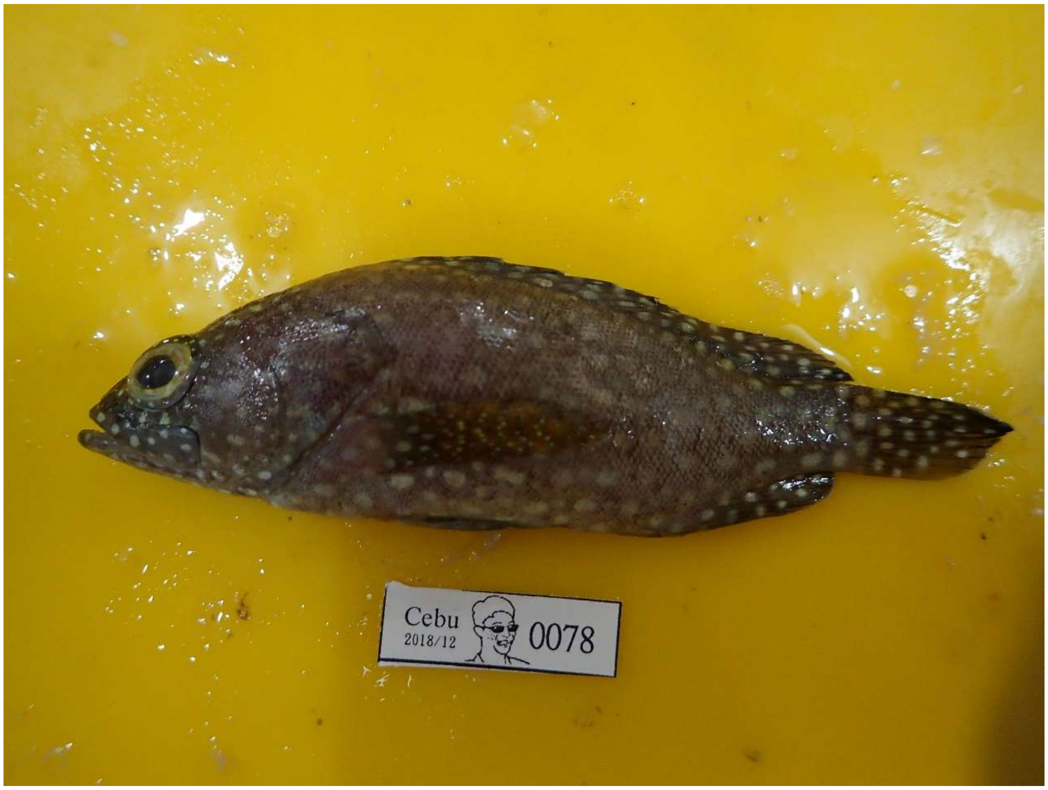

DOS 06955, *Epinephelus ongus*, OR114131. (specimen not preserved)

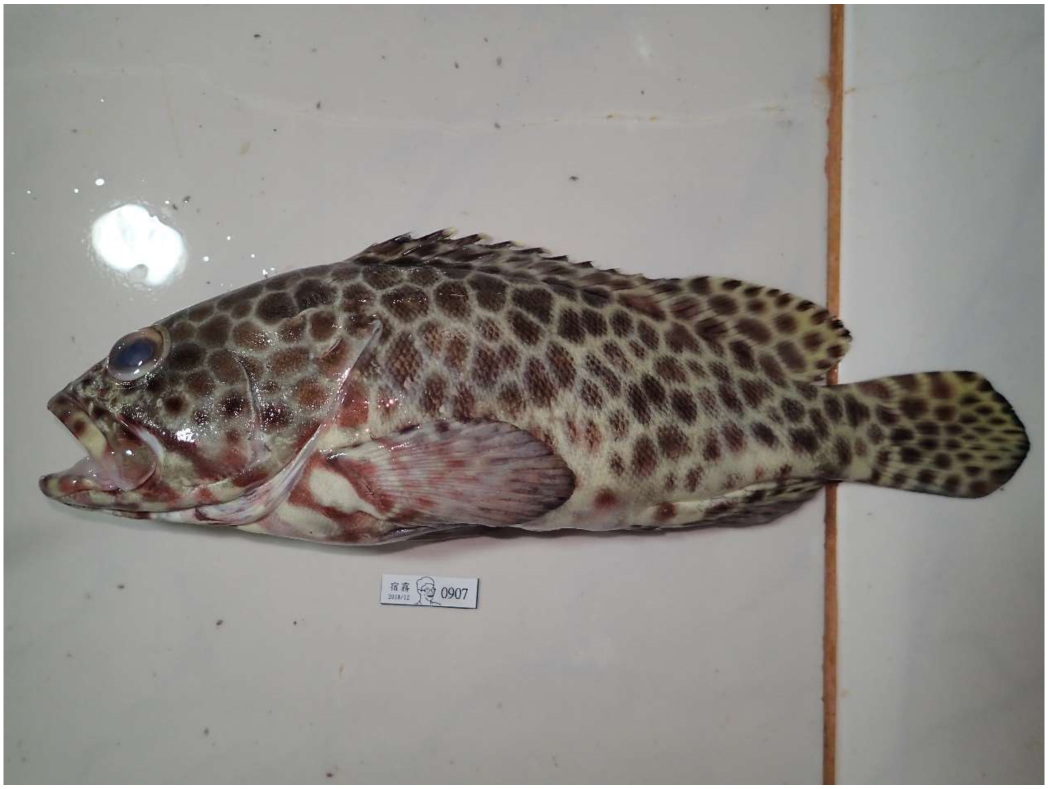

DOS 06952-1, *Epinephelus quoyanus*, OR114128. (specimen not preserved)

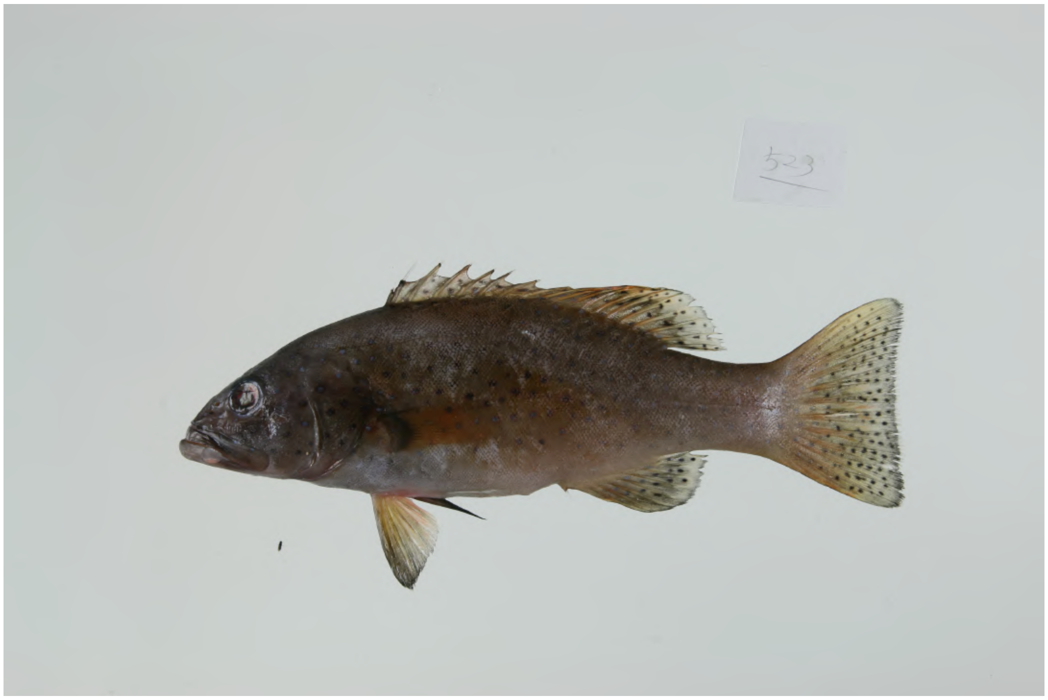

DOS 08661, *Plectropomus leopardus*, OR114237.

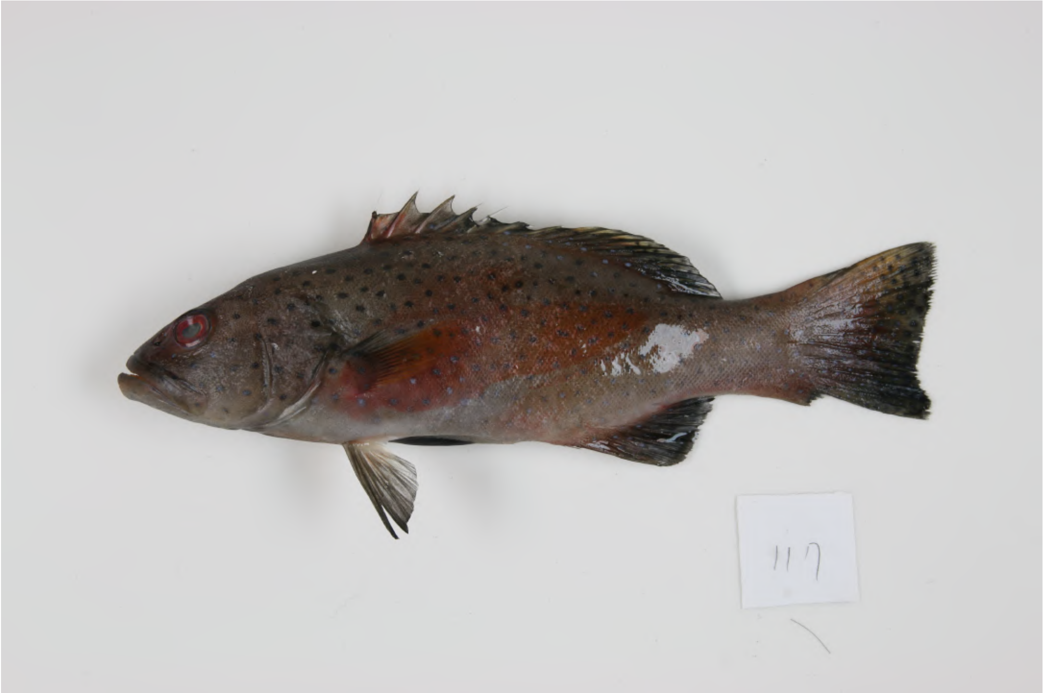

DOS 08662, *Plectropomus leopardus*, OR114238.

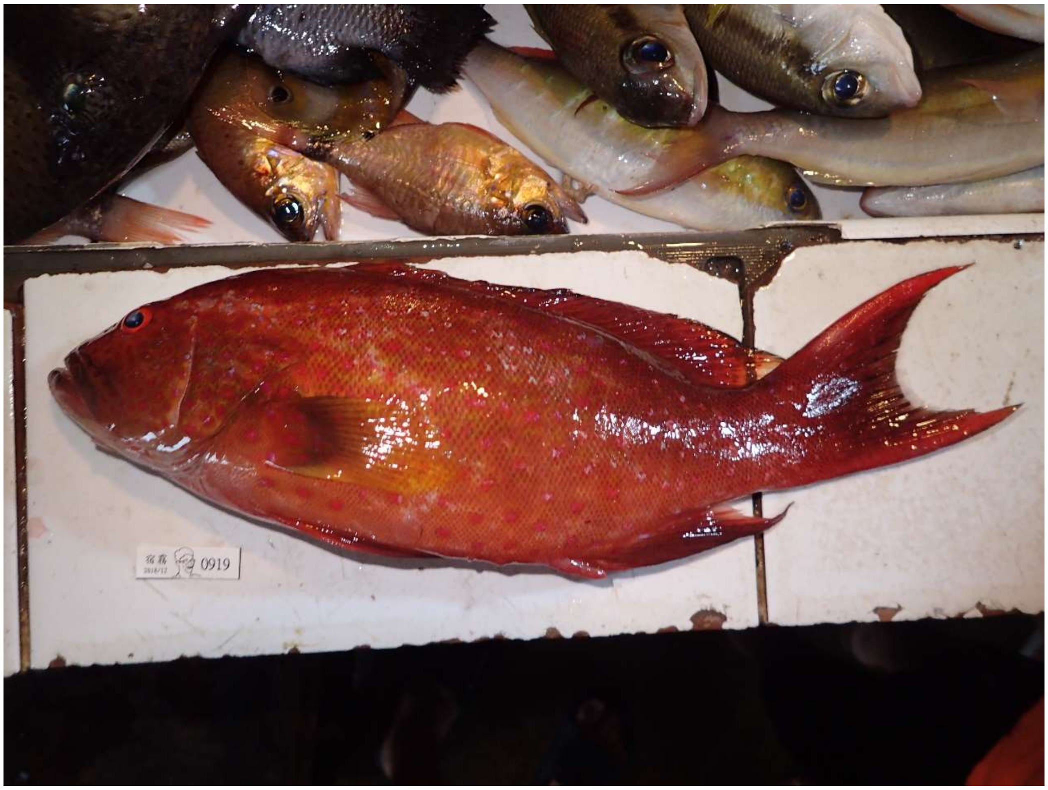

DOS 06956-1, *Variola albimarginata*, OR114132. (specimen not preserved)

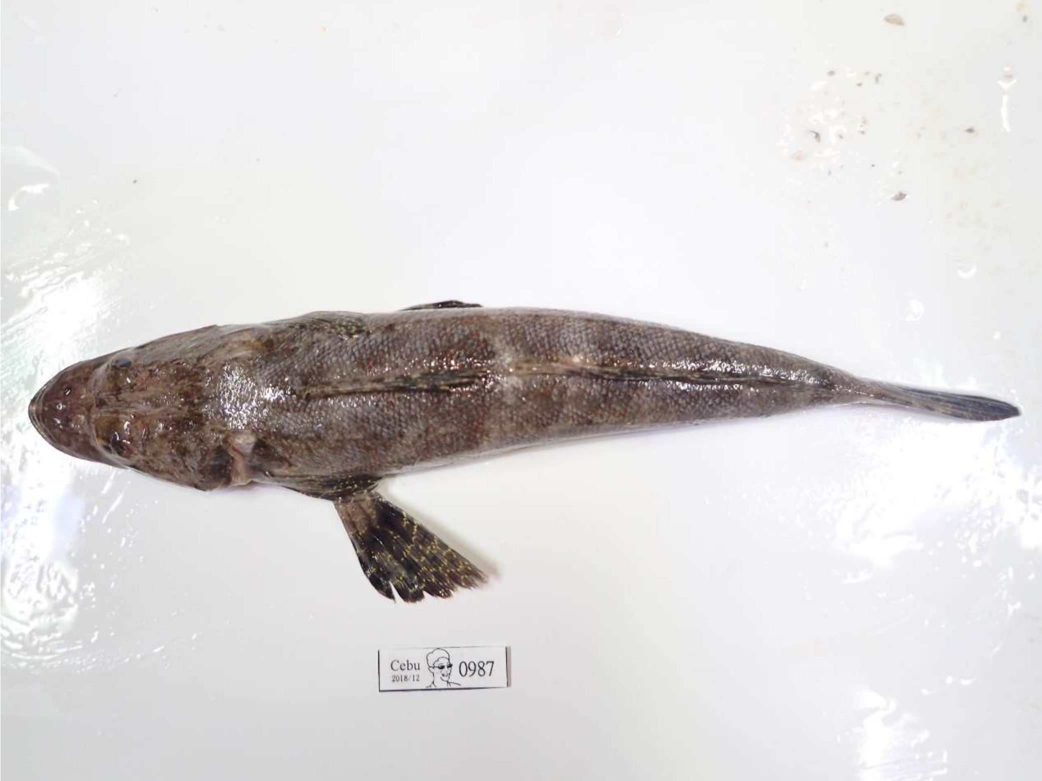

DOS 06869-1, *Cymbacephalus nematophthalmus*, OR114044.

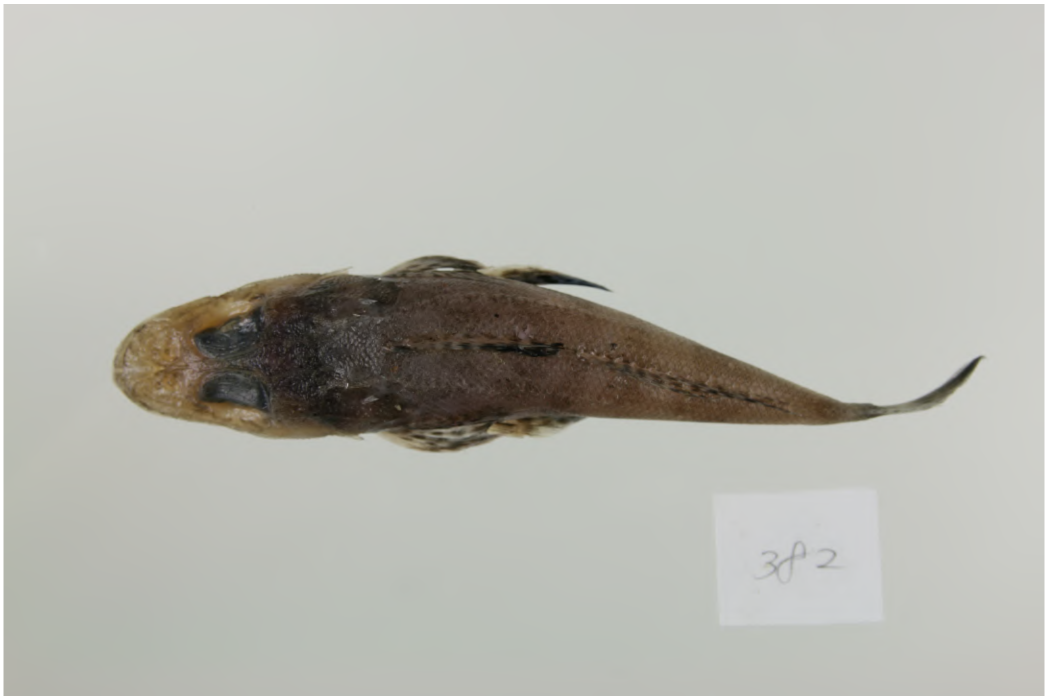

DOS 08677, *Rogadius pristiger*, OR114259.

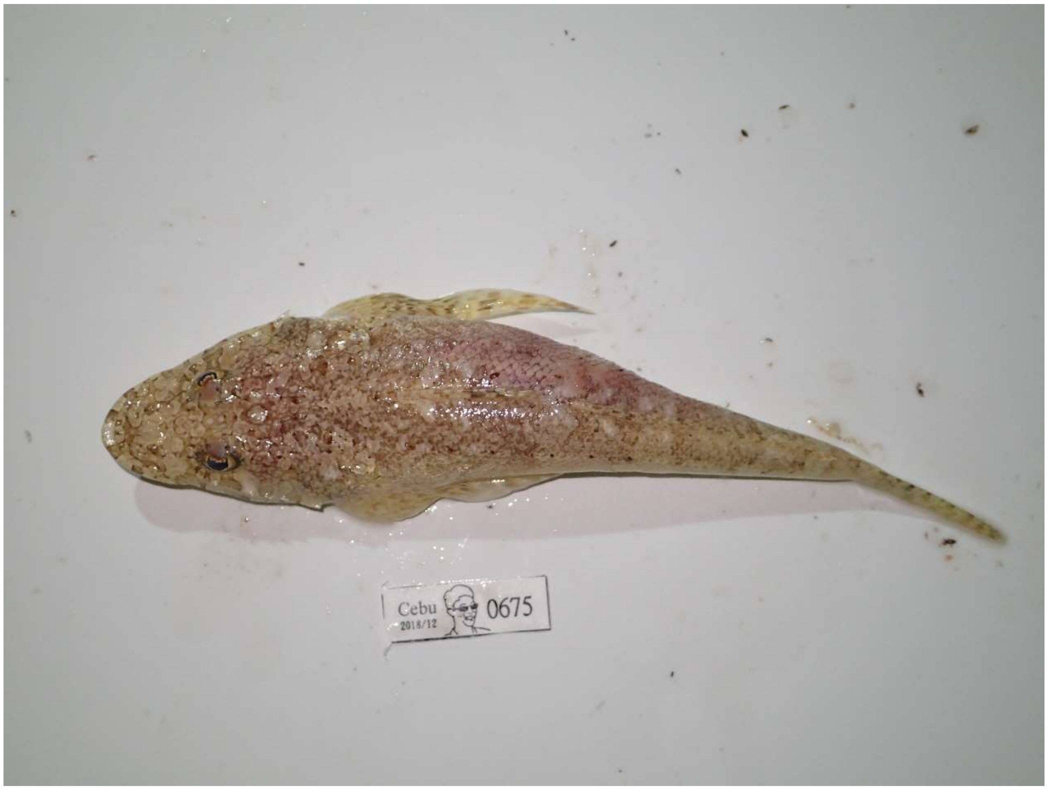

DOS 06870-1, *Sunagocia arenicola*, OR114045.

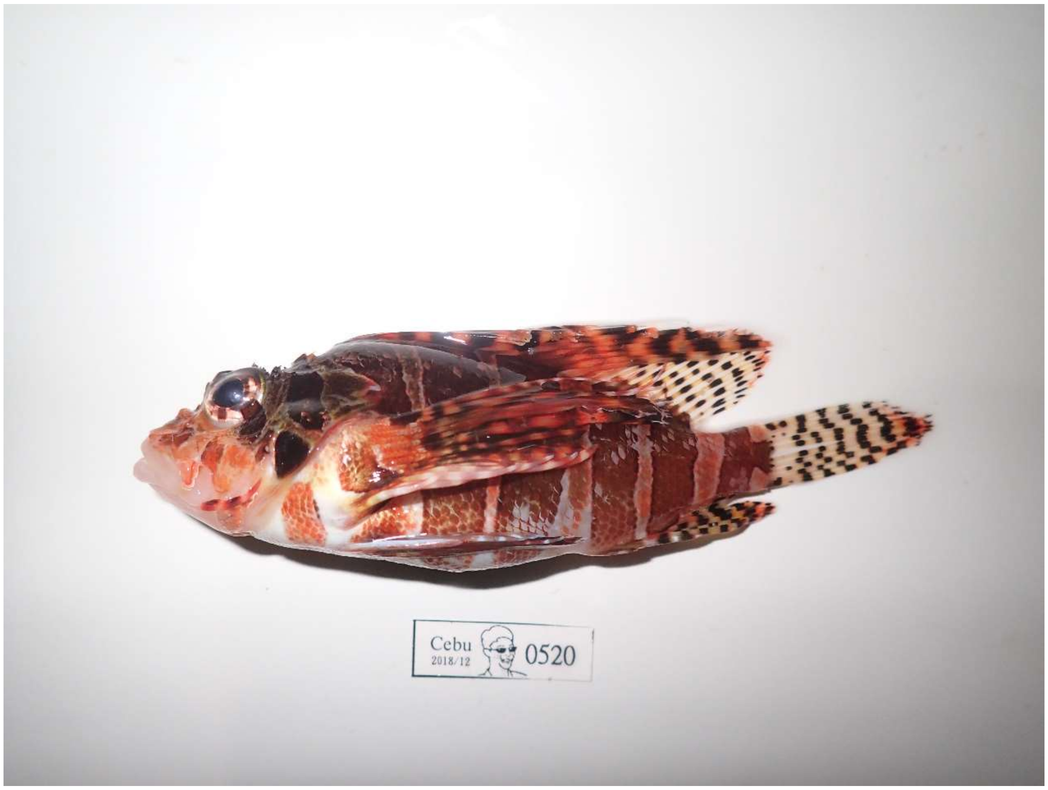

DOS 06932, *Dendrochirus zebra*, OR114108.

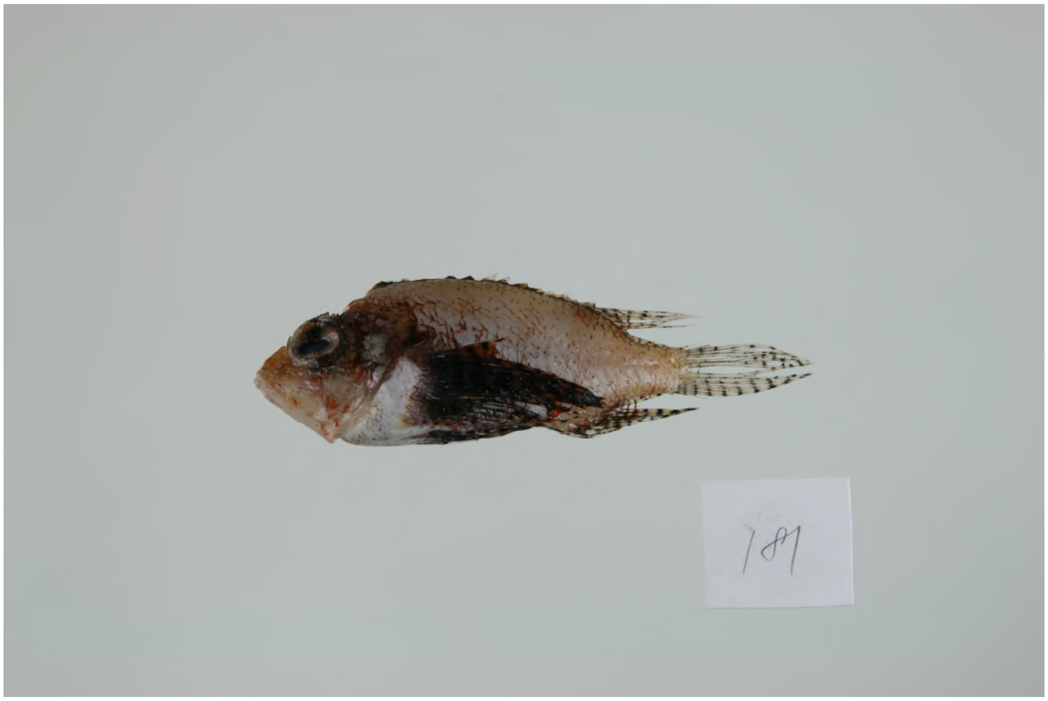

DOS 08678, *Neochirus brachyptera*, OR114260.

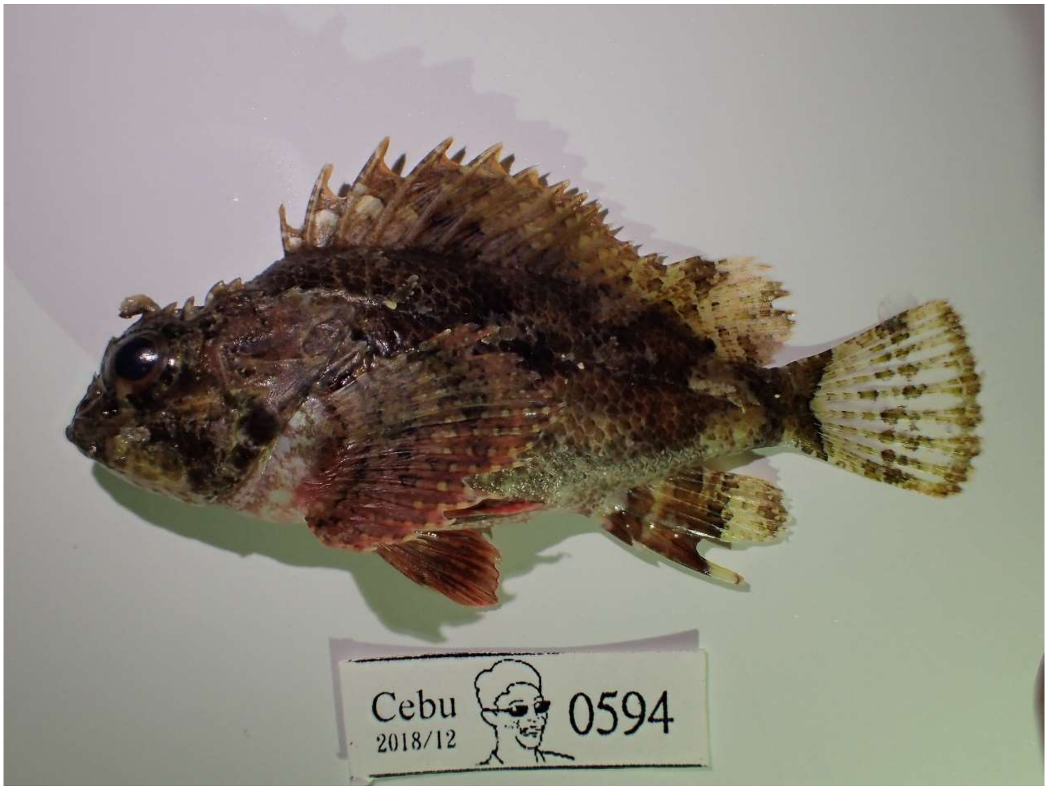

DOS 06933, *Parascorpaena mossambica*, OR114109.

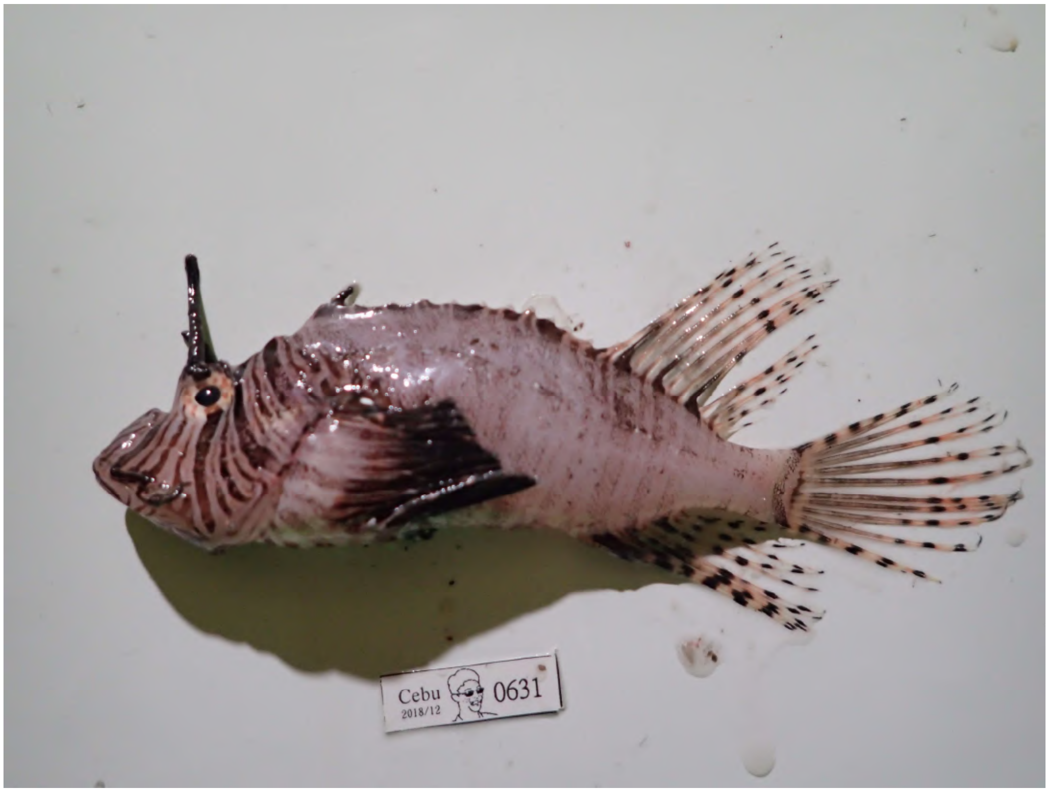

DOS 06935-1, *Pterois volitans*, OR114111.

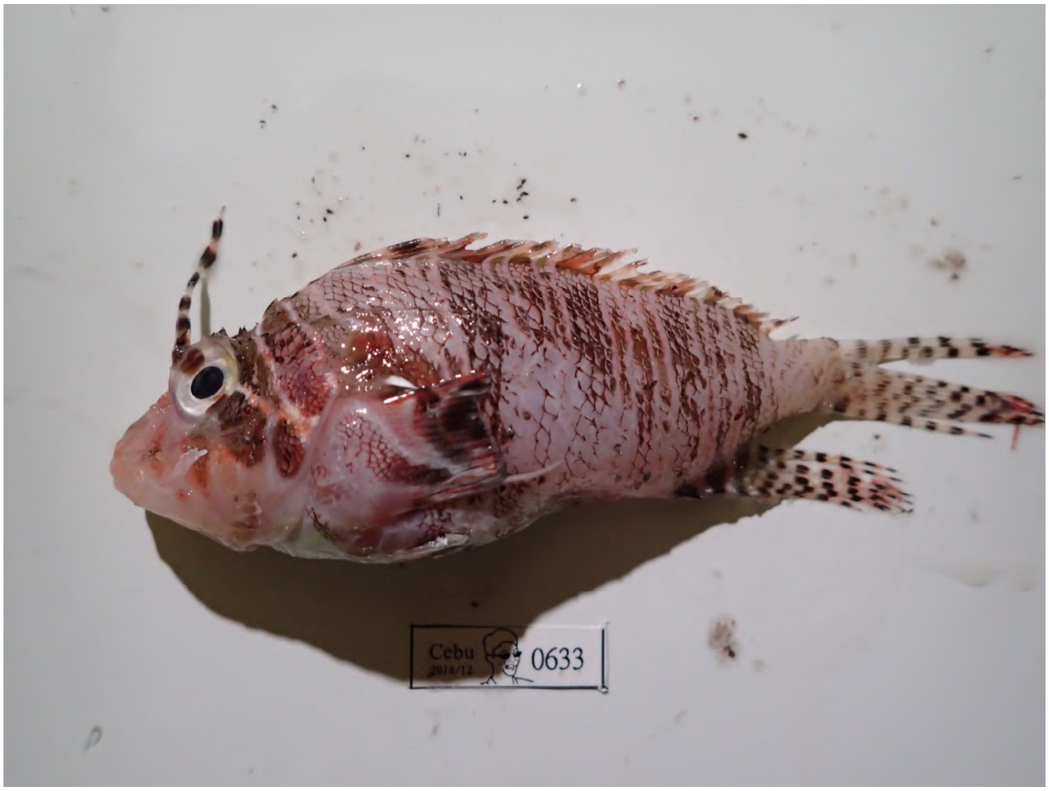

DOS 06934-2, *Pteropterus antennatus*, OR114110.

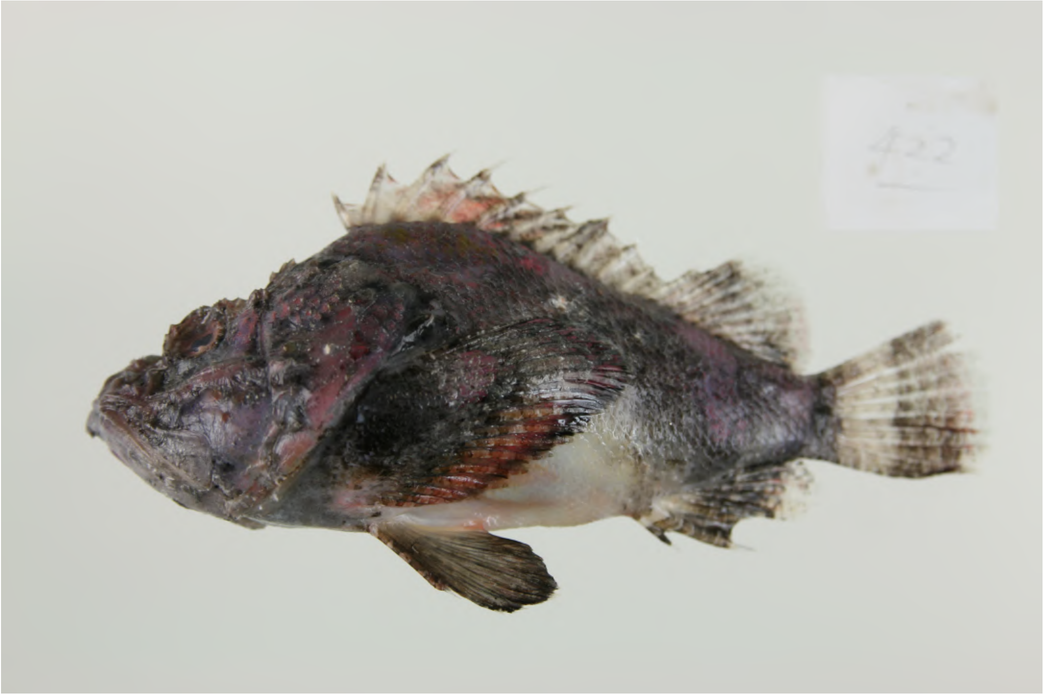

DOS 08679, *Scorpaenopsis neglecta*, OR114261.

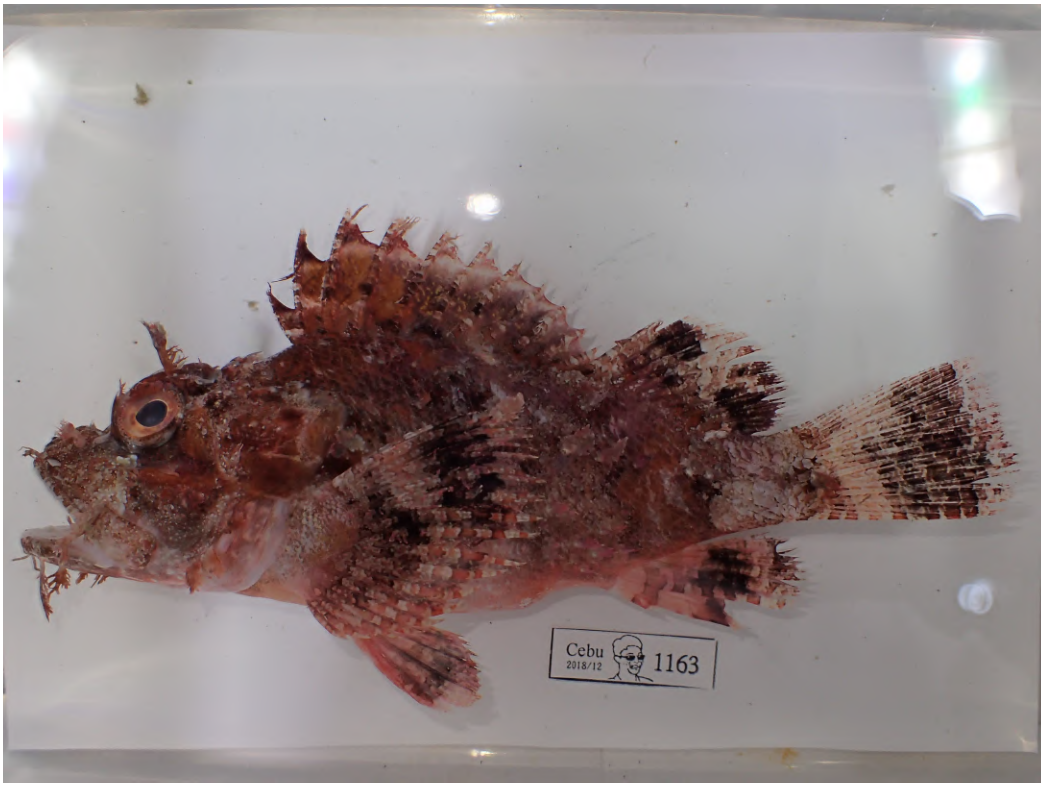

DOS 06936, *Scorpaenopsis venosa*, OR114112.

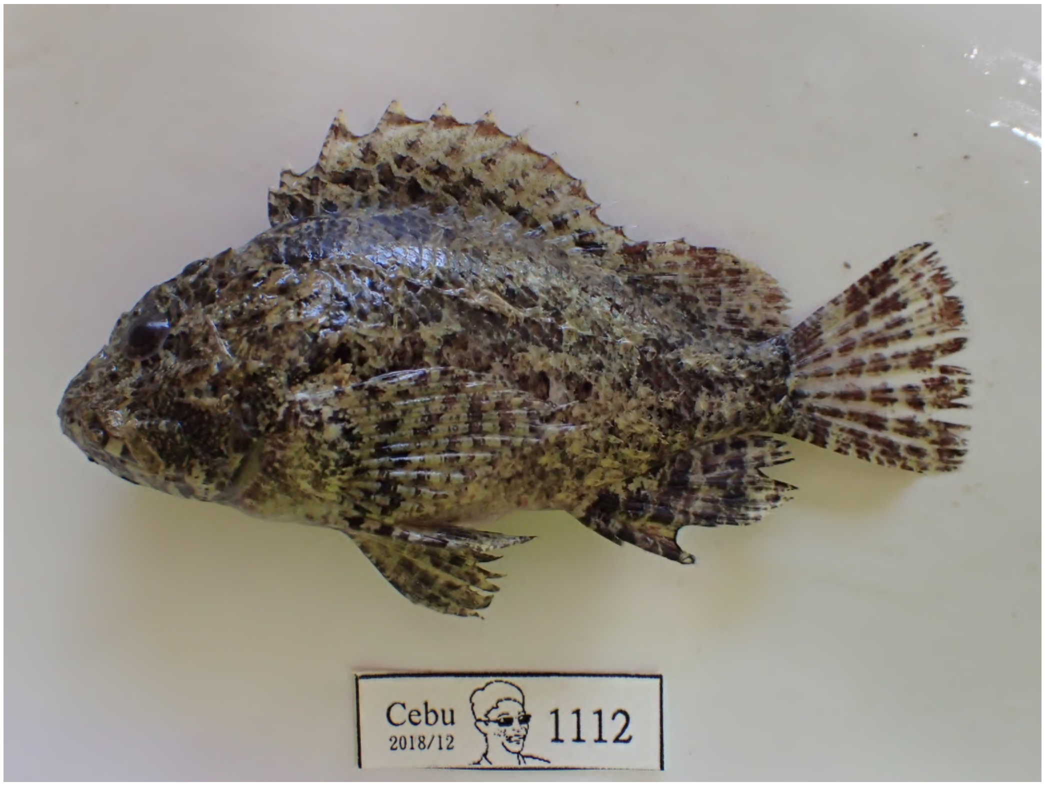

DOS 06937-2, *Sebastapistes strongia*, OR114113. (specimen not preserved)

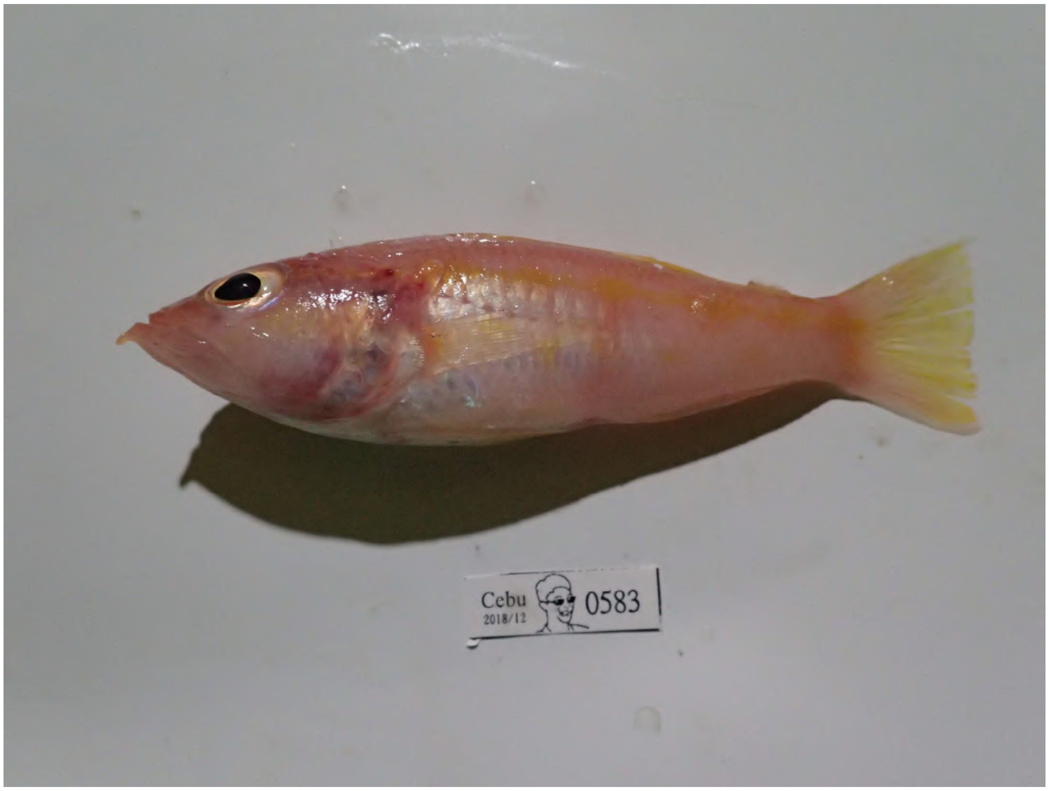

DOS 06868-1, *Chelidoperca* sp., OR114043.

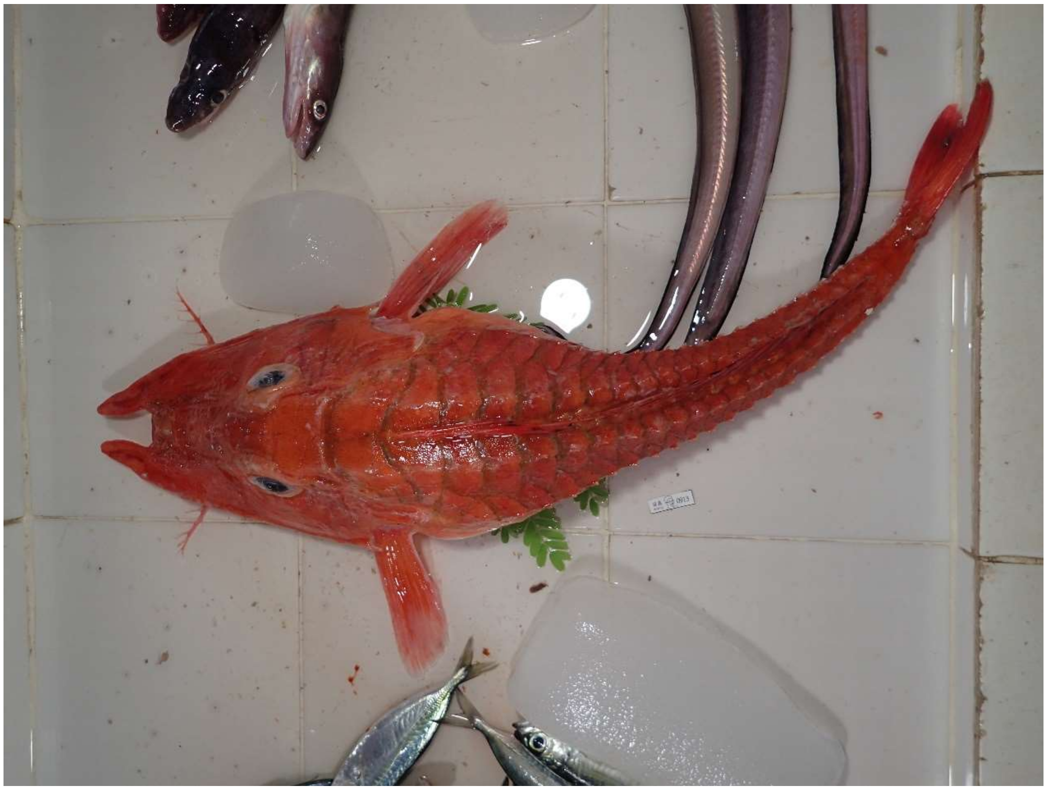

DOS 06993, *Satyrichthys laticeps*, OR114162. (specimen not preserved)

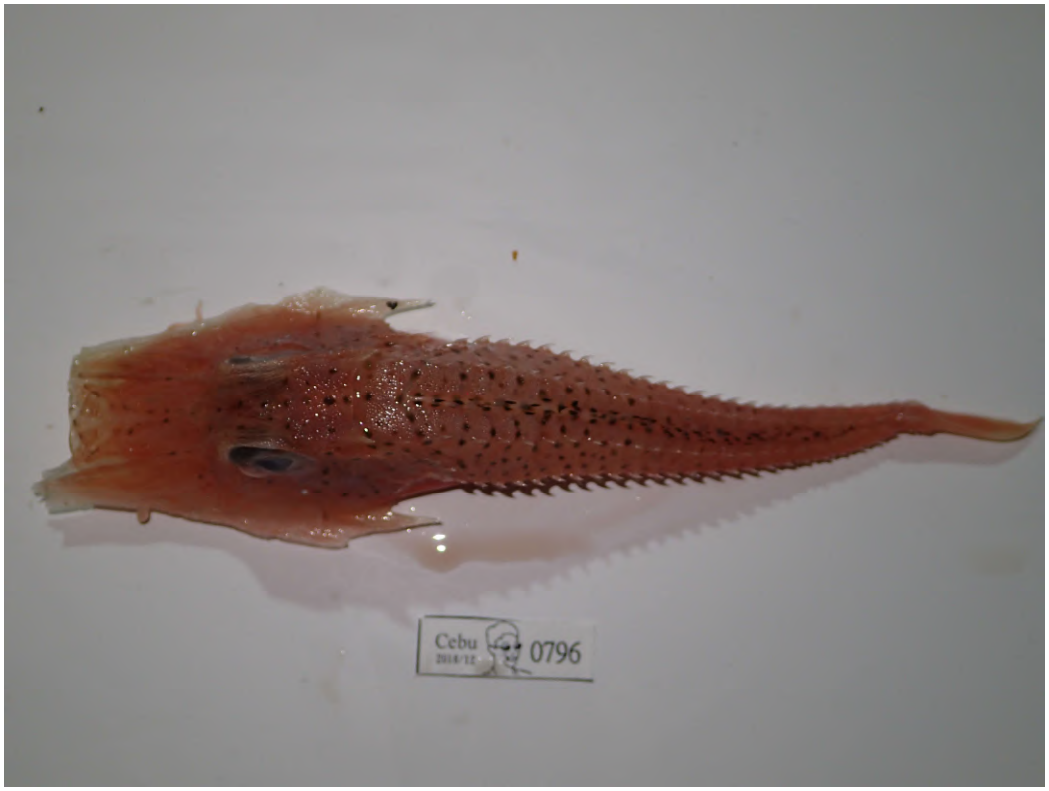

DOS 06865-1, *Satyrichthys rieffeli*, OR114040.

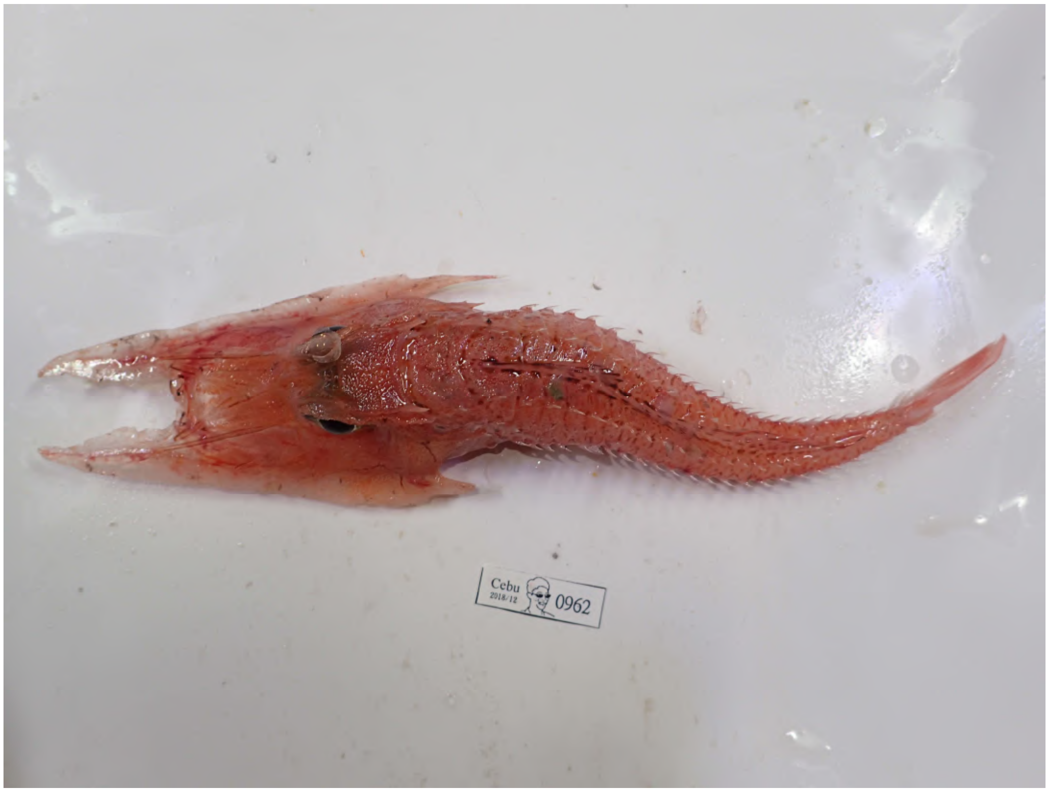

DOS 06866, *Satyrichthys welchi*, OR114041.

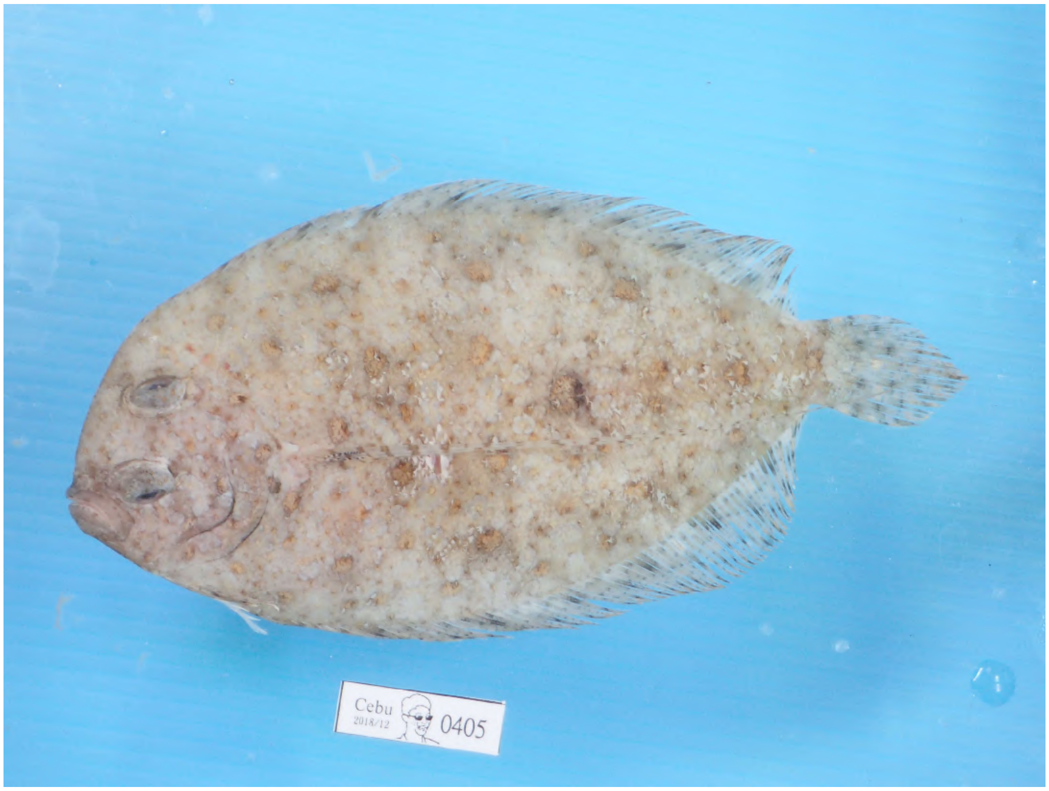

DOS 06622, *Bothus pantherinus*, OR113813.

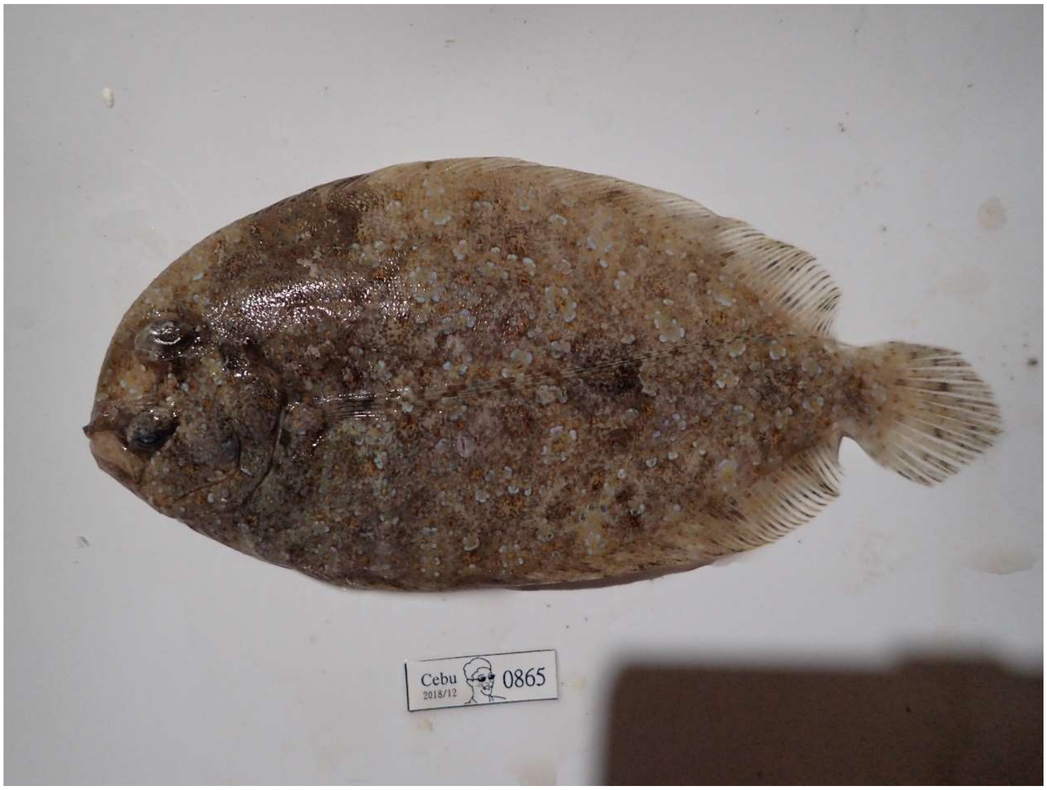

DOS 06623, *Bothus pantherinus*, OR113814.

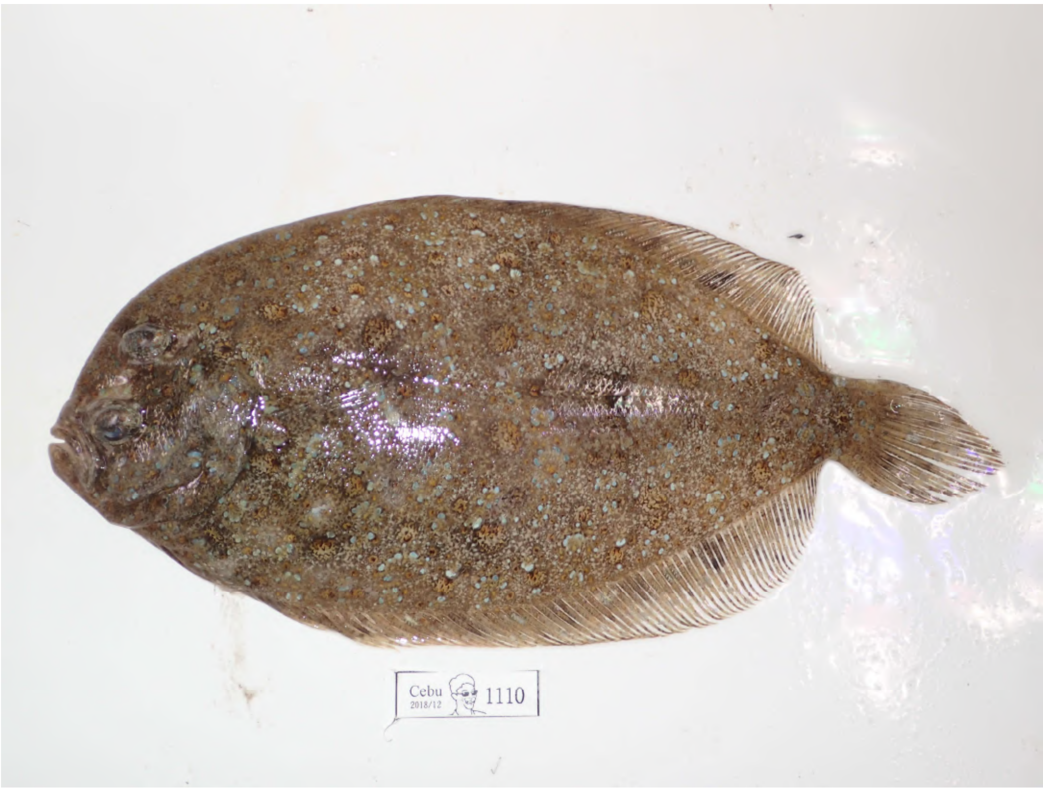

DOS 06864-1, *Bothus pantherinus*, OR114039.

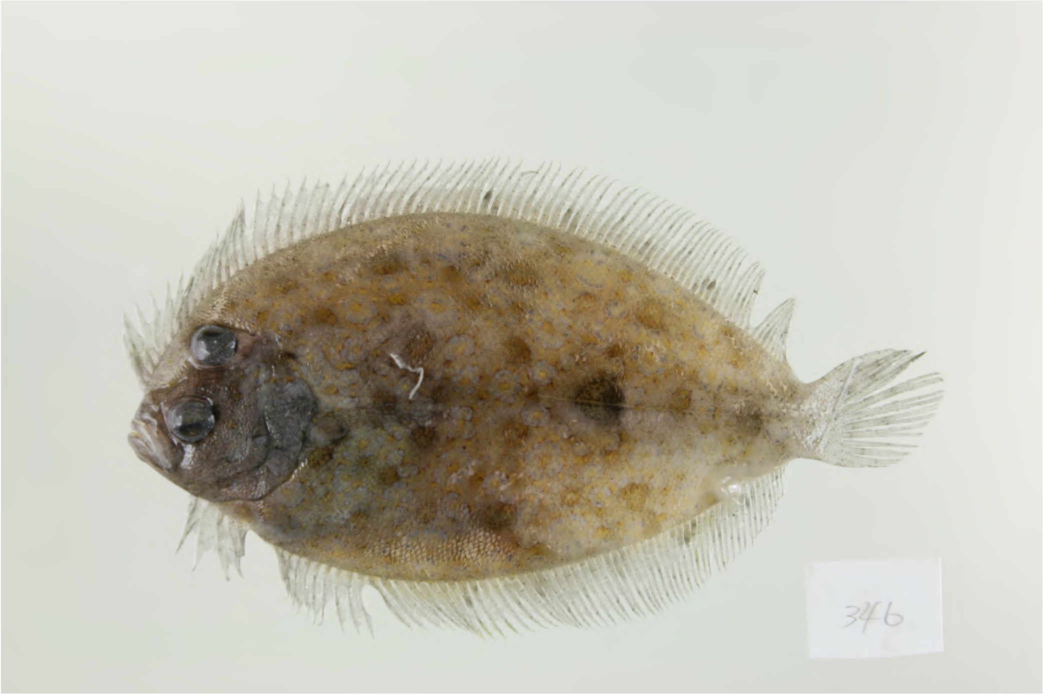

DOS 08669, *Bothus pantherinus*, OR114247.

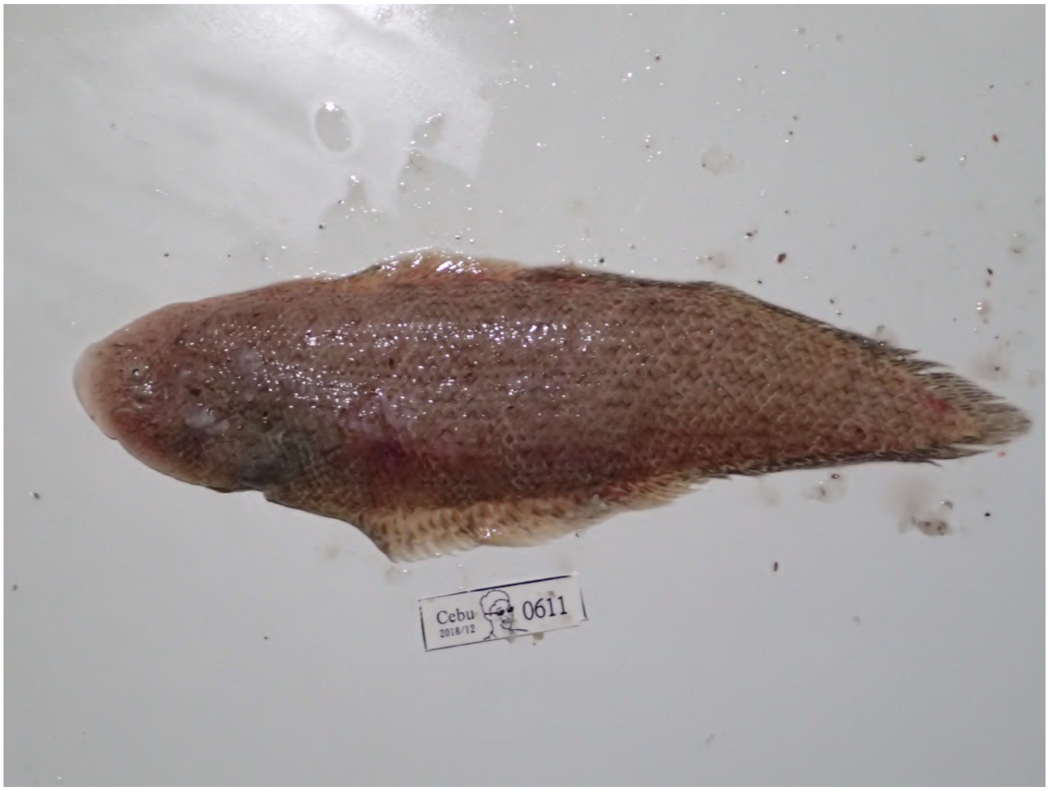

DOS 06683-1, *Cynoglossus* sp., OR113873.

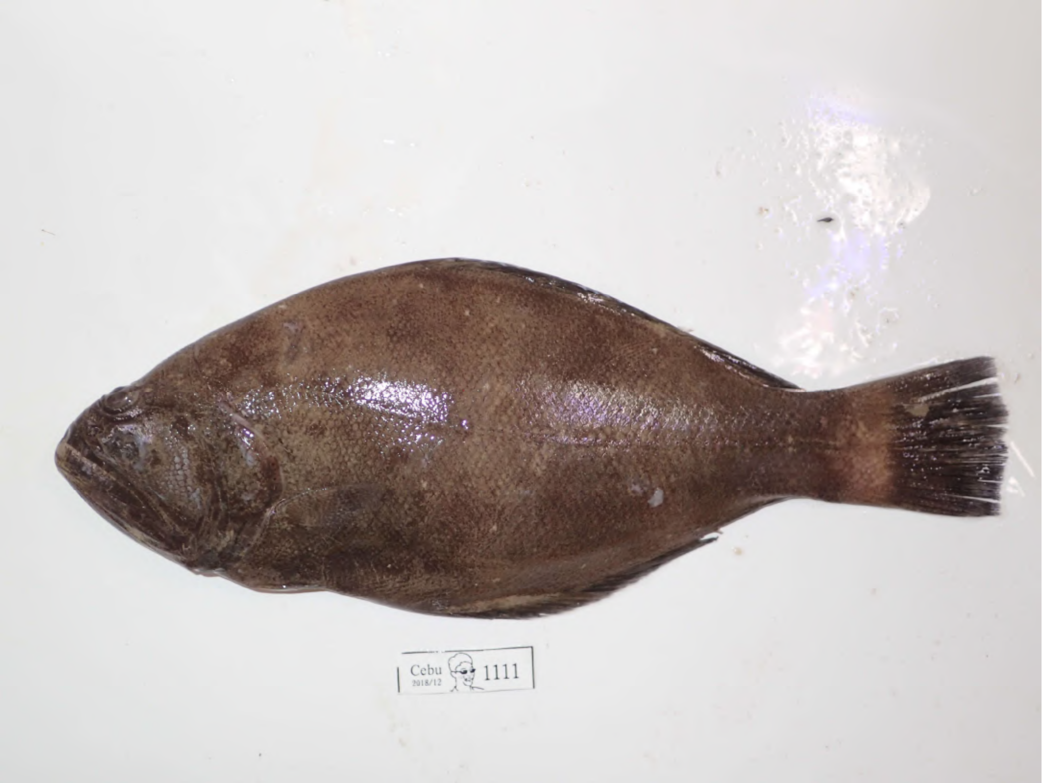

DOS 06909-2, *Psettodes erumei*, OR114086.

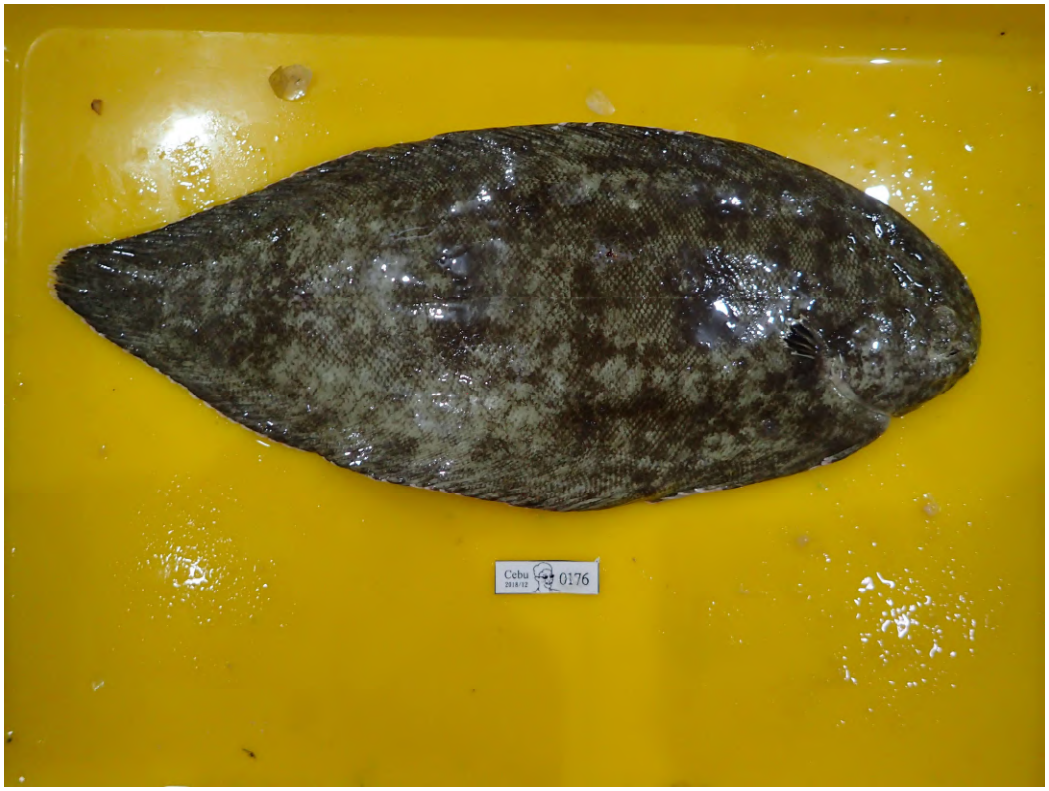

DOS 06964, *Brachirus aspilos*, OR114139. (specimen not preserved)

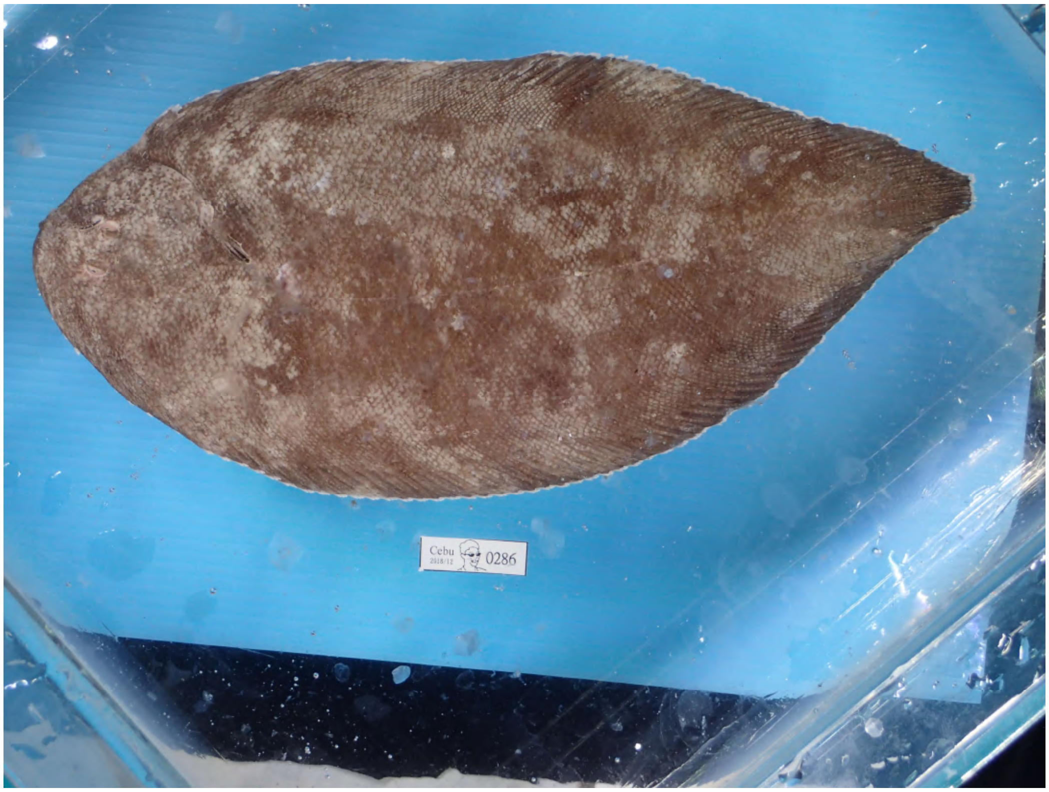

DOS 06966-1, *Brachirus aspilos*, OR114140. (specimen not preserved)

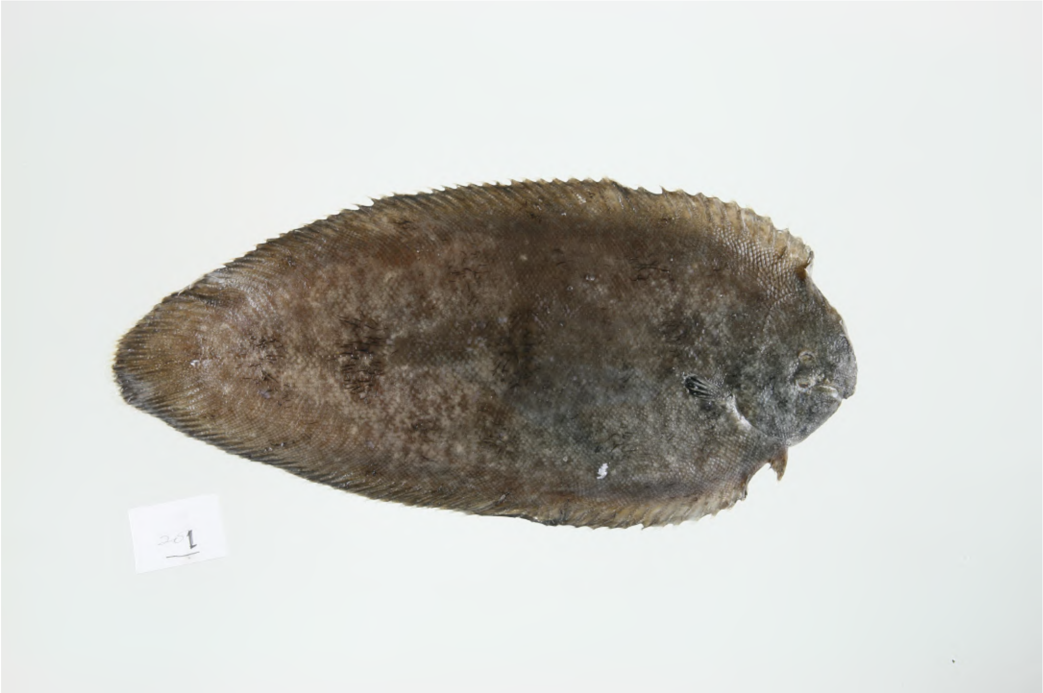

DOS 08670-1, *Brachirus aspilos*, OR114248.

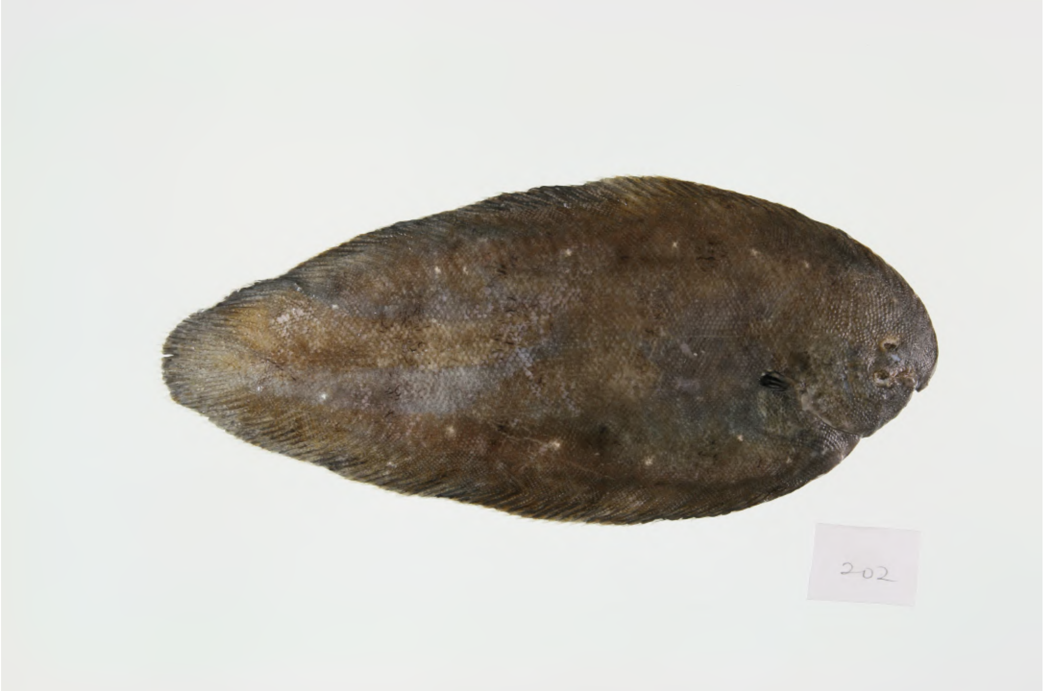

DOS 08670-2, *Brachirus aspilos*, OR114249.

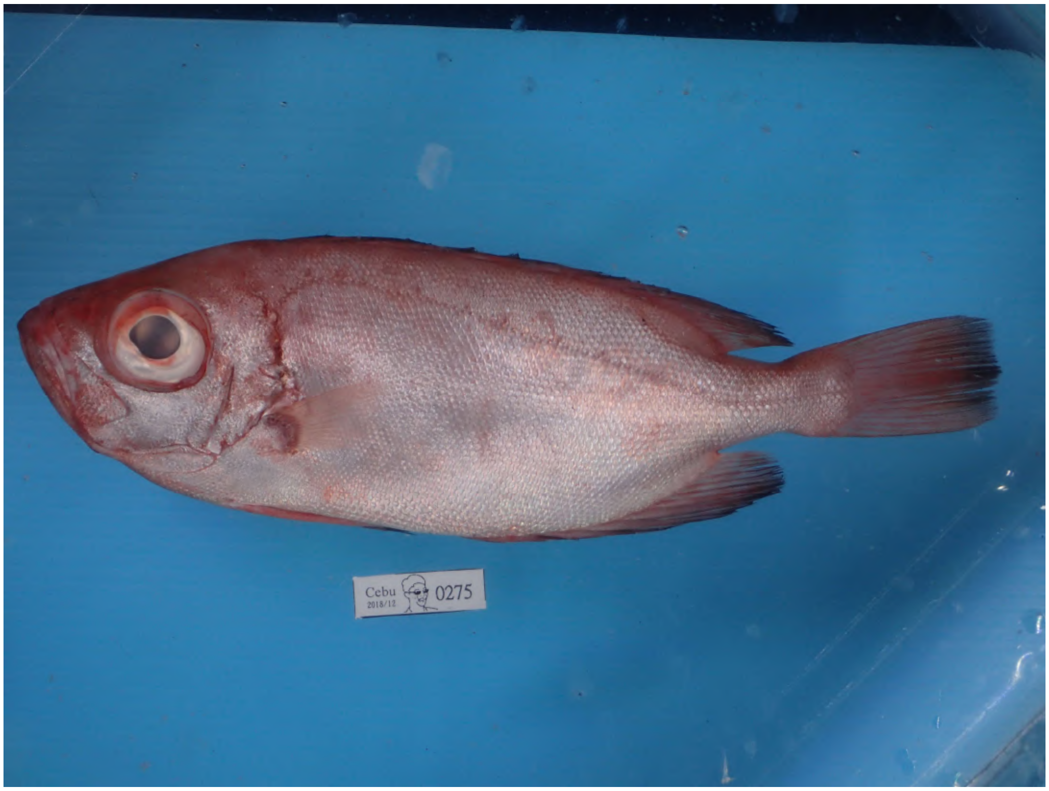

DOS 06908-1, *Priacanthus blochii*, OR114085.

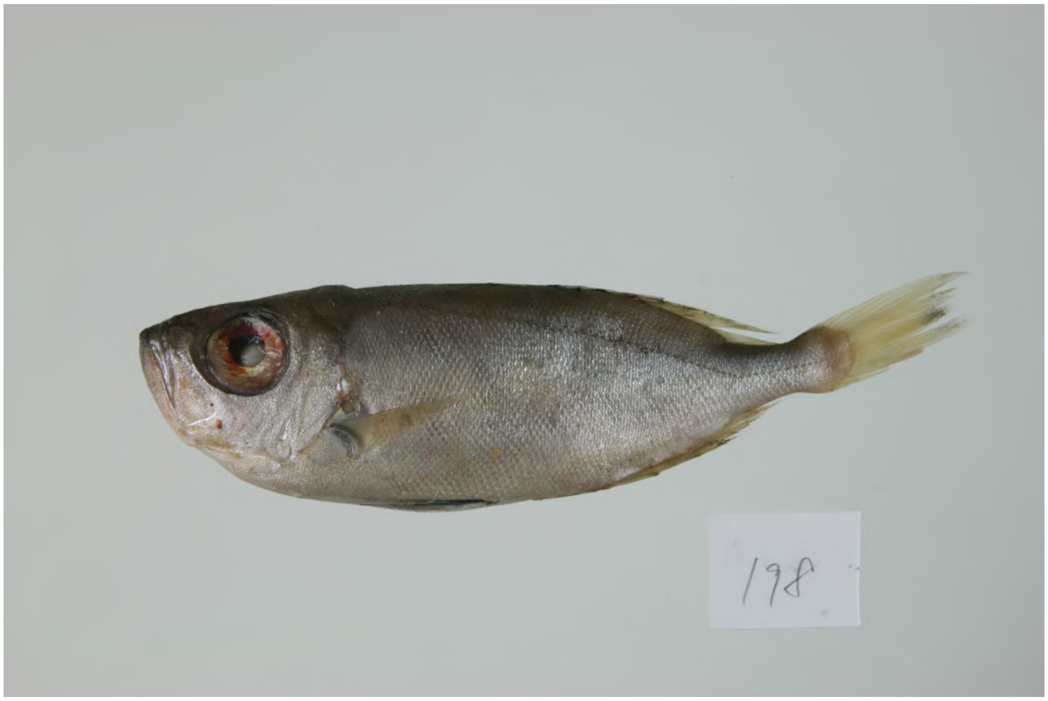

DOS 08660, *Priacanthus macracanthus*, OR114236.

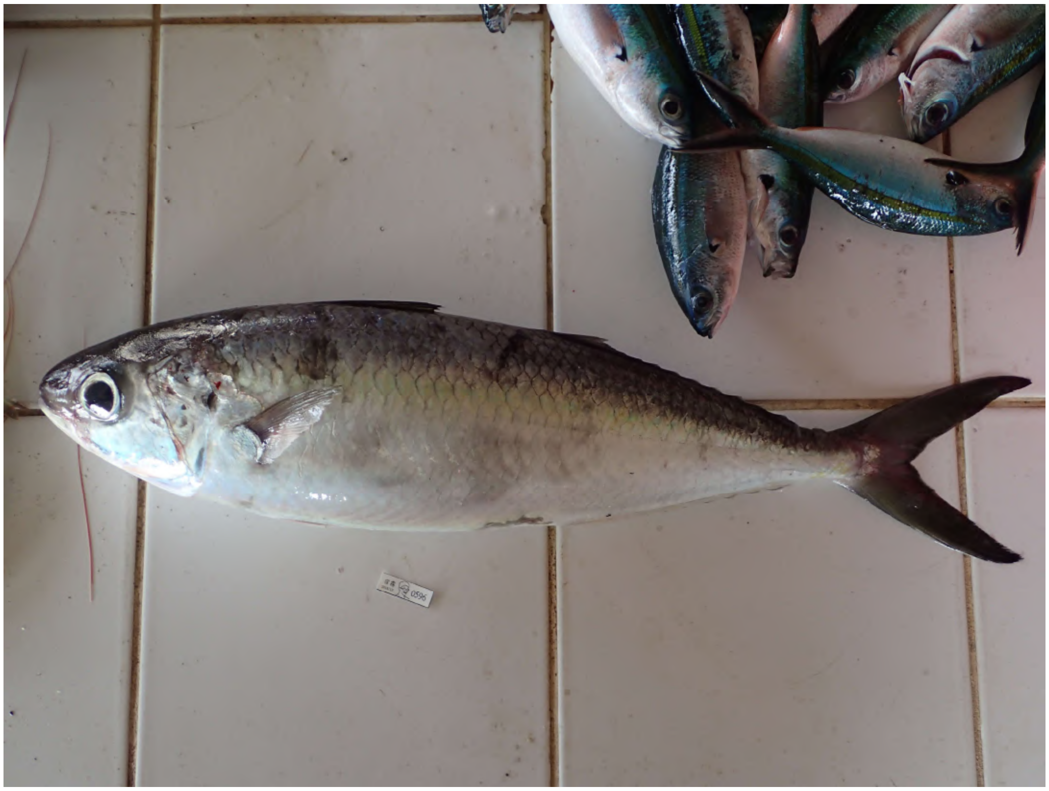

DOS 06859, *Ariomma brevimanus*, OR114035. (specimen not preserved)

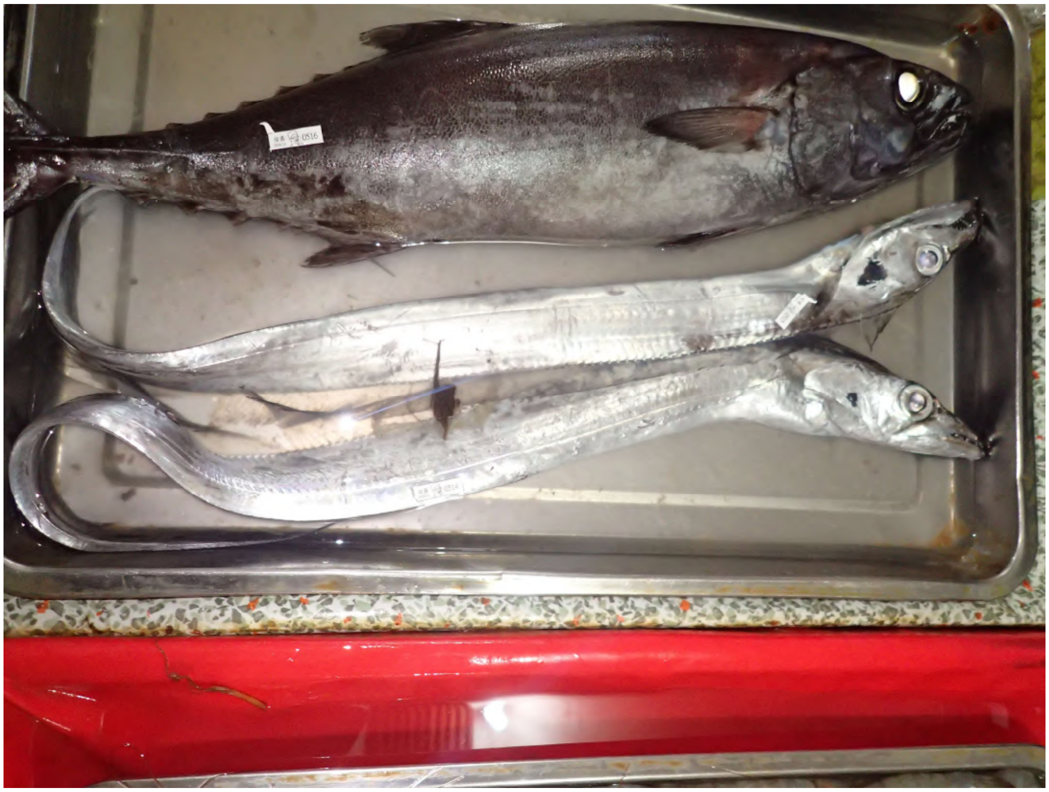

DOS 06699, *Lepidocybium flavobrunneum*, OR113887. (top, specimen not preserved)

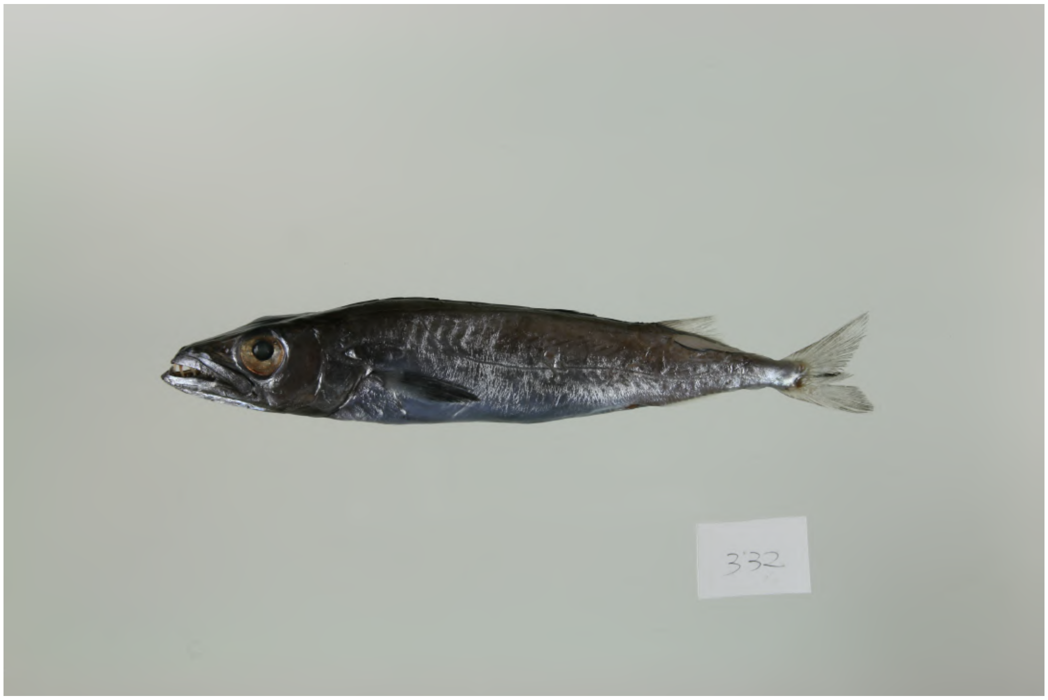

DOS 08671, *Rexea bengalensis*, OR114250.

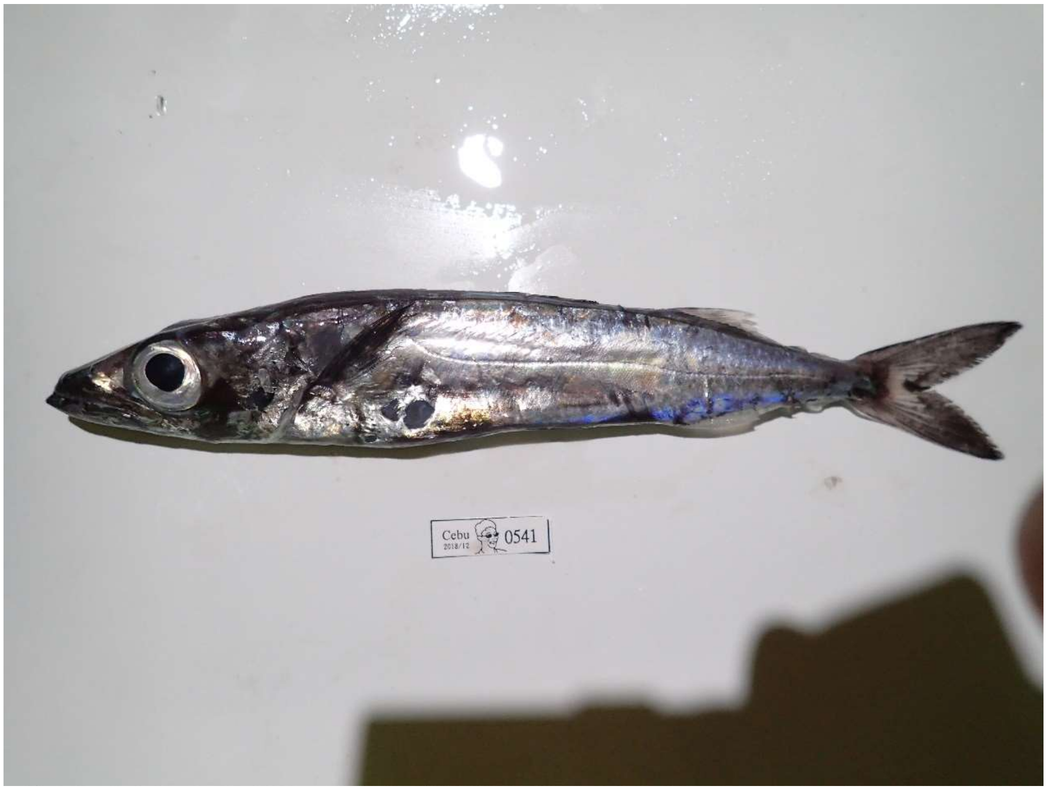

DOS 06700-1, *Rexea prometheoides*, OR113888.

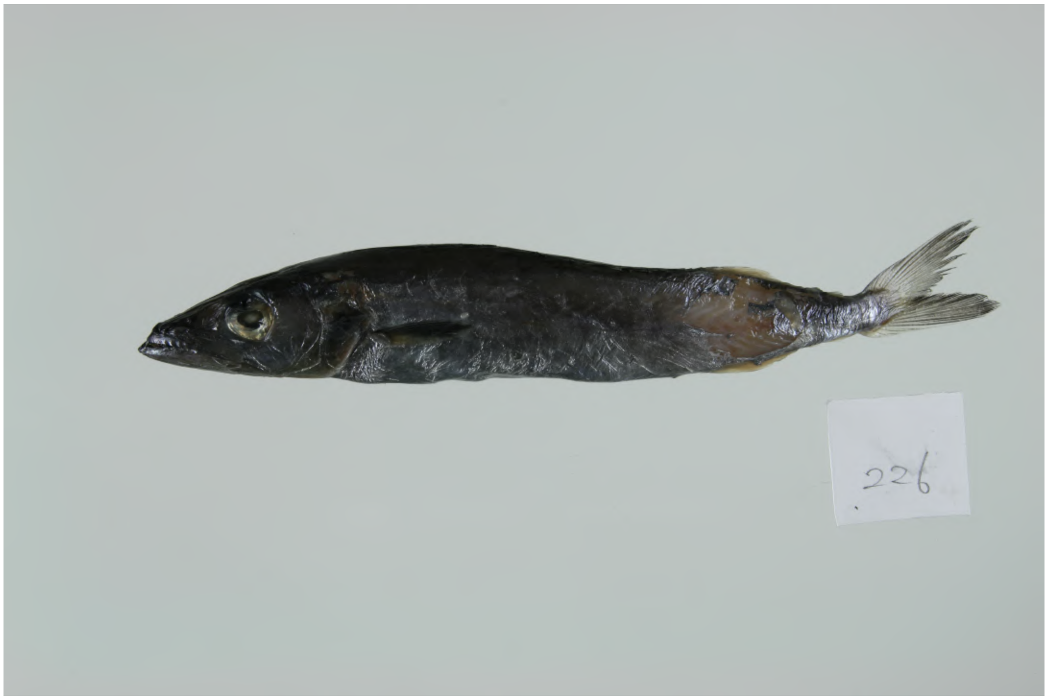

DOS 08672, *Rexea prometheoides*, OR114251.

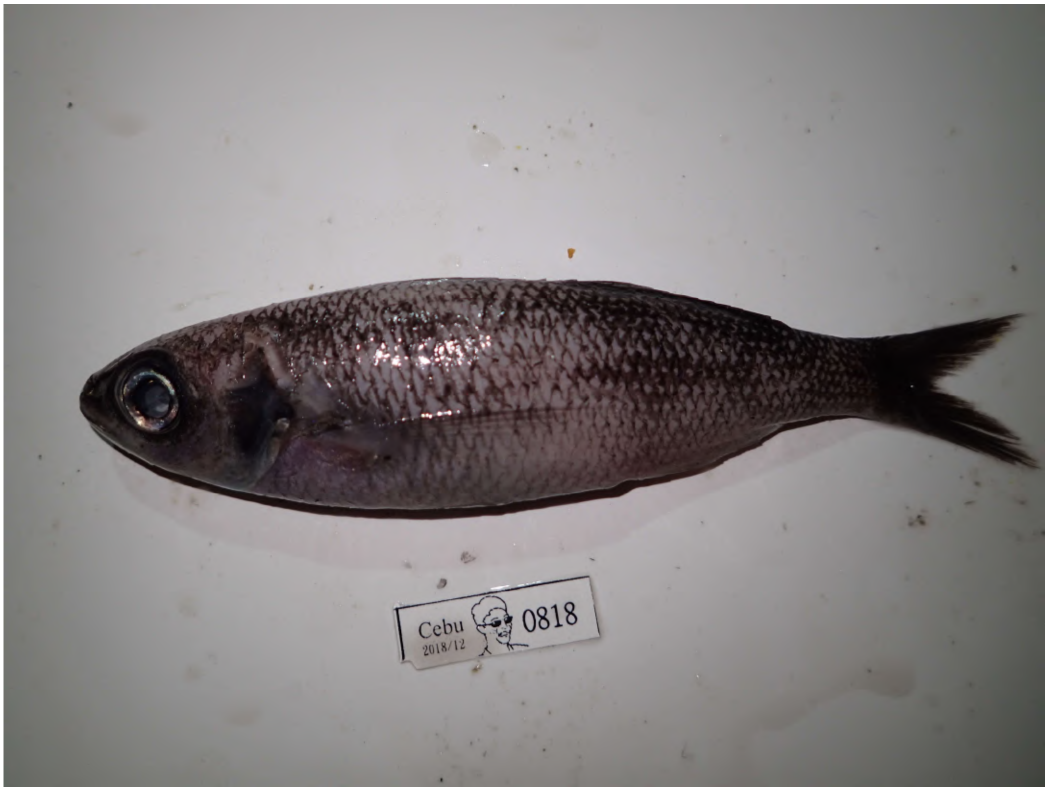

DOS 06656-1, *Cubiceps pauciradiatus*, OR113847.

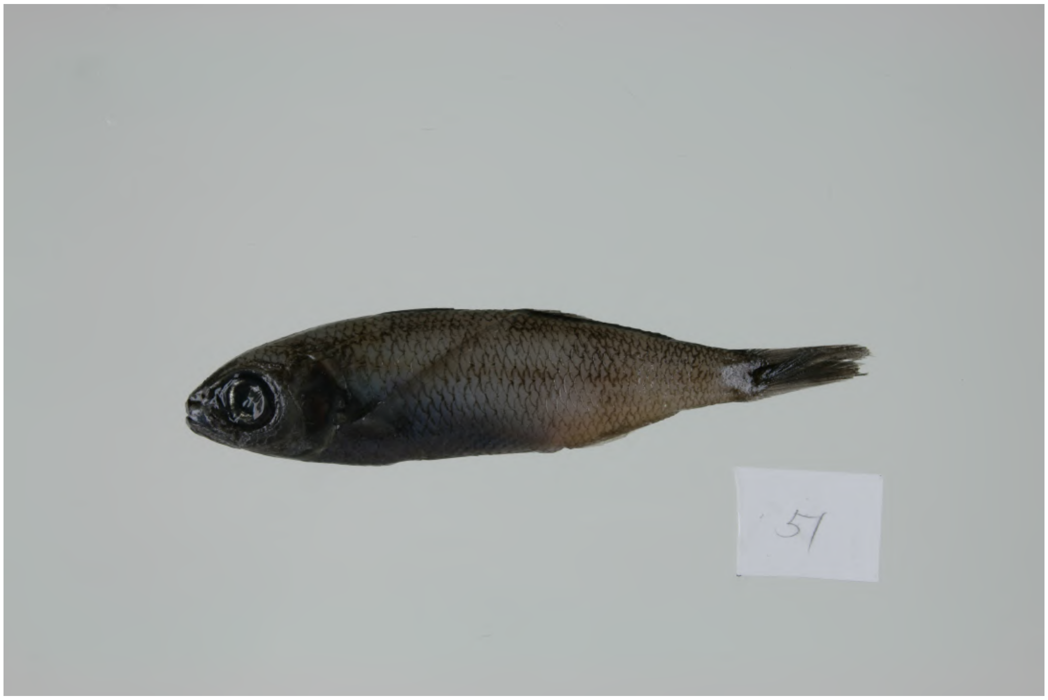

DOS 08673-1, *Cubiceps pauciradiatus*, OR114252.

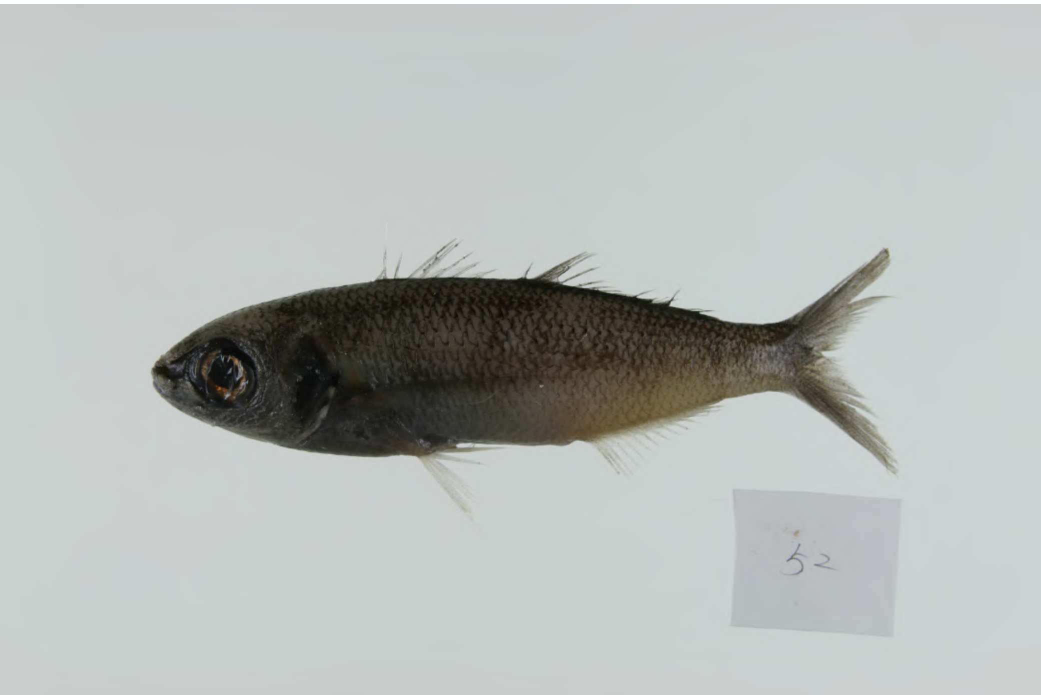

DOS 08673-2, *Cubiceps pauciradiatus*, OR114253.

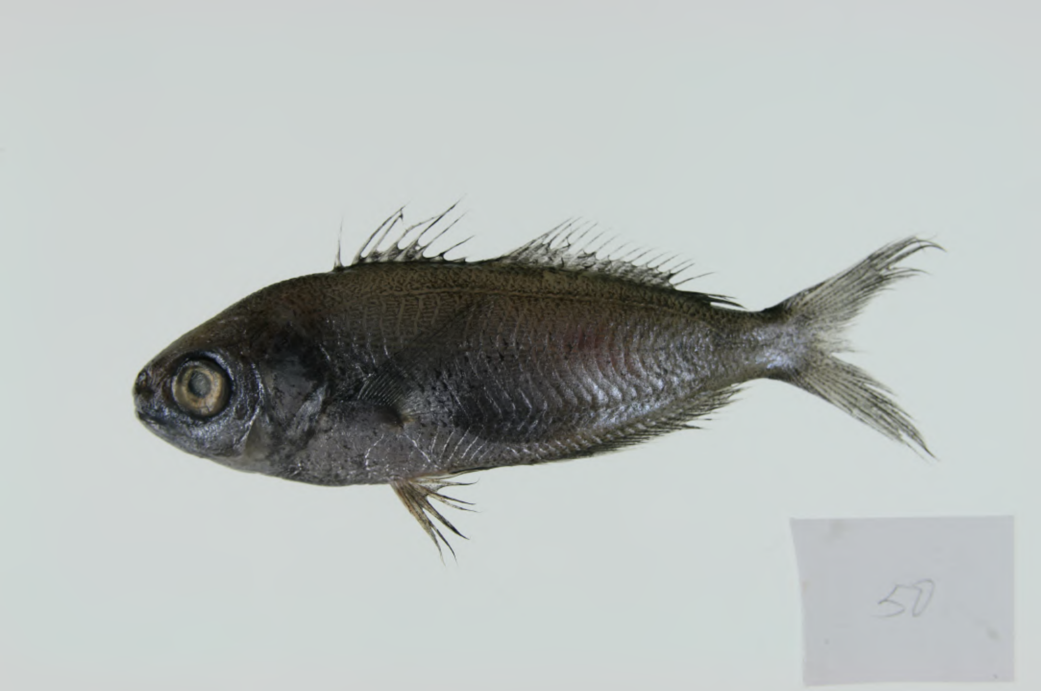

DOS 08674, *Cubiceps whiteleggii*, OR114254.

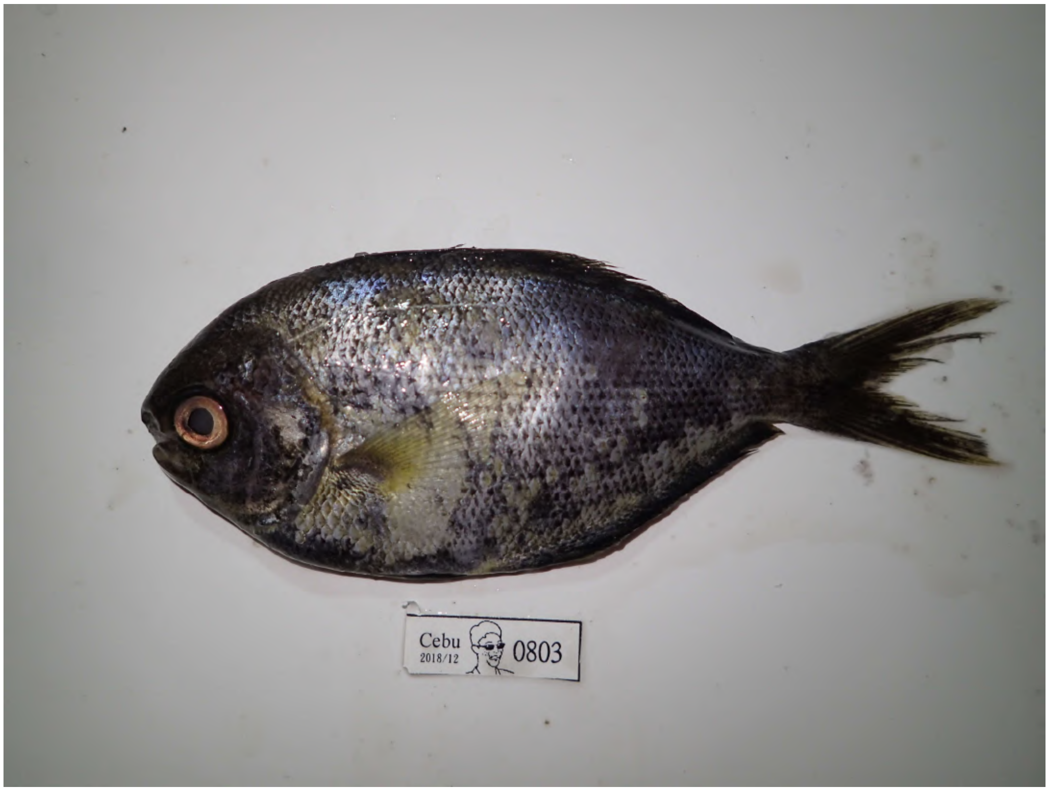

DOS 06860-1, *Psenes cyanophrys*, OR114036.

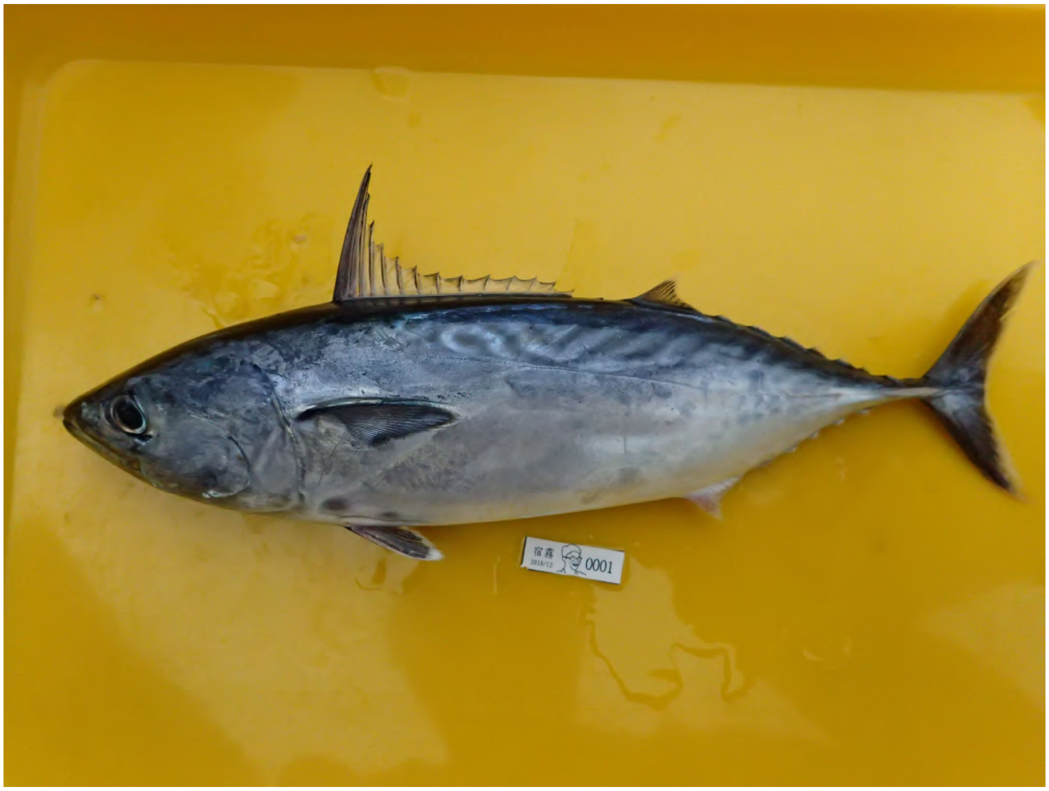

DOS 06925-1, *Euthynnus affinis*, OR114101. (specimen not preserved)

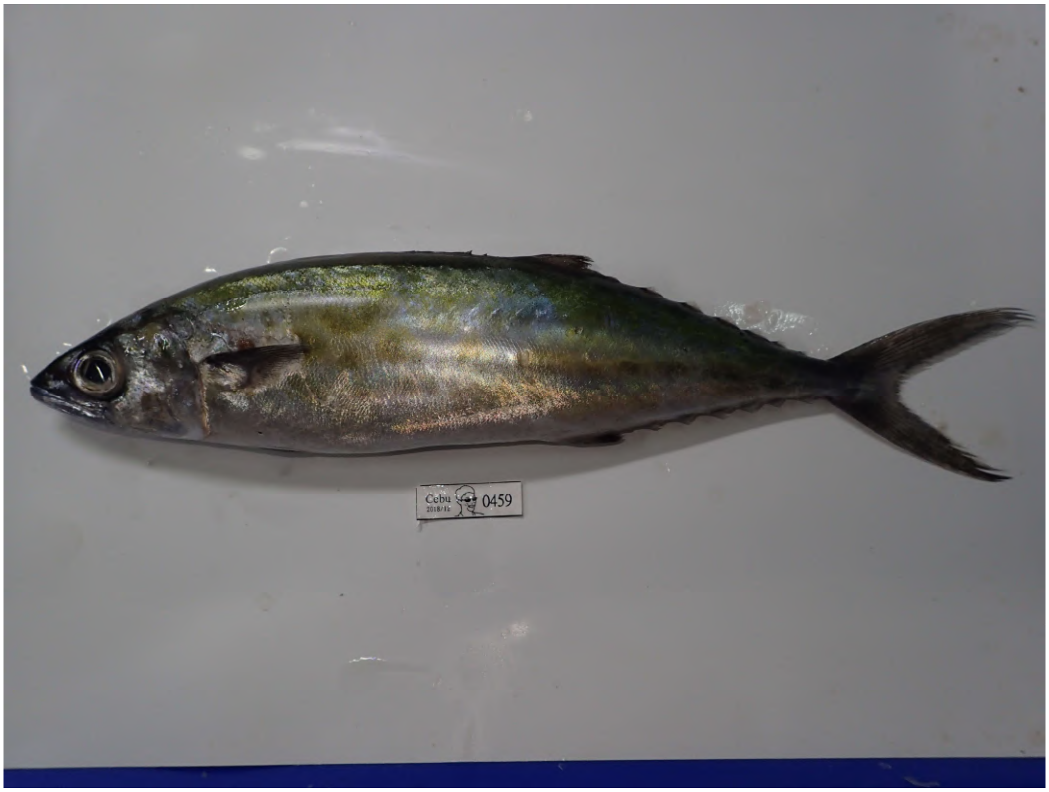

DOS 06930, *Grammatorcynus bilineatus*, OR114106.

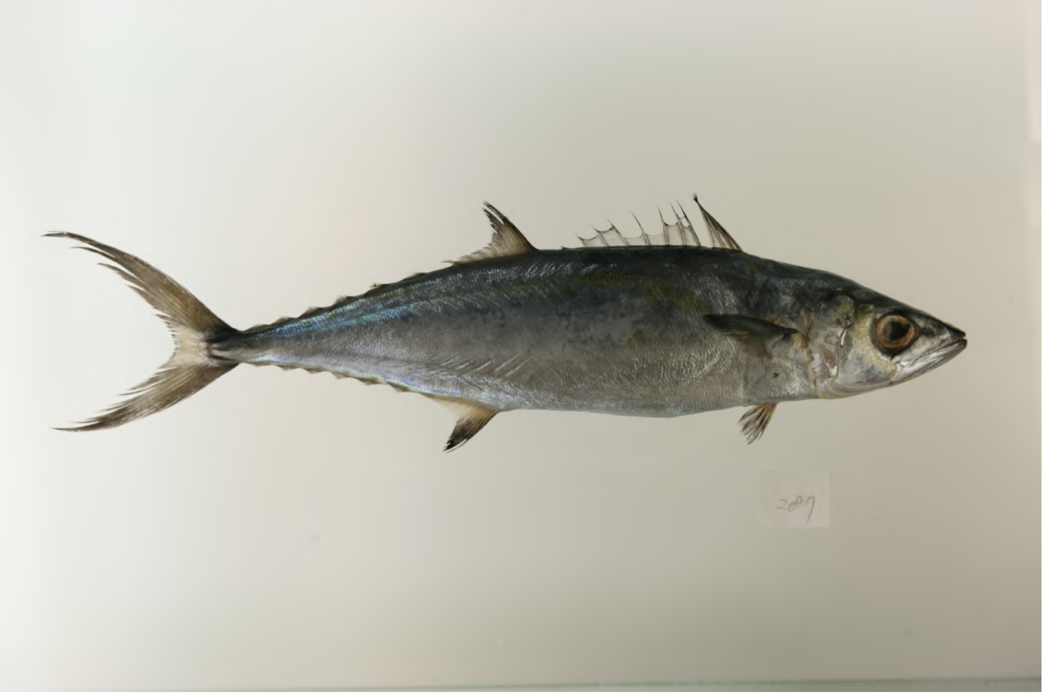

DOS 08675-1, *Grammatorcynus bilineatus*, OR114255.

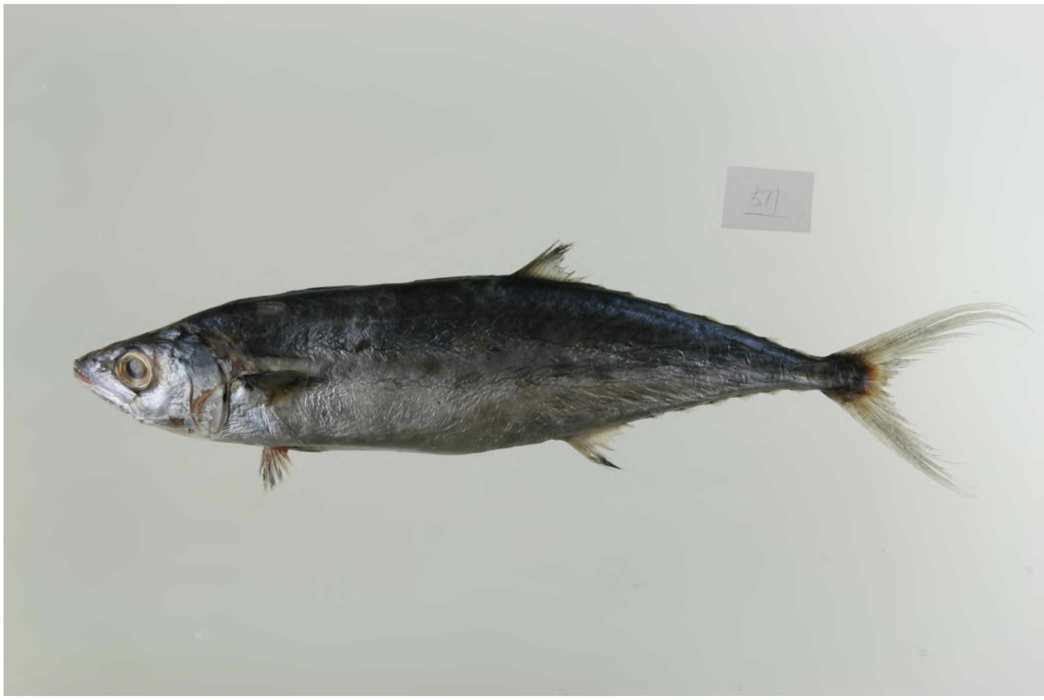

DOS 08675-2, *Grammatorcynus bilineatus*, OR114256.

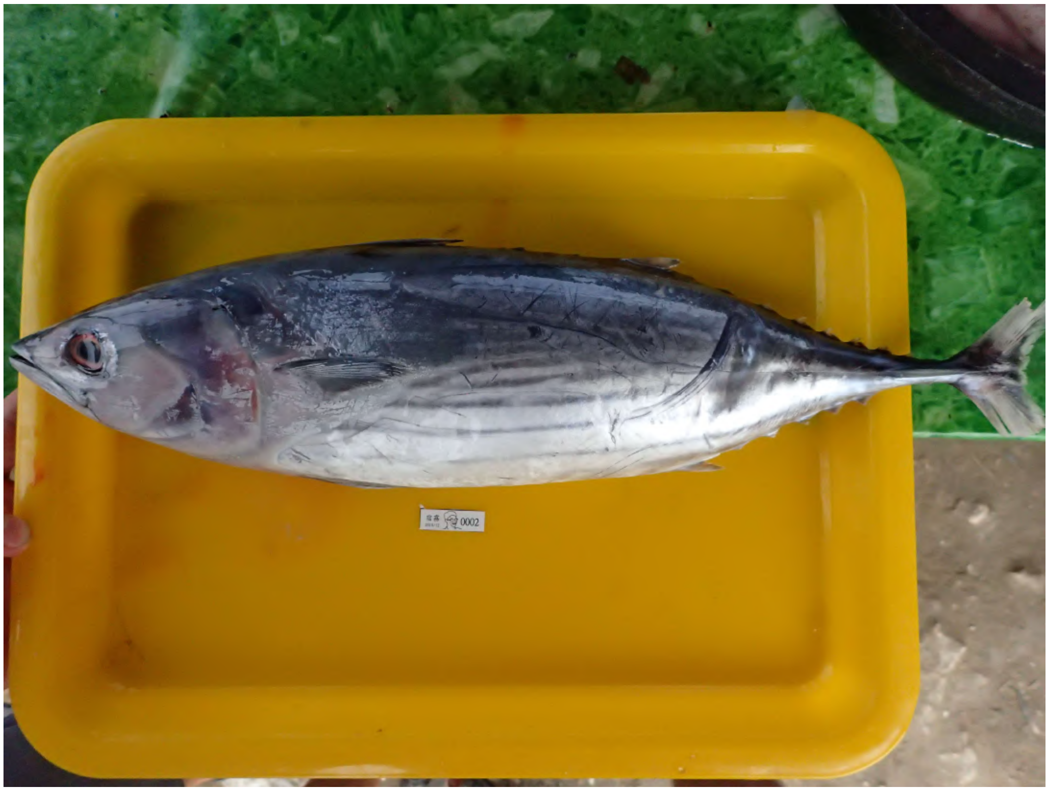

DOS 06926-1, *Katsuwonus pelamis*, OR114102. (specimen not preserved)

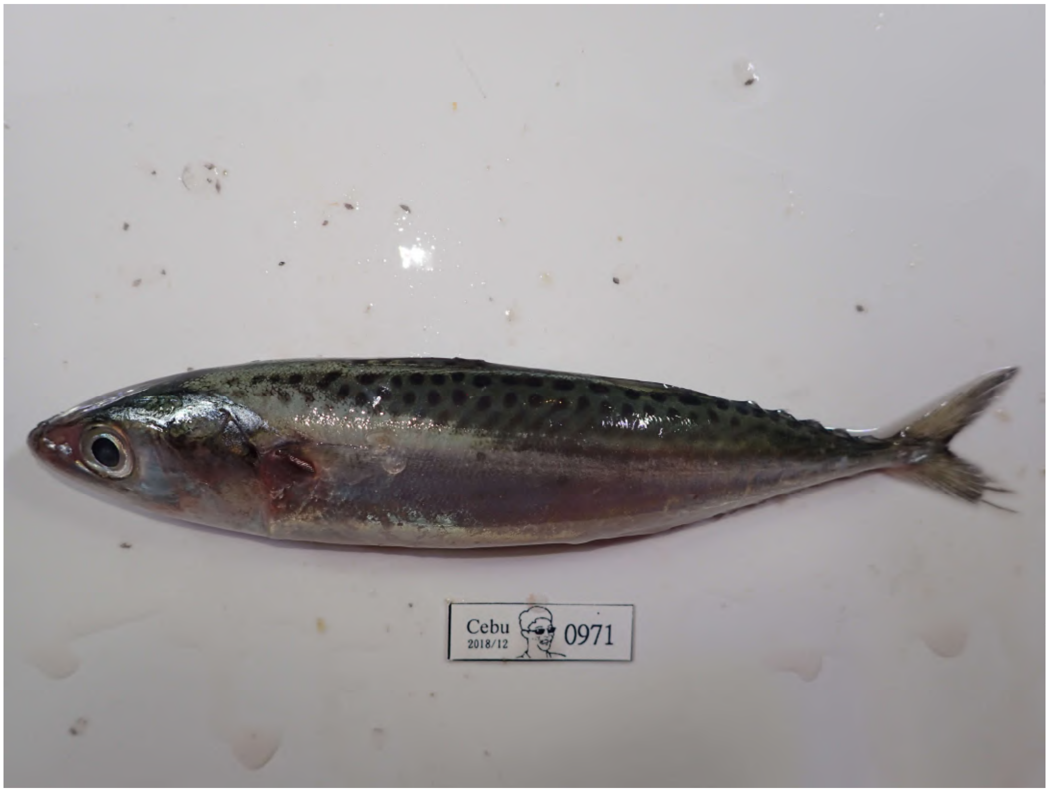

DOS 06927-4, *Rastrelliger kanagurta*, OR114103.

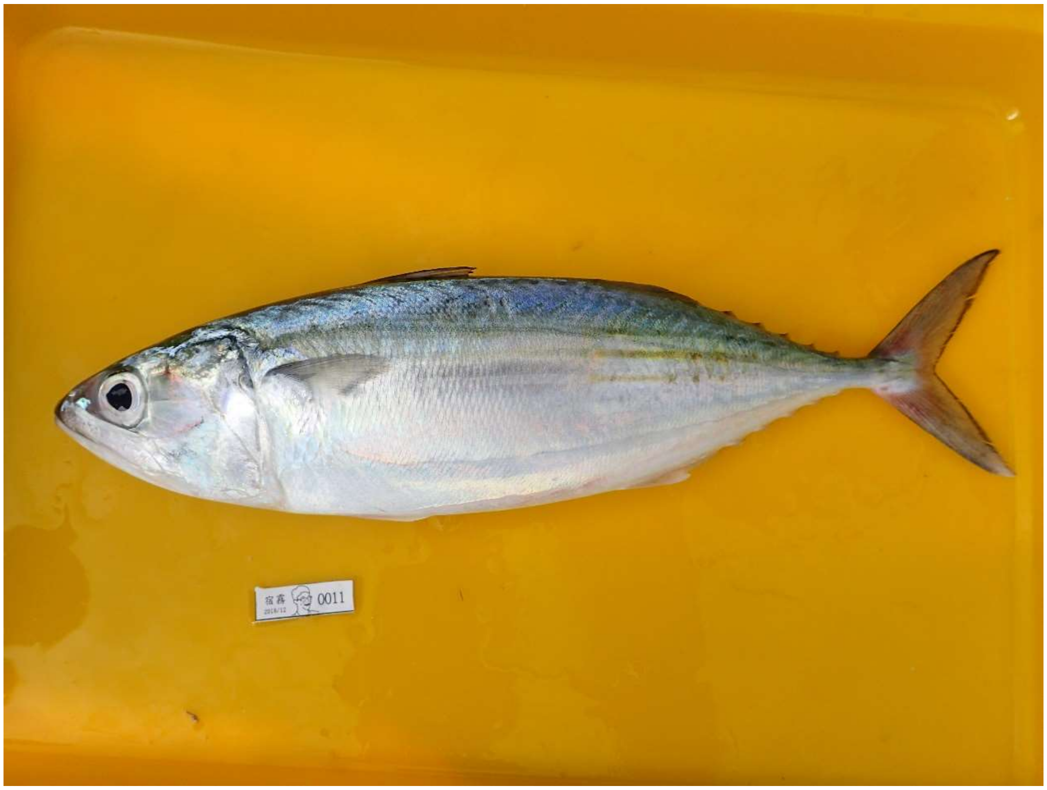

DOS 06928-1, *Rastrelliger kanagurta*, OR114104. (specimen not preserved)

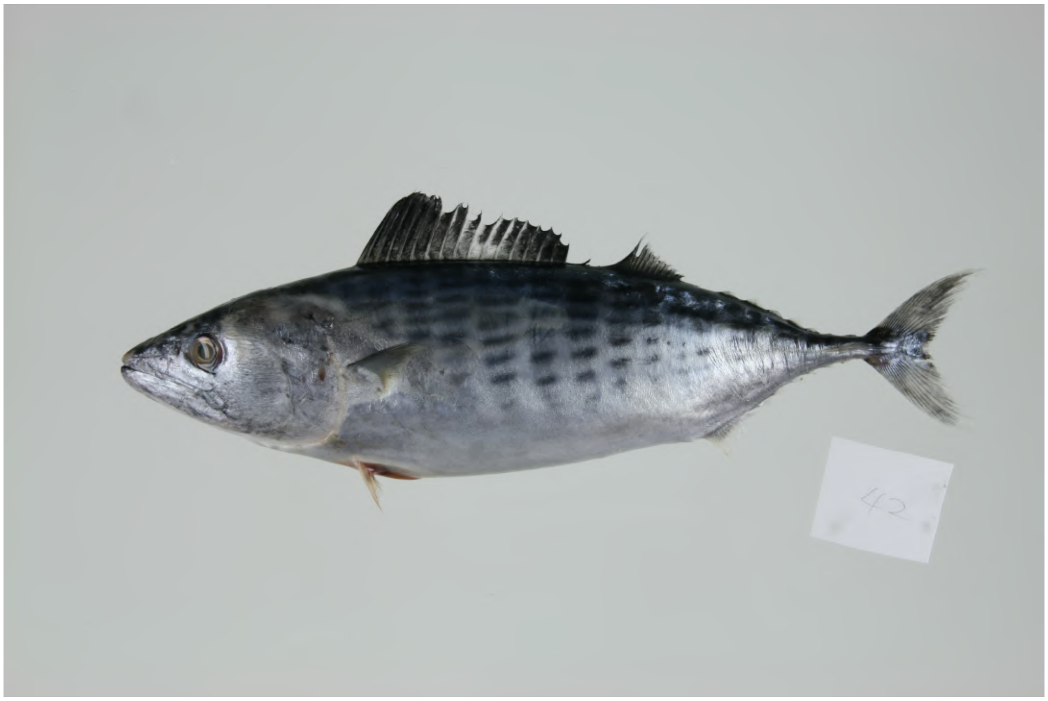

DOS 08676-1, *Sarda orientalis*, OR114257.

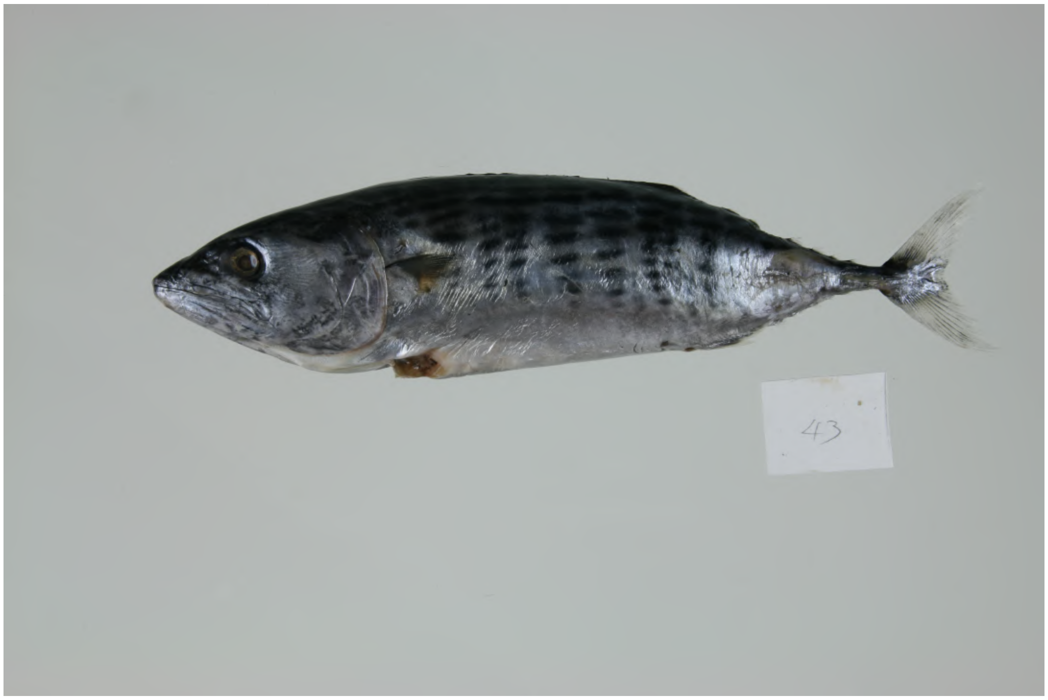

DOS 08676-2, *Sarda orientalis*, OR114258.

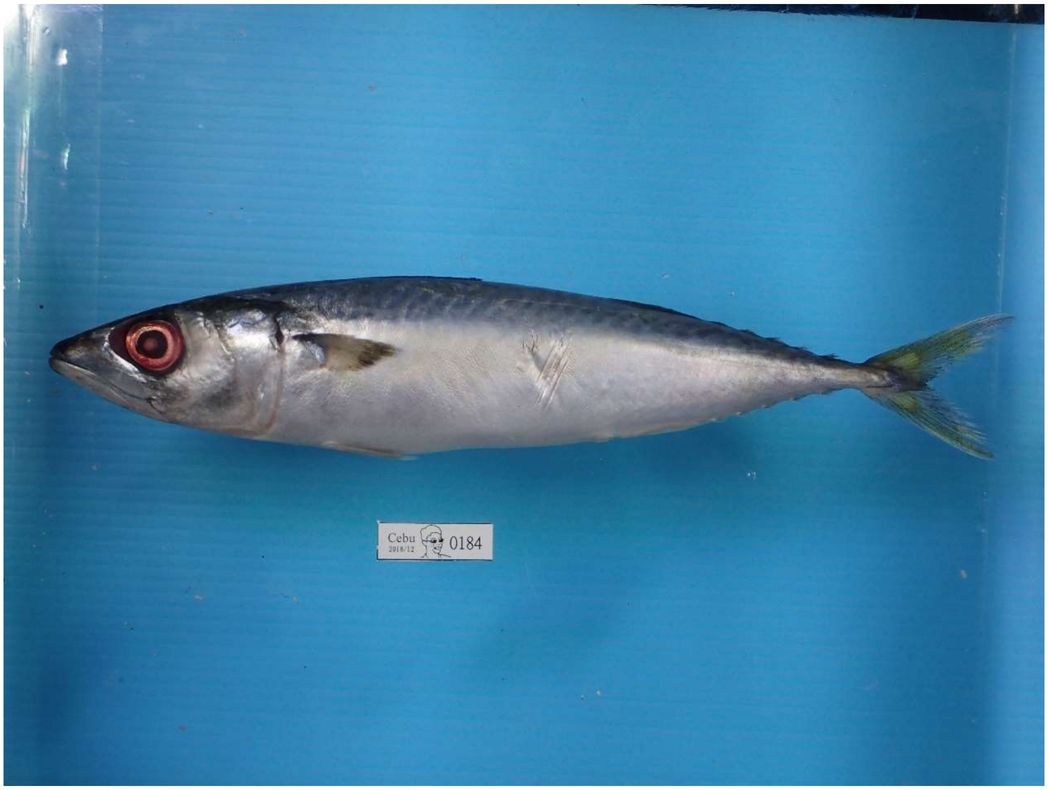

DOS 06929-1, *Scomber japonicus*, OR114105.

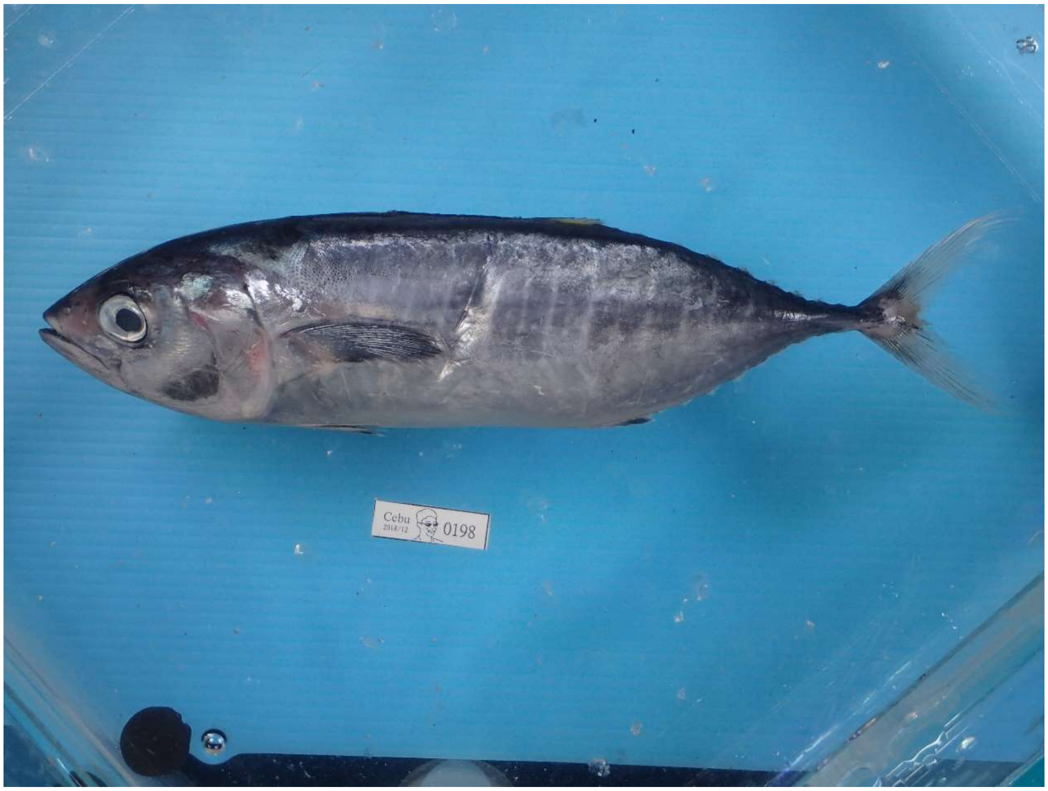

DOS 06931-1, *Thunnus albacares*, OR114107. (specimen not preserved)

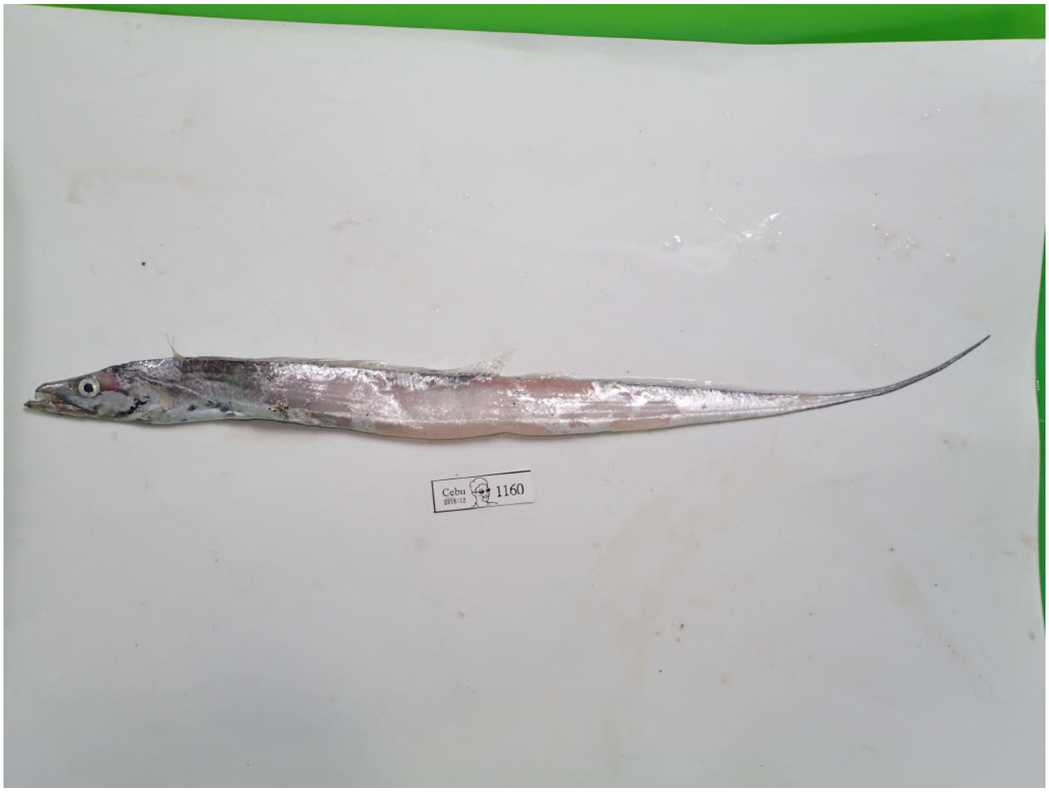

DOS 06991-1, *Trichiurus nanhaiensis*, OR114160.

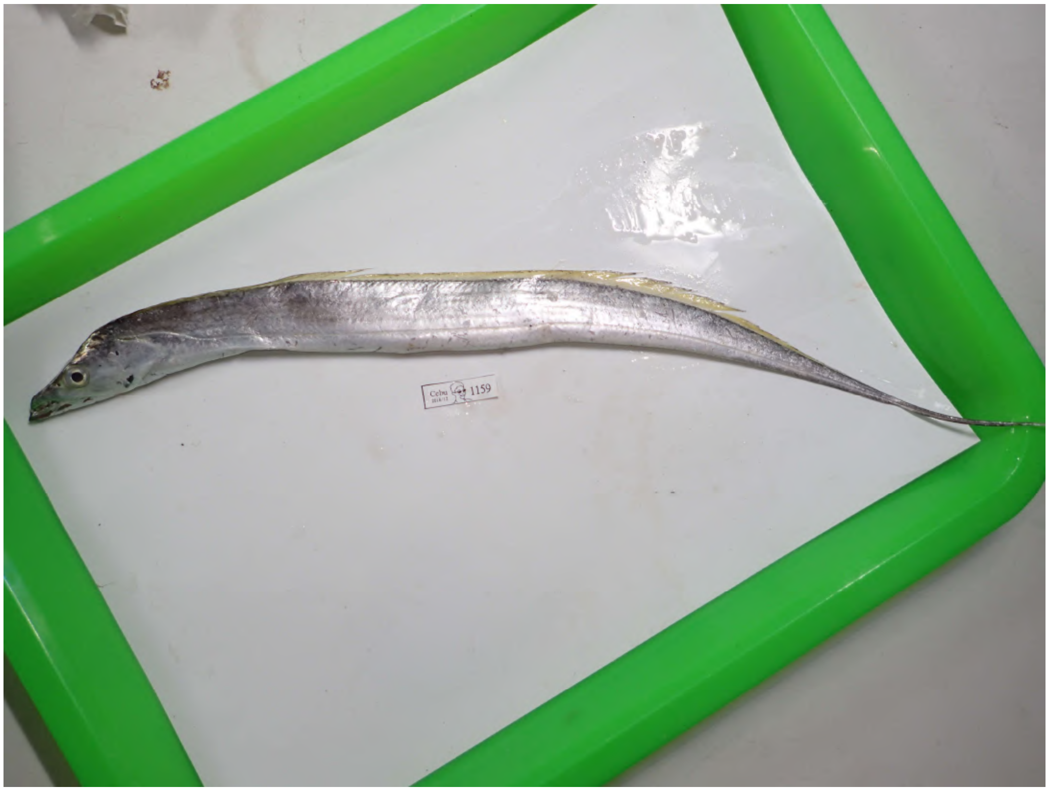

DOS 06992-3, *Trichiurus nanhaiensis*, OR114161.

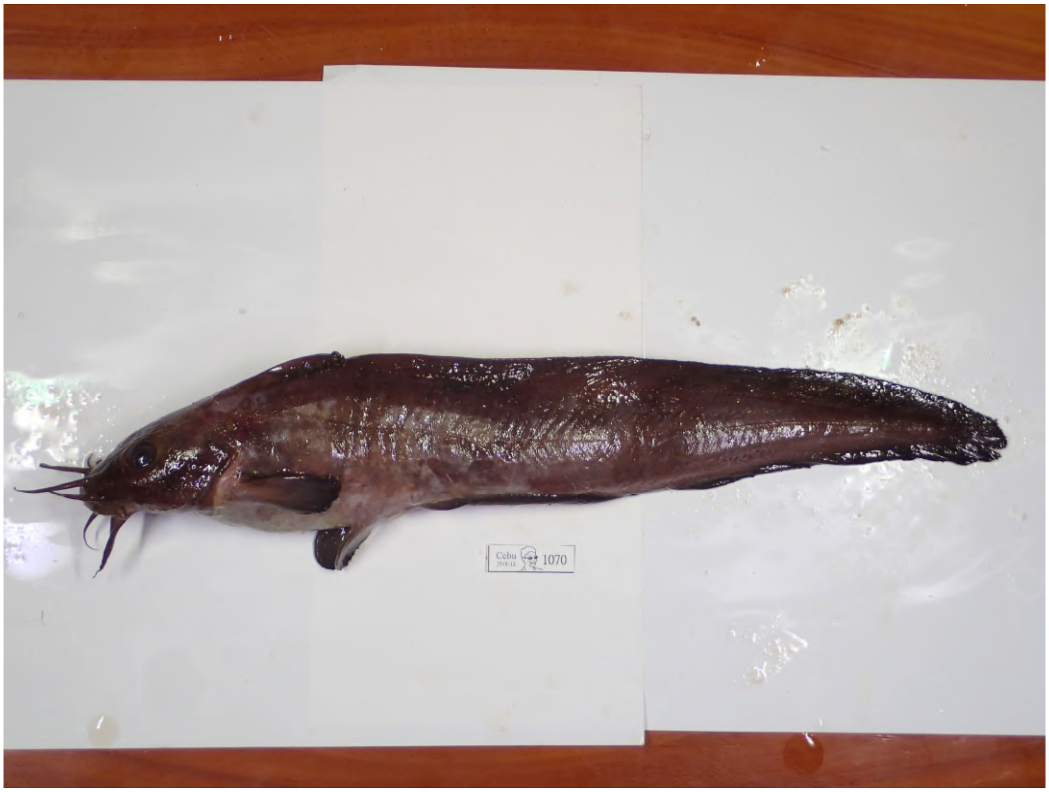

DOS 06873, *Paraplotosus* sp., OR114050.

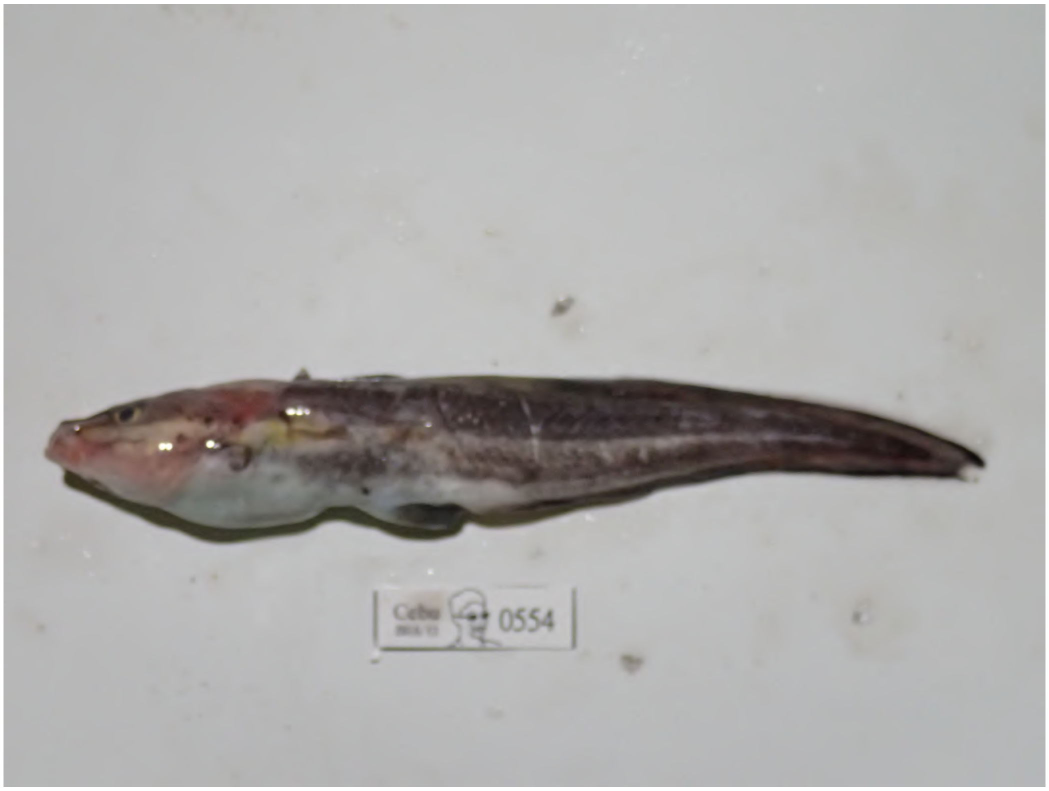

DOS 06871-1, *Plotosus lineatus*, OR114046.

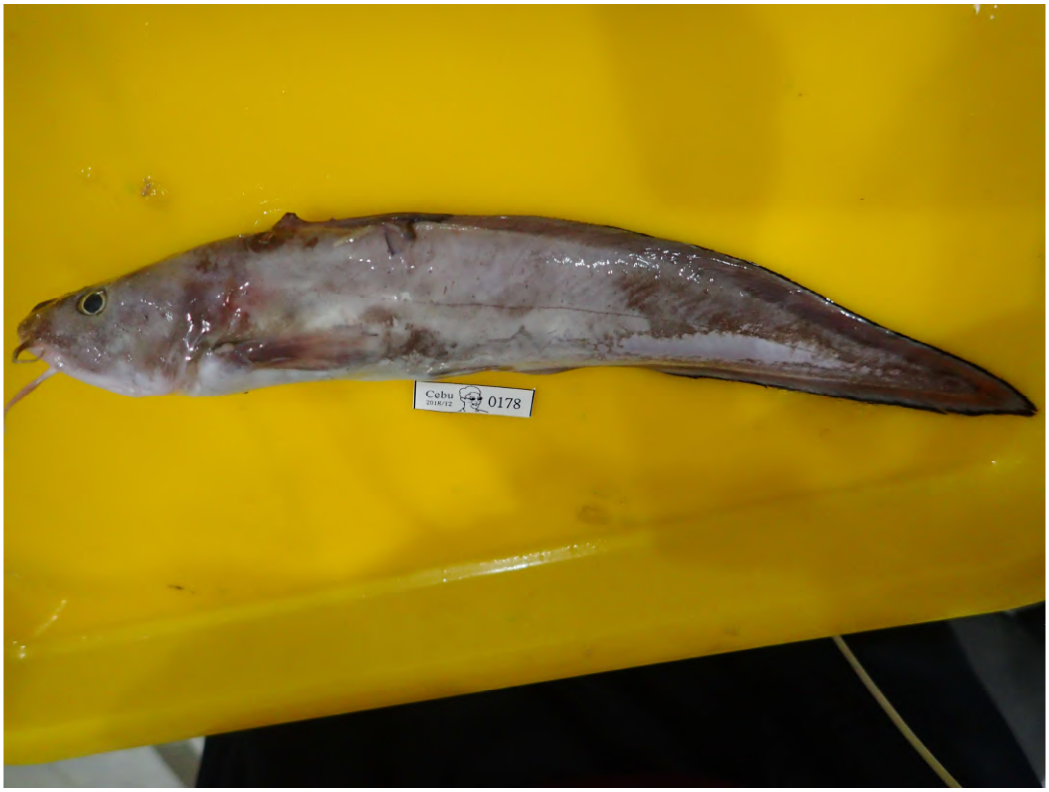

DOS 06872-1, *Plotosus lineatus*, OR114047.

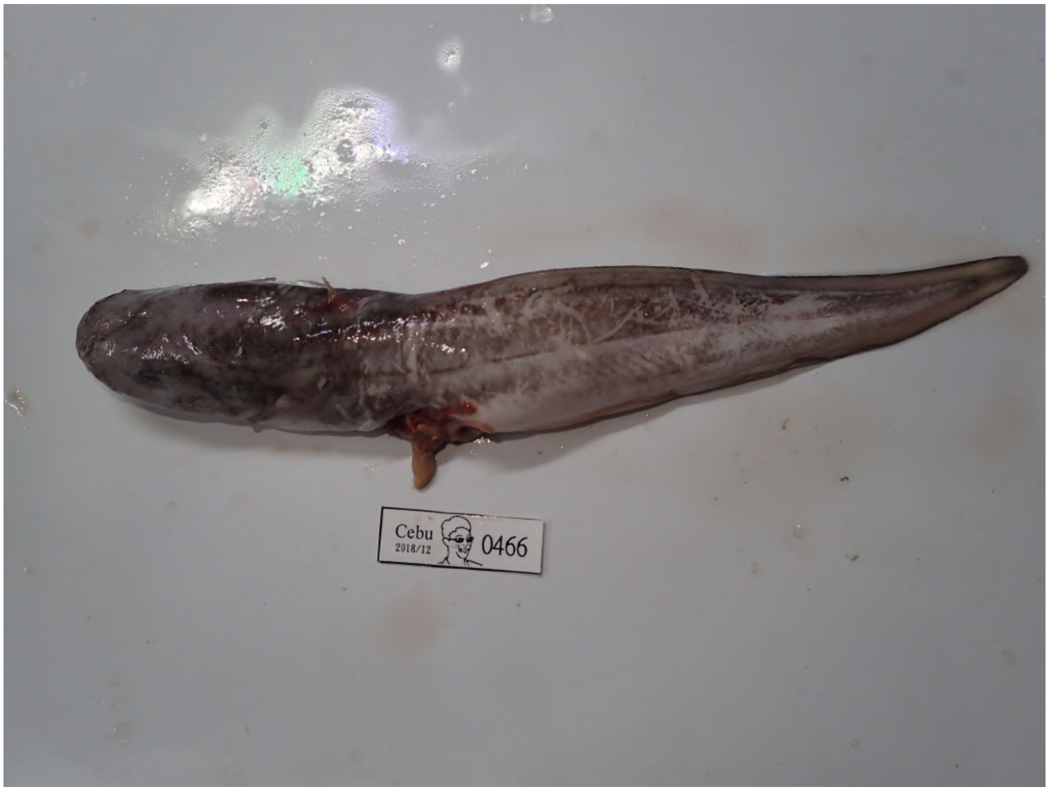

DOS 06872-4, *Plotosus lineatus*, OR114048.

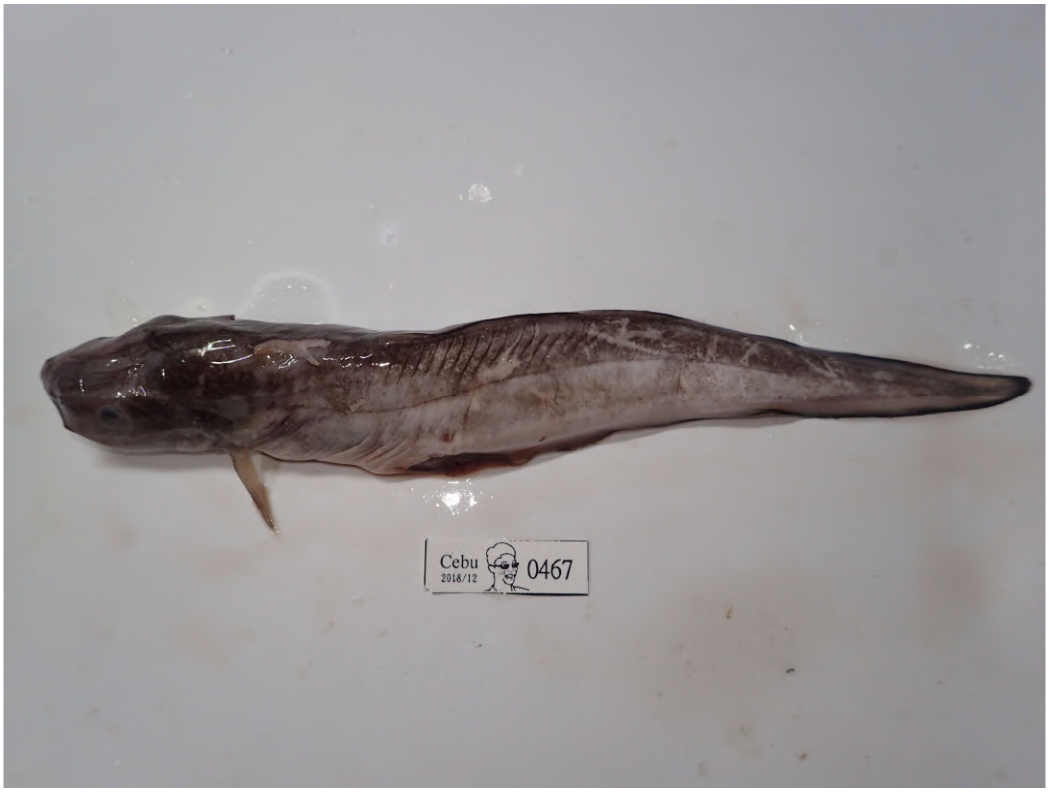

DOS 06872-5, *Plotosus lineatus*, OR114049.

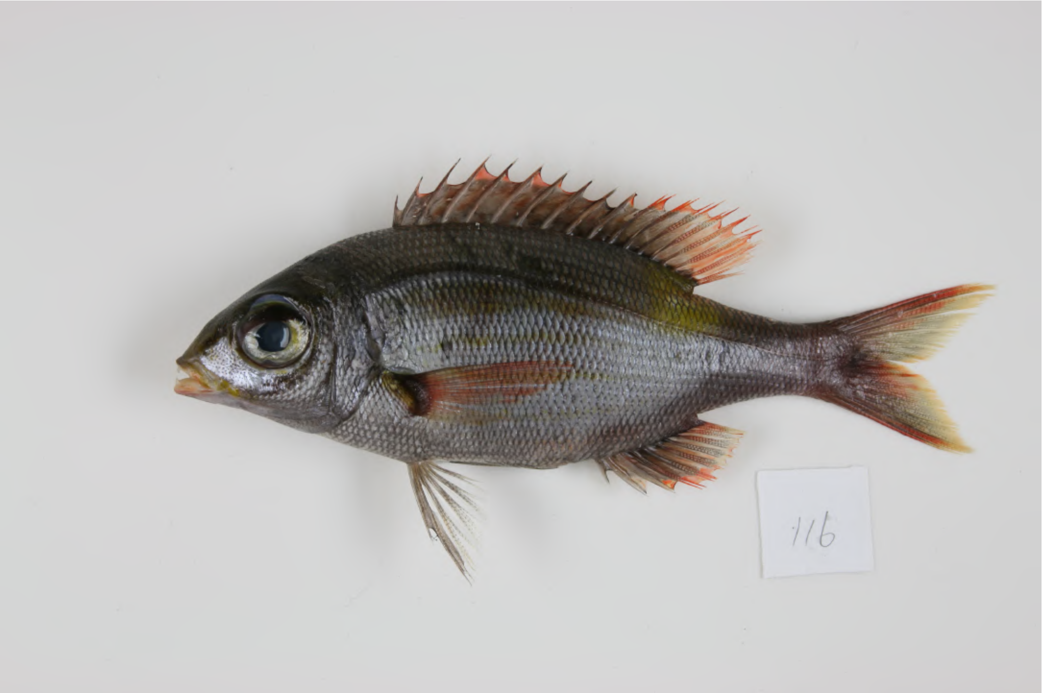

DOS 08680, *Gnathodentex aureolineatus*, OR114262.

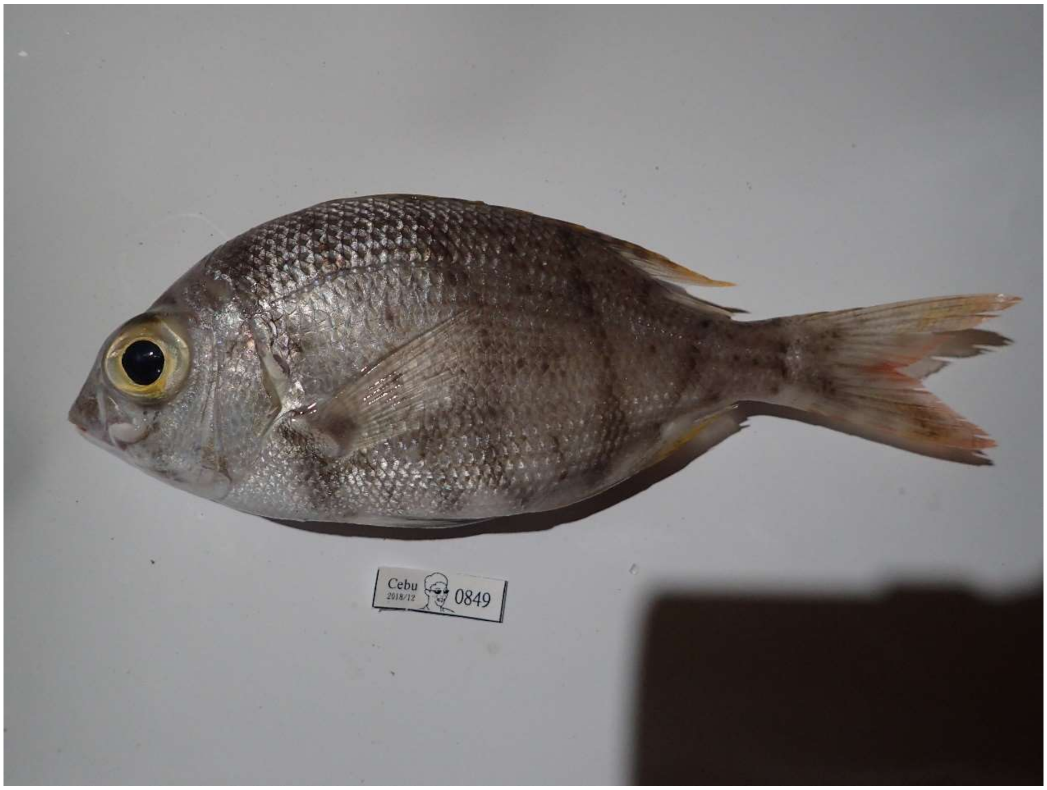

DOS 06770-1, *Gymnocranius griseus*, OR113953.

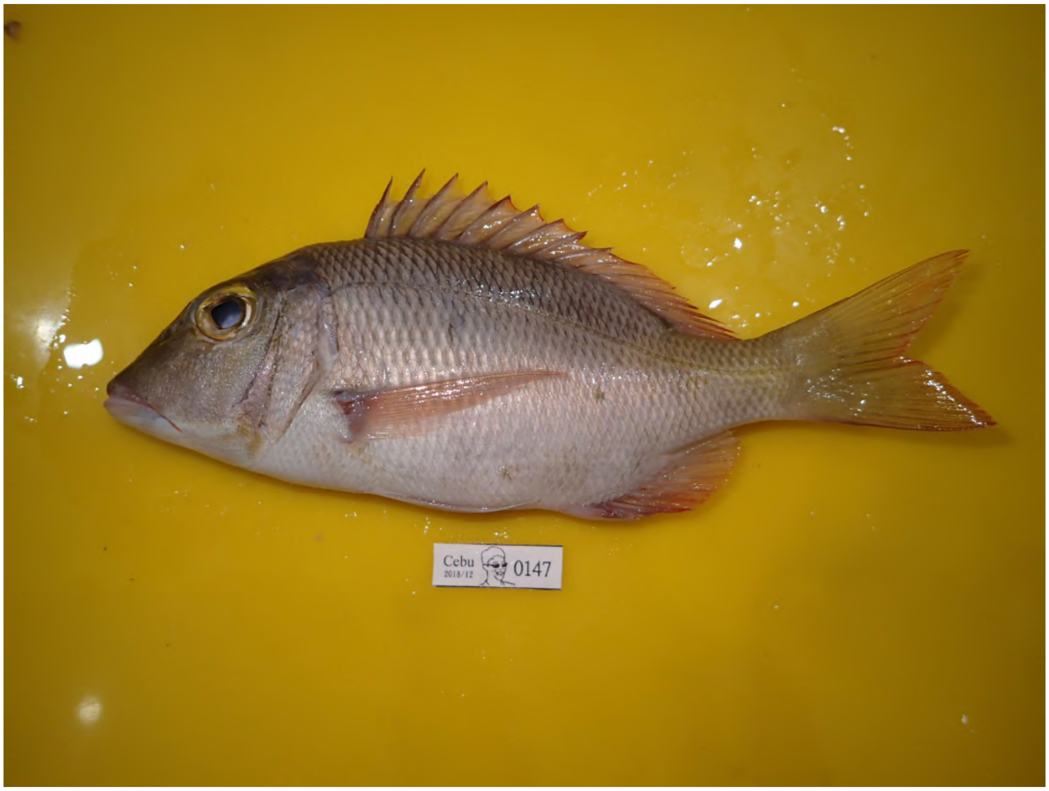

DOS 06774-1, *Lethrinus atkinsoni*, OR113956. (specimen not preserved)

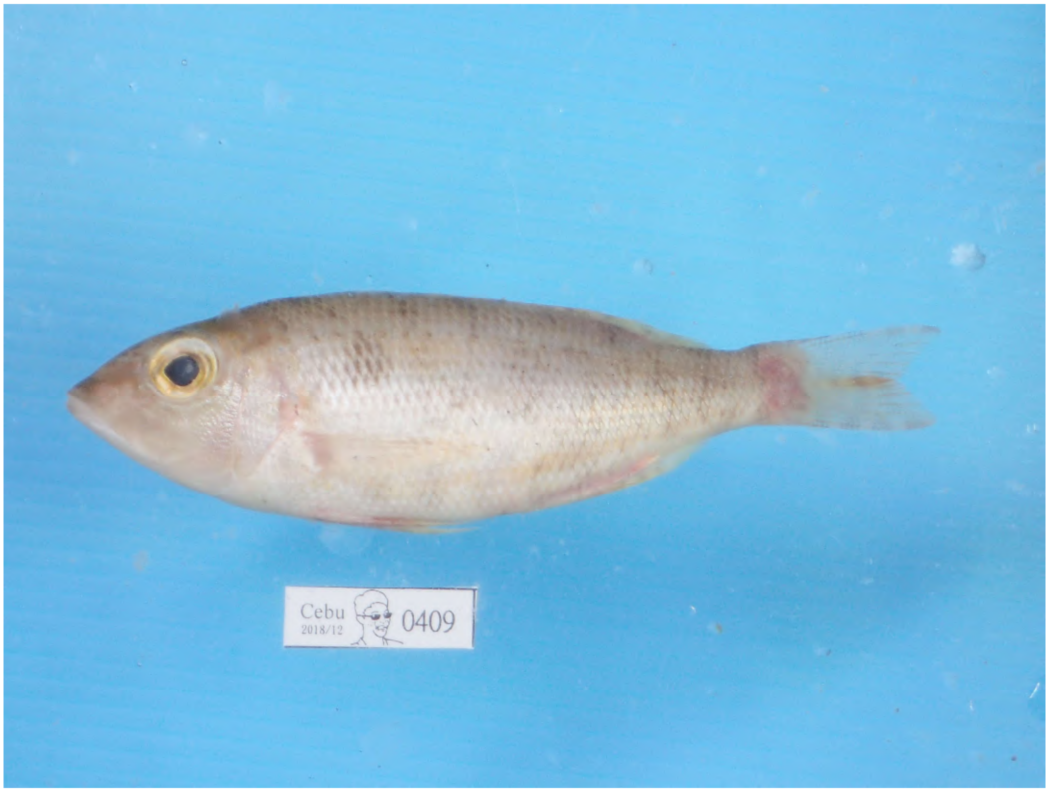

DOS 06778-2, *Lethrinus genivittatus*, OR113959.

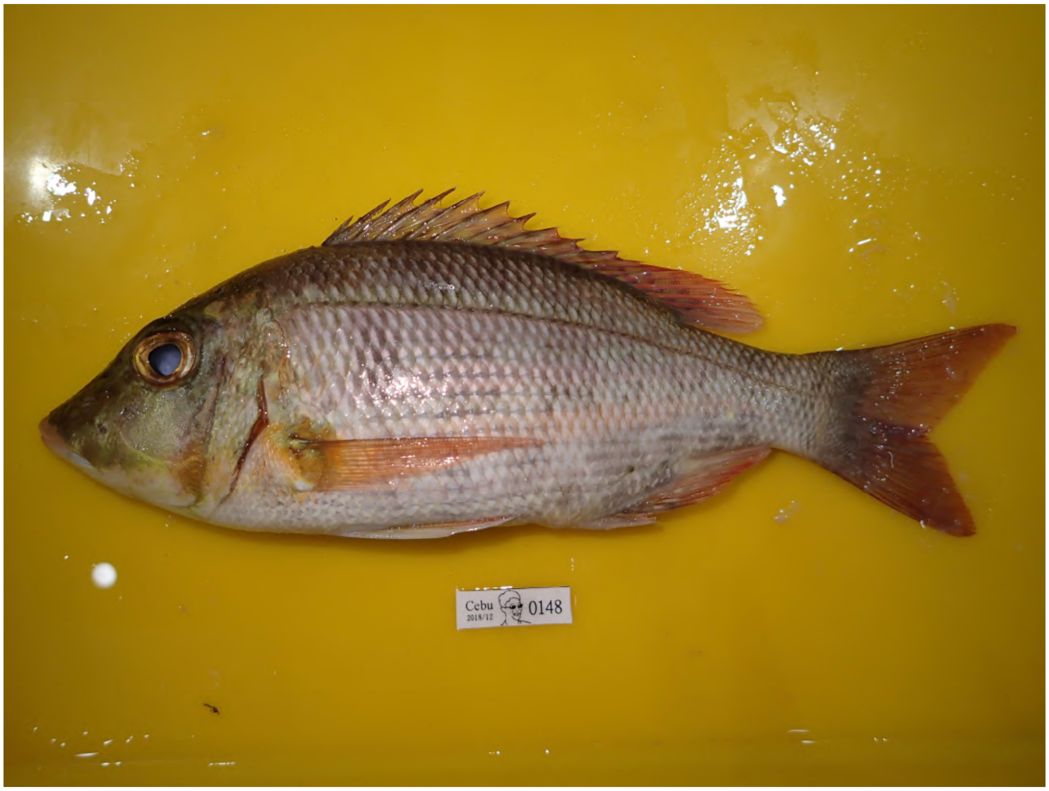

DOS 06771, *Lethrinus obsoletus*, OR113954.

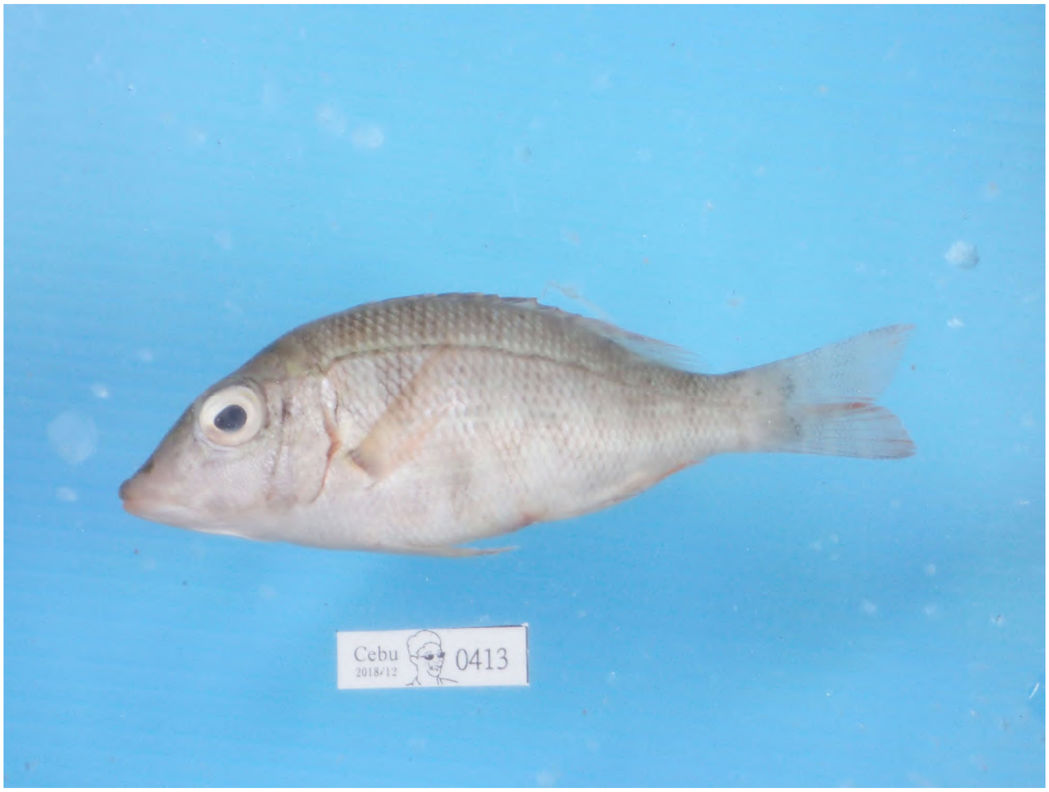

DOS 06773, *Lethrinus obsoletus*, OR113955. (specimen not preserved)

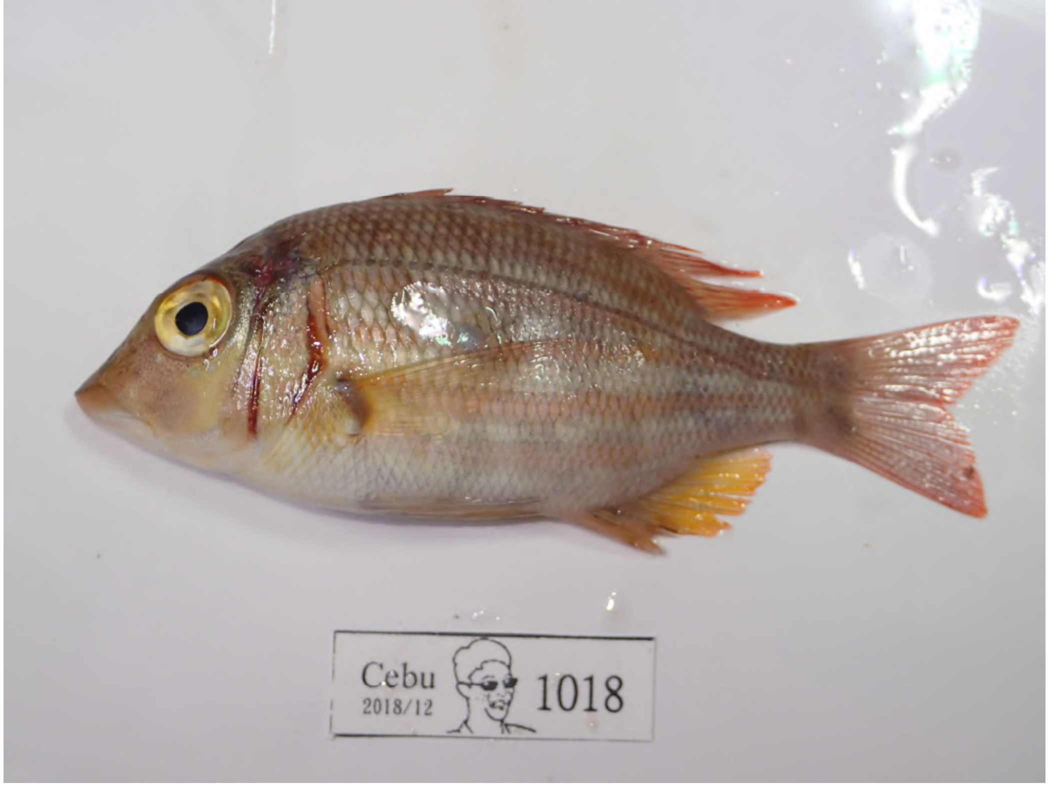

DOS 06776, *Lethrinus ornatus*, OR113957.

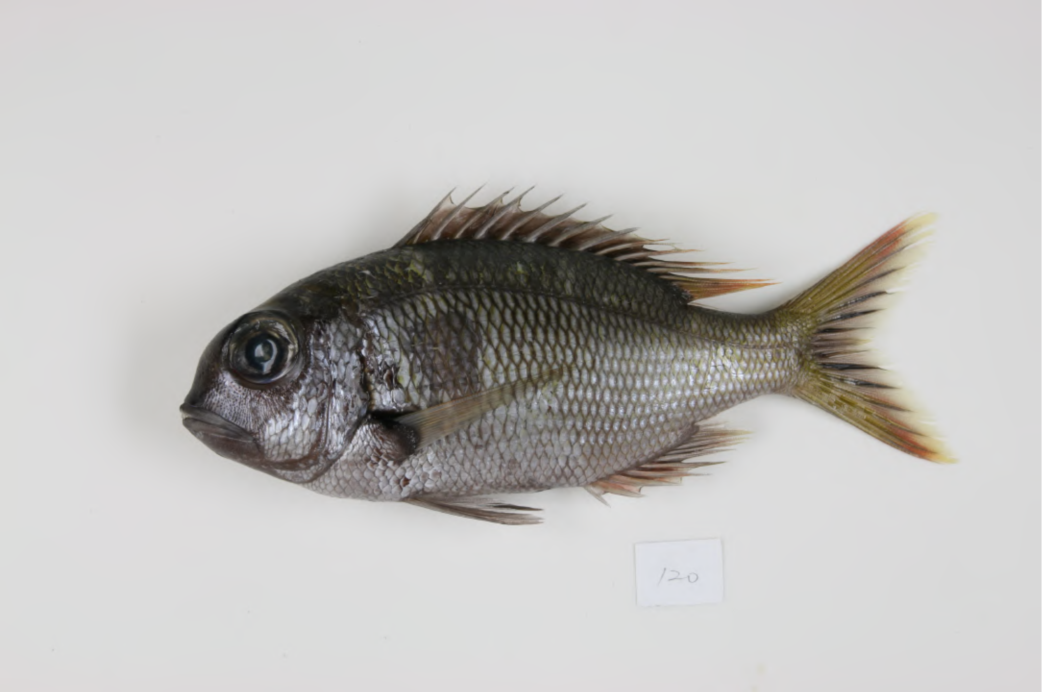

DOS 08681, *Monotaxis heterodon*, OR114263.

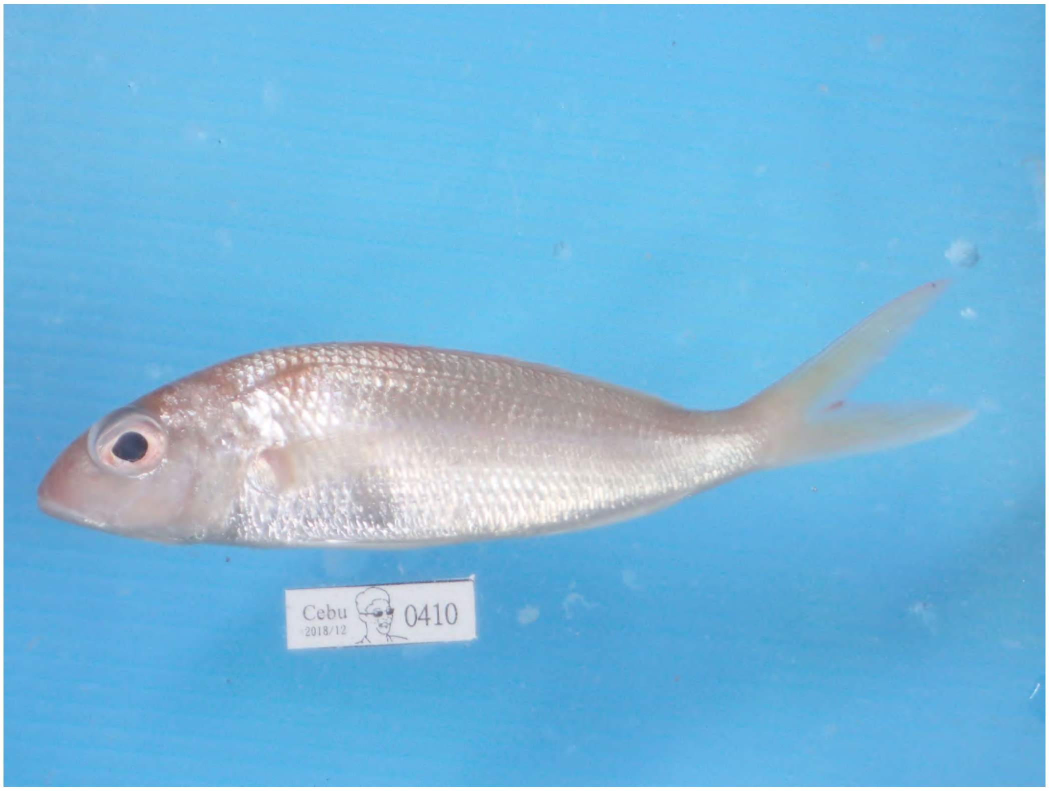

DOS 06836, *Nemipterus furcosus*, OR114012.

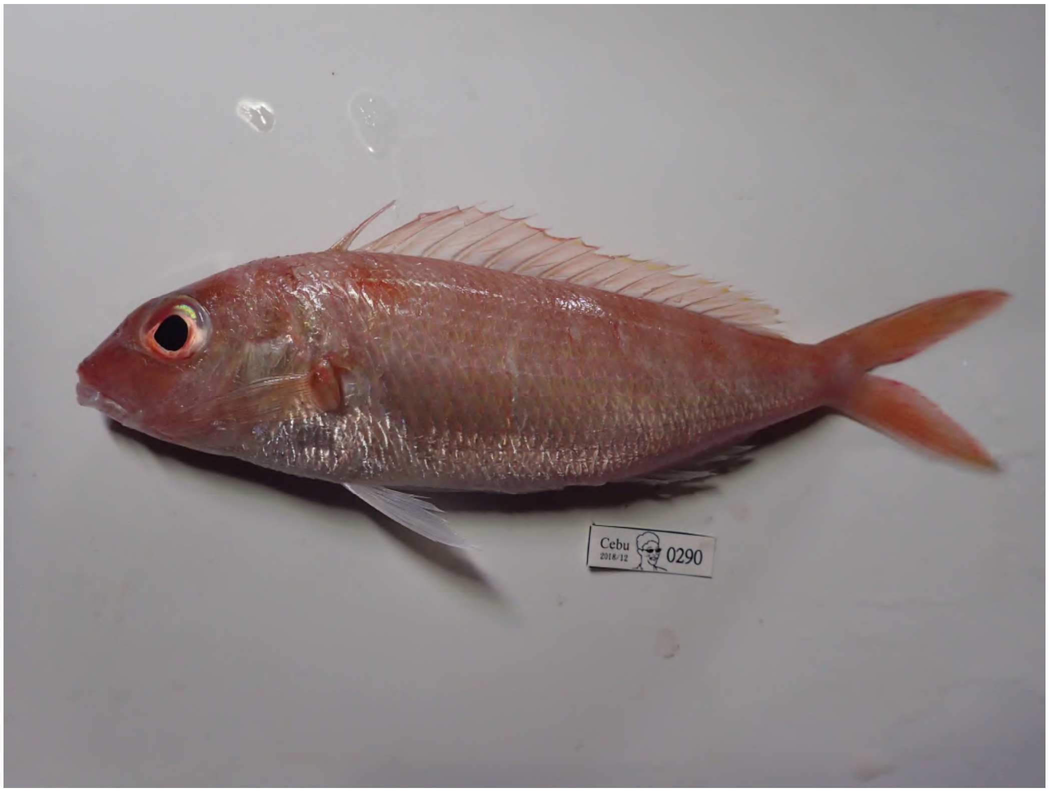

DOS 06838, *Nemipterus furcosus*, OR114014.

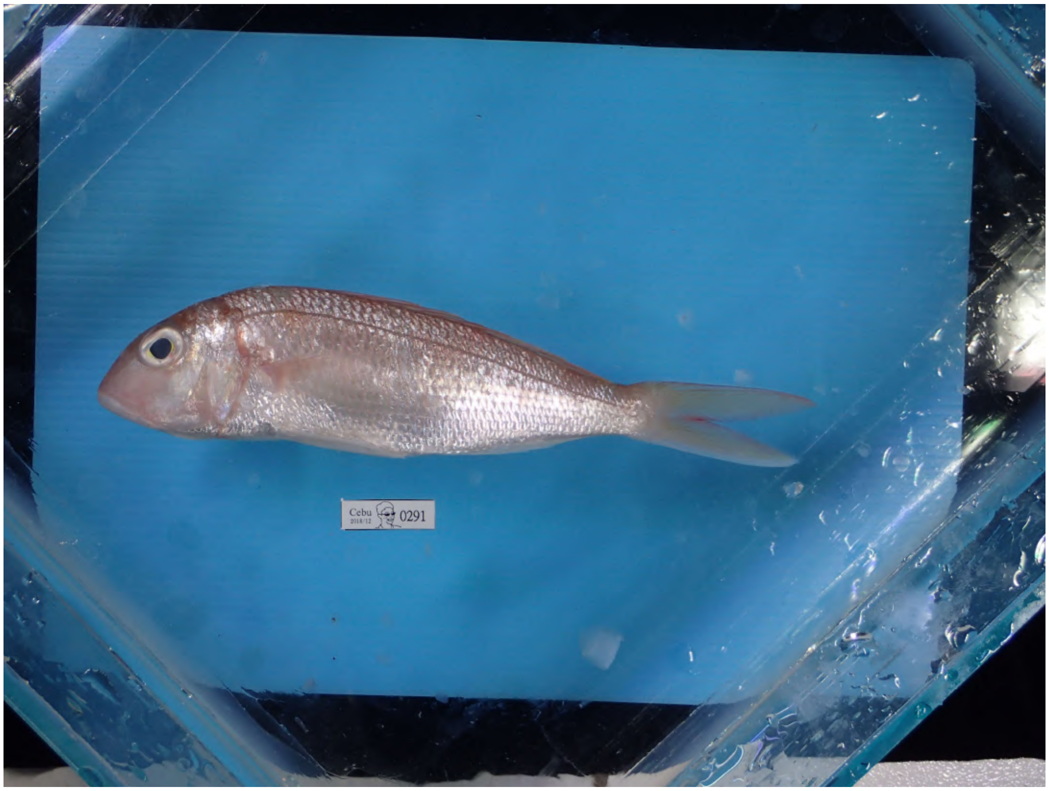

DOS 06839-1, *Nemipterus furcosus*, OR114015. (specimen not preserved)

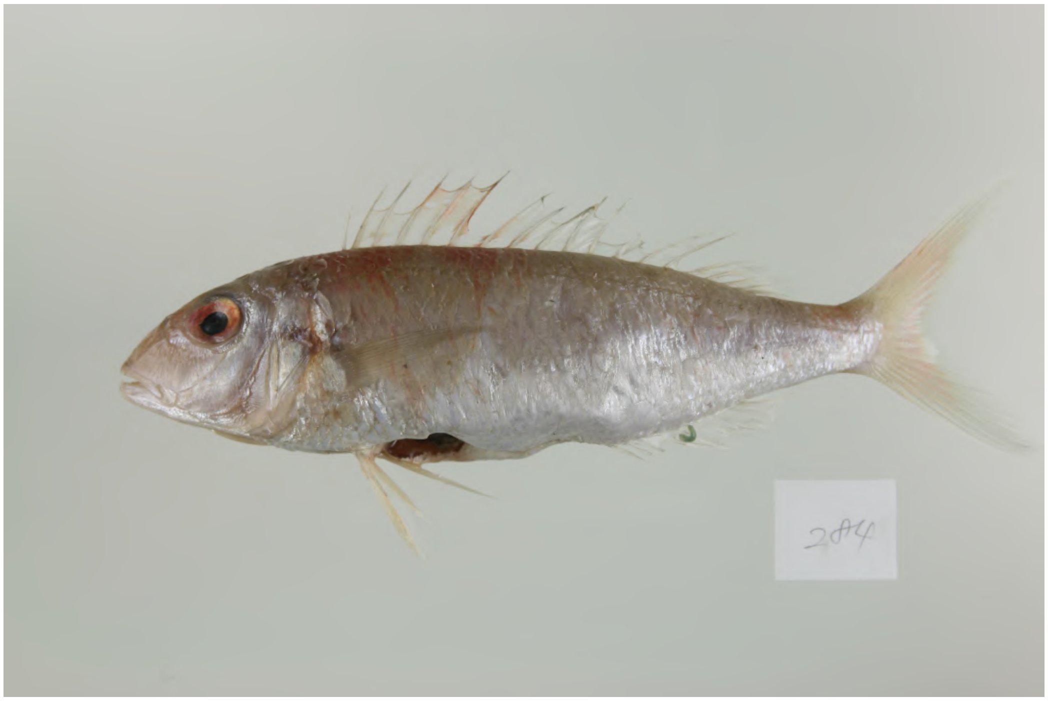

DOS 08682, *Nemipterus furcosus*, OR114264.

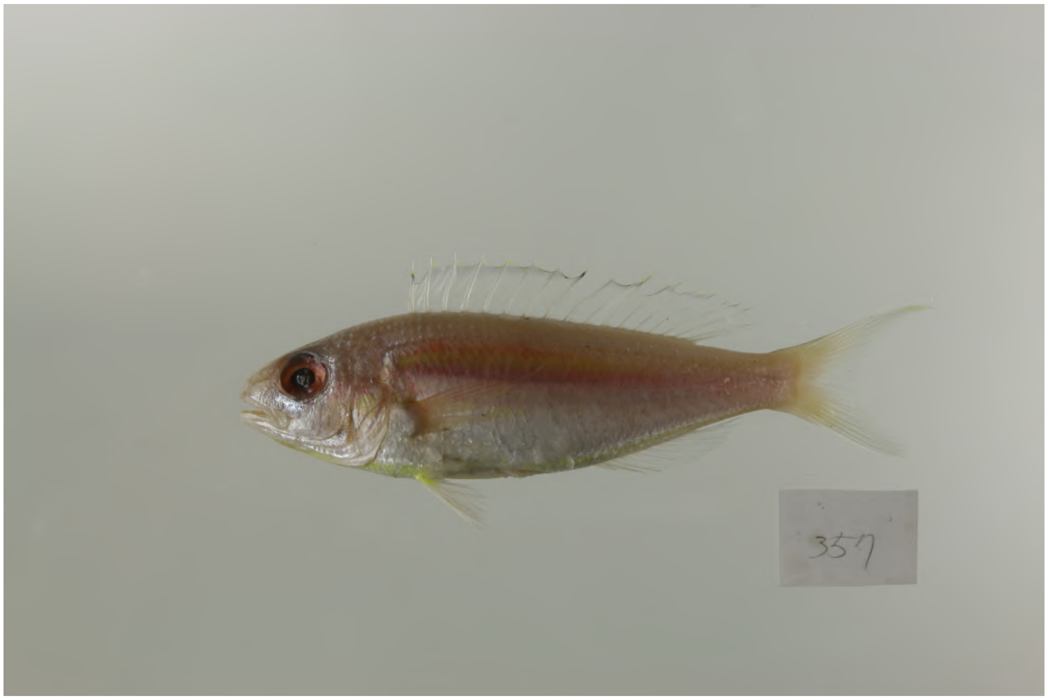

DOS 08683, *Nemipterus* sp., OR114265.

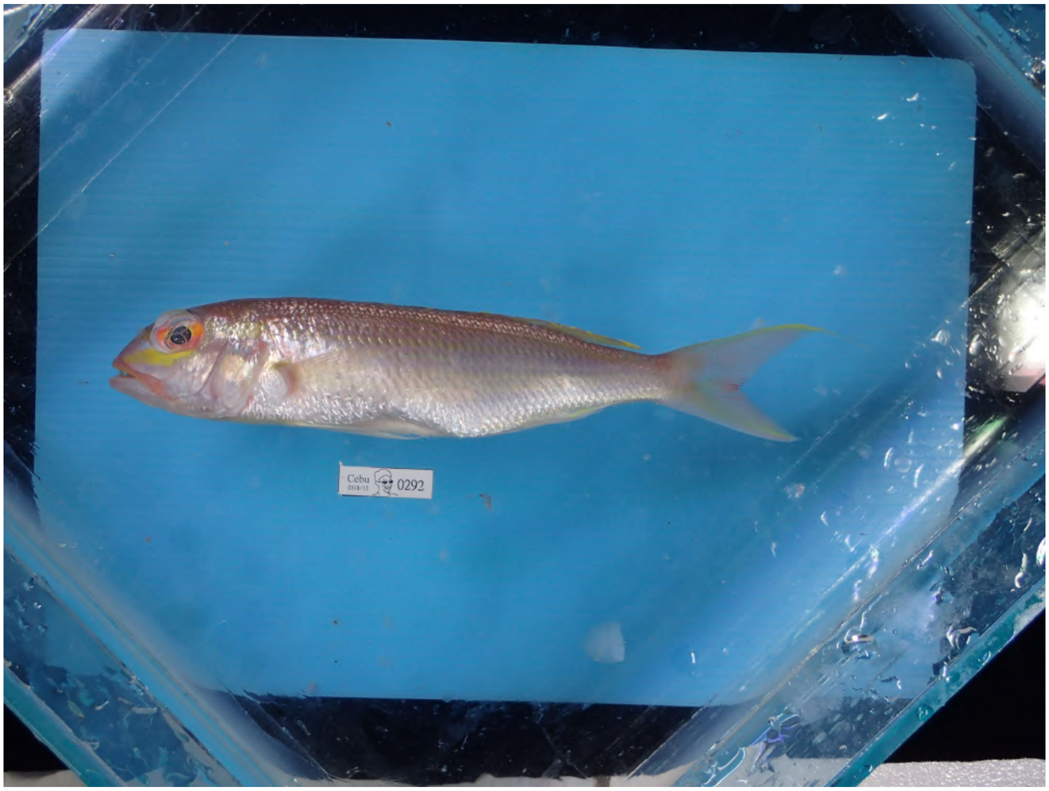

DOS 06840, *Nemipterus zysron*, OR114016. (specimen not preserved)

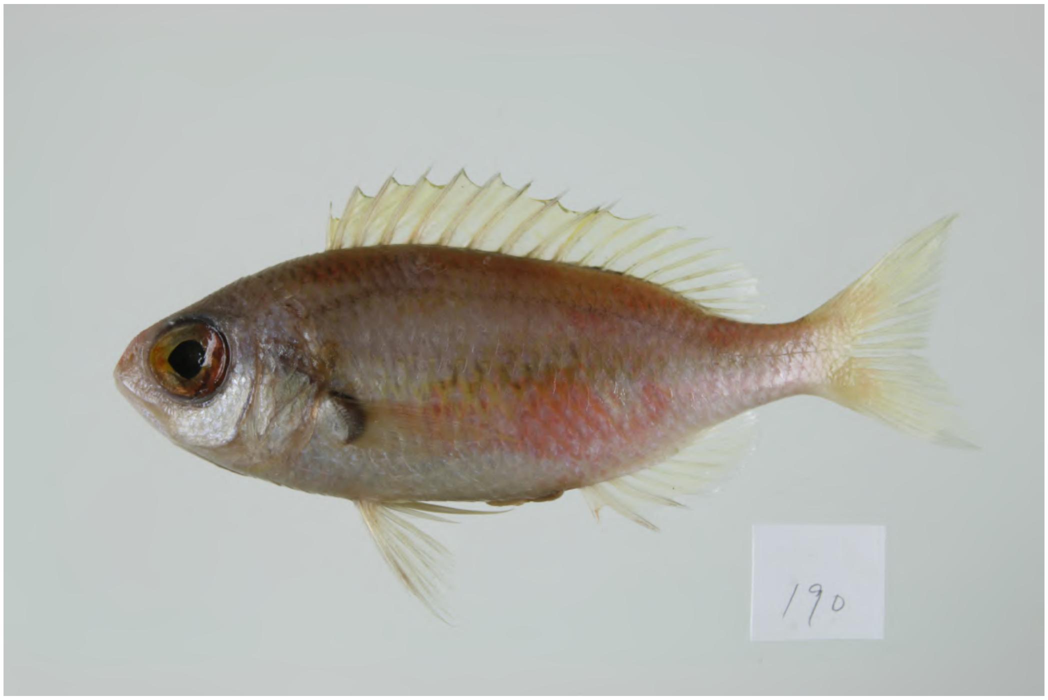

DOS 08684, *Parascolopsis akatamae*, OR114266.

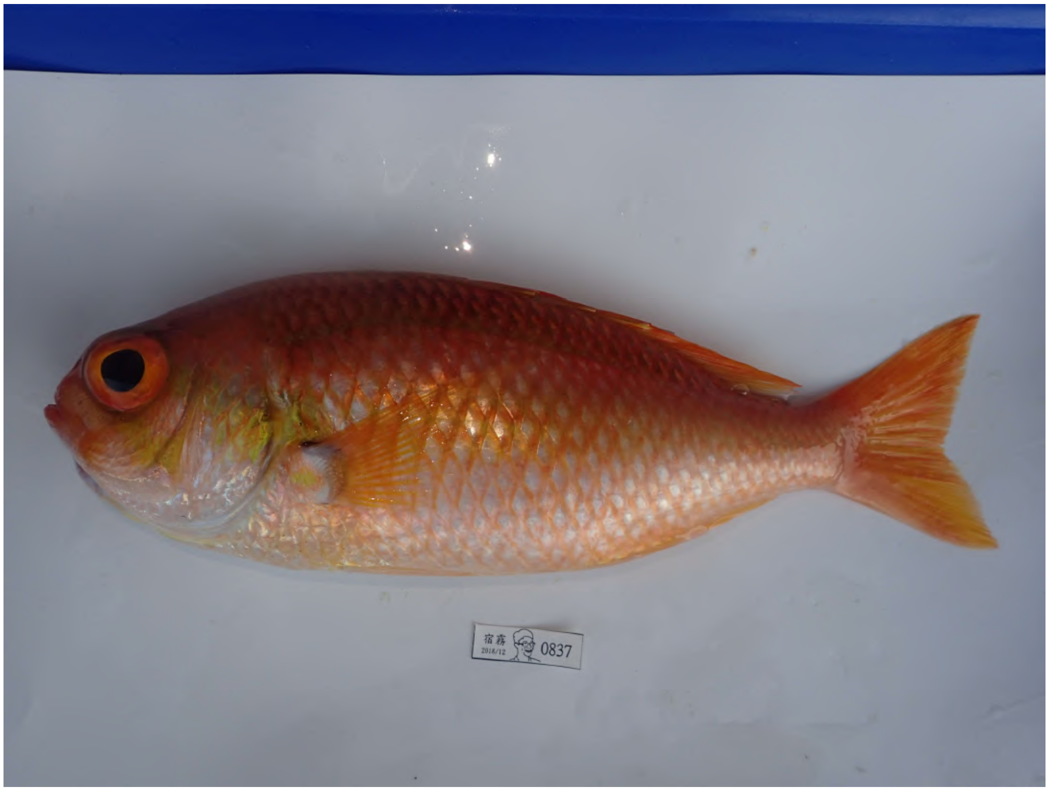

DOS 06843, *Parascolopsis eriomma*, OR114018. (specimen not preserved)

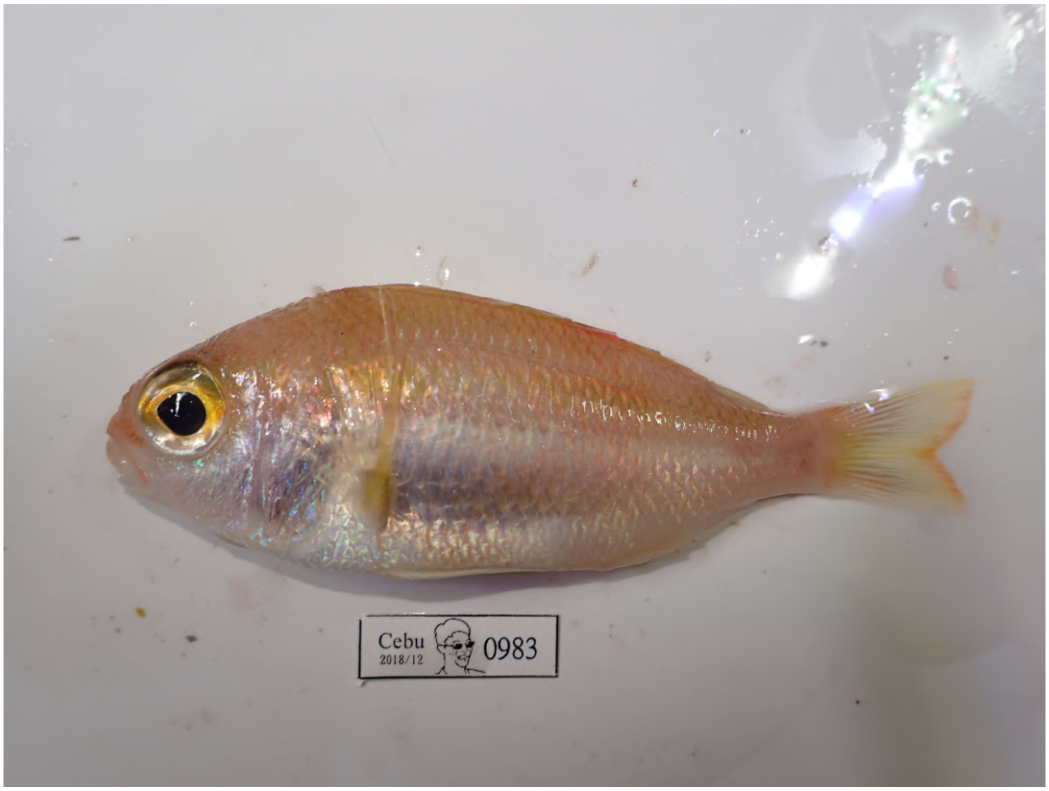

DOS 06844-4, *Parascolopsis rufomaculata*, OR114019.

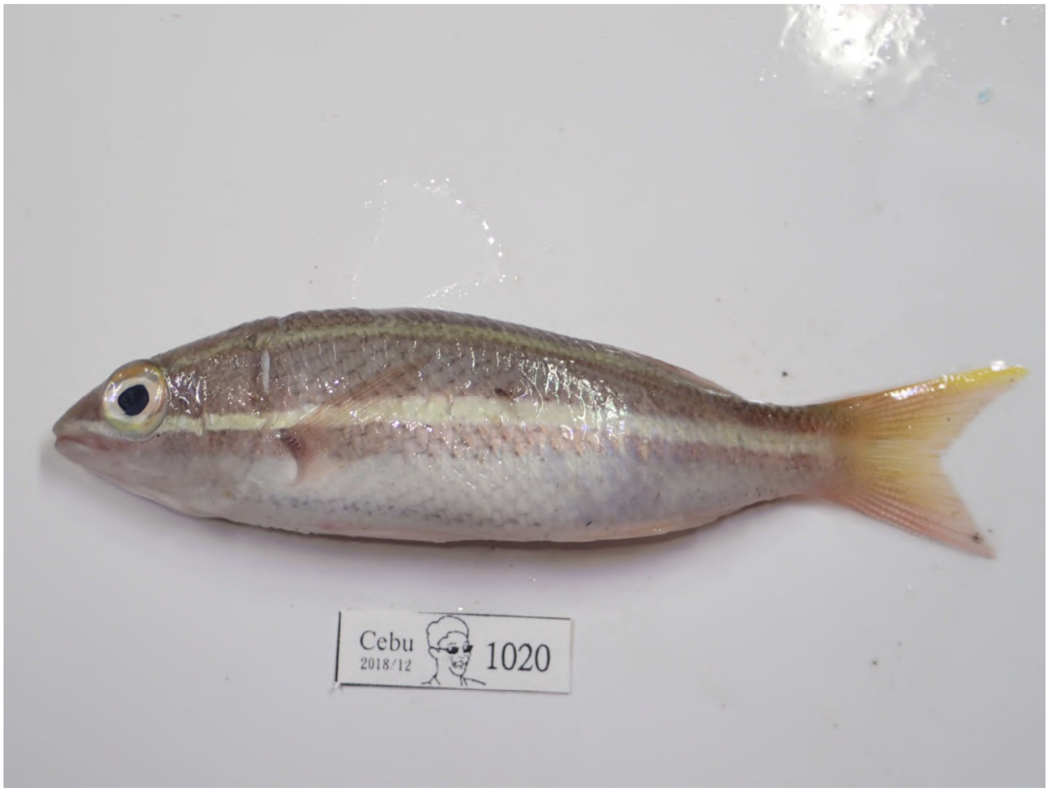

DOS 06848, *Pentapodus bifasciatus*, OR114023.

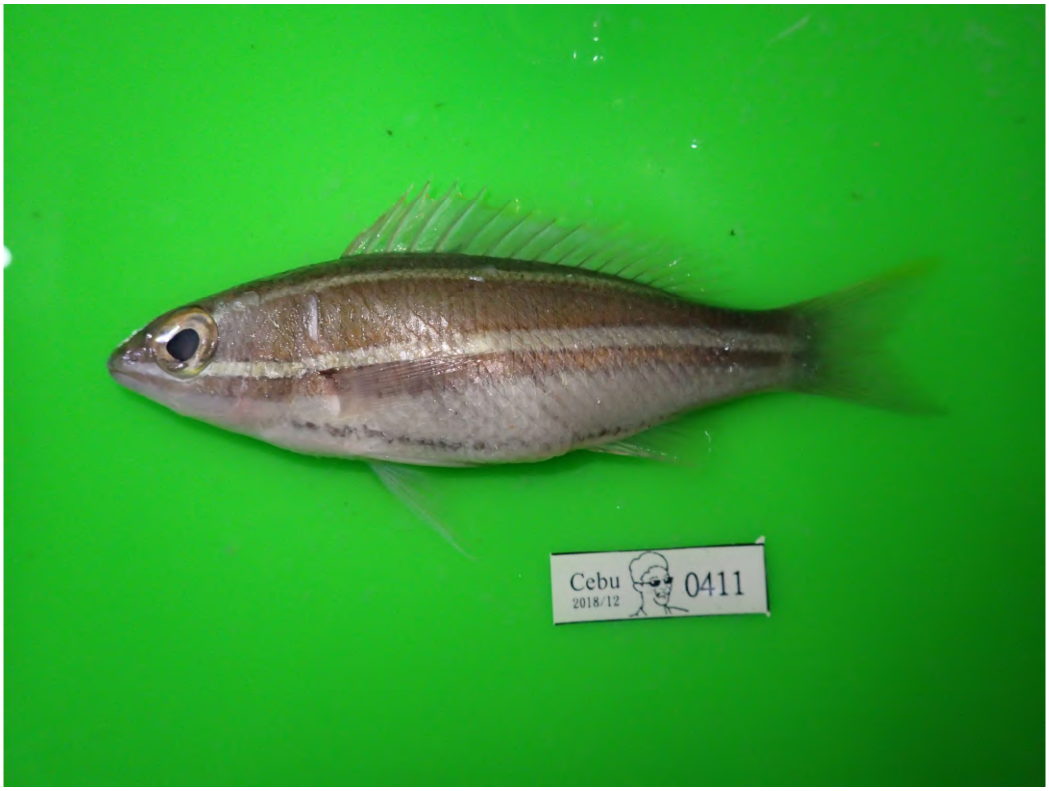

DOS 06849, *Pentapodus bifasciatus*, OR114024.

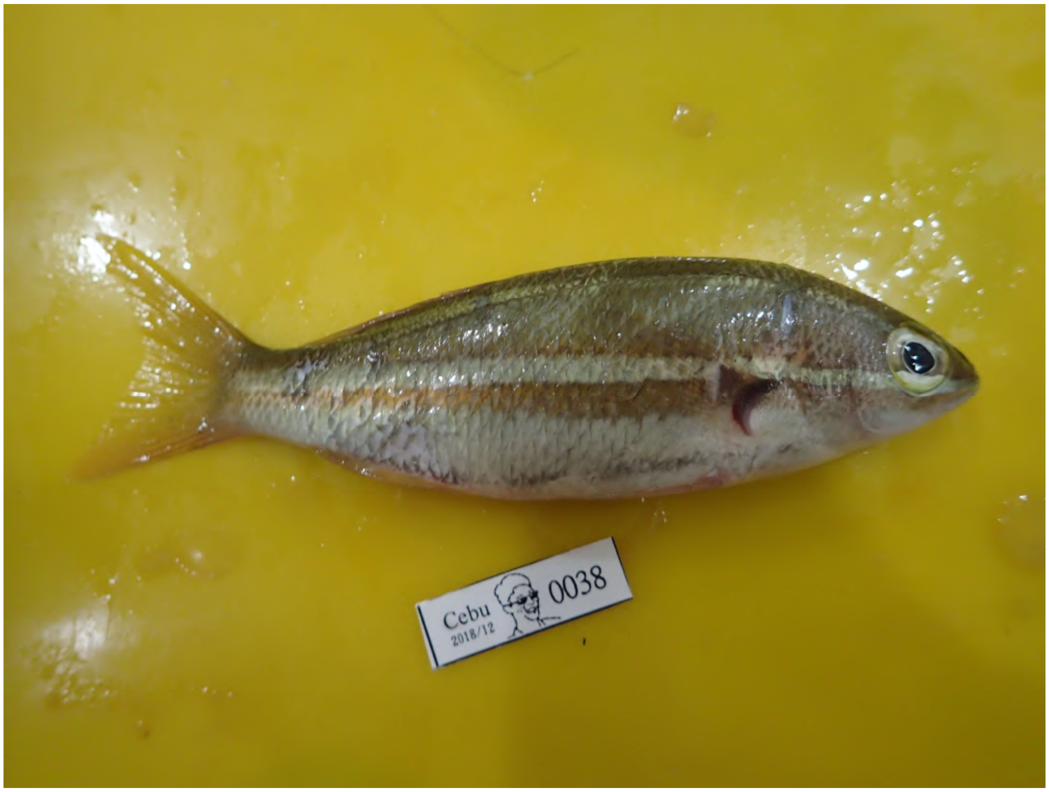

DOS 06854, *Pentapodus bifasciatus*, OR114030. (specimen not preserved)

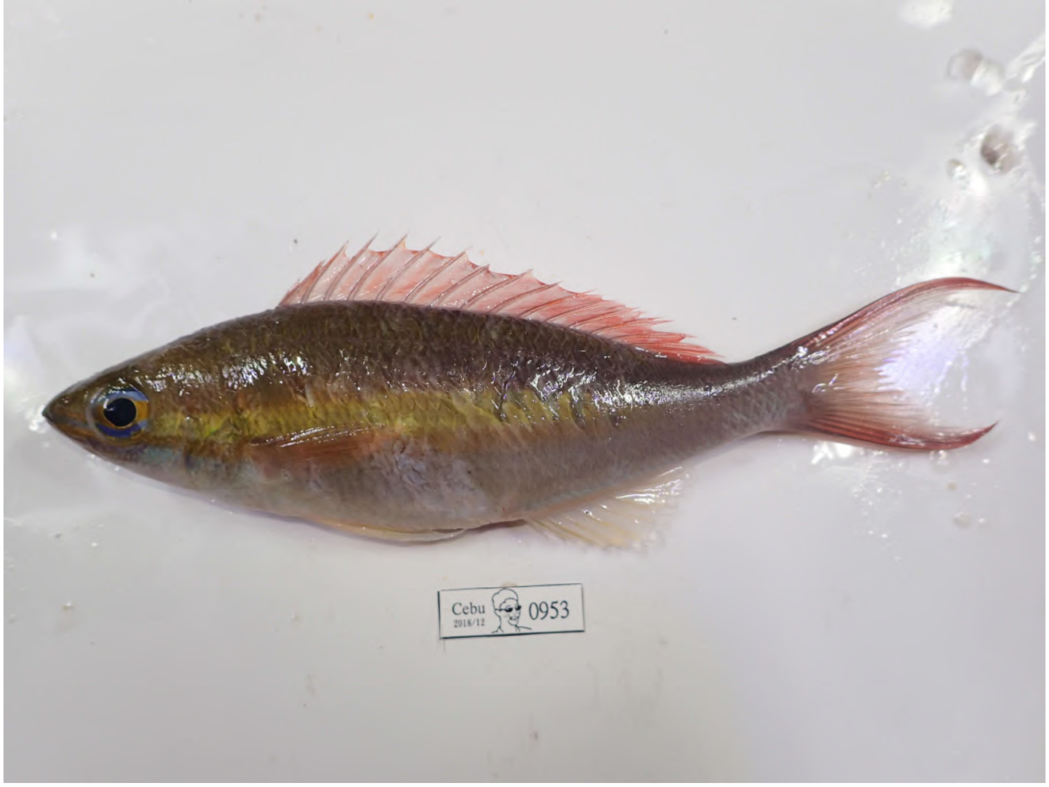

DOS 06846, *Pentapodus caninus*, OR114021.

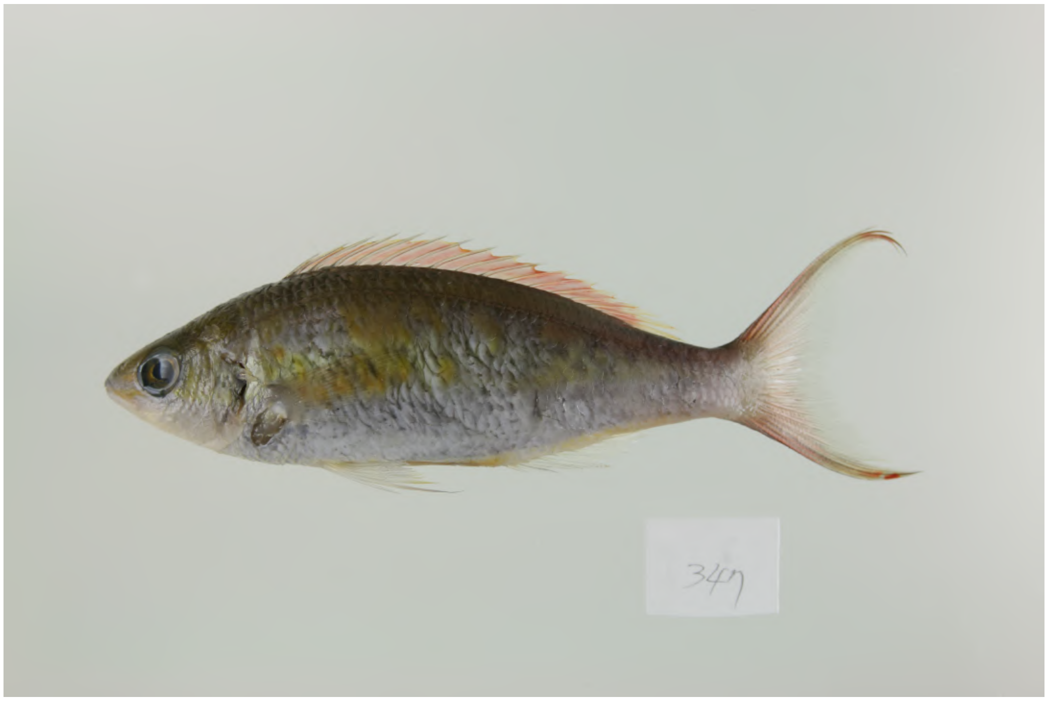

DOS 08685, *Pentapodus caninus*, OR114267.

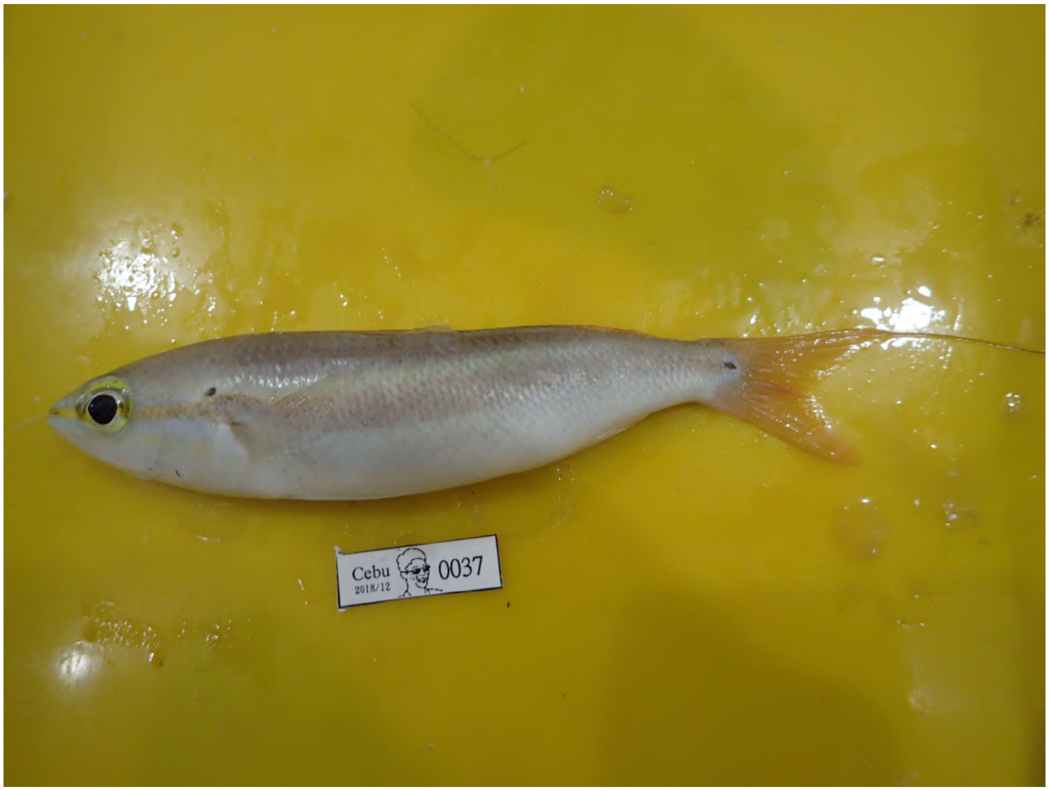

DOS 06845-2, *Pentapodus setosus*, OR114020. (specimen not preserved)

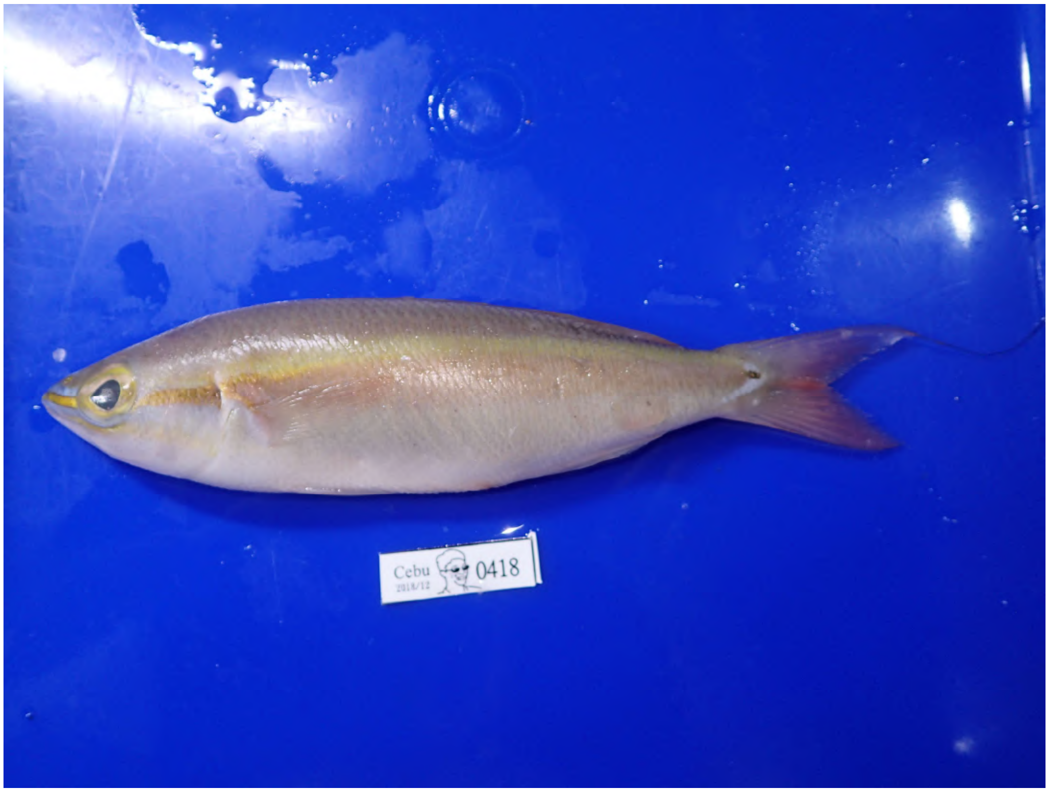

DOS 06847, *Pentapodus setosus*, OR114022. (specimen not preserved)

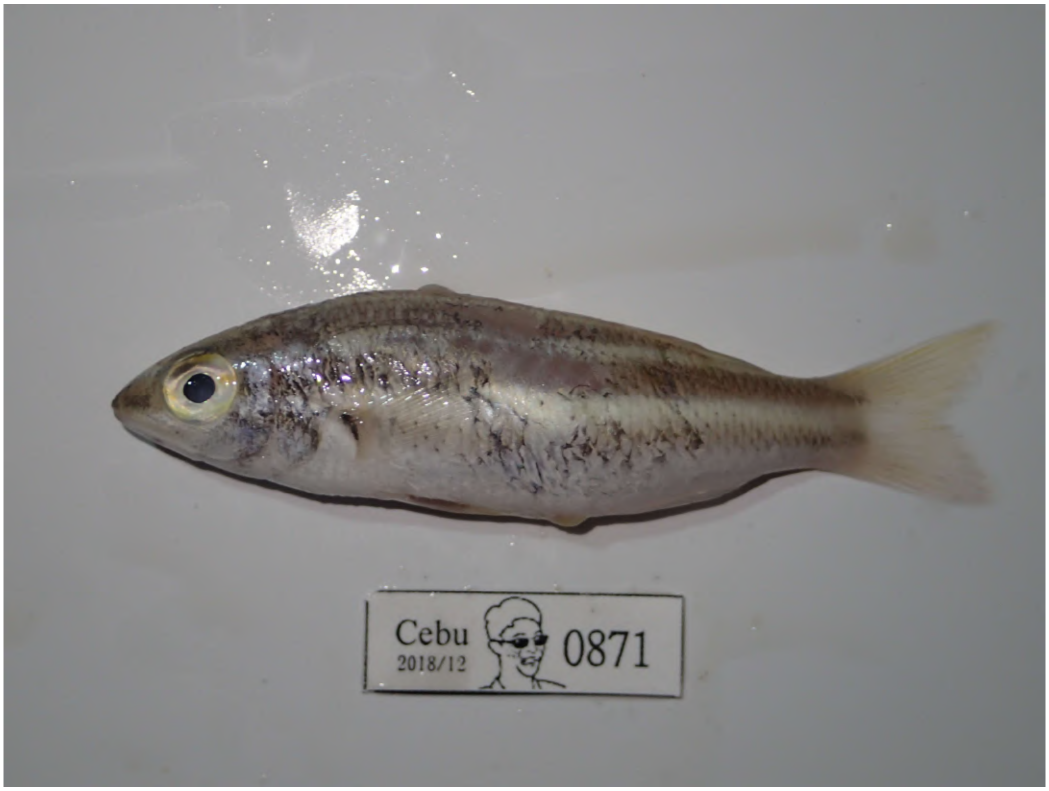

DOS 06855-2, *Pentapodus trivittatus*, OR114031.

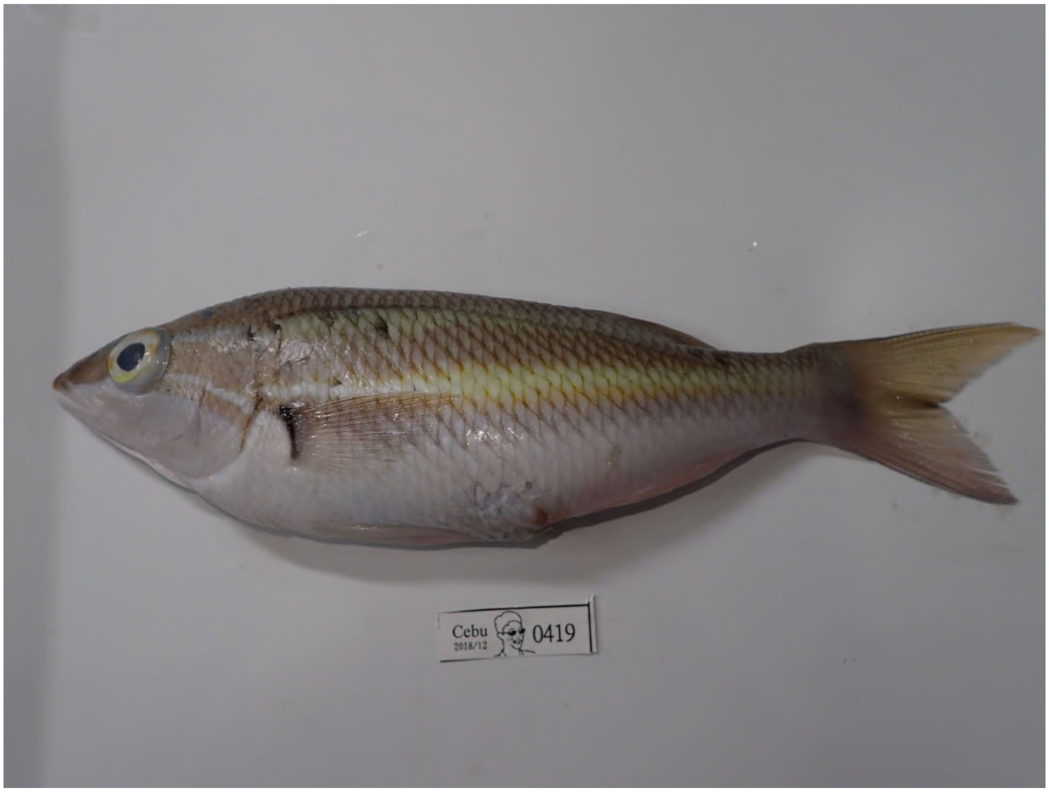

DOS 06856-1, *Pentapodus trivittatus*, OR114032.

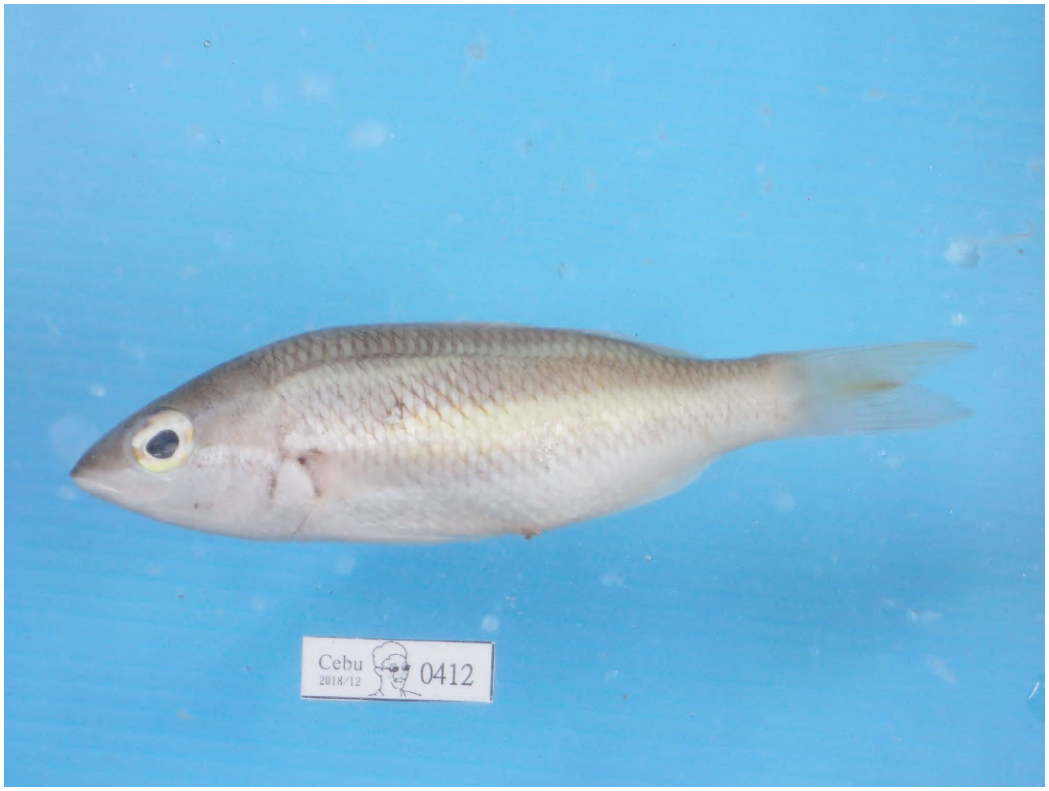

DOS 06857, *Pentapodus trivittatus*, OR114033.

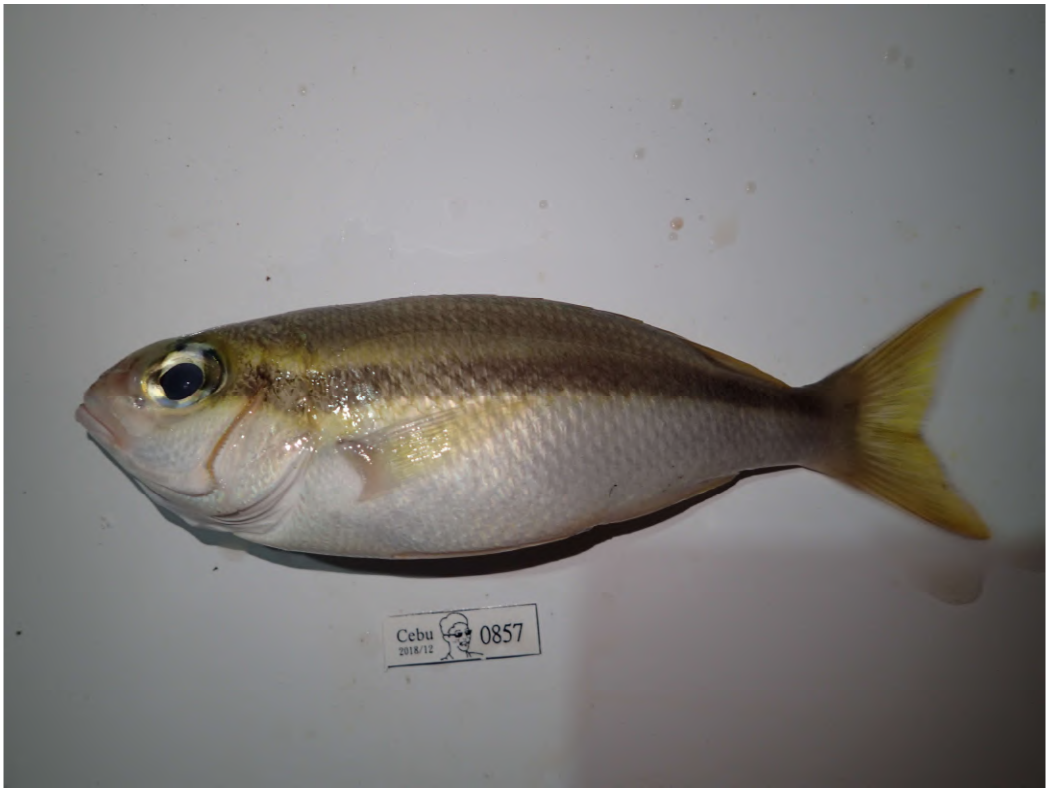

DOS 06850-1, *Scolopsis affinis*, OR114025.

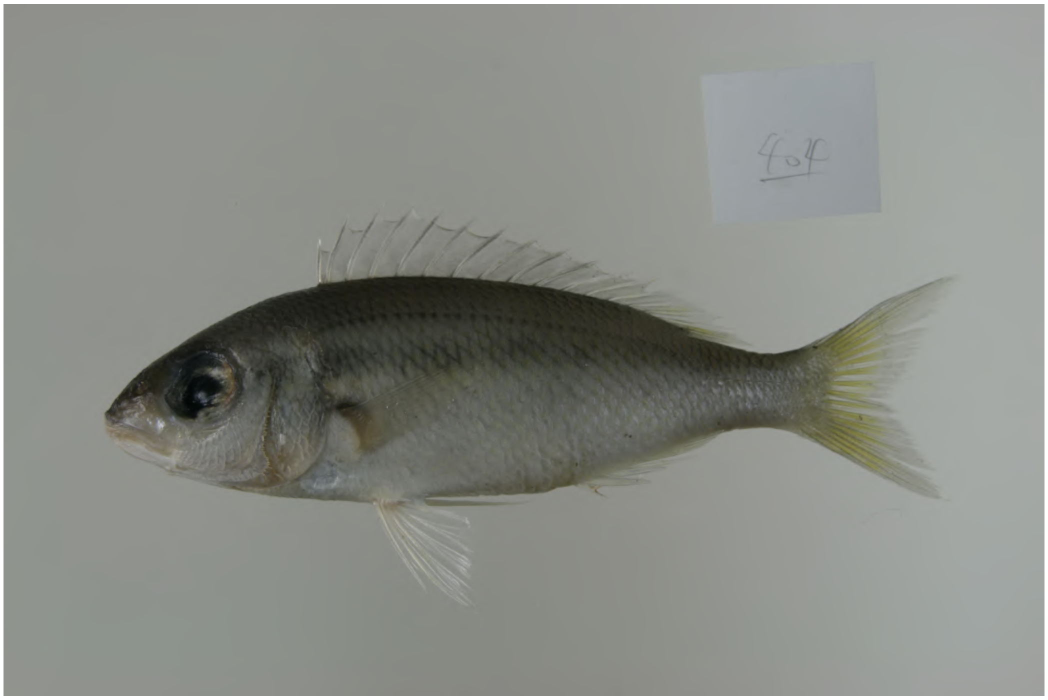

DOS 08686-1, *Scolopsis affinis*, OR114268.

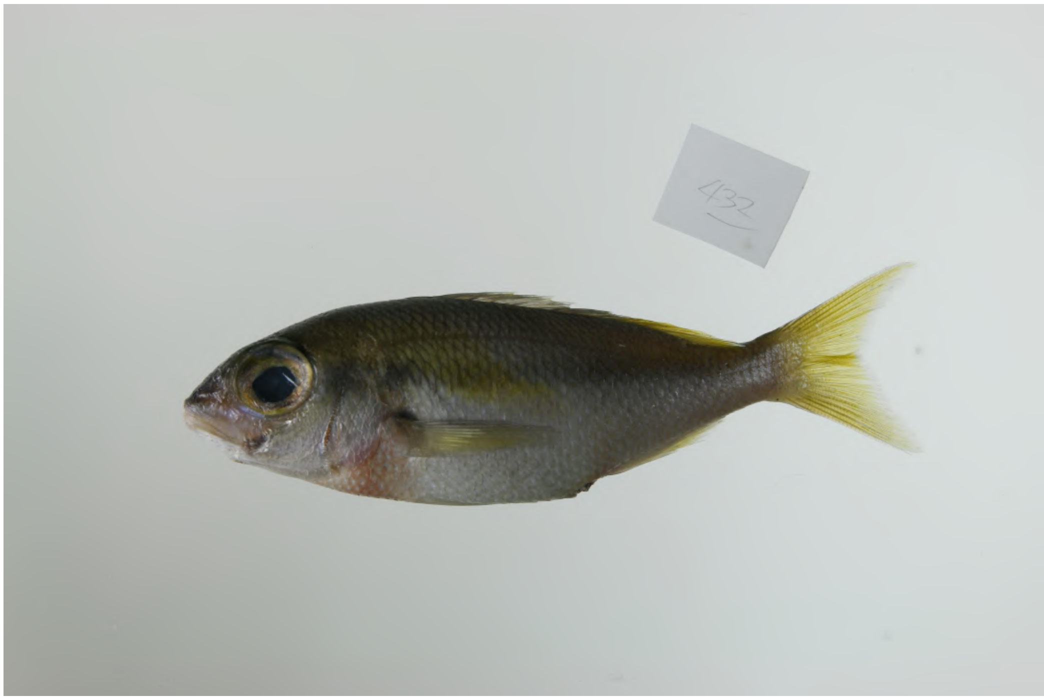

DOS 08686-2, *Scolopsis affinis*, OR114269.

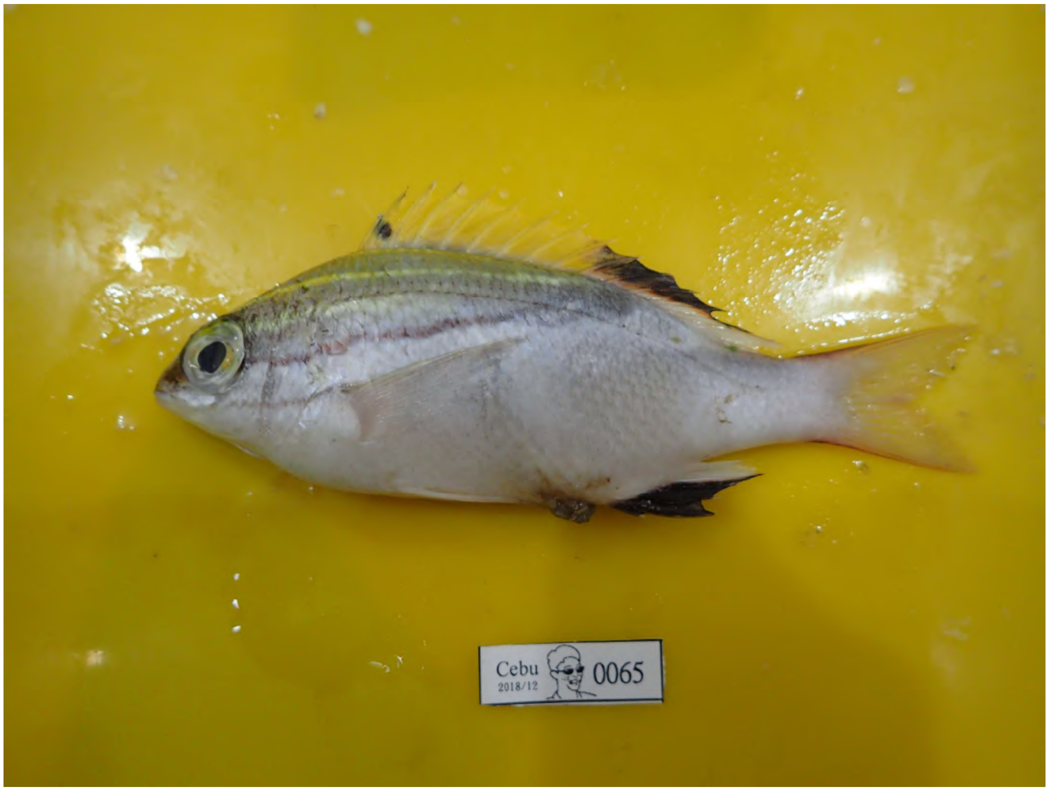

DOS 06851-1, *Scolopsis bilineata*, OR114026. (specimen not preserved)

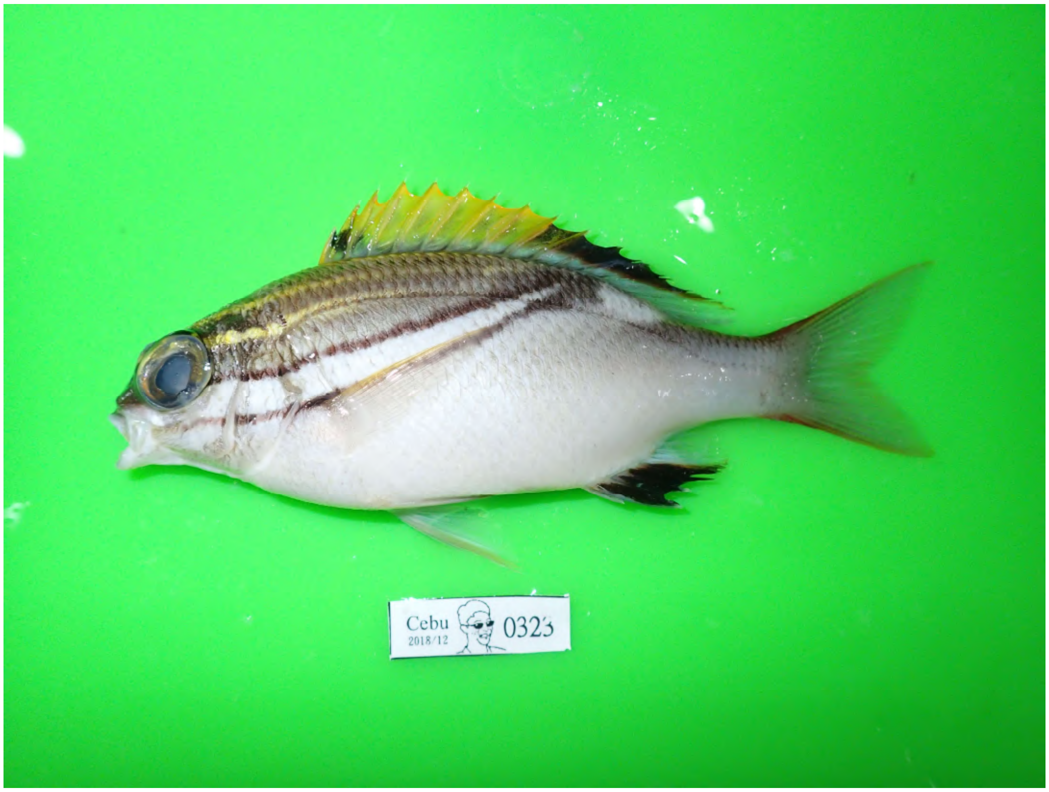

DOS 06851-3, *Scolopsis bilineata*, OR114027.

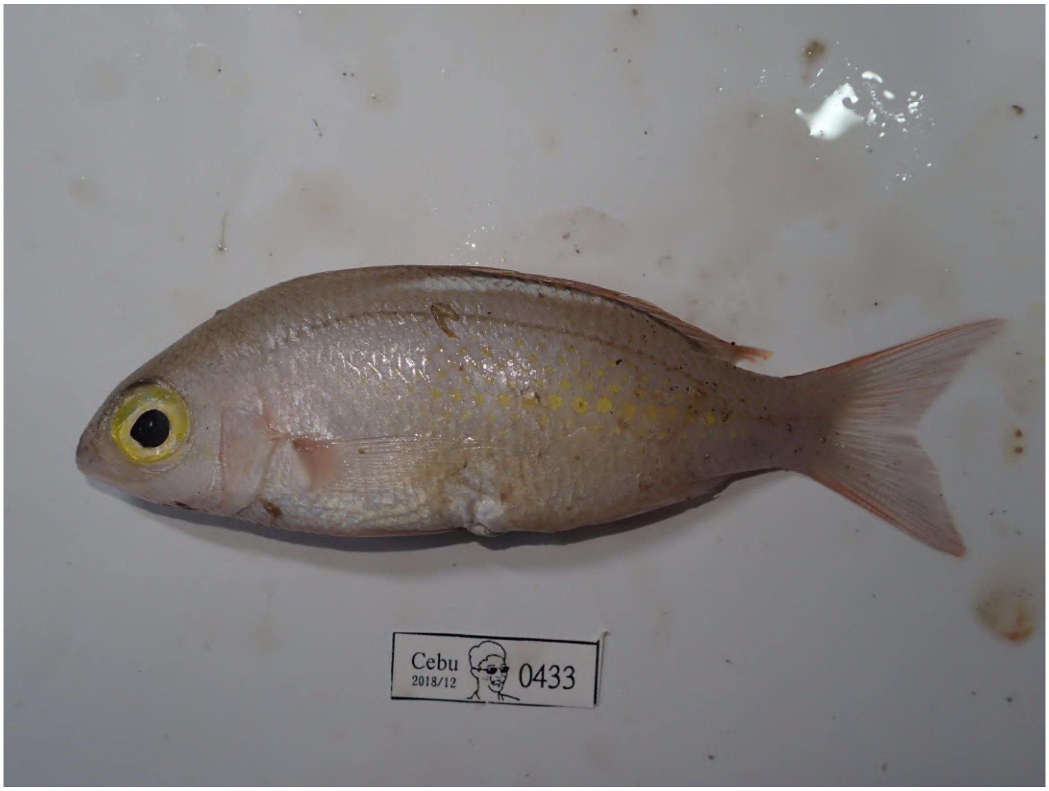

DOS 06842, *Scolopsis ciliata*, OR114017.

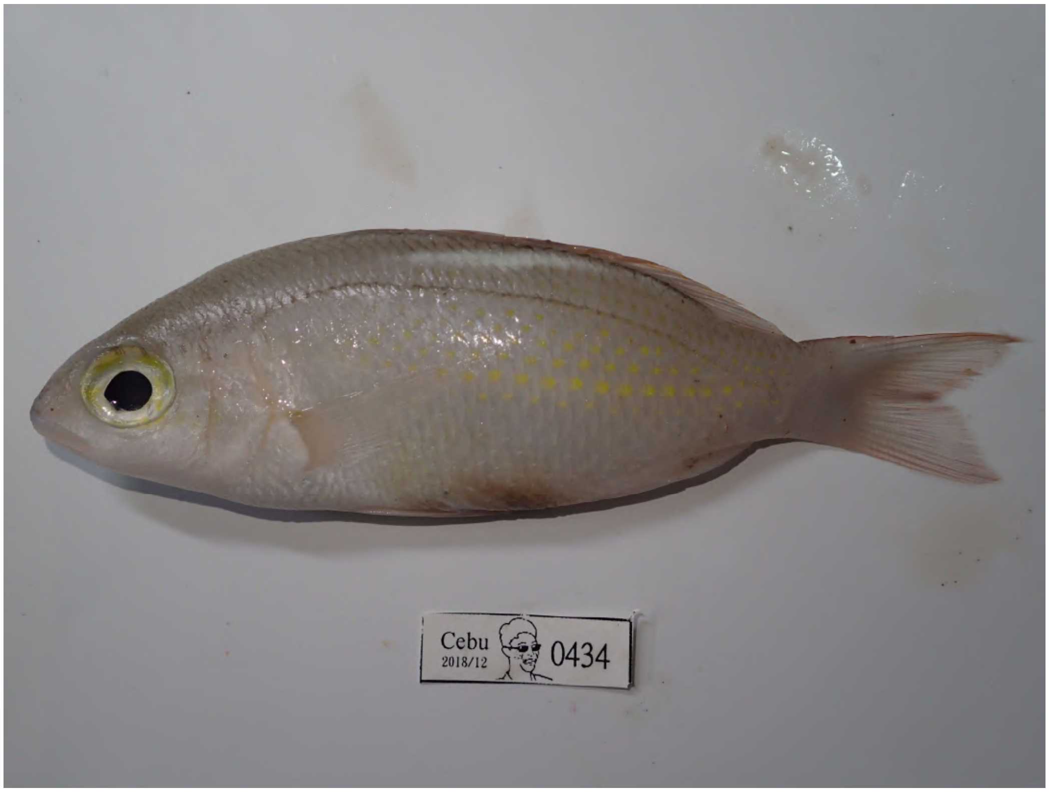

DOS 06852-3, *Scolopsis ciliata*, OR114028.

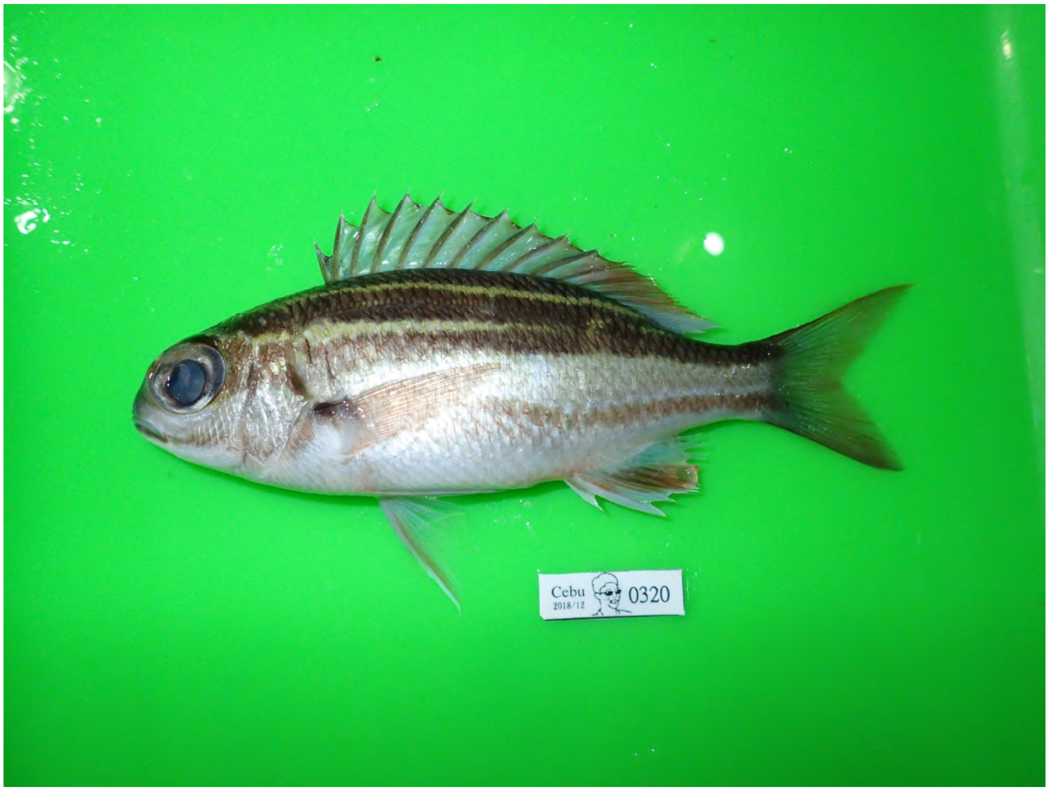

DOS 06853-1, *Scolopsis lineata*, OR114029.

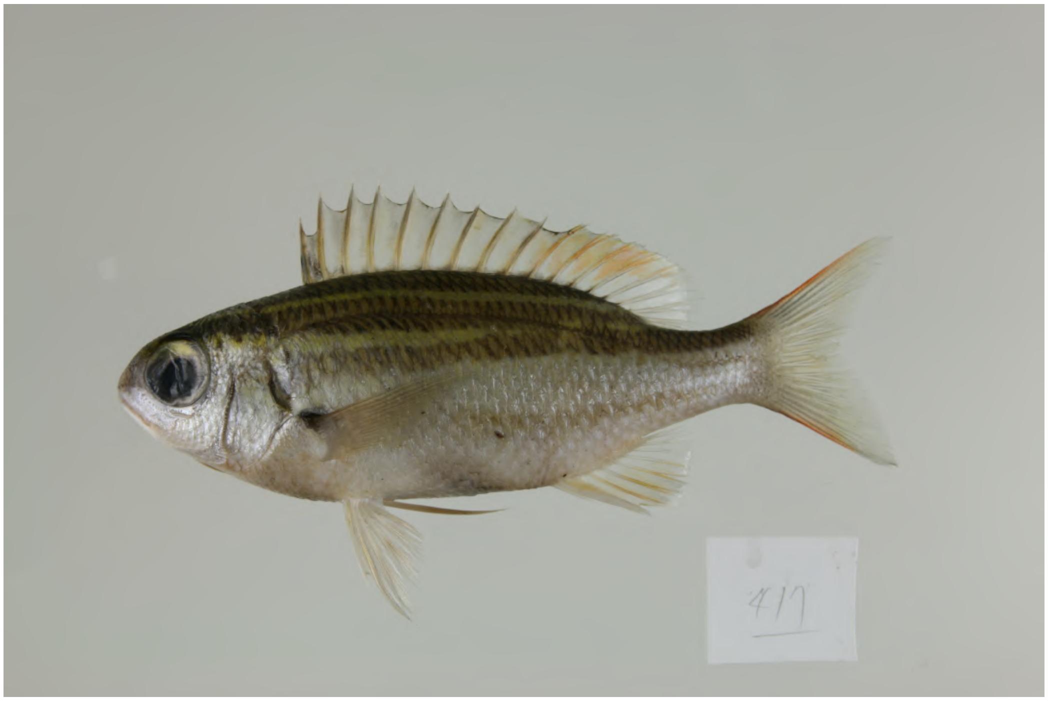

DOS 08687, *Scolopsis lineata*, OR114270.

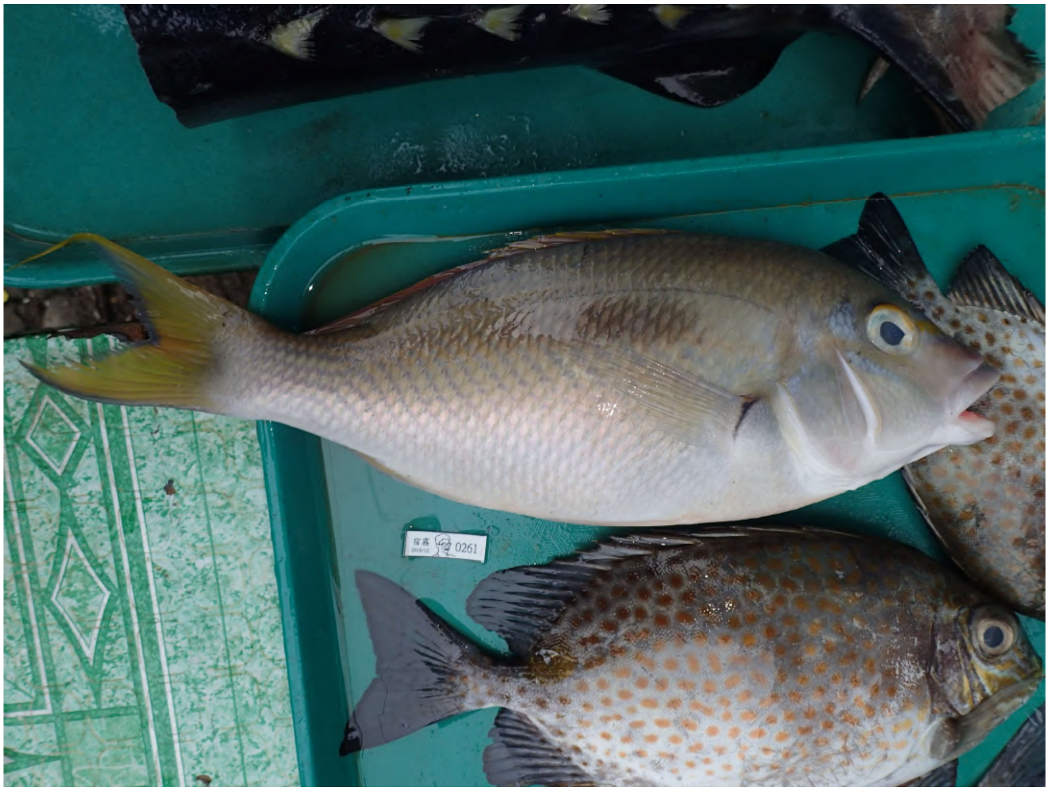

DOS 06777-1, *Scolopsis monogramma*, OR113958. (top, specimen not preserved)

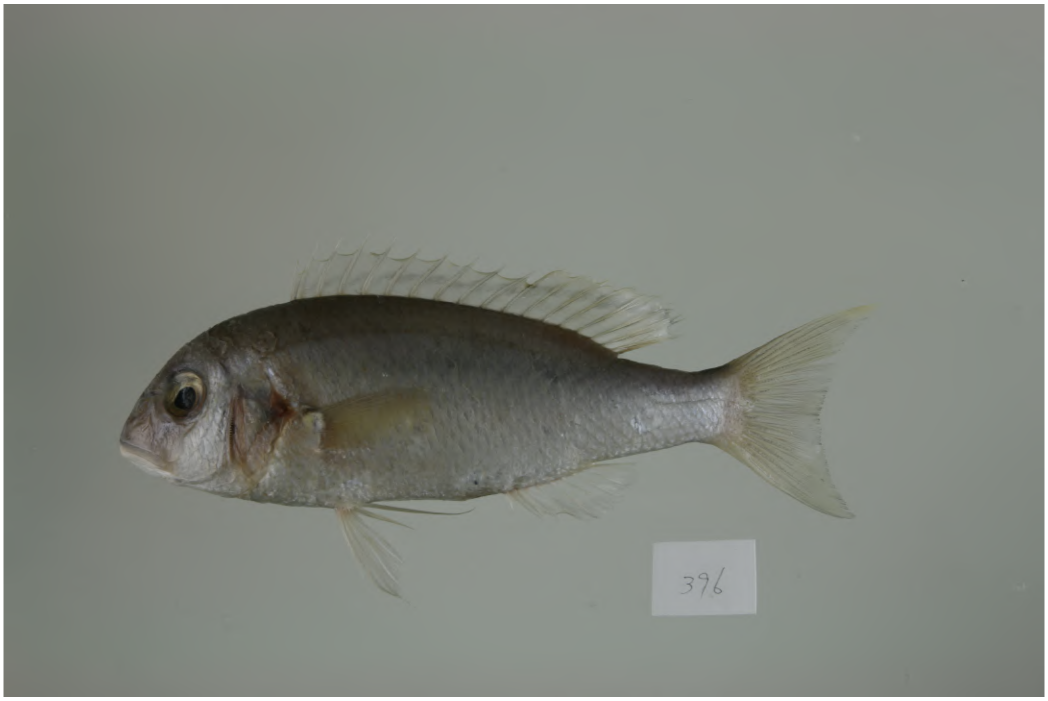

DOS 08688, *Scolopsis taenioptera*, OR114271.

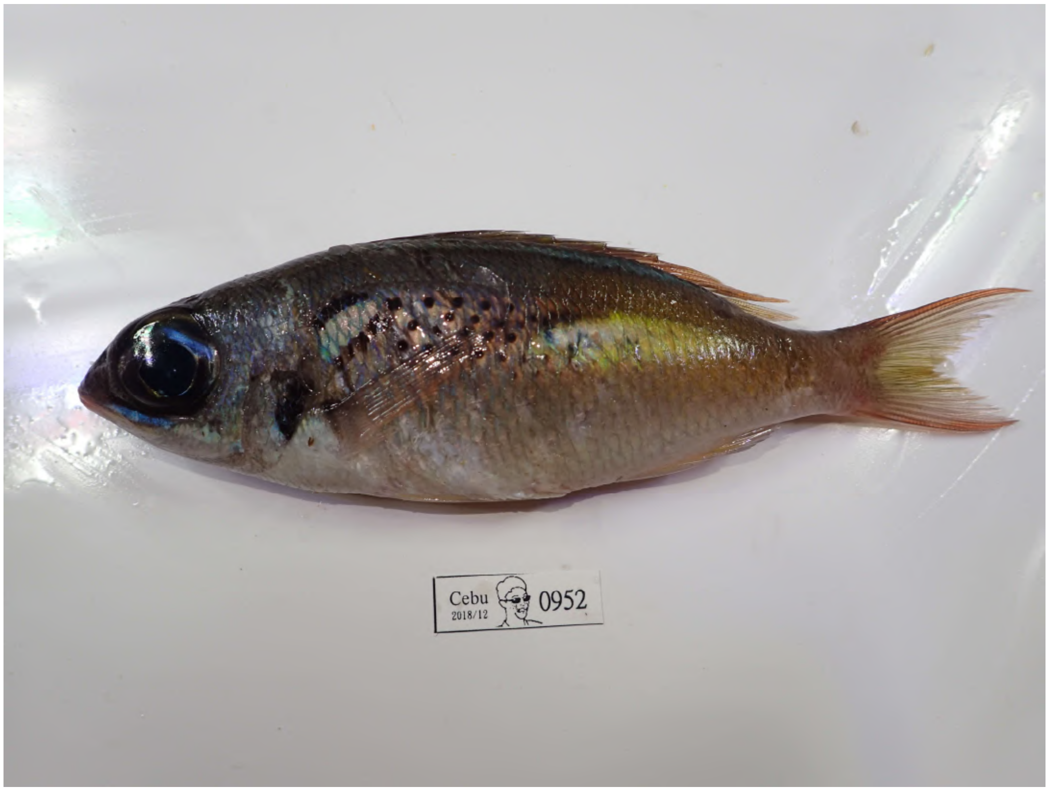

DOS 06858, *Scolopsis xenochrous*, OR114034.

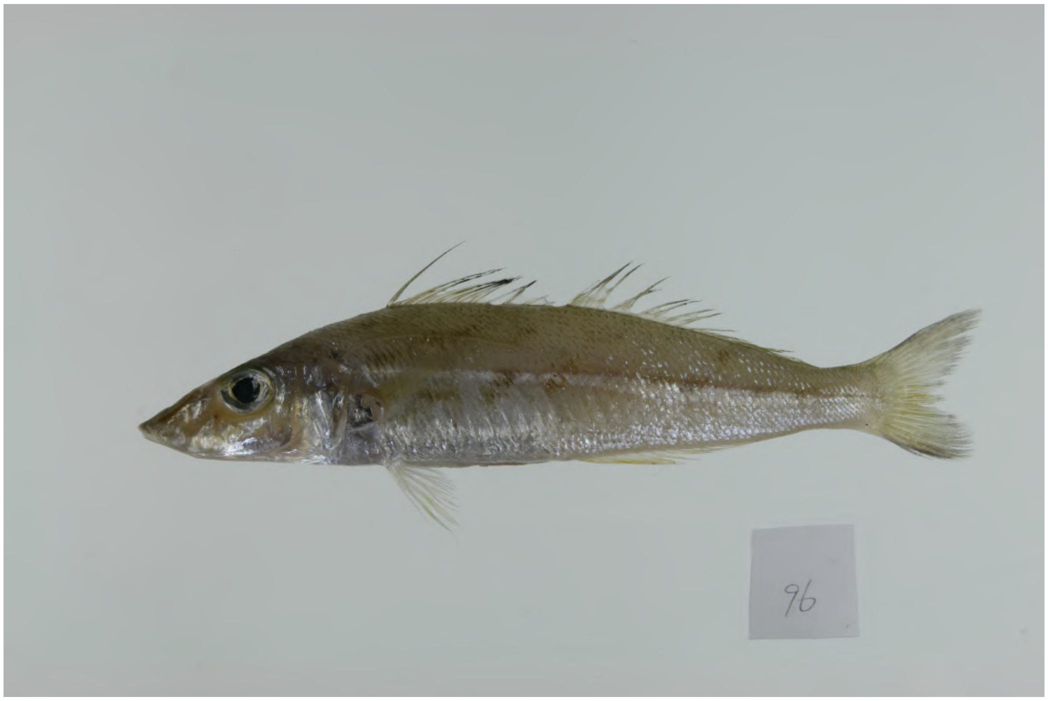

DOS 08689-1, *Sillago aeolus*, OR114272.

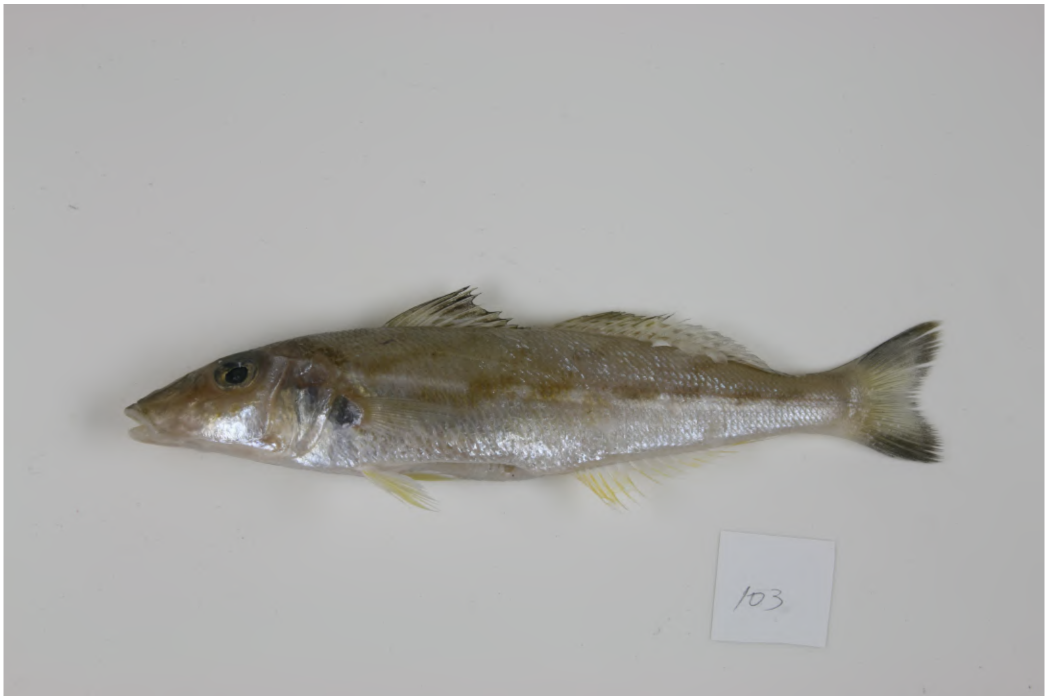

DOS 08689-2, *Sillago aeolus*, OR114273.

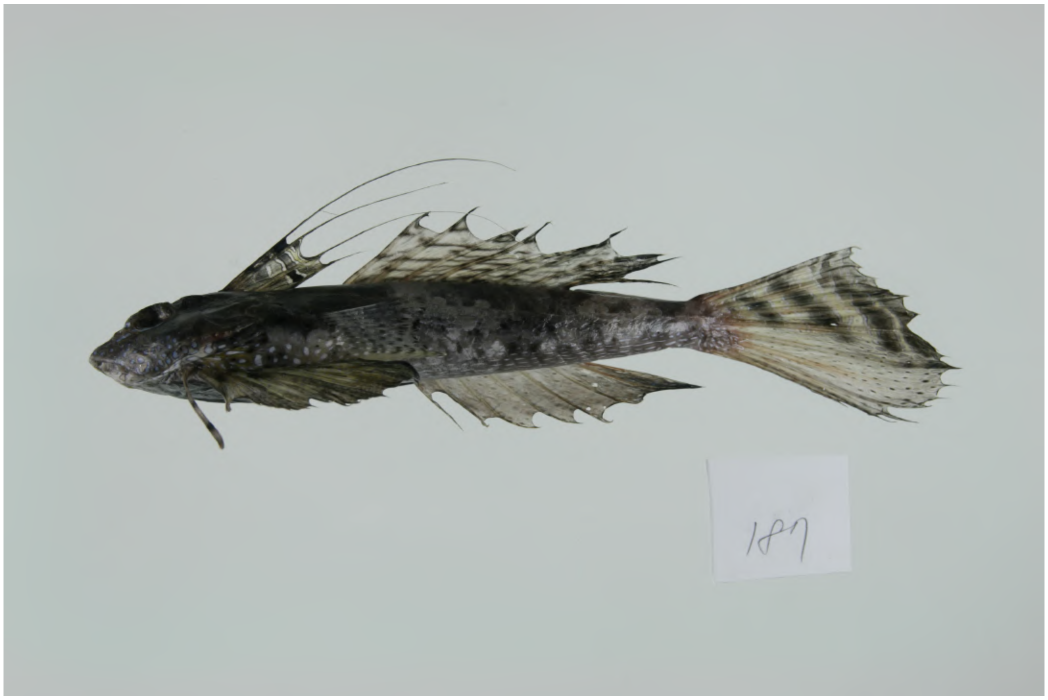

DOS 08634, *Dactylopus dactylopus*, OR114206.

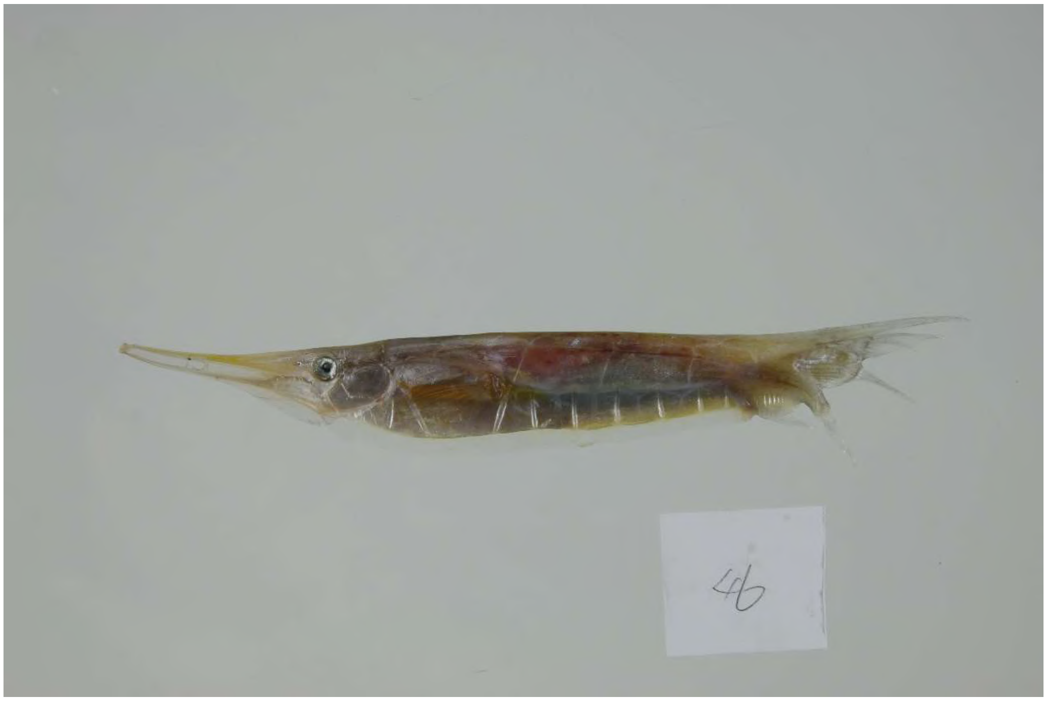

DOS 08690, *Centriscus scutatus*, OR114274.

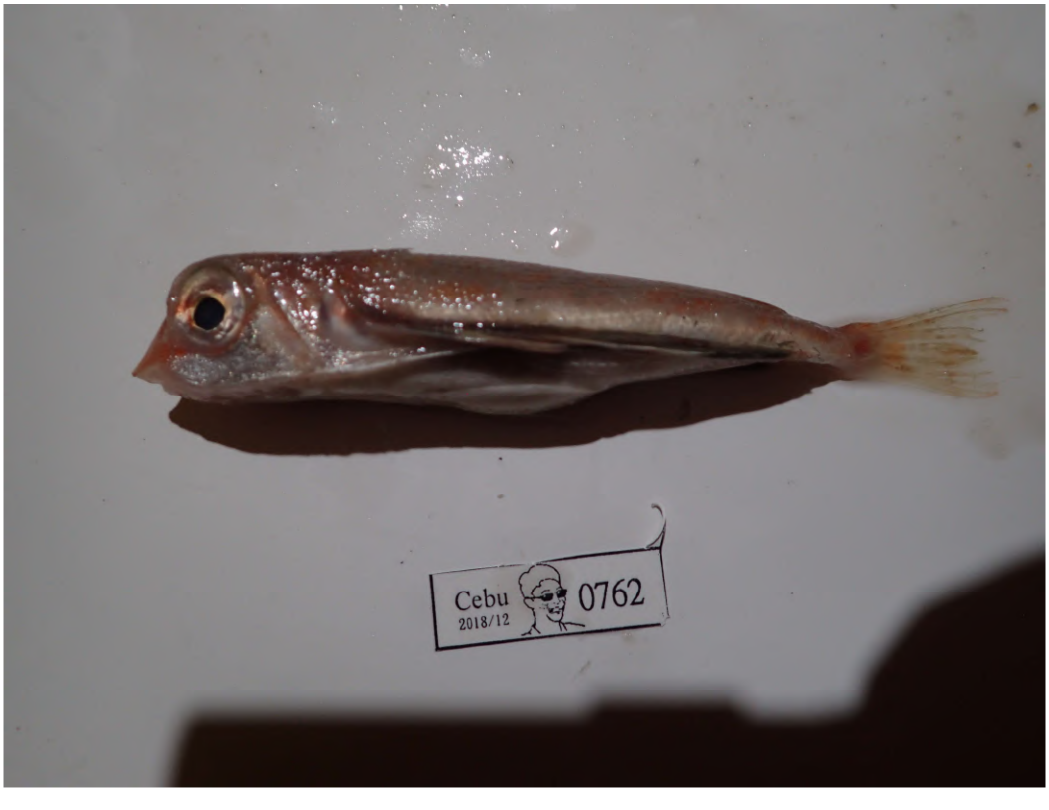

DOS 06684, *Dactyloptena tiltoni*, OR113874.

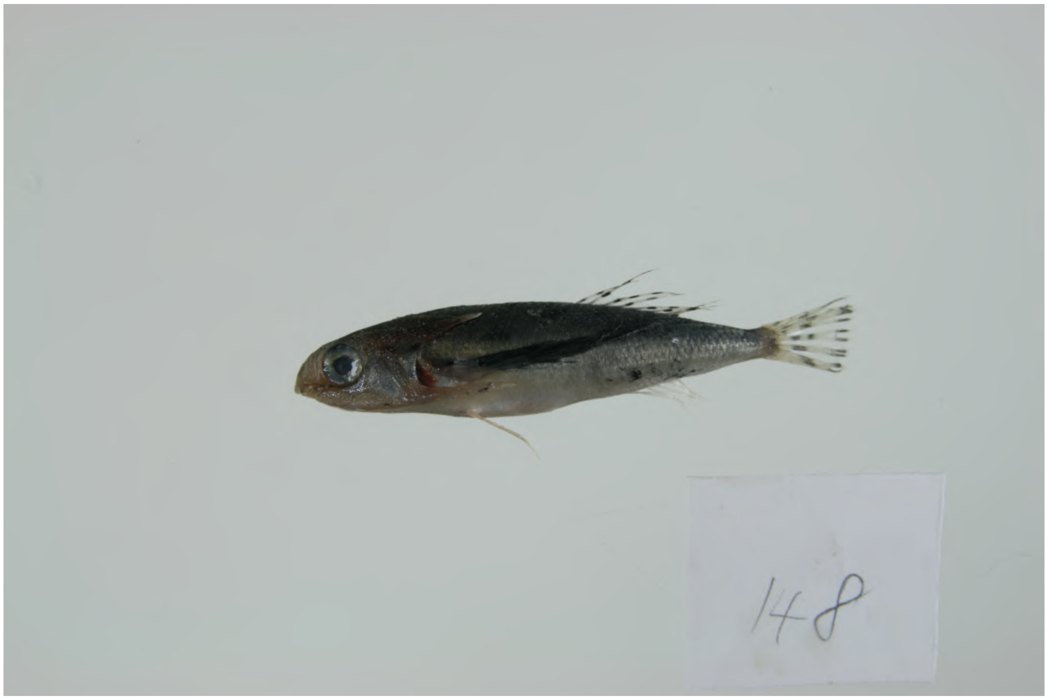

DOS 08691-1, *Dactyloptena tiltoni*, OR113875.

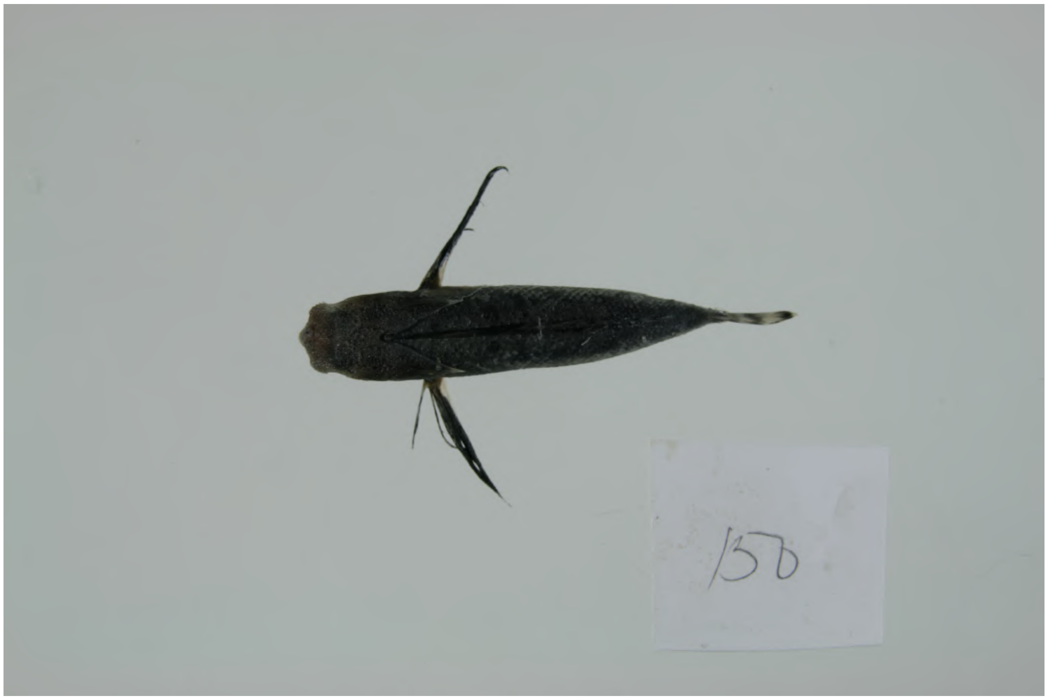

DOS 08691-2, *Dactyloptena tiltoni*, OR113876.

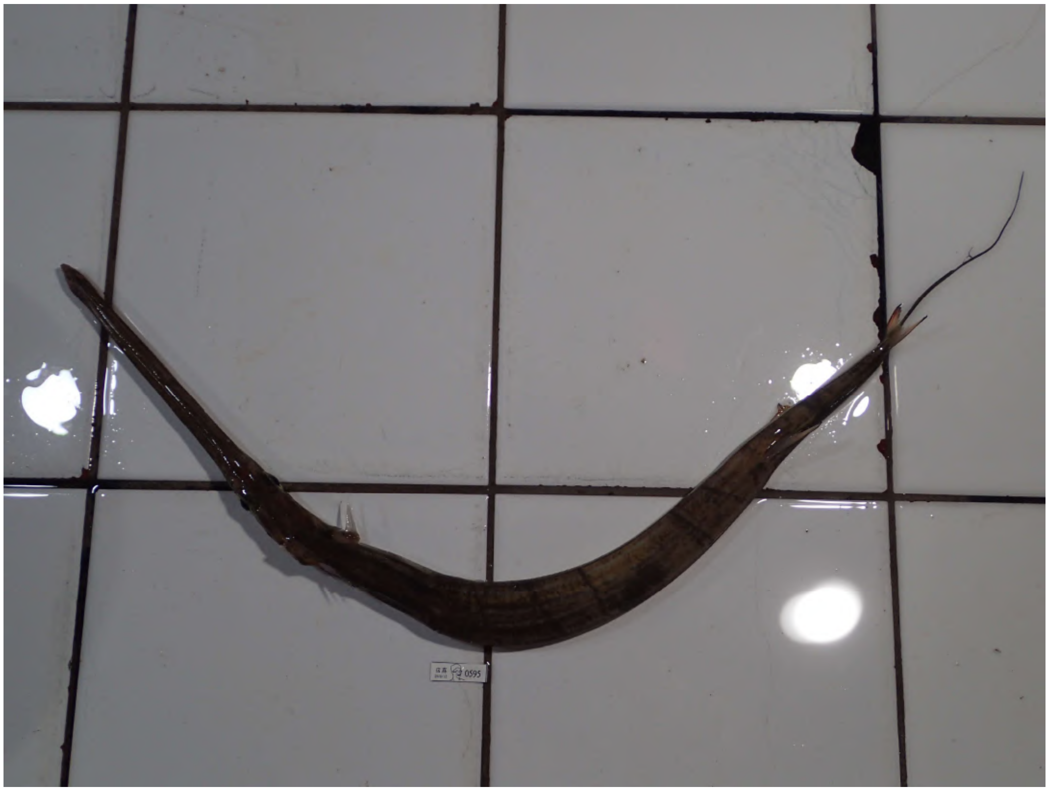

DOS 06698-1, *Fistularia commersonii*, OR113886. (specimen not preserved)

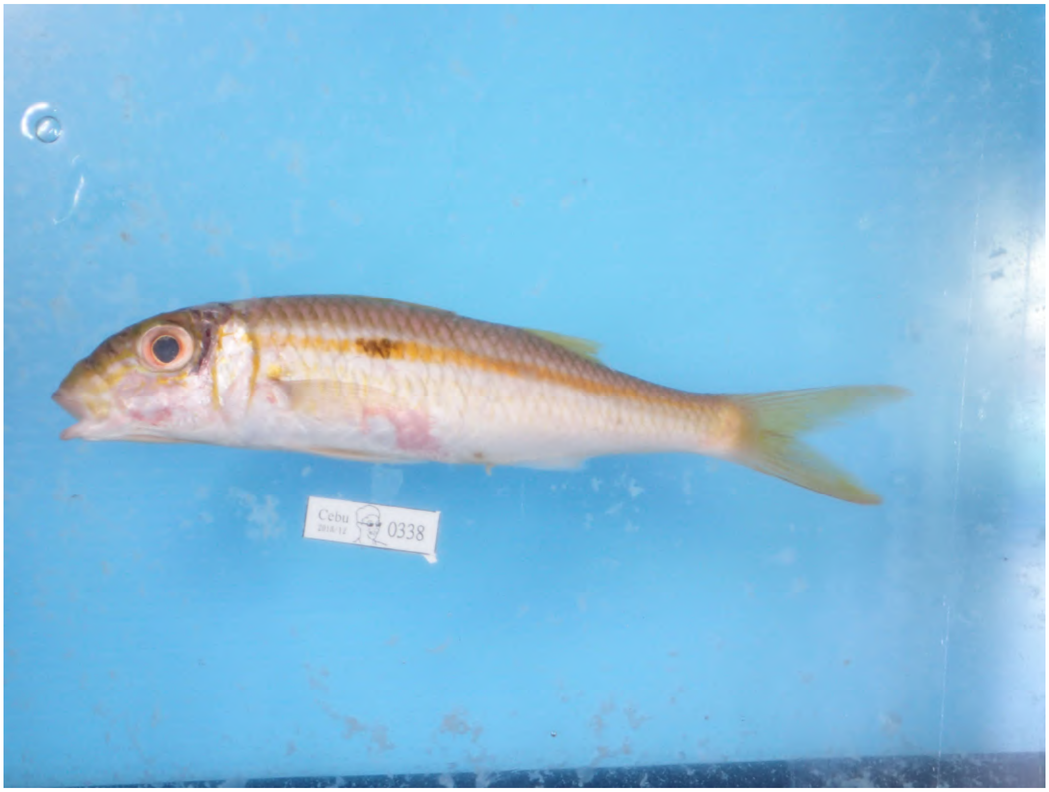

DOS 06809-1, *Mulloidichthys flavolineatus*, OR113991.

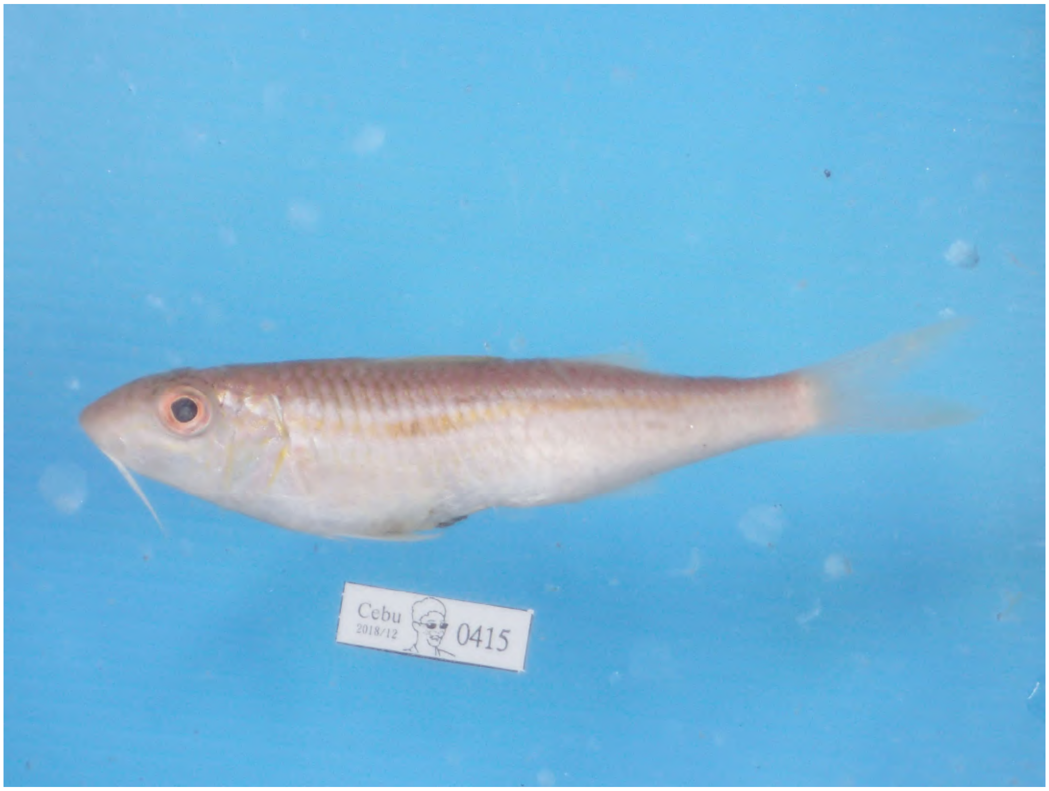

DOS 06812, *Mulloidichthys flavolineatus*, OR113993.

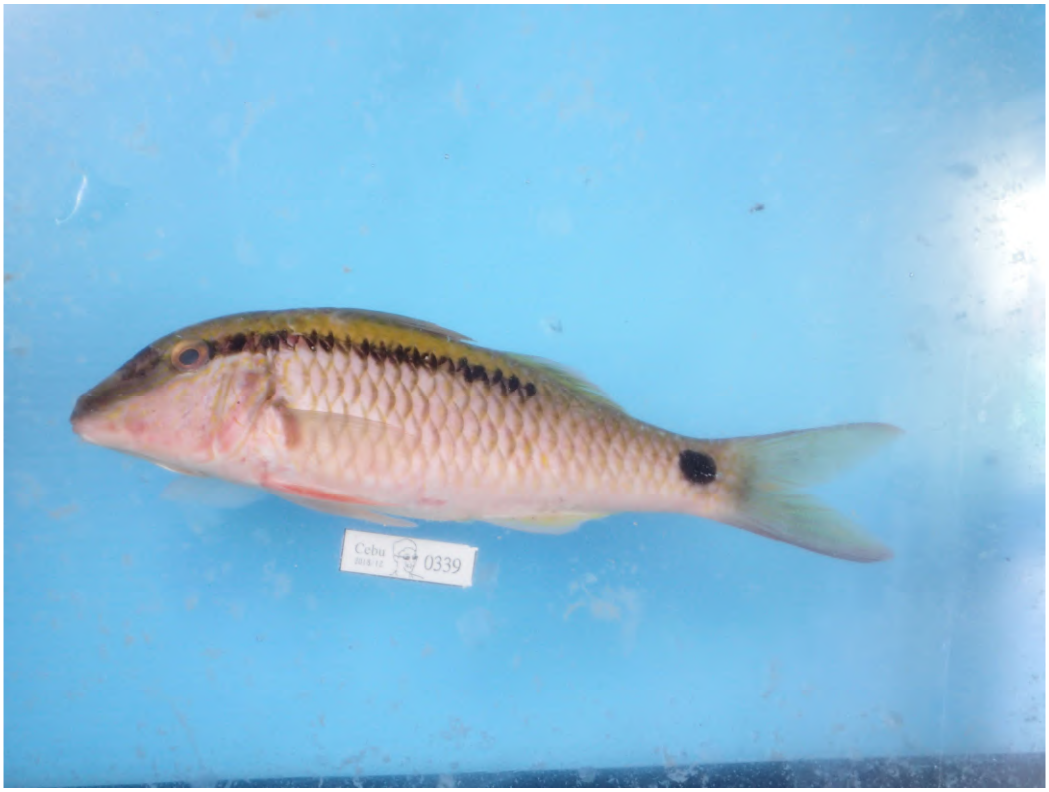

DOS 06811-1, *Parupeneus barberinus*, OR113992. (specimen not preserved)

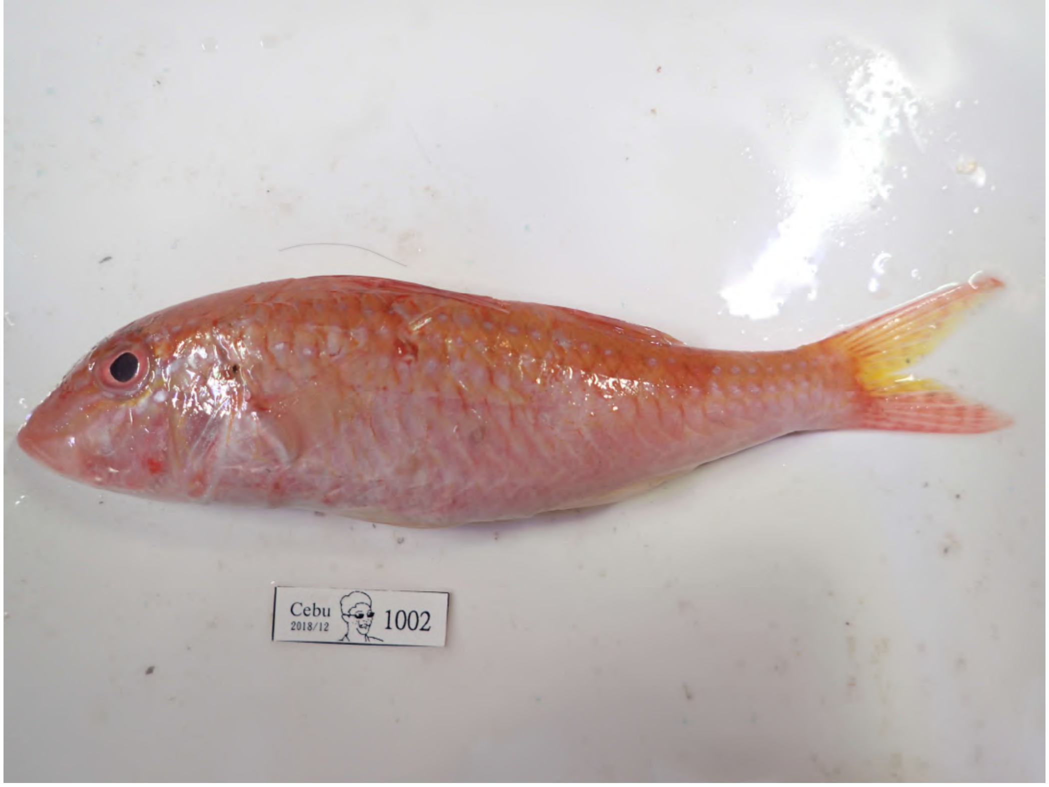

DOS 06813-1, *Parupeneus heptacanthus*, OR113994. (specimen not preserved)

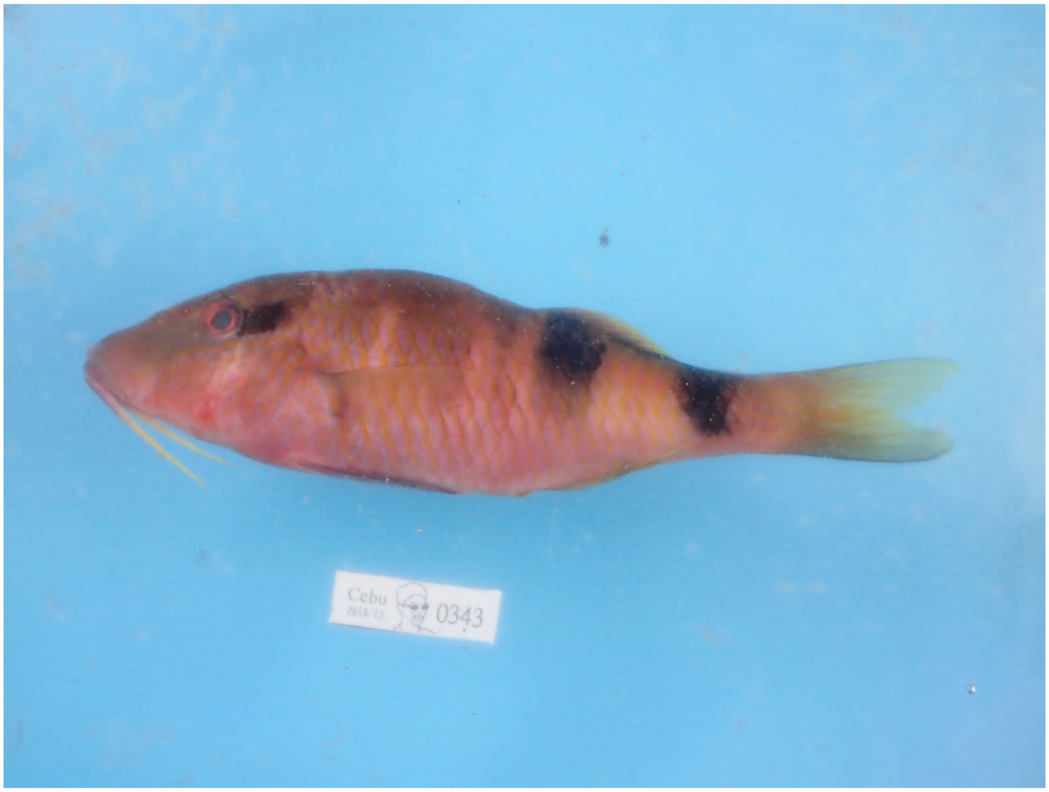

DOS 06814-1, *Parupeneus multifasciatus*, OR113995.

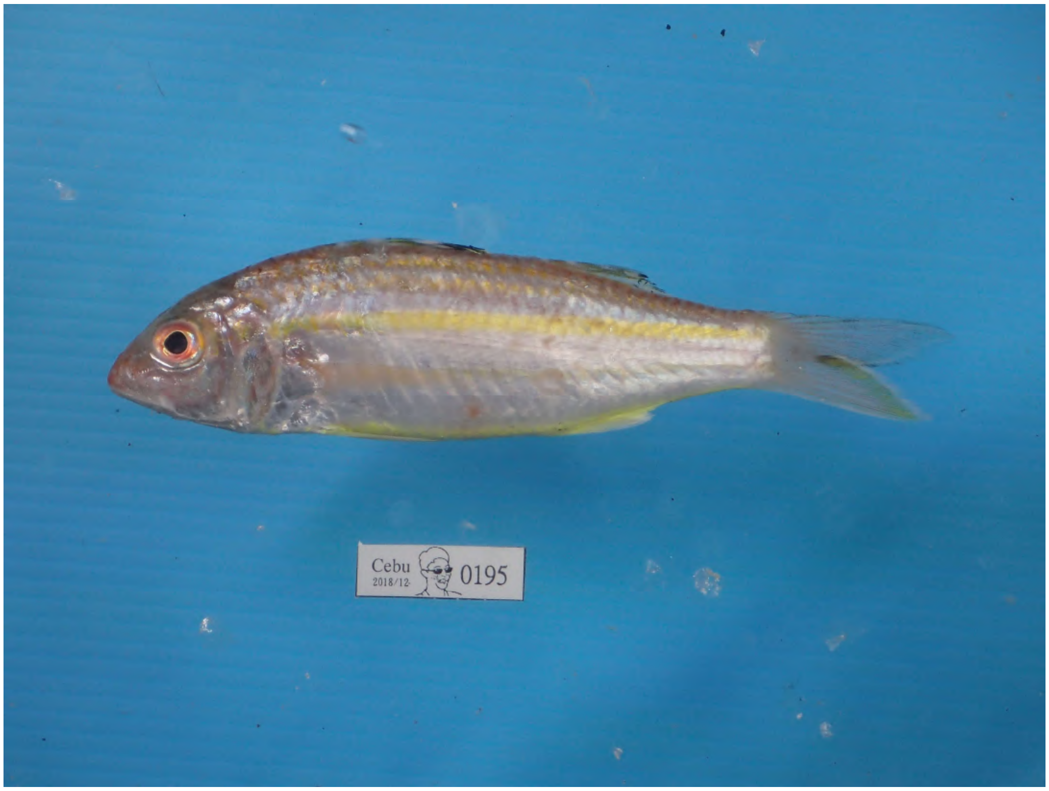

DOS 06816-1, *Upeneus sulphureus*, OR113997.

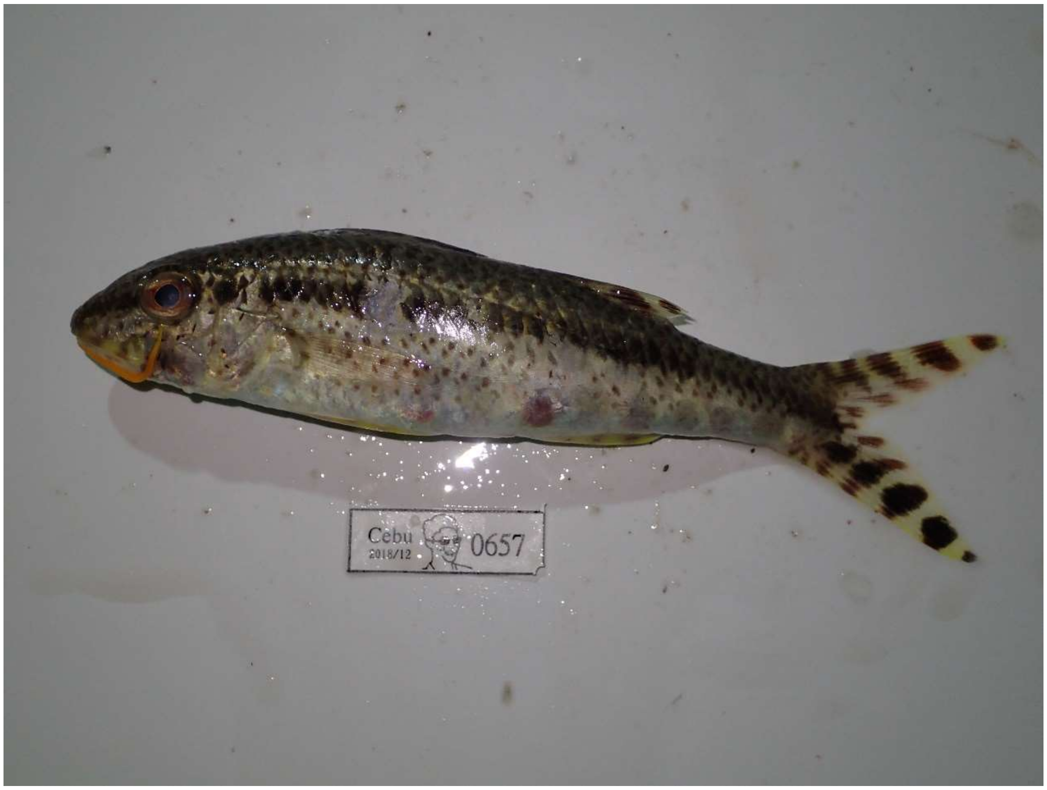

DOS 06815, *Upeneus tragula*, OR113996.

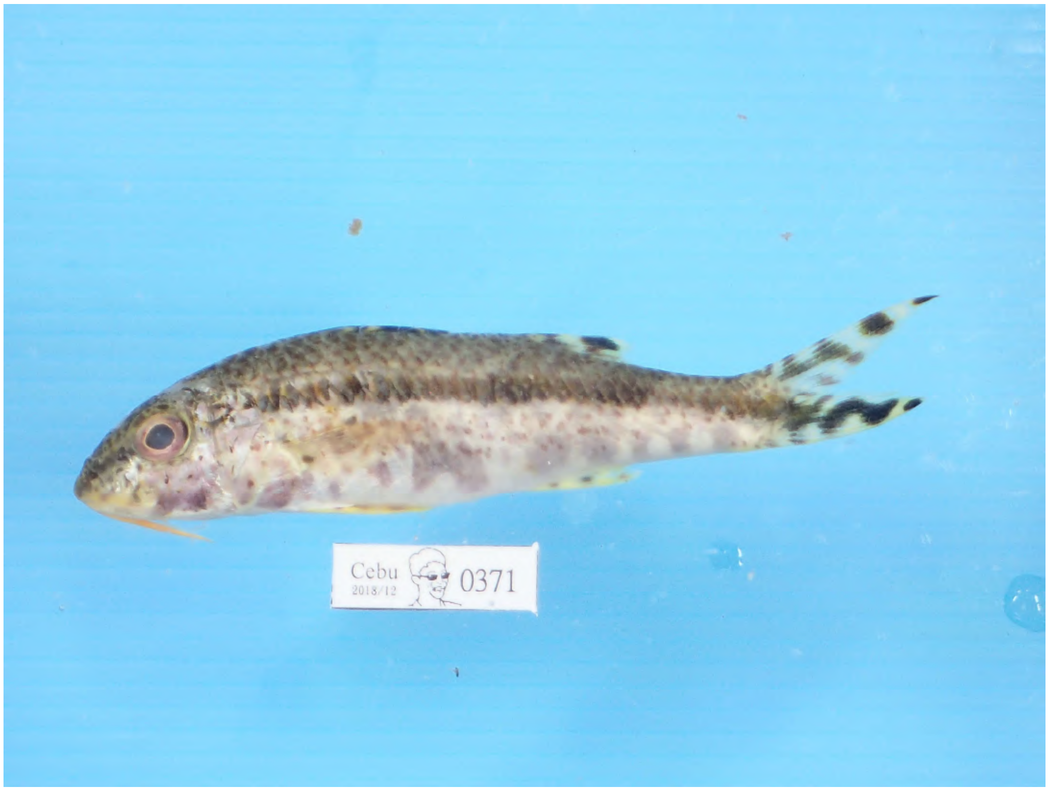

DOS 06817-1, *Upeneus tragula*, OR113998.

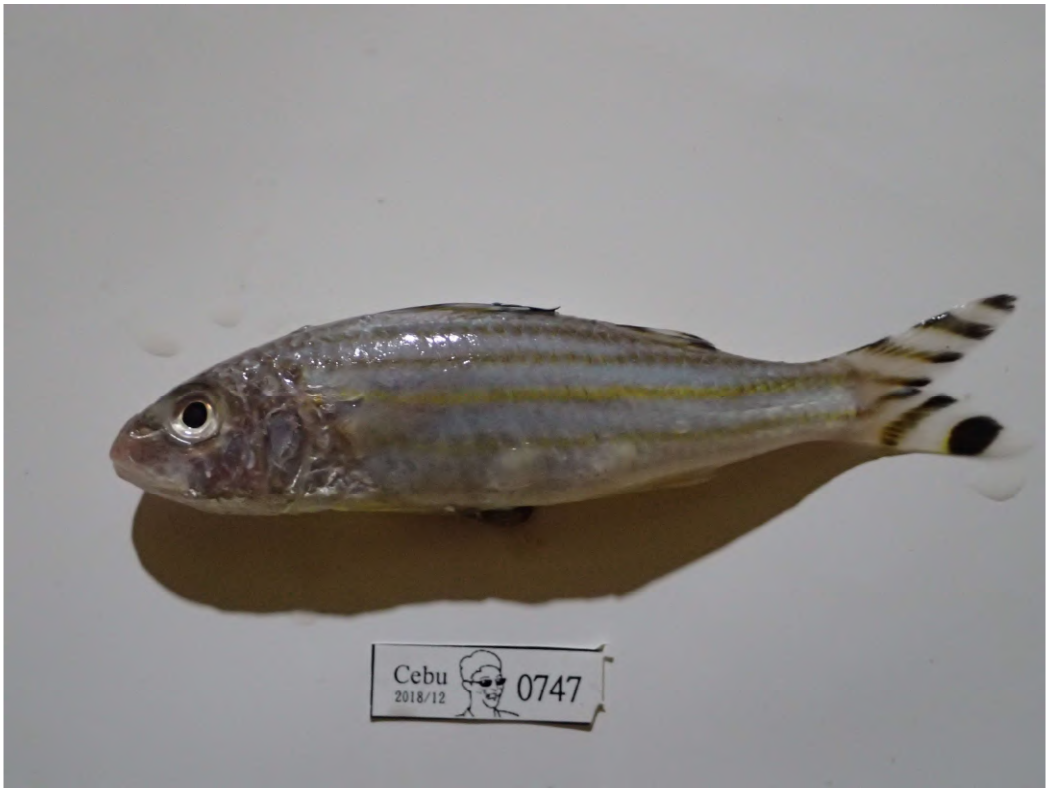

DOS 06818-1, *Upeneus vittatus*, OR113999.

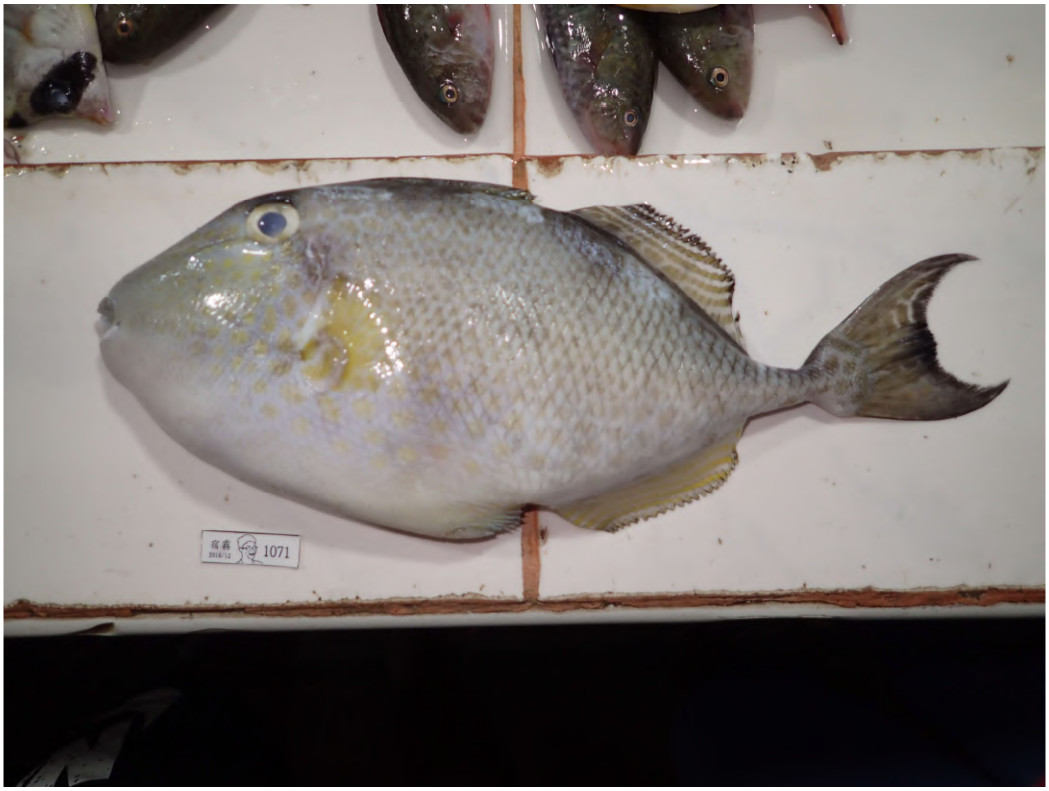

DOS 06605-1, *Abalistes stellatus*, OR113796. (specimen not preserved)

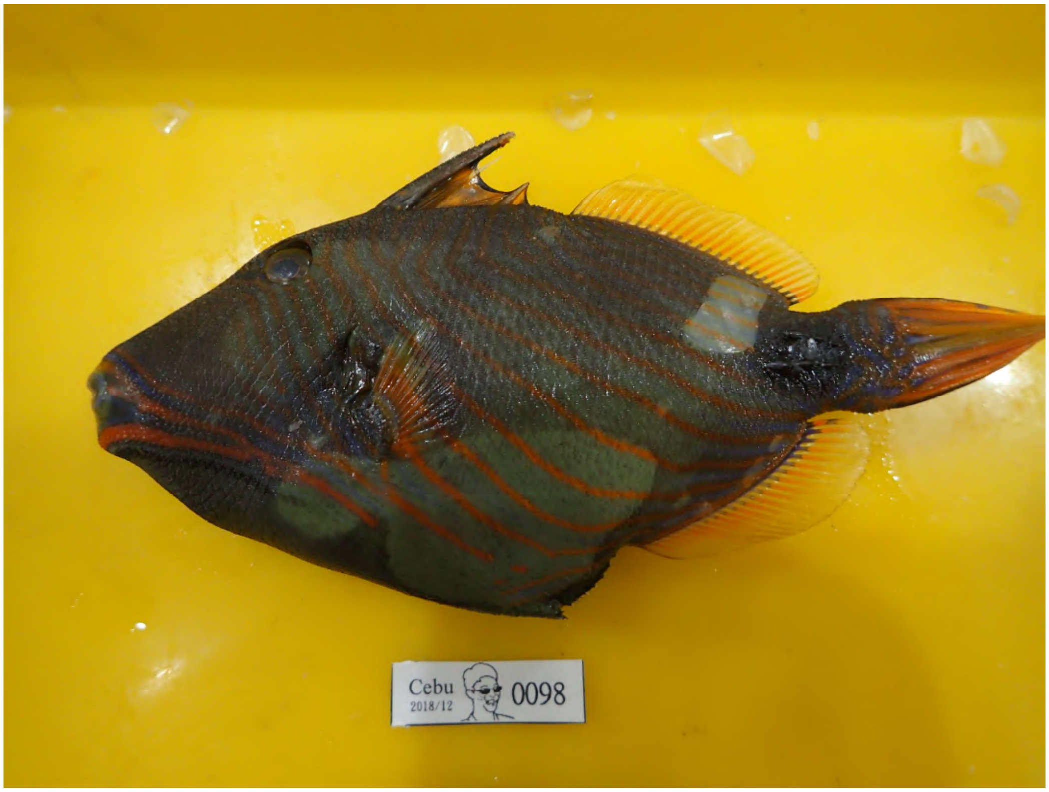

DOS 06606-1, *Balistapus undulatus*, OR113797. (specimen not preserved)

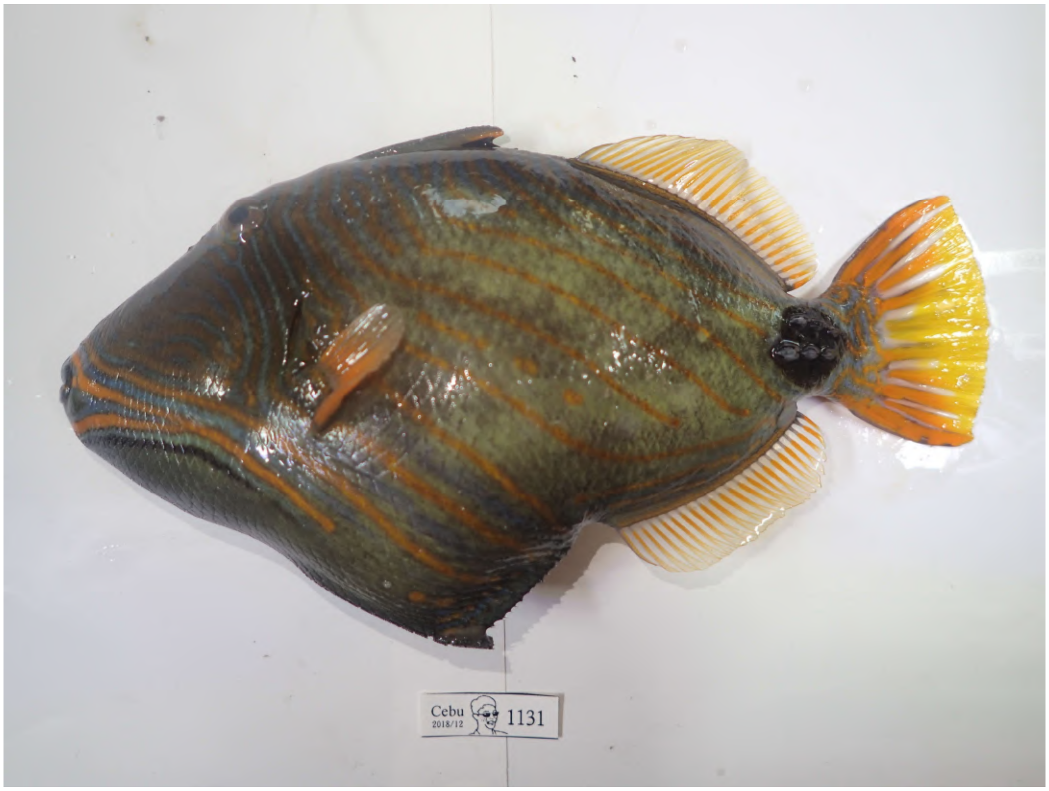

DOS 06607, *Balistapus undulatus*, OR113798. (specimen not preserved)

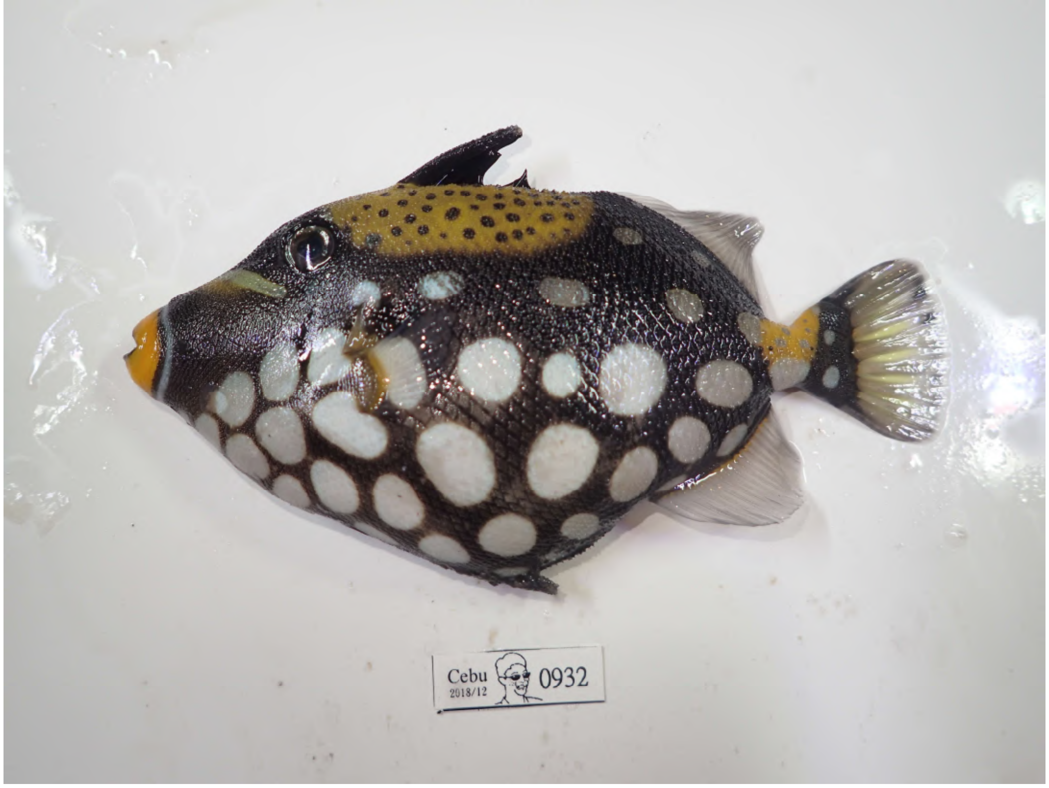

DOS 06608, *Balistoides conspicillum*, OR113799.

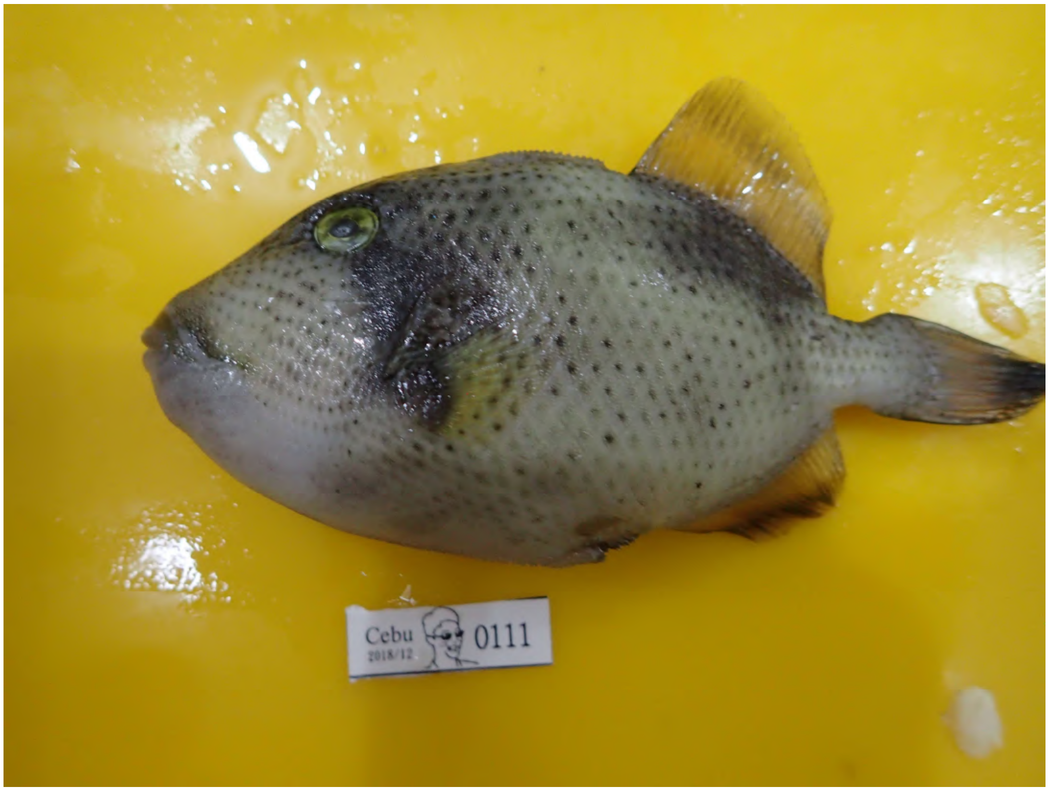

DOS 06609-1, *Balistoides viridescens*, OR113800.

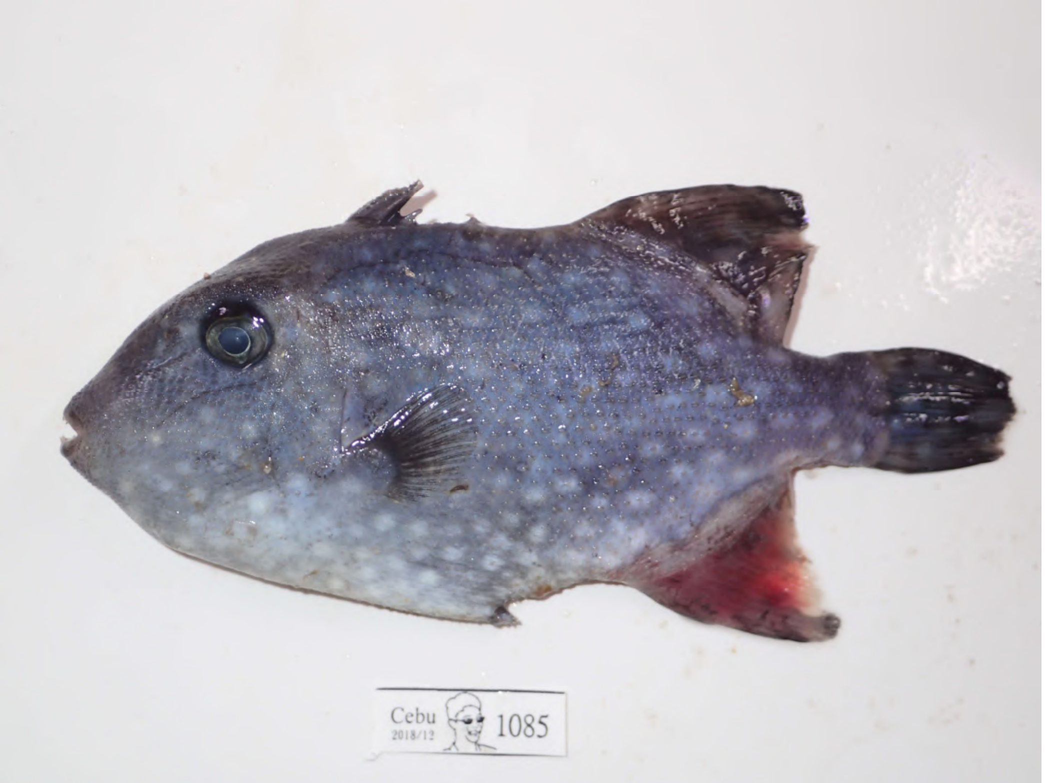

DOS 06610, *Canthidermis maculata*, OR113801.

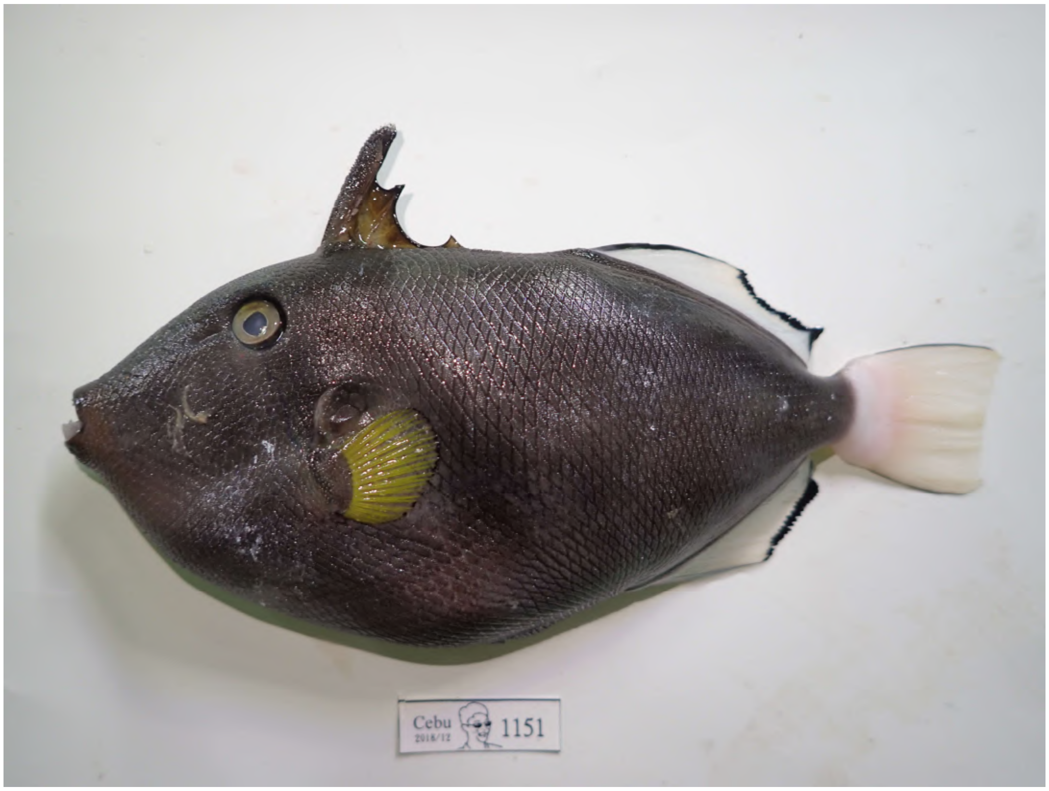

DOS 06611, *Melichthys vidua*, OR113802.

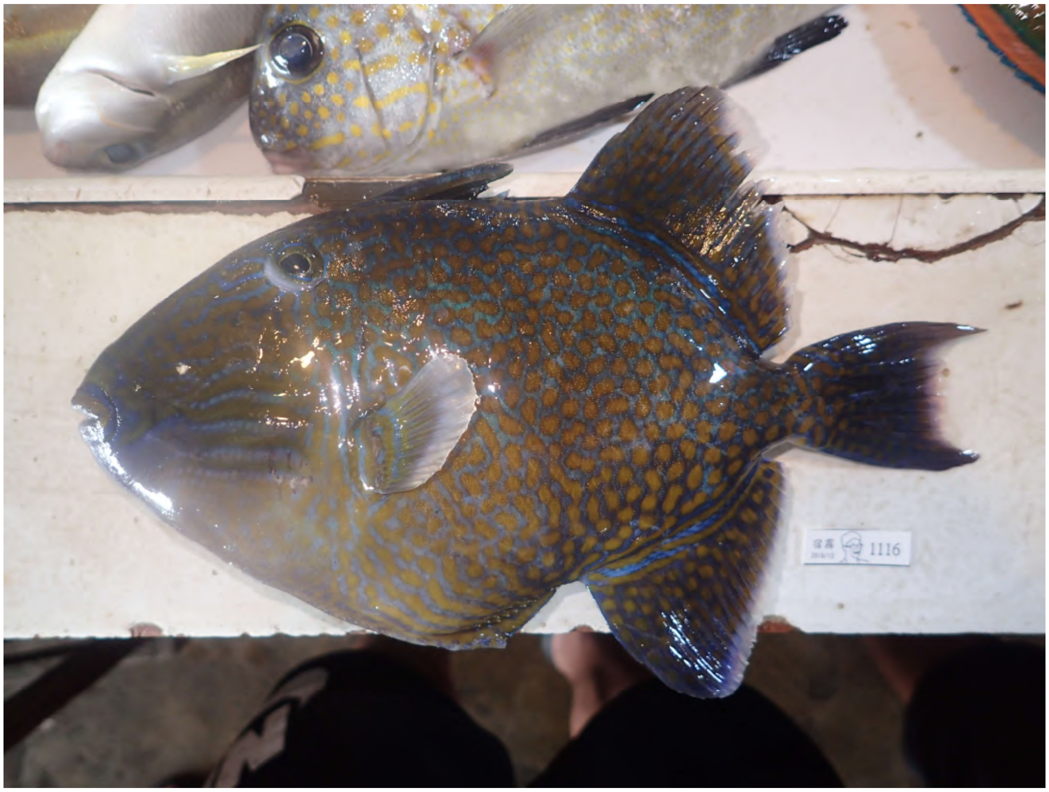

DOS 06612, *Pseudobalistes fuscus*, OR113803. (specimen not preserved)

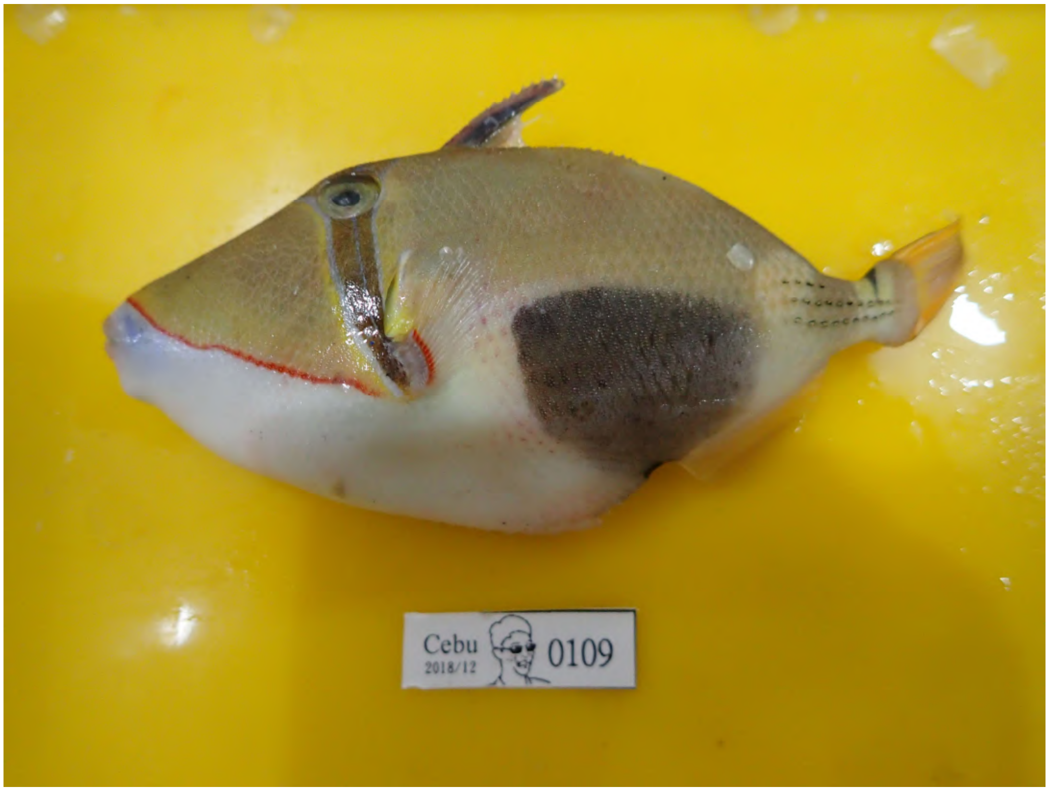

DOS 06613-1, *Rhinecanthus verrucosus*, OR113804.

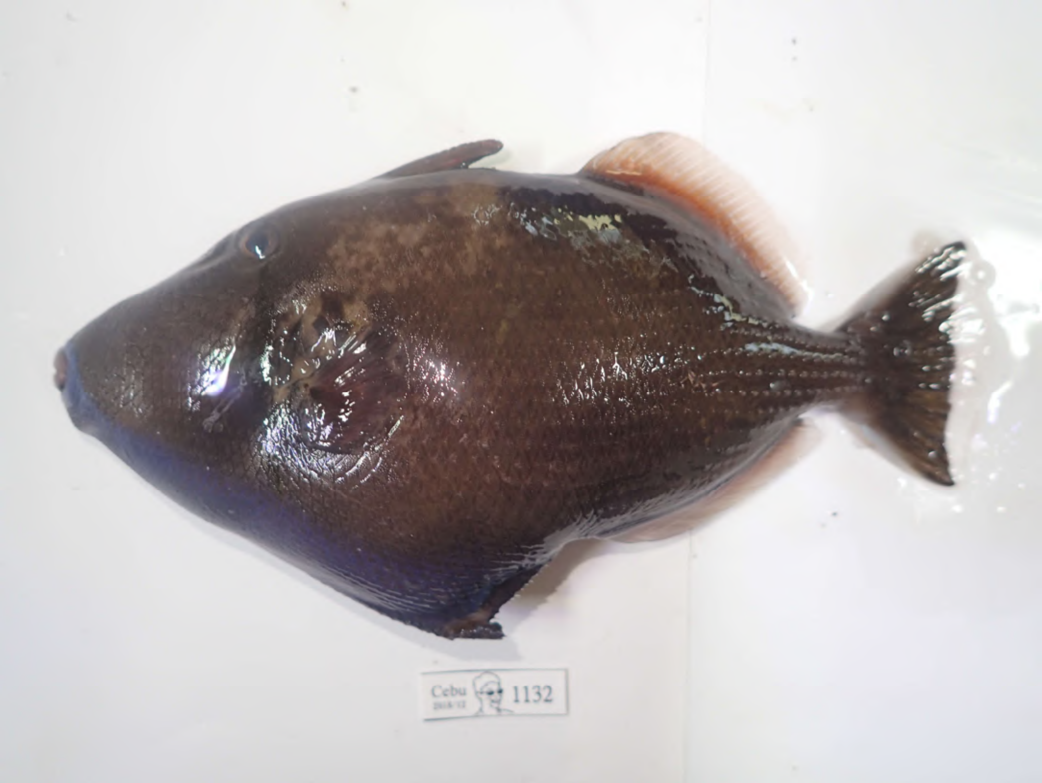

DOS 06614, *Sufflamen chrysopterum*, OR113805.

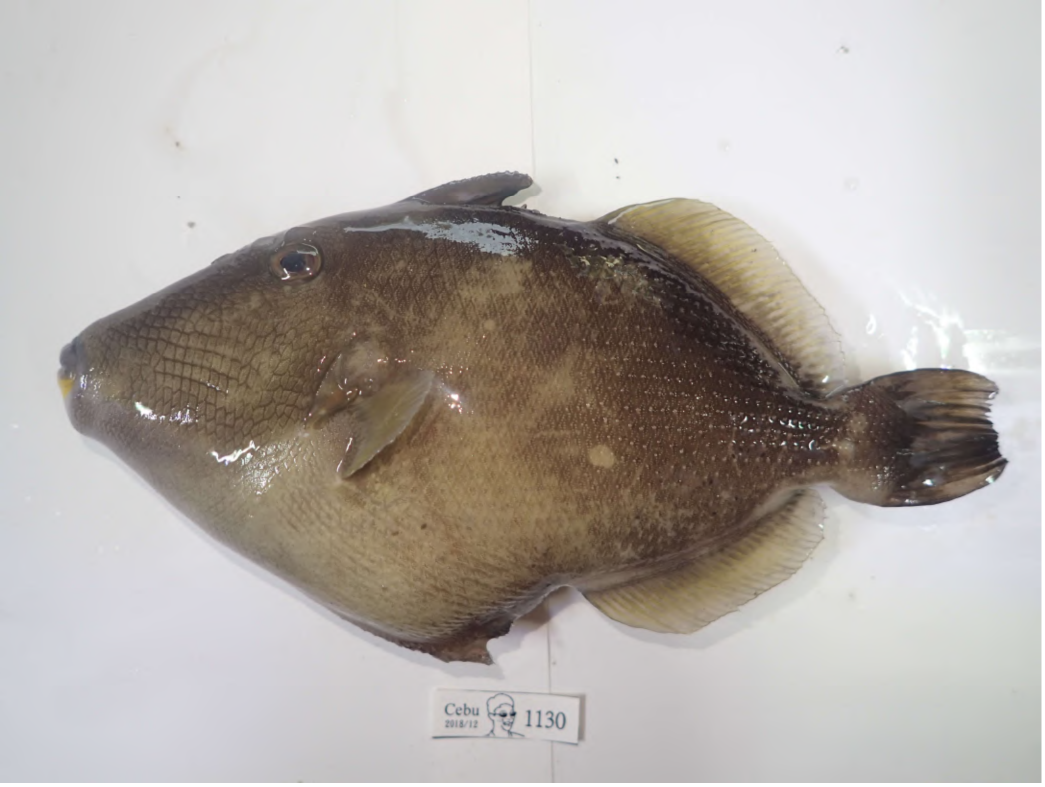

DOS 06615, *Sufflamen fraenatum*, OR113806.

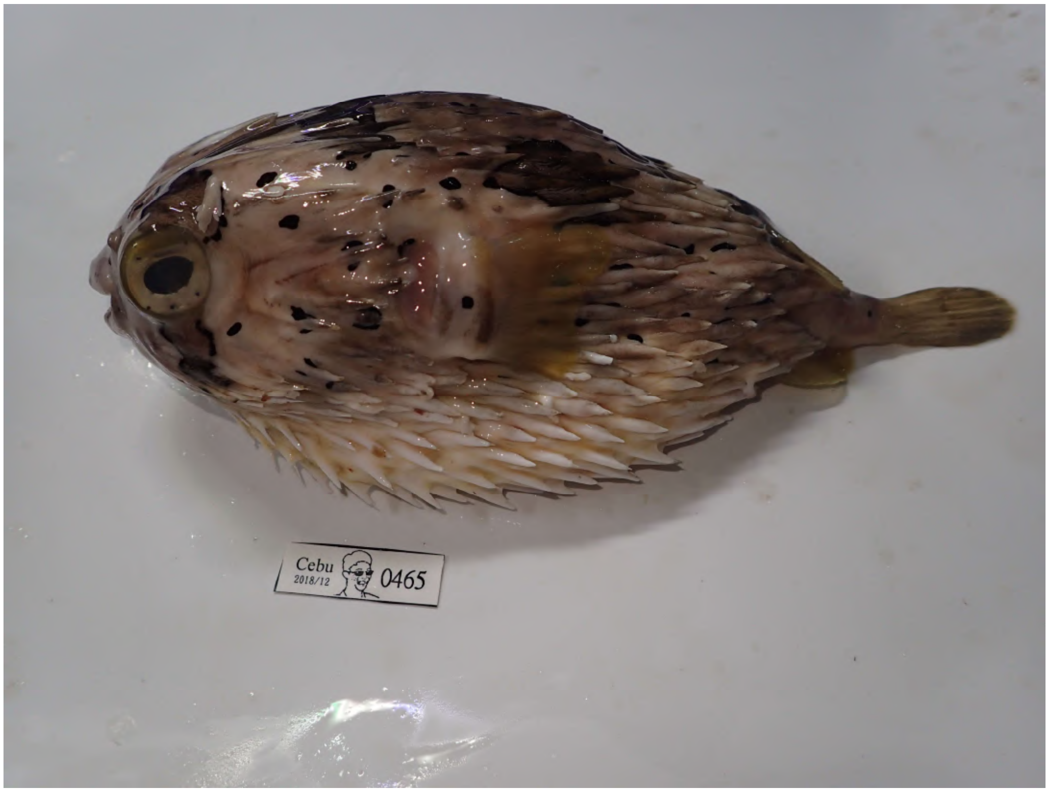

DOS 06685, *Diodon holocanthus*, OR113875. (specimen not preserved)

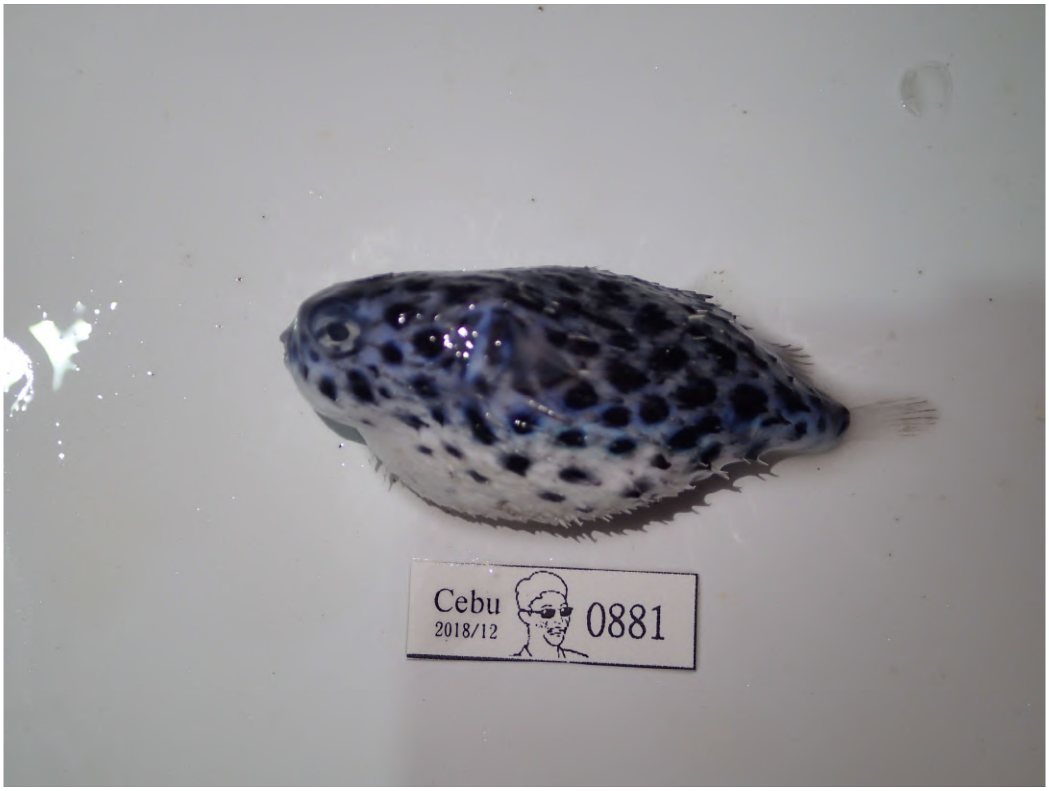

DOS 06686, *Diodon liturosus*,OR113876.

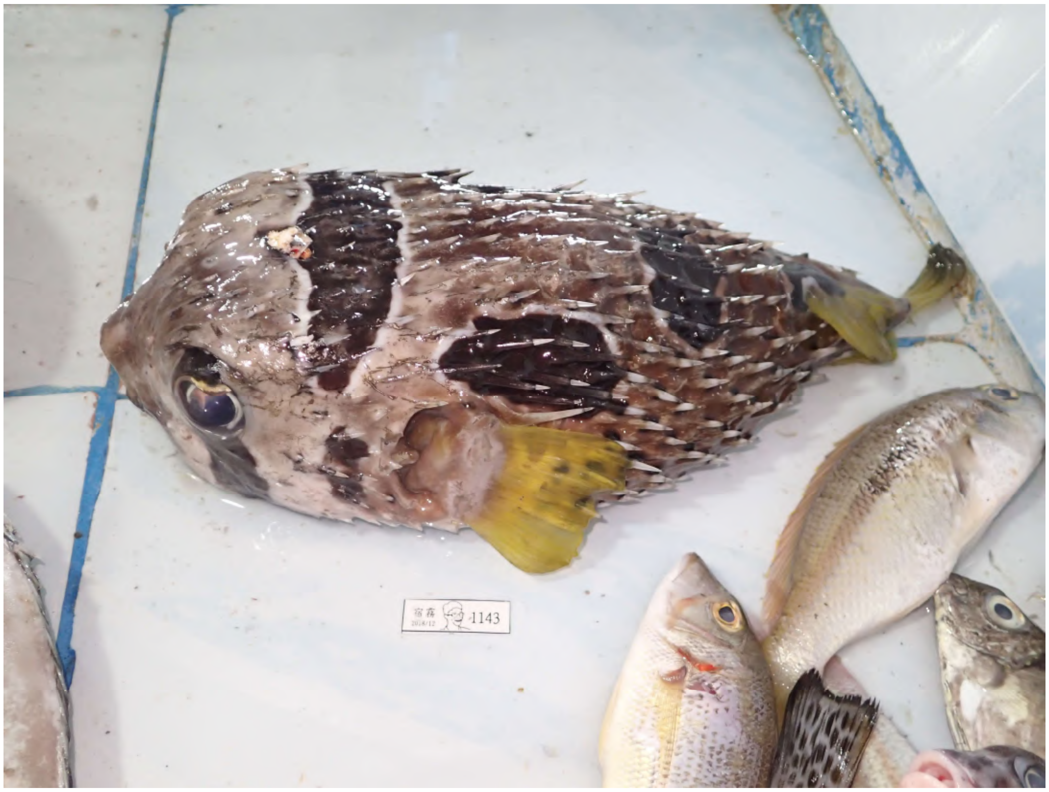

DOS 06687, *Diodon liturosus*, OR113877. (specimen not preserved)

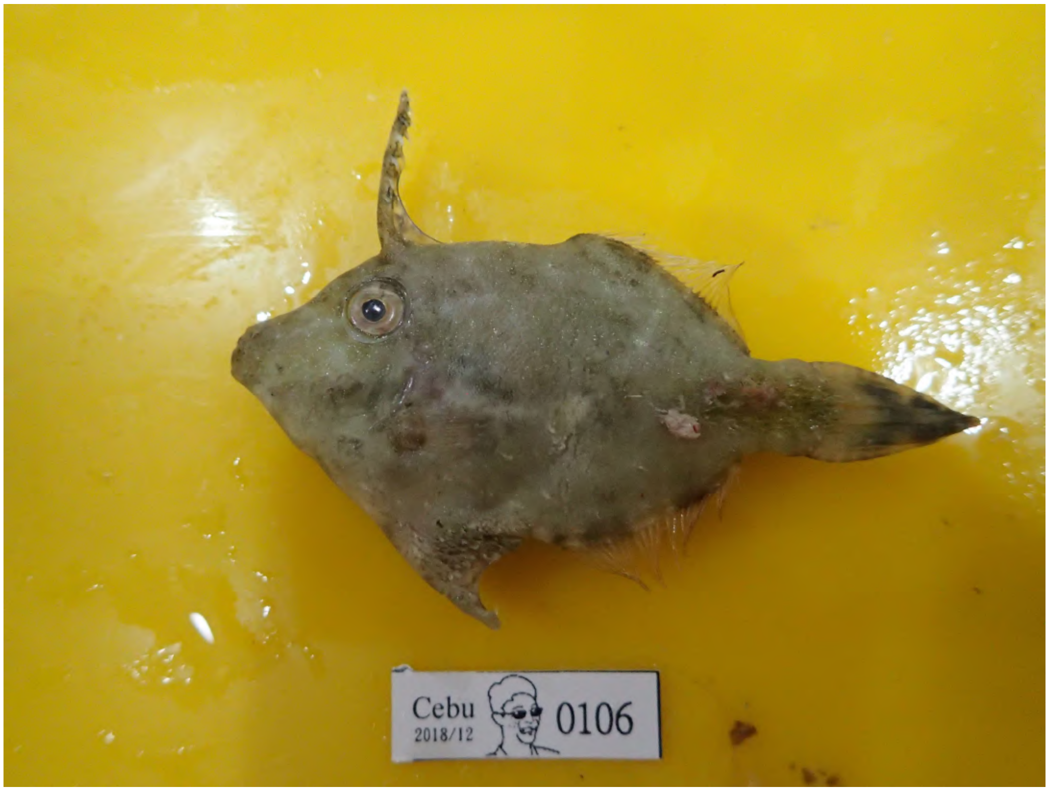

DOS 06795-1, *Acreichthys tomentosus*, OR113977. (specimen not preserved)

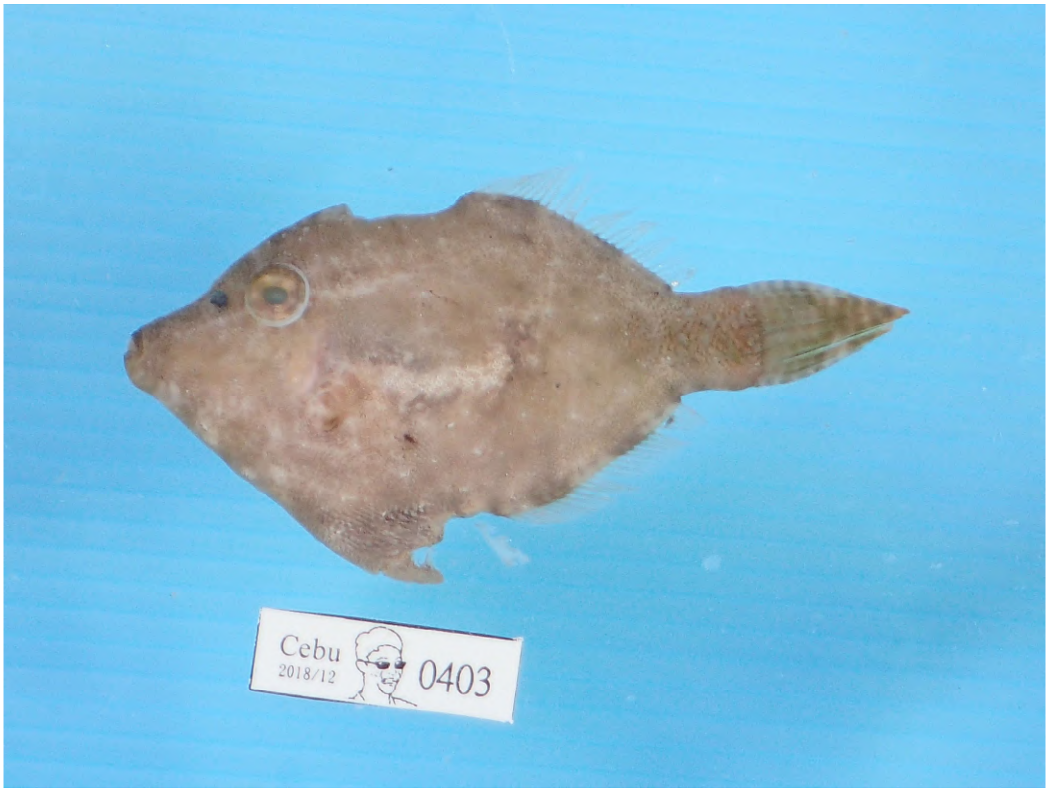

DOS 06803-1, *Acreichthys tomentosus*, OR113985.

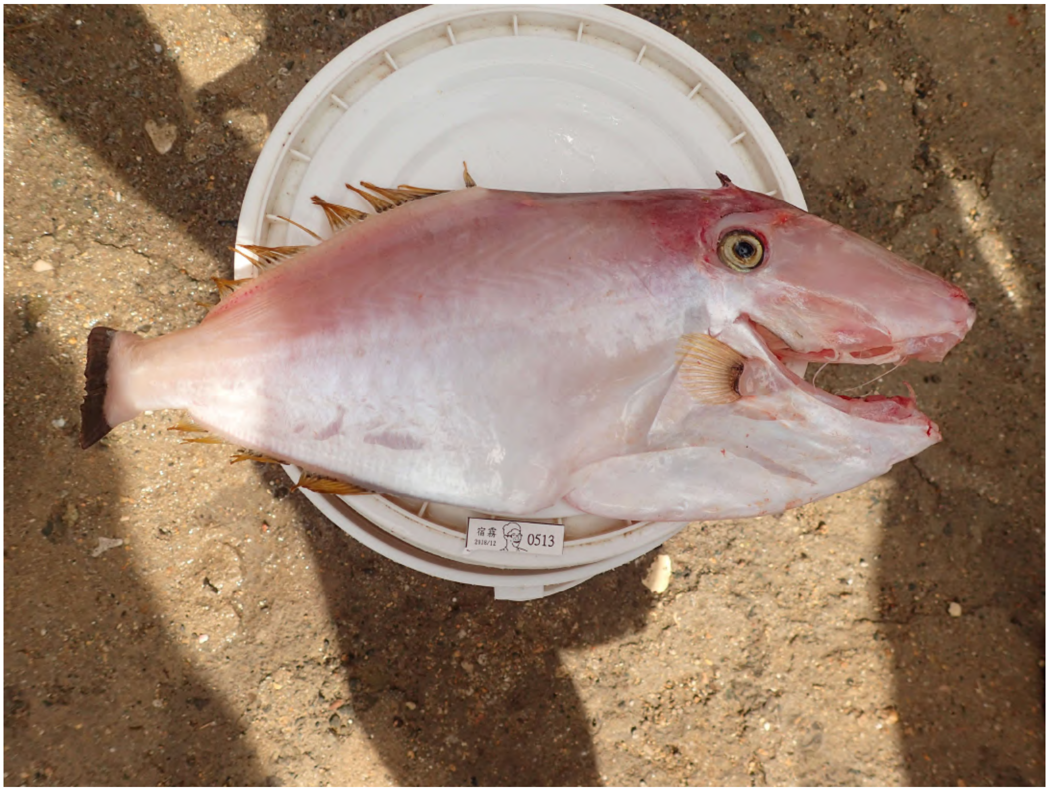

DOS 06801, *Aluterus monoceros*, OR113983. (specimen not preserved)

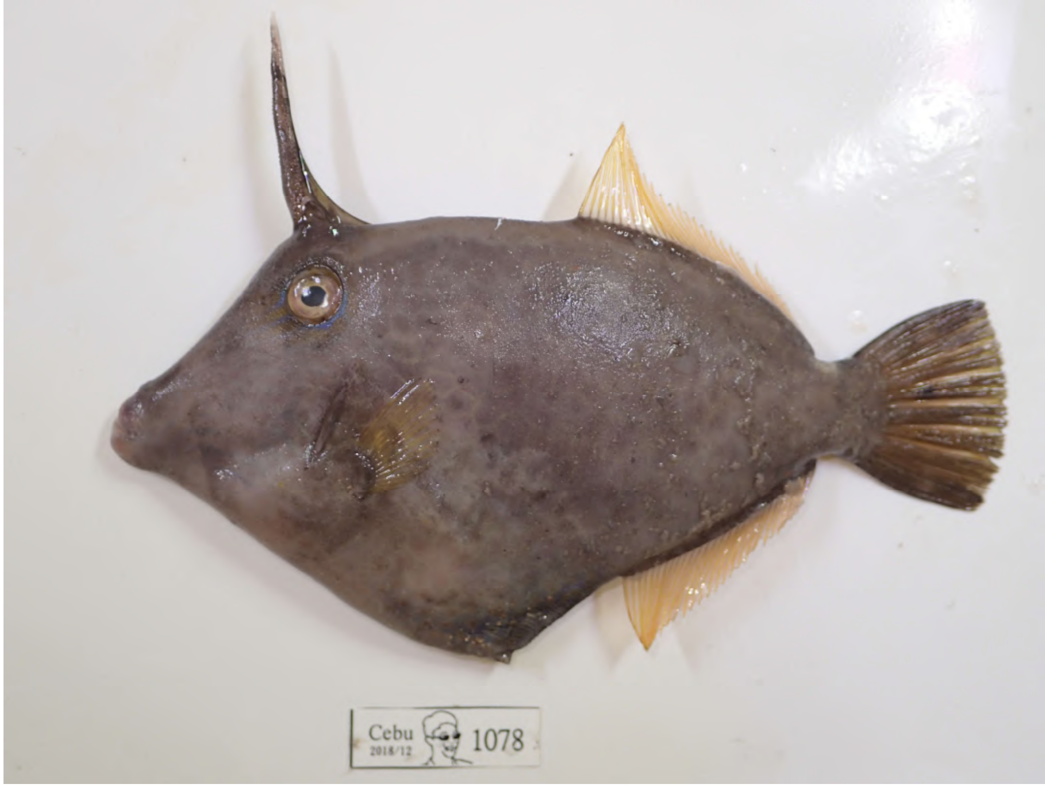

DOS 06796, *Cantherhines pardalis*, OR113978.

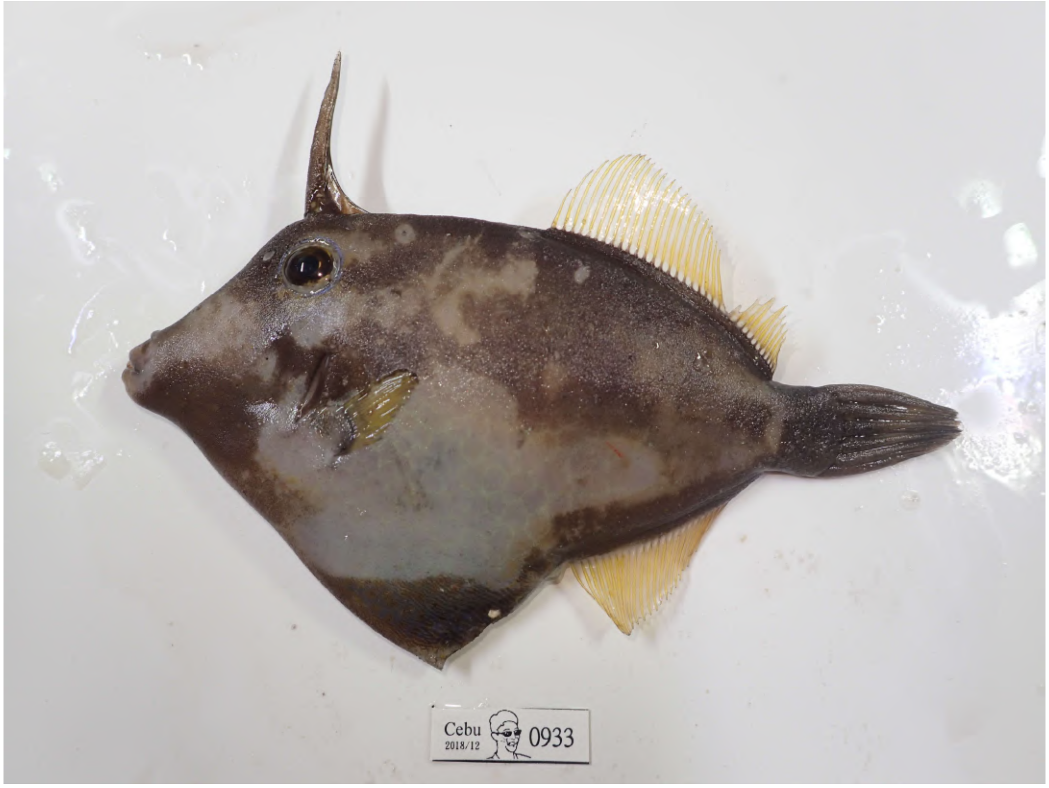

DOS 06802-1, *Cantherhines pardalis*, OR113984. (specimen not preserved)

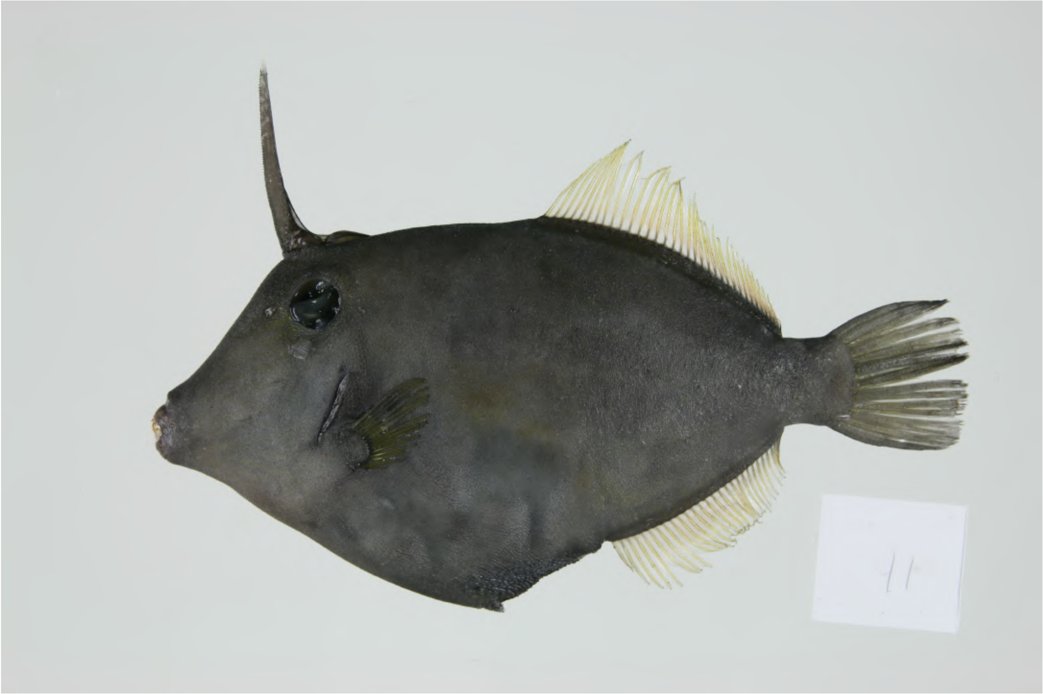

DOS 08692, *Cantherhines pardalis*, OR114277.

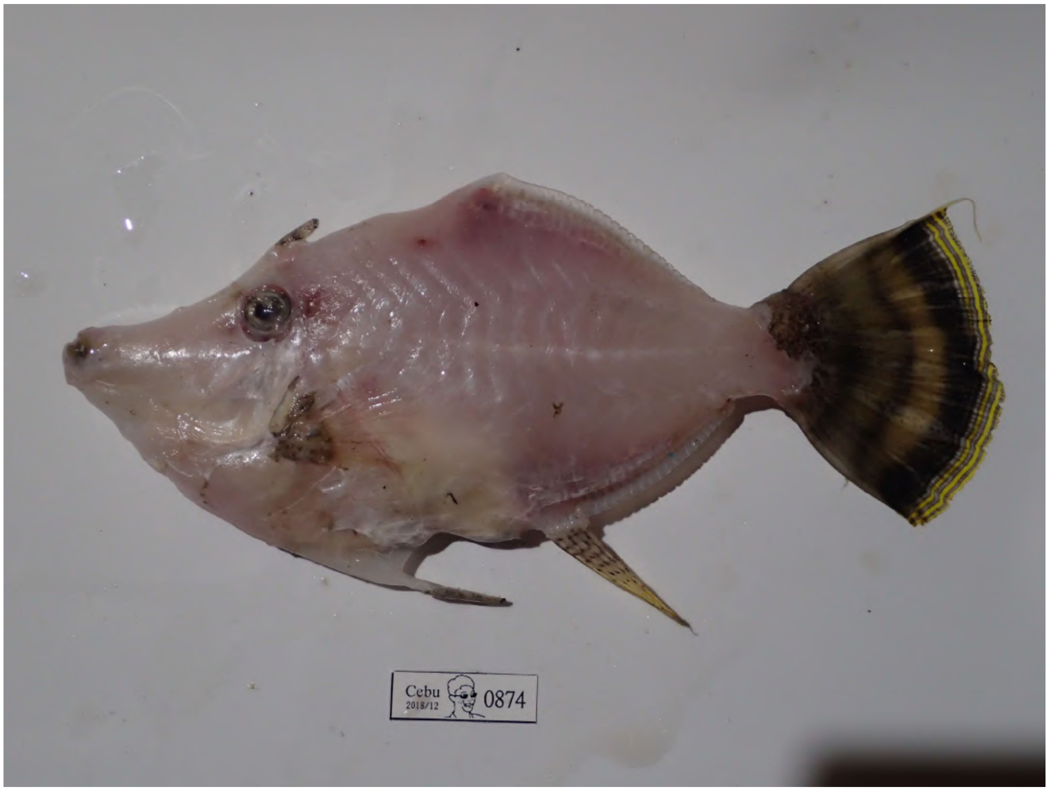

DOS 06799, *Monacanthus chinensis*, OR113981.

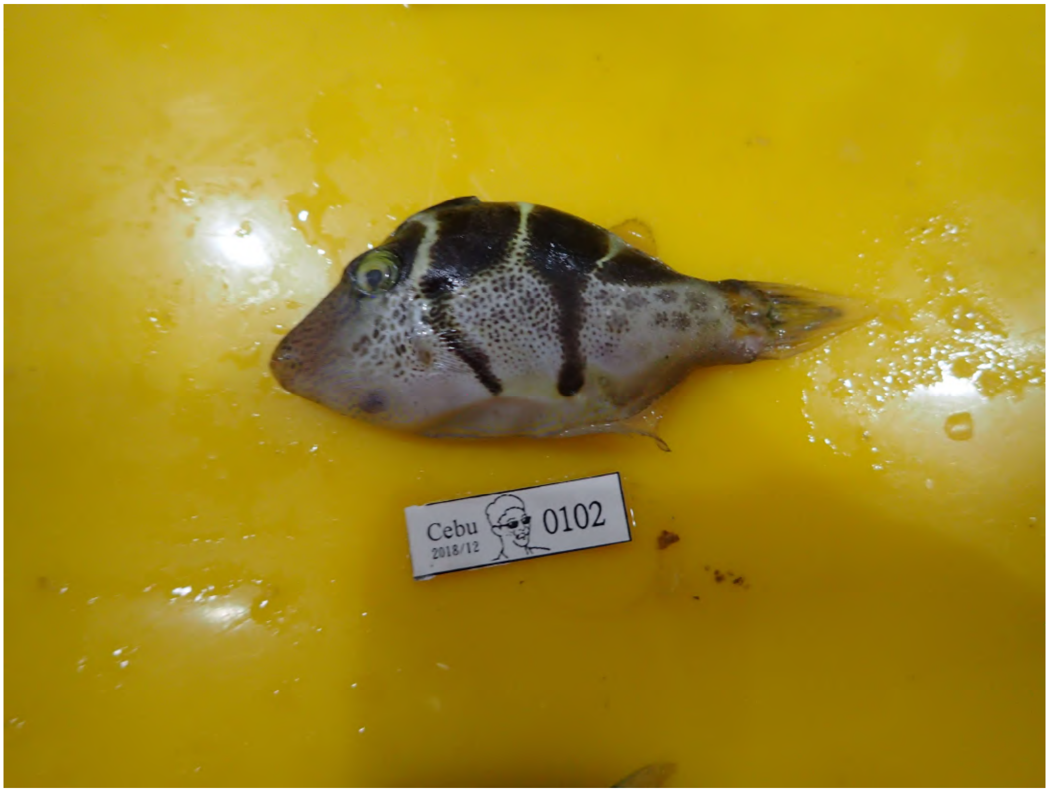

DOS 06986, *Paraluteres prionurus*, OR114155.

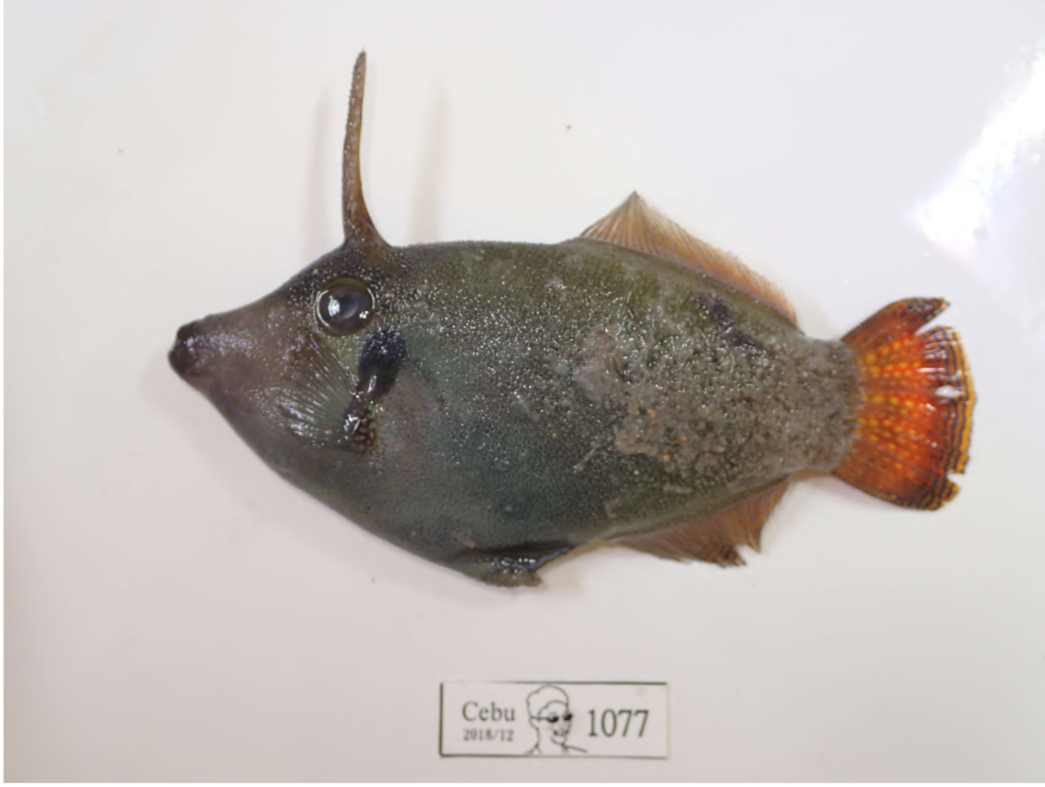

DOS 06798, *Pervagor janthinosoma*, OR113980.

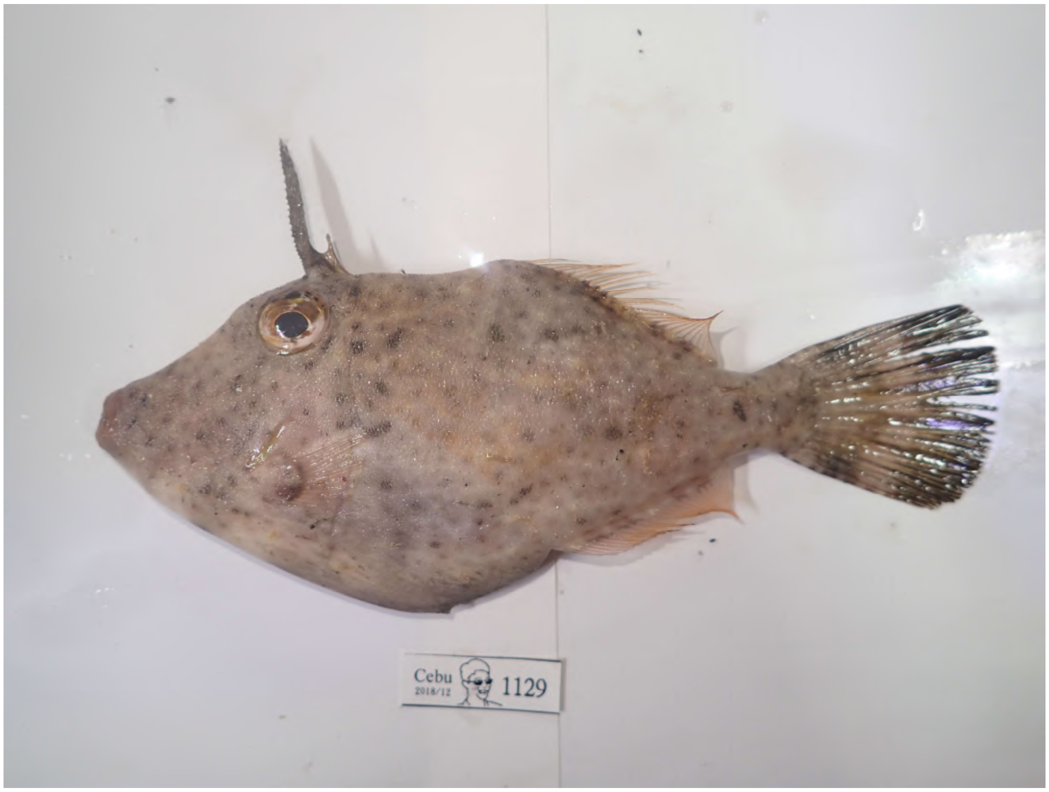

DOS 06797-2, *Pseudomonacanthus macrurus*, OR113979.

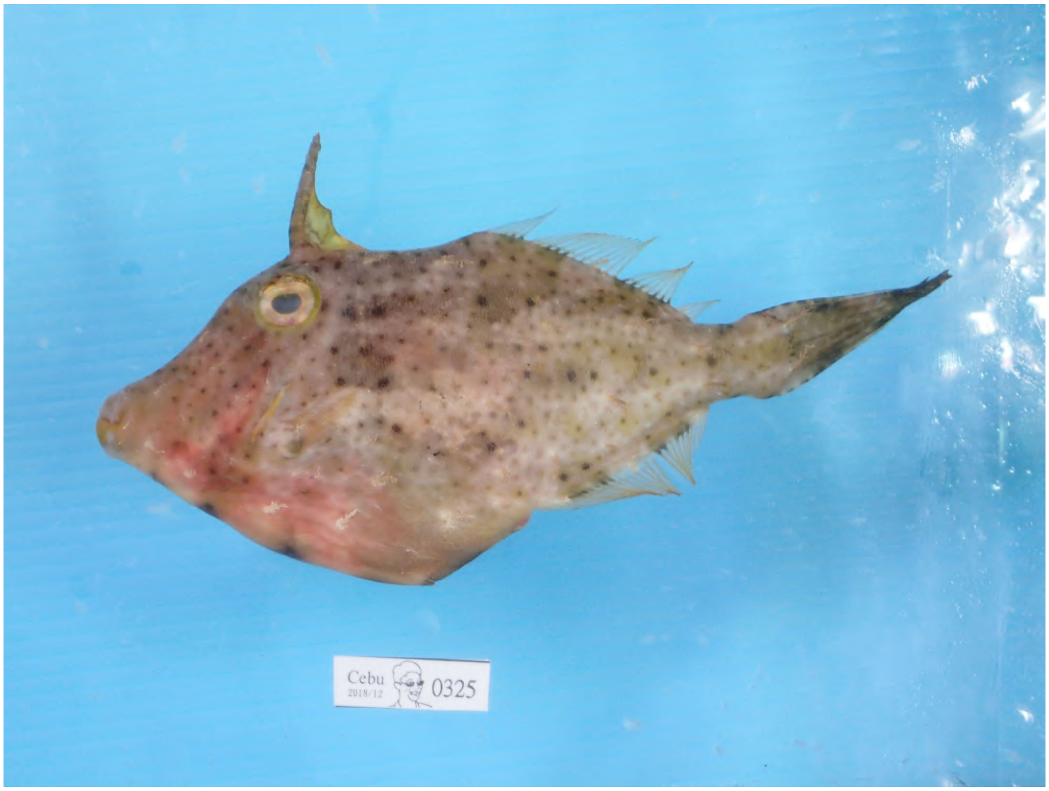

DOS 06800-1, *Pseudomonacanthus macrurus*, OR113982. (specimen not preserved)

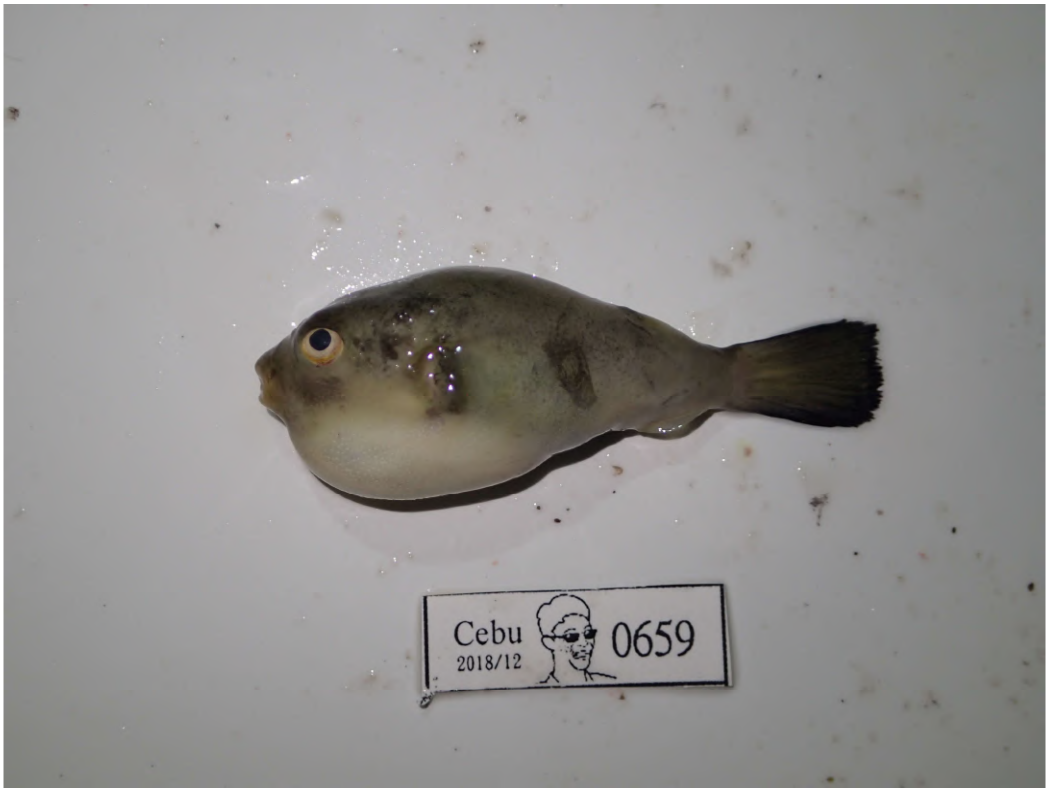

DOS 06982, *Arothron immaculatus*, OR114152.

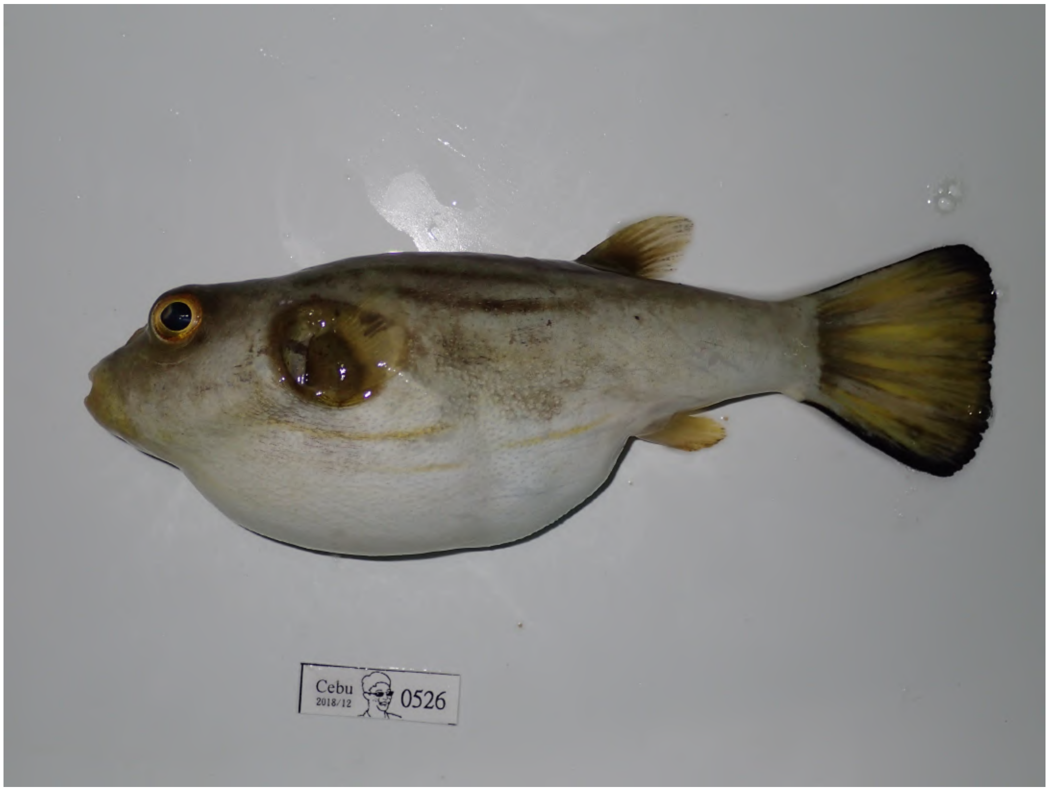

DOS 06983, *Arothron manilensis*, OR114153.

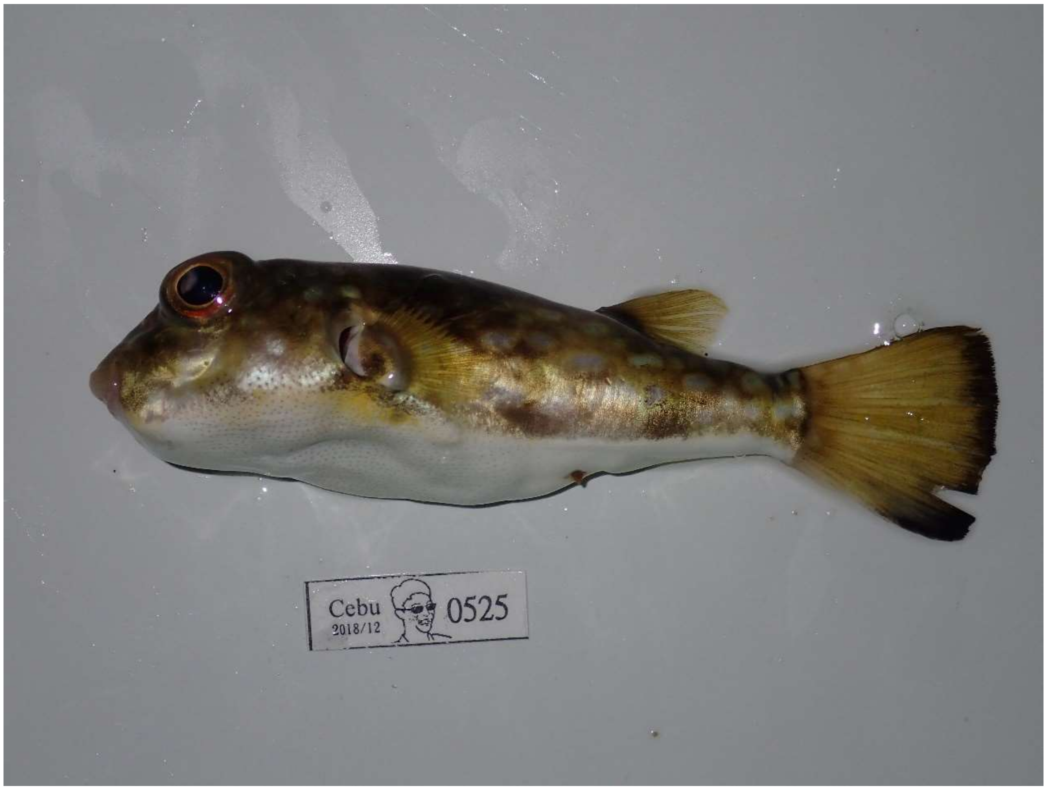

DOS 06984, *Chelonodontops patoca*, OR114154.

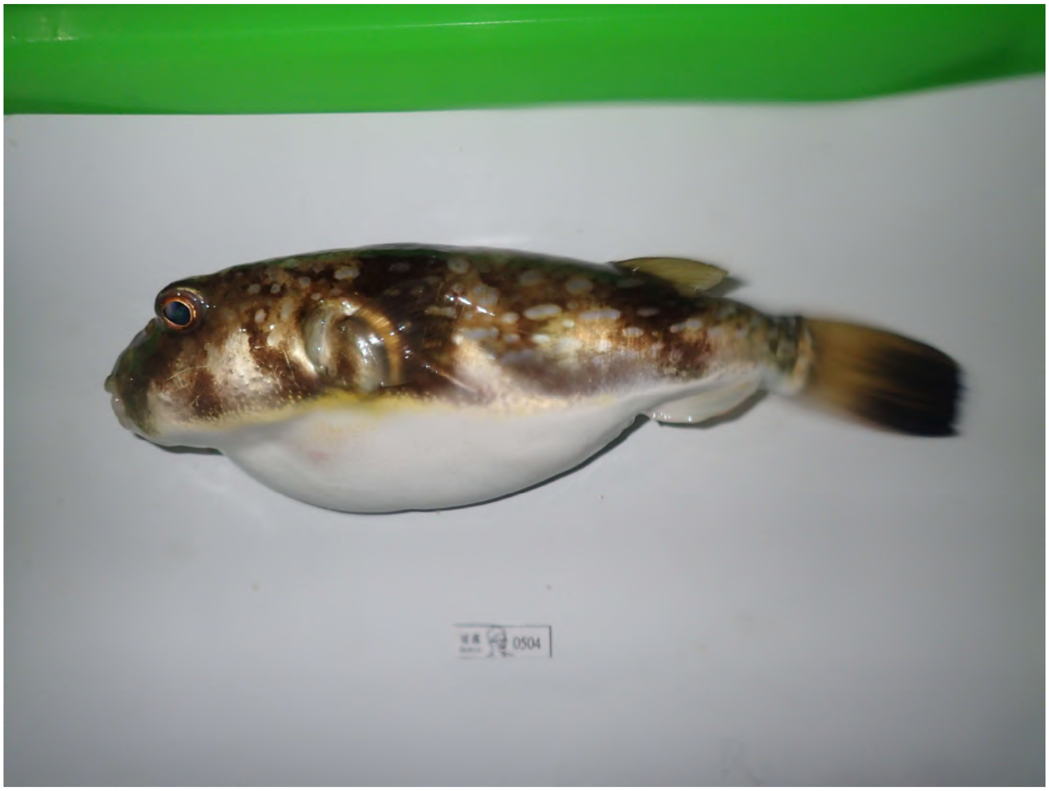

DOS 06987, *Chelonodontops patoca*, OR114156. (specimen not preserved)

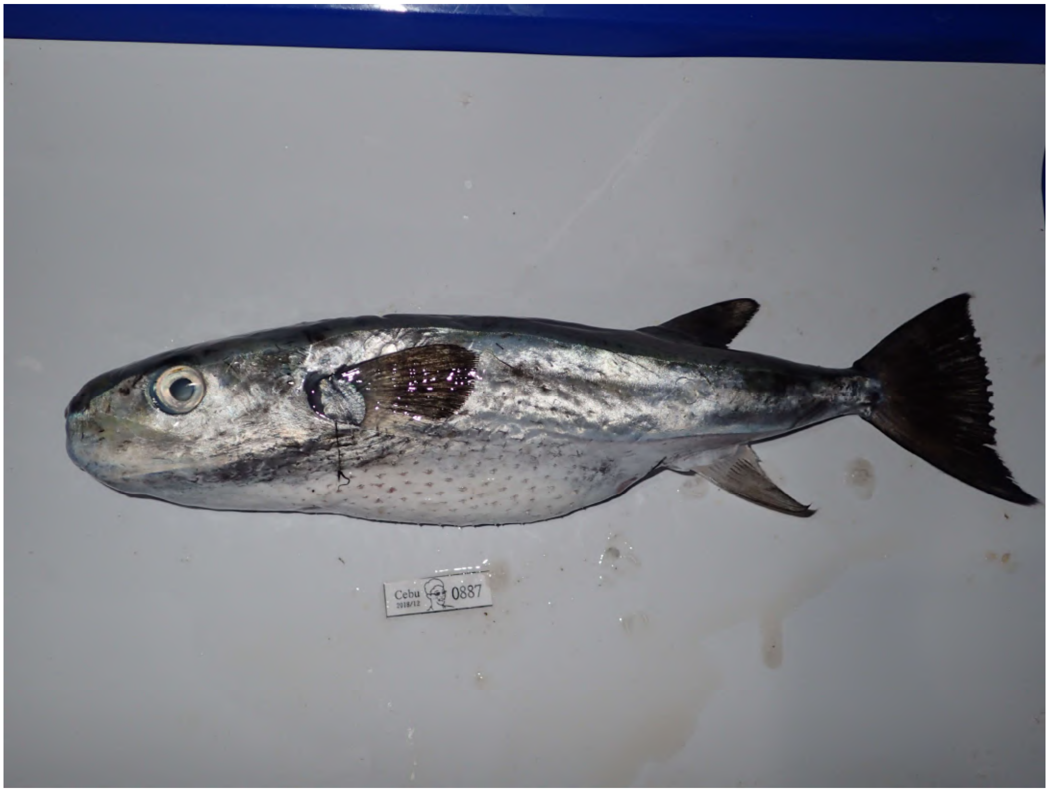

DOS 06988, *Lagocephalus lagocephalus*, OR114157.

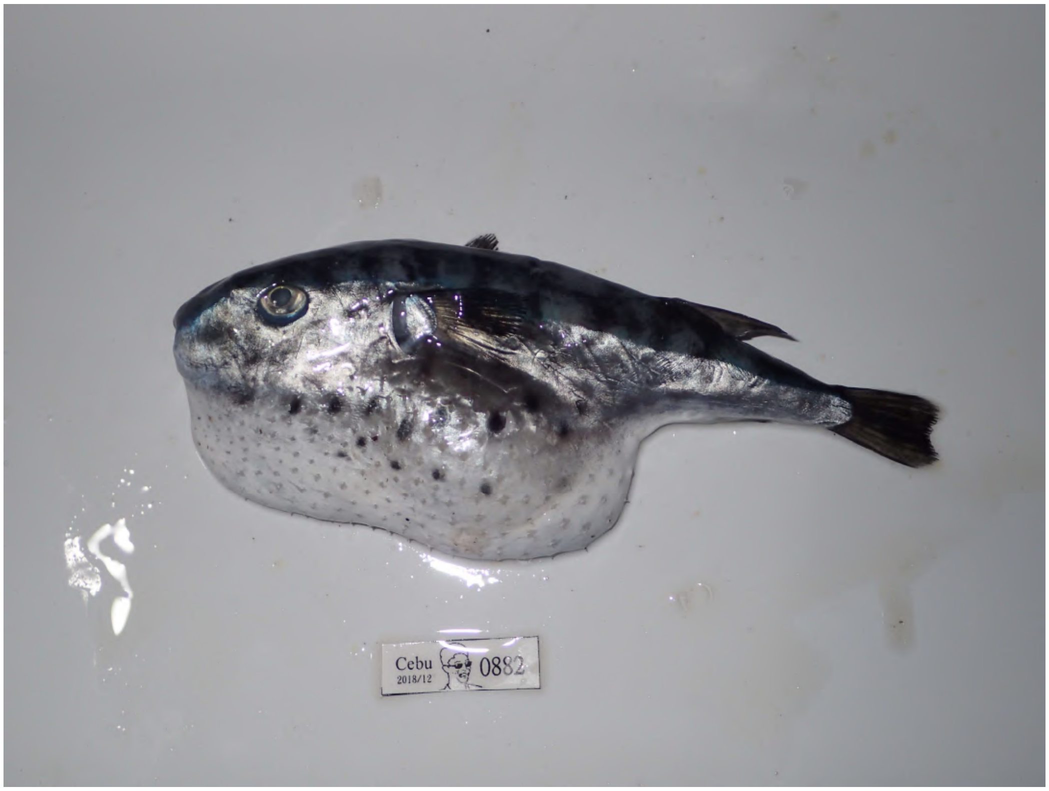

DOS 06989-1, *Lagocephalus lagocephalus*, OR114158.

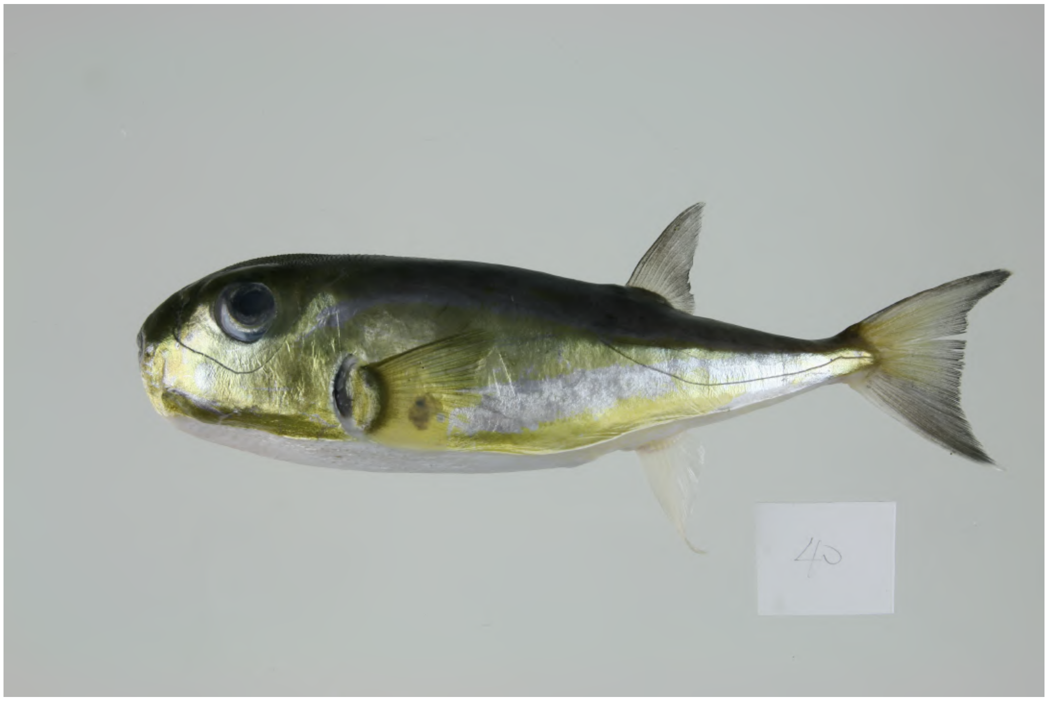

DOS 08693-1, *Lagocephalus spadiceus*, OR114278.

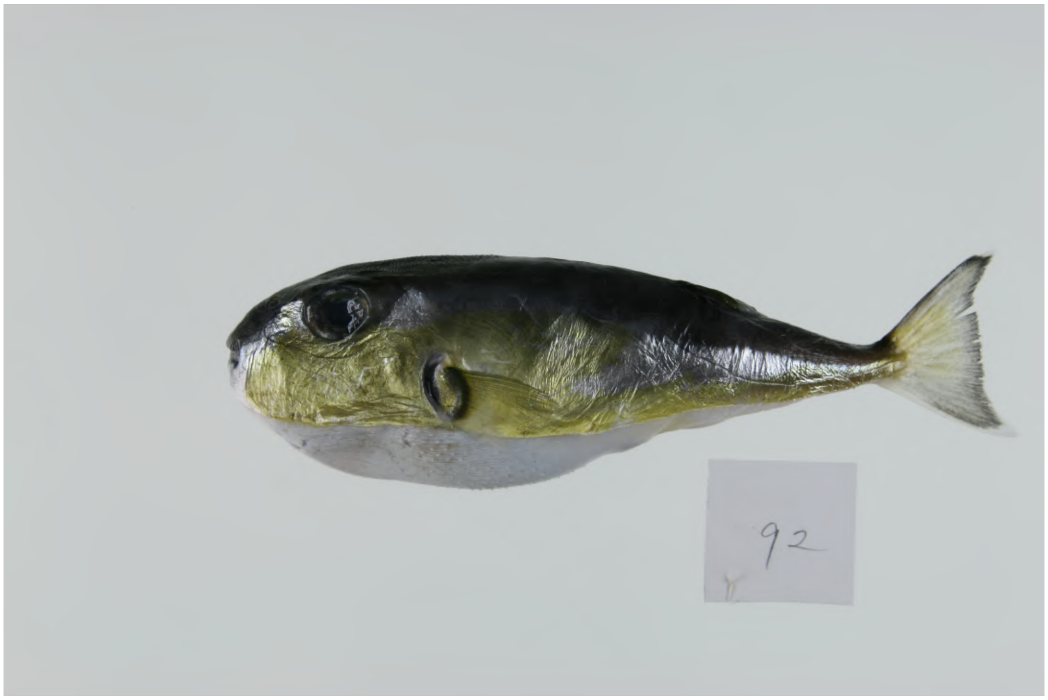

DOS 08693-2, *Lagocephalus spadiceus*, OR114279.

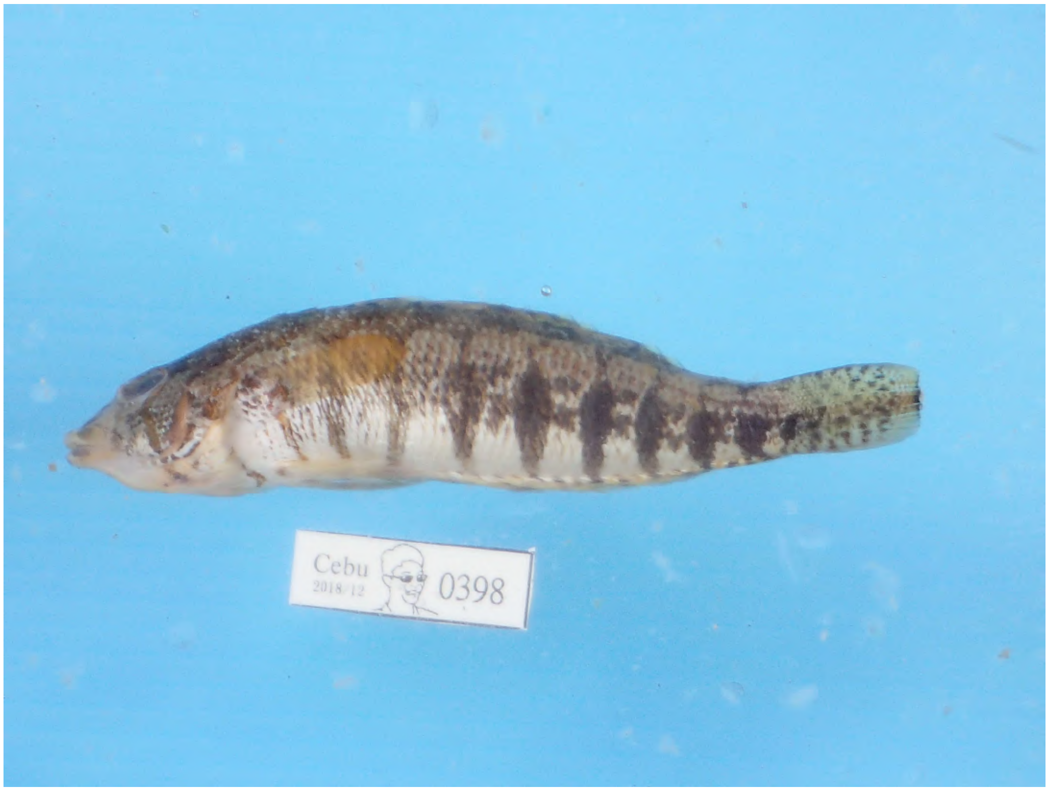

DOS 06867-2, *Parapercis cylindrica*, OR114042.

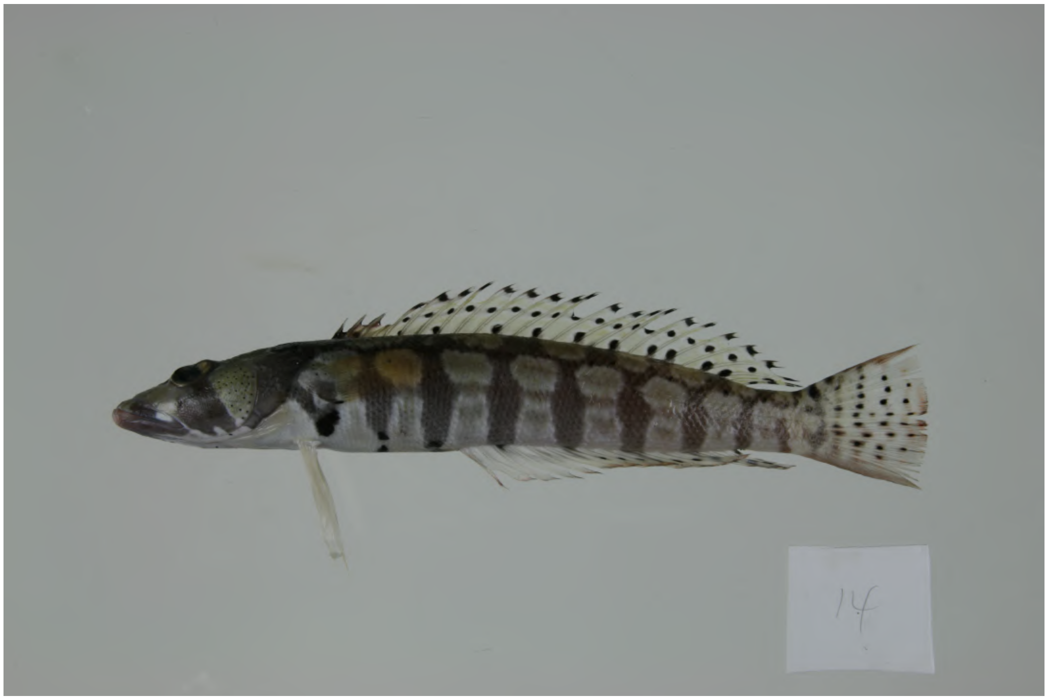

DOS 08694, *Parapercis tetracantha*, OR114280.

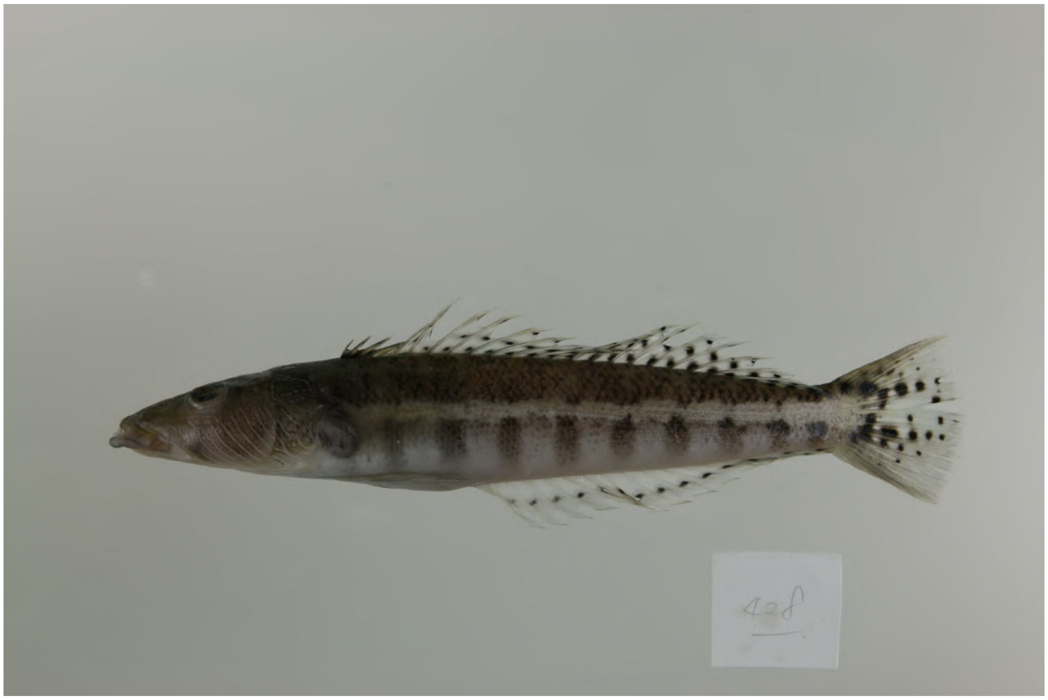

DOS 08695, *Parapercis xanthozona*, OR114281.

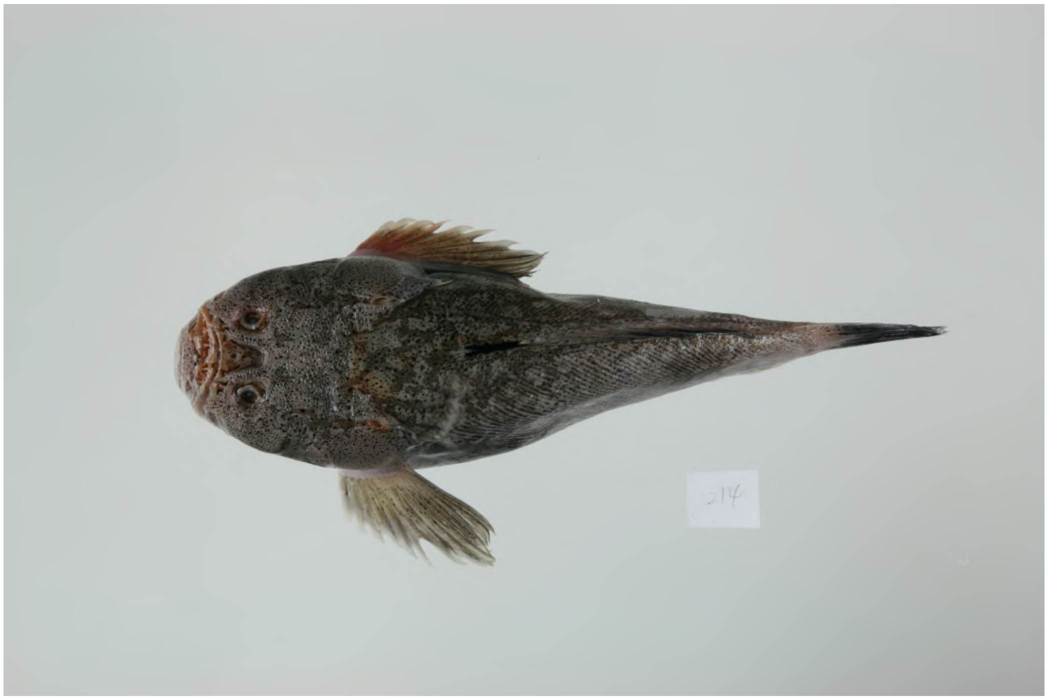

DOS 08696-1, *Uranoscopus bicinctus*, OR114282.

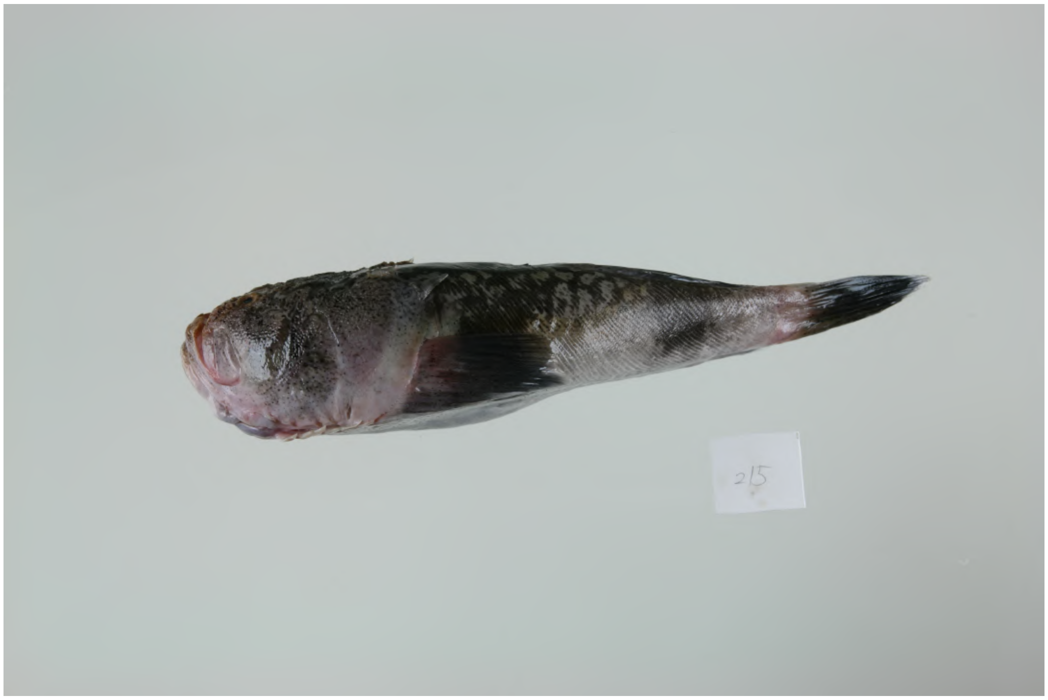

DOS 08696-2, *Uranoscopus bicinctus*, OR114283.
