# Supplementary material for: DNA barcoding of marine teleost fishes (Teleostei) in Cebu, the Philippines, a biodiversity hotspot of the coral triangle
Source: Sci Rep. 2023 Sep 8;13:14867. doi: 10.1038/s41598-023-41832-9 (PMC10491795; doi:10.1038/s41598-023-41832-9)
Supplement: Supplementary file 2 — Supplementary Figures. [file 41598_2023_41832_MOESM2_ESM.pdf]

DNA barcoding of marine teleost fishes (Teleostei) in Cebu, the Philippines, a biodiversity hotspot of the coral triangle

Wen-Chien Huang[1,2,**], Florence Chan Evacitas[3,**], Rodulf Anthony Balisco[4,5], Cleto L. Nañola Jr.[6], Tak-Kei Chou[4], Wei-Cheng Jhuang[1,2], Chih-Wei Chang[7], Kang-Ning Shen[7], Kwang-Tsao Shao[4,8], Te-Yu Liao[4,*]

[1]Doctoral Degree Program in Marine Biotechnology, National Sun Yat-sen University, Kaohsiung, Taiwan
[2]Doctoral Degree Program in Marine Biotechnology, Academia Sinica, Taipei, Taiwan
[3]Department of Biology and Environmental Science, University of the Philippines Cebu, Cebu City, Philippines
[4]Department of Oceanography, National Sun Yat-sen University, Kaohsiung, Taiwan
[5]College of Fisheries and Aquatic Sciences, Western Philippines University, Puerto Princesa City, Palawan, Philippines
[6]Department of Biological Sciences and Environmental Studies, University of the Philippines Mindanao, Davao City, Philippines
[7]Marine Ecology and Conservation Research Center, National Academy of Marine Research, Kaohsiung, Taiwan
[8]Biodiversity Research Center, Academia Sinica, Taipei, Taiwan

**Corresponding author*: swp0117@gmail.com
**Authors contributed equally

Supplementary Figure S2. Neighbor-joining tree based on 538 *COI* sequences using Tamura-Nei + Γ model with 1,000 bootstrap replications. Numerals beside the internal branches indicate bootstrap values, and values below 50 are not shown.

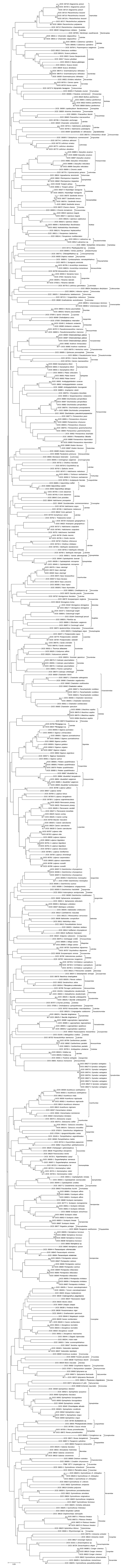

Supplementary Figure S3. Maximum likelihood (ML) trees for *COI* sequences of the 19 non-monophyletic genera in 12 families (showed in Table 4). Trees were built using best-suggested models, bootstraps with 1,000 replications, and outgroup sequences of available closest taxa from our data set. Numerals beside the internal branches indicate bootstrap values, and values below 50 are not shown.

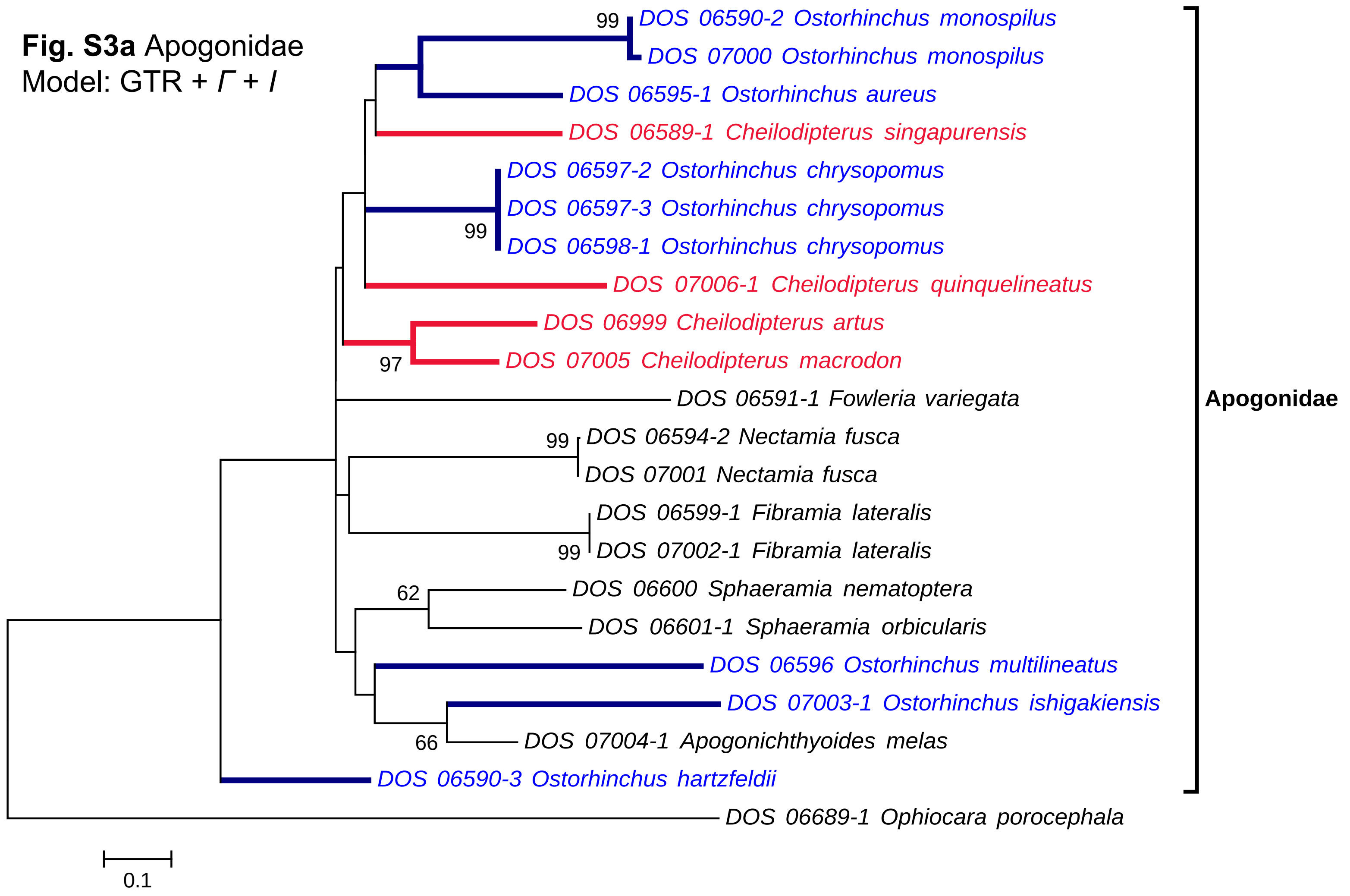

Fig. S3a Apogonidae
Model: GTR + *Γ* + *I*
99
DOS 06590-2 Ostorhinchus monospilus
DOS 07000 Ostorhinchus monospilus
DOS 06595-1 Ostorhinchus aureus
DOS 06589-1 Cheilodipterus singapurensis
DOS 06597-2 Ostorhinchus chrysopomus
DOS 06597-3 Ostorhinchus chrysopomus
99
DOS 06598-1 Ostorhinchus chrysopomus
DOS 07006-1 Cheilodipterus quinquelineatus
DOS 06999 Cheilodipterus artus
97
DOS 07005 Cheilodipterus macrodon
DOS 06591-1 Fowleria variegata
99
DOS 06594-2 Nectamia fusca
DOS 07001 Nectamia fusca
DOS 06599-1 Fibramia lateralis
99
DOS 07002-1 Fibramia lateralis
62
DOS 06600 Sphaeramia nematoptera
DOS 06601-1 Sphaeramia orbicularis
DOS 06596 Ostorhinchus multilineatus
DOS 07003-1 Ostorhinchus ishigakiensis
66
DOS 07004-1 Apogonichthyoides melas
DOS 06590-3 Ostorhinchus hartzfeldii
DOS 06689-1 Ophiocara porocephala
Apogonidae
0.1

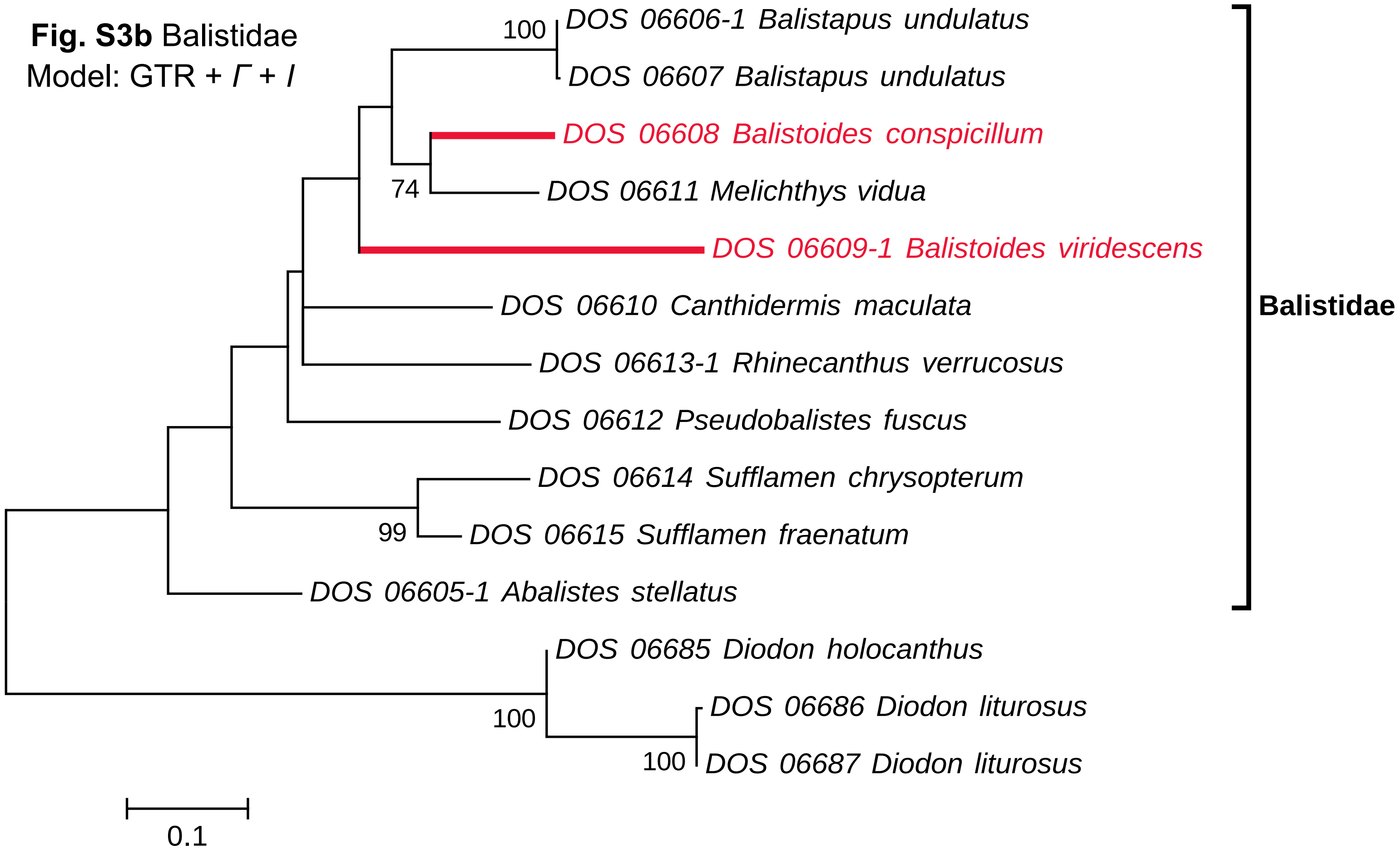
Fig. S3b Balistidae
Model: GTR + Γ + I
100
DOS 06606-1 Balistapus undulatus
DOS 06607 Balistapus undulatus
DOS 06608 Balistoides conspicillum
74
DOS 06611 Melichthys vidua
DOS 06609-1 Balistoides viridescens
DOS 06610 Canthidermis maculata
DOS 06613-1 Rhinecanthus verrucosus
DOS 06612 Pseudobalistes fuscus
DOS 06614 Sufflamen chrysopterum
99
DOS 06615 Sufflamen fraenatum
DOS 06605-1 Abalistes stellatus
Balistidae
DOS 06685 Diodon holocanthus
100
DOS 06686 Diodon liturosus
100
DOS 06687 Diodon liturosus
0.1

Fig. S3c Blenniidae
Model: GTR + Γ + I

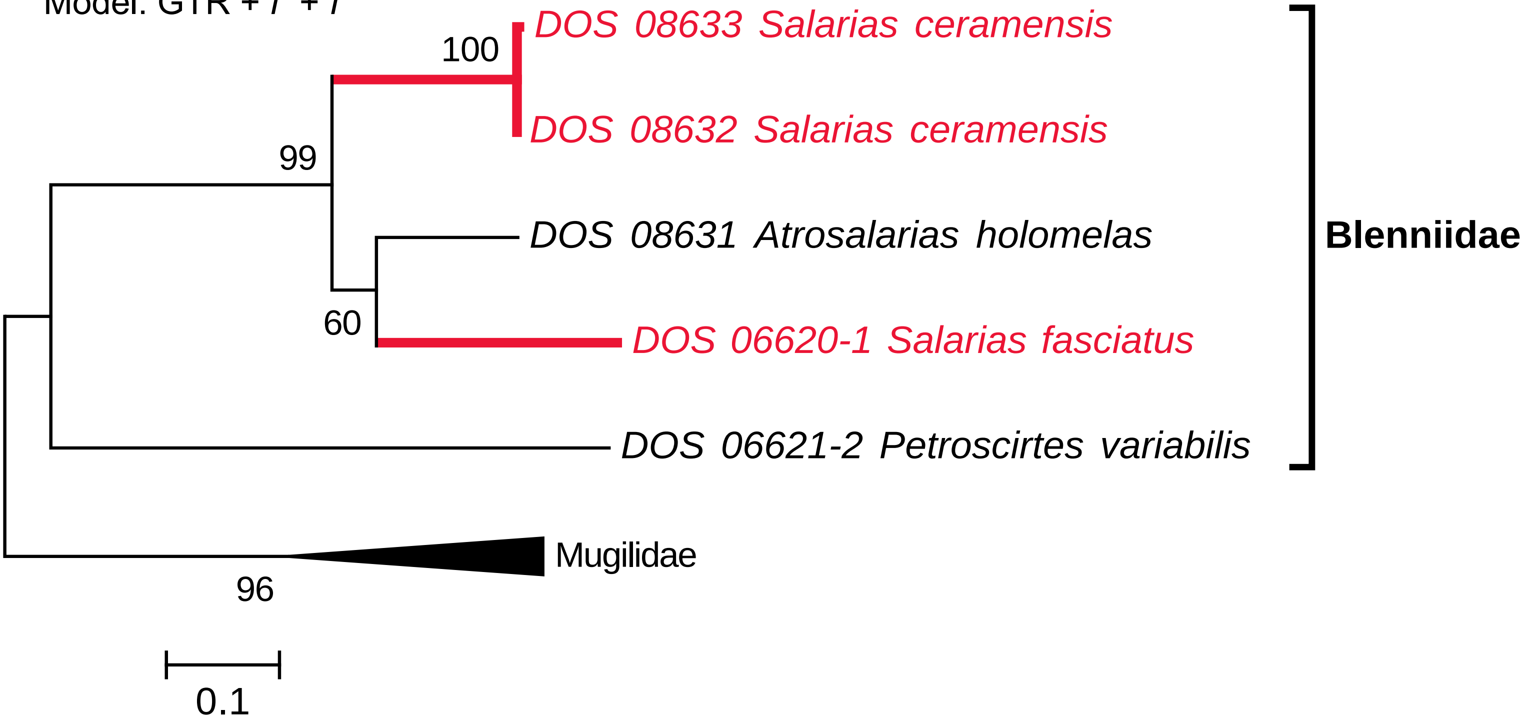

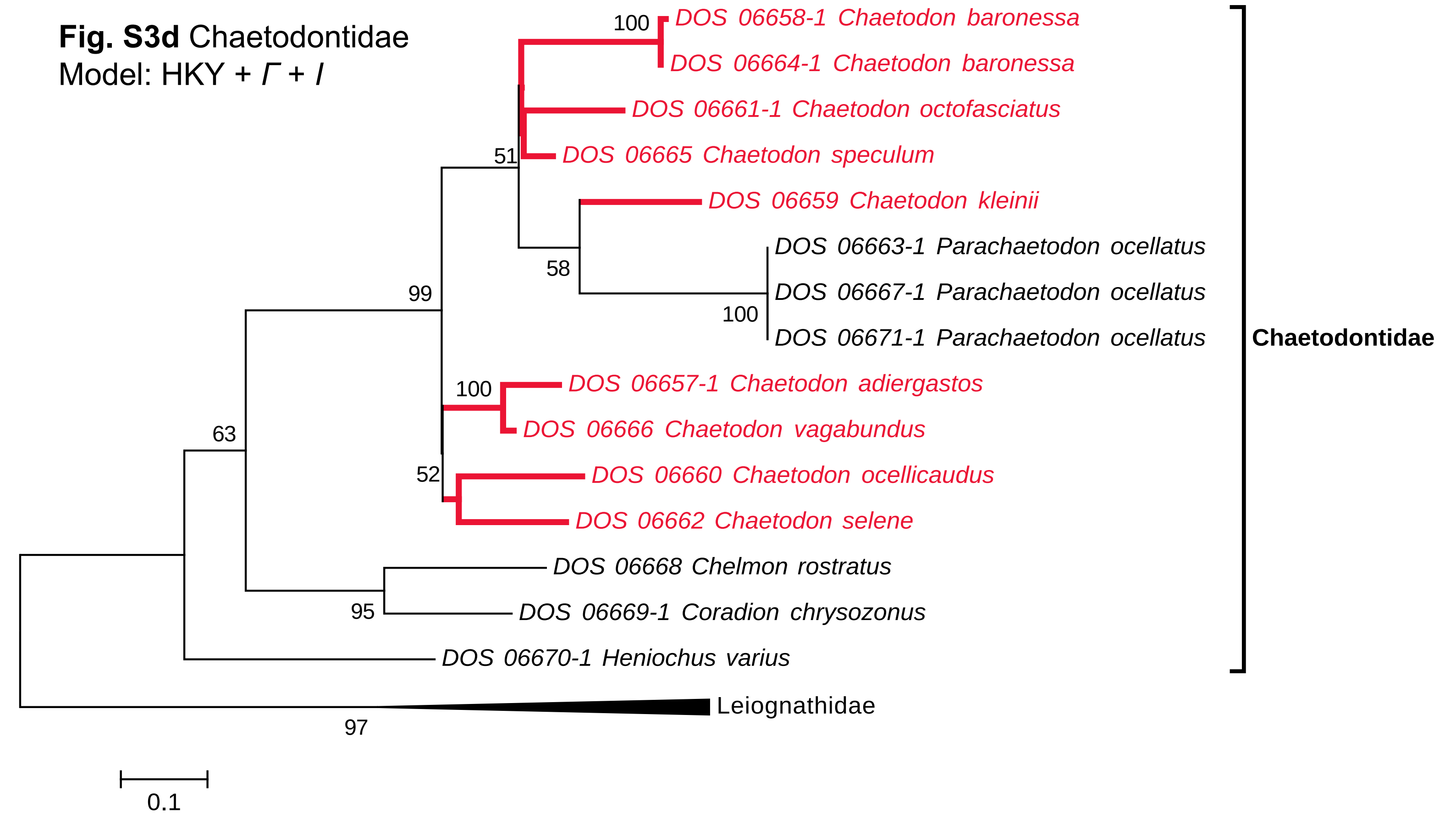

Fig. S3d Chaetodontidae
Model: HKY + Γ + I
100
DOS 06658-1 Chaetodon baronessa
DOS 06664-1 Chaetodon baronessa
DOS 06661-1 Chaetodon octofasciatus
51
DOS 06665 Chaetodon speculum
DOS 06659 Chaetodon kleinii
DOS 06663-1 Parachaetodon ocellatus
58
DOS 06667-1 Parachaetodon ocellatus
99
100
DOS 06671-1 Parachaetodon ocellatus
Chaetodontidae
100
DOS 06657-1 Chaetodon adiergastos
63
DOS 06666 Chaetodon vagabundus
52
DOS 06660 Chaetodon ocellicaudus
DOS 06662 Chaetodon selene
DOS 06668 Chelmon rostratus
95
DOS 06669-1 Coradion chrysozonus
DOS 06670-1 Heniochus varius
Leiognathidae
97
0.1

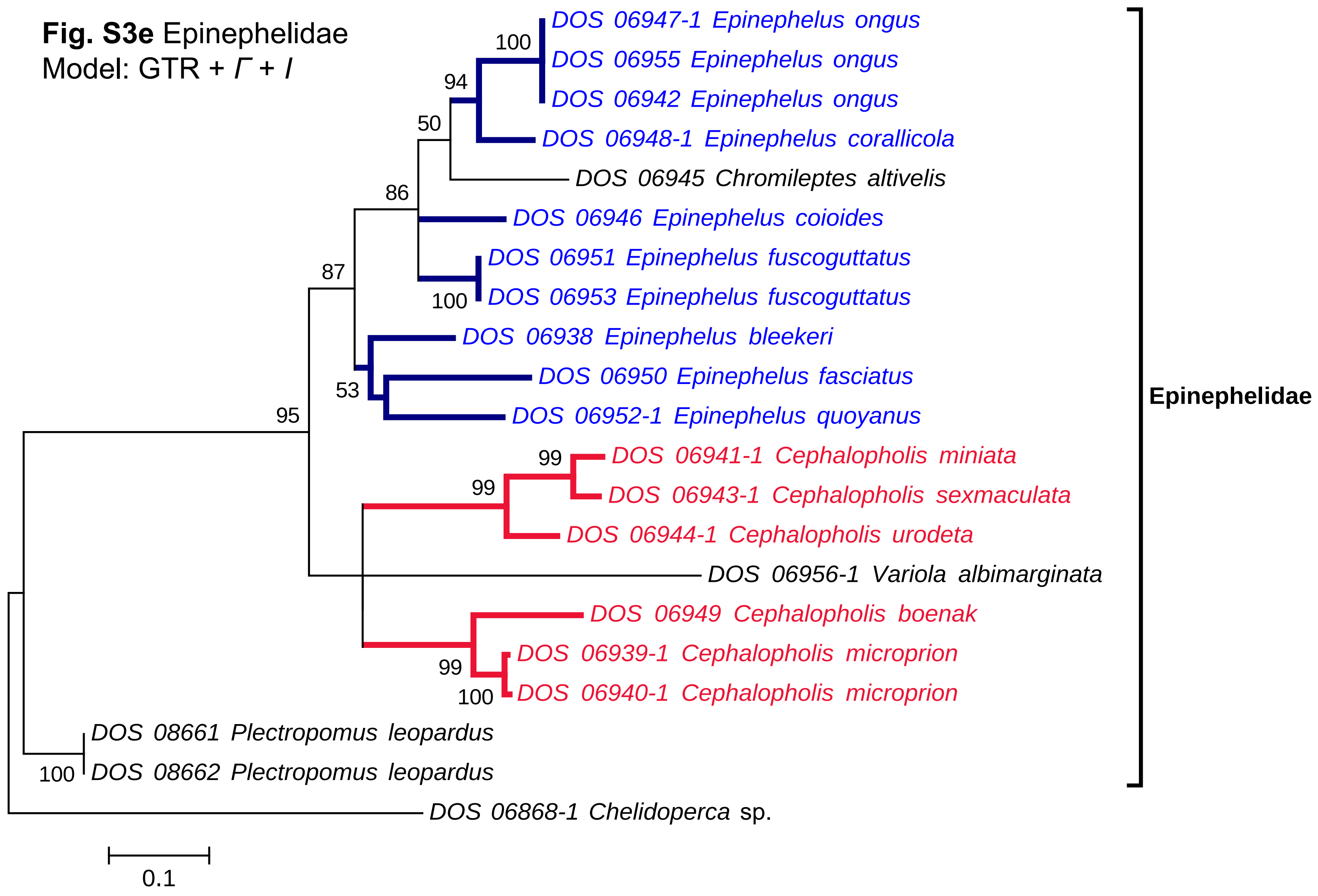
Fig. S3e Epinephelidae
Model: GTR + *Γ* + *I*
DOS 06947-1 Epinephelus ongus
DOS 06955 Epinephelus ongus
DOS 06942 Epinephelus ongus
DOS 06948-1 Epinephelus corallicola
DOS 06945 Chromileptes altivelis
DOS 06946 Epinephelus coioides
DOS 06951 Epinephelus fuscoguttatus
DOS 06953 Epinephelus fuscoguttatus
DOS 06938 Epinephelus bleekeri
DOS 06950 Epinephelus fasciatus
DOS 06952-1 Epinephelus quoyanus
DOS 06941-1 Cephalopholis miniata
DOS 06943-1 Cephalopholis sexmaculata
DOS 06944-1 Cephalopholis urodeta
DOS 06956-1 Variola albimarginata
DOS 06949 Cephalopholis boenak
DOS 06939-1 Cephalopholis microprion
DOS 06940-1 Cephalopholis microprion
DOS 08661 Plectropomus leopardus
DOS 08662 Plectropomus leopardus
DOS 06868-1 Chelidoperca sp.
Epinephelidae
100
94
50
86
100
87
53
95
99
99
99
100
100
0.1

Fig. S3f Gempylidae
Model: GTR + *Γ* + *I*

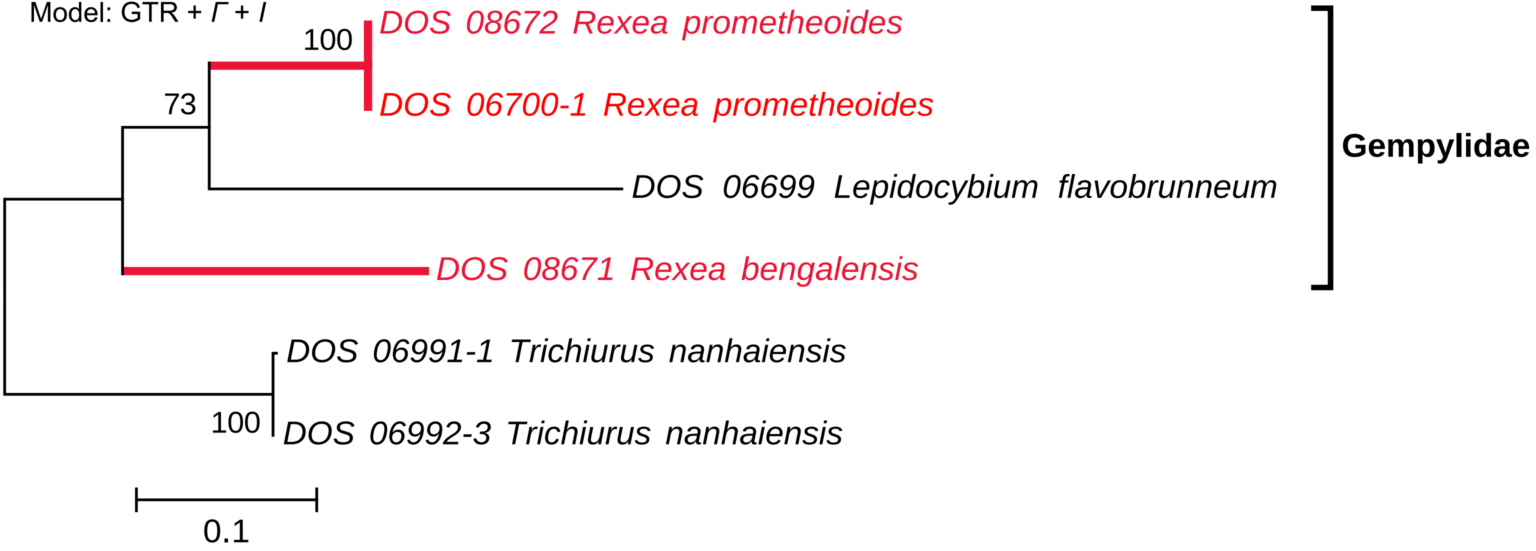

Fig. S3g Haemulidae & Lutjanidae
Model: GTR + Γ + I

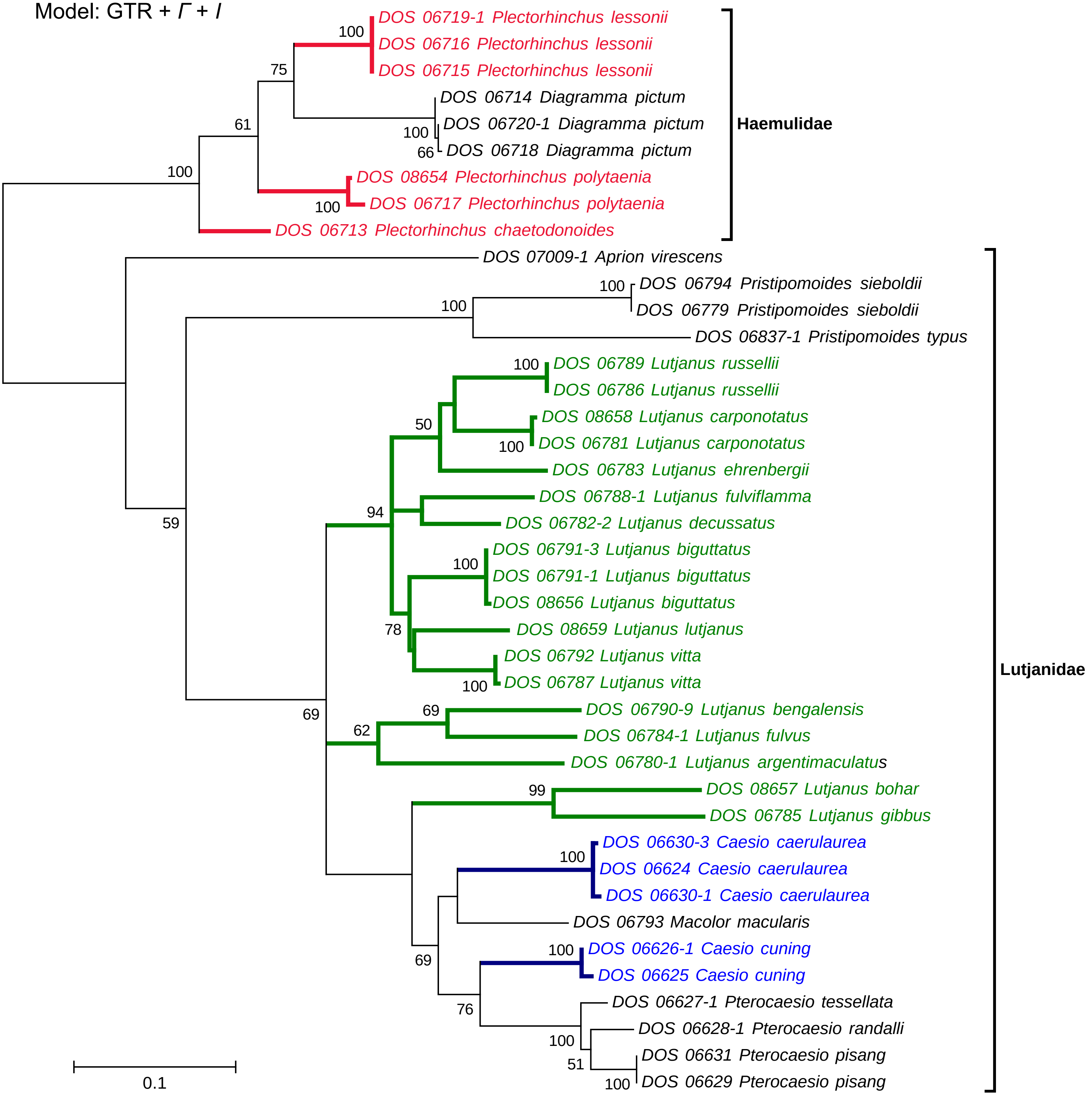

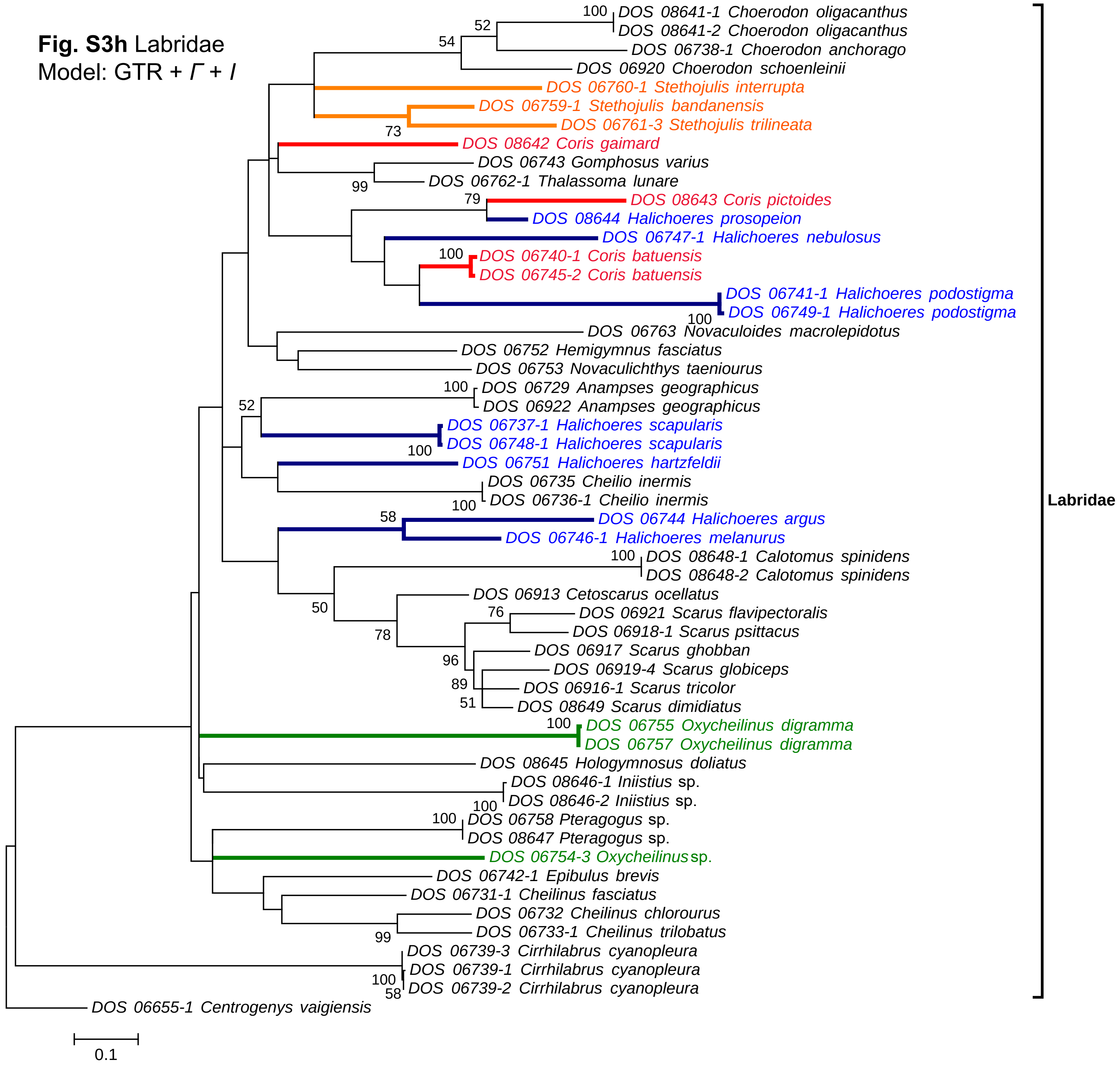

Fig. S3h Labridae
Model: GTR + Γ + I
DOS 08641-1 Choerodon oligacanthus
DOS 08641-2 Choerodon oligacanthus
DOS 06738-1 Choerodon anchorago
DOS 06920 Choerodon schoenleinii
DOS 06760-1 Stethojulis interrupta
DOS 06759-1 Stethojulis bandanensis
DOS 06761-3 Stethojulis trilineata
DOS 08642 Coris gaimard
DOS 06743 Gomphosus varius
DOS 06762-1 Thalassoma lunare
DOS 08643 Coris pictoides
DOS 08644 Halichoeres prosopeion
DOS 06747-1 Halichoeres nebulosus
DOS 06740-1 Coris batuensis
DOS 06745-2 Coris batuensis
DOS 06741-1 Halichoeres podostigma
DOS 06749-1 Halichoeres podostigma
DOS 06763 Novaculoides macrolepidotus
DOS 06752 Hemigymnus fasciatus
DOS 06753 Novaculichthys taeniourus
DOS 06729 Anampses geographicus
DOS 06922 Anampses geographicus
DOS 06737-1 Halichoeres scapularis
DOS 06748-1 Halichoeres scapularis
DOS 06751 Halichoeres hartzfeldii
DOS 06735 Cheilio inermis
DOS 06736-1 Cheilio inermis
DOS 06744 Halichoeres argus
DOS 06746-1 Halichoeres melanurus
DOS 08648-1 Calotomus spinidens
DOS 08648-2 Calotomus spinidens
DOS 06913 Cetoscarus ocellatus
DOS 06921 Scarus flavipectoralis
DOS 06918-1 Scarus psittacus
DOS 06917 Scarus ghobban
DOS 06919-4 Scarus globiceps
DOS 06916-1 Scarus tricolor
DOS 08649 Scarus dimidiatus
DOS 06755 Oxycheilinus digramma
DOS 06757 Oxycheilinus digramma
DOS 08645 Hologymnosus doliatus
DOS 08646-1 Iniistius sp.
DOS 08646-2 Iniistius sp.
DOS 06758 Pteragogus sp.
DOS 08647 Pteragogus sp.
DOS 06754-3 Oxycheilinus sp.
DOS 06742-1 Epibulus brevis
DOS 06731-1 Cheilinus fasciatus
DOS 06732 Cheilinus chlorourus
DOS 06733-1 Cheilinus trilobatus
DOS 06739-3 Cirrhilabrus cyanopleura
DOS 06739-1 Cirrhilabrus cyanopleura
DOS 06739-2 Cirrhilabrus cyanopleura
DOS 06655-1 Centrogenys vaigiensis
Labridae
0.1
100
52
54
73
99
79
100
100
52
100
100
58
100
50
76
78
96
89
51
100
100
100
99
100
58

Fig. S3i Muraenidae
Model: GTR + *Γ* + *I*

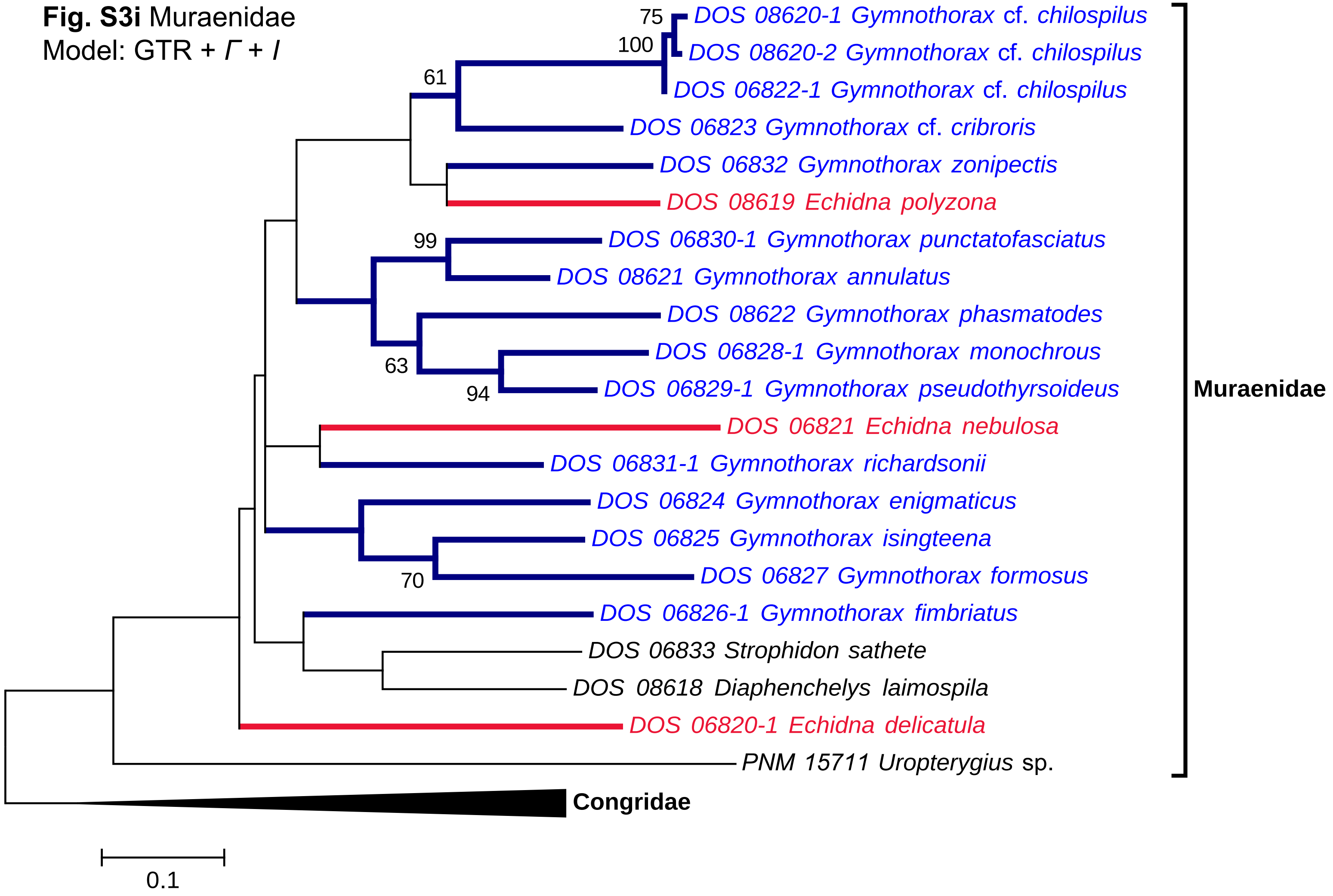

Fig. S3j Nomeidae
Model: GTR + *Γ*

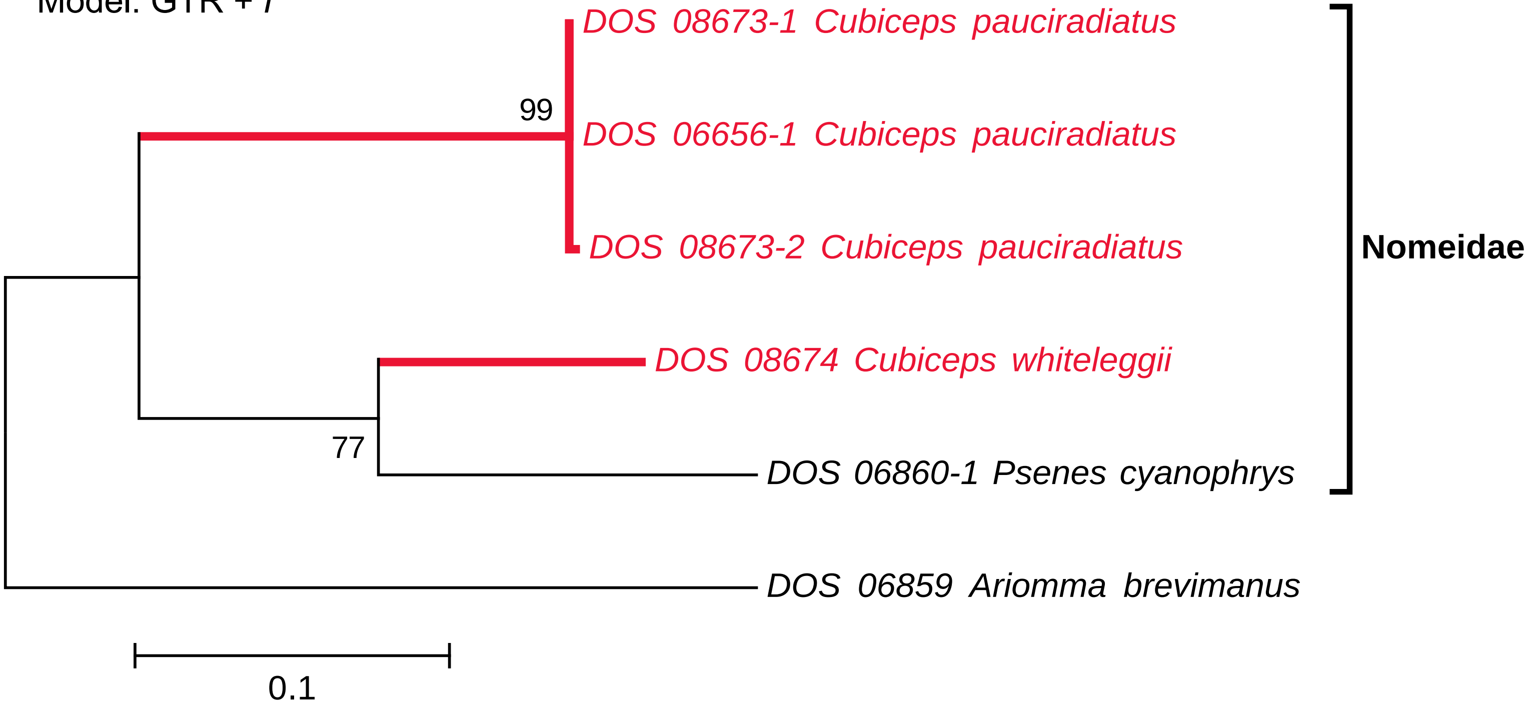

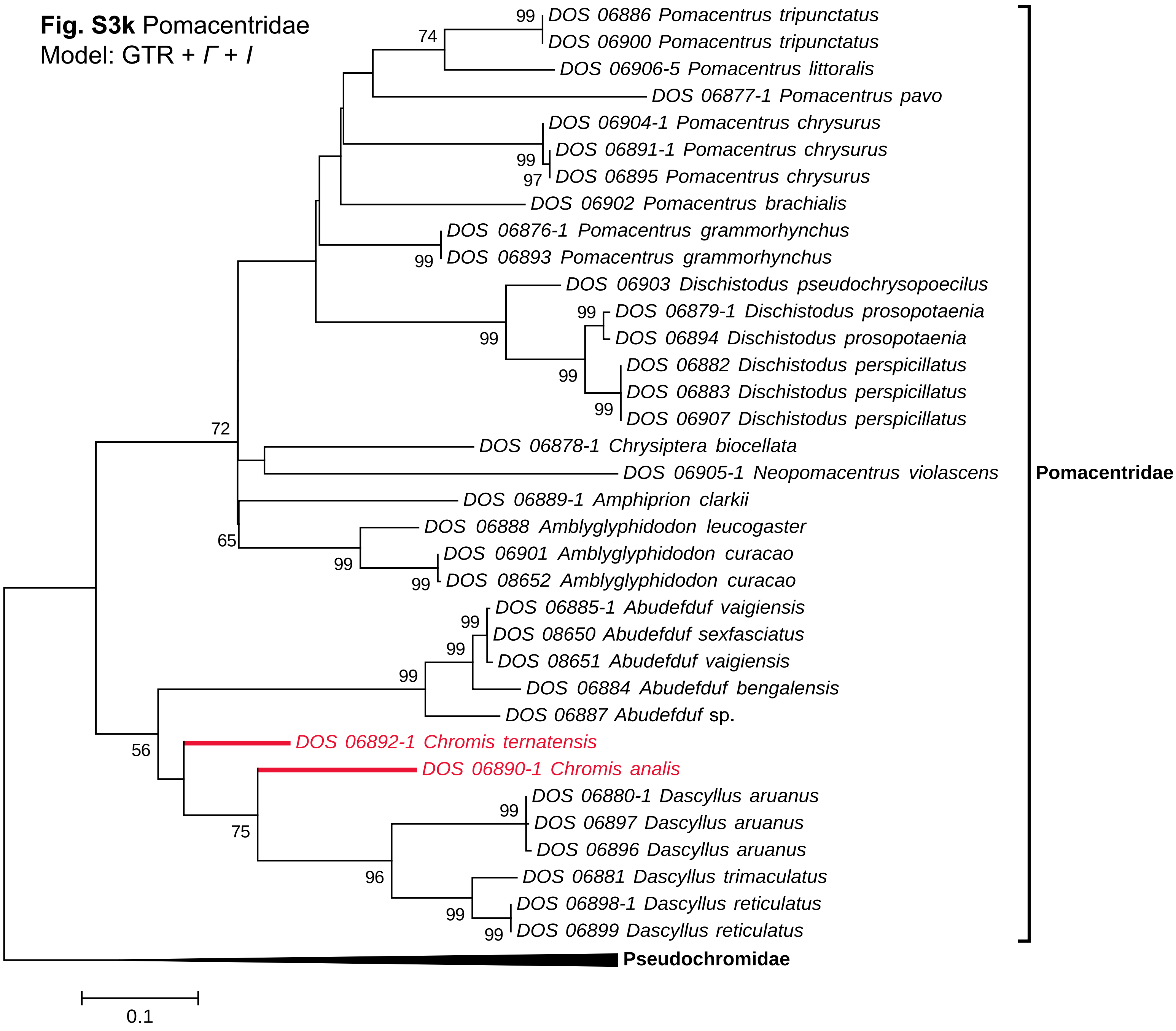

Fig. S3k Pomacentridae
Model: GTR + *Γ* + *I*
